# miR-30 Family miRNAs Mediate the Effect of Chronic Social Defeat Stress on Hippocampal Neurogenesis in Mouse Depression Model

**DOI:** 10.3389/fnmol.2019.00188

**Published:** 2019-08-08

**Authors:** Nitin Khandelwal, Sandeep Kumar Dey, Sumana Chakravarty, Arvind Kumar

**Affiliations:** ^1^Epigenetics and Neuropsychiatric Disorders’ Laboratory, CSIR-Centre for Cellular and Molecular Biology (CCMB), Hyderabad, India; ^2^Department of Cell Biology, CSIR-Indian Institute of Chemical Technology (IICT), Hyderabad, India; ^3^Division of Biological Sciences, Academy of Scientific and Innovative Research (AcSIR), Ghaziabad, India

**Keywords:** miRNAs, neurogenesis, depression, dentate gyrus, neurosphere, social defeat stress model

## Abstract

Depression is a debilitating psychiatric disorder with a high rate of relapse and a low rate of response to antidepressant treatment. There is a dearth of new antidepressants due to an incomplete understanding of the molecular mechanisms involved in its etiopathology. Chronic stress appears to be one of the foremost underlying causes of depression. Studies in animal models in the past decade have implicated epigenetic mechanisms in mediating the negative effects of chronic stressful events on the progression/manifestation of depression and other co-morbid neuropsychiatric disorders. However, non-coding RNAs, another layer of epigenetic regulation is relatively less studied in depression. Here, using the chronic social defeat stress (CSDS)-induced depression model, we hypothesized dysregulation in miRNA-mRNA networks in the neurogenic dentate gyrus (DG) region of male C57BL/6 mice. Among several dysregulated miRNAs identified *via* miRNA arrays, the most striking finding was the downregulation of miRNAs of the miR-30 family in stressed/defeated mice. To investigate miRNAs in the DG-resident neural stem/progenitor cells (NSCs/NPCs), we used the *in vitro* neurosphere culture, where proliferating NSCs/NPCs were subjected to differentiation. Among several differentially expressed miRNAs, we observed an upregulation of miR-30 family miRNAs upon differentiation. To search for the gene targets of these miRNAs, we performed gene arrays followed by bioinformatics analysis, miRNA manipulations and luciferase assays. Our results suggest that miR-30 family miRNAs mediate chronic stress-induced depression-like phenotype by altering hippocampal neurogenesis and neuroplasticity *via* controlling the epigenetic and transcription regulators such as *Mll3* and *Runx1*; and cell signaling regulators like *Socs3, Ppp3r1, Gpr125*, and *Nrp1*.

## Introduction

Depression is one of the major debilitating mental disorders that affect a significant portion of the global population. According to a recent report by the World Health Organization (WHO), almost 350 million people in the world are suffering from depression, and hence it has been termed as a “Hidden Burden” for humanity (Smith, 2014). Despite the heavy impact of depression on society and its higher prevalence, the mechanisms involved in the etiopathology of depression are not yet completely understood. One of the major reasons is its association with both genetic and epigenetic elements. Among the epigenetic factors, prime attention has been given to the changes in histone modifications and DNA methylation, which in turn, alter the expression of critical genes thus affecting the neural circuitry involved (Uchida et al., 2018; Kuehner et al., 2019). However, there is another layer of gene regulation, mediated by the non-coding RNAs, which is less explored in the context of depression and related mood disorders.

Out of the 90% of the transcribed eukaryotic genome, barely 2% codes for functional proteins, leaving a major portion as untranslated or non-coding RNAs (Spadaro and Bredy, 2012). Non-coding RNAs are, in fact, key drivers of gene expression because of their regulatory role in transcription and translation (Kaikkonen et al., 2011). Among the major classes of small regulatory ncRNAs, miRNAs are the most studied and ubiquitous. They have been shown to target almost 60% of the protein-coding mRNAs involved in diverse biological processes such as development, apoptosis, stem cell self-renewal and differentiation. Moreover, the ncRNAs are also implicated in etiology of a variety of diseases (Kloosterman and Plasterk, 2006; Gangaraju and Lin, 2009; Hayes et al., 2014).

In recent years, miRNAs have been demonstrated as one of the vital components involved in the regulation of several neuronal functions and brain-related disorders. They regulate genes involved in different stages of neurogenesis, viz. self-renewal, fate specification, migration, maturation, and functional integration of neurons into the existing brain circuitry (Shi et al., 2010). miR-132 has been reported to be involved in the integration of newborn neurons in the adult dentate gyrus (DG; Luikart et al., 2011), whereas miR-137 has been shown to modulate proliferation and differentiation of adult neural stem cells (NSCs; Szulwach et al., 2010). Increased expression of specific miRNAs is also observed in particular cell types. miR-124, the most abundant miRNA in the mouse brain, is found to be upregulated in differentiating and mature neurons (Deo et al., 2006), while miR-9, another neuron-specific miRNA, is majorly expressed in the precursor cells of the central nervous system (Deo et al., 2006). Numerous studies have been conducted to understand the function of miRNAs in synaptic plasticity, cognition, learning, and memory, and also in neuropsychiatric disorders (Bredy et al., 2011; Spadaro and Bredy, 2012), where clinical and preclinical studies have demonstrated their role in the progression of major depressive disorder (MDD), schizophrenia (Dwivedi, 2014; Caputo et al., 2015) and in depression models (Mouillet-Richard et al., 2012).

The recent investigations have established the role of miRNAs in many neuropsychiatric disorders, although their function in chronic stress-induced depression, anxiety, and related mood disorders has not yet understood. As these psychiatric disorders are known to be associated with attenuated adult neurogenesis, we attempted to uncover miRNAs which regulate adult neurogenesis in the hippocampal DG region of mouse brain, using chronic social defeat stress (CSDS) model, one of the most appropriate and well-known animal models for mimicking human depression (Golden et al., 2011). DG is one of the two major brain regions where neurogenesis occurs in the adult mammalian brain (Gage, 2000). There are reports of alteration in hippocampal neurogenesis upon exposure to CSDS (Lagace et al., 2010; Van Bokhoven et al., 2011; Hollis and Kabbaj, 2014), which can be due to the dysregulation of several gene regulatory mechanisms including non-coding RNAs.

Nevertheless, non-coding RNAs, particularly, microRNAs are least explored in the neurogenic DG in the etiopathology of depression. Regardless, there are a few reports that suggest changes in the levels of some miRNAs in proliferation and differentiation of DG NSCs/progenitor cells (NPCs; Liu and Zhao, 2009; Szulwach et al., 2010; Kawahara et al., 2012; Oliver and Mandyam, 2018). We hypothesized alteration in the proteins involved in miRNA biogenesis in DG after CSDS. Two of these proteins, Drosha and DGCR8, are required for the cleavage of primary miRNA to precursor miRNA in the nucleus (Lee et al., 2003; Han et al., 2004) while Dicer is required for the cytoplasmic processing (Bernstein et al., 2001). We found changes in the expression of Drosha, which gave us a good rationale to perform miRNA arrays on the cells from the DG regions of the defeated and control mice. As CSDS is known to affect neurogenesis, we performed similar, experiments on proliferating and differentiating NSCs/NPCs derived from the DG of adult mice. The arrays uncovered numerous dysregulated miRNAs, wherein the most striking finding was of miR-30 family miRNAs. We also performed mRNA arrays to identify the genes through which specific miRNA-mRNA network might mediate the effects of CSDS on hippocampal neurogenesis. We report a novel function of miR-30 miRNAs in altered hippocampal neurogenesis in response to chronic stress. We also indicate few of the target genes through which these specific miRNAs might function and bring about neural and behavioral changes leading to depression, anxiety and related mood disorders.

## Materials and Methods

### Animals

Age and weight-matched male C57BL/6-NCrl (will be called C57 henceforth) mice of 6–8 weeks age were used as experimental animals, whereas 6–8 months old, aggressive CD1 retired breeder males were used as resident mice or stressor in the social defeat experiment. All experiments were performed in accordance with standard protocols and procedures approved by the Institutional Animal Ethics Committee (IAEC) of the CSIR-Centre for Cellular and Molecular Biology (CCMB). Animals were maintained at 12 h/12 h light and dark cycle, 25°C ± 1°C temperature, 60% relative humidity and standard chow and water *ad libitum*.

### Experimental Design and Statistical Analysis

The number of samples in each group for all the experiments is mentioned in the legends of the respective figures. Statistical analyses were performed using two-sample Student’s *t*-test and analysis of variance (ANOVA) for the behavioral experiments and two-sample *t*-tests for the quantitative PCR (q-PCR) experiments. “*p*” values for these tests are presented in the legends. A value of “*p*” less than 0.05 is considered to be significant.

### Chronic Social Defeat Stress (CSDS)

The CSDS paradigm is a well-established protocol in mice for inducing depression-like phenotype, which is comparable to depression in humans (Berton et al., 2006; Krishnan et al., 2007). Mice were subjected to CSDS as described in earlier studies (Veeraiah et al., 2014; Pathak et al., 2017). Briefly, C57 male mice were housed in individual cages with the aggressor CD1 mouse, separated by a perforated Plexiglas barrier. Every day the experimental C57 mouse was allowed to interact with the aggressor mouse for 5 min, where it was attacked and subjected to agonistic behavior by the “resident” aggressor. For the remaining part of the day, experimental mouse remained in sensory contact with the aggressor mouse and continued to feel hostile environment. Throughout the defeat paradigm, each day, the experimental mice were exposed to different, unfamiliar CD1 aggressors to avoid any habituation. Pairs of C57 mice in a similar home cage separated by a Plexiglas barrier formed the non-stressed, control group. Mouse from the control group was also permitted to interact with another mouse of the same group daily for 5 min, where both of them showed friendly behavior. The CSDS protocol was followed for 10 days, and behavior tests were conducted on the 11th day. To avoid any elevated plus maze (EPM)-induced behavioral changes, mice were first subjected to the Social Interaction (SI) test in the morning and after 2 h, EPM test was conducted. Finally, after 2 h mice were sacrificed and their brain regions were micro dissected for molecular studies.

### Sucrose Preference Test (SPT)

Sucrose Preference Test (SPT) was carried out as discussed earlier (Veeraiah et al., 2014; Pathak et al., 2017). In brief, mice were acclimatized to drink water/sucrose from two bottles, prior to the start of the stress protocol. Thereafter, from the 1st day onwards, animals were given a two-bottle choice: plain water or 2% sucrose containing water, throughout the stress period, i.e., 10 days. The positions of the bottles were interchanged every day to eliminate the possibility of a location-induced bias. The volume of the consumed liquid was measured daily, and the sucrose preference was calculated in percentage as [(volume of 2% sucrose containing water consumed)/total volume of (plain water + 2% sucrose containing water) consumed] × 100. Sucrose preference for the last 4 days was taken as a measurement of anhedonia.

### Social Interaction (SI) Test

In this test, mice were assessed for their approach-avoidance behavior towards an unfamiliar social target as reported earlier (Krishnan et al., 2007; Veeraiah et al., 2014; Pathak et al., 2017), with minor modifications. Briefly, each mouse was introduced in an open arena, and its movement was tracked for two consecutive sessions of 5 min each. The open arena contained a small perforated Plexiglas cage, kept in the middle of the shorter side. Using Ethovision 3.1, the arena was divided virtually into the interaction zone (the area surrounding the small cage) and corner zones (two corners on the opposite side of the arena). During the first test session (“without a target”), the cage was kept empty; while during the next session (“with target”), an unfamiliar CD1 aggressor mouse was kept into the cage. The interaction ratio was calculated in percentage as follows:

{[Time spent by the mouse in the interaction zone in presence of the target (2nd session)/Total time spent in the interaction zone in the absence and presence of the target (1st session + 2nd session)] × 100}.

Mice showing interaction ratio less than 50% were considered truly defeated or depressed and were used for further studies.

### Elevated Plus Maze (EPM) Test

Mice were assessed for the anxiety-like behavior in the EPM test as described earlier (Krishnan et al., 2007; Pathak et al., 2017). EPM consisted of a plus-shaped wooden apparatus elevated at 100 cm above the ground, with two open and two closed arms and a central region at their intersection. Briefly, experimental mice were placed individually in the central region of the EPM and allowed to explore for 5 min, where time spent by mice in each arm was measured using Ethovision 3.1 (Noldus, Netherlands). Percent time spent in the open arms was calculated as follows:

{[Time spent by the mouse in open arms (s)/Total time of the trial, i.e., 300 s] × 100}.

Mice spending less time in the open arms as compared to the control mice were considered to have an anxiety-like phenotype.

### Tissue Collection

Twenty-four hours after the last stress, behavior tests were performed and mice were sacrificed by cervical dislocation. The brain was removed immediately, rinsed in ice-cold 1× PBS and 1 mm thick slices were obtained using a mouse brain matrix (Zivic rodent brain slicer matrix). The punches of the DG were taken out from the three consecutive 1 mm brain slices containing hippocampus (in between −1.0 to −4.0 position from Bregma) using a 16 gauge needle and collected in 1.5 ml tubes, snap-frozen in liquid N_2_ and stored at −80°C for further study.

### RNA Isolation and mRNA Expression Analysis by RT-qPCR

RNA was isolated using mirVana^TM^ miRNA Isolation Kit (*Ambion*), treated with DNase I (*New England Biolabs*) as per the manufacturer’s instructions. After quality check and quantification using Nanodrop, RNA was subjected to first-strand cDNA synthesis using SuperScript^TM^ III Reverse Transcriptase enzyme (*Invitrogen*). Upon dilution, cDNA samples were subjected to real-time quantitative PCR (RT-qPCR) using SYBR^®^ Premix Ex Taq^TM^ II (Tli RNaseH Plus) 2× buffer (*Takara*) and following primer pairs:

*Dcx*: forward (5′TCTGTTTCCCAGGCAATGCT3′), reverse (5′AAAGGGCCTGCTCTAACCAGT3′); *Dgcr8*: forward (5′GCGCGGGTGTGTAAGAATA3′), reverse (5′GCTTGACCATTTTGCTGCTCTC3′); *Dicer*: forward (5′GCTCAGTTCCCGTGTACAGTCA3′), reverse (5′TCGGATTGCCAGCAGTTAAGTT3′); *Drosha*: forward (5′GCCCGGCTTCAACAGTTACC’), reverse (5′TGGGGACCGCCTCTCATTCT3′); *G6pdh*: forward (5′CTGAGGAGTCGGAGCTGGATCTAA3′), reverse (5′AGTTCATCACTCCGGACAAAGTGC3′); *Gapdh*: forward (5′TGAAGTCGCAGGAGACAACCT3′), reverse (5′ATGGCCTTCCGTGTTCCTA3′); *Gfap*: forward (5′AGTGGCCACCAGTAACATGCAA3′), reverse (5′GCGATAGTCGTTAGCTTCGTGCTT3′); *Gpr125*: forward (5′TGGCTCCTTTAGACGTGCAGTT3′), reverse (5′ATTCTGCACGCTTCCTTCCACA3′); *Hmga2*: forward (5′AAGGAGCTCAAACCTCACCTCT3′), reverse (5′AAAGGCTGAGCTGGTTGTAGCA3′); *Ier3*: forward (5′GGCTCTGGTCCCGAAATTTTCA3′), reverse (5′CAATGTTGGGTTCCTCGGTTGGT3′); *Ifngr1*: forward (5′TTGTAGCCTCACCGCCTATCA3′), reverse (5′CGTGCTCTGCCATCTTTGTTTC3′); *Khnyn*: forward (5′CCACCGTGACATAACCGTCTTT3′), reverse (5′ACTCGTGAGGGTGTCAGGGAAA3′); *Lcn2*: forward (5′ACAATGTCACCTCCATCCTGGTCA3′), reverse (5′CCATGGCGAACTGGTTGTAGTCC3′); *Mll3*: forward (5′GGCTGAAGTCGTGACCTTTGA3′), reverse (5′CGGAATCTTGTGCTGGTCATCT3′); *Nestin*: forward (5′TTGAGTGGGGCTGCAGCTAATGTT3′), reverse (5′GGGGCATCTAAATGGTCAATCGCT3′); *Nrp1*: forward (5′GTCCACCTCAACAGCACAAAGA3′), reverse (5′TACAGCCACACAAGGAAGGGAA3′); *Omg*: forward (5′CTGAAATGCCTCGACAAAGCAC3′), reverse (5′AGCATGACCACAGCATTGAGCA3′); *Osmr*: forward (5′CGACATCAATGGCTCAGAGACAAA3′), reverse (5′CGTGCATCTGGAGTTGTGACCTT3′); *Ppp3r1*: forward (5′GCCTCATGAAGCCAACTAAGTG3′), reverse (5′TTAAAGAGCCAACCCCTTCCCT3′); *Ptgfrn*: forward (5′GATTCAGGGTCTTGGCAGTACA3′), reverse (5′AAGGGAAAGGTAGGTCCGATCA3′); *Ptk9*: forward (5′AGCTCGAAAGGTACCAGACAGT3′), reverse (5′AGCGATCCTTGGCTTTCGAACA3′); *Rcor1*: forward (5′GAATGGGAAGCAGAACATGGGA3′), reverse (5′TGCGTATCTTACGTCGAGGACA3′); *Runx1*: forward (5′AAGAACAGGGTGAGTCAGCCAT3′), reverse (5′CAACGGGTTGTGATCCTCAAGA3′); *Socs3*: forward (5′CGCTGGAACTTGTTTGCGCTTT3′), reverse (5′TTGGGCAGTGGGAGTGGTTATT3′); *Sox2*: forward (5′CAACGGCAGCTACAGCATGAT3′), reverse (5′TGCGAGTAGGACATGCTGTAGGT3′)*;Tbp*: forward (5′GAATTGTACCGCAGCTTCAAAA3′), reverse (5′AGTGCAATGGTCTTTAGGTCAAGTT3′).

PCR reactions were set up in triplicates in the MicroAmp^®^ Optical 384-Well Reaction Plate (Applied Biosystems) in ViiA^TM^ 7 Real-Time PCR System (Applied Biosystems, Foster City, CA, USA). Gapdh (Glyceraldehyde 3-phosphate dehydrogenase), Tbp (TATA-box binding protein) or G6pdh (Glucose 6-phosphate dehydrogenase) was used as housekeeping genes in different experiments, for normalization. Data were analyzed using the ΔΔCt method.

### miRNAs Expression Analysis by RT-qPCR

miRNAs were reverse transcribed using TaqMan MicroRNA Reverse Transcription Kit (Applied Biosystems, Foster City, CA, USA) as per the manufacturer’s instruction. In brief, 10 ng of the total RNA was used to synthesize cDNA for miRNAs using MultiScribe^TM^ Reverse Transcriptase and miRNA-specific RT primers (5×) by incubating at 16°C for 30 min, 42°C for 30 min and 85°C for 5 min. cDNA samples for specific miRNAs were subjected to the real-time qPCR using TaqMan 2× Universal PCR Master Mix and miRNA-specific TaqMan MicroRNA Assays (20x). Data were analyzed using the ΔΔCt method where snoRNA202 was used as housekeeping control for normalization.

### miRNA Microarray Using GeneChip miRNA Array v1.0/v3.0 (*Affymetrix*)

Total RNA was isolated using the mirVana RNA isolation kit (*Ambion*), which was labeled using the FlashTag Biotin HSR RNA labeling kit (*Genisphere*), as per the manufacturer’s instructions. In brief, 1 μg of total RNA was supplemented with RNA spike control oligos, diluted ATP mix, Poly A polymerase, and incubated at 37°C for Poly (A) tailing of the non-poly (A) containing RNAs. This was further incubated with FlashTag Biotin HSR Ligation mix and T4 DNA Ligase, followed by ending the reaction with HSR Stop Solution. The generated biotin-labeled RNA sample was mixed with the 2x hybridization mix, Formamide, DMSO, eukaryotic hybridization controls and oligonucleotide B2; incubated at 99°C and 45°C each for 5 min, and then injected in the GeneChip miRNA arrays (*Affymetrix*) that were then kept for hybridization at 48°C at 60 rpm in the hybridization oven (*Affy Hub oven 640*) for 16 h. Thereafter, arrays were washed and stained at Fluidics Station 450 using fluidics script FS450_0003 and scanned using GeneChip Scanner 3000 7G. The scanned *.CEL files were exported for the analysis.

For identifying the dysregulated miRNAs in DG samples from defeated mice, miRNA v1.0 arrays were used, which were analyzed using miRNA QC Tool version 1.0.33.0, where the *.CEL files were uploaded followed by background adjustment (by BC-CG Adjust), normalization (quantile) and finally the generation of summarization file (by median polish). The summarization data provided the expression data in the log intensity values for all the miRNA species. We have selected only the mouse miRNAs and analyzed them to identify miRNAs with a fold change ≥1.2 in defeated samples, as compared to the controls. The miRNA microarray data have been submitted to the GEO with accession number GSE132821^1^.

On the other hand, identification of differentially regulated miRNAs in proliferating neurospheres and differentiating cells was performed using miRNA v3.0 arrays, which were analyzed using Expression Console software (*Affymetrix*). In this case, *.CEL files were uploaded and using “MicroRNA Arrays RMA + DABG” workflow, summarization file was generated. We have selected the differentially regulated miRNAs in both the conditions with a change ≥ 1.2-fold and *p*-value ≤ 0.05 for further studies. The miRNA microarray data have been submitted to the GEO with accession number GSE132822^2^.

### mRNA Array Using GeneChip Mouse Gene 1.0 ST Array (*Affymetrix*)

Total RNA was isolated using mirVana RNA isolation kit (*Ambion*), which was then reverse transcribed to sense strand cDNA using WT Expression kit (*Ambion*), as per manufacturer’s instructions. In brief, for each sample, 200 ng of total RNA was added with diluted poly-A controls and subjected to reverse transcription for the synthesis of first-strand cDNA followed by second-strand cDNA synthesis. Double-stranded cDNA was incubated at 42°C for 16 h with RNA polymerase to generate antisense RNA (aRNA) by *in vitro* transcription. aRNA was cleaned up and purified using nucleic acid binding magnetic beads, and further subjected to synthesize 2nd cycle cDNA, which was again cleaned up and purified after RNase H-mediated hydrolysis of aRNA. Eluted single-stranded sense cDNA was then fragmented and labeled, and prepared for hybridization using the GeneChip WT Terminal Labeling and Hybridization kit (*Affymetrix*). The appropriate volume of the hybridization mix was filled in the probe array cartridge and incubated at 45°C for 16 h at 60 rpm in the hybridization oven (*Affy Hub oven 640*). Post-hybridization, array cartridge was fixed on the GeneChip Fluidics Station (*Affymetrix*) and washed and stained using the GeneChip wash and stain kit (*Affymetrix*). Finally, the array was scanned on the GeneChip Scanner 3000 7G (*Affymetrix*) and the data was exported in the form of *.CEL files.

The raw data were processed with standard RMA and DABG filtering options using AltAnalyze v2.0.7 software (Emig et al., 2010). Briefly, the Affymetrix *.CEL files were Quantile normalized, median-polish summarized using Affymetrix Power Tools (APTs) provided through AltAnalyze (Emig et al., 2010). DABG *p*-value of 0.05 was considered for a probeset to be expressed. Differential gene-level expression analysis was performed for all pair-wise comparisons using the moderated unpaired *t*-test. For gene-level expression, 1.2-fold change with a *p*-value of 0.05 was considered to be significant. The mRNA microarray data have been submitted to the GEO with accession number GSE132819^3^.

### mRNA Array Using MouseWG-6 v2.0 Expression BeadChip Kit (*Illumina*)

Pure quality total RNA was isolated by mirVana RNA isolation kit (*Ambion*) and then labeled by TargetAmp^TM^-Nano Labeling Kit for Illumina^®^ Expression BeadChip^®^ (*Illumina*), according to manufacturer’s protocol. In brief, poly (A) RNA present in the total RNA (300 ng) was reverse transcribed to first-strand cDNA using the SuperScript III reverse transcriptase enzyme and oligo (dT) primer containing T7 RNA Polymerase promoter sequence at its 5′ end. The first-strand cDNA was converted to double-stranded cDNA by DNA polymerase enzyme, followed by *in vitro* transcription using T7 RNA polymerase and biotin-labeled UTPs along with unlabeled NTPs to generate biotin-labeled antisense RNA. Biotin-aRNA was purified using RNeasy MinElute Cleanup Kit (*Qiagen*), as per manufacturer’s instructions and quantified.

Purified Biotin-aRNA (1.5 μg) was mixed with Hyb mix buffer, and loaded on the BeadChip. Six samples were loaded separately on one BeadChip, which was then fixed in the Hyb chamber and kept in a hybridization oven (*Illumina 6527*) for 16 h at 58°C with mild shaking. Post-hybridization, BeadChip was washed by high-temperature wash buffer, 100% ethanol and wash E1BC buffer, and then incubated with Block E1BC buffer, followed by staining with streptavidin-cy3 after diluting it in Block E1 buffer at 1:2,000 dilution for 10 min each. Finally, BeadChip was dried by low-speed centrifugation after a wash with E1BC buffer and scanned on the HiScan system (*Illumina*) using the BeadChip specific *.dmap files. Image data files (*.idat) generated from the scanner were imported to the GenomeStudio software (*Illumina*), and analyzed using the Binary Manifest Files (*.bgx) in the Gene Expression module. Individual samples were assigned to their respective groups and data were exported after the background subtraction in the GeneSpring GX readable format (*.txt).

The *.txt files were loaded in the GeneSpring 12.0 software (*Agilent*) for the analysis and visualization of the data. This process includes data normalization, hierarchical cluster analysis (“Pearson Absolute” distance metric and “Average” linkage rule), and statistical analysis. Genes with a change of ≥1.5-fold (late differentiation vs. proliferation; early differentiation vs. proliferation and late differentiation vs. early differentiation) and mean differences (*p* < 0.05) were used for further analysis. The mRNA microarray data have been submitted to the GEO with accession number GSE132820^4^.

### Maintenance of Mammalian Cell Lines

HEK293 cells and mouse glioma cells GL261 were grown as monolayers in culture flasks and dishes in Dulbecco’s minimum essential medium (DMEM, *Gibco*), supplemented with 10% Fetal Bovine Serum (FBS, *Gibco*). These cells were maintained at 37°C in a humidified, 5% CO_2_ incubator and passaged and expanded at 75%–80% confluency.

### Transient Transfection of miRNA Mimics and Inhibitors Using Lipofectamine 2000 in GL261 Cell Lines

GL261 cells were transfected with miRNA mimics and inhibitors (*mirVana*) using Lipofectamine 2000 (*Invitrogen*) according to the manufacturer’s instructions. Cells were transfected at 60% confluency in serum-free medium in a 24 well plate, where 10 picomoles of miRNA mimics were added with 1 μl of Lipofectamine 2000 and 20 picomoles of miRNA inhibitors were added with 1.5 μl of Lipofectamine 2000 in one well. Growth medium was replaced with serum-containing medium after 5–6 h, and cells were collected 48 h post-transfection. Negative mimics and inhibitors (*mirVana*) were used as negative controls.

### Luciferase Assay

To validate direct gene targets of the selected miRNAs, 3′UTR sequences (containing seed sequences) of few of the target genes predicted by bioinformatics analysis were cloned in pmir-GLO Dual-Luciferase miRNA Target Expression vector (*Promega*), downstream of the firefly luciferase gene. These UTRs were cloned under the PGK promoter using double restriction digestion with either NheI and XhoI or PmeI and NheI, as per manufacturer’s instructions. 3′UTRs were amplified using following primer pairs: *Mll3*: forward (5′TAGGCTAGCGTCACAGAATGGTCCAGCACTT3′), reverse (5′CTAGCTCGAGACAACCAAACTCGGCAGGACAA3′); *Knhyn*: forward (5′TAGGCTAGCTCAACCTGAGGAAGGACCACGAA3′), reverse (5′CTAGCTCGAGTCAGGGACTTGCTACATGAACCCA3′); *Omg*: forward (5′TAGGCTAGCAACGTGCCCCTATCTAACCAGT3′), reverse (5′CTAGCTCGAGGAGTGGCTCACGTTTCATTCACCT3′); *Ppp3r1*: forward (5′TAGGCTAGCAGTTCAGTGCTGTTGCCA3′), reverse (5′CTAGCTCGAGCTCTAAAGATTTCACAACATGACA3′); *Rcor1*: forward (5′GTTTAAATCACCTAGCCATCTGCATCACA3′), reverse (5′TAGGCTAGCTCCACATGGTTAGTGCAGTCTC3′); *Socs3*: forward (5′TAGGCTAGCAAGGGAGGCAGATCAACAGATG3′), reverse (5′CTAGCTCGAGGCAAAGTCTGAGTTGAACTGGG3′). All the forward primers contain restriction site for NheI in their sequence while the reverse primers contain site for XhoI, except for *Rcor1* which contains site for PmeI in the forward primer whereas for NheI in the reverse primer.

The 3′UTR clones were transfected (200 ng per well) along with the respective miRNA mimics (20 picomoles) and inhibitors (20 picomoles) in HEK293 cells using Lipofectamine 2000 (1.5 μl per well), in 24 well plates. After 36 h, transfected cells were washed once with 1× PBS, and then lysed in 100 μl of 1x passive lysis buffer for 15 min at room temperature on a rocker platform, and collected in 1.5 ml tubes. The lysate was centrifuged, and 10 μl of the lysate supernatant was added to one well of a 96-well Luminometry plate. Luciferase Assay Reagent (LAR II, 30 μl) was mixed with the lysate supernatant and luminescence was measured using a Luminometer. This was followed by mixing of 30 μl of Stop and Glo reagent and measuring the luminescence again.

The first readings provided luminescence by the Firefly luciferase, whereas the second ones by the *Renilla* Luciferase. The readings for all the negative and positive controls for the mimic, inhibitor and 3′UTR clones were taken and used in analyzing the data. For the validation of any gene as the direct target of a specific miRNA, the experiment was repeated thrice, each time in triplicates. Final data were analyzed by normalizing the Firefly readings with the *Renilla* readings and comparing the data from positive and negative mimics’ treatment.

### Primary Neural Stem/Progenitor Cell (NSC/NPC) Culture From the DG of Adult Mice

For the primary culture of NSCs/NPCs from the DG of adult mice, the protocol described earlier was followed (Reynolds and Weiss, 1992). Briefly, mice were sacrificed by cervical dislocation, brain tissue was dissected out in cold MEM media (*HiMedia*), minced and dissociated using activated papain (*Worthington*, LK003178) at room temperature for 30 min. Papain was activated by reconstituting it in 5 ml of HBSS (*Gibco*) with 50 μl of 1 mg/ml DNase I enzyme (*Sigma*), followed by incubation at 37°C for 30 min. Papain activity was then arrested using 10% serum containing DMEM/F12 medium (*Gibco*). Cells were further passed through 40 μm cell strainers to obtain a single cell suspension, followed by pelleting at 700 g for 5 min. Pellet was washed twice with incomplete DMEM/F12 medium, and finally resuspended in 10 ml complete proliferative medium supplemented with 1 ml of mouse proliferative supplement (10×, *Stemcell Technologies*, 05702), 100 μl of Penicillin-Streptomycin Solution (100×, *Gibco*), 200 μl of BSA (1% in water, *Sigma*), 10 μl of bFGF (10 μg/ml in MEM, *Gibco*), 10 μl of EGF (20 μg/ml in MEM, *Calbiochem*), 10 μl of heparin (0.1% in water, *Sigma*) and remaining of Neurocult basal medium (*Stemcell Technologies*, 05700). Cells were further seeded in T-75 flasks and allowed to grow into neurospheres in a 5% CO_2_ incubator at 37°C for 8–9 days.

For passaging, floating neurospheres were pelleted and dissociated into a single cell suspension by treating with Accutase (*Gibco*) at 37°C for 5 min, followed by trituration. The accutase activity was arrested by NeuroCult basal medium and cells were counted after passing through a 40 μm cell strainer. Cells were pelleted at 700 g for 5 min and resuspended in an appropriate volume of complete NeuroCult proliferation medium. Cells were either transferred to the flasks for the formation of neurospheres (20,000 cells/ml) or grown on coated cell culture dishes as a monolayer (100,000 cells/ml). The coating was done with Poly-D-Lysine (*Sigma*, 0.1 mg/ml) and Laminin, mouse purified (*Millipore*, 10 μg/ml), for 2 h each at 37°C, followed by washing with 1× PBS.

### Differentiation of the NSCs/NPCs

After culturing NSCs/NPCs for 2–3 days in the proliferation conditions on coated dishes, proliferation medium was replaced with the differentiation medium [10 ml of differentiation medium contains 1 ml of NeuroCult^TM^ differentiation supplement (mouse; 10×, *Stemcell Technologies*, 05703), 100 μl of Penicillin-Streptomycin Solution (100×, *Gibco*), 200 μl of BSA (1%, *Sigma*) and remaining of NeuroCult^TM^ basal medium]. The differentiated cells were collected at different time-points during differentiation—the early differentiation stage (at day 2 of differentiation) and late differentiation stage (at day 7 of differentiation), for various experiments.

## Results

### Chronic Social Defeat Stress (CSDS) Induces Depression and Anxiety-Like Phenotype in the Mouse

**Figure 1:**
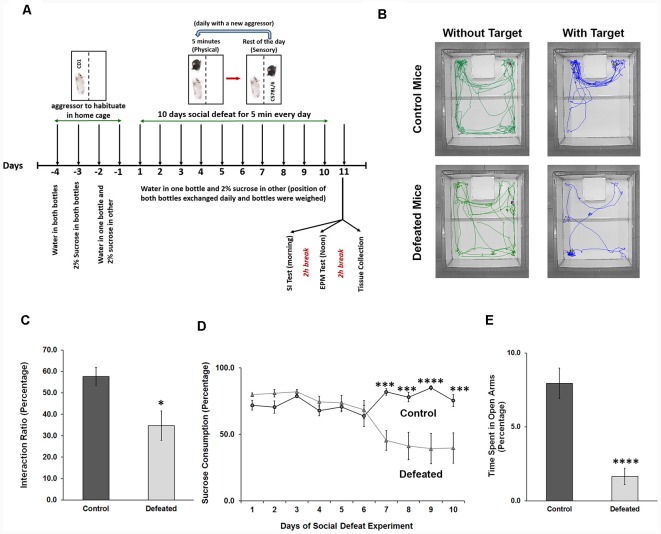
Chronic social defeat stress (CSDS) induces anxiety and depression-like phenotype in mice. **(A)** Representation of the timeline of the experiments, which shows the stress paradigm and the behavior tests conducted.** (B)** The movement tracks of both control and defeated C57BL/6 mouse in presence and absence of target CD1 mouse are shown, where control mouse spends more time in the interaction zone in presence of the target, while stressed mouse spends significantly lesser time. **(C)** Interaction ratio is calculated for mice from both the groups in percentage, which is ratio of time spent in the interaction zone in presence of a target to the total time spent in the interaction zone (in presence + in absence), and the average is being plotted, *n* = 9 mice in each group, two-sample *t*-test, *p* = 0.011, data represented as Mean ± standard error of the mean (SEM). **(D)** Percentage sucrose consumption is calculated daily for every mouse and daily average consumption for both the groups is plotted, *n* = 12 mice in each group, two-way analysis of variance (ANOVA) with repeated measures, *p* = 0.0002, 0.0002, <0.0001 and 0.0003, respectively, data represented as Mean ± SEM. **(E)** Percentage of time spent in open arms of the elevated plus maze (EPM) for both the groups, which is the ratio of time in the open arms to the total time (300 s) of the test and the average is being plotted. *n* = 10 mice in each group, two-sample *t*-test, *p* = 0.0001, data represented as Mean ± SEM. *p ≤ 0.05, ***p ≤ 0.001, ****p ≤ 0.0001.

CSDS for 10 days is a well-established aggressive stress model, which results in depression and anxiety-like phenotype in experimental C57BL/6 mouse (Figure 1A). After 24 h of last defeat stress (11th Day), 60%–70% of the experimental mice showed an interaction ratio of less than 50% (signifying the development of social avoidance, a hallmark of depression; Figures 1B,C). CSDS led to a reduced intake of 2% sucrose solution as compared to plain water in the last 4 days of the paradigm (indicating the development of anhedonia, another hallmark of depression; Figure 1D). Mice with social avoidance and anhedonia were considered as truly defeated. Correspondingly, defeated mice spent significantly lesser time in open arms of the EPM (Figure 1E) than the control mice, which signified elevated anxiety-like phenotype. Mice showing both the depression and anxiety-like conditions (will be referred to as depressed), were used in subsequent experiments.

### CSDS Induced Downregulation of Drosha, a Key Protein of miRNA Biogenesis, and Dysregulation of Numerous miRNAs in the Dentate Gyrus of Defeated Mice

**Figure 2:**
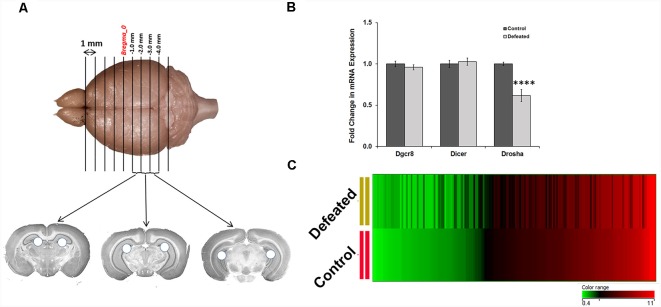
CSDS causes significant downregulation in the expression of Drosha and dysregulation in the expression of several miRNAs in the dentate gyrus (DG) of defeated mice. **(A)** Three 1 mm thick slices of mouse brain from the hippocampus were taken in between −1.0 mm to −4.0 mm position from the Bregma, where holes represent the dissected DG punches taken, using a 16-gauge needle. **(B)** Fold change in mRNA expression of genes involved in miRNA biogenesis in the DG of defeated mice, compared to the controls; geometric mean (GM) of Ct values of *Gapdh* and *Tbp* was used for normalization; *n* = 8 samples in each group, two-sample *t*-test, *p* = 0.0001, data represented as Mean ± SEM. **(C)** Heat map of miRNAs getting either upregulated or downregulated in the DG of defeated mice as compared to the controls, with ≥1.2-fold difference; *n* = 4 samples in each group, where every sample was prepared by pooling equal amount of RNA from DG of three individual mice. The heat map depicts the corresponding expression levels of miRNAs in the defeated group when they are sorted in the ascending order of their expression in the control group. ****p ≤ 0.0001.

To comprehend the role of miRNAs in DG (dissected as described in the “Materials and Methods” section and as shown in Figure 2A) upon CSDS exposure, we first assayed mRNA expression of genes coding for *Dgcr8*, Dicer, and Drosha using RT-qPCR, and the data showed significantly reduced transcript levels of Drosha in the DG of defeated mice, compared to the controls (Figure 2B). Downregulation of Drosha in DG might affect the downstream production of mature miRNAs. Therefore, we performed a miRNA array using GeneChip miRNA array v1.0 (*Affymetrix*), where 188 miRNAs (change ≥1.2-fold) were found differentially regulated; 106 miRNAs were upregulated, while 82 were downregulated in the DG of defeated mice, compared to control mice (Figure 2C). The dysregulated miRNAs are listed in Supplementary Tables S1, S2. Most of the dysregulated miRNAs showed a difference of 1.2–1.5-fold, which is sufficient to cause a significant impact on the downstream gene regulation in the brain. Additionally, few of the dysregulated miRNAs even exhibited a differential regulation of more than 1.5-fold (17 upregulated and 27 downregulated).

### CSDS Led to Downregulation of miR-30 Family miRNAs and Dysregulation of Hundreds of mRNAs in the DG of Defeated Mice

**Figure 3:**
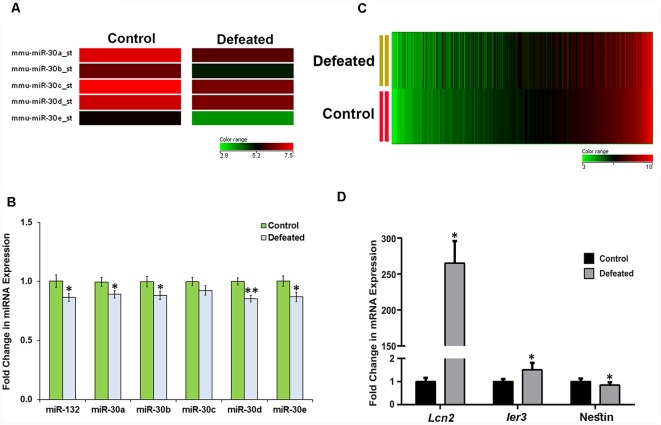
CSDS causes downregulation of miR-30 family miRNAs and dysregulation of several genes in the DG of defeated mice. **(A)** Heat map showing differential expression of five miR-30 family members in the DG of defeated mice as compared to the controls. **(B)** Validation of miR-30 miRNAs and miR-132 by TaqMan RT-qPCR in the individual DG samples from both control and defeated group; snoRNA-202 was used as a normalization control; *n* = 12-samples in each group, two-sample *t*-test, *p* = 0.039, 0.045, 0.046, 0.174, 0.0017 and 0.036, respectively, data represented as Mean ± SEM. **(C)** Heat map of differentially regulated genes in the DG of defeated mice when the genes are sorted in the ascending order of their expression in the control group. The heat map shows the dysregulated genes in the defeated vs. control group with ≥ 1.2-fold difference and *p* ≤ 0.05, *n* = 3 samples in each group, where every sample was prepared by pooling equal amount of RNA from DG of three individual mice. **(D)** mRNA expression profile of *Lcn2* and *Ier3* in the defeated mice as compared to the controls by RT-qPCR. Both the genes were found upregulated in the array data and showed a similar profile upon validation. Likewise, Nestin also showed mRNA expression in the individual samples similar to that of gene array, upon validation by RT-qPCR. Ct values of *Gapdh* were used for normalization; *n* = 9 mice in each group, two-sample *t*-test, *p* = 0.05, 0.04 and 0.03, respectively, data is represented as Mean ± SEM. *p ≤ 0.05, **p ≤ 0.01.

Among the attenuated miRNAs in our array dataset, interestingly, a significant reduction was observed (change ≥1.5-fold) in the expression levels of all the five members (a–e) of a well-conserved miR-30 miRNA family (Figure 3A). We validated their expression levels in individual samples by RT-qPCR. Indeed, there was a significant downregulation in the expression of all miR-30 miRNAs except miR-30c, although the trend showed a decrease here as well (Figure 3B).

To explore in detail the gene targets through which miR-30 family might act to alter the neural and behavioral response to CSDS, we performed a gene array analysis using Gene 1.0 ST array (*Affymetrix*) on the same DG samples that were used for miRNA array. Data analysis led us to identify 959 dysregulated genes, in which 519 genes were upregulated while 440 genes were downregulated (change ≥1.2-fold, *p* ≤ 0.05) in the DG of CSDS mice, compared to controls (Figure 3C). The dysregulated genes are listed in Supplementary Tables S3, S4.

Several genes involved in immune response, defense response, neuro-inflammatory response and responses associated with depression phenotype in animal models were found upregulated. One of the genes, lipocallin-2 (*Lcn2*) which we found as highly expressed in defeated mice, has already been reported to be upregulated in mouse hippocampus in psychosocial stress and involved in neuronal excitability and anxiety in response to stress (Mucha et al., 2011). Similarly, we also found upregulation in immediate early response-3 (*Ier3*) transcript, which has previously been shown to go up under cellular stress, inflammation and tumorigenesis (Arlt and Schäfer, 2011). The expression profile of these genes was validated in individual samples by RT-qPCR (Figure 3D). On the other hand, the downregulated genes comprised of bone morphogenetic protein 4 (*Bmp4*), fibroblast growth factor receptor 2 (*Fgfr2*), GLI-Kruppel family member GLI2 (*Gli2*), *Nestin*, SRY-box containing gene 2 (*Sox2*), which are involved in cytoskeletal organization and differentiation of neurons and are reportedly affected in depression models. We validated downregulation of *Nestin*, one of the markers of NSCs/NPCs’ proliferation by RT-qPCR in individual samples (Figure 3D).

### Differentiation of Adult Mouse DG Derived NSCs/NPCs in Culture led to the Dysregulation of Hundreds of miRNAs Including miR-30 Family Members, and Thousands of Genes

**Figure 4:**
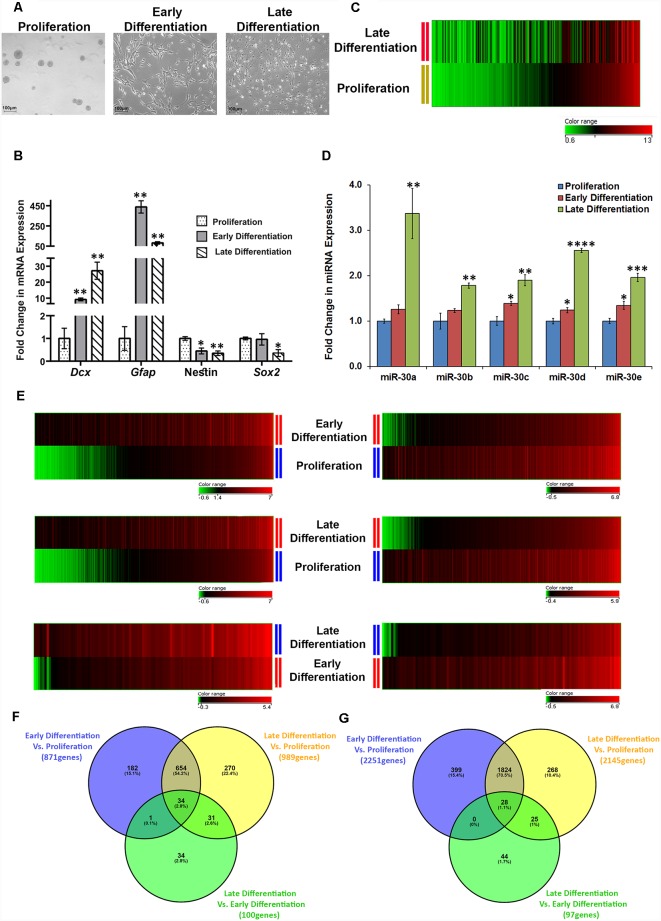
Differentiation of adult mouse DG derived neurospheres causes differential regulation of numerous miRNAs including miR-30 miRNAs and alteration in expression of thousands of genes. **(A)** Representative images of proliferating neurospheres, cells from an early stage of differentiation (early differentiation, day 2) and cells from a later stage of differentiation (late differentiation, day 7). **(B)** mRNA expression of *Dcx, Gfap* (markers for neuronal and glial differentiation, respectively), Nestin [neural stem cell (NSC) marker] and *Sox2* (proliferation marker) in proliferation, and differentiation (early and late) stages of adult DG derived NSCs/NPCs. The mRNA expression pattern of these genes corroborates with different stages of the NSC/NPC culture. Geometrical Mean of Ct values of *G6pdh* and *Tbp* was used for normalization; *n* = 3 samples in each group, two-sample *t*-test, *p* = 0.004, 0.002, 0.023 and 0.92, respectively for early differentiation (day 2) samples, *p* = 0.008, 0.002, 0.006 and 0.05, respectively for late differentiation (day 7) samples; data is represented as Mean ± SEM. **(C)** Heat map for the corresponding expression of miRNAs in cells from the late stage of differentiation when miRNAs are sorted in the ascending order of their expression in proliferating neurospheres. Dysregulated miRNAs in late differentiation vs. proliferation with ≥1.2-fold difference and *p* ≤ 0.05 are shown where *n* = 3 samples in proliferation and *n* = 2 samples in late differentiation (could not collect data from one of the miRNA arrays due to the technical issue). **(D)** Fold change in miRNA expression of miR-30 miRNAs in cells from early as well as late differentiation stage, as compared to the proliferating neurospheres; *n* = 3 samples in each group, two-sample *t*-test, *p* = 0.07, 0.27, 0.02, 0.05 and 0.03, respectively for early differentiation samples, *p* = 0.01, 0.01, 0.004, 0.0001 and 0.001, respectively for late differentiation samples, data is represented as Mean ± SEM. **(E)** The left panels demonstrate the heat maps showing upregulated genes in cells from early differentiation stage vs. proliferating neurospheres, late differentiation stage vs. proliferating neurospheres and late differentiation stage vs. early differentiation stage, respectively (≥1.5-fold change, *p* ≤ 0.05). The right panels show the downregulated genes for the same comparison groups (≥1.5-fold change, *p* ≤ 0.05). **(F)** Venn diagram showing common and exclusively upregulated genes when the three comparison groups are paired with each other. **(G)** Venn diagram showing common and exclusively downregulated genes when the three comparison groups are paired with each other. *p ≤ 0.05, **p ≤ 0.01, ***p ≤ 0.001, ****p ≤ 0.0001.

The adverse effect of CSDS on the DG neurogenesis is expected to affect the properties of the resident NSCs/NPCs. As we could identify a miRNA family implicated in the defeat-induced changes in mouse DG, it was imperative to explore the involvement of this miRNA family along with others in the proliferation/differentiation of the NSCs/NPCs. Here, we used the neurosphere culture system, a well-established *ex vivo* model to study neurogenesis. We collected samples from three stages in culture, proliferating neurospheres, differentiating NSCs/NPCs from an early time point (day 2) and that of later time point (day 7; Figure 4A). The mRNA expression levels of a few of the key markers (*Dcx, Gfap*, *Nestin*, and *Sox2*) were checked that corroborated with their respective stages (Figure 4B). The analysis of GeneChip miRNA array v3.0 (*Affymetrix*) data resulted in the identification of 105 upregulated and 120 downregulated miRNAs (change ≥1.2-fold, *p* ≤ 0.05; Figure 4C; listed in Supplementary Tables S5, S6) in the late stage of differentiation (late differentiation), as compared to the proliferating phase (proliferation). The expression of all the members of miR-30 family was found to be altered here too, and they showed upregulation in the differentiating cells as compared to the proliferating NSCs/NPCs, which was validated by RT-qPCR. There was a 15%–20% increase in their expression upon early differentiation (day 2), which further increased to 200%–300% in late differentiation (day 7; Figure 4D).

To identify the gene targets of miR-30 miRNAs, a gene array was performed using Illumina^®^ Expression BeadChip^®^ (*Illumina*) on the samples from three different stages. The analysis revealed an upregulation of 871 and 989 genes in early and late differentiation, respectively, as compared to the proliferating stage (change ≥1.5-fold and *p* ≤ 0.05), where 688 genes were common in both the differentiation stages. Thirty-four out of 688 genes, showed an even higher level of upregulation when the cells were differentiated for 7 days (Figures 4E,F). Further analysis revealed that 182, 270 and 34 genes were upregulated exclusively in early differentiation vs. proliferation, late differentiation vs. proliferation, and late differentiation vs. early differentiation, respectively. The list of upregulated mRNAs in different comparisons is shown in Supplementary Tables S7, S9 and S11.

The number of attenuated genes in differentiation was comparatively high, as 2,251 genes in the early differentiation stage and 2,145 genes in late differentiation stage were found downregulated as compared to the proliferating NSCs/NPCs (change ≥1.5-fold and *p* ≤ 0.05). Among the overlapping 1,852 downregulated genes between early and late differentiation, the attenuation in the expression of 28 genes was much higher in the later stage of differentiation (Figures 4E,G). Moreover, 399, 268 and 44 genes were found to be downregulated exclusively in early differentiation vs. proliferation, late differentiation vs. proliferation and late differentiation vs. early proliferation, respectively. The list of downregulated mRNAs in different comparisons is shown in Supplementary Tables S8, S10 and S12.

### Bioinformatics Analysis and Validation Experiments Yielded a Number of Gene Targets of miR-30 miRNAs

As miR-30 miRNAs were downregulated in the DG of defeated mice and upregulated in late differentiation stage in the NSC/NPC culture, a dataset of upregulated mRNAs in defeated DG samples and downregulated mRNAs in late differentiation stage were used to find the probable gene targets of these miRNAs.

Using four of the available databases, miRDB, mirTarBase, TarBase, and microT-CDS, predicted and validated targets of the miR-30 miRNAs were listed out and matched with the respective gene array data set to identify overlapping hits. We also used one of our unpublished dataset (high throughput sequencing data on DG samples from defeated mice) to search for targets. Seven upregulated genes in the defeated mouse DG and six downregulated genes in the differentiated cells were selected for further validation (see Table 1).

**Table 1:** List of selected genes as probable targets of miR-30 miRNAs and differentially regulated in gene arrays.

Upregulated genes in DG samples from defeated mice		Downregulated genes in differentiated cells	
*Khnyn*	KH and NYN domain containing protein	*Rcor1*	REST Corepressor 1
*Socs3*	Suppressor of Cytokine Signaling 3	*Omg*	Oligodendrocyte Myelin Glycoprotein
*Ptgfrn*	Prostaglandin F2 Receptor Inhibitor	*Ppp3r1*	Protein Phosphatase 3 Regulatory
			Subunit B, Alpha
*Nrp1*	Neuropilin 1	*Nrp1*	Neuropilin 1
*Osmr*	Oncostatin M Receptor	*Gpr125*	G-Protein Coupled Receptor 125
*Runx1*	Runt-related transcription factor 1, also	*Mll3*	Mixed Lineage Leukemia 3, also known as
	known as *Aml1* (Acute Myeloid Leukemia 1)		*Kmt2c* (Histone Lysine Methyltransferase 2C)
*Ifngr1*	Interferon Gamma Receptor 1

**Figure 5:**
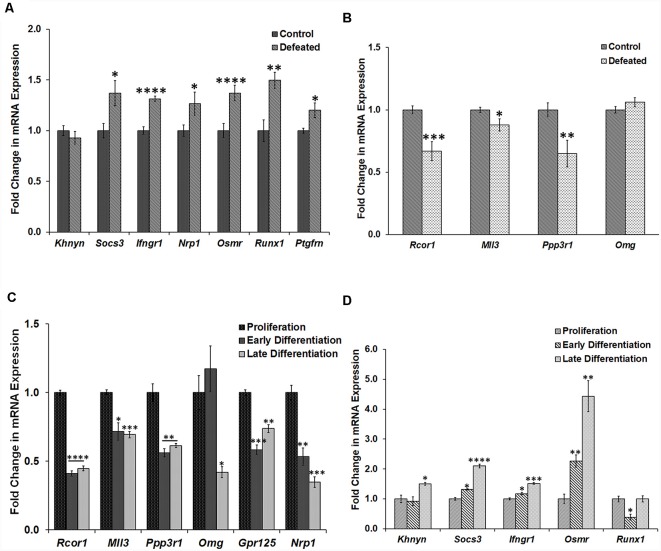
Validation of selected dysregulated genes by RT-qPCR in both the models.** (A)** Fold change in mRNA expression of the selected genes (*Khnyn, Socs3, Ifngr1, Nrp1, Osmr, Runx1*, and *Ptgfrn*) in the DG of defeated mice as compared to the control mice, *n* = 10 mice in each group; GM of Ct values of *Gapdh* and *Tbp* was used for the normalization, two-sample *t*-test, *p* = 0.38, 0.019, <0.0001, 0.0498, <0.0001, 0.0017 and 0.02, respectively, data represented as Mean ± SEM. **(B)** Fold change in mRNA expression of the selected genes (*Rcor1, Mll3, Ppp3r1*, and *Omg*) in DG of the defeated mice as compared to the control mice; GM of Ct values of *Gapdh* and Tbp was used for normalization, *n* = 10 mice in each group, two-sample *t*-test, *p* = 0.0008, 0.033, 0.0089 and 0.1861 respectively, data represented as Mean ± SEM. **(C)** Fold change in mRNA expression of selected genes (*Rcor1, Mll3, Ppp3r1, Omg, Gpr125*, and *Nrp1*) in the cells from early and late stage of differentiation as compared to the proliferating neurospheres; GM of Ct values of *G6pdh* and *Tbp* was used for the normalization, *n* = 3 samples in each group, two-sample *t*-test, p *=* <0.0001, 0.012, 0.0036, 0.449, 0.0005 and 0.0046, respectively for the early differentiation samples and p *=* <0.0001, 0.001, 0.0044, 0.0111, 0.0017 and 0.0006, respectively for the late differentiation samples, data represented as Mean ± SEM. **(D)** Fold change in mRNA expression of selected genes (*Khnyn, Socs3, Ifngr1, Nrp1, Osmr*, and *Runx1*) in the cells from early and late stage of differentiation as compared to the proliferating neurospheres; GM of Ct values of *G6pdh* and *Tbp* was used for normalization, *n* = 3 samples in each group, two-sample *t*-test, *p* = 0.70, 0.011, 0.03, 0.008 and 0.012, respectively for the early differentiation samples and *p* = 0.0184, 0.0001, 0.0003, 0.003 and 1.0, respectively for the late differentiation samples, data represented as Mean ± SEM. *p ≤ 0.05, **p ≤ 0.01, ***p ≤ 0.001, ****p ≤ 0.0001.

The selected potential miR-30 miRNA targets from the array data were validated individually by RT-qPCR in defeated DG samples, where except *Khnyn* all genes (*Socs3, Ifngr1, Nrp1, Osmr, Runx1*, and *Ptgfrn*) showed significant upregulation (Figure 5A). Similarly, array results were replicated for all the six genes (*Rcor1, Mll3, Ppp3r1, Omg, Gpr125*, and* Nrp1*) in samples from the three stages of NSC/NPC culture (Figure 5C).

Additionally, expression of the gene targets validated in defeated DG samples was also assayed in the proliferating and differentiating NSC/NPC culture. Here, only *Runx1* showed downregulation in the early stage of differentiation as compared to the proliferating neurospheres. The other genes (*Socs3, Ifngr1, Osmr, Khnyn*) instead showed an upregulation in the differentiating cells (Figure 5D), which suggested their miR-30 independent regulation. Similarly, genes validated in the culture experiments did not appear to be targeted by miR-30 miRNAs in the defeated DG as they were either downregulated or did not show any change in their expression (Figure 5B).

### Manipulation of miR-30 miRNAs in GL261 Mouse Glioma Cells Alters Most of the Probable miR-30 miRNA Gene Targets

**Figure 6:**
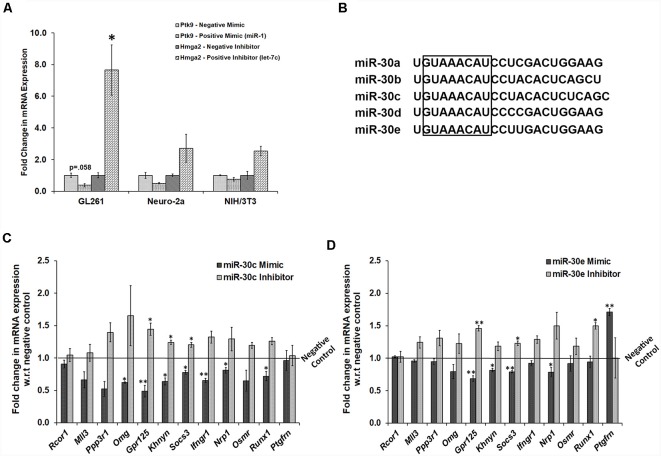
Transfection of mimic and inhibitors of miR-30 family miRNAs in GL261 cells indicates the regulation of several genes by these miRNAs. **(A)** mRNA expression of *Ptk9* with the use of positive (miR-1) mimic as compared to the negative mimic in GL261, Neuro-2a, and NIH/3T3 cells. It also presents mRNA expression of *Hmga2* with the use of positive inhibitor (against let-7c) as compared to the negative inhibitor in all the three cell lines. *Gapdh* was used as a normalization control; *n* = 3 samples in each group, two-sample *t*-test, *p* = 0.058, 0.124 and 0.163, respectively for the positive mimic transfection and *p* = 0.011, 0.194 and 0.059, respectively for positive inhibitor transfection, data is represented as Mean ± SEM. **(B)** Aligned sequences of mature miR-30 miRNAs, where highlighted box shows seed region from position number 2–9 that is fully conserved in all the family members. **(C)** Fold change in mRNA expression of probable gene targets upon manipulation of miR-30c levels in GL261 cells by transfecting with its mimic and inhibitor. The expression data of these genes are analyzed with respect to their expression levels in cells transfected with negative mimic and negative inhibitor, respectively. *Gapdh* was used as a normalization control; *n* = 3 samples in each group; *p* = 0.352, 0.055, 0.054, 0.017, 0.006, 0.027, 0.020, 0.007, 0.014, 0.159, 0.026 and 0.832, respectively in miR-30c mimic transfections and *p* = 0.679, 0.605, 0.157, 0.164, 0.022, 0.030, 0.050, 0.101, 0.218, 0.055, 0.069 and 0.873, respectively in miR-30c inhibitor transfections, data represented as Mean ± SEM. **(D)** Fold change in mRNA expression of probable gene targets upon transfection of mimic and inhibitor of miR-30 miRNAs. The expression data of these genes are analyzed with respect to their expression levels in cells transfected with negative mimic and negative inhibitor, respectively. *Gapdh* was used as a normalization control; *n* = 3 samples in each group, *p* = 0.507, 0.111, 0.538, 0.223, 0.005, 0.017, 0.003, 0.227, 0.042, 0.585, 0.631 and 0.003, respectively in miR-30e mimic transfections and *p* = 0.821, 0.124, 0.18, 0.248, 0.006, 0.197, 0.021, 0.06, 0.091, 0.292, 0.019 and 0.99, respectively in miR-30e inhibitor transfections, data represented as Mean ± SEM. *p ≤ 0.05, **p ≤ 0.01.

To confirm the probable targets, we tried to manipulate miR-30 miRNAs in NSC/NPC culture. However, due to poor transfection efficiency in primary neural culture, mouse cell lines were used, and expression profiling of the target genes was performed by RT-qPCR. We selected three different cell lines, GL261, Neuro-2a, and NIH3T3, and checked for their experimental efficiency, using negative and positive controls (both mimics and inhibitors). We chose GL261 cells for further experiments, as the effect on expression levels of respective genes using control mimics and inhibitors was most significant in these cells as compared to Neuro-2a and NIH3T3 cells (Figure 6A). It should be noted that all five members of the miR-30 family are encoded from three different genomic locations, which form three miRNA clusters that are 100 percent conserved in their seed regions (Figure 6B). Therefore, for manipulation, we selected two members, miR-30c and miR-30e, and transfected GL261 cells with their mimics and inhibitors.

Upon transcriptional analysis, except *Rcor1* and *Ptgfrn*, all other genes were found to be targets of miR-30 miRNAs. The mRNA expression of these genes was attenuated following treatment with mimics of either miR-30c or miR-30e, whereas the use of 30c and 30e inhibitors led to an increase in expression level (Figures 6C,D). Although the difference was not significant in a few cases due to variability, the trend confirmed these targets.

### miR-30 family miRNAs Directly Bind to the 3′UTR of *Socs3, Mll3*, and *Ppp3r1* and Downregulate Their Expression

To assess whether the targets identified or selected after manipulation are directly regulated by miR-30 miRNAs, a luciferase reporter assay was performed for few targets where 3′UTRs of the target genes, cloned into the pmirGLO vector (Promega), were co-transfected with miRNA mimics and inhibitors into HEK-293 cells.

**Figure 7:**
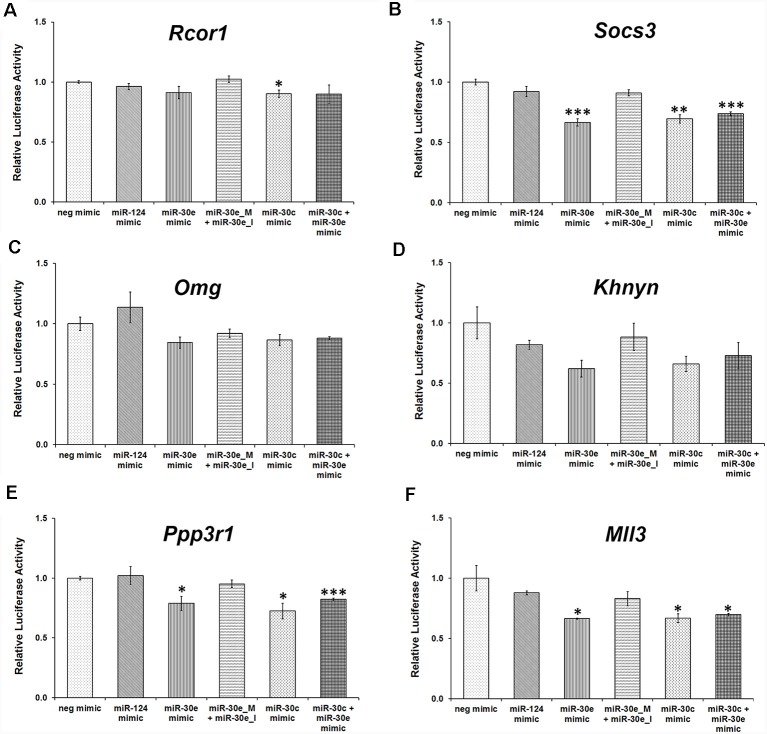
Relative luciferase activity in HEK-293 cells transfected with various 3′UTR clones along with miR-30 mimics and inhibitors. HEK-293 cells were transfected with luciferase reporter constructs having different 3′UTR clones, *Rcor1* **(A)**, *Socs3* **(B)**, *Omg* **(C)**, *Khnyn* **(D)**, *Ppp3r1* **(E)** and *Mll3* **(F)**. Luciferase activity readings of different transfection sets were normalized with their respective negative mimics. Transfection with miR-124 was also used as a negative control. All the experiments were carried out thrice in triplicates; two-sample *t*-test, *p* = 0.366, 0.165, 0.492, 0.042 and 0.25, respectively for *Rcor1* 3′UTR, *p* = 0.278, 0.0009, 0.063, 0.0017 and 0.0008, respectively for *Socs3* 3′UTR, *p* = 0.472, 0.10, 0.301, 0.143 and 0.109, respectively for *Omg* 3′UTR, *p* = 0.305, 0.064, 0.539, 0.081 and 0.191, respectively for *Khnyn* 3′UTR, *p* = 0.823, 0.0265, 0.238, 0.014 and 0.0006, respectively for *Ppp3r1* 3′UTR, *p* = 0.366, 0.033, 0.229, 0.039 and 0.045, respectively for *Mll3* 3′UTR. *p ≤ 0.05, **p ≤ 0.01, ***p ≤ 0.001.

This assay confirmed that *Socs3, Mll3*, and *Ppp3r1* are direct targets of the miR-30 miRNAs (Figures 7B,E,F) as relative luciferase activity was attenuated significantly in the presence of miR-30e mimic, whereas it was analogous to the negative mimic when miR-30e mimic was used with an equimolar ratio of miR-30e inhibitor. Likewise, the use of either miR-30c mimic or a combination of both miR-30c and miR-30e mimics significantly decreased the relative luciferase activity. Use of miR-124 mimic (a negative control) did not show any change in the luciferase activity. However, *Khnyn* also showed a similar trend in the luciferase activity upon transfection with mimics and inhibitors but the difference was not significant (Figure 7D). *Rcor1* and *Omg* do not appear to be direct targets of miR-30 miRNAs (Figures 7A,C).

## Discussion

Depression is a debilitating mental illness, attributed majorly to chronic stress. Molecular investigations using animal models of depression suggest that being subjected to stress for a prolonged period affects gene expression and causes an alteration in neuronal morphology and functional biology, thus resulting in depression, anxiety, and related mood disorders. The mood or affective disorders have been shown to be associated with long-term or persistent changes in hippocampal remodeling and/or neuroplasticity, which are correlated with the attenuation in DG neurogenesis. We have observed a significant decrease in DG neurogenesis 24 h after the last defeat stress using CSDS paradigm (data not shown here). In this window, we have also observed alterations in the expression of a number of genes involved in synaptic plasticity and hippocampal remodeling. Thus, molecular studies on DG samples in this window (24 h after the last defeat stress) appears to be relevant to understand the molecular mechanisms underlying DG and hippocampal neuroplasticity, which sustain the mood disorder. There are recent studies using the CSDS paradigm that showed how 10 days of defeat stress affect cell proliferation and neurogenesis in the hippocampus, specifically in the DG (Yap et al., 2006; Lagace et al., 2010). But unlike our study, the attenuation in neurogenesis was observed in a different time window following the last defeat stress episode. In another parallel study, we have also observed a significant decrease in the number of BrdU positive cells in mouse DG 24 h after the last defeat episode in CSDS paradigm. The analysis of immunostained hippocampal sections for NESTIN (a marker for the proliferating cells) and DCX (a marker for neuronal precursors and immature neurons) showed significant attenuation in the number of cells positive for these markers in DG of defeated mice when compared to non-stressed control animals (unpublished findings from the lab). Moreover, the RT-qPCR data also suggested attenuation in DG neurogenesis, as evident by significant downregulation of Nestin transcript in DG of defeated mice (Figure 3D).

Diverse gene regulatory mechanisms mediated by epigenetic modifiers have been studied extensively in the regulation of these multifactorial psychiatric disorders. microRNAs, one of the major classes of small regulatory non-coding RNAs, have been implicated in various neurological and psychiatric disorders, and targeting them may have a therapeutic relevance (Miller and Wahlestedt, 2010). However, a clear insight into the miRNA-affected gene regulation of the neural and behavioral changes in stress-induced depression models is still to be obtained. Towards this direction, our efforts have headed us to uncover dysregulation of miRNA-mRNA networks in the hippocampal DG of the CSDS-induced depressed mice. Our study started with the finding where we spotted a 40% reduction in the mRNA expression of Drosha, one of the key proteins in miRNA biogenesis, in the DG of the depressed mice. This observation pushed us to search for the downstream effects in terms of expression of the miRNAs using high throughput techniques. The miRNA arrays indicated the dysregulation of hundreds of miRNAs in the hippocampal neurogenic region of DG, which may affect the translation of several genes involved in regulating neuroglial as well as neurogenic responses that either lead to or as well are caused by depression.

Similar to previous findings where miR-9, miR-34c, miR-132, miR-212, and others have been shown to be implicated in various neuropsychiatric and neurodevelopmental disorders (Szulwach et al., 2010; Im et al., 2012; Miller et al., 2012), we detected the dysregulation of these miRNAs in our miRNA array data from the DG of the defeated mice. We also validated the reduced expression of miR-132, by RT-qPCR in these samples (Figure 3B). Since miR-132 has been widely reported to regulate neuroplasticity and neural activity (Luikart et al., 2011; Zheng et al., 2013), compromised hippocampal synaptic plasticity observed in stress and depression (Pittenger and Duman, 2008), and also in our study of socially defeated mice could be attributed to the attenuated expression of miR-132. There is no human study till date that shows our novel findings (dysregulation of Drosha and a number of miRNAs, including mir-30 family members in mouse DG in CSDS model of depression) in post mortem brain samples from depressed individuals.

However, the most interesting finding in our study was the decrease in expression of all the members of the miR-30 microRNA family, in the DG of the CSDS-induced anxious and depressed mice. CSDS paradigm has been reported to have an adverse effect on neurogenesis in the DG, where NSCs/NPCs reside (Lagace et al., 2010; Van Bokhoven et al., 2011; Hollis and Kabbaj, 2014). To understand the role of miRNAs in the regulation of the dynamic process of neurogenesis in the CSDS-affected adult brain, we established neurosphere culture derived from the cells isolated from the DG region of the defeated and control mouse brain. However, to our surprise, the number and size of neurospheres were dramatically low in the cells isolated from the defeated mice, which further reduced with the subsequent passages (data not shown here). Consequently, NSCs/NPCs derived from the DG of control mice were cultured to explore the function of miRNAs in their maintenance, division (proliferation) and differentiation. Analysis of the miRNA array data from the proliferating and differentiating NSCs/NPCs led us to uncover dysregulation of numerous miRNAs, especially the elevated expression of miR-30 miRNAs upon differentiation.

miR-30 family of miRNAs comprises five members: miR-30a, miR-30b, miR-30c, miR-30d and miR-30e (Wang et al., 2013), which have shown to be involved in several cellular and physiological processes. These miRNAs act both as tumor suppressors (Kao et al., 2014; Zhao et al., 2014; Tsukasa et al., 2016) as well as oncogenic miRNAs (Mao et al., 2018). They also have been implicated in myogenic, osteoblast and adipocyte differentiation (Balderman et al., 2012; Guess et al., 2015). However, their role has not yet been explored in the regulation of stem cells of neural origin. Furthermore, their implication in few brain disorders has been reported, as shown by reduced miR-30b/30e levels in the prefrontal cortex of schizophrenic patients (Perkins et al., 2007) as well as a strong association of functional single nucleotide polymorphisms (SNPs) in pre-miR-30e with schizophrenia (Xu et al., 2010a) and MDDs (Xu et al., 2010b).

To the best of our knowledge, this is the first report which describes the association of miR-30 miRNAs with the proliferation and differentiation of NSCs/NPCs as well as their possible involvement in CSDS-induced anxiety and depression-like phenotype in the mouse. Our results suggest that miR-30 miRNAs could be promoting differentiation of NSCs/NPCs by suppressing several key factors required for their proliferation. We also believe that one of the several factors for reduced DG neurogenesis under depressed/defeated conditions could be the compromised NSC/NPC differentiation elicited by the reduced miR-30 miRNA levels.

It should be noted that the miR-30 miRNAs were not manipulated in NPCs/NSCs, because of the difficulty in achieving good transfection efficiency with the mimics and inhibitors. Rather, we have used mouse glioma cell line GL261, which is a limitation of our study. A better molecular insight into our finding was availed using bioinformatics, luciferase reporter assays and from previously published reports, where we identified specific gene targets through which miR-30 miRNAs might be mediating the effects of CSDS on the hippocampal neurogenesis and neuroplasticity. One of these gene targets is *Socs3*, which has been shown to be repressed by miR-30 in glioma stem cells (Che et al., 2015) and is induced by Interleukin-6 (IL6; Shuai and Liu, 2003). IL6 functions both as an anti-inflammatory and a pro-inflammatory cytokine, and it’s level increases in blood upon exposure to the CSDS paradigm (Merlot et al., 2003). We propose that miR-30 miRNAs could be an additional regulatory mechanism to keep *Socs3* silent in the healthy mouse brain. Upon defeat, reduced miR-30 miRNA levels and increased blood IL6 levels together might lead to an increase in *Socs3* expression which is a neuroprotective mechanism (Mingmalairak et al., 2010). Increase in *Socs3* could trigger the genes involved in immune, inflammatory and defense responses which, were found upregulated in our microarray data upon pathway analysis.

*Ppp3r1* is a critical gene in the calcineurin signaling pathway and involved in neural induction during the embryonic development (Cho et al., 2014) has been reported to be targeted by miR-30a (Wu et al., 2015). We also observed the direct targeting of 3′UTR of *Ppp3r1* gene by two of the miR-30 members, and propose its role in the induction of NSC/NPC differentiation; wherein it is required only in the first few hours and is repressed later by high miR-30 levels. Our results demonstrate that *Mll3*, a methyltransferase that methylates lysine 4 of histone H3 and activates transcription, is a novel direct target of miR-30 miRNAs. We hypothesize that *Mll3* is required for the activation of genes involved in the proliferation of NSCs/NPCs, and an increase in miR-30 miRNAs upon differentiation suppresses *Mll3* expression, thereby preventing the expression of the proliferation markers. We also spotted an inverse correlation in the expression of *Gpr125* with that of miR-30 miRNAs, which was further validated by bioinformatics and miRNA manipulation experiments. *Gpr125* has been shown to be expressed more in undifferentiated and progenitor germline cells (Seandel et al., 2007); in conjunction with this, we observed its higher expression in proliferating NSCs/NPCs. However, its reduced levels upon differentiation could possibly be ascribed to the increased expression of miR-30 miRNAs. Nevertheless, regulation of these genes does not appear to be miR-30-mediated in mouse DG upon CSDS, which corroborates with the fact that miRNA action is highly regulated, and a particular gene is targeted and repressed only under specific circumstances.

Interestingly, two of the genes, *Nrp1* and *Runx1*, appeared to be regulated by miR-30 miRNAs in both *in vitro* and *in vivo* systems. Our manipulation experiments, bioinformatics predictions, and previous reports advocate *Nrp1* targeting by miR-30 miRNAs (Ben-Ami et al., 2009; Han et al., 2015), although their direct binding on its 3′UTR has not been shown. *Nrp1* is upregulated in the post-mortem prefrontal cortex of individuals suffering from the MDD and has been proposed to be associated with neuronal morphological alterations in depression and related neuropsychiatric disorders (Goswami et al., 2013). Here, for the first time, we observed *Nrp1* upregulation in the DG of CSDS-induced depressed mouse, which might be due to the attenuated miR-30 levels, and thus can result in the defeat-induced neurobehavioral changes. Additionally, *Nrp1* has been demonstrated as an essential protein for the proliferation of NSCs and neurosphere formation (Shetty et al., 2013). On a similar line, we observed a higher expression of *Nrp1* in the proliferating neurospheres. We propose that an increase in miR-30 expression suppresses proliferation by targeting and repressing *Nrp1*. Recent studies have reported a noteworthy role of *Runx1*, a transcriptional factor as well as a miR-30 target, in the proliferation and differentiation of several neuronal and mesenchymal stem cells (Kim et al., 2014; Logan et al., 2015). Interestingly, its overexpression induces neuronal differentiation, whereas its inhibition reduces the proliferation of neurospheres, suggesting its requirement in both proliferation and differentiation pathways (Logan et al., 2015). Previous studies, as well as our findings of an inverse correlation between Runx1 expression and miR-30 levels in both *in vivo* and *ex vivo* systems, suggest miR-30 miRNAs-mediated *Runx1* regulation in NSCs/NPCs in the primary cell cultures as well as in the DG.

Our study proposes a significant role of miR-30 family miRNAs in chronic stress-induced neural and neurogenic changes underlying depression and related affective disorders, *via* targeting several genes that include few novel ones. However, this could be just one of the several regulatory mechanisms involved in complex brain disorders. Moreover, further downstream studies may be required to substantiate the regulation of these targets through miR-30 miRNAs. Furthermore, it would be interesting to decrypt the mechanism of altered expression of miR-30 miRNAs at different stages, especially either at the transcriptional stage or at the final processing level. Our results also reiterate the fact that the activity of a miRNA depends upon the cell/tissue type, under different conditions. Finally, apart from miR-30 miRNAs and their gene targets, our study uncovers several dysregulated miRNAs and mRNAs in the DG due to chronic stress-induced depression. This would probably be helpful in unfolding the miRNA-mRNA networks involved in the etiopathology of depression and related disorders.

## Data Availability

The data discussed in this publication have been deposited in NCBI’s Gene Expression Omnibus (Edgar et al., 2002) and are accessible through GEO Series accession number GSE132823 (https://www.ncbi.nlm.nih.gov/geo/query/acc.cgi?acc = GSE132823).

## Ethics Statement

This study on mouse was carried out in accordance with the recommendations of the Institutional Animal Ethics Committee (IAEC) of the Centre for Cellular and Molecular Biology (CCMB, Hyderabad, India). The protocol was approved by the IAEC/CCMB/2016–17.

## Author Contributions

NK, SD, and SC performed all the experiments. NK and AK analyzed the data and made the figures. NK, AK, and SC wrote the manuscript.

## Conflict of Interest Statement

The authors declare that the research was conducted in the absence of any commercial or financial relationships that could be construed as a potential conflict of interest.

## Footnotes

^1^https://www.ncbi.nlm.nih.gov/geo/query/acc.cgi?acc=GSE132821

^2^https://www.ncbi.nlm.nih.gov/geo/query/acc.cgi?acc=GSE132822

^3^https://www.ncbi.nlm.nih.gov/geo/query/acc.cgi?acc=GSE132819

^4^https://www.ncbi.nlm.nih.gov/geo/query/acc.cgi?acc=GSE132820

## Supplementary Material

TABLE S1 The list comprises of miRNAs, which demonstrated increased expression (fold change ≥1.2) in the DG of defeated mice when compared with the controls.

TABLE S2 The list comprises of miRNAs, which demonstrated decreased expression (fold change ≥1.2) in the DG of defeated mice when compared with the controls.

TABLE S3 The list comprises of genes, which demonstrated increased mRNA expression (fold change ≥1.2 and *p* ≤ 0.05) in the DG of defeated mice when compared with the controls.

TABLE S4 The list comprises of genes, which demonstrated decreased mRNA expression (fold change ≥1.2 and *p* ≤ 0.05) in the DG of defeated mice when compared with the controls.

TABLE S5 The list comprises of miRNAs, which demonstrated increased expression (fold change ≥1.2 and *p* ≤ 0.05) in the differentiated cells from the late phase (day 7) when compared with the proliferating neurospheres.

TABLE S6 The list comprises of miRNAs, which demonstrated decreased expression (fold change ≥1.2 and *p* ≤ 0.05) in the differentiated cells from the late phase (day 7) when compared with the proliferating neurospheres.

TABLE S7 The list comprises of genes which demonstrated increased expression (fold change ≥1.5 and *p* ≤ 0.05) in the differentiated cells from an early phase (day 2) when compared with the proliferating neurospheres.

TABLE S8 The list comprises of genes which demonstrated decreased expression (fold change ≥1.5 and *p* ≤ 0.05) in the differentiated cells from an early phase (day 2) when compared with the proliferating neurospheres.

TABLE S9 The list comprises of genes which demonstrated increased expression (fold change ≥1.5 and *p* ≤ 0.05) in the differentiated cells from the late phase (day 7) when compared with the proliferating neurospheres.

TABLE S10 The list comprises of genes which demonstrated decreased expression (fold change ≥1.5 and *p* ≤ 0.05) in the differentiated cells from the late phase (day 7) when compared with the proliferating neurospheres.

TABLE S11 The list comprises of genes which demonstrated increased expression (fold change ≥1.5 and *p* ≤ 0.05) in the differentiated cells from the late phase (day 7) when compared with the differentiated cells from an early phase (day 2).

TABLE S12 The list comprises of genes which demonstrated decreased expression (fold change ≥1.5 and *p* ≤ 0.05) in the differentiated cells from the late phase (day 7) when compared with the differentiated cells from an early phase (day 2).
