# Supplementary material for: miR-30 Family miRNAs Mediate the Effect of Chronic Social Defeat Stress on Hippocampal Neurogenesis in Mouse Depression Model
Source: Front Mol Neurosci. 2019 Aug 8;12:188. doi: 10.3389/fnmol.2019.00188 (PMC6694739; doi:10.3389/fnmol.2019.00188)
Supplement: TABLE S1 — The list comprises of miRNAs, which demonstrated increased expression (fold change ≥1.2) in the DG of defeated mice when compared with the controls. [file Table_1.pdf]

Table S1. List of miRNAs, which demonstrated increased expression (fold change ≥ 1.2) in the DG of defeated mice when compared with the controls

Name of the miRNA	Fold Change
mmu-let-7c-1-star	1.25
mmu-let-7i	1.26
mmu-miR-106b	1.46
mmu-miR-106b-star	1.27
mmu-miR-1195	1.66
mmu-miR-1198	1.23
mmu-miR-124-star	1.24
mmu-miR-125a-3p	1.28
mmu-miR-125b-3p	1.20
mmu-miR-125b-star	1.68
mmu-miR-127-star	1.30
mmu-miR-128	1.20
mmu-miR-134	1.31
mmu-miR-139-3p	1.29
mmu-miR-152	1.20
mmu-miR-15a	1.74
mmu-miR-16	1.23
mmu-miR-17	1.34
mmu-miR-17-star	1.79
mmu-miR-181a-1-star	1.26
mmu-miR-182	1.30
mmu-miR-187	1.20
mmu-miR-18a-star	1.24
mmu-miR-191-star	1.26
mmu-miR-193b	1.25
mmu-miR-194	1.32
mmu-miR-195	1.29
mmu-miR-196a-star	1.30
mmu-miR-199a-3p	1.36
mmu-miR-199a-5p	1.34
mmu-miR-200a	1.44
mmu-miR-200b	1.21
mmu-miR-21	1.29
mmu-miR-212	1.52
mmu-miR-214-star	1.21
mmu-miR-21-star	1.47
mmu-miR-24-2-star	1.23
mmu-miR-27b	1.39
mmu-miR-28	1.75
mmu-miR-296-5p	1.35
mmu-miR-297a-star	1.25
mmu-miR-297c-star	1.24
mmu-miR-298	1.32
mmu-miR-299	1.45
mmu-miR-300	1.20
mmu-miR-30b-star	1.26
mmu-miR-30c-2-star	1.50
mmu-miR-320	1.28
mmu-miR-324-3p	1.41
mmu-miR-324-5p	1.23
mmu-miR-326	1.33
mmu-miR-328	1.29
mmu-miR-329	1.46
mmu-miR-330-star	1.22
mmu-miR-337-5p	1.29
mmu-miR-342-5p	1.40
mmu-miR-345-3p	1.49

mmu-miR-345-5p	1.36
mmu-miR-346	1.22
mmu-miR-369-5p	1.35
mmu-miR-376b	1.38
mmu-miR-376c-star	1.22
mmu-miR-381	1.52
mmu-miR-409-3p	1.38
mmu-miR-410	2.35
mmu-miR-423-3p	1.62
mmu-miR-425	1.34
mmu-miR-425-star	1.30
mmu-miR-433	1.27
mmu-miR-466f-5p	1.40
mmu-miR-467d	1.21
mmu-miR-467f	1.42
mmu-miR-468	1.28
mmu-miR-485-star	1.47
mmu-miR-497	1.71
mmu-miR-532-3p	1.35
mmu-miR-540-3p	1.22
mmu-miR-574-3p	1.20
mmu-miR-574-5p	1.32
mmu-miR-592	1.40
mmu-miR-652	1.29
mmu-miR-666-3p	1.64
mmu-miR-666-5p	1.94
mmu-miR-667	1.59
mmu-miR-668	1.38
mmu-miR-669f	1.39
mmu-miR-671-5p	1.42
mmu-miR-672	1.26
mmu-miR-674-star	1.23
mmu-miR-685	1.55
mmu-miR-690	1.23
mmu-miR-698	1.27
mmu-miR-707	1.42
mmu-miR-720	1.64
mmu-miR-743b-3p	1.22
mmu-miR-744	1.20
mmu-miR-760	1.40
mmu-miR-764-5p	1.21
mmu-miR-770-5p	1.20
mmu-miR-7a-star	1.20
mmu-miR-805	1.23
mmu-miR-872-star	1.59
mmu-miR-874	1.31
mmu-miR-875-3p	1.23
mmu-miR-92b	1.28
mmu-miR-93	1.24
