# Supplementary material for: miR-30 Family miRNAs Mediate the Effect of Chronic Social Defeat Stress on Hippocampal Neurogenesis in Mouse Depression Model
Source: Front Mol Neurosci. 2019 Aug 8;12:188. doi: 10.3389/fnmol.2019.00188 (PMC6694739; doi:10.3389/fnmol.2019.00188)
Supplement: TABLE S2 — The list comprises of miRNAs, which demonstrated decreased expression (fold change ≥1.2) in the DG of defeated mice when compared with the controls. [file Table_2.pdf]

Table S2. List of miRNAs, which demonstrated decreased expression (fold change ≥ 1.2) in the DG of defeated mice when compared with the controls

Name of the miRNA	Fold Change
mmu-miR-122	0.811
mmu-miR-125a-5p	0.819
mmu-miR-125b-5p	0.815
mmu-miR-126-3p	0.672
mmu-miR-129-3p	0.424
mmu-miR-129-5p	0.490
mmu-miR-130a	0.473
mmu-miR-132	0.677
mmu-miR-137	0.810
mmu-miR-138-star	0.751
mmu-miR-146b	0.609
mmu-miR-150-star	0.706
mmu-miR-153	0.663
mmu-miR-154	0.700
mmu-miR-181c	0.831
mmu-miR-183-star	0.805
mmu-miR-193-star	0.738
mmu-miR-203-star	0.805
mmu-miR-204	0.652
mmu-miR-206	0.791
mmu-miR-20a	0.468
mmu-miR-20a-star	0.759
mmu-miR-218	0.826
mmu-miR-219	0.796
mmu-miR-25	0.820
mmu-miR-26a	0.351
mmu-miR-27a-star	0.824
mmu-miR-296-3p	0.802
mmu-miR-299-star	0.699
mmu-miR-29b	0.728
mmu-miR-30a	0.228
mmu-miR-30a-star	0.499
mmu-miR-30b	0.253
mmu-miR-30c	0.161
mmu-miR-30d	0.423
mmu-miR-30e	0.255
mmu-miR-30e-star	0.710
mmu-miR-322-star	0.777
mmu-miR-325-star	0.350
mmu-miR-331-5p	0.762
mmu-miR-335-5p	0.594
mmu-miR-339-5p	0.711
mmu-miR-34b-3p	0.596
mmu-miR-34c	0.639
mmu-miR-34c-star	0.397
mmu-miR-351	0.613
mmu-miR-361	0.661
mmu-miR-362-3p	0.825
mmu-miR-362-5p	0.818
mmu-miR-380-3p	0.754
mmu-miR-380-5p	0.818
mmu-miR-411-star	0.690
mmu-miR-421	0.372
mmu-miR-431	0.767
mmu-miR-434-3p	0.795
mmu-miR-448	0.730
mmu-miR-450b-3p	0.826
mmu-miR-466g	0.763

mmu-miR-466i	0.790
mmu-miR-470-star	0.833
mmu-miR-483	0.814
mmu-miR-490	0.772
mmu-miR-493	0.731
mmu-miR-495	0.684
mmu-miR-501-5p	0.806
mmu-miR-539	0.697
mmu-miR-541	0.409
mmu-miR-542-5p	0.626
mmu-miR-551b	0.782
mmu-miR-669c	0.765
mmu-miR-673-3p	0.808
mmu-miR-684	0.702
mmu-miR-691	0.782
mmu-miR-708	0.424
mmu-miR-721	0.764
mmu-miR-743a	0.826
mmu-miR-744-star	0.641
mmu-miR-759	0.799
mmu-miR-764-3p	0.781
mmu-miR-883a-5p	0.768
mmu-miR-9	0.679
mmu-miR-9-star	0.281
