# Supplementary material for: miR-30 Family miRNAs Mediate the Effect of Chronic Social Defeat Stress on Hippocampal Neurogenesis in Mouse Depression Model
Source: Front Mol Neurosci. 2019 Aug 8;12:188. doi: 10.3389/fnmol.2019.00188 (PMC6694739; doi:10.3389/fnmol.2019.00188)
Supplement: TABLE S3 — The list comprises of genes, which demonstrated increased mRNA expression (fold change ≥1.2 and p ≤ 0.05) in the DG of defeated mice when compared with the controls. [file Table_3.pdf]

**Table S3. List of genes, which demonstrated increased expression (fold change ≥ 1.2 and p ≤ 0.05) in the DG of defeated mice when compared with the controls.**

| Gene Symbol | Ensembl Id | Gene Name | Fold Change |
|---|---|---|---|
| *1500001M20Rik* | ENSMUSG00000030316 | RIKEN cDNA 1500001M20 gene | 1.65 |
| *1600014C10Rik* | ENSMUSG00000054676 | RIKEN cDNA 1600014C10 gene | 1.30 |
| *1700029I15Rik* | ENSMUSG00000044916 | RIKEN cDNA 1700029I15 gene | 1.22 |
| *1810062G17Rik* | ENSMUSG00000027713 | RIKEN cDNA 1810062G17 gene | 1.31 |
| *1810063B05Rik* | ENSMUSG00000051671 | RIKEN cDNA 1810063B05 gene | 1.59 |
| *2410017I17Rik* | ENSMUSG00000038311 | RIKEN cDNA 2410017I17 gene | 1.23 |
| *4632419I22Rik* | ENSMUSG00000085208 | RIKEN cDNA 4632419I22 gene | 1.33 |
| *4921517D22Rik* | ENSMUSG00000049902 | RIKEN cDNA 4921517D22 gene | 1.23 |
| *4930402F06Rik* | ENSMUSG00000079421 | RIKEN cDNA 4930402F06 gene | 1.25 |
| *4930430A15Rik* | ENSMUSG00000027157 | RIKEN cDNA 4930430A15 gene | 1.28 |
| *4930432M17Rik* | ENSMUSG00000074248 | RIKEN cDNA 4930432M17 gene | 1.26 |
| *4930433N12Rik* | ENSMUSG00000042360 | RIKEN cDNA 4930433N12 gene | 1.89 |
| *4930526D03Rik* | ENSMUSG00000045794 | RIKEN cDNA 4930526D03 gene | 1.27 |
| *4931428L18Rik* | ENSMUSG00000086727 | RIKEN cDNA 4931428L18 gene | 1.22 |
| *4933404M02Rik* | ENSMUSG00000075302 | RIKEN cDNA 4933404M02 gene | 1.41 |
| *5330438D12Rik* | ENSMUSG00000052291 | RIKEN cDNA 5330438D12 gene | 1.47 |
| *5430419D17Rik* | ENSMUSG00000006204 | RIKEN cDNA 5430419D17 gene | 1.25 |
| *5730577I03Rik* | ENSMUSG00000062470 | RIKEN cDNA 5730577I03 gene | 1.34 |
| *5S_rRNA* | ENSMUSG00000075790 | 5S ribosomal RNA | 1.24 |
| *6030498E09Rik* | ENSMUSG00000051361 | RIKEN cDNA 6030498E09 gene | 1.43 |
| *6330409D20Rik* | ENSMUSG00000009551 | RIKEN cDNA 6330409D20 gene | 1.52 |
| *9030617O03Rik* | ENSMUSG00000021185 | RIKEN cDNA 9030617O03 gene | 1.21 |
| *9230020A06Rik* | ENSMUSG00000072753 | RIKEN cDNA 9230020A06 gene | 1.33 |
| *9330158H04Rik* | ENSMUSG00000073154 | RIKEN cDNA 9330158H04 gene | 1.54 |
| *Aars2* | ENSMUSG00000023938 | alanyl-tRNA synthetase 2, mitochondrial (putative) | 2.42 |
| *Abca14* | ENSMUSG00000062017 | ATP-binding cassette, sub-family A (ABC1), member 14 | 1.52 |
| *Abca9* | ENSMUSG00000041797 | ATP-binding cassette, sub-family A (ABC1), member 9 | 1.28 |
| *Abcb1a* | ENSMUSG00000040584 | ATP-binding cassette, sub-family B (MDR/TAP), member 1A | 1.25 |
| *Abcc12* | ENSMUSG00000036872 | ATP-binding cassette, sub-family C (CFTR/MRP), member 12 | 1.42 |
| *Abhd8* | ENSMUSG00000007950 | abhydrolase domain containing 8 | 1.25 |
| *AC141881.2* | ENSMUSG00000092783 | | 1.24 |
| *Ace2* | ENSMUSG00000015405 | angiotensin I converting enzyme (peptidyl-dipeptidase A) 2 | 1.22 |
| *Ace3* | ENSMUSG00000087418 | angiotensin I converting enzyme (peptidyl-dipeptidase A) 3 | 1.93 |
| *Acer2* | ENSMUSG00000038007 | alkaline ceramidase 2 | 1.90 |
| *Acnat2* | ENSMUSG00000060317 | acyl-coenzyme A amino acid N-acyltransferase 2 | 1.36 |
| *Acvr2a* | ENSMUSG00000052155 | activin receptor IIA | 1.24 |
| *Acvrl1* | ENSMUSG00000000530 | activin A receptor, type II-like 1 | 1.48 |
| *Adam32* | ENSMUSG00000037437 | a disintegrin and metallopeptidase domain 32 | 1.52 |
| *Adam7* | ENSMUSG00000022056 | a disintegrin and metallopeptidase domain 7 | 1.50 |
| *Adamts7* | ENSMUSG00000032363 | a disintegrin-like and metallopeptidase (reprolysin type) with thrombospondin type 1 motif, 7 | 1.59 |
| *Adm* | ENSMUSG00000030790 | adrenomedullin | 1.34 |
| *AF357428* | ENSMUSG00000065749 | snoRNA AF357428 | 1.26 |
| *Agpat2* | ENSMUSG00000026922 | 1-acylglycerol-3-phosphate O-acyltransferase 2 (lysophosphatidic acid acyltransferase, beta) | 1.22 |
| *Agxt* | ENSMUSG00000026272 | alanine-glyoxylate aminotransferase | 1.27 |
| *AI324046* | ENSMUSG00000076615 | expressed sequence AI324046 | 2.02 |
| *AI607873* | ENSMUSG00000073490 | expressed sequence AI607873 | 1.24 |
| *Aif1* | ENSMUSG00000024397 | allograft inflammatory factor 1 | 1.38 |
| *Akr1c6* | ENSMUSG00000021210 | aldo-keto reductase family 1, member C6 | 1.27 |
| *Aldob* | ENSMUSG00000028307 | aldolase B, fructose-bisphosphate | 1.37 |
| *Alox12e* | ENSMUSG00000018907 | arachidonate lipoxygenase, epidermal | 1.46 |
| *Aloxe3* | ENSMUSG00000020892 | arachidonate lipoxygenase 3 | 1.24 |
| *Ambp* | ENSMUSG00000028356 | alpha 1 microglobulin/bikunin | 1.39 |
| *Anxa2* | ENSMUSG00000032231 | annexin A2 | 1.69 |
| *Apoa1* | ENSMUSG00000032083 | apolipoprotein A-I | 1.28 |
| *Apod* | ENSMUSG00000022548 | apolipoprotein D | 1.28 |
| *Apold1* | ENSMUSG00000090698 | apolipoprotein L domain containing 1 | 1.25 |
| *Arc* | ENSMUSG00000022602 | activity regulated cytoskeletal-associated protein | 1.41 |
| *Arhgef19* | ENSMUSG00000028919 | Rho guanine nucleotide exchange factor (GEF) 19 | 1.36 |
| *Arl13b* | ENSMUSG00000022911 | ADP-ribosylation factor-like 13B | 1.33 |

| | | | |
|---|---|---|---|
| *Arpc1b* | ENSMUSG00000029622 | actin related protein 2/3 complex, subunit 1B | 1.26 |
| *Arpc5* | ENSMUSG00000008475 | actin related protein 2/3 complex, subunit 5 | 1.56 |
| *Art3* | ENSMUSG00000034842 | ADP-ribosyltransferase 3 | 1.64 |
| *Atp10d* | ENSMUSG00000046808 | ATPase, class V, type 10D | 1.29 |
| *B2m* | ENSMUSG00000060802 | beta-2 microglobulin | 1.25 |
| *BC051665* | ENSMUSG00000042243 | cDNA sequence BC051665 | 1.29 |
| *Bin2* | ENSMUSG00000075411 | bridging integrator 2 | 1.29 |
| *Bnc2* | ENSMUSG00000028487 | basonuclin 2 | 1.28 |
| *Brix1* | ENSMUSG00000022247 | BRX1, biogenesis of ribosomes, homolog (S. cerevisiae) | 1.26 |
| *Bst1* | ENSMUSG00000029082 | bone marrow stromal cell antigen 1 | 1.41 |
| *C1qa* | ENSMUSG00000036887 | complement component 1, q subcomponent, alpha polypeptide | 1.99 |
| *C1qb* | ENSMUSG00000036905 | complement component 1, q subcomponent, beta polypeptide | 1.50 |
| *C1qc* | ENSMUSG00000036896 | complement component 1, q subcomponent, C chain | 1.34 |
| *C1qtnf6* | ENSMUSG00000022440 | C1q and tumor necrosis factor related protein 6 | 1.51 |
| *C330013J21Rik* | ENSMUSG00000074529 | RIKEN cDNA C330013J21 gene | 1.55 |
| *C3ar1* | ENSMUSG00000040552 | complement component 3a receptor 1 | 1.38 |
| *C4a* | ENSMUSG00000015451 | complement component 4A (Rodgers blood group) | 1.59 |
| *C5ar1* | ENSMUSG00000049130 | complement component 5a receptor 1 | 1.54 |
| *Capn11* | ENSMUSG00000058626 | calpain 11 | 1.42 |
| *Casp4* | ENSMUSG00000033538 | caspase 4, apoptosis-related cysteine peptidase | 1.36 |
| *Cbr2* | ENSMUSG00000025150 | carbonyl reductase 2 | 1.34 |
| *Ccdc120* | ENSMUSG00000031150 | coiled-coil domain containing 120 | 1.28 |
| *Ccdc13* | ENSMUSG00000079235 | coiled-coil domain containing 13 | 1.46 |
| *Ccdc135* | ENSMUSG00000031786 | coiled-coil domain containing 135 | 1.34 |
| *Ccdc136* | ENSMUSG00000029769 | coiled-coil domain containing 136 | 1.32 |
| *Cckar* | ENSMUSG00000029193 | cholecystokinin A receptor | 1.22 |
| *Ccr1* | ENSMUSG00000025804 | chemokine (C-C motif) receptor 1 | 1.24 |
| *Cd300ld* | ENSMUSG00000034641 | CD300 molecule-like family member d | 1.30 |
| *Cd86* | ENSMUSG00000022901 | CD86 antigen | 1.33 |
| *Cd93* | ENSMUSG00000027435 | CD93 antigen | 1.42 |
| *Cdc42bpg* | ENSMUSG00000024769 | CDC42 binding protein kinase gamma (DMPK-like) | 1.64 |
| *Cdkn1a* | ENSMUSG00000023067 | cyclin-dependent kinase inhibitor 1A (P21) | 1.68 |
| *Ceacam2* | ENSMUSG00000054385 | carcinoembryonic antigen-related cell adhesion molecule 2 | 1.42 |
| *Cfh* | ENSMUSG00000026365 | complement component factor h | 1.24 |
| *Cfp* | ENSMUSG00000001128 | complement factor properdin | 1.29 |
| *Ch25h* | ENSMUSG00000050370 | cholesterol 25-hydroxylase | 1.23 |
| *Chst4* | ENSMUSG00000035930 | carbohydrate (chondroitin 6/keratan) sulfotransferase 4 | 1.26 |
| *Cish* | ENSMUSG00000032578 | cytokine inducible SH2-containing protein | 1.22 |
| *Clec2h* | ENSMUSG00000030364 | C-type lectin domain family 2, member h | 1.32 |
| *Clec4a3* | ENSMUSG00000043832 | C-type lectin domain family 4, member a3 | 1.31 |
| *Cnga4* | ENSMUSG00000030897 | cyclic nucleotide gated channel alpha 4 | 1.22 |
| *Cp* | ENSMUSG00000003617 | ceruloplasmin | 1.48 |
| *Crip3* | ENSMUSG00000023968 | cysteine-rich protein 3 | 1.44 |
| *Csf3r* | ENSMUSG00000028859 | colony stimulating factor 3 receptor (granulocyte) | 1.45 |
| *Csk* | ENSMUSG00000032312 | c-src tyrosine kinase | 1.20 |
| *Cst9* | ENSMUSG00000027445 | cystatin 9 | 1.22 |
| *Ctla2a* | ENSMUSG00000044258 | cytotoxic T lymphocyte-associated protein 2 alpha | 1.76 |
| *Ctla2b* | ENSMUSG00000074874 | cytotoxic T lymphocyte-associated protein 2 beta | 1.30 |
| *Ctr9* | ENSMUSG00000005609 | Ctr9, Paf1/RNA polymerase II complex component, homolog (S. cerevisiae) | 1.45 |
| *Ctsc* | ENSMUSG00000030560 | cathepsin C | 1.41 |
| *Ctsh* | ENSMUSG00000032359 | cathepsin H | 1.35 |
| *Ctss* | ENSMUSG00000038642 | cathepsin S | 1.34 |
| *Ctsz* | ENSMUSG00000016256 | cathepsin Z | 1.21 |
| *Cwh43* | ENSMUSG00000029154 | cell wall biogenesis 43 C-terminal homolog (S. cerevisiae) | 1.32 |
| *Cyp2c70* | ENSMUSG00000060613 | cytochrome P450, family 2, subfamily c, polypeptide 70 | 1.42 |
| *Cyyr1* | ENSMUSG00000041134 | cysteine and tyrosine-rich protein 1 | 1.34 |
| *Darc* | ENSMUSG00000037872 | Duffy blood group, chemokine receptor | 1.45 |
| *Dcun1d3* | ENSMUSG00000048787 | DCN1, defective in cullin neddylation 1, domain containing 3 (S. cerevisiae) | 1.34 |
| *Ddit4l* | ENSMUSG00000046818 | DNA-damage-inducible transcript 4-like | 1.61 |
| *Dennd1b* | ENSMUSG00000056268 | DENN/MADD domain containing 1B | 1.27 |
| *Dennd4a* | ENSMUSG00000053641 | DENN/MADD domain containing 4A | 1.21 |
| *Dhx8* | ENSMUSG00000034931 | DEAH (Asp-Glu-Ala-His) box polypeptide 8 | 1.23 |
| *Diap1* | ENSMUSG00000024456 | diaphanous homolog 1 (Drosophila) | 1.20 |

| | | | |
|---|---|---|---|
| *Dlx5* | ENSMUSG00000029755 | distal-less homeobox 5 | 1.26 |
| *Dnase1* | ENSMUSG00000005980 | deoxyribonuclease I | 1.26 |
| *Dnase1l1* | ENSMUSG00000019088 | deoxyribonuclease 1-like 1 | 1.53 |
| *Dtx2* | ENSMUSG00000004947 | deltex 2 homolog (Drosophila) | 1.25 |
| *Duox2* | ENSMUSG00000068452 | dual oxidase 2 | 1.40 |
| *Dusp1* | ENSMUSG00000024190 | dual specificity phosphatase 1 | 1.24 |
| *E030010A14Rik* | ENSMUSG00000048572 | RIKEN cDNA E030010A14 gene | 1.41 |
| *E2f1* | ENSMUSG00000027490 | E2F transcription factor 1 | 1.31 |
| *E430018J23Rik* | ENSMUSG00000078580 | RIKEN cDNA E430018J23 gene | 1.20 |
| *Echs1* | ENSMUSG00000025465 | enoyl Coenzyme A hydratase, short chain, 1, mitochondrial | 1.31 |
| *Eltd1* | ENSMUSG00000039167 | EGF, latrophilin seven transmembrane domain containing 1 | 1.42 |
| *Eng* | ENSMUSG00000026814 | endoglin | 1.26 |
| *Esam* | ENSMUSG00000001946 | endothelial cell-specific adhesion molecule | 1.34 |
| *Esr1* | ENSMUSG00000019768 | estrogen receptor 1 (alpha) | 1.39 |
| *Esyt1* | ENSMUSG00000025366 | extended synaptotagmin-like protein 1 | 1.32 |
| *Fam107a* | ENSMUSG00000021750 | family with sequence similarity 107, member A | 1.22 |
| *Fam114a1* | ENSMUSG00000029185 | family with sequence similarity 114, member A1 | 1.27 |
| *Fam167a* | ENSMUSG00000035095 | family with sequence similarity 167, member A | 1.46 |
| *Fam3a* | ENSMUSG00000031399 | family with sequence similarity 3, member A | 1.21 |
| *Fas* | ENSMUSG00000024778 | Fas (TNF receptor superfamily member 6) | 1.33 |
| *Fasl* | ENSMUSG00000000817 | Fas ligand (TNF superfamily, member 6) | 1.35 |
| *Fbxl20* | ENSMUSG00000020883 | F-box and leucine-rich repeat protein 20 | 1.29 |
| *Fcer1g* | ENSMUSG00000058715 | Fc receptor, IgE, high affinity I, gamma polypeptide | 1.50 |
| *Fcgr2b* | ENSMUSG00000026656 | Fc receptor, IgG, low affinity IIb | 1.51 |
| *Fcgr3* | ENSMUSG00000059498 | Fc receptor, IgG, low affinity III | 1.41 |
| *Fcrls* | ENSMUSG00000015852 | Fc receptor-like S, scavenger receptor | 1.24 |
| *Ffar2* | ENSMUSG00000051314 | free fatty acid receptor 2 | 1.41 |
| *Fgd5* | ENSMUSG00000034037 | FYVE, RhoGEF and PH domain containing 5 | 1.22 |
| *Fgg* | ENSMUSG00000033860 | fibrinogen gamma chain | 1.26 |
| *Flg* | ENSMUSG00000092163 | filaggrin | 1.49 |
| *Fn1* | ENSMUSG00000026193 | fibronectin 1 | 1.40 |
| *Frem1* | ENSMUSG00000059049 | Fras1 related extracellular matrix protein 1 | 1.36 |
| *Fut9* | ENSMUSG00000055373 | fucosyltransferase 9 | 1.20 |
| *Fxyd5* | ENSMUSG00000009687 | FXYD domain-containing ion transport regulator 5 | 1.51 |
| *Galnt2* | ENSMUSG00000089704 | UDP-N-acetyl-alpha-D-galactosamine:polypeptide N-acetylgalactosaminyltransferase 2 | 1.44 |
| *Gbp7* | ENSMUSG00000040253 | guanylate binding protein 7 | 1.23 |
| *Gdpd3* | ENSMUSG00000030703 | glycerophosphodiester phosphodiesterase domain containing 3 | 1.25 |
| *Gga2* | ENSMUSG00000030872 | golgi associated, gamma adaptin ear containing, ARF binding protein 2 | 1.33 |
| *Gjc1* | ENSMUSG00000034520 | gap junction protein, gamma 1 | 1.27 |
| *Gls2* | ENSMUSG00000044005 | glutaminase 2 (liver, mitochondrial) | 1.24 |
| *Gm11428* | ENSMUSG00000069792 | predicted gene 11428 | 2.34 |
| *Gm11517* | ENSMUSG00000081249 | predicted gene 11517 | 1.32 |
| *Gm12830* | ENSMUSG00000055198 | predicted gene 12830 | 1.32 |
| *Gm13103* | ENSMUSG00000029451 | predicted gene 13103 | 1.65 |
| *Gm13364* | ENSMUSG00000085942 | predicted gene 13364 | 1.33 |
| *Gm14115* | ENSMUSG00000081971 | predicted gene 14115 | 1.37 |
| *Gm14781* | ENSMUSG00000064129 | predicted gene 14781 | 1.74 |
| *Gm14926* | ENSMUSG00000081587 | predicted gene 14926 | 1.26 |
| *Gm15368* | ENSMUSG00000081488 | predicted gene 15368 | 1.83 |
| *Gm16378* | ENSMUSG00000074046 | predicted gene 16378 | 1.29 |
| *Gm16519* | ENSMUSG00000066983 | predicted gene, 16519 | 1.20 |
| *Gm20489* | ENSMUSG00000092463 | predicted gene 20489 | 1.65 |
| *Gm20547* | ENSMUSG00000092511 | predicted gene 20547 | 1.36 |
| *Gm41* | ENSMUSG00000080069 | predicted pseudogene 41 | 1.27 |
| *Gm5637* | ENSMUSG00000046993 | predicted pseudogene 5637 | 1.23 |
| *Gm5678* | ENSMUSG00000082815 | predicted gene 5678 | 1.21 |
| *Gm5941* | ENSMUSG00000071726 | predicted gene 5941 | 1.23 |
| *Gm6132* | ENSMUSG00000069200 | predicted pseudogene 6132 | 1.30 |
| *Gm7665* | ENSMUSG00000063628 | predicted pseudogene 7665 | 1.46 |
| *Gm7676* | ENSMUSG00000068631 | predicted gene 7676 | 2.05 |
| *Gm9934* | ENSMUSG00000054061 | predicted gene 9934 | 1.32 |
| *Gpnmb* | ENSMUSG00000029816 | glycoprotein (transmembrane) nmb | 1.30 |
| *Grip2* | ENSMUSG00000030098 | glutamate receptor interacting protein 2 | 1.25 |

| | | | |
|---|---|---|---|
| *Grm2* | ENSMUSG00000023192 | glutamate receptor, metabotropic 2 | 1.24 |
| *Gsdma2* | ENSMUSG00000017211 | gasdermin A2 | 1.97 |
| *Gsn* | ENSMUSG00000026879 | gelsolin | 1.34 |
| *Gyltl1b* | ENSMUSG00000040434 | glycosyltransferase-like 1B | 1.29 |
| *Gypc* | ENSMUSG00000090523 | glycophorin C | 1.40 |
| *Gys2* | ENSMUSG00000030244 | glycogen synthase 2 | 1.40 |
| *Gzmc* | ENSMUSG00000079186 | granzyme C | 1.25 |
| *H2-K1* | ENSMUSG00000061232 | histocompatibility 2, K1, K region | 1.84 |
| *H2-T22* | ENSMUSG00000056116 | histocompatibility 2, T region locus 22 | 1.47 |
| *H47* | ENSMUSG00000075701 | histocompatibility 47 | 1.25 |
| *Hba-a1* | ENSMUSG00000069919 | hemoglobin alpha, adult chain 1 | 1.30 |
| *Hba-a2* | ENSMUSG00000069917 | hemoglobin alpha, adult chain 2 | 1.28 |
| *Hbb-b1* | ENSMUSG00000052305 | hemoglobin, beta adult major chain | 1.63 |
| *Hbb-b2* | ENSMUSG00000073940 | hemoglobin, beta adult minor chain | 1.63 |
| *Hcfc1* | ENSMUSG00000031386 | host cell factor C1 | 1.21 |
| *Hdac11* | ENSMUSG00000034245 | histone deacetylase 11 | 1.39 |
| *Herc6* | ENSMUSG00000029798 | hect domain and RLD 6 | 1.30 |
| *Hist1h2ap* | ENSMUSG00000069304 | histone cluster 1, H2ap | 1.26 |
| *Hist1h3f* | ENSMUSG00000059309 | histone cluster 1, H3f | 1.36 |
| *Hist2h2bb* | ENSMUSG00000050936 | histone cluster 2, H2bb | 1.21 |
| *Hist2h3c2* | ENSMUSG00000081058 | histone cluster 2, H3c2 | 1.37 |
| *Hnrnpf* | ENSMUSG00000042079 | heterogeneous nuclear ribonucleoprotein F | 2.33 |
| *Hoxa7* | ENSMUSG00000038236 | homeobox A7 | 1.52 |
| *Hpd* | ENSMUSG00000029445 | 4-hydroxyphenylpyruvic acid dioxygenase | 1.40 |
| *Hpn* | ENSMUSG00000001249 | hepsin | 1.25 |
| *Icam4* | ENSMUSG00000001014 | intercellular adhesion molecule 4, Landsteiner-Wiener blood group | 1.37 |
| *Ier3* | ENSMUSG00000003541 | immediate early response 3 | 1.38 |
| *Ifi30* | ENSMUSG00000031838 | interferon gamma inducible protein 30 | 1.40 |
| *Ifih1* | ENSMUSG00000026896 | interferon induced with helicase C domain 1 | 1.33 |
| *Ifit1* | ENSMUSG00000034459 | interferon-induced protein with tetratricopeptide repeats 1 | 1.59 |
| *Ifit3* | ENSMUSG00000074896 | interferon-induced protein with tetratricopeptide repeats 3 | 1.40 |
| *Ifitm1* | ENSMUSG00000025491 | interferon induced transmembrane protein 1 | 1.66 |
| *Ifitm2* | ENSMUSG00000060591 | interferon induced transmembrane protein 2 | 1.48 |
| *Ifitm3* | ENSMUSG00000025492 | interferon induced transmembrane protein 3 | 2.09 |
| *Ifitm6* | ENSMUSG00000059108 | interferon induced transmembrane protein 6 | 1.62 |
| *Ifnb1* | ENSMUSG00000048806 | interferon beta 1, fibroblast | 1.23 |
| *Ifne* | ENSMUSG00000045364 | interferon epsilon | 1.44 |
| *Ifngr1* | ENSMUSG00000020009 | interferon gamma receptor 1 | 1.21 |
| *Ift27* | ENSMUSG00000016637 | intraflagellar transport 27 homolog (Chlamydomonas) | 1.32 |
| *Igfbp6* | ENSMUSG00000023046 | insulin-like growth factor binding protein 6 | 1.35 |
| *Igfbp7* | ENSMUSG00000036256 | insulin-like growth factor binding protein 7 | 2.00 |
| *Igj* | ENSMUSG00000067149 | immunoglobulin joining chain | 1.42 |
| *Il15ra* | ENSMUSG00000023206 | interleukin 15 receptor, alpha chain | 1.74 |
| *Il17rc* | ENSMUSG00000030281 | interleukin 17 receptor C | 1.35 |
| *Il18bp* | ENSMUSG00000070427 | interleukin 18 binding protein | 1.28 |
| *Il28b* | ENSMUSG00000060747 | interleukin 28B | 1.39 |
| *Il34* | ENSMUSG00000031750 | interleukin 34 | 1.27 |
| *Ipo11* | ENSMUSG00000078933 | importin 11 | 1.65 |
| *Irak3* | ENSMUSG00000020227 | interleukin-1 receptor-associated kinase 3 | 1.36 |
| *Irf2bp1* | ENSMUSG00000044030 | interferon regulatory factor 2 binding protein 1 | 1.25 |
| *Irf8* | ENSMUSG00000041515 | interferon regulatory factor 8 | 1.43 |
| *Irgm1* | ENSMUSG00000046879 | immunity-related GTPase family M member 1 | 1.50 |
| *Itga10* | ENSMUSG00000090210 | integrin, alpha 10 | 1.64 |
| *Itga6* | ENSMUSG00000027111 | integrin alpha 6 | 1.40 |
| *Itgam* | ENSMUSG00000030786 | integrin alpha M | 1.44 |
| *Itgb5* | ENSMUSG00000022817 | integrin beta 5 | 1.22 |
| *Jakmip1* | ENSMUSG00000063646 | janus kinase and microtubule interacting protein 1 | 1.91 |
| *Kcne4* | ENSMUSG00000047330 | potassium voltage-gated channel, Isk-related subfamily, gene 4 | 1.22 |
| *Kcnj12* | ENSMUSG00000042529 | potassium inwardly-rectifying channel, subfamily J, member 12 | 1.25 |
| *Kcnq1* | ENSMUSG00000009545 | potassium voltage-gated channel, subfamily Q, member 1 | 1.51 |
| *Khnyn* | ENSMUSG00000047153 | KH and NYN domain containing | 1.24 |
| *Kir3dl1* | ENSMUSG00000031424 | killer cell immunoglobulin-like receptor, three domains, long cytoplasmic tail, 1 | 1.51 |
| *Klk1b11* | ENSMUSG00000044485 | kallikrein 1-related peptidase b11 | 1.26 |
| *Klra4* | ENSMUSG00000079852 | killer cell lectin-like receptor, subfamily A, member 4 | 1.61 |

| | | | |
|---|---|---|---|
| *Krt17* | ENSMUSG00000035557 | keratin 17 | 1.24 |
| *Krt7* | ENSMUSG00000023039 | keratin 7 | 1.37 |
| *Krt83* | ENSMUSG00000047641 | keratin 83 | 1.78 |
| *Lag3* | ENSMUSG00000030124 | lymphocyte-activation gene 3 | 1.26 |
| *Lamc2* | ENSMUSG00000026479 | laminin, gamma 2 | 1.47 |
| *Lcn2* | ENSMUSG00000026822 | lipocalin 2 | 17.49 |
| *Lcp1* | ENSMUSG00000021998 | lymphocyte cytosolic protein 1 | 1.65 |
| *Lct* | ENSMUSG00000026354 | lactase | 1.31 |
| *Lenep* | ENSMUSG00000078173 | lens epithelial protein | 1.48 |
| *Lgals3* | ENSMUSG00000050335 | lectin, galactose binding, soluble 3 | 1.39 |
| *Lgals3bp* | ENSMUSG00000033880 | lectin, galactoside-binding, soluble, 3 binding protein | 1.72 |
| *Lgi4* | ENSMUSG00000036560 | leucine-rich repeat LGI family, member 4 | 1.20 |
| *Llgl2* | ENSMUSG00000020782 | lethal giant larvae homolog 2 (Drosophila) | 1.69 |
| *Lrba* | ENSMUSG00000028080 | LPS-responsive beige-like anchor | 1.32 |
| *Lrrc8c* | ENSMUSG00000054720 | leucine rich repeat containing 8 family, member C | 1.32 |
| *Lrriq1* | ENSMUSG00000019892 | leucine-rich repeats and IQ motif containing 1 | 1.25 |
| *Lsr* | ENSMUSG00000001247 | lipolysis stimulated lipoprotein receptor | 1.28 |
| *Ltb* | ENSMUSG00000024399 | lymphotoxin B | 1.55 |
| *Ltf* | ENSMUSG00000032496 | lactotransferrin | 1.36 |
| *Ly6a* | ENSMUSG00000075602 | lymphocyte antigen 6 complex, locus A | 1.74 |
| *Ly6c1* | ENSMUSG00000079018 | lymphocyte antigen 6 complex, locus C1 | 1.64 |
| *Ly6c2* | ENSMUSG00000022584 | lymphocyte antigen 6 complex, locus C2 | 1.68 |
| *Ly86* | ENSMUSG00000021423 | lymphocyte antigen 86 | 1.39 |
| *Lztfl1* | ENSMUSG00000025245 | leucine zipper transcription factor-like 1 | 1.27 |
| *Mafb* | ENSMUSG00000074622 | v-maf musculoaponeurotic fibrosarcoma oncogene family, protein B (avian) | 1.27 |
| *Mageb17-ps* | ENSMUSG00000081960 | melanoma antigen family B, 17, pseudogene | 1.33 |
| *Mamstr* | ENSMUSG00000042918 | MEF2 activating motif and SAP domain containing transcriptional regulator | 1.70 |
| *Man2c1* | ENSMUSG00000032295 | mannosidase, alpha, class 2C, member 1 | 1.41 |
| *Map2k3* | ENSMUSG00000018932 | mitogen-activated protein kinase kinase 3 | 1.44 |
| *Map4k5* | ENSMUSG00000034761 | mitogen-activated protein kinase kinase kinase kinase 5 | 1.25 |
| *March5* | ENSMUSG00000023307 | membrane-associated ring finger (C3HC4) 5 | 1.44 |
| *Matn4* | ENSMUSG00000016995 | matrilin 4 | 1.24 |
| *Mcm9* | ENSMUSG00000058298 | minichromosome maintenance complex component 9 | 1.24 |
| *Men1* | ENSMUSG00000024947 | multiple endocrine neoplasia 1 | 1.48 |
| *Mettl13* | ENSMUSG00000026694 | methyltransferase like 13 | 1.42 |
| *Mettl16* | ENSMUSG00000010554 | methyltransferase like 16 | 1.27 |
| *Mfsd2a* | ENSMUSG00000028655 | major facilitator superfamily domain containing 2A | 1.29 |
| *Mgat1* | ENSMUSG00000020346 | mannoside acetylglucosaminyltransferase 1 | 1.22 |
| *Mgp* | ENSMUSG00000030218 | matrix Gla protein | 1.45 |
| *Micall2* | ENSMUSG00000036718 | MICAL-like 2 | 1.22 |
| *Mir27b* | ENSMUSG00000065475 | microRNA 27b | 1.22 |
| *Mir718* | ENSMUSG00000076127 | microRNA 718 | 1.78 |
| *Mmp8* | ENSMUSG00000005800 | matrix metallopeptidase 8 | 1.73 |
| *Mpeg1* | ENSMUSG00000046805 | macrophage expressed gene 1 | 1.20 |
| *Mpp7* | ENSMUSG00000057440 | membrane protein, palmitoylated 7 (MAGUK p55 subfamily member 7) | 1.48 |
| *Mrpl36* | ENSMUSG00000021607 | mitochondrial ribosomal protein L36 | 1.73 |
| *Ms4a6c* | ENSMUSG00000079419 | membrane-spanning 4-domains, subfamily A, member 6C | 1.73 |
| *Msh6* | ENSMUSG00000005370 | mutS homolog 6 (E. coli) | 1.25 |
| *Msln* | ENSMUSG00000063011 | mesothelin | 1.21 |
| *Msn* | ENSMUSG00000031207 | moesin | 1.34 |
| *Mtor* | ENSMUSG00000028991 | mechanistic target of rapamycin (serine/threonine kinase) | 1.38 |
| *Mustn1* | ENSMUSG00000042485 | musculoskeletal, embryonic nuclear protein 1 | 1.59 |
| *Myadml2* | ENSMUSG00000025141 | myeloid-associated differentiation marker-like 2 | 1.26 |
| *Myct1* | ENSMUSG00000046916 | myc target 1 | 1.24 |
| *Myd88* | ENSMUSG00000032508 | myeloid differentiation primary response gene 88 | 1.42 |
| *Mylk3* | ENSMUSG00000031698 | myosin light chain kinase 3 | 1.63 |
| *Myo1f* | ENSMUSG00000024300 | myosin IF | 1.28 |
| *Myo3a* | ENSMUSG00000025716 | myosin IIIA | 3.02 |
| *Nbr1* | ENSMUSG00000017119 | neighbor of Brca1 gene 1 | 1.28 |
| *Ncf1* | ENSMUSG00000015950 | neutrophil cytosolic factor 1 | 1.26 |
| *Ndc80* | ENSMUSG00000024056 | NDC80 homolog, kinetochore complex component (S. cerevisiae) | 1.51 |
| *Necap1* | ENSMUSG00000030327 | NECAP endocytosis associated 1 | 1.66 |

| | | | |
|---|---|---|---|
| *Nfkb2* | ENSMUSG00000025225 | nuclear factor of kappa light polypeptide gene enhancer in B-cells 2, p49/p100 | 1.21 |
| *Ngp* | ENSMUSG00000032484 | neutrophilic granule protein | 3.18 |
| *Nkapl* | ENSMUSG00000059395 | NFKB activating protein-like | 1.24 |
| *Nkg7* | ENSMUSG00000004612 | natural killer cell group 7 sequence | 1.24 |
| *Nmb* | ENSMUSG00000025723 | neuromedin B | 1.47 |
| *Npas4* | ENSMUSG00000045903 | neuronal PAS domain protein 4 | 1.52 |
| *Npffr2* | ENSMUSG00000035528 | neuropeptide FF receptor 2 | 1.25 |
| *Npy1r* | ENSMUSG00000036437 | neuropeptide Y receptor Y1 | 1.33 |
| *Nr4a1* | ENSMUSG00000023034 | nuclear receptor subfamily 4, group A, member 1 | 1.28 |
| *n-R5s204* | ENSMUSG00000064906 | nuclear encoded rRNA 5S 204 | 1.26 |
| *Ntsr1* | ENSMUSG00000027568 | neurotensin receptor 1 | 1.29 |
| *Nudt5* | ENSMUSG00000025817 | nudix (nucleoside diphosphate linked moiety X)-type motif 5 | 1.25 |
| *Oas1f* | ENSMUSG00000053765 | 2'-5' oligoadenylate synthetase 1F | 1.41 |
| *Oas2* | ENSMUSG00000032690 | 2'-5' oligoadenylate synthetase 2 | 1.61 |
| *Oasl1* | ENSMUSG00000041827 | 2'-5' oligoadenylate synthetase-like 1 | 1.39 |
| *Oasl2* | ENSMUSG00000029561 | 2'-5' oligoadenylate synthetase-like 2 | 1.82 |
| *Olfml3* | ENSMUSG00000027848 | olfactomedin-like 3 | 1.23 |
| *Olfr1193* | ENSMUSG00000060827 | olfactory receptor 1193 | 1.30 |
| *Olfr1463* | ENSMUSG00000052277 | olfactory receptor 1463 | 1.21 |
| *Olfr354* | ENSMUSG00000055088 | olfactory receptor 354 | 1.36 |
| *Olfr767* | ENSMUSG00000059762 | olfactory receptor 767 | 1.65 |
| *Olfr963* | ENSMUSG00000064110 | olfactory receptor 963 | 1.31 |
| *Oma1* | ENSMUSG00000035069 | OMA1 homolog, zinc metallopeptidase (S. cerevisiae) | 1.54 |
| *Oprd1* | ENSMUSG00000050511 | opioid receptor, delta 1 | 1.20 |
| *Osgepl1* | ENSMUSG00000026096 | O-sialoglycoprotein endopeptidase-like 1 | 1.23 |
| *Osmr* | ENSMUSG00000022146 | oncostatin M receptor | 1.51 |
| *Osta* | ENSMUSG00000035699 | organic solute transporter alpha | 1.26 |
| *P2ry2* | ENSMUSG00000032860 | purinergic receptor P2Y, G-protein coupled 2 | 1.60 |
| *Pabpc1l* | ENSMUSG00000054582 | poly(A) binding protein, cytoplasmic 1-like | 1.38 |
| *Pard3b* | ENSMUSG00000052062 | par-3 partitioning defective 3 homolog B (C. elegans) | 2.49 |
| *Parp14* | ENSMUSG00000034422 | poly (ADP-ribose) polymerase family, member 14 | 1.35 |
| *Pax5* | ENSMUSG00000014030 | paired box gene 5 | 1.50 |
| *Pcdhb12* | ENSMUSG00000043458 | protocadherin beta 12 | 1.27 |
| *Pcgf6* | ENSMUSG00000025050 | polycomb group ring finger 6 | 1.20 |
| *Pde6a* | ENSMUSG00000024575 | phosphodiesterase 6A, cGMP-specific, rod, alpha | 1.21 |
| *Pdzd2* | ENSMUSG00000022197 | PDZ domain containing 2 | 3.42 |
| *Peg10* | ENSMUSG00000092035 | paternally expressed 10 | 1.43 |
| *Pex14* | ENSMUSG00000028975 | peroxisomal biogenesis factor 14 | 1.20 |
| *Pglyrp1* | ENSMUSG00000030413 | peptidoglycan recognition protein 1 | 1.41 |
| *Phgdh* | ENSMUSG00000053398 | 3-phosphoglycerate dehydrogenase | 1.42 |
| *Pik3ap1* | ENSMUSG00000025017 | phosphoinositide-3-kinase adaptor protein 1 | 1.27 |
| *Pilrb2* | ENSMUSG00000066682 | paired immunoglobin-like type 2 receptor beta 2 | 1.29 |
| *Pkd1l2* | ENSMUSG00000034416 | polycystic kidney disease 1 like 2 | 1.42 |
| *Pla2g2f* | ENSMUSG00000028749 | phospholipase A2, group IIF | 1.22 |
| *Plcxd2* | ENSMUSG00000087141 | phosphatidylinositol-specific phospholipase C, X domain containing 2 | 1.23 |
| *Plekha4* | ENSMUSG00000040428 | pleckstrin homology domain containing, family A (phosphoinositide binding specific) member 4 | 1.30 |
| *Plin1* | ENSMUSG00000030546 | perilipin 1 | 1.34 |
| *Plin4* | ENSMUSG00000002831 | perilipin 4 | 1.23 |
| *Pml* | ENSMUSG00000036986 | promyelocytic leukemia | 1.20 |
| *Podxl* | ENSMUSG00000025608 | podocalyxin-like | 1.52 |
| *Polr2a* | ENSMUSG00000005198 | polymerase (RNA) II (DNA directed) polypeptide A | 1.20 |
| *Ppbp* | ENSMUSG00000029372 | pro-platelet basic protein | 1.52 |
| *Pqlc2* | ENSMUSG00000028744 | PQ loop repeat containing 2 | 1.20 |
| *Pqlc3* | ENSMUSG00000045679 | PQ loop repeat containing | 1.35 |
| *Prokr2* | ENSMUSG00000050558 | prokineticin receptor 2 | 1.80 |
| *Prol1* | ENSMUSG00000064156 | proline rich, lacrimal 1 | 1.45 |
| *Prr13* | ENSMUSG00000023048 | proline rich 13 | 1.23 |
| *Prrc2b* | ENSMUSG00000039262 | proline-rich coiled-coil 2B | 1.24 |
| *Psg18* | ENSMUSG00000003505 | pregnancy specific glycoprotein 18 | 1.57 |
| *Ptpn6* | ENSMUSG00000004266 | protein tyrosine phosphatase, non-receptor type 6 | 1.25 |
| *Rac2* | ENSMUSG00000033220 | RAS-related C3 botulinum substrate 2 | 1.34 |
| *Racgap1* | ENSMUSG00000023015 | Rac GTPase-activating protein 1 | 1.40 |

| | | | |
|---|---|---|---|
| *Rad54b* | ENSMUSG00000066307 | RAD54 homolog B (S. cerevisiae) | 1.72 |
| *Rad54b* | ENSMUSG00000078773 | RAD54 homolog B (S. cerevisiae) | 1.26 |
| *Rbm28* | ENSMUSG00000029701 | RNA binding motif protein 28 | 1.24 |
| *Rcn3* | ENSMUSG00000019539 | reticulocalbin 3, EF-hand calcium binding domain | 1.41 |
| *Relt* | ENSMUSG00000008318 | RELT tumor necrosis factor receptor | 1.34 |
| *Retnlg* | ENSMUSG00000022651 | resistin like gamma | 1.59 |
| *Rfc3* | ENSMUSG00000033970 | replication factor C (activator 1) 3 | 1.33 |
| *Rgl2* | ENSMUSG00000041354 | ral guanine nucleotide dissociation stimulator-like 2 | 1.20 |
| *Rhbdl3* | ENSMUSG00000017692 | rhomboid, veinlet-like 3 (Drosophila) | 1.51 |
| *Rhbg* | ENSMUSG00000001417 | Rhesus blood group-associated B glycoprotein | 1.29 |
| *Rhpn2* | ENSMUSG00000030494 | rhophilin, Rho GTPase binding protein 2 | 1.36 |
| *Riok1* | ENSMUSG00000021428 | RIO kinase 1 (yeast) | 1.46 |
| *Rnf213* | ENSMUSG00000070327 | ring finger protein 213 | 1.46 |
| *Rnf215* | ENSMUSG00000003581 | ring finger protein 215 | 1.26 |
| *Rpap1* | ENSMUSG00000034032 | RNA polymerase II associated protein 1 | 1.66 |
| *Rtp1* | ENSMUSG00000033383 | receptor transporter protein 1 | 1.21 |
| *Rtp4* | ENSMUSG00000033355 | receptor transporter protein 4 | 1.31 |
| *S100a11* | ENSMUSG00000027907 | S100 calcium binding protein A11 (calgizzarin) | 1.31 |
| *S100a8* | ENSMUSG00000056054 | S100 calcium binding protein A8 (calgranulin A) | 1.56 |
| *S100a9* | ENSMUSG00000056071 | S100 calcium binding protein A9 (calgranulin B) | 1.31 |
| *Sag* | ENSMUSG00000056055 | retinal S-antigen | 1.26 |
| *Scgb3a1* | ENSMUSG00000064057 | secretoglobin, family 3A, member 1 | 4.22 |
| *Scpep1* | ENSMUSG00000000278 | serine carboxypeptidase 1 | 1.22 |
| *Sele* | ENSMUSG00000026582 | selectin, endothelial cell | 1.25 |
| *Selk* | ENSMUSG00000042682 | selenoprotein K | 1.28 |
| *Selp* | ENSMUSG00000026580 | selectin, platelet | 2.22 |
| *Sema4d* | ENSMUSG00000021451 | sema domain, immunoglobulin domain (Ig), transmembrane domain (TM) and short cytoplasmic domain, (semaphorin) 4D | 1.31 |
| *Serpina10* | ENSMUSG00000061947 | serine (or cysteine) peptidase inhibitor, clade A (alpha-1 antiproteinase, antitrypsin), member 10 | 1.24 |
| *Serpina6* | ENSMUSG00000060807 | serine (or cysteine) peptidase inhibitor, clade A, member 6 | 1.32 |
| *Serpinb6b* | ENSMUSG00000042842 | serine (or cysteine) peptidase inhibitor, clade B, member 6b | 1.41 |
| *Serpinh1* | ENSMUSG00000070436 | serine (or cysteine) peptidase inhibitor, clade H, member 1 | 1.33 |
| *Sfmbt1* | ENSMUSG00000006527 | Scm-like with four mbt domains 1 | 1.53 |
| *Sfpi1* | ENSMUSG00000002111 | SFFV proviral integration 1 | 1.28 |
| *Sgsh* | ENSMUSG00000005043 | N-sulfoglucosamine sulfohydrolase (sulfamidase) | 1.29 |
| *Sh3pxd2b* | ENSMUSG00000040711 | SH3 and PX domains 2B | 1.49 |
| *Sh3tc1* | ENSMUSG00000036553 | SH3 domain and tetratricopeptide repeats 1 | 1.30 |
| *Shisa5* | ENSMUSG00000025647 | shisa homolog 5 (Xenopus laevis) | 1.23 |
| *Sidt2* | ENSMUSG00000034908 | SID1 transmembrane family, member 2 | 1.40 |
| *Siglecg* | ENSMUSG00000030468 | sialic acid binding Ig-like lectin G | 1.29 |
| *Six3* | ENSMUSG00000038805 | sine oculis-related homeobox 3 homolog (Drosophila) | 1.21 |
| *Sla* | ENSMUSG00000022372 | src-like adaptor | 1.31 |
| *Slc12a8* | ENSMUSG00000035506 | solute carrier family 12 (potassium/chloride transporters), member 8 | 1.35 |
| *Slc12a9* | ENSMUSG00000037344 | solute carrier family 12 (potassium/chloride transporters), member 9 | 1.58 |
| *Slc16a3* | ENSMUSG00000025161 | solute carrier family 16 (monocarboxylic acid transporters), member 3 | 1.32 |
| *Slc16a4* | ENSMUSG00000027896 | solute carrier family 16 (monocarboxylic acid transporters), member 4 | 1.24 |
| *Slc18a2* | ENSMUSG00000025094 | solute carrier family 18 (vesicular monoamine), member 2 | 1.23 |
| *Slc26a1* | ENSMUSG00000046959 | solute carrier family 26 (sulfate transporter), member 1 | 1.20 |
| *Slc2a1* | ENSMUSG00000028645 | solute carrier family 2 (facilitated glucose transporter), member 1 | 1.31 |
| *Slc2a3* | ENSMUSG00000003153 | solute carrier family 2 (facilitated glucose transporter), member 3 | 1.23 |
| *Slc38a5* | ENSMUSG00000031170 | solute carrier family 38, member 5 | 1.40 |
| *Slc45a1* | ENSMUSG00000039838 | solute carrier family 45, member 1 | 1.27 |
| *Slc7a1* | ENSMUSG00000041313 | solute carrier family 7 (cationic amino acid transporter, y+ system), member 1 | 1.20 |
| *Slco6c1* | ENSMUSG00000026331 | solute carrier organic anion transporter family, member 6c1 | 1.20 |
| *Smox* | ENSMUSG00000027333 | spermine oxidase | 1.24 |
| *SNORD115* | ENSMUSG00000075781 | Small nucleolar RNA SNORD115 | 1.29 |
| *Socs7* | ENSMUSG00000038485 | suppressor of cytokine signaling 7 | 1.28 |
| *Sphk1* | ENSMUSG00000061878 | sphingosine kinase 1 | 1.38 |
| *Spib* | ENSMUSG00000008193 | Spi-B transcription factor (Spi-1/PU.1 related) | 1.24 |

| | | | |
|---|---|---|---|
| *Spsb1* | ENSMUSG00000039911 | splA/ryanodine receptor domain and SOCS box containing 1 | 1.64 |
| *Srcap* | ENSMUSG00000053877 | Snf2-related CREBBP activator protein | 1.34 |
| *Srl* | ENSMUSG00000022519 | sarcalumenin | 1.28 |
| *Srpr* | ENSMUSG00000032042 | signal recognition particle receptor ('docking protein') | 1.47 |
| *Ssh1* | ENSMUSG00000042121 | slingshot homolog 1 (Drosophila) | 1.47 |
| *Stat1* | ENSMUSG00000026104 | signal transducer and activator of transcription 1 | 1.42 |
| *Stk19* | ENSMUSG00000061207 | serine/threonine kinase 19 | 1.45 |
| *Stra6* | ENSMUSG00000032327 | stimulated by retinoic acid gene 6 | 1.21 |
| *Stra8* | ENSMUSG00000029848 | stimulated by retinoic acid gene 8 | 1.21 |
| *Stx19* | ENSMUSG00000047854 | syntaxin 19 | 1.28 |
| *Sult1a1* | ENSMUSG00000030711 | sulfotransferase family 1A, phenol-preferring, member 1 | 1.23 |
| *Susd2* | ENSMUSG00000006342 | sushi domain containing 2 | 1.29 |
| *Suv420h2* | ENSMUSG00000059851 | suppressor of variegation 4-20 homolog 2 (Drosophila) | 1.35 |
| *Suz12* | ENSMUSG00000017548 | suppressor of zeste 12 homolog (Drosophila) | 1.23 |
| *Tbx3* | ENSMUSG00000018604 | T-box 3 | 1.22 |
| *Tcea1* | ENSMUSG00000033813 | transcription elongation factor A (SII) 1 | 1.45 |
| *Tctex1d4* | ENSMUSG00000047671 | Tctex1 domain containing 4 | 1.33 |
| *Tet2* | ENSMUSG00000040943 | tet oncogene family member 2 | 1.21 |
| *Tgfbi* | ENSMUSG00000035493 | transforming growth factor, beta induced | 1.48 |
| *Tgfbr2* | ENSMUSG00000032440 | transforming growth factor, beta receptor II | 1.31 |
| *Thbd* | ENSMUSG00000074743 | thrombomodulin | 1.31 |
| *Tiam1* | ENSMUSG00000002489 | T-cell lymphoma invasion and metastasis 1 | 1.43 |
| *Tle2* | ENSMUSG00000034771 | transducin-like enhancer of split 2, homolog of Drosophila E(spl) | 1.46 |
| *Tmbim1* | ENSMUSG00000006301 | transmembrane BAX inhibitor motif containing 1 | 1.79 |
| *Tmed1* | ENSMUSG00000032180 | transmembrane emp24 domain containing 1 | 1.23 |
| *Tmem139* | ENSMUSG00000071506 | transmembrane protein 139 | 1.26 |
| *Tmem14c* | ENSMUSG00000021361 | transmembrane protein 14C | 1.40 |
| *Tmem164* | ENSMUSG00000047045 | transmembrane protein 164 | 1.26 |
| *Tmem168* | ENSMUSG00000029569 | transmembrane protein 168 | 1.40 |
| *Tmem173* | ENSMUSG00000024349 | transmembrane protein 173 | 1.27 |
| *Tmem176a* | ENSMUSG00000023367 | transmembrane protein 176A | 1.39 |
| *Tmem176b* | ENSMUSG00000029810 | transmembrane protein 176B | 1.30 |
| *Tmem210* | ENSMUSG00000026963 | transmembrane protein 210 | 1.24 |
| *Tmem222* | ENSMUSG00000028857 | transmembrane protein 222 | 1.29 |
| *Tmem52* | ENSMUSG00000023153 | transmembrane protein 52 | 1.28 |
| *Tmem71* | ENSMUSG00000036944 | transmembrane protein 71 | 1.34 |
| *Tnfaip1* | ENSMUSG00000017615 | tumor necrosis factor, alpha-induced protein 1 (endothelial) | 1.27 |
| *Tnn* | ENSMUSG00000026725 | tenascin N | 1.42 |
| *Tor3a* | ENSMUSG00000060519 | torsin family 3, member A | 1.26 |
| *Trabd* | ENSMUSG00000015363 | TraB domain containing | 1.28 |
| *Trim27* | ENSMUSG00000021326 | tripartite motif-containing 27 | 1.30 |
| *Trim40* | ENSMUSG00000073399 | tripartite motif-containing 40 | 1.86 |
| *Tsga10ip* | ENSMUSG00000039330 | testis specific 10 interacting protein | 1.31 |
| *Tshz2* | ENSMUSG00000047907 | teashirt zinc finger family member 2 | 1.43 |
| *Tspo* | ENSMUSG00000041736 | translocator protein | 1.20 |
| *Tubb1* | ENSMUSG00000016255 | tubulin, beta 1 | 1.23 |
| *U1* | ENSMUSG00000089390 | U1 spliceosomal RNA | 1.34 |
| *U6* | ENSMUSG00000065859 | U6 spliceosomal RNA | 1.30 |
| *U6* | ENSMUSG00000064386 | U6 spliceosomal RNA | 1.28 |
| *U6* | ENSMUSG00000075966 | U6 spliceosomal RNA | 1.27 |
| *U6* | ENSMUSG00000064920 | U6 spliceosomal RNA | 1.26 |
| *U6* | ENSMUSG00000065802 | U6 spliceosomal RNA | 1.23 |
| *Ubash3a* | ENSMUSG00000042345 | ubiquitin associated and SH3 domain containing, A | 1.78 |
| *Ucp2* | ENSMUSG00000033685 | uncoupling protein 2 (mitochondrial, proton carrier) | 1.38 |
| *Ugt1a9* | ENSMUSG00000090175 | UDP glucuronosyltransferase 1 family, polypeptide A9 | 1.46 |
| *Umodl1* | ENSMUSG00000054134 | uromodulin-like 1 | 1.24 |
| *Usp18* | ENSMUSG00000030107 | ubiquitin specific peptidase 18 | 1.38 |
| *Usp50* | ENSMUSG00000027364 | ubiquitin specific peptidase 50 | 1.31 |
| *Uty* | ENSMUSG00000068457 | ubiquitously transcribed tetratricopeptide repeat gene, Y chromosome | 1.37 |
| *Vcam1* | ENSMUSG00000027962 | vascular cell adhesion molecule 1 | 1.23 |
| *Vmn1r239-ps* | ENSMUSG00000067262 | vomeronasal 1 receptor 239, pseudogene | 1.27 |
| *Vmn1r65* | ENSMUSG00000066850 | vomeronasal 1 receptor 65 | 1.25 |
| *Vmn2r109* | ENSMUSG00000090572 | vomeronasal 2, receptor 109 | 1.22 |
| *Vmn2r15* | ENSMUSG00000091375 | vomeronasal 2, receptor 15 | 1.23 |

| | | | |
|---|---|---|---|
| *Vmn2r66* | ENSMUSG00000072241 | vomeronasal 2, receptor 66 | 1.27 |
| *Vwa5b2* | ENSMUSG00000046613 | von Willebrand factor A domain containing 5B2 | 1.45 |
| *Vwc2l* | ENSMUSG00000045648 | von Willebrand factor C domain-containing protein 2-like | 1.65 |
| *Vwf* | ENSMUSG00000001930 | Von Willebrand factor homolog | 2.05 |
| *Wdr64* | ENSMUSG00000026523 | WD repeat domain 64 | 1.25 |
| *Wdr78* | ENSMUSG00000035126 | WD repeat domain 78 | 1.21 |
| *Wnt6* | ENSMUSG00000033227 | wingless-related MMTV integration site 6 | 1.20 |
| *Wtip* | ENSMUSG00000036459 | WT1-interacting protein | 1.21 |
| *Xbp1* | ENSMUSG00000020484 | X-box binding protein 1 | 1.21 |
| *Xpo5* | ENSMUSG00000067150 | exportin 5 | 1.24 |
| *Zbtb7a* | ENSMUSG00000035011 | zinc finger and BTB domain containing 7a | 1.37 |
| *Zcchc9* | ENSMUSG00000021621 | zinc finger, CCHC domain containing 9 | 1.39 |
| *Zdhhc19* | ENSMUSG00000052363 | zinc finger, DHHC domain containing 19 | 1.25 |
| *Zfp345* | ENSMUSG00000074731 | zinc finger protein 345 | 1.21 |
| *Zfp408* | ENSMUSG00000075040 | zinc finger protein 408 | 1.25 |
| *Zfp418* | ENSMUSG00000034538 | zinc finger protein 418 | 1.20 |
| *Zfp446* | ENSMUSG00000033961 | zinc finger protein 446 | 1.24 |
| *Zfp456* | ENSMUSG00000078995 | zinc finger protein 456 | 1.37 |
| *Zfp53* | ENSMUSG00000057409 | zinc finger protein 53 | 1.29 |
| *Zfp626* | ENSMUSG00000030604 | zinc finger protein 626 | 1.24 |
| *Zfp677* | ENSMUSG00000062743 | zinc finger protein 677 | 1.25 |
| *Zfp74* | ENSMUSG00000059975 | zinc finger protein 74 | 1.34 |
| *Zfp825* | ENSMUSG00000069208 | zinc finger protein 825 | 1.20 |
| *Zscan30* | ENSMUSG00000024274 | zinc finger and SCAN domain containing 30 | 1.49 |
