# Supplementary material for: miR-30 Family miRNAs Mediate the Effect of Chronic Social Defeat Stress on Hippocampal Neurogenesis in Mouse Depression Model
Source: Front Mol Neurosci. 2019 Aug 8;12:188. doi: 10.3389/fnmol.2019.00188 (PMC6694739; doi:10.3389/fnmol.2019.00188)
Supplement: TABLE S4 — The list comprises of genes, which demonstrated decreased mRNA expression (fold change ≥1.2 and p ≤ 0.05) in the DG of defeated mice when compared with the controls. [file Table_4.pdf]

Table S4. List of genes, which demonstrated decreased expression (fold change ≥ 1.2 and p ≤ 0.05) in the DG of defeated mice when compared with the controls.

Gene Symbol	Ensembl Id	Gene Name	Fold Change
0610007P08Rik	ENSMUSG00000021470	RIKEN cDNA 0610007P08 gene	0.82
0610007P22Rik	ENSMUSG00000015126	RIKEN cDNA 0610007P22 gene	0.80
1110021J02Rik	ENSMUSG00000024018	RIKEN cDNA 1110021J02 gene	0.67
1600029O15Rik	ENSMUSG00000057818	RIKEN cDNA 1600029O15 gene	0.78
1700001F09Rik	ENSMUSG00000072603	RIKEN cDNA 1700001F09 gene	0.83
1700003M02Rik	ENSMUSG00000028294	RIKEN cDNA 1700003M02 gene	0.76
1700022I11Rik	ENSMUSG00000028451	RIKEN cDNA 1700022I11 gene	0.70
1700023E05Rik	ENSMUSG00000029248	RIKEN cDNA 1700023E05 gene	0.72
1700029F09Rik	ENSMUSG00000026049	RIKEN cDNA 1700029F09 gene	0.59
1700034I23Rik	ENSMUSG00000074619	RIKEN cDNA 1700034I23 gene	0.76
2310003L22Rik	ENSMUSG00000027384	RIKEN cDNA 2310003L22 gene	0.69
2310079F23Rik	ENSMUSG00000029097	RIKEN cDNA 2310079F23 gene	0.66
2410015M20Rik	ENSMUSG00000049760	RIKEN cDNA 2410015M20 gene	0.81
2410141K09Rik	ENSMUSG00000074832	RIKEN cDNA 2410141K09 gene	0.82
2810417H13Rik	ENSMUSG00000040204	RIKEN cDNA 2810417H13 gene	0.72
3110003A17Rik	ENSMUSG00000078453	RIKEN cDNA 3110003A17 gene	0.81
3110039M20Rik	ENSMUSG00000089922	RIKEN cDNA 3110039M20 gene	0.54
4930438A08Rik	ENSMUSG00000069873	RIKEN cDNA 4930438A08 gene	0.81
4930447C04Rik	ENSMUSG00000021098	RIKEN cDNA 4930447C04 gene	0.53
4930506M07Rik	ENSMUSG00000041362	RIKEN cDNA 4930506M07 gene	0.80
4930512M02Rik	ENSMUSG00000062511	RIKEN cDNA 4930512M02 gene	0.71
4930547N16Rik	ENSMUSG00000035365	RIKEN cDNA 4930547N16 gene	0.81
4930578C19Rik	ENSMUSG00000037358	RIKEN cDNA 4930578C19 gene	0.83
4932442L08Rik	ENSMUSG00000043549	RIKEN cDNA 4932442L08 gene	0.65
4933427G17Rik	ENSMUSG00000030877	RIKEN cDNA 4933427G17 gene	0.63
5330437I02Rik	ENSMUSG00000046610	RIKEN cDNA 5330437I02 gene	0.81
6820408C15Rik	ENSMUSG00000032680	RIKEN cDNA 6820408C15 gene	0.80
A130023I24Rik	ENSMUSG00000050066	RIKEN cDNA A130023I24 gene	0.82
A1bg	ENSMUSG00000022347	alpha-1-B glycoprotein	0.48
A630010A05Rik	ENSMUSG00000075395	RIKEN cDNA A630010A05 gene	0.83
Abca4	ENSMUSG00000028125	ATP-binding cassette, sub-family A (ABC1), member 4	0.76
Abca8b	ENSMUSG00000020620	ATP-binding cassette, sub-family A (ABC1), member 8b	0.83
Abcb4	ENSMUSG00000042476	ATP-binding cassette, sub-family B (MDR/TAP), member 4	0.69
Abcc2	ENSMUSG00000025194	ATP-binding cassette, sub-family C (CFTR/MRP), member 2	0.73
Abi3bp	ENSMUSG00000035258	ABI gene family, member 3 (NESH) binding protein	0.78
Acaa1b	ENSMUSG00000010651	acetyl-Coenzyme A acyltransferase 1B	0.81
Acbd7	ENSMUSG00000026644	acyl-Coenzyme A binding domain containing 7	0.80
Acox2	ENSMUSG00000021751	acyl-Coenzyme A oxidase 2, branched chain	0.73
Acp1	ENSMUSG00000044573	acid phosphatase 1, soluble	0.65
Acta2	ENSMUSG00000035783	actin, alpha 2, smooth muscle, aorta	0.81
Actr10	ENSMUSG00000021076	ARP10 actin-related protein 10 homolog (S. cerevisiae)	0.78
Actr1a	ENSMUSG00000025228	ARP1 actin-related protein 1 homolog A, centractin alpha (yeast)	0.81
Adam2	ENSMUSG00000022039	a disintegrin and metallopeptidase domain 2	0.83
Adam6b	ENSMUSG00000051804	a disintegrin and metallopeptidase domain 6B	0.72
Adamts20	ENSMUSG00000022449	a disintegrin-like and metallopeptidase (reprolysin type) with thrombospondin type 1 motif, 20	0.65
Adora2b	ENSMUSG00000018500	adenosine A2b receptor	0.80
Adrb2	ENSMUSG00000045730	adrenergic receptor, beta 2	0.83
AI413582	ENSMUSG00000062753	expressed sequence AI413582	0.77
Akap13	ENSMUSG00000066406	A kinase (PRKA) anchor protein 13	0.45
Akr1c13	ENSMUSG00000021213	aldo-keto reductase family 1, member C13	0.65
Akr1c21	ENSMUSG00000021207	aldo-keto reductase family 1, member C21	0.65
Alox15	ENSMUSG00000018924	arachidonate 15-lipoxygenase	0.82
Alpk3	ENSMUSG00000038763	alpha-kinase 3	0.80
Alpl	ENSMUSG00000028766	alkaline phosphatase, liver/bone/kidney	0.75
Alppl2	ENSMUSG00000026246	alkaline phosphatase, placental-like 2	0.75
Amotl1	ENSMUSG00000013076	angiomotin-like 1	0.77
Ampd3	ENSMUSG00000005686	adenosine monophosphate deaminase 3	0.77
Amy2b	ENSMUSG00000083079	amylase 2b	0.73
Aqp4	ENSMUSG00000024411	aquaporin 4	0.75
Arhgap4	ENSMUSG00000031389	Rho GTPase activating protein 4	0.72
Arhgef1	ENSMUSG00000040940	Rho guanine nucleotide exchange factor (GEF) 1	0.79

Arl6ip4	ENSMUSG00000029404	ADP-ribosylation factor-like 6 interacting protein 4	0.83
Arl6ip6	ENSMUSG00000026960	ADP-ribosylation factor-like 6 interacting protein 6	0.78
Asb14	ENSMUSG00000021898	ankyrin repeat and SOCS box-containing 14	0.83
Ascl2	ENSMUSG00000009248	achaete-scute complex homolog 2 (Drosophila)	0.75
Aspdh	ENSMUSG00000038704	aspartate dehydrogenase domain containing	0.77
Avil	ENSMUSG00000025432	advillin	0.60
AW146020	ENSMUSG00000035125	expressed sequence AW146020	0.77
B230216G23Rik	ENSMUSG00000071112	RIKEN cDNA B230216G23 gene	0.80
B230319C09Rik	ENSMUSG00000051844	RIKEN cDNA B230319C09 gene	0.77
Bag2	ENSMUSG00000042215	BCL2-associated athanogene 2	0.70
BC002059	ENSMUSG00000060149	cDNA sequence BC002059	0.71
BC030500	ENSMUSG00000049946	cDNA sequence BC030500	0.75
BC049730	ENSMUSG00000045587	cDNA sequence BC049730	0.78
Bcl2l14	ENSMUSG00000030200	BCL2-like 14 (apoptosis facilitator)	0.72
Bmf	ENSMUSG00000040093	BCL2 modifying factor	0.60
Bmp4	ENSMUSG00000021835	bone morphogenetic protein 4	0.78
Brwd1	ENSMUSG00000022914	bromodomain and WD repeat domain containing 1	0.83
Brwd3	ENSMUSG00000063663	bromodomain and WD repeat domain containing 3	0.70
Btf3l4	ENSMUSG00000028568	basic transcription factor 3-like 4	0.82
Calb2	ENSMUSG00000003657	calbindin 2	0.52
Calcb	ENSMUSG00000030666	calcitonin-related polypeptide, beta	0.79
Car9	ENSMUSG00000028463	carbonic anhydrase 9	0.77
Casp2	ENSMUSG00000029863	caspase 2	0.68
Catsper4	ENSMUSG00000048003	cation channel, sperm associated 4	0.82
Ccdc114	ENSMUSG00000040189	coiled-coil domain containing 114	0.75
Ccdc122	ENSMUSG00000034795	coiled-coil domain containing 122	0.75
Ccdc77	ENSMUSG00000030177	coiled-coil domain containing 77	0.76
Cdc14b	ENSMUSG00000033102	CDC14 cell division cycle 14 homolog B (S. cerevisiae)	0.77
Ces1a	ENSMUSG00000071047	carboxylesterase 1A	0.78
Chat	ENSMUSG00000021919	choline acetyltransferase	0.62
Cks1b	ENSMUSG00000028044	CDC28 protein kinase 1b	0.72
Cldn13	ENSMUSG00000008843	claudin 13	0.82
Clgn	ENSMUSG00000002190	calmegin	0.79
Clvs2	ENSMUSG00000019785	clavesin 2	0.83
Cntd1	ENSMUSG00000078653	cyclin N-terminal domain containing 1	0.78
Col16a1	ENSMUSG00000040690	collagen, type XVI, alpha 1	0.78
Col5a2	ENSMUSG00000026042	collagen, type V, alpha 2	0.64
Col6a4	ENSMUSG00000032572	collagen, type VI, alpha 4	0.69
Coq6	ENSMUSG00000021235	coenzyme Q6 homolog (yeast)	0.76
Cpn1	ENSMUSG00000025196	carboxypeptidase N, polypeptide 1	0.63
Crispld1	ENSMUSG00000025776	cysteine-rich secretory protein LCCL domain containing 1	0.82
Crybb2	ENSMUSG00000042240	crystallin, beta B2	0.73
Cse1l	ENSMUSG00000002718	chromosome segregation 1-like (S. cerevisiae)	0.82
Csn1s1	ENSMUSG00000070702	casein alpha s1	0.78
Csn2	ENSMUSG00000063157	casein beta	0.76
Cyp3a59	ENSMUSG00000061292	cytochrome P450, subfamily 3A, polypeptide 59	0.77
Cyp4a12a	ENSMUSG00000066071	cytochrome P450, family 4, subfamily a, polypeptide 12a	0.74
Cyp4b1	ENSMUSG00000028713	cytochrome P450, family 4, subfamily b, polypeptide 1	0.78
Cyp51	ENSMUSG00000001467	cytochrome P450, family 51	0.78
Dbp	ENSMUSG00000059824	D site albumin promoter binding protein	0.80
Dcdc2a	ENSMUSG00000035910	doublecortin domain containing 2a	0.81
Diap2	ENSMUSG00000034480	diaphanous homolog 2 (Drosophila)	0.49
Dnaic1	ENSMUSG00000061322	dynein, axonemal, intermediate chain 1	0.67
Dnm3os	ENSMUSG00000078190	dynamin 3, opposite strand	0.69
Dok7	ENSMUSG00000044716	docking protein 7	0.80
Dph3	ENSMUSG00000021905	DPH3 homolog (KTI11, S. cerevisiae)	0.58
Drd3	ENSMUSG00000022705	dopamine receptor D3	0.83
Dydc2	ENSMUSG00000021791	DPY30 domain containing 2	0.71
Efhb	ENSMUSG00000023931	EF hand domain family, member B	0.75
Ehmt1	ENSMUSG00000036893	euchromatic histone methyltransferase 1	0.77
Eif4e2	ENSMUSG00000026254	eukaryotic translation initiation factor 4E member 2	0.83
Epyc	ENSMUSG00000019936	epiphycan	0.82
Ercc8	ENSMUSG00000021694	excision repaiross-complementing rodent repair deficiency, complementation group 8	0.60
Esco2	ENSMUSG00000022034	establishment of cohesion 1 homolog 2 (S. cerevisiae)	0.76
F12	ENSMUSG00000021492	coagulation factor XII (Hageman factor)	0.80

F9	ENSMUSG00000031138	coagulation factor IX	0.79
Fabp7	ENSMUSG00000019874	fatty acid binding protein 7, brain	0.69
Fah	ENSMUSG00000030630	fumarylacetoacetate hydrolase	0.77
Fam115e	ENSMUSG00000018656	family with sequence similarity 115, member E	0.76
Fam38a	ENSMUSG00000014444	family with sequence similarity 38, member A	0.57
Fam83f	ENSMUSG00000022408	family with sequence similarity 83, member F	0.65
Fbn1	ENSMUSG00000027204	fibrillin 1	0.76
Fbxo40	ENSMUSG00000047746	F-box protein 40	0.76
Fbxo47	ENSMUSG00000070336	F-box protein 47	0.70
Fbxw9	ENSMUSG00000008167	F-box and WD-40 domain protein 9	0.72
Fcgbp	ENSMUSG00000047730	Fc fragment of IgG binding protein	0.75
Fgfr2	ENSMUSG00000030849	fibroblast growth factor receptor 2	0.80
Foxi1	ENSMUSG00000047861	forkhead box I1	0.80
Foxp2	ENSMUSG00000029563	forkhead box P2	0.69
Frat1	ENSMUSG00000067199	frequently rearranged in advanced T-cell lymphomas	0.80
Frmd7	ENSMUSG00000036131	FERM domain containing 7	0.78
Furin	ENSMUSG00000030530	furin (paired basic amino acid cleaving enzyme)	0.69
Gab3	ENSMUSG00000032750	growth factor receptor bound protein 2-associated protein 3	0.73
Galnt12	ENSMUSG00000039774	UDP-N-acetyl-alpha-D-galactosamine:polypeptide N-acetylgalactosaminyltransferase 12	0.81
Ghr	ENSMUSG00000055737	growth hormone receptor	0.80
Gjc2	ENSMUSG00000043448	gap junction protein, gamma 2	0.82
Glb1l	ENSMUSG00000026200	galactosidase, beta 1-like	0.69
Gli2	ENSMUSG00000048402	GLI-Kruppel family member GLI2	0.75
Glt25d2	ENSMUSG00000032649	glycosyltransferase 25 domain containing 2	0.81
Gm10156	ENSMUSG00000066245	predicted gene 10156	0.69
Gm10410	ENSMUSG00000072741	predicted gene 10410	0.74
Gm10663	ENSMUSG00000074302	predicted gene 10663	0.61
Gm10988	ENSMUSG00000078767	predicted gene 10988	0.46
Gm11349	ENSMUSG00000080840	predicted gene 11349	0.79
Gm12302	ENSMUSG00000070402	predicted gene 12302	0.78
Gm12321	ENSMUSG00000083171	predicted gene 12321	0.77
Gm12365	ENSMUSG00000083851	predicted gene 12365	0.74
Gm12642	ENSMUSG00000082325	predicted gene 12642	0.82
Gm12717	ENSMUSG00000081696	predicted gene 12717	0.79
Gm1332	ENSMUSG00000074628	predicted gene 1332	0.77
Gm13359	ENSMUSG00000082951	predicted gene 13359	0.81
Gm14085	ENSMUSG00000079071	predicted gene 14085	0.63
Gm14105	ENSMUSG00000074763	predicted gene 14105	0.82
Gm15453	ENSMUSG00000081535	predicted gene 15453	0.68
Gm15495	ENSMUSG00000081732	predicted gene 15495	0.80
Gm16432	ENSMUSG00000066667	predicted gene 16432	0.83
Gm16462	ENSMUSG00000071735	predicted gene 16462	0.62
Gm16843	ENSMUSG00000076619	predicted gene, 16843	0.57
Gm17462	ENSMUSG00000091626	predicted gene, 17462	0.60
Gm17672	ENSMUSG00000090847	predicted gene, 17672	0.60
Gm2663	ENSMUSG00000090480	predicted gene 2663	0.83
Gm2783	ENSMUSG00000090316	predicted gene 2783	0.60
Gm3594	ENSMUSG00000090943	predicted gene 3594	0.80
Gm3636	ENSMUSG00000091754	predicted gene 3636	0.69
Gm3662	ENSMUSG00000091663	predicted gene 3662	0.60
Gm3696	ENSMUSG00000092167	predicted gene 3696	0.75
Gm3727	ENSMUSG00000091325	predicted gene 3727	0.65
Gm3739	ENSMUSG00000091472	predicted gene 3739	0.76
Gm3755	ENSMUSG00000091227	predicted gene 3755	0.65
Gm3760	ENSMUSG00000091042	predicted gene 3760	0.65
Gm4027	ENSMUSG00000092019	predicted gene 4027	0.72
Gm4736	ENSMUSG00000079276	predicted gene 4736	0.76
Gm4955	ENSMUSG00000037849	predicted gene 4955	0.59
Gm5277	ENSMUSG00000091361	predicted gene 5277	0.71
Gm5431	ENSMUSG00000058163	predicted gene 5431	0.67
Gm5458	ENSMUSG00000071574	predicted gene 5458	0.81
Gm6356	ENSMUSG00000091400	predicted gene 6356	0.64
Gm6455	ENSMUSG00000070940	predicted gene 6455	0.74
Gm6905	ENSMUSG00000090938	predicted gene 6905	0.75
Gm7651	ENSMUSG00000092227	predicted gene 7651	0.73

Gm8374	ENSMUSG00000091676	predicted gene 8374	0.78
Gm867	ENSMUSG00000050157	predicted gene 867	0.80
Gm9246	ENSMUSG00000082080	predicted gene 9246	0.72
Gm9705	ENSMUSG00000062464	predicted gene 9705	0.77
Gng8	ENSMUSG00000063594	guanine nucleotide binding protein (G protein), gamma 8	0.71
Golga2	ENSMUSG00000002546	golgi autoantigen, golgin subfamily a, 2	0.75
Gzme	ENSMUSG00000022156	granzyme E	0.79
H2-DMb1	ENSMUSG00000079547	histocompatibility 2, class II, locus Mb1	0.68
Hat1	ENSMUSG00000027018	histone aminotransferase 1	0.83
Hdhd3	ENSMUSG00000038422	haloacid dehalogenase-like hydrolase domain containing 3	0.77
Hif1an	ENSMUSG00000036450	hypoxia-inducible factor 1, alpha subunit inhibitor	0.75
Hoxd1	ENSMUSG00000042448	homeobox D1	0.79
Hrc	ENSMUSG00000038239	histidine rich calcium binding protein	0.82
Hsd3b7	ENSMUSG00000042289	hydroxy-delta-5-steroid dehydrogenase, 3 beta- and steroid delta-isomerase 7	0.83
Htatip2	ENSMUSG00000039745	HIV-1 tat interactive protein 2, homolog (human)	0.38
Hus1b	ENSMUSG00000076430	Hus1 homolog b (S. pombe)	0.82
Huwe1	ENSMUSG00000025261	HECT, UBA and WWE domain containing 1	0.72
Hyal1	ENSMUSG00000010051	hyaluronoglucosaminidase 1	0.69
Ica1	ENSMUSG00000062995	islet cell autoantigen 1	0.77
Igf2bp3	ENSMUSG00000029814	insulin-like growth factor 2 mRNA binding protein 3	0.78
Igkv12-41	ENSMUSG00000076568	immunoglobulin kappa chain variable 12-41	0.80
Igkv14-111	ENSMUSG00000076519	immunoglobulin kappa variable 14-111	0.58
Igsf5	ENSMUSG00000000159	immunoglobulin superfamily, member 5	0.79
Ikbkb	ENSMUSG00000031537	inhibitor of kappaB kinase beta	0.74
Impdh1	ENSMUSG00000003500	inosine 5'-phosphate dehydrogenase 1	0.80
Iqcd	ENSMUSG00000029601	IQ motif containing D	0.74
Itga9	ENSMUSG00000039115	integrin alpha 9	0.75
Kctd9	ENSMUSG00000034327	potassium channel tetramerisation domain containing 9	0.82
Kif21b	ENSMUSG00000041642	kinesin family member 21B	0.79
Kif24	ENSMUSG00000028438	kinesin family member 24	0.66
Kif2c	ENSMUSG00000028678	kinesin family member 2C	0.66
Klk1b26	ENSMUSG00000053719	kallikrein 1-related petidase b26	0.80
Klk1b27	ENSMUSG00000063177	kallikrein 1-related peptidase b27	0.77
Kpna7	ENSMUSG00000038770	karyopherin alpha 7 (importin alpha 8)	0.61
Krt73	ENSMUSG00000063661	keratin 73	0.73
Krtap3-3	ENSMUSG00000069722	keratin associated protein 3-3	0.78
Lama5	ENSMUSG00000015647	laminin, alpha 5	0.81
Larp1b	ENSMUSG00000037814	La ribonucleoprotein domain family, member 1B	0.73
Lca5	ENSMUSG00000032258	Leber congenital amaurosis 5 (human)	0.82
Lce1d	ENSMUSG00000078658	late cornified envelope 1D	0.83
Lect1	ENSMUSG00000022025	leukocyte cell derived chemotaxin 1	0.72
Lif	ENSMUSG00000034394	leukemia inhibitory factor	0.83
Lilrb4	ENSMUSG00000062593	leukocyte immunoglobulin-like receptor, subfamily B, member 4	0.80
Lix1	ENSMUSG00000047786	limb expression 1 homolog (chicken)	0.83
Lman1l	ENSMUSG00000056271	lectin, mannose-binding 1 like	0.78
Loxhd1	ENSMUSG00000032818	lipoxygenase homology domains 1	0.82
Lrrc18	ENSMUSG00000041673	leucine rich repeat containing 18	0.76
Ltbp4	ENSMUSG00000040488	latent transforming growth factor beta binding protein 4	0.76
Ly75	ENSMUSG00000026980	lymphocyte antigen 75	0.71
Mad2l1bp	ENSMUSG00000034509	MAD2L1 binding protein	0.74
Mageb2	ENSMUSG00000073069	melanoma antigen, family B, 2	0.62
Man1a2	ENSMUSG00000008763	mannosidase, alpha, class 1A, member 2	0.71
Mapre1	ENSMUSG00000027479	microtubule-associated protein, RP/EB family, member 1	0.76
Mcoln2	ENSMUSG00000011008	mucolipin 2	0.77
Meis1	ENSMUSG00000020160	Meis homeobox 1	0.73
Melk	ENSMUSG00000035683	maternal embryonic leucine zipper kinase	0.78
Mettl11a	ENSMUSG00000026857	methyltransferase like 11A	0.75
Mfrp	ENSMUSG00000034739	membrane-type frizzled-related protein	0.74
Mgl2	ENSMUSG00000040950	macrophage galactose N-acetyl-galactosamine specific lectin 2	0.36
Mib1	ENSMUSG00000024294	mindbomb homolog 1 (Drosophila)	0.80
Mill2	ENSMUSG00000040987	MHC I like leukocyte 2	0.79
Mir692-1	ENSMUSG00000076218	microRNA 692-1	0.79
Mis18bp1	ENSMUSG00000047534	MIS18 binding protein 1	0.82
Mospd1	ENSMUSG00000023074	motile sperm domain containing 1	0.74
Mpo	ENSMUSG00000009350	myeloperoxidase	0.79

Mpzl1	ENSMUSG00000026566	myelin protein zero-like 1	0.79
Mrc1	ENSMUSG00000026712	mannose receptor, C type 1	0.32
Mre11a	ENSMUSG00000031928	meiotic recombination 11 homolog A (S. cerevisiae)	0.71
Mtap7d3	ENSMUSG00000067878	MAP7 domain containing 3	0.73
Mtf1	ENSMUSG00000028890	metal response element binding transcription factor 1	0.65
Mtm1	ENSMUSG00000031337	X-linked myotubular myopathy gene 1	0.72
Mtmr1	ENSMUSG00000015214	myotubularin related protein 1	0.83
Mtmr4	ENSMUSG00000018401	myotubularin related protein 4	0.74
Muc4	ENSMUSG00000079620	mucin 4	0.82
Myo1c	ENSMUSG00000017774	myosin IC	0.72
Myo1g	ENSMUSG00000020437	myosin IG	0.78
Naaladl1	ENSMUSG00000054999	N-acetylated alpha-linked acidic dipeptidase-like 1	0.81
Naip3	ENSMUSG00000082956	NLR family, apoptosis inhibitory protein 3	0.75
Neb	ENSMUSG00000026950	nebulin	0.79
Nes	ENSMUSG00000004891	nestin	0.74
Nexn	ENSMUSG00000039103	nexilin	0.65
Nln	ENSMUSG00000021710	neurolysin (metallopeptidase M3 family)	0.80
Nostrin	ENSMUSG00000034738	nitric oxide synthase trafficker	0.71
Npy	ENSMUSG00000029819	neuropeptide Y	0.81
Npy2r	ENSMUSG00000028004	neuropeptide Y receptor Y2	0.75
Nr3c2	ENSMUSG00000031618	nuclear receptor subfamily 3, group C, member 2	0.79
Ntn5	ENSMUSG00000070564	netrin 5	0.75
Nudt1	ENSMUSG00000036639	nudix (nucleoside diphosphate linked moiety X)-type motif 1	0.77
Nup62cl	ENSMUSG00000072944	nucleoporin 62 C-terminal like	0.67
Nup88	ENSMUSG00000040667	nucleoporin 88	0.77
Nyx	ENSMUSG00000051228	nyctalopin	0.75
Oc90	ENSMUSG00000015001	otoconin 90	0.83
Ofd1	ENSMUSG00000040586	oral-facial-digital syndrome 1 gene homolog (human)	0.80
Olfr1021-ps1	ENSMUSG00000082754	olfactory receptor 1021, pseudogene 1	0.80
Olfr1128	ENSMUSG00000075156	olfactory receptor 1128	0.79
Olfr1129	ENSMUSG00000062272	olfactory receptor 1129	0.79
Olfr1282	ENSMUSG00000074964	olfactory receptor 1282	0.75
Olfr13	ENSMUSG00000043605	olfactory receptor 13	0.77
Olfr1477	ENSMUSG00000071629	olfactory receptor 1477	0.79
Olfr1508	ENSMUSG00000057564	olfactory receptor 1508	0.81
Olfr153	ENSMUSG00000061520	olfactory receptor 153	0.77
Olfr22-ps1	ENSMUSG00000079875	olfactory receptor 22, pseudogene 1	0.80
Olfr43	ENSMUSG00000070377	olfactory receptor 43	0.76
Olfr430	ENSMUSG00000050134	olfactory receptor 430	0.73
Olfr466	ENSMUSG00000049806	olfactory receptor 466	0.78
Olfr47	ENSMUSG00000061210	olfactory receptor 47	0.83
Olfr573-ps1	ENSMUSG00000052785	olfactory receptor 573, pseudogene 1	0.81
Olfr623	ENSMUSG00000055124	olfactory receptor 623	0.65
Olfr659	ENSMUSG00000073922	olfactory receptor 659	0.78
Olfr769	ENSMUSG00000042801	olfactory receptor 769	0.83
Olfr951	ENSMUSG00000060129	olfactory receptor 951	0.81
P2ry12	ENSMUSG00000036353	purinergic receptor P2Y, G-protein coupled 12	0.68
Palld	ENSMUSG00000058056	palladin, cytoskeletal associated protein	0.77
Panx1	ENSMUSG00000031934	pannexin 1	0.82
Papd4	ENSMUSG00000042167	PAP associated domain containing 4	0.80
Parl	ENSMUSG00000033918	presenilin associated, rhomboid-like	0.78
Pclo	ENSMUSG00000061601	piccolo (presynaptic cytomatrix protein)	0.63
Pdp2	ENSMUSG00000048371	pyruvate dehyrogenase phosphatase catalytic subunit 2	0.80
Pfn4	ENSMUSG00000020639	profilin family, member 4	0.68
Pglyrp4	ENSMUSG00000042250	peptidoglycan recognition protein 4	0.75
Phka2	ENSMUSG00000031295	phosphorylase kinase alpha 2	0.80
Phospho2	ENSMUSG00000027088	phosphatase, orphan 2	0.68
Pik3c2b	ENSMUSG00000026447	phosphoinositide-3-kinase, class 2, beta polypeptide	0.69
Pim1	ENSMUSG00000024014	proviral integration site 1	0.64
Pkd1l1	ENSMUSG00000046634	polycystic kidney disease 1 like 1	0.77
Pklr	ENSMUSG00000041237	pyruvate kinase liver and red blood cell	0.75
Pla2g4e	ENSMUSG00000050211	phospholipase A2, group IVE	0.80
Pltp	ENSMUSG00000017754	phospholipid transfer protein	0.68
Plxnb3	ENSMUSG00000031385	plexin B3	0.79
Pole	ENSMUSG00000007080	polymerase (DNA directed), epsilon	0.75
Polr3e	ENSMUSG00000030880	polymerase (RNA) III (DNA directed) polypeptide E	0.77

Polr3g	ENSMUSG00000035834	polymerase (RNA) III (DNA directed) polypeptide G	0.71
Ppef2	ENSMUSG00000029410	protein phosphatase, EF hand calcium-binding domain 2	0.81
Ppp4r1	ENSMUSG00000061950	protein phosphatase 4, regulatory subunit 1	0.80
Pramef12	ENSMUSG00000028591	PRAME family member 12	0.57
Prc1	ENSMUSG00000038943	protein regulator of cytokinesis 1	0.60
Prkg1	ENSMUSG00000052920	protein kinase, cGMP-dependent, type I	0.62
Prl7d1	ENSMUSG00000021348	prolactin family 7, subfamily d, member 1	0.73
Prpf40b	ENSMUSG00000023007	PRP40 pre-mRNA processing factor 40 homolog B (yeast)	0.73
Prss58	ENSMUSG00000051936	protease, serine 58	0.75
Psg23	ENSMUSG00000074359	pregnancy-specific glycoprotein 23	0.78
Psmb5-ps	ENSMUSG00000080992	proteasome (prosome, macropain) subunit, beta type 5, pseudogene	0.72
Ptpn4	ENSMUSG00000026384	protein tyrosine phosphatase, non-receptor type 4	0.73
Pycr1	ENSMUSG00000025140	pyrroline-5-carboxylate reductase 1	0.79
Rab34	ENSMUSG00000002059	RAB34, member of RAS oncogene family	0.78
Rad52	ENSMUSG00000030166	RAD52 homolog (S. cerevisiae)	0.49
Rarb	ENSMUSG00000017491	retinoic acid receptor, beta	0.77
Rbm3	ENSMUSG00000031167	RNA binding motif protein 3	0.65
Rbms3	ENSMUSG00000039607	RNA binding motif, single stranded interacting protein	0.74
Rfc1	ENSMUSG00000029191	replication factor C (activator 1) 1	0.73
Rmi1	ENSMUSG00000035367	RMI1, RecQ mediated genome instability 1, homolog (S. cerevisiae)	0.68
Rnf17	ENSMUSG00000000365	ring finger protein 17	0.68
Rnmt	ENSMUSG00000009535	RNA (guanine-7-) methyltransferase	0.78
Rpa3	ENSMUSG00000012483	replication protein A3	0.80
Rps6ka4	ENSMUSG00000024952	ribosomal protein S6 kinase, polypeptide 4	0.78
Rsph10b2	ENSMUSG00000075569	radial spoke head 10 homolog B (Chlamydomonas)	0.67
Rtdr1	ENSMUSG00000009070	rhabdoid tumor deletion region gene 1	0.65
Rtf1	ENSMUSG00000027304	Rtf1, Paf1/RNA polymerase II complex component, homolog (S. cerevisiae)	0.73
Ryr3	ENSMUSG00000057378	ryanodine receptor 3	0.76
Scel	ENSMUSG00000022123	sciellin	0.72
Scgb3a2	ENSMUSG00000038791	secretoglobin, family 3A, member 2	0.79
Scube1	ENSMUSG00000016763	signal peptide, CUB domain, EGF-like 1	0.69
Sectm1a	ENSMUSG00000025165	secreted and transmembrane 1A	0.70
Sephs1	ENSMUSG00000026662	selenophosphate synthetase 1	0.82
Serpina3m	ENSMUSG00000079012	serine (or cysteine) peptidase inhibitor, clade A, member 3M	0.77
Serpinb1a	ENSMUSG00000044734	serine (or cysteine) peptidase inhibitor, clade B, member 1a	0.77
Serpinb1b	ENSMUSG00000051029	serine (or cysteine) peptidase inhibitor, clade B, member 1b	0.75
Serpinb3a	ENSMUSG00000044594	serine (or cysteine) peptidase inhibitor, clade B (ovalbumin), member 3A	0.75
Serpinf1	ENSMUSG00000000753	serine (or cysteine) peptidase inhibitor, clade F, member 1	0.80
Set	ENSMUSG00000054766	SET nuclear oncogene	0.52
Sfn	ENSMUSG00000047281	stratifin	0.50
Sh3rf1	ENSMUSG00000031642	SH3 domain containing ring finger 1	0.79
Slc20a1	ENSMUSG00000027397	solute carrier family 20, member 1	0.79
Slc36a3	ENSMUSG00000049491	solute carrier family 36 (proton/amino acid symporter), member 3	0.79
Slc4a11	ENSMUSG00000074796	solute carrier family 4, sodium bicarbonate transporter-like, member 11	0.62
Slc5a2	ENSMUSG00000030781	solute carrier family 5 (sodium/glucose cotransporter), member 2	0.66
Slc5a9	ENSMUSG00000028544	solute carrier family 5 (sodium/glucose cotransporter), member 9	0.74
Slc6a15	ENSMUSG00000019894	solute carrier family 6 (neurotransmitter transporter), member 15	0.76
Slc7a11	ENSMUSG00000027737	solute carrier family 7 (cationic amino acid transporter, y+ system), member 11	0.79
Slit2	ENSMUSG00000031558	slit homolog 2 (Drosophila)	0.72
Smarca5	ENSMUSG00000031715	SWI/SNF related, matrix associated, actin dependent regulator of chromatin, subfamily 5	0.50
Sox2	ENSMUSG00000074637	SRY-box containing gene 2	0.74
Spag11a	ENSMUSG00000079842	sperm associated antigen 11A	0.83
Spag16	ENSMUSG00000053153	sperm associated antigen 16	0.73
Spag6	ENSMUSG00000022783	sperm associated antigen 6	0.62
St8sia5	ENSMUSG00000025425	ST8 alpha-N-acetyl-neuraminide alpha-2,8-sialyltransferase 5	0.82
Stac	ENSMUSG00000032502	src homology three (SH3) and cysteine rich domain	0.76
Stil	ENSMUSG00000028718	Scl/Tal1 interrupting locus	0.77
Stoml2	ENSMUSG00000028455	stomatin (Epb7.2)-like 2	0.65
Supv3l1	ENSMUSG00000020079	suppressor of var1, 3-like 1 (S. cerevisiae)	0.81
Susd3	ENSMUSG00000021384	sushi domain containing 3	0.81
Svs5	ENSMUSG00000017004	seminal vesicle secretory protein 5	0.77
Syt15	ENSMUSG00000041479	synaptotagmin XV	0.74

Tac1	ENSMUSG00000061762	tachykinin 1	0.75
Taok3	ENSMUSG00000061288	TAO kinase 3	0.83
Tcf7	ENSMUSG00000000782	transcription factor 7, T-cell specific	0.83
Tcfap2b	ENSMUSG00000025927	transcription factor AP-2 beta	0.71
Tdh	ENSMUSG00000021953	L-threonine dehydrogenase	0.49
Tex9	ENSMUSG00000032219	testis expressed gene 9	0.51
Tg	ENSMUSG00000053469	thyroglobulin	0.77
Tle6	ENSMUSG00000034758	transducin-like enhancer of split 6, homolog of Drosophila E(spl)	0.73
Tlr9	ENSMUSG00000045322	toll-like receptor 9	0.78
Tlx1	ENSMUSG00000025215	T-cell leukemia, homeobox 1	0.80
Tmed5	ENSMUSG00000063406	transmembrane emp24 protein transport domain containing 5	0.81
Tmem144	ENSMUSG00000027956	transmembrane protein 144	0.83
Tmem202	ENSMUSG00000049526	transmembrane protein 202	0.74
Tmem35	ENSMUSG00000033578	transmembrane protein 35	0.74
Tmprss15	ENSMUSG00000022857	transmembrane protease, serine 15	0.71
Tnc	ENSMUSG00000028364	tenascin C	0.79
Tnfrsf10b	ENSMUSG00000022074	tumor necrosis factor receptor superfamily, member 10b	0.79
Treh	ENSMUSG00000032098	trehalase (brush-border membrane glycoprotein)	0.77
Trim59	ENSMUSG00000034317	tripartite motif-containing 59	0.42
Trnau1ap	ENSMUSG00000028898	tRNA selenocysteine 1 associated protein 1	0.82
Troap	ENSMUSG00000032783	trophinin associated protein	0.82
Trpm7	ENSMUSG00000027365	transient receptor potential cation channel, subfamily M, member 7	0.64
Tspan18	ENSMUSG00000023461	tetraspanin 18	0.75
Tspan2	ENSMUSG00000027858	tetraspanin 2	0.79
Ttll6	ENSMUSG00000038756	tubulin tyrosine ligase-like family, member 6	0.82
Tuft1	ENSMUSG00000005968	tuftelin 1	0.58
U6	ENSMUSG00000064615	U6 spliceosomal RNA	0.75
U6	ENSMUSG00000065696	U6 spliceosomal RNA	0.83
Ugt8a	ENSMUSG00000032854	UDP galactosyltransferase 8A	0.76
Unc45a	ENSMUSG00000030533	unc-45 homolog A (C. elegans)	0.75
Usp1	ENSMUSG00000028560	ubiquitin specific peptidase 1	0.62
Vill	ENSMUSG00000038775	villin-like	0.57
Vmn1r32	ENSMUSG00000062905	vomeronasal 1 receptor 32	0.70
Vmn1r56	ENSMUSG00000091874	vomeronasal 1 receptor 56	0.83
Vmn2r110	ENSMUSG00000091259	vomeronasal 2, receptor 110	0.82
Vmn2r111	ENSMUSG00000073445	vomeronasal 2, receptor 111	0.78
Vmn2r12	ENSMUSG00000090688	vomeronasal 2, receptor 12	0.70
Vps54	ENSMUSG00000020128	vacuolar protein sorting 54 (yeast)	0.74
Wdr60	ENSMUSG00000042050	WD repeat domain 60	0.82
Wdyhv1	ENSMUSG00000022359	WDYHV motif containing 1	0.82
Wee2	ENSMUSG00000037159	WEE1 homolog 2 (S. pombe)	0.52
Wls	ENSMUSG00000028173	wntless homolog (Drosophila)	0.76
Wtap	ENSMUSG00000060475	Wilms' tumour 1-associating protein	0.77
Xaf1	ENSMUSG00000040483	XIAP associated factor 1	0.73
Ylpm1	ENSMUSG00000021244	YLP motif containing 1	0.74
Zcchc6	ENSMUSG00000035248	zinc finger, CCHC domain containing 6	0.59
Zfp114	ENSMUSG00000068962	zinc finger protein 114	0.82
Zfp259	ENSMUSG00000032078	zinc finger protein 259	0.79
Zfp46	ENSMUSG00000051351	zinc finger protein 46	0.77
Zfp616	ENSMUSG00000069476	zinc finger protein 616	0.71
Zfp760	ENSMUSG00000067928	zinc finger protein 760	0.68
Zfp82	ENSMUSG00000058447	zinc finger protein 82	0.72
Zfp945	ENSMUSG00000059142	zinc finger protein 945	0.75
Zfyve19	ENSMUSG00000068580	zinc finger, FYVE domain containing 19	0.66
Zpld1	ENSMUSG00000064310	zona pellucida like domain containing 1	0.80
Zwilch	ENSMUSG00000032400	Zwilch, kinetochore associated, homolog (Drosophila)	0.78
