# Supplementary material for: miR-30 Family miRNAs Mediate the Effect of Chronic Social Defeat Stress on Hippocampal Neurogenesis in Mouse Depression Model
Source: Front Mol Neurosci. 2019 Aug 8;12:188. doi: 10.3389/fnmol.2019.00188 (PMC6694739; doi:10.3389/fnmol.2019.00188)
Supplement: TABLE S5 — The list comprises of miRNAs, which demonstrated increased expression (fold change ≥1.2 and p ≤ 0.05) in the differentiated cells from the late phase (day 7) when compared with the proliferating neurospheres. [file Table_5.pdf]

Table S5. List of miRNAs, which demonstrated increased expression (fold change ≥ 1.2 and p ≤ 0.05) in the differentiated cells from the late phase (day 7) when compared with the proliferating neurospheres

Name of the miRNA	Fold Change
mmu-let-7f	2.14
mmu-let-7f-1-star	1.36
mmu-let-7g	1.95
mmu-let-7i	1.47
mmu-miR-1186b	1.26
mmu-miR-1196	1.69
mmu-miR-1224	9.88
mmu-miR-129-5p	2.14
mmu-miR-149-star	5.28
mmu-miR-152	1.93
mmu-miR-155	2.32
mmu-miR-1839-5p	2.05
mmu-miR-185	2.81
mmu-miR-1892	2.70
mmu-miR-1899	1.39
mmu-miR-1906	2.24
mmu-miR-1934-star	18.19
mmu-miR-193b-star	2.89
mmu-miR-194	3.09
mmu-miR-1940	3.44
mmu-miR-1943-star	2.43
mmu-miR-1947-star	1.58
mmu-miR-1971	1.23
mmu-miR-1982-star	2.15
mmu-miR-203	4.27
mmu-miR-211-star	3.36
mmu-miR-2137	2.68
mmu-miR-2182	1.88
mmu-miR-24-2-star	1.80
mmu-miR-2861	3.24
mmu-miR-291b-5p	1.74
mmu-miR-29a	2.55
mmu-miR-29b-1-star	2.30
mmu-miR-3059	1.28
mmu-miR-3072-star	1.65
mmu-miR-3077-star	4.49
mmu-miR-3081-star	1.47
mmu-miR-3082-5p	3.50
mmu-miR-3090-star	3.78
mmu-miR-3096-3p	21.69
mmu-miR-3096-5p	3.25
mmu-miR-3096b-3p	23.33
mmu-miR-3096b-5p	6.02
mmu-miR-3099-star	1.35
mmu-miR-30a	2.95
mmu-miR-30b	1.88
mmu-miR-30e	2.72
mmu-miR-31	2.85
mmu-miR-3102-star	8.82
mmu-miR-3104-5p	9.92
mmu-miR-3107-star	1.67
mmu-miR-326-star	2.62
mmu-miR-328-star	2.63
mmu-miR-344g-5p	1.31
mmu-miR-346-star	14.50

mmu-miR-3472	1.83
mmu-miR-3475	6.99
mmu-miR-34b-5p	3.97
mmu-miR-365-1-star	3.22
mmu-miR-3960	2.92
mmu-miR-449b	1.80
mmu-miR-466a-5p	1.24
mmu-miR-466c-5p	2.27
mmu-miR-466e-3p	1.66
mmu-miR-466f	1.64
mmu-miR-466f-5p	1.77
mmu-miR-466h-5p	1.80
mmu-miR-466i-5p	1.66
mmu-miR-466j	2.24
mmu-miR-466m-5p	1.88
mmu-miR-466q	1.46
mmu-miR-467h	1.24
mmu-miR-5105	5.78
mmu-miR-5109	2.45
mmu-miR-5112	3.65
mmu-miR-5115	12.67
mmu-miR-511-5p	1.45
mmu-miR-5122	9.95
mmu-miR-5126	3.44
mmu-miR-5128	2.13
mmu-miR-5129	1.43
mmu-miR-5130	4.74
mmu-miR-5132	2.79
mmu-miR-665-star	2.80
mmu-miR-666-3p	1.32
mmu-miR-667	1.58
mmu-miR-668	1.31
mmu-miR-669a-3p	2.25
mmu-miR-669a-5p	2.20
mmu-miR-669b	1.80
mmu-miR-669c	2.42
mmu-miR-669d	2.41
mmu-miR-669e	2.45
mmu-miR-669f-5p	1.83
mmu-miR-669l	1.58
mmu-miR-669m-5p	2.01
mmu-miR-669o-3p	2.56
mmu-miR-669o-5p	1.76
mmu-miR-680	2.36
mmu-miR-698	1.85
mmu-miR-705	3.05
mmu-miR-711	7.08
mmu-miR-743a-star	1.34
mmu-miR-762	10.38
mmu-miR-96	1.25
