# Supplementary material for: miR-30 Family miRNAs Mediate the Effect of Chronic Social Defeat Stress on Hippocampal Neurogenesis in Mouse Depression Model
Source: Front Mol Neurosci. 2019 Aug 8;12:188. doi: 10.3389/fnmol.2019.00188 (PMC6694739; doi:10.3389/fnmol.2019.00188)
Supplement: TABLE S6 — The list comprises of miRNAs, which demonstrated decreased expression (fold change ≥1.2 and p ≤ 0.05) in the differentiated cells from the late phase (day 7) when compared with the proliferating neurospheres. [file Table_6.pdf]

Table S6. List of miRNAs, which demonstrated decreased expression (fold change ≥ 1.2 and p ≤ 0.05) in the differentiated cells from the late phase (day 7) when compared with the proliferating neurospheres

Name of the miRNA	Fold Change
mmu-let-7d-star	0.56
mmu-let-7e-star	0.74
mmu-miR-103	0.81
mmu-miR-106a	0.27
mmu-miR-106b	0.54
mmu-miR-106b-star	0.19
mmu-miR-1195	0.60
mmu-miR-1249	0.70
mmu-miR-125a-3p	0.54
mmu-miR-126-3p	0.61
mmu-miR-128	0.51
mmu-miR-1306-3p	0.41
mmu-miR-130b	0.32
mmu-miR-132	0.26
mmu-miR-138	0.48
mmu-miR-139-5p	0.18
mmu-miR-149	0.25
mmu-miR-151-3p	0.25
mmu-miR-15b	0.49
mmu-miR-17	0.24
mmu-miR-17-star	0.23
mmu-miR-181a	0.42
mmu-miR-181b	0.37
mmu-miR-181c	0.44
mmu-miR-181d	0.54
mmu-miR-184	0.22
mmu-miR-187	0.59
mmu-miR-188-5p	0.41
mmu-miR-18a	0.10
mmu-miR-1907	0.44
mmu-miR-190-star	0.61
mmu-miR-191	0.47
mmu-miR-1930	0.73
mmu-miR-1935	0.53
mmu-miR-193-star	0.26
mmu-miR-1946a	0.30
mmu-miR-1946b	0.24
mmu-miR-1965	0.24
mmu-miR-1981	0.53
mmu-miR-1983	0.50
mmu-miR-19b	0.49
mmu-miR-206	0.23
mmu-miR-20a	0.29
mmu-miR-20b	0.32
mmu-miR-212-3p	0.20
mmu-miR-212-5p	0.73

mmu-miR-2136	0.73
mmu-miR-219-3p-star	0.47
mmu-miR-25	0.65
mmu-miR-25-star	0.20
mmu-miR-27a-star	0.77
mmu-miR-28c	0.72
mmu-miR-296-3p	0.54
mmu-miR-297a-star	0.64
mmu-miR-298	0.46
mmu-miR-29b	0.73
mmu-miR-301b	0.59
mmu-miR-3060	0.52
mmu-miR-3074-1-3p	0.54
mmu-miR-3093-3p	0.57
mmu-miR-3093-5p	0.54
mmu-miR-3102	0.30
mmu-miR-322	0.57
mmu-miR-322-star	0.49
mmu-miR-324-3p	0.37
mmu-miR-324-5p	0.44
mmu-miR-325-star	0.26
mmu-miR-328	0.62
mmu-miR-331-3p	0.56
mmu-miR-338-5p	0.32
mmu-miR-342-3p	0.38
mmu-miR-342-5p	0.30
mmu-miR-345-3p	0.31
mmu-miR-345-5p	0.42
mmu-miR-351	0.14
mmu-miR-351-star	0.28
mmu-miR-362-3p	0.76
mmu-miR-362-5p	0.16
mmu-miR-378-star	0.43
mmu-miR-383	0.20
mmu-miR-3968	0.60
mmu-miR-421	0.43
mmu-miR-423-3p	0.10
mmu-miR-425	0.37
mmu-miR-425-star	0.47
mmu-miR-467g	0.75
mmu-miR-483	0.45
mmu-miR-484	0.29
mmu-miR-500	0.51
mmu-miR-501-3p	0.20
mmu-miR-501-5p	0.53
mmu-miR-503	0.30
mmu-miR-5097	0.37
mmu-miR-5099	0.35
mmu-miR-5100	0.27
mmu-miR-5103	0.78

mmu-miR-5110	0.30
mmu-miR-5117	0.62
mmu-miR-532-3p	0.12
mmu-miR-532-5p	0.38
mmu-miR-542-5p	0.52
mmu-miR-652	0.55
mmu-miR-669m-3p	0.69
mmu-miR-671-3p	0.43
mmu-miR-672	0.16
mmu-miR-674	0.49
mmu-miR-674-star	0.29
mmu-miR-676	0.30
mmu-miR-700	0.62
mmu-miR-709	0.66
mmu-miR-720	0.32
mmu-miR-760-3p	0.73
mmu-miR-760-5p	0.65
mmu-miR-9	0.34
mmu-miR-92a	0.13
mmu-miR-92a-1-star	0.60
mmu-miR-93	0.43
mmu-miR-93-star	0.13
mmu-miR-99b	0.30
mmu-miR-99b-star	0.15
