# Supplementary material for: miR-30 Family miRNAs Mediate the Effect of Chronic Social Defeat Stress on Hippocampal Neurogenesis in Mouse Depression Model
Source: Front Mol Neurosci. 2019 Aug 8;12:188. doi: 10.3389/fnmol.2019.00188 (PMC6694739; doi:10.3389/fnmol.2019.00188)
Supplement: TABLE S7 — The list comprises of genes which demonstrated increased expression (fold change ≥1.5 and p ≤ 0.05) in the differentiated cells from an early phase (day 2) when compared with the proliferating neurospheres. [file Table_7.pdf]

Table S7. List of genes, which demonstrated increased expression (fold change ≥ 1.5 and p ≤ 0.05) in the differentiated cells from an early phase (day 2) when compared with the proliferating neurospheres

Gene Symbol	Accession No.	Gene Definition	Fold Change
1010001D01Rik	NM_001081456.1		1.77
1110012J17Rik	XM_130807.1	RIKEN cDNA 1110012J17 gene (1110012J17Rik)	1.88
1110017I16Rik	NM_008876.2	RIKEN cDNA 1110017I16 gene (1110017I16Rik), transcript variant 1	6.15
1110036D12Rik	NM_148927.1		2.83
1190002N15Rik	NM_001001335.1		1.59
1190005I06Rik	NM_013746.1	RIKEN cDNA 1190005I06 gene (1190005I06Rik)	1.93
1190007F08Rik	NM_133244.2	RIKEN cDNA 1190007F08 gene (1190007F08Rik)	2.17
1300010F03Rik	NM_011121.3	PREDICTED: RIKEN cDNA 1300010F03 gene (1300010F03Rik)	1.73
1500015O10Rik	NM_152804.1	RIKEN cDNA 1500015O10 gene (1500015O10Rik)	3.03
1500031L02Rik	NM_011495.1	RIKEN cDNA 1500031L02 gene (1500031L02Rik)	1.50
1520402A20Rik	NM_011123.2		1.51
1700003E16Rik	NM_008880.2	PREDICTED: RIKEN cDNA 1700003E16 gene, transcript variant 1 (1700003E16Rik)	1.81
1700007K13Rik	NM_178711.2	RIKEN cDNA 1700007K13 gene (1700007K13Rik)	2.23
1700009P17Rik	NM_032398.1	RIKEN cDNA 1700009P17 gene (1700009P17Rik)	1.92
1700016J18Rik	NM_028199.2	PREDICTED: RIKEN cDNA 1700016J18 gene (1700016J18Rik)	3.02
1700019G17Rik	NM_008882.2	RIKEN cDNA 1700019G17 gene (1700019G17Rik)	2.06
1700025K23Rik	NM_172775.1	RIKEN cDNA 1700025K23 gene (1700025K23Rik)	1.99
1700027N10Rik	NM_008884.4	RIKEN cDNA 1700027N10 gene (1700027N10Rik)	2.31
1700029J07Rik	NM_008885.2	RIKEN cDNA 1700029J07 gene (1700029J07Rik)	2.46
1700084C01Rik	NM_175498.3	RIKEN cDNA 1700084C01 gene (1700084C01Rik)	2.24
1700088E04Rik	NM_025443.2	RIKEN cDNA 1700088E04 gene (1700088E04Rik)	3.21
1810007P19Rik	NM_013723.2	RIKEN cDNA 1810007P19 gene (1810007P19Rik)	1.58
1810013B01Rik	NM_008893.2		1.66
1810028F09Rik	NM_011131.2		1.55
2210408F11Rik	NM_008894.1		1.69
2310001H12Rik	NM_027196	PREDICTED: RIKEN cDNA 2310001H12 gene, transcript variant 21 (2310001H12Rik)	1.57
2310007A19Rik	NM_011132.1	RIKEN cDNA 2310007A19 gene (2310007A19Rik)	2.52
2310007F21Rik	NM_025882.3	RIKEN cDNA 2310007F21 gene (2310007F21Rik)	1.69
2310014G06Rik	NM_017462.2	RIKEN cDNA 2310014G06 gene (2310014G06Rik)	1.75
2310022B05Rik	NM_009088.2	RIKEN cDNA 2310022B05 gene (2310022B05Rik)	2.93
2310040A07Rik	NM_009087.1	PREDICTED: RIKEN cDNA 2310040A07 gene (2310040A07Rik)	1.71
2310040G07Rik	NM_022811.2		1.51
2310079P10Rik	NM_009089		2.03
2410025L10Rik	NM_026329.2	RIKEN cDNA 2410025L10 gene (2410025L10Rik)	1.98
2610035D17Rik	NM_145632.1	PREDICTED: RIKEN cDNA 2610035D17 gene (2610035D17Rik)	1.96
2610109H07Rik	NM_025901.2	RIKEN cDNA 2610109H07 gene (2610109H07Rik)	1.52
2610524A10Rik	NM_153415.3		2.06
2700046G09Rik	NM_026398.2	PREDICTED: RIKEN cDNA 2700046G09 gene (2700046G09Rik), misc RNA.	1.61
2700060E02Rik	NM_008898.1	RIKEN cDNA 2700060E02 gene (2700060E02Rik)	3.49
2810003C17Rik	NM_011141.2	RIKEN cDNA 2810003C17 gene (2810003C17Rik)	3.09
2810402K13Rik	NM_026438.2		1.74
2810454F19Rik	NM_146141.1		1.56
2810459M11Rik	NM_145610		2.61
2900011O08Rik	NM_008247.2	RIKEN cDNA 2900011O08 gene (2900011O08Rik)	2.30
2900026A02Rik	NM_080555.2		1.64
2900060N12Rik	NM_008904.1		1.55
3010015F07Rik	XM_001002886.2		1.92
3100002J23Rik	NM_008907.1	PREDICTED: RIKEN cDNA 3100002J23 gene, transcript variant 2 (3100002J23Rik)	3.85
3110004L20Rik	NM_011149.2	RIKEN cDNA 3110004L20 gene (3110004L20Rik)	1.63
3110018K01Rik	NM_008908.3		2.26
3110023G01Rik	NM_026352.2		1.56
3110049J23Rik	NM_134084.1	RIKEN cDNA 3110049J23 gene (3110049J23Rik)	1.64
3526401B18Rik	NM_028677		1.60
3830406C13Rik	XM_994766.1	RIKEN cDNA 3830406C13 gene (3830406C13Rik), transcript variant 2	1.54
4432405B04Rik	NM_011151.1	RIKEN cDNA 4432405B04 gene (4432405B04Rik)	1.69
4631416L12Rik	NM_008014.3	RIKEN cDNA 4631416L12 gene (4631416L12Rik)	1.66
4832420L08Rik	NM_001098231.1		1.69
4833409N03Rik	NM_031868.2		2.12
4833414E09Rik	NM_027892.2		1.56
4833420G17Rik	NM_008889.1	RIKEN cDNA 4833420G17 gene (4833420G17Rik)	1.86
4833441J24Rik	NM_144828.1		3.16
4930402H24Rik	NM_146154.1		2.66
4930438O05Rik	NM_017374.3		1.61

4930455F23Rik	NM_027531.1	RIKEN cDNA 4930455F23 gene (4930455F23Rik)	1.64
4930528G09Rik	NM_001081458.1		1.62
4930570C03Rik	NM_008913.1	RIKEN cDNA 4930570C03 gene (4930570C03Rik)	1.68
4931426K16Rik	NM_008914		1.50
4932425I24Rik	NM_024459.2	RIKEN cDNA 4932425I24 gene (4932425I24Rik)	2.01
4932443I19Rik	NM_024209.2	PREDICTED: RIKEN cDNA 4932443I19 gene, transcript variant 1 (4932443I19Rik)	1.93
4933407L21Rik	NM_001081214.1		7.11
4933421G18Rik	NM_172574.1		1.53
4933434I06Rik	NM_145150.1	RIKEN cDNA 4933434I06 gene (4933434I06Rik)	4.54
4933439C20Rik	NM_028243.2	RIKEN cDNA 4933439C20 gene (4933439C20Rik)	1.67
5031425E22Rik	NM_016764.3		1.75
5031439G07Rik	NM_007453.2		2.07
5033414K04Rik	NM_054077.3	RIKEN cDNA 5033414K04 gene (5033414K04Rik)	1.80
5330403J18Rik	NM_011156.2		2.75
5330431K02Rik	NM_011073.2		1.54
5430405G05Rik	NM_008854.3		2.20
5430434G16Rik	NM_011100.3		1.52
5430435G22Rik	NM_145401.1	RIKEN cDNA 5430435G22 gene (5430435G22Rik)	1.80
5730402C02Rik	NM_021880.2		2.22
5730410E15Rik	NM_011158.3	RIKEN cDNA 5730410E15 gene (5730410E15Rik), transcript variant c	2.67
6030410I10Rik	NM_008855.2		2.07
6230400G14Rik	NM_027230.3		2.11
6330403K07Rik	NM_028444.1	RIKEN cDNA 6330403K07 gene (6330403K07Rik)	3.97
6330405H19	NM_008859.1		1.77
6330414G02Rik	NM_008925.1		7.42
6330505N24Rik	NM_029239.2	RIKEN cDNA 6330505N24 gene (6330505N24Rik)	1.98
6430537H07Rik	NM_133740.1	RIKEN cDNA 6430537H07 gene (6430537H07Rik)	2.18
6430548M08Rik	NM_013768.2		2.43
6430550H21Rik	NM_145404.1	RIKEN cDNA 6430550H21 gene (6430550H21Rik)	1.94
7330410H16Rik	NM_011170.1		1.58
7530408C15Rik	NM_011172.1		2.13
8030467N07Rik	NM_021381.3		1.62
9130020G22Rik	AK029921		1.51
9330132O05Rik	NM_197945.2		1.72
9330158N06Rik	NM_134129.2		1.64
9330175B01Rik	NM_172697.1		2.06
9330177P20Rik	NM_178774.3		1.67
9630009A08Rik	NM_028447.2		1.91
9830134K01Rik	NM_178738.2		1.54
9830143E02Rik	NM_011179.2		1.51
9830169C18Rik	NM_028707.3	RIKEN cDNA 9830169C18 gene (9830169C18Rik)	1.53
A230057G18Rik	NM_020261.2		7.18
A230057M07Rik	NM_133948.4		1.84
A330021E22Rik	NM_011967.2	RIKEN cDNA A330021E22 gene (A330021E22Rik)	2.56
A730036E13Rik	NM_011969.1	PREDICTED: RIKEN cDNA A730036E13 gene (A730036E13Rik)	1.96
A830073O21Rik	NM_011185.3		1.70
A830080H07Rik	NM_013640.1		1.82
A830081L15Rik	NM_011970.3		1.93
A830094I09Rik	NM_011971.4		1.50
A930008A22Rik	NM_008945.2		2.08
Abat	NM_011186.1	4-aminobutyrate aminotransferase (Abat), nuclear gene encoding mitochondrial protein	1.85
Abcc12	NM_008946.2	ATP-binding cassette, sub-family C (CFTR/MRP), member 12 (Abcc12)	2.53
Abcc4	NM_011187.1	ATP-binding cassette, sub-family C (CFTR/MRP), member 4 (Abcc4)	1.53
Abcc5	NM_008947	ATP-binding cassette, sub-family C (CFTR/MRP), member 5 (Abcc5), transcript variant 2	2.11
Abhd1	NM_011188.1	abhydrolase domain containing 1 (Abhd1), transcribed RNA.	2.72
Abhd14b	NM_008949.2	abhydrolase domain containing 14b (Abhd14b)	3.79
Abhd3	NM_011874.2	abhydrolase domain containing 3 (Abhd3)	1.54
Ablim2	NM_008950.1	actin-binding LIM protein 2 (Ablim2)	2.39
Acaa2	NM_025894.1		1.66
Acadm	NM_021526	acyl-Coenzyme A dehydrogenase, medium chain (Acadm)	1.73
Acot1	NM_080554.2	acyl-CoA thioesterase 1 (Acot1)	2.16
Acot11	NM_026545.2	acyl-CoA thioesterase 11 (Acot11)	1.67
Acss1	NM_011192.3	acyl-CoA synthetase short-chain family member 1 (Acss1), nuclear gene encoding mitochondrial protein	2.79
Acta2	NM_026873.2	actin, alpha 2, smooth muscle, aorta (Acta2)	3.01
Actn1	NM_008960.2	actinin, alpha 1 (Actn1)	2.43
Adamts16	NM_007982.1	a disintegrin-like and metallopeptidase (reprolysin type) with thrombospondin type 1 motif, 16 (Adamts16)	2.51

Adamts9	NM_008973.2		1.65
Adamtsl1	NM_011203.2		1.62
Adck4	NM_011212.2		1.77
Adcy2	NM_011216.2	adenylate cyclase 2 (Adcy2)	1.63
Adcy8	NM_001081306.1	adenylate cyclase 8 (Adcy8)	3.37
Adora1	NM_030723.1	adenosine A1 receptor (Adora1), transcript variant 1	2.38
Adra2a	NM_008988.2	adrenergic receptor, alpha 2a (Adra2a)	9.74
Agt	NM_172437.2	angiotensinogen (serpin peptidase inhibitor, clade A, member 8) (Agt)	2.47
Agtrap	XM_001476573.1	angiotensin II, type I receptor-associated protein (Agtrap)	1.50
Ahnak	NM_013645.3	AHNAK nucleoprotein (desmoyokin) (Ahnak), transcript variant 1	16.58
AI646023	NM_021495.2	expressed sequence AI646023 (AI646023)	1.68
AI662250	NM_133705.1	expressed sequence AI662250 (AI662250)	2.23
AI875142	NM_153781.1		2.73
Ak3l1	NM_027455.1	adenylate kinase 3-like 1 (Ak3l1), nuclear gene encoding mitochondrial protein	2.19
Akap14	NM_001081054.2	A kinase (PRKA) anchor protein 14 (Akap14)	2.94
Akap8l	NM_008997.3	A kinase (PRKA) anchor protein 8-like (Akap8l)	1.69
Aldh3a2	NM_175543		1.60
Aldh5a1	XM_283428.6		1.97
Aldh6a1	NM_026405.3	aldehyde dehydrogenase family 6, subfamily A1 (Aldh6a1), nuclear gene encoding mitochondrial protein	2.22
Aldoc	NM_033475.2	aldolase C, fructose-bisphosphate (Aldoc)	1.78
Ampd3	NM_198163.1	adenosine monophosphate deaminase 3 (Ampd3)	1.82
Amy1	XM_125709.3	amylase 1, salivary (Amy1)	1.95
Amy2-2	NM_009001.3	amylase 2-2, pancreatic (Amy2-2)	6.05
Angpt1	NM_023537.4	angiopoietin 1 (Angpt1)	1.89
Angptl4	NM_031874.4	angiopoietin-like 4 (Angptl4)	3.71
Ank	XM_980542.1	progressive ankylosis (Ank)	3.15
Anxa11	NM_024456.2	annexin A11 (Anxa11)	1.60
Anxa5	NM_173781.3	annexin A5 (Anxa5)	1.95
Ap3m2	NM_173413.2	adaptor-related protein complex 3, mu 2 subunit (Ap3m2)	3.28
Apoe	NM_011231.1	apolipoprotein E (Apoe)	6.43
Aqp4	NM_145510.1	aquaporin 4 (Aqp4)	5.72
Aqp9	NM_026817.3	aquaporin 9 (Aqp9)	1.85
Arf2	NM_011234.2	ADP-ribosylation factor 2 (Arf2)	1.58
Arhgef4	NM_053269.2	Rho guanine nucleotide exchange factor (GEF) 4 (Arhgef4)	2.73
Armc3	NM_009015.2	armadillo repeat containing 3 (Armc3)	2.27
Armc9	NM_009017.1	armadillo repeat containing 9 (Armc9), transcript variant 1	1.64
Arrdc3	NM_009057.2		2.31
Arsb	NM_011973.2	arylsulfatase B (Arsb)	2.15
Arsg	NM_018740.1	arylsulfatase G (Arsg)	2.02
Asphd2	NM_175211	aspartate beta-hydroxylase domain containing 2 (Asphd2)	1.82
Ass1	NM_023130.2	argininosuccinate synthetase 1 (Ass1)	1.79
Atf3	NM_019444.2	activating transcription factor 3 (Atf3)	1.57
Atg12	NM_009391.3	autophagy-related 12 (yeast) (Atg12)	1.53
Atp1a1	NM_011239.2	ATPase, Na+/K+ transporting, alpha 1 polypeptide (Atp1a1)	2.32
Atp1b1	NM_011241	ATPase, Na+/K+ transporting, beta 1 polypeptide (Atp1b1)	1.99
Atp1b2	NM_021329.2	ATPase, Na+/K+ transporting, beta 2 polypeptide (Atp1b2)	1.83
Atp8a1	NM_024457.2	ATPase, aminophospholipid transporter (APLT), class I, type 8A, member 1 (Atp8a1), transcript variant 2	1.55
Atxn1	NM_172413.2	ataxin 1 (Atxn1)	1.77
AU021034	NM_144850.1	expressed sequence AU021034 (AU021034)	3.33
AW555464	XM_001475793.1		1.75
Axl	NM_025936.1	AXL receptor tyrosine kinase (Axl)	2.04
B230112P13Rik	NM_009025.2		2.64
B230208N19Rik	NM_177644.4	PREDICTED: RIKEN cDNA B230208N19 gene, transcript variant 1 (B230208N19Rik)	1.50
B230311B06Rik	NM_138956.3	PREDICTED: RIKEN cDNA B230311B06 gene (B230311B06Rik)	1.75
B230342M21Rik	NM_178045.3	RIKEN cDNA B230342M21 gene (B230342M21Rik)	1.90
B230342N21Rik	NM_009030.3		1.93
B3galt5	NM_009031.2	UDP-Gal:betaGlcNAc beta 1,3-galactosyltransferase, polypeptide 5 (B3galt5)	1.82
B3gat1	NM_011249.1	beta-1,3-glucuronyltransferase 1 (glucuronosyltransferase P) (B3gat1)	2.24
B930008G03Rik	NM_026453.1		3.44
B930045J24Rik	XM_131139.4		1.58
B9d1	NM_178660.2	B9 protein domain 1 (B9d1)	1.91
Bace2	NM_011252.2	beta-site APP-cleaving enzyme 2 (Bace2)	2.97
BB128963	NM_019712.3		1.55
Bbc3	NM_133878.2	BCL2 binding component 3 (Bbc3)	1.72
Bbox1	NM_021525.2	butyrobetaine (gamma), 2-oxoglutarate dioxygenase 1 (gamma-butyrobetaine hydroxylase) (Bbox1)	3.46
Bbs2	NM_021557	Bardet-Biedl syndrome 2 (human) (Bbs2)	1.58

Bbs4	NM_134006.4	Bardet-Biedl syndrome 4 (human) (Bbs4)	1.81
BC022687	NM_011261.2	cDNA sequence BC022687 (BC022687)	1.60
BC024537	NM_009048.1		1.69
BC030476	NM_181988.1	cDNA sequence BC030476 (BC030476)	3.77
BC031353	NM_026159.4	cDNA sequence BC031353 (BC031353)	1.51
BC038167	NM_011264.3		2.21
BC050811	NM_011258.1	cDNA sequence BC050811 (BC050811)	2.20
Bcar3	NM_020022.2	breast cancer anti-estrogen resistance 3 (Bcar3)	1.62
Bcas3	XM_132528.3	breast carcinoma amplified sequence 3 (Bcas3)	2.44
Bcl2l11	NM_145480	BCL2-like 11 (apoptosis facilitator) (Bcl2l11), transcript variant 1	1.62
Bdh2	NM_028128.1	3-hydroxybutyrate dehydrogenase, type 2 (Bdh2)	2.82
Bicc1	NM_028713.1	bicaudal C homolog 1 (Drosophila) (Bicc1)	1.70
Birc1cl	NM_009056.1		2.18
Bmpr1b	NM_016846.3	bone morphogenetic protein receptor, type 1B (Bmpr1b)	1.59
Bzrap1	NM_009059.2	benzodiazapine receptor associated protein 1 (Bzrap1)	2.98
C030009J22Rik	NM_177740.4		5.30
C030027H14Rik	NM_009061		1.65
C030048H21Rik	NM_009062.3	PREDICTED: RIKEN cDNA C030048H21 gene (C030048H21Rik)	1.70
C130023A14Rik	NM_011880.2		1.54
C130072A16Rik	NM_029879.2		2.02
C1ql2	NM_010117.1	complement component 1, q subcomponent-like 2 (C1ql2)	2.44
C230070D10Rik	NM_183163.2		2.48
C3	NM_007483.2		4.22
C430004E15Rik	NM_153514.4	RIKEN cDNA C430004E15 gene (C430004E15Rik)	1.70
C4a	NM_007484.1	complement component 4A (Rodgers blood group) (C4a)	5.55
C4b	NM_023275.2	complement component 4B (Childo blood group) (C4b)	5.11
C530044C16Rik	NM_145999.2		3.69
C630002C17Rik	NM_008164.1		5.48
Cachd1	NM_027897.3	cache domain containing 1 (Cachd1)	2.15
Cacna2d3	NM_028724.2	calcium channel, voltage-dependent, alpha2/delta subunit 3 (Cacna2d3)	2.04
Cacnb4	NM_024182.4	calcium channel, voltage-dependent, beta 4 subunit (Cacnb4), transcript variant 2	1.71
Camk2g	NR_003278.1	calcium/calmodulin-dependent protein kinase II gamma (Camk2g)	1.87
Capn1	NM_011271.2	calpain 1 (Capn1)	1.50
Capn5	NM_026001.2	calpain 5 (Capn5)	1.55
Carhsp1	NM_020012.1	calcium regulated heat stable protein 1 (Carhsp1)	1.62
Casq2	NM_028862.2	calsequestrin 2 (Casq2)	1.76
Cat	NM_183204.2	catalase (Cat)	1.51
Cc2d2a	NM_153762.3	coiled-coil and C2 domain containing 2A (Cc2d2a)	1.54
Ccdc19	NM_011278.3	PREDICTED: coiled-coil domain containing 19, transcript variant 1 (Ccdc19)	1.77
Ccdc39	NM_134064.1	coiled-coil domain containing 39 (Ccdc39)	2.07
Ccdc96	NM_009070.1	coiled-coil domain containing 96 (Ccdc96)	1.70
Ccrk	NM_019413.2	cell cycle related kinase (Ccrk)	1.84
Cd109	NM_026632.3	CD109 antigen (Cd109)	1.67
Cd59a	NM_011285.1	CD59a antigen (Cd59a)	3.91
Cd82	NM_011287.1	CD82 antigen (Cd82)	1.67
Cdc42ep4	NM_009076.1	CDC42 effector protein (Rho GTPase binding) 4 (Cdc42ep4)	2.24
Cdo1	NM_009438.3	cysteine dioxygenase 1, cytosolic (Cdo1)	2.18
Celsr1	NM_009077.2	cadherin, EGF LAG seven-pass G-type receptor 1 (flamingo homolog, Drosophila) (Celsr1)	1.85
Cercam	NM_029751.3	cerebral endothelial cell adhesion molecule (Cercam)	1.51
Cetn4	NM_009078.1	centrin 4 (Cetn4)	2.47
Cfp	NM_022891.2	complement factor properdin (Cfp)	1.52
Chi3l1	NM_024218.2	chitinase 3-like 1 (Chi3l1)	2.26
Chka	NM_009080.2	choline kinase alpha (Chka), transcript variant 2	1.86
Ckap5	NM_011289.1	cytoskeleton associated protein 5 (Ckap5)	1.56
Cldn11	NM_011975.3	claudin 11 (Cldn11)	1.73
Clip4	NM_009081	CAP-GLY domain containing linker protein family, member 4 (Clip4)	1.53
Clk1	NM_009082.2	CDC-like kinase 1 (Clk1), transcript variant 1	1.51
Clk4	NM_013762.1	CDC like kinase 4 (Clk4)	1.53
Cml1	NM_009083.2	camello-like 1 (Cml1)	2.41
Cml4	NM_053257.1	camello-like 4 (Cml4)	2.19
Cmtm5	NM_172086	CKLF-like MARVEL transmembrane domain containing 5 (Cmtm5)	4.93
Cnp	NM_026724.1	2',3'-cyclic nucleotide 3' phosphodiesterase (Cnp)	2.20
Cnp1	NM_025592.3		1.63
Col22a1	NM_019865.2	PREDICTED: collagen, type XXII, alpha 1, transcript variant 3 (Col22a1)	2.65
Col23a1	NM_025589.1	procollagen, type XXIII, alpha 1 (Col23a1)	1.53
Col8a2	NM_026069.2		1.79
Copg2as2	NM_001048057.1		1.63
Cpe	NM_026055.1		1.80
Cryab	NM_024212.2	crystallin, alpha B (Cryab)	2.68

Crygs	NM_018860.3	crystallin, gamma S (Crygs)	1.70
Csgalnact1	NM_025433.3	chondroitin sulfate N-acetylgalactosaminyltransferase 1 (Csgalnact1)	1.73
Cspp1	NM_011292.1	centrosome and spindle pole associated protein 1 (Cspp1)	1.67
Cst3	NM_007475.4	cystatin C (Cst3)	1.77
Ctdsp2	NM_018853.3	CTD (carboxy-terminal domain, RNA polymerase II, polypeptide A) small phosphatase 2 (Ctdsp2)	1.53
Ctnna2	NM_026020.4	catenin (cadherin associated protein), alpha 2 (Ctnna2), transcript variant 2	1.95
Ctnnal1	NM_019642.3	catenin (cadherin associated protein), alpha-like 1 (Ctnnal1)	2.57
Ctsa	NM_026308.2	cathepsin A (Ctsa), transcript variant 2	3.16
Cxcr4	NM_023396.4		3.19
Cyhr1	NM_025963.1	cysteine and histidine rich 1 (Cyhr1), transcript variant 2	2.11
Cyp1b1	NM_013725.3	cytochrome P450, family 1, subfamily b, polypeptide 1 (Cyp1b1)	2.64
Cyp4f13	NM_011295.2	cytochrome P450, family 4, subfamily f, polypeptide 13 (Cyp4f13)	1.79
Cyp4f14	NM_026533.1	cytochrome P450, family 4, subfamily f, polypeptide 14 (Cyp4f14)	2.86
Cyp4v3	NM_009091.1	cytochrome P450, family 4, subfamily v, polypeptide 3 (Cyp4v3)	2.08
Cyp7b1	NM_013647.1	cytochrome P450, family 7, subfamily b, polypeptide 1 (Cyp7b1)	2.17
D030034I04Rik	NM_023133.1		2.25
D0H4S114	NM_008503.4	DNA segment, human D4S114 (D0H4S114)	5.11
D10Ertd610e	NM_025587.2	DNA segment, Chr 10, ERATO Doi 610, expressed (D10Ertd610e)	1.87
D16H22S680E	NM_011297.2	DNA segment, Chr 16, human D22S680E, expressed (D16H22S680E)	1.67
D230005E09Rik	NM_024266.3		2.02
D330014H01Rik	NM_013765.1		1.56
D430042O09Rik	NM_001033865.1	RIKEN cDNA D430042O09 gene (D430042O09Rik)	2.15
D4Ertd681e	NM_026467.2		2.51
D5Ertd579e	NM_009093.1	DNA segment, Chr 5, ERATO Doi 579, expressed (D5Ertd579e)	1.52
D630004N19Rik	NM_012052.1		1.52
D930015E06Rik	NM_016959.2		1.58
D9Ertd392e	NM_009094.1		1.82
Dapk1	NM_009095.1	death associated protein kinase 1 (Dapk1), transcript variant 2	1.79
Darc	NM_009096.2	Duffy blood group, chemokine receptor (Darc)	2.09
Ddb2	NM_009097.1	damage specific DNA binding protein 2 (Ddb2)	1.59
Ddo	NM_153587.2	D-aspartate oxidase (Ddo)	4.35
Ddr1	NM_011300.2	discoidin domain receptor family, member 1 (Ddr1), transcript variant 1	2.05
Ddx3y	NM_009098.2	DEAD (Asp-Glu-Ala-Asp) box polypeptide 3, Y-linked (Ddx3y)	1.66
Dhrs1	NM_029767.2	dehydrogenase/reductase (SDR family) member 1 (Dhrs1)	3.90
Dhx32	NM_133626.2	DEAH (Asp-Glu-Ala-His) box polypeptide 32 (Dhx32)	1.66
Dido1	NM_009103	death inducer-obliterator 1 (Dido1), transcript variant 1	1.59
Diras1	NM_009104.1	DIRAS family, GTP-binding RAS-like 1 (Diras1)	1.52
Dixdc1	NM_199447.2		1.64
Dlgap1	NM_026041.2	discs, large (Drosophila) homolog-associated protein 1 (Dlgap1)	1.76
Dm15	NM_028244.1		1.81
Dnajb13	NM_145620.4	DnaJ (Hsp40) related, subfamily B, member 13 (Dnajb13)	3.02
Dnajb2	NM_001013381.1	DnaJ (Hsp40) homolog, subfamily B, member 2 (Dnajb2), transcript variant 2	1.50
Dnajb9	XM_137041.6	DnaJ (Hsp40) homolog, subfamily B, member 9 (Dnajb9)	1.74
Dnajc28	NM_025546.2	DnaJ (Hsp40) homolog, subfamily C, member 28 (Dnajc28), transcript variant 2	1.52
Dnali1	NM_025290.3	dynein, axonemal, light intermediate polypeptide 1 (Dnali1)	2.49
Dner	NM_001081346.1	delta/notch-like EGF-related receptor (Dner)	1.72
Dpysl4	NM_153457.6	dihydropyrimidinase-like 4 (Dpysl4)	1.97
Dpysl5	NM_001003930	dihydropyrimidinase-like 5 (Dpysl5)	2.35
Drp2	NM_177708.5	dystrophin related protein 2 (Drp2)	5.58
Dscr1	NM_019685.1		1.72
Dtx4	NM_011304.3	deltex 4 homolog (Drosophila) (Dtx4)	1.55
Dync2li1	NM_025614.1	dynein cytoplasmic 2 light intermediate chain 1 (Dync2li1)	1.96
Dynlrb2	NM_019743.3	dynein light chain roadblock-type 2 (Dynlrb2)	4.93
Dysf	NM_001042607.1	dysferlin (Dysf), transcript variant 1	3.34
E030030K01Rik	NM_011309.3		1.90
E030049G20Rik	NM_011313.2	RIKEN cDNA E030049G20 gene (E030049G20Rik), transcript variant 1	1.73
E130203B14Rik	XM_620495.3	RIKEN cDNA E130203B14 gene (E130203B14Rik)	2.68
E130308A19Rik	NM_030692.1	RIKEN cDNA E130308A19 gene (E130308A19Rik), transcript variant 2	1.58
E330009J07Rik	NM_019748.2		1.66
E430002G05Rik	NM_178280.3		2.03
Ech1	XM_620286.3	enoyl coenzyme A hydratase 1, peroxisomal (Ech1)	1.68
Echdc2	NM_021788.1	enoyl Coenzyme A hydratase domain containing 2 (Echdc2)	2.00
Ednra	NM_016926.1	endothelin receptor type A (Ednra)	1.84
Ednrb	NM_025436.1	endothelin receptor type B (Ednrb)	1.51
EG232599	NM_009127.3	predicted gene, EG232599 (EG232599)	3.28
EG546143	NM_183216.3	PREDICTED: predicted gene, EG546143 (EG546143)	2.35
EG632802	NM_009130.1	PREDICTED: predicted gene, EG632802 (EG632802)	1.95
EG665378	NM_009162.3	predicted gene, EG665378 (EG665378)	1.90
Emp2	NM_001099742.1	epithelial membrane protein 2 (Emp2)	1.80
Endod1	AK002910.1	endonuclease domain containing 1 (Endod1)	2.02

Enpp5	AK021409.1	ectonucleotide pyrophosphatase/phosphodiesterase 5 (Enpp5)	1.51
Entpd2	AK031598.1	ectonucleoside triphosphate diphosphohydrolase 2 (Entpd2)	5.93
Entpd5	AK050665.1	ectonucleoside triphosphate diphosphohydrolase 5 (Entpd5), transcript variant 2	1.59
Ephb1	AK017012.1	Eph receptor B1 (Ephb1)	1.81
Ephx2	XM_358429.1	epoxide hydrolase 2, cytoplasmic (Ephx2)	1.50
F730003H07Rik	AK083573.1		2.00
F830002E14Rik	AK019418.1		4.57
Fads3	AK087461.1	fatty acid desaturase 3 (Fads3)	1.77
Fah		fumarylacetoacetate hydrolase (Fah)	1.71
Fam102a	AK076154.1	family with sequence similarity 102, member A (Fam102a)	2.02
Fam134b	AK081191.1	family with sequence similarity 134, member B (Fam134b), transcript variant 2	6.45
Fam184a	NM_172938.2	family with sequence similarity 184, member A (Fam184a)	2.68
Fam20a	NM_019708.3	family with sequence similarity 20, member A (Fam20a)	1.82
Fank1	NM_007926.2	fibronectin type 3 and ankyrin repeat domains 1 (Fank1)	1.62
Fas	NM_008304.2	Fas (TNF receptor superfamily member 6) (Fas)	4.48
Fbxo2	NM_011520.3	F-box protein 2 (Fbxo2)	12.89
Fbxo21	NM_023374.3	F-box protein 21 (Fbxo21)	1.52
Fbxo25	NM_025848.2	F-box protein 25 (Fbxo25)	1.52
Fbxo32	NM_138741.1	F-box protein 32 (Fbxo32)	1.65
Fbxo36	NM_019951.1	F-box protein 36 (Fbxo36)	1.52
Fbxo41	NM_025468.2	F-box protein 41 (Fbxo41)	1.64
Fbxo44	NM_024206.4	F-box protein 44 (Fbxo44)	1.67
Fcgrt	NM_011343.2	Fc receptor, IgG, alpha chain transporter (Fcgrt)	1.75
Fer1l3	NM_153055.2	PREDICTED: fer-1-like 3, myoferlin (C. elegans), transcript variant 1 (Fer1l3)	1.54
Fez1	NM_001039088.1	fasciculation and elongation protein zeta 1 (zygin I) (Fez1)	1.81
Fez2	NM_009152.2		1.60
Fhl1	NM_011976.1	four and a half LIM domains 1 (Fhl1), transcript variant 1	2.46
Flrt2	NM_009154.1	fibronectin leucine rich transmembrane protein 2 (Flrt2)	1.51
Fmn2	NM_172537.2	formin 2 (Fmn2)	2.37
Fn3k	NM_025814.1	fructosamine 3 kinase (Fn3k), transcript variant 1	1.66
Fos	NM_011354.2	FBJ osteosarcoma oncogene (Fos)	2.70
Foxj1	NM_009252.2	forkhead box J1 (Foxj1)	1.50
Fstl1	NM_009254.2	follistatin-like 1 (Fstl1)	1.62
Fyco1	NM_009255.2	FYVE and coiled-coil domain containing 1 (Fyco1)	1.64
Fzd6	NM_001013370.1		1.57
Gab1	AK011630	growth factor receptor bound protein 2-associated protein 1 (Gab1)	2.36
Gabarapl1	NM_030241.2	gamma-aminobutyric acid (GABA(A)) receptor-associated protein-like 1 (Gabarapl1)	1.54
Gabrb1	NM_026175.4	gamma-aminobutyric acid (GABA-A) receptor, subunit beta 1 (Gabrb1)	1.58
Galntl1	NM_029157.3	UDP-N-acetyl-alpha-D-galactosamine:polypeptide N-acetylgalactosaminyltransferase-like 1 (Galntl1)	4.11
Ganc	NM_133953.2	glucosidase, alpha; neutral C (Ganc)	1.71
Garnl3	NM_153053.3	GTPase activating RANGAP domain-like 3 (Garnl3)	3.90
Gats	NM_175102		1.64
Gbp2	NM_173374.3	guanylate binding protein 2 (Gbp2)	4.89
Gca	NM_009186.4	grancalcin (Gca)	1.85
Gcnt2	NM_001093753.1	glucosaminyl (N-acetyl) transferase 2, I-branching enzyme (Gcnt2), transcript variant 3	1.63
Gdap1l1	NM_011358.1	ganglioside-induced differentiation-associated protein 1-like 1 (Gdap1l1)	1.58
Gdpd2	NM_020587.1	glycerophosphodiester phosphodiesterase domain containing 2 (Gdpd2)	2.86
Gfap	NM_025573.3		16.27
Ggnbp1	NM_145512.3	gametogenetin binding protein 1 (Ggnbp1)	2.36
Gja1	NM_027324.4	gap junction membrane channel protein alpha 1 (Gja1)	2.33
Glb1l	NM_178639.2		1.58
Glipr2	NM_011361.1		2.09
Glud1	NM_133220.2		1.75
Glycam1	NM_028232.1	glycosylation dependent cell adhesion molecule 1 (Glycam1)	3.23
Gm973	NM_199007.1	gene model 973, (NCBI) (Gm973)	2.59
Gmpr	NM_030750.2	guanosine monophosphate reductase (Gmpr)	2.12
Gnao1	NM_024480.3	guanine nucleotide binding protein, alpha O (Gnao1), transcript variant A	1.62
Got1l1	NM_019464.2	glutamic-oxaloacetic transaminase 1-like 1 (Got1l1)	1.63
Gpam	NM_139302.1	glycerol-3-phosphate acyltransferase, mitochondrial (Gpam), nuclear gene encoding mitochondrial protein	1.98
Gpld1	NM_177364.3	glycosylphosphatidylinositol specific phospholipase D1 (Gpld1)	1.54
Gpm6b	NM_011368.3		1.58
Gpr123	NM_175259.4	G protein-coupled receptor 123 (Gpr123)	1.64
Gpr137b-ps	NM_009171	G protein-coupled receptor 137B, pseudogene (Gpr137b-ps), non-coding RNA.	1.60
Gpr17	NM_001077596.1	G protein-coupled receptor 17 (Gpr17)	1.72
Gpr23	NM_172257.3	G protein-coupled receptor 23 (Gpr23)	2.09
Gpr37l1	NM_001081337.1	G protein-coupled receptor 37-like 1 (Gpr37l1)	2.24
Gprc5b	NM_022432.3	G protein-coupled receptor, family C, group 5, member B (Gprc5b)	2.00

Gpx1	NM_153056.1	glutathione peroxidase 1 (Gpx1)	2.17
Grhpr	NM_013929.1	glyoxylate reductase/hydroxypyruvate reductase (Grhpr)	1.62
Gria1	NM_001039090.1	glutamate receptor, ionotropic, AMPA1 (alpha 1) (Gria1)	5.29
Grk4	NM_028151.2	G protein-coupled receptor kinase 4 (Grk4), transcript variant 1	1.52
Gsta3	NM_145468.1	glutathione S-transferase, alpha 3 (Gsta3), transcript variant 2	1.70
Gsta4	NM_153567.1	glutathione S-transferase, alpha 4 (Gsta4)	1.73
H13	NM_013612.1	histocompatibility 13 (H13)	1.98
H2-D1	NM_009194.2	histocompatibility 2, D region locus 1 (H2-D1)	1.53
H3f3b	NM_030683	PREDICTED: H3 histone, family 3B (H3f3b)	1.62
Hbp1	XM_147213.1		1.63
Hc	NM_025807.1	hemolytic complement (Hc)	2.00
Hdc	NM_054087.2	histidine decarboxylase (Hdc)	2.58
Hexim1	NM_018861.2	hexamethylene bis-acetamide inducible 1 (Hexim1)	1.50
Hey1	NM_019687.3	hairy/enhancer-of-split related with YRPW motif 1 (Hey1)	1.65
Hhatl	NM_024211.2	hedgehog acyltransferase-like (Hhatl)	1.62
Hist1h2ac	NM_001081048.1	histone cluster 1, H2ac (Hist1h2ac)	1.52
Hist1h2be	NM_026071.2	histone cluster 1, H2be (Hist1h2be)	2.76
Hist2h2aa1	NM_146118.2	histone cluster 2, H2aa1 (Hist2h2aa1)	1.88
Hist2h2aa2	NM_133668.2	histone cluster 2, H2aa2 (Hist2h2aa2)	1.88
Hopx	NM_144793.1	HOP homeobox (Hopx)	5.50
Hrsp12	NM_026542.1	heat-responsive protein 12 (Hrsp12)	2.59
Hsd3b2	NM_007451.3	hydroxy-delta-5-steroid dehydrogenase, 3 beta- and steroid delta-isomerase 2 (Hsd3b2)	2.47
Hspa2	NM_022880.1	heat shock protein 2 (Hspa2), transcript variant 1	2.20
Hspb8	NM_023596.3	heat shock protein 8 (Hspb8)	9.69
Htatip2	NM_011400.2	HIV-1 tat interactive protein 2, homolog (human) (Htatip2)	1.53
Iap	NM_022885.2		1.63
Id4	NM_023214.3	inhibitor of DNA binding 4 (Id4)	4.20
Idb2	NM_175090.3		2.04
Ifit3	NM_078484.1	interferon-induced protein with tetratricopeptide repeats 3 (Ifit3)	1.77
Ifitm1	NM_144893.1	interferon induced transmembrane protein 1 (Ifitm1)	1.65
Ifngr2	NM_177766.2	interferon gamma receptor 2 (Ifngr2)	2.21
Ift122	NM_175434.3	intraflagellar transport 122 homolog (Chlamydomonas) (Ift122)	1.65
Ift81	NM_024249.4	intraflagellar transport 81 homolog (Chlamydomonas) (Ift81)	1.56
Igfbp5	NM_175121.3	insulin-like growth factor binding protein 5 (Igfbp5)	17.37
Igfbp7	NM_001012305.1	insulin-like growth factor binding protein 7 (Igfbp7)	3.02
Igfbpl1	NM_139143.2	insulin-like growth factor binding protein-like 1 (Igfbpl1)	3.81
Igsf11	NM_008202.2	immunoglobulin superfamily, member 11 (Igsf11)	7.57
Iigp2	NM_177388.3	interferon inducible GTPase 2 (Iigp2)	1.63
Il1r2	NM_133891.2	interleukin 1 receptor, type II (Il1r2)	1.62
Il6st	NM_152808.2	interleukin 6 signal transducer (Il6st)	2.87
Ing4	NM_027872.3	inhibitor of growth family, member 4 (Ing4)	1.57
Inpp5k	NM_018760.1	inositol polyphosphate 5-phosphatase K (Inpp5k)	1.56
Insl6	XM_147798.4	insulin-like 6 (Insl6)	1.62
Iqcb1	NM_021530.2	IQ calmodulin-binding motif containing 1 (Iqcb1)	1.53
Iqcg	NM_178703.2	IQ motif containing G (Iqcg)	1.69
Itga3	NM_009320.3	integrin alpha 3 (Itga3)	2.52
Itgb5	NM_008135.4	integrin beta 5 (Itgb5)	1.56
Itih3	NM_011406.1	inter-alpha trypsin inhibitor, heavy chain 3 (Itih3)	2.05
Itm2b	NM_032008.3	integral membrane protein 2B (Itm2b)	2.21
Itpk1	NM_025531.2	inositol 1,3,4-triphosphate 5/6 kinase (Itpk1)	1.53
Itpkb	NM_025690.3		2.19
Jak1	NM_016769	Janus kinase 1 (Jak1)	2.07
Jakmip1	NM_133716.2	janus kinase and microtubule interacting protein 1 (Jakmip1)	1.82
Kank4	NM_019710.1	KN motif and ankyrin repeat domains 4 (Kank4)	2.32
Kcna6	NM_011420.2		4.43
Kcnk1	NM_022316.1	potassium channel, subfamily K, member 1 (Kcnk1)	1.51
Kcnk2	NM_013870.2	potassium channel, subfamily K, member 2 (Kcnk2)	1.64
Kctd5	NM_026796.1	potassium channel tetramerisation domain containing 5 (Kctd5)	1.69
Kif21a	NM_172339.2	kinesin family member 21A (Kif21a)	2.27
Klhdc8b	NM_009221.2	kelch domain containing 8B (Klhdc8b)	2.21
Klhl26	NR_002898.1	kelch-like 26 (Drosophila) (Klhl26), transcript variant 1	1.59
Kndc1	NM_198214.2	kinase non-catalytic C-lobe domain (KIND) containing 1 (Kndc1)	4.05
Ky	NM_001046637.1	kyphoscoliosis peptidase (Ky)	3.35
Lamb2	NM_009225.2	laminin, beta 2 (Lamb2)	1.82
Lamp2	NM_009226.4	lysosomal-associated membrane protein 2 (Lamp2), transcript variant 1	1.68
Lgals3	NM_026943.1	lectin, galactose binding, soluble 3 (Lgals3)	4.42
Lgals4	NM_026095.4	lectin, galactose binding, soluble 4 (Lgals4)	2.34
Lgals8	XM_892627.2	lectin, galactose binding, soluble 8 (Lgals8)	1.60
Lgi3	NM_009228.1	leucine-rich repeat LGI family, member 3 (Lgi3)	1.60
Lgsn	NM_172951.1	lengsin, lens protein with glutamine synthetase domain (Lgsn)	1.65

Lhfp	NM_025664.5	lipoma HMGIC fusion partner (Lhfp)	1.52
Limk2	NM_175407.3	LIM motif-containing protein kinase 2 (Limk2), transcript variant 3	1.94
Lix1	NM_007706.3	limb expression 1 homolog (chicken) (Lix1)	1.96
Llgl1	NM_019654.2	lethal giant larvae homolog 1 (Drosophila) (Llgl1)	1.59
LOC100041388	NM_013671.2	PREDICTED: similar to ORF1 (LOC100041388)	1.78
LOC100043798	NM_001034964.1	PREDICTED: hypothetical protein LOC100043798 (LOC100043798)	1.60
LOC100043821	NM_021377.1	PREDICTED: hypothetical protein LOC100043821 (LOC100043821)	2.39
LOC100044124	NM_030889.2	PREDICTED: similar to Nedd4 binding protein 2 (LOC100044124)	1.79
LOC100044177	NM_011436.3	PREDICTED: hypothetical protein LOC100044177 (LOC100044177)	2.17
LOC100045005	NM_009234.3	PREDICTED: similar to Deltex3 (LOC100045005), misc RNA.	1.84
LOC100045501	NM_177753.2	PREDICTED: hypothetical protein LOC100045501 (LOC100045501)	1.82
LOC100045680	NM_011444.1	PREDICTED: similar to complement C4 (LOC100045680)	1.59
LOC100045869	NM_011448	PREDICTED: similar to Limb expression 1 homolog (chicken) (LOC100045869)	2.90
LOC100046120	NM_012031.1	PREDICTED: similar to clusterin (LOC100046120)	8.74
LOC100046207	NM_017407.1	PREDICTED: similar to Lymphocyte antigen 6H precursor (Ly-6H) (LOC100046207)	2.59
LOC100046741	NM_015773.1	PREDICTED: similar to red-1 (LOC100046741)	3.42
LOC100046770	NM_172561.1	PREDICTED: similar to histone macroH2A1.2 (LOC100046770)	1.80
LOC100047126	NM_010097.2	PREDICTED: similar to Sctr protein, transcript variant 1 (LOC100047126)	1.66
LOC100047193	NM_016962.1	PREDICTED: similar to kinesin family member 9 (LOC100047193), misc RNA.	1.67
LOC100047214	XM_147847.4	PREDICTED: similar to PTEN induced putative kinase 1 (LOC100047214)	2.84
LOC100047226	NM_026470.3	PREDICTED: hypothetical protein LOC100047226 (LOC100047226), misc RNA.	1.54
LOC100047264	NM_025565.1	PREDICTED: hypothetical protein LOC100047264 (LOC100047264)	1.90
LOC100047674	NM_029701.1	PREDICTED: similar to solute carrier family 35 (UDP-glucuronic acid/UDP-N-acetylgalactosamine dual transporter), member D1 (LOC100047674)	1.81
LOC100047738	NM_001029936.2	PREDICTED: similar to DENN/MADD domain containing 1A (LOC100047738)	2.03
LOC100047936	NM_153176.4	PREDICTED: similar to sortilin-related receptor, LDLR class A repeats-containing (LOC100047936), misc RNA.	2.06
LOC100048083	NM_011451.2	PREDICTED: similar to Tect2 (LOC100048083), misc RNA.	1.75
LOC100048331	XM_207079.2	PREDICTED: similar to DnaJ (Hsp40) homolog, subfamily A, member 4 (LOC100048331), misc RNA.	1.51
LOC100048534	NM_013675.3	PREDICTED: similar to D19Ertd652e protein (LOC100048534)	1.54
LOC270589	NM_025287.2		1.71
LOC331595	NM_009263.1		1.59
LOC333331	NM_033524	PREDICTED: similar to medium-chain acyl-CoA dehydrogenase (LOC333331), misc RNA.	1.57
LOC380653	NM_011898.2		2.05
LOC381000	NM_145134.2		2.07
LOC381132	NM_009270.3		1.68
LOC381260	NM_033218.1		2.02
LOC381738	XM_977299.1		2.65
LOC381946	NM_080448.4		1.72
LOC382163	NM_175347.4		2.60
LOC383154	NM_009272.2		1.73
LOC385256	NM_016795.3		2.22
LOC385274	NM_019684.1		2.10
LOC385825	NM_026130.1		1.78
LOC385877	NM_009275.4		1.56
LOC385959	AK080830		1.54
LOC385992	NM_175229.3		1.55
LOC386005	XM_356132.1		2.42
LOC386021	NM_023464.2		1.69
LOC386078	NM_026155.1		2.04
LOC386144	NM_182990.2		1.88
LOC386218	NM_020491.4		1.62
LOC386256	NM_009217.1		1.99
LOC386268	NM_026899.2		1.61
LOC386298	NM_133726.2		1.56
LOC386360	NM_009176.2		1.84
LOC633360	NM_029811.2	PREDICTED: hypothetical LOC633360 (LOC633360)	1.88
LOC638935	NM_145933.3	PREDICTED: similar to peptidylarginine deiminase, type II (LOC638935)	2.11
LOC667370	NM_012028.2	PREDICTED: similar to interferon-induced protein with tetratricopeptide repeats 3 (LOC667370)	1.81
LOC675572	NM_009181.1	PREDICTED: hypothetical LOC675572 (LOC675572)	3.54
LOC677369	NM_138672.2	PREDICTED: similar to Alpha-2-macroglobulin (LOC677369), misc RNA.	2.26
LOC677375	NM_001077712.1	PREDICTED: hypothetical LOC677375 (LOC677375)	2.15
Lrp2	NM_029682.4	low density lipoprotein receptor-related protein 2 (Lrp2)	1.97
Lrrc36	NM_146258.1	leucine rich repeat containing 36 (Lrrc36)	1.61
Lrrc48	NM_133774.4	leucine rich repeat containing 48 (Lrrc48)	1.93
Lrrc51	NM_199018.1	PREDICTED: leucine rich repeat containing 51, transcript variant 2 (Lrrc51)	2.23
Lrrc8	NM_025303.2		1.92

Lrrn1	NM_009285.3	leucine rich repeat protein 1, neuronal (Lrrn1)	1.72
Luc7l2	NM_009287.4		1.54
Luzp2	NM_001081103.1	leucine zipper protein 2 (Luzp2)	1.66
Lxn	NM_016737.1	latexin (Lxn)	2.25
Lypd1	NM_133810.2	Ly6/Plaur domain containing 1 (Lypd1)	1.55
Macrod1	NM_175031.3	MACRO domain containing 1 (Macrod1)	1.71
Man1c1	NM_011499.2		1.54
Map1lc3b	NM_133789.2	microtubule-associated protein 1 light chain 3 beta (Map1lc3b)	1.56
Map3k7ip2	NM_024222.2	mitogen-activated protein kinase kinase kinase 7 interacting protein 2 (Map3k7ip2)	2.50
Mapk8ip1	NM_009294.3	mitogen activated protein kinase 8 interacting protein 1 (Mapk8ip1)	1.58
Mapk8ip2	NM_009295.1	mitogen-activated protein kinase 8 interacting protein 2 (Mapk8ip2)	1.53
Mapt	NM_011507.1		1.96
March2	NM_172695.2	membrane-associated ring finger (C3HC4) 2 (March2)	1.75
Matn4	NM_172294.1	matrilin 4 (Matn4)	3.24
Mboat2	NM_028072.4	membrane bound O-acyltransferase domain containing 2 (Mboat2)	1.58
Mdh1b	NM_133354.1	malate dehydrogenase 1B, NAD (soluble) (Mdh1b)	1.92
Mfsd7b	NM_019929.3	major facilitator superfamily domain containing 7B (Mfsd7b)	2.23
Mgll	NM_178652.2	monoglyceride lipase (Mgll)	2.11
Mgst1	NM_181423.2	microsomal glutathione S-transferase 1 (Mgst1)	1.55
Mia1	NM_199196.1		1.68
Mid1ip1	NM_022030.3	Mid1 interacting protein 1 (gastrulation specific G12-like (zebrafish)) (Mid1ip1)	1.77
Mks1	NM_009302.2	Meckel syndrome, type 1 (Mks1)	1.55
Mlc1	NM_025932.1	megalencephalic leukoencephalopathy with subcortical cysts 1 homolog (human) (Mlc1)	1.67
Mlf1	XM_359260.3		1.57
Mll5	NM_013681.1		1.61
Mmd	NM_019796.4	monocyte to macrophage differentiation-associated (Mmd)	1.51
Mmd2	NM_183312.3	monocyte to macrophage differentiation-associated 2 (Mmd2)	7.44
Mmp2	NM_030245.2	matrix metallopeptidase 2 (Mmp2)	1.81
Mmp24	NM_020024.3	matrix metallopeptidase 24 (Mmp24)	1.62
Morn5	BC019668	MORN repeat containing 5 (Morn5)	1.83
Mpdz	NM_027139.3	multiple PDZ domain protein (Mpdz)	1.61
Mpp6	NM_145968.1	membrane protein, palmitoylated 6 (MAGUK p55 subfamily member 6) (Mpp6)	1.70
Mpv17l	NM_178598.2	Mpv17 transgene, kidney disease mutant-like (Mpv17l)	1.61
Mras	NM_019754.3	muscle and microspikes RAS (Mras)	1.89
Msi2h	NM_198294.2		1.54
Msx1	NM_011529.1	homeobox, msh-like 1 (Msx1)	1.63
Mt2	NM_019636.2	metallothionein 2 (Mt2)	2.91
Mt3	NM_194334.2	metallothionein 3 (Mt3)	3.37
Myadm	NM_009321.2	myeloid-associated differentiation marker (Myadm)	2.08
Mycbpap	NM_173038.3	Mycbp associated protein (Mycbpap)	1.66
Myh8	NM_134011.1	myosin, heavy polypeptide 8, skeletal muscle, perinatal (Myh8)	1.57
Myl9	NM_025355.2	PREDICTED: myosin, light polypeptide 9, regulatory (Myl9)	1.73
Myst4	NM_025703.2	MYST histone acetyltransferase monocytic leukemia 4 (Myst4)	1.51
Myt1	NM_001039474.1	myelin transcription factor 1 (Myt1)	1.55
N4bp2	NM_011544.2	NEDD4 binding protein 2 (N4bp2)	1.72
Nacc2	NM_001037877.2	nucleus accumbens associated 2, BEN and BTB (POZ) domain containing (Nacc2), transcript variant 2	1.61
Nbl1	NM_001079822.1	neuroblastoma, suppression of tumorigenicity 1 (Nbl1)	2.59
Ndrg1	NM_013685.2	N-myc downstream regulated gene 1 (Ndrg1)	1.62
Ndrg2	NM_023755.2		6.75
Ndrl	NM_015749.2		1.66
Nedd4l	NM_013686.3	neural precursor cell expressed, developmentally down-regulated gene 4-like (Nedd4l)	1.72
Nedd9	NM_011565.2	neural precursor cell expressed, developmentally down-regulated gene 9 (Nedd9)	2.23
Nek1	NM_009347.1	PREDICTED: NIMA (never in mitosis gene a)-related expressed kinase 1, transcript variant 4 (Nek1)	1.65
Nek5	NM_011569.2	NIMA (never in mitosis gene a)-related expressed kinase 5 (Nek5)	1.55
Nek9	NM_020584.1	NIMA (never in mitosis gene a)-related expressed kinase 9 (Nek9)	1.56
Nfia	NM_009361.1		2.00
Ngef	NM_019678.2		3.79
Nid2	NM_011638.3	nidogen 2 (Nid2)	3.85
Nisch	NM_009373.3		1.67
Nme5	NM_025920.3	non-metastatic cells 5, protein expressed in (nucleoside-diphosphate kinase) (Nme5)	5.51
Nme7	NM_025790.1	non-metastatic cells 7, protein expressed in (nucleoside-diphosphate kinase) (Nme7), transcript variant 1	1.56
Nope	NM_011568.1	neighbor of Punc E11 (Nope)	2.29
Npal3	NM_025435.2	NIPA-like domain containing 3 (Npal3)	2.61

Nrbp2	XM_126580.1	nuclear receptor binding protein 2 (Nrbp2)	12.98
Nrxn3	NM_145585.1	neurexin III (Nrxn3)	1.68
Nsg2	NM_144543.1	neuron specific gene family member 2 (Nsg2)	2.00
Ntrk2	NM_011589.1	neurotrophic tyrosine kinase, receptor, type 2 (Ntrk2), transcript variant 1	2.33
Ntsr2	NM_013899.1	neurotensin receptor 2 (Ntsr2)	6.79
Nuak1	NM_011590.2	NUAK family, SNF1-like kinase, 1 (Nuak1)	4.56
Nuak2	NM_025616.3	NUAK family, SNF1-like kinase, 2 (Nuak2)	2.17
Nudt10	NM_013898.1	nudix (nucleoside diphosphate linked moiety X)-type motif 10 (Nudt10)	2.22
Nxn	NM_013897.1	nucleoredoxin (Nxn)	2.57
Oat	NM_013896.3	ornithine aminotransferase (Oat)	1.93
Odz4	NM_011593.2	odd Oz/ten-m homolog 4 (Drosophila) (Odz4)	1.51
Ogn	NM_011594.3	osteoglycin (Ogn)	1.50
Ogt	NM_011595.2	O-linked N-acetylglucosamine (GlcNAc) transferase (UDP-N-acetylglucosamine:polypeptide-N-acetylglucosaminyl transferase) (Ogt)	1.64
Olfml1	NM_080639.3	olfactomedin-like 1 (Olfml1)	2.58
Oplah	NM_025372.1	5-oxoprolinase (ATP-hydrolysing) (Oplah)	1.54
Ormdl3	NM_009387.1	ORM1-like 3 (S. cerevisiae) (Ormdl3)	1.71
Osbpl5	NM_026708.1		1.92
Osmr	NM_019725.1	oncostatin M receptor (Osmr)	2.08
Pacrg	NM_053254.2	Park2 co-regulated (Pacrg)	4.38
Pacs2	NM_172664.2	phosphofurin acidic cluster sorting protein 2 (Pacs2)	1.83
Padi2	NM_025360.1	peptidyl arginine deiminase, type II (Padi2)	2.03
Palld	NM_026211.1	PREDICTED: palladin, cytoskeletal associated protein, transcript variant 6 (Palld)	2.04
Paqr7	NM_178638.2	progestin and adipoQ receptor family member VII (Paqr7)	2.03
Pawr	NM_134142.1	PRKC, apoptosis, WT1, regulator (Pawr)	1.89
Pcbp4	NM_173453.2	poly(rC) binding protein 4 (Pcbp4)	1.73
Pcdh10	NM_178789.4	protocadherin 10 (Pcdh10), transcript variant 4	1.56
Pcyt1b	NM_025460.1	phosphate cytidylyltransferase 1, choline, beta isoform (Pcyt1b), transcript variant 2	1.68
Pdgfrl	NM_133804.2	platelet-derived growth factor receptor-like (Pdgfrl)	1.56
Pdlim2	NM_027215.2	PDZ and LIM domain 2 (Pdlim2)	1.75
Pdlim7	NM_025387.1		1.66
Pdrg1	NM_145570.1	p53 and DNA damage regulated 1 (Pdrg1)	1.69
Pdzrn3	NM_025326.2	PDZ domain containing RING finger 3 (Pdzrn3)	3.64
Pea15	NM_023056.3		1.68
Peli2	NM_172608.1		2.72
Pfkfb2	NM_146103.1	6-phosphofructo-2-kinase/fructose-2,6-biphosphatase 2 (Pfkfb2)	1.89
Pfkfb4	NM_199199.2	6-phosphofructo-2-kinase/fructose-2,6-biphosphatase 4 (Pfkfb4)	2.11
Phf19	NM_027865.1	PHD finger protein 19 (Phf19)	1.61
Phkg1	NM_025693.3	phosphorylase kinase gamma 1 (Phkg1)	4.41
Phxr4	NM_028766.1		2.14
Pik3ip1	NM_172614.3	phosphoinositide-3-kinase interacting protein 1 (Pik3ip1)	2.66
Pitpnm2	NM_138751.1	phosphatidylinositol transfer protein, membrane-associated 2 (Pitpnm2)	2.59
Pkmyt1	NM_028355.3	protein kinase, membrane associated tyrosine/threonine 1 (Pkmyt1)	1.85
Pkp4	NM_029478.3	plakophilin 4 (Pkp4), transcript variant 2	1.78
Pla2g7	NM_028264.2	phospholipase A2, group VII (platelet-activating factor acetylhydrolase, plasma) (Pla2g7)	2.58
Plcd1	NM_001033271.3		1.50
Plcd4	NM_178936.3	phospholipase C, delta 4 (Plcd4), transcript variant 1	2.23
Pld2	NM_198167.2	phospholipase D2 (Pld2)	1.53
Plekha4	NM_028097.3	pleckstrin homology domain containing, family A (phosphoinositide binding specific) member 4 (Plekha4)	2.38
Plekhb1	NM_027415.2	pleckstrin homology domain containing, family B (evectins) member 1 (Plekhb1)	2.86
Plp1	NM_025915.3	proteolipid protein (myelin) 1 (Plp1)	2.19
Plscr2	NM_133706.2	phospholipid scramblase 2 (Plscr2)	1.62
Plscr4	NM_146260.2		1.76
Plxdc1	NM_001080134.1	plexin domain containing 1 (Plxdc1)	3.12
Plxnb1	NM_025284.3	plexin B1 (Plxnb1)	1.54
Pnma2	NM_021278.2	paraneoplastic antigen MA2 (Pnma2)	2.50
Podxl	NM_028651.2	podocalyxin-like (Podxl)	1.64
Ppap2a	NM_011607.2	phosphatidic acid phosphatase 2a (Ppap2a), transcript variant 1	1.92
Ppap2b	NM_134131.1	phosphatidic acid phosphatase type 2B (Ppap2b)	2.58
Ppil6	NM_020275.3	PREDICTED: peptidylprolyl isomerase (cyclophilin)-like 6, transcript variant 2 (Ppil6)	1.65
Ppm2c	NM_013749.1	protein phosphatase 2C, magnesium dependent, catalytic subunit (Ppm2c), nuclear gene encoding mitochondrial protein, transcript variant 1	4.00
Ppp1r1b	NM_178589.2	protein phosphatase 1, regulatory (inhibitor) subunit 1B (Ppp1r1b)	2.36
Ppp2r2b	NM_001083587.1	protein phosphatase 2 (formerly 2A), regulatory subunit B (PR 52), beta isoform (Ppp2r2b), transcript variant 1	2.33

Pqlc3	NM_172609.3	PQ loop repeat containing (Pqlc3)	1.51
Prdx6	NM_001037170.2	peroxiredoxin 6 (Prdx6)	1.53
Prelp	NM_138599.4	proline arginine-rich end leucine-rich repeat (Prelp)	3.44
Prf1	NM_011623.1	perforin 1 (pore forming protein) (Prf1)	3.12
Prkag2	NM_011627.3	protein kinase, AMP-activated, gamma 2 non-catalytic subunit (Prkag2)	1.86
Prkcdbp	NM_009415.1	protein kinase C, delta binding protein (Prkcdbp)	1.69
Prkcq	NM_022314.2		1.52
Prnp	NM_001001491.1	prion protein (Prnp)	1.83
Prodh	NM_182839.1		1.63
Prom	NM_026481.2		2.59
Prosapip1	NM_133780.2	ProSAPiP1 protein (Prosapip1)	1.79
Psap	NM_176842.2	prosaposin (Psap)	1.55
Psd2	NM_013837.1	pleckstrin and Sec7 domain containing 2 (Psd2)	8.26
Psg23	NM_028109.3	pregnancy-specific glycoprotein 23 (Psg23)	1.58
Psmd5	AK089281	proteasome (prosome, macropain) 26S subunit, non-ATPase, 5 (Psmd5)	1.53
Ptpre	NM_011632.2	protein tyrosine phosphatase, receptor type, E (Ptpre)	1.99
Punc	NM_009423.3	putative neuronal cell adhesion molecule (Punc)	1.94
Pvalb	NM_172406.2	parvalbumin (Pvalb)	2.94
Pygb	NM_028173.1	brain glycogen phosphorylase (Pygb)	5.69
Rab11b	NM_026508.2	RAB11B, member RAS oncogene family (Rab11b)	2.14
Rab11fip4	NM_001024206.1		2.44
Rab32	NM_021789.2	RAB32, member RAS oncogene family (Rab32)	1.74
Rab36	NM_133977.2		1.54
Rab3d	NM_144551.3	RAB3D, member RAS oncogene family (Rab3d)	1.76
Rab6b	NM_030706.1		1.61
Rabl2a	NM_009054.2	RAB, member of RAS oncogene family-like 2A (Rabl2a)	1.82
Rage	NM_011588.2	renal tumor antigen (Rage)	1.77
Ralgps1	NM_025863.2		2.13
Rapgef3	NM_178110.2	Rap guanine nucleotide exchange factor (GEF) 3 (Rapgef3)	1.91
Rarres1	NM_053100.1	PREDICTED: retinoic acid receptor responder (tazarotene induced) 1 (Rarres1)	1.74
Rassf4		Ras association (RalGDS/AF-6) domain family member 4 (Rassf4)	5.14
Rdh5	NM_133975.4	retinol dehydrogenase 5 (Rdh5)	3.26
Reln	XM_912265.2	reelin (Reln)	5.51
Retsat	NM_011640.2	retinol saturase (all trans retinol 13,14 reductase) (Retsat)	1.85
Rftn2	NM_001025246.1	raftlin family member 2 (Rftn2)	2.34
Rfx2	NM_178111.3	regulatory factor X, 2 (influences HLA class II expression) (Rfx2)	3.81
Rgl2	NM_012035.2	ral guanine nucleotide dissociation stimulator-like 2 (Rgl2)	1.59
Rgma	AK036590	RGM domain family, member A (Rgma)	2.50
Rgs2	NM_001077364.1		1.89
Rgs4	NM_023910.5	regulator of G-protein signaling 4 (Rgs4)	1.58
Rhbdl2	NM_025677.2	rhomboid, veinlet-like 2 (Drosophila) (Rhbdl2)	2.24
Rhob	NM_025537.3	ras homolog gene family, member B (Rhob)	1.70
Rhpn1	NM_021884.3	rhophilin, Rho GTPase binding protein 1 (Rhpn1)	1.57
Rhpn2	NM_173007.3	rhophilin, Rho GTPase binding protein 2 (Rhpn2)	2.31
Rin2	NM_145928.1	Ras and Rab interactor 2 (Rin2)	1.53
Rnase1	NM_197996.2	ribonuclease, RNase A family, 1 (pancreatic) (Rnase1)	3.94
Rnf182	NM_028841.2	ring finger protein 182 (Rnf182)	1.62
Rpgr	NM_019793.2	retinitis pigmentosa GTPase regulator (Rpgr)	1.81
Rps6ka5	NM_019656.3	ribosomal protein S6 kinase, polypeptide 5 (Rps6ka5)	1.59
Rsad1	NM_138631.1	radical S-adenosyl methionine domain containing 1 (Rsad1)	1.53
Rshl3	NM_009437.4	PREDICTED: radial spokehead-like 3 (Rshl3)	3.47
Rsph1	XM_001476544.1	radial spoke head 1 homolog (Chlamydomonas) (Rsph1)	2.48
Rtn1	NM_009441.1	reticulon 1 (Rtn1), transcript variant 1	2.13
S100a1	NM_028235.1	S100 calcium binding protein A1 (S100a1)	1.70
S100a6	XM_127105.8	S100 calcium binding protein A6 (calcyclin) (S100a6)	3.10
Samd9l	NM_198311.1	PREDICTED: sterile alpha motif domain containing 9-like, transcript variant 1 (Samd9l)	1.93
Scd4	NM_027412.3	stearoyl-coenzyme A desaturase 4 (Scd4)	4.59
Scg3	NM_133923.3	secretogranin III (Scg3)	3.71
Scg5	NM_021324.4	secretogranin V (Scg5)	1.57
Scgb1c1	NM_053273.2	secretoglobin, family 1C, member 1 (Scgb1c1)	1.99
scl0002255.1_1	NM_175274.3		2.10
scl0002540.1_6	NM_011653.2		2.29
scl0003799.1_2	XM_147357.1		1.55
Sdc2	NM_146116.1	syndecan 2 (Sdc2)	1.76
Sema4g	NM_011655.4	sema domain, immunoglobulin domain (Ig), transmembrane domain (TM) and short cytoplasmic domain, (semaphorin) 4G (Sema4g)	1.78
Sema6d	NM_026473.2	sema domain, transmembrane domain (TM), and cytoplasmic domain, (semaphorin) 6D (Sema6d), transcript variant 1	2.51
Sept3	NM_019676.1	septin 3 (Sept3)	1.56
Serpina3n	NM_134024.2	serine (or cysteine) peptidase inhibitor, clade A, member 3N (Serpina3n)	2.37

Serpinb6a	NM_172745.2	serine (or cysteine) peptidase inhibitor, clade B, member 6a (Serpinb6a)	2.28
Sesn1	NM_138628.1	sestrin 1 (Sesn1)	2.18
Sft2d2	NM_011660.3	SFT2 domain containing 2 (Sft2d2)	1.53
Sfxn5	NM_025334.3	sideroflexin 5 (Sfxn5)	2.75
Sh3bp5l	NM_001009935.2	SH3 binding domain protein 5 like (Sh3bp5l)	1.57
Shisa4	NM_025299.1	shisa homolog 4 (Xenopus laevis) (Shisa4)	1.54
Shroom3	NM_178604.3	shroom family member 3 (Shroom3), transcript variant 3	1.92
Sidt2	NM_020557.3	SID1 transmembrane family, member 2 (Sidt2)	1.53
Sirt2	NM_021288.3	sirtuin 2 (silent mating type information regulation 2, homolog) 2 (S. cerevisiae) (Sirt2)	2.09
Sirt7	NR_000040.1	sirtuin 7 (silent mating type information regulation 2, homolog) 7 (S. cerevisiae) (Sirt7)	1.53
Slc11a1	NM_024187	solute carrier family 11 (proton-coupled divalent metal ion transporters), member 1 (Slc11a1)	1.67
Slc14a2	NM_133671.1		1.64
Slc15a2	NM_028283.2		2.19
Slc16a9	NM_009457.3	solute carrier family 16 (monocarboxylic acid transporters), member 9 (Slc16a9)	1.82
Slc22a4	NM_016682.2	solute carrier family 22 (organic cation transporter), member 4 (Slc22a4)	2.39
Slc25a18	NM_026872.1	solute carrier family 25 (mitochondrial carrier), member 18 (Slc25a18)	1.54
Slc29a3	NM_026454.3	solute carrier family 29 (nucleoside transporters), member 3 (Slc29a3)	1.58
Slc2a1	NM_025985.4	solute carrier family 2 (facilitated glucose transporter), member 1 (Slc2a1)	1.63
Slc35a2	NM_016786.3	solute carrier family 35 (UDP-galactose transporter), member 2 (Slc35a2)	1.53
Slc38a2	NM_145578.1	solute carrier family 38, member 2 (Slc38a2)	2.94
Slc39a12	NM_027315.3	solute carrier family 39 (zinc transporter), member 12 (Slc39a12)	4.37
Slc44a1	NM_026024.2	solute carrier family 44, member 1 (Slc44a1)	1.61
Slc44a2	NM_133907.3	solute carrier family 44, member 2 (Slc44a2)	2.78
Slc46a3	NM_138589.2	solute carrier family 46, member 3 (Slc46a3)	1.56
Slc6a9	NM_025666.2	solute carrier family 6 (neurotransmitter transporter, glycine), member 9 (Slc6a9)	2.08
Slc8a1	NM_019562.1		1.90
Smoc1	NM_030724.1	SPARC related modular calcium binding 1 (Smoc1)	2.04
Snph	NM_011673	syntaphilin (Snph)	2.46
Snta1	NM_139297.4	syntrophin, acidic 1 (Snta1)	2.05
Sntg2	NM_201641.2	syntrophin, gamma 2 (Sntg2)	2.13
Sobp	NM_145079.2	sine oculis-binding protein homolog (Drosophila) (Sobp)	1.76
Socs2	NM_201410.1	suppressor of cytokine signaling 2 (Socs2)	1.55
Sorbs1	NM_010931.2	sorbin and SH3 domain containing 1 (Sorbs1), transcript variant 5	2.53
Sorl1	NM_144873.2	sortilin-related receptor, LDLR class A repeats-containing (Sorl1)	2.05
Sox21	NM_013881.3	SRY-box containing gene 21 (Sox21)	1.68
Sox9	NM_011677.2		2.63
Spag1	NM_009477.1	sperm associated antigen 1 (Spag1)	1.83
Spag6	NM_025650.2	sperm associated antigen 6 (Spag6)	2.19
Sparcl1	NM_025407.2	SPARC-like 1 (mast9, hevin) (Sparcl1)	1.78
Spata13	NM_025899.2		1.63
Spnb1	NM_025641.3	spectrin beta 1 (Spnb1)	1.57
Spop	NM_009479.2	speckle-type POZ protein (Spop)	2.15
Srgap3	NM_146144.2	SLIT-ROBO Rho GTPase activating protein 3 (Srgap3)	3.17
Srl	NM_138592.3	sarcalumenin (Srl)	2.44
Srpk3	NM_133857.3	serine/arginine-rich protein specific kinase 3 (Srpk3)	1.56
Srr	NM_030180.2		1.82
Sstr2	NM_001013378.1		1.97
St5	NM_026031.3	suppression of tumorigenicity 5 (St5), transcript variant 2	1.97
St8sia2	NM_013840.2	ST8 alpha-N-acetyl-neuraminide alpha-2,8-sialyltransferase 2 (St8sia2)	2.30
Stard13	NM_009498.3	StAR-related lipid transfer (START) domain containing 13 (Stard13)	1.54
Stard8	NM_016796.2	START domain containing 8 (Stard8)	3.62
Stim1	NM_013933.2	stromal interaction molecule 1 (Stim1)	1.51
Stk36	NM_019806.5	serine/threonine kinase 36 (fused homolog, Drosophila) (Stk36)	2.17
Sulf1	NM_011690.2	sulfatase 1 (Sulf1)	2.11
Syap1	NM_139307.2	synapse associated protein 1 (Syap1)	2.68
Syn2	NM_173016.3	synapsin II (Syn2)	1.88
Synm	NM_020505.2	synemin, intermediate filament protein (Synm), transcript variant 3	1.69
Tagln3	NM_011692.2	transgelin 3 (Tagln3)	2.34
Tbc1d2b	NM_011694.3	TBC1 domain family, member 2B (Tbc1d2b)	1.64
Tbcel	NM_011695.2	tubulin folding cofactor E-like (Tbcel)	2.25
Tceal6	NM_011696.1	transcription elongation factor A (SII)-like 6 (Tceal6)	1.75
Tcf25	NM_009505.3	transcription factor 25 (basic helix-loop-helix) (Tcf25), transcript variant 1	1.54
Tcf3	NM_011697.2	transcription factor 3 (Tcf3), transcript variant 1	1.56
Tcfcp2l1	NM_009506.2	transcription factor CP2-like 1 (Tcfcp2l1)	1.82
Tecta	NM_009507.3	tectorin alpha (Tecta)	1.85
Tekt1	NM_178600.2	tektin 1 (Tekt1)	1.81
Terf2ip	NM_027121.3	telomeric repeat binding factor 2, interacting protein (Terf2ip)	1.73
Tgm2	NM_013703.1	transglutaminase 2, C polypeptide (Tgm2)	1.56

Thra	NM_019780.1		1.57
Timp2	NM_027338.1	tissue inhibitor of metalloproteinase 2 (Timp2)	2.91
Timp3	NM_177876.4	tissue inhibitor of metalloproteinase 3 (Timp3)	1.59
Tmem108	NM_139061.3	transmembrane protein 108 (Tmem108)	1.82
Tmem117	AK039554	transmembrane protein 117 (Tmem117)	1.54
Tmem166	NM_172767.2	transmembrane protein 166 (Tmem166)	1.52
Tmem25	NM_153423	transmembrane protein 25 (Tmem25)	1.83
Tmem43	NM_021714.3	transmembrane protein 43 (Tmem43)	1.58
Tmem47	NM_145218.3	transmembrane protein 47 (Tmem47)	4.09
Tmie	NM_027963.2	transmembrane inner ear (Tmie)	2.85
Tmtc4	XM_917905.3	transmembrane and tetratricopeptide repeat containing 4 (Tmtc4)	1.55
Tns3	NM_027740.3	tensin 3 (Tns3)	1.66
Tpbg	NM_146039.3	trophoblast glycoprotein (Tpbg)	1.81
Tppp	NM_027725.2	tubulin polymerization promoting protein (Tppp)	1.57
Tppp3	NM_134139.1	tubulin polymerization-promoting protein family member 3 (Tppp3)	2.00
Tprkb	NM_028599.1	Tp53rk binding protein (Tprkb)	2.73
Traf1	NM_030234.2		4.89
Trim2	NM_146254.2	tripartite motif protein 2 (Trim2)	1.57
Trpc7	NM_029896.1	transient receptor potential cation channel, subfamily C, member 7 (Trpc7)	1.92
Trps1	XM_001479426.1		2.04
Tsc22d3	NM_198606.1	TSC22 domain family, member 3 (Tsc22d3), transcript variant 1	1.58
Tsc22d4	XM_132006	TSC22 domain family, member 4 (Tsc22d4)	2.00
Tspan14	NM_145940.2	tetraspanin 14 (Tspan14)	1.76
Tspan15	NM_001042565.2	tetraspanin 15 (Tspan15)	1.89
Tspan17	NM_021539.4	tetraspanin 17 (Tspan17)	2.89
Tspan3	NM_177618.4	tetraspanin 3 (Tspan3)	1.64
Tspan6	NM_170779.1	tetraspanin 6 (Tspan6)	1.65
Tst	NM_177327.3	thiosulfate sulfurtransferase, mitochondrial (Tst), nuclear gene encoding mitochondrial protein	2.23
Ttc28	NM_133784.2	PREDICTED: tetratricopeptide repeat domain 28 (Ttc28)	1.69
Ttc30b	NM_013753.1	tetratricopeptide repeat domain 30B (Ttc30b)	2.97
Ttc8	NM_013842.2	tetratricopeptide repeat domain 8 (Ttc8), transcript variant 2	1.55
Ttll3	NM_133216.2	tubulin tyrosine ligase-like family, member 3 (Ttll3)	2.79
Ttyh1	NM_028816.2	tweety homolog 1 (Drosophila) (Ttyh1), transcript variant 2	6.19
Ttyh2	NM_010247.1	tweety homolog 2 (Drosophila) (Ttyh2)	2.91
Tuba1a	NM_001033237.1	tubulin, alpha 1A (Tuba1a)	1.52
Tubb2c	NM_026570.1	tubulin, beta 2c (Tubb2c)	1.70
Txlnb	NM_025347.1	taxilin beta (Txlnb)	1.61
Txnip	NM_018753.5	thioredoxin interacting protein (Txnip), transcript variant 1	2.66
Tyki	NM_018871.2		1.86
Ugp2	NM_011738.1	UDP-glucose pyrophosphorylase 2 (Ugp2)	2.01
Ugt1a10	NM_011739.2	UDP glycosyltransferase 1 family, polypeptide A10 (Ugt1a10)	5.07
Ugt1a6a	NM_145356.3	UDP glucuronosyltransferase 1 family, polypeptide A6A (Ugt1a6a)	1.75
Ugt1a6b	NM_025970.1	UDP glucuronosyltransferase 1 family, polypeptide A6B (Ugt1a6b)	1.71
Ulk2	NM_172735.2	Unc-51 like kinase 2 (C. elegans) (Ulk2)	1.55
Uros	NM_025893.2	uroporphyrinogen III synthase (Uros)	1.51
Usp53	NM_026025.1	ubiquitin specific peptidase 53 (Usp53)	1.76
Usp54	NM_026647.2	ubiquitin specific peptidase 54 (Usp54)	1.58
Vamp4	NM_025883.3	vesicle-associated membrane protein 4 (Vamp4)	1.58
Vasn	NM_011546.2	vasorin (Vasn)	1.76
Vat1l	NM_178694.3	vesicle amine transport protein 1 homolog-like (T. californica) (Vat1l)	1.71
Vhl	NM_011743.2	von Hippel-Lindau syndrome (Vhl)	1.53
Vps37b	NM_028245.1	vacuolar protein sorting 37B (yeast) (Vps37b)	1.76
Vwa5a	NM_011749.4	von Willebrand factor A domain containing 5A (Vwa5a)	1.65
Wdr16	NM_172483.1	WD repeat domain 16 (Wdr16)	1.56
Wdr60	NM_011751.2	WD repeat domain 60 (Wdr60)	1.54
Wdr69	NM_172575.2	WD repeat domain 69 (Wdr69)	3.44
Wdr78	AK034574	WD repeat domain 78 (Wdr78)	2.93
Wipi1	NM_145600.1	WD repeat domain, phosphoinositide interacting 1 (Wipi1)	1.68
Wsb1	NM_011756.4	WD repeat and SOCS box-containing 1 (Wsb1), transcript variant 2	1.70
Wwc1	NM_178679.2	WW, C2 and coiled-coil domain containing 1 (Wwc1)	2.78
X99384	NM_007564.2	cDNA sequence X99384 (X99384)	2.46
Yeats2	NM_001081144.1	YEATS domain containing 2 (Yeats2)	1.62
Ypel3	NM_175751.3	yippee-like 3 (Drosophila) (Ypel3)	2.65
Zcchc18	NM_175433.4	zinc finger, CCHC domain containing 18 (Zcchc18), transcript variant 3	3.26
Zer1	NM_175466.2	zer-1 homolog (C. elegans) (Zer1)	1.51
Zfp277	NM_001039718.1	zinc finger protein 277 (Zfp277), transcript variant 1	1.50
Zfp288	NM_009572.3		2.57
Zfp36	NM_023322.2	zinc finger protein 36 (Zfp36)	1.50
Zfp36l1	NM_023162.4	zinc finger protein 36, C3H type-like 1 (Zfp36l1)	1.76
Zfp608	NM_199143.1	zinc finger protein 608 (Zfp608)	1.69

Zfp91-cntf	XM_358311	zinc finger protein 91, ciliary neurotrophic factor transcription unit (Zfp91-cntf)	8.90
Zhx1	NM_025635.1	zinc fingers and homeoboxes 1 (Zhx1), transcript variant 1	1.84
Zkscan14	NR_003292.1	zinc finger with KRAB and SCAN domains 14 (Zkscan14)	1.57
Zxda	NM_011777.2	zinc finger, X-linked, duplicated A (Zxda), non-coding RNA.	2.63
