# Supplementary material for: miR-30 Family miRNAs Mediate the Effect of Chronic Social Defeat Stress on Hippocampal Neurogenesis in Mouse Depression Model
Source: Front Mol Neurosci. 2019 Aug 8;12:188. doi: 10.3389/fnmol.2019.00188 (PMC6694739; doi:10.3389/fnmol.2019.00188)
Supplement: TABLE S8 — The list comprises of genes which demonstrated decreased expression (fold change ≥1.5 and p ≤ 0.05) in the differentiated cells from an early phase (day 2) when compared with the proliferating neurospheres. [file Table_8.pdf]

Table S8. List of genes, which demonstrated decreased expression (fold change ≥ 1.5 and p ≤ 0.05) in the differentiated cells from an early phase (day 2) when compared with the proliferating neurospheres

Gene Symbol	Accession No.	Gene Definition	Fold Change
0610007J10Rik	NM_017380.1		0.40
0610007P14Rik	NM_001009818.1	RIKEN cDNA 0610007P14 gene (0610007P14Rik)	0.63
0610010E21Rik	AK018717	RIKEN cDNA 0610010E21 gene (0610010E21Rik)	0.59
0610010F05Rik	NM_021446.2		0.57
0610012G03Rik	NM_001033140.3		0.56
1100001F19Rik	NM_027860		0.55
1110001A07Rik	NM_025320	RIKEN cDNA 1110001A07 gene (1110001A07Rik)	0.56
1110001C20Rik	NM_133186		0.49
1110001J03Rik	XM_124187.1	RIKEN cDNA 1110001J03 gene (1110001J03Rik)	0.50
1110001N06Rik	NM_025377.1		0.62
1110002E23Rik	NM_177730		0.57
1110003F05Rik	NM_025363.2		0.54
1110007L15Rik	AK003256	RIKEN cDNA 1110007L15 gene (1110007L15Rik)	0.55
1110007M04Rik	AK003291	RIKEN cDNA 1110007M04 gene (1110007M04Rik)	0.33
1110008B24Rik			0.62
1110008F13Rik	NM_026269.1	RIKEN cDNA 1110008F13 gene (1110008F13Rik)	0.31
1110008P14Rik	NM_026742.3	RIKEN cDNA 1110008P14 gene (1110008P14Rik)	0.38
1110012D08Rik	XM_177016.2	RIKEN cDNA 1110012D08 gene (1110012D08Rik)	0.47
1110018J23Rik	NM_026124.2		0.52
1110019N10Rik	NM_198001.1	RIKEN cDNA 1110019N10 gene (1110019N10Rik)	0.52
1110020P15Rik	NM_178066.2	RIKEN cDNA 1110020P15 gene (1110020P15Rik)	0.61
1110021J02Rik	NM_172963.3		0.57
1110021L09Rik	NM_026754.2	PREDICTED: RIKEN cDNA 1110021L09 gene (1110021L09Rik)	0.64
1110038B12Rik	XM_140742.4	PREDICTED: RIKEN cDNA 1110038B12 gene, transcript variant 6 (1110038B12Rik)	0.53
1110059E24Rik	NM_026753.1	RIKEN cDNA 1110059E24 gene (1110059E24Rik)	0.66
1110067D22Rik	NM_197979.2	RIKEN cDNA 1110067D22 gene (1110067D22Rik)	0.47
1190002F15Rik			0.53
1190009E20Rik	XM_001474669.1		0.61
1200003C05Rik		RIKEN cDNA 1200003C05 gene (1200003C05Rik)	0.50
1500001L15Rik	XM_922038.2		0.67
1500001M20Rik	NM_025423.1	RIKEN cDNA 1500001M20 gene (1500001M20Rik)	0.58
1500012F01Rik	NM_173752.3	RIKEN cDNA 1500012F01 gene (1500012F01Rik)	0.64
1500032D16Rik		RIKEN cDNA 1500032D16 gene (1500032D16Rik), transcript variant 2	0.53
1500034J01Rik	XM_147036	PREDICTED: RIKEN cDNA 1500034J01 gene (1500034J01Rik)	0.61
1600012H06Rik	NM_197988.1	RIKEN cDNA 1600012H06 gene (1600012H06Rik), transcript variant 4	0.62
1700011E24Rik	NM_001033144.1	PREDICTED: RIKEN cDNA 1700011E24 gene, transcript variant 2 (1700011E24Rik)	0.22
1700013A01Rik			0.59
1700021F05Rik	NM_024205.1	RIKEN cDNA 1700021F05 gene (1700021F05Rik)	0.62
1700025G04Rik	XM_001002205.2	RIKEN cDNA 1700025G04 gene (1700025G04Rik)	0.41
1700027M01Rik	XM_127738.3		0.64
1700029F09Rik	NM_026894.1	PREDICTED: RIKEN cDNA 1700029F09 gene, transcript variant 20 (1700029F09Rik)	0.39
1700037H04Rik	NM_001081005.1	RIKEN cDNA 1700037H04 gene (1700037H04Rik)	0.56
1700041B20Rik	NM_024283.2		0.49
1700086L19Rik	NM_025892.1	PREDICTED: RIKEN cDNA 1700086L19 gene (1700086L19Rik)	0.56
1700113I22Rik	NM_001083891.1	RIKEN cDNA 1700113I22 gene (1700113I22Rik)	0.50
1810006K21Rik	XM_912953.3	PREDICTED: RIKEN cDNA 1810006K21 gene (1810006K21Rik)	0.50
1810009A15Rik	AK028065	RIKEN cDNA 1810009A15 gene (1810009A15Rik)	0.65
1810022K09Rik	NM_001083882.1		0.52
1810027O10Rik	XM_001478332.1		0.56
1810035L17Rik	NM_027040.1	RIKEN cDNA 1810035L17 gene (1810035L17Rik)	0.59
1810055G02Rik	NM_001081275.1	RIKEN cDNA 1810055G02 gene (1810055G02Rik)	0.46
1810063B05Rik	XM_128789.5	RIKEN cDNA 1810063B05 gene (1810063B05Rik)	0.58
2010007H12Rik	AK005934	RIKEN cDNA 2010007H12 gene (2010007H12Rik)	0.67
2010107E04Rik	XM_994504.1	RIKEN cDNA 2010107E04 gene (2010107E04Rik)	0.55
2010309L07Rik	NM_029331.2		0.60
2010317E24Rik	NM_026411.1		0.51
2010321I05Rik	NM_197990.2		0.65
2010321M09Rik	NM_183254.1	RIKEN cDNA 2010321M09 gene (2010321M09Rik), transcript variant 1	0.62
2210411K11Rik	NM_023544	RIKEN cDNA 2210411K11 gene (2210411K11Rik)	0.66
2210412D01Rik	NM_029338.3	RIKEN cDNA 2210412D01 gene (2210412D01Rik)	0.64
2310003F16Rik	XM_919077.2	RIKEN cDNA 2310003F16 gene (2310003F16Rik)	0.65
2310003L22Rik	NM_001033148.2	RIKEN cDNA 2310003L22 gene (2310003L22Rik)	0.57
2310005E10Rik	NM_026091.2	RIKEN cDNA 2310005E10 gene (2310005E10Rik)	0.60
2310005N03Rik	XM_485065	RIKEN cDNA 2310005N03 gene (2310005N03Rik)	0.49

2310008M10Rik	NM_001033185.1	RIKEN cDNA 2310008M10 gene (2310008M10Rik)	0.58
2310009B15Rik	XM_358420.3	RIKEN cDNA 2310009B15 gene (2310009B15Rik)	0.57
2310014H01Rik	NM_138581.2	PREDICTED: RIKEN cDNA 2310014H01 gene, transcript variant 3 (2310014H01Rik)	0.32
2310016C08Rik	NM_026865.2	RIKEN cDNA 2310016C08 gene (2310016C08Rik)	0.41
2310016M24Rik	XM_983803.1	RIKEN cDNA 2310016M24 gene (2310016M24Rik)	0.57
2310036D04Rik	NM_172701.2		0.53
2310037I24Rik	NM_025463.2	RIKEN cDNA 2310037I24 gene (2310037I24Rik)	0.66
2310039H08Rik	NM_029631	RIKEN cDNA 2310039H08 gene (2310039H08Rik)	0.59
2310044H10Rik	XM_485210		0.52
2310045K21Rik	XM_109683.4		0.58
2310051F07Rik			0.61
2310061F22Rik	NM_026958.2	RIKEN cDNA 2310061F22 gene (2310061F22Rik)	0.66
2310079P03Rik	NM_028077.2		0.62
2400010C15Rik	NM_174987.3		0.52
2410003K15Rik	NM_027242.3		0.39
2410015M20Rik	NM_027360.2	RIKEN cDNA 2410015M20 gene (2410015M20Rik)	0.63
2410015N17Rik		RIKEN cDNA 2410015N17 gene (2410015N17Rik)	0.30
2410016F01Rik	XM_130053.2		0.67
2410016O06Rik			0.65
2410022L05Rik	NM_175153.3	RIKEN cDNA 2410022L05 gene (2410022L05Rik)	0.64
2510009E07Rik		RIKEN cDNA 2510009E07 gene (2510009E07Rik)	0.63
2600005C20Rik	NM_029384.1		0.48
2610002D18Rik	NM_133722.1		0.64
2610017I09Rik	XM_901486.2	PREDICTED: RIKEN cDNA 2610017I09 gene (2610017I09Rik), misc RNA.	0.47
2610019E17Rik	NM_026318.2		0.45
2610024B07Rik	NM_027093.3		0.50
2610024G14Rik	NM_172398.3	RIKEN cDNA 2610024G14 gene (2610024G14Rik)	0.60
2610024H22Rik	NM_025511.2		0.62
2610029G23Rik	NM_025506.2	RIKEN cDNA 2610029G23 gene (2610029G23Rik)	0.56
2610036L11Rik	NM_025857.2	PREDICTED: RIKEN cDNA 2610036L11 gene, transcript variant 1 (2610036L11Rik)	0.26
2610039C10Rik	NM_025509.2	RIKEN cDNA 2610039C10 gene (2610039C10Rik)	0.40
2610101N10Rik	NM_001081226.1	RIKEN cDNA 2610101N10 gene (2610101N10Rik)	0.65
2610103J23Rik	NM_001082975.1		0.61
2610207P08Rik	XM_001003622.2		0.59
2610307O08Rik	NM_023516.3	PREDICTED: RIKEN cDNA 2610307O08 gene, transcript variant 12 (2610307O08Rik)	0.29
2610510J17Rik	NM_183256.2	RIKEN cDNA 2610510J17 gene (2610510J17Rik)	0.39
2700029M09Rik	NM_175149.3	PREDICTED: RIKEN cDNA 2700029M09 gene (2700029M09Rik)	0.46
2700055A20Rik	XM_488563		0.62
2700092H06Rik	NM_133714.2		0.56
2700094K13Rik	NM_025966.3	RIKEN cDNA 2700094K13 gene (2700094K13Rik), transcript variant 2	0.17
2810025M15Rik	XM_912979.2	PREDICTED: RIKEN cDNA 2810025M15 gene (2810025M15Rik), misc RNA.	0.34
2810405J04Rik		RIKEN cDNA 2810405J04 gene (2810405J04Rik)	0.53
2810407C02Rik	NM_197991	RIKEN cDNA 2810407C02 gene (2810407C02Rik)	0.62
2810410M20Rik	AK009823	RIKEN cDNA 2810410M20 gene (2810410M20Rik)	0.61
2810417H13Rik		RIKEN cDNA 2810417H13 gene (2810417H13Rik)	0.16
2810417K24Rik	NM_153775.1		0.42
2810423A18Rik	AK010232		0.49
2810428I15Rik			0.65
2900010M23Rik	AK010307	RIKEN cDNA 2900010M23 gene (2900010M23Rik)	0.58
2900060B14Rik	XM_132537.3		0.61
2900060P06Rik	NM_153152.3		0.52
3000004C01Rik	NM_023203.1	RIKEN cDNA 3000004C01 gene (3000004C01Rik)	0.41
3110001D03Rik	NM_022423.1	RIKEN cDNA 3110001D03 gene (3110001D03Rik)	0.62
3110009E18Rik	NM_023633	RIKEN cDNA 3110009E18 gene (3110009E18Rik)	0.52
3110013H01Rik	NM_025556.2		0.45
3110043A19Rik	NM_028596.2	PREDICTED: RIKEN cDNA 3110043A19 gene (3110043A19Rik)	0.57
3300001G02Rik	NM_001001881.1	RIKEN cDNA 3300001G02 gene (3300001G02Rik)	0.50
3632451O06Rik	NM_028244	RIKEN cDNA 3632451O06 gene (3632451O06Rik)	0.41
3830612M24	XM_131720.2		0.18
3930401E15Rik	XR_035175.1		0.64
4631422C13Rik		PREDICTED: RIKEN cDNA 4631422C13 gene (4631422C13Rik)	0.64
4632417K18Rik		RIKEN cDNA 4632417K18 gene (4632417K18Rik)	0.30
4833424O15Rik	NM_019836.2	RIKEN cDNA 4833424O15 gene (4833424O15Rik)	0.46
4833426J09Rik			0.65
4833427B12Rik	NM_026312.2		0.58
4930427A07Rik	XM_902349.2	RIKEN cDNA 4930427A07 gene (4930427A07Rik)	0.65
4930461P20Rik	XM_488549.3	PREDICTED: RIKEN cDNA 4930461P20 gene (4930461P20Rik)	0.63
4930547N16Rik	NM_025642.1	RIKEN cDNA 4930547N16 gene (4930547N16Rik)	0.55

4930572J05Rik	NM_026476.1	RIKEN cDNA 4930572J05 gene (4930572J05Rik)	0.58
4932417H02Rik	NM_026002	RIKEN cDNA 4932417H02 gene (4932417H02Rik)	0.62
4933401P20Rik	NM_027426.2		0.64
4933407C03Rik	XM_127746.2	PREDICTED: RIKEN cDNA 4933407C03 gene, transcript variant 6 (4933407C03Rik)	0.65
4933407P14Rik	XM_921606.2		0.62
4933427G23Rik	NM_028131.1	PREDICTED: RIKEN cDNA 4933427G23 gene (4933427G23Rik), misc RNA.	0.52
5430417J04Rik	NM_028258.1		0.63
5530401N12Rik	XM_001474993.1		0.38
5730410I19Rik	XR_035347.1	RIKEN cDNA 5730410I19 gene (5730410I19Rik)	0.59
5730427N09Rik	NM_027314	RIKEN cDNA 5730427N09 gene (5730427N09Rik)	0.57
5730449L18Rik	NM_026528.3	RIKEN cDNA 5730449L18 gene (5730449L18Rik)	0.65
5730494J16Rik	XM_132529.2		0.36
5730525O22Rik	NM_001037279.1		0.60
5730528L13Rik	NM_145144.1	RIKEN cDNA 5730528L13 gene (5730528L13Rik)	0.65
5830411I20	XR_035184.1		0.40
5930416I19Rik		RIKEN cDNA 5930416I19 gene (5930416I19Rik)	0.48
6030405A18	NM_133747.2		0.59
6230416J20Rik	NM_001040396.2		0.66
6330549D23Rik	NM_024428.2	RIKEN cDNA 6330549D23 gene (6330549D23Rik), non-coding RNA.	0.51
6330578E17Rik	NM_026515.2	RIKEN cDNA 6330578E17 gene (6330578E17Rik)	0.56
6332401O19Rik	AK013109	RIKEN cDNA 6332401O19 gene (6332401O19Rik)	0.64
6430402L23Rik			0.41
6430527G18Rik	XM_147329.1	RIKEN cDNA 6430527G18 gene (6430527G18Rik)	0.37
6430537F04			0.50
6430706D22Rik	XM_484890	RIKEN cDNA 6430706D22 gene (6430706D22Rik)	0.48
6530418L21Rik	NM_026063.1	RIKEN cDNA 6530418L21 gene (6530418L21Rik)	0.44
6720458F09Rik	NM_144518.3	RIKEN cDNA 6720458F09 gene (6720458F09Rik)	0.48
6720463M24Rik	NM_172884	RIKEN cDNA 6720463M24 gene (6720463M24Rik)	0.52
6720469N11Rik			0.52
8030402P03Rik	NM_183095.1		0.62
8430410K20Rik		RIKEN cDNA 8430410K20 gene (8430410K20Rik)	0.65
9430029K10Rik	NM_197959.1		0.65
9430065L19Rik	AK076147		0.45
9430080K19Rik	XM_001002726.1		0.48
9530018I07Rik	NM_025849.2		0.54
9530058B02Rik	NM_001033167.1	RIKEN cDNA 9530058B02 gene (9530058B02Rik)	0.58
9530064J02	NM_028439.1		0.15
9930014A18Rik	XM_203393.1		0.47
A230046K03Rik		RIKEN cDNA A230046K03 gene (A230046K03Rik)	0.66
A230079E18Rik	AK014074		0.46
A430005L14Rik	XM_001476479.1	RIKEN cDNA A430005L14 gene (A430005L14Rik)	0.64
A830059I20Rik	NM_026085.2		0.33
A930030J18Rik	NM_030093.1		0.52
AA960436		expressed sequence AA960436 (AA960436)	0.60
Aaas	NM_026142.1	achalasia, adrenocortical insufficiency, alacrimia (Aaas)	0.63
Aacs	NM_178141.2	acetoacetyl-CoA synthetase (Aacs)	0.55
Aadacl1		arylacetamide deacetylase-like 1 (Aadacl1)	0.45
Aard	AK076210	alanine and arginine rich domain containing protein (Aard)	0.53
Aars	NM_026486.1	alanyl-tRNA synthetase (Aars)	0.46
Aarsd1	NM_001081295.1	alanyl-tRNA synthetase domain containing 1 (Aarsd1)	0.66
Aatk	XM_984472.1	apoptosis-associated tyrosine kinase (Aatk)	0.22
Abcb6	NM_026640.2	ATP-binding cassette, sub-family B (MDR/TAP), member 6 (Abcb6), nuclear gene encoding mitochondrial protein	0.56
Abcb7	AK029315	PREDICTED: ATP-binding cassette, sub-family B (MDR/TAP), member 7, transcript variant 5 (Abcb7)	0.51
Abcf1		ATP-binding cassette, sub-family F (GCN20), member 1 (Abcf1)	0.63
Abcf2	NM_027845.1	ATP-binding cassette, sub-family F (GCN20), member 2 (Abcf2), nuclear gene encoding mitochondrial protein	0.60
Abi2	NM_026127.3	abl-interactor 2 (Abi2)	0.57
Acap3	NM_029425.1	ArfGAP with coiled-coil, ankyrin repeat and PH domains 3 (Acap3)	0.36
Acat2		acetyl-Coenzyme A acetyltransferase 2 (Acat2)	0.40
Acbd3	XM_195728	acyl-Coenzyme A binding domain containing 3 (Acbd3)	0.65
Acly	NM_133923.2	ATP citrate lyase (Acly)	0.43
Acn9	AK015050		0.65
Aco2	NM_134041.2	aconitase 2, mitochondrial (Aco2), nuclear gene encoding mitochondrial protein	0.56
Acot7	NM_027507.1	acyl-CoA thioesterase 7 (Acot7)	0.42
Acpl2	NM_029115.1	acid phosphatase-like 2 (Acpl2)	0.57
Acsbg1	XM_001473469.1	acyl-CoA synthetase bubblegum family member 1 (Acsbg1)	0.40
Acsl5	XM_355576.1	acyl-CoA synthetase long-chain family member 5 (Acsl5)	0.36

Acss2	NM_029249.2	acyl-CoA synthetase short-chain family member 2 (Acss2)	0.55
Actb	NM_026353.2	actin, beta (Actb)	0.52
Actl6a	NM_198607.1	actin-like 6A (Actl6a)	0.51
Actr10	XM_357260.1	ARP10 actin-related protein 10 homolog (S. cerevisiae) (Actr10)	0.57
Actr3	NM_028898.2	ARP3 actin-related protein 3 homolog (yeast) (Actr3)	0.58
Adam12	NM_001081025.1	a disintegrin and metallopeptidase domain 12 (meltrin alpha) (Adam12)	0.21
Adam15	XM_620624.3	a disintegrin and metallopeptidase domain 15 (metargidin) (Adam15), transcript variant 2	0.64
Adam17	AK016608	a disintegrin and metallopeptidase domain 17 (Adam17)	0.58
Adamts19	XM_001004724.2	a disintegrin-like and metallopeptidase (reprolysin type) with thrombospondin type 1 motif, 19 (Adamts19)	0.43
Adamts4	XM_129929.1		0.36
Adap1	NM_199019.1	ArfGAP with dual PH domains 1 (Adap1)	0.55
Adm	NM_027707	adrenomedullin (Adm)	0.43
Adpgk	XR_005167.1		0.60
Adprh	NM_027728.2	ADP-ribosylarginine hydrolase (Adprh)	0.57
Adrb2	NM_001004146.1	adrenergic receptor, beta 2 (Adrb2)	0.53
Adrbk1	XM_149592.1	adrenergic receptor kinase, beta 1 (Adrbk1)	0.61
Adrm1	XM_128281	adhesion regulating molecule 1 (Adrm1)	0.62
Adsl	NM_001003948.1		0.63
Aes	AK030367		0.63
Afg3l1	NM_026606.1	AFG3(ATPase family gene 3)-like 1 (yeast) (Afg3l1)	0.64
Agap1	AK077378	ArfGAP with GTPase domain, ankyrin repeat and PH domain 1 (Agap1), transcript variant 2	0.53
Agfg1	AK030671	ArfGAP with FG repeats 1 (Agfg1)	0.60
Agfg2		ArfGAP with FG repeats 2 (Agfg2), transcript variant 2	0.53
Agpat6	NM_145509.1	1-acylglycerol-3-phosphate O-acyltransferase 6 (lysophosphatidic acid acyltransferase, zeta) (Agpat6)	0.64
Ahctf1		AT hook containing transcription factor 1 (Ahctf1)	0.56
Ahcy	NM_027442		0.50
Ahcyl1	NM_178765.3	S-adenosylhomocysteine hydrolase-like 1 (Ahcyl1)	0.66
AI413582	NM_025666.1	expressed sequence AI413582 (AI413582)	0.56
AI448196	NM_021552.4		0.50
AI481316	NM_025677.1		0.60
AI593442	AK077637	expressed sequence AI593442 (AI593442), transcript variant 1	0.50
AI851790	AK017789	expressed sequence AI851790 (AI851790)	0.55
Aip	NM_028137.2	aryl-hydrocarbon receptor-interacting protein (Aip)	0.63
Ak2		adenylate kinase 2 (Ak2), transcript variant 2	0.55
Akap12	NM_197981.1	A kinase (PRKA) anchor protein (gravin) 12 (Akap12)	0.11
Akap7	NM_177854	A kinase (PRKA) anchor protein 7 (Akap7)	0.47
Akirin1	AK031346	akirin 1 (Akirin1)	0.65
Akr1a4		aldo-keto reductase family 1, member A4 (aldehyde reductase) (Akr1a4)	0.63
AL022832	NM_173400		0.63
Alad	NM_134022.2	aminolevulinate, delta-, dehydratase (Alad)	0.58
Alas1	XM_358637.1	aminolevulinic acid synthase 1 (Alas1)	0.51
Aldh18a1		aldehyde dehydrogenase 18 family, member A1 (Aldh18a1), nuclear gene encoding mitochondrial protein, transcript variant 1	0.48
Aldoa	NM_001033301.2	aldolase A, fructose-bisphosphate (Aldoa)	0.63
Alg8	NR_003619.1	asparagine-linked glycosylation 8 homolog (yeast, alpha-1,3-glucosyltransferase) (Alg8)	0.56
Alg9	NM_198006.3	asparagine-linked glycosylation 9 homolog (yeast, alpha 1,2 mannosyltransferase) (Alg9)	0.62
Amotl1	NM_177013.3	angiomotin-like 1 (Amotl1)	0.31
Amph	AK078178	amphiphysin (Amph)	0.66
Anapc5	NM_145836.1	anaphase-promoting complex subunit 5 (Anapc5), transcript variant 1	0.66
Angpt2		angiopoietin 2 (Angpt2)	0.05
Ank3	NM_178689.3	ankyrin 3, epithelial (Ank3), transcript variant 6	0.23
Anln	NM_172286	anillin, actin binding protein (Anln)	0.21
Anp32e	NM_172930.2	acidic (leucine-rich) nuclear phosphoprotein 32 family, member E (Anp32e)	0.34
Anxa3	NM_198652.1	annexin A3 (Anxa3)	0.36
Ap2b1	NM_175398.3	adaptor-related protein complex 2, beta 1 subunit (Ap2b1), transcript variant 1	0.54
Ap4s1	NM_177374.2	adaptor-related protein complex AP-4, sigma 1 (Ap4s1)	0.67
Apaf1	NM_175265.4	apoptotic peptidase activating factor 1 (Apaf1), transcript variant 1	0.63
Apex1	NM_177138.2	apurinic/apyrimidinic endonuclease 1 (Apex1)	0.66
Apitd1		apoptosis-inducing, TAF9-like domain 1 (Apitd1)	0.66
Aplp2		amyloid beta (A4) precursor-like protein 2 (Aplp2), transcript variant 3	0.64
Appbp2	AK078743		0.57
Aprin	AK078769	androgen-induced proliferation inhibitor (Aprin)	0.62
Aprt	NM_145609.1		0.58
Arc	AK082376	activity regulated cytoskeletal-associated protein (Arc)	0.58

Ard1	AK020369		0.57
Ard1a	AK034130	ARD1 homolog A, N-acetyltransferase (S. cerevisiae) (Ard1a)	0.42
Areg	XM_128267	amphiregulin (Areg)	0.27
Arf6	NM_177462	ADP-ribosylation factor 6 (Arf6)	0.53
Arhgap18	XM_284281.2	Rho GTPase activating protein 18 (Arhgap18)	0.27
Arl2bp	AK034952	ADP-ribosylation factor-like 2 binding protein (Arl2bp), transcript variant 1	0.65
Arl4c		ADP-ribosylation factor-like 4C (Arl4c)	0.57
Arl6ip1		ADP-ribosylation factor-like 6 interacting protein 1 (Arl6ip1)	0.63
Armcx2	NM_026633.1	armadillo repeat containing, X-linked 2 (Armcx2)	0.55
Arpc1b	XM_489200	actin related protein 2/3 complex, subunit 1B (Arpc1b)	0.30
Arpc2	AK035833	actin related protein 2/3 complex, subunit 2 (Arpc2)	0.67
Arpc3	AK036567	actin related protein 2/3 complex, subunit 3 (Arpc3)	0.58
Asah1	AK036626	N-acylsphingosine amidohydrolase 1 (Asah1)	0.43
Ascc2	NM_172581.1	activating signal cointegrator 1 complex subunit 2 (Ascc2)	0.55
Asns	NM_177166.2	asparagine synthetase (Asns)	0.48
Aspm	NM_001033375.2	asp (abnormal spindle)-like, microcephaly associated (Drosophila) (Aspm)	0.37
Atad3a	XM_489103	ATPase family, AAA domain containing 3A (Atad3a)	0.60
Atf4	AK038731	activating transcription factor 4 (Atf4)	0.37
Atf7ip	AK038962	activating transcription factor 7 interacting protein (Atf7ip)	0.55
Atg3	NM_172447.2	autophagy-related 3 (yeast) (Atg3)	0.60
Atic	NM_175287.3	5-aminoimidazole-4-carboxamide ribonucleotide formyltransferase/IMP cyclohydrolase (Atic)	0.55
Atp13a3	XM_989657.2	PREDICTED: ATPase type 13A3, transcript variant 1 (Atp13a3)	0.66
Atp1b3	NM_021427.1	ATPase, Na+/K+ transporting, beta 3 polypeptide (Atp1b3)	0.52
Atp5a1	NM_177118	ATP synthase, H+ transporting, mitochondrial F1 complex, alpha subunit, isoform 1 (Atp5a1), nuclear gene encoding mitochondrial protein	0.43
Atp5b		ATP synthase, H+ transporting mitochondrial F1 complex, beta subunit (Atp5b), nuclear gene encoding mitochondrial protein	0.59
Atp5c1	AK044025	ATP synthase, H+ transporting, mitochondrial F1 complex, gamma polypeptide 1 (Atp5c1)	0.51
Atp5d	NM_177225	ATP synthase, H+ transporting, mitochondrial F1 complex, delta subunit (Atp5d)	0.45
Atp5f1	NM_172768	ATP synthase, H+ transporting, mitochondrial F0 complex, subunit b, isoform 1 (Atp5f1), nuclear gene encoding mitochondrial protein	0.52
Atp5g2	AK044660	ATP synthase, H+ transporting, mitochondrial F0 complex, subunit c (subunit 9), isoform 2 (Atp5g2)	0.55
Atp5g3	NM_133954.1	ATP synthase, H+ transporting, mitochondrial F0 complex, subunit c (subunit 9), isoform 3 (Atp5g3), nuclear gene encoding mitochondrial protein	0.66
Atp5h	NM_153416.1	ATP synthase, H+ transporting, mitochondrial F0 complex, subunit d (Atp5h), nuclear gene encoding mitochondrial protein	0.55
Atp5j	NM_030210.1	ATP synthase, H+ transporting, mitochondrial F0 complex, subunit F (Atp5j), nuclear gene encoding mitochondrial protein	0.54
Atp5l	NM_178772.2	ATP synthase, H+ transporting, mitochondrial F0 complex, subunit g (Atp5l), nuclear gene encoding mitochondrial protein	0.44
Atp5o	NM_175503.3	ATP synthase, H+ transporting, mitochondrial F1 complex, O subunit (Atp5o), nuclear gene encoding mitochondrial protein	0.55
Atp6v0a1	NM_146217.3		0.62
Atp6v1c1	NM_144829.1	ATPase, H+ transporting, lysosomal V1 subunit C1 (Atp6v1c1)	0.62
Atp6v1d	NM_007377.3	ATPase, H+ transporting, lysosomal V1 subunit D (Atp6v1d)	0.63
Atp6v1e1	NM_172961.2	ATPase, H+ transporting, lysosomal V1 subunit E1 (Atp6v1e1)	0.60
Atp6v1h	NM_023732.2	ATPase, H+ transporting, lysosomal V1 subunit H (Atp6v1h)	0.58
Atpif1	XM_907304.2	ATPase inhibitory factor 1 (Atpif1), nuclear gene encoding mitochondrial protein	0.62
Atxn10	NM_172912.4	ataxin 10 (Atxn10)	0.49
AU040320	NM_001033336.2	expressed sequence AU040320 (AU040320), transcript variant 3	0.54
Aup1	NM_176839.1	ancient ubiquitous protein 1 (Aup1)	0.66
Aurka	NM_013854.1	aurora kinase A (Aurka)	0.19
Aurkaip1	NM_013853.1	aurora kinase A interacting protein 1 (Aurkaip1)	0.61
Aurkb	NR_003522.1	aurora kinase B (Aurkb)	0.25
AV028368	NM_029631.2		0.65
AW120700	NM_134130.1		0.55
B020018G12Rik	NM_198127.1	RIKEN cDNA B020018G12 gene (B020018G12Rik)	0.61
B230114H05Rik	NM_177678.6		0.58
B230208H17Rik	NM_177470	RIKEN cDNA B230208H17 gene (B230208H17Rik)	0.65
B230343A10Rik	NM_007382.1		0.56
B2m	NM_207223.1	beta-2 microglobulin (B2m)	0.62
B3galt6	NM_009338.3	UDP-Gal:betaGal beta 1,3-galactosyltransferase, polypeptide 6 (B3galt6)	0.51
B3gnt5	NM_133225.2	UDP-GlcNAc:betaGal beta-1,3-N-acetylglucosaminyltransferase 5 (B3gnt5)	0.66
B4galt3	NM_134037.2	UDP-Gal:betaGlcNAc beta 1,4-galactosyltransferase, polypeptide 3 (B4galt3)	0.50
Bag1	XM_355744.1	Bcl2-associated athanogene 1 (Bag1)	0.62

Bahcc1	NM_080633.2	BAH domain and coiled-coil containing 1 (Bahcc1)	0.42
Bai2	NM_012006.2	brain-specific angiogenesis inhibitor 2 (Bai2)	0.63
Banf1	NM_025590.3	barrier to autointegration factor 1 (Banf1), transcript variant 2	0.28
Bat1a	NM_133348.1	HLA-B-associated transcript 1A (Bat1a)	0.66
Bax	NM_153420.2	Bcl2-associated X protein (Bax)	0.64
BC003236	NM_053178.2		0.47
BC003885	NM_027976.2	cDNA sequence BC003885 (BC003885)	0.59
BC003965	NM_080575.1		0.52
BC017647	NM_019811.3	cDNA sequence BC017647 (BC017647)	0.45
BC019806	NM_007392.2		0.65
BC024659	NM_007393.3	PREDICTED: cDNA sequence BC024659 (BC024659)	0.61
BC031781	NM_019673.1	cDNA sequence BC031781 (BC031781)	0.66
BC039210	NM_134156.1	PREDICTED: cDNA sequence BC039210 (BC039210)	0.57
BC046331	NM_019785.1	cDNA sequence BC046331 (BC046331)	0.60
BC046404	NM_023735.1	cDNA sequence BC046404 (BC046404)	0.62
BC051227	NM_007400.2	cDNA sequence BC051227 (BC051227)	0.65
BC056474	NM_009614.2	cDNA sequence BC056474 (BC056474)	0.62
BC060632	NM_009615.5	cDNA sequence BC060632 (BC060632)	0.62
BC067047	NM_172053.2	cDNA sequence BC067047 (BC067047)	0.47
BC085271	NM_175506.3	cDNA sequence BC085271 (BC085271)	0.45
Bcas1	NM_172845.1	breast carcinoma amplified sequence 1 (Bcas1)	0.42
Bccip		BRCA2 and CDKN1A interacting protein (Bccip)	0.57
Bcl7c	XM_131451.2	B-cell CLL/lymphoma 7C (Bcl7c)	0.54
Bcs1l	NM_172723.4	BCS1-like (yeast) (Bcs1l)	0.66
Bdnf	NM_133770.1	brain derived neurotrophic factor (Bdnf), transcript variant 3	0.66
Bex2	NM_153534.2	brain expressed X-linked 2 (Bex2)	0.60
Bhlhb2	NM_009623.2	basic helix-loop-helix domain containing, class B2 (Bhlhb2)	0.57
Bicd2	NM_009627.1		0.62
Bin1	NM_001008533.2	bridging integrator 1 (Bin1)	0.57
Birc5	NM_028121.1	baculoviral IAP repeat-containing 5 (Birc5), transcript variant 1	0.30
Blm	NM_007414.3	Bloom syndrome homolog (human) (Blm), transcript variant 1	0.42
Bloc1s2	NM_007417.2		0.65
Blvra	NM_007420.2		0.62
Bmp7	NM_130863.1	bone morphogenetic protein 7 (Bmp7)	0.64
Bnip2	NM_019822.3	BCL2/adenovirus E1B interacting protein 2 (Bnip2), transcript variant alpha	0.55
Bok	NM_009634		0.31
Bola2	NM_010347	bolA-like 2 (E. coli) (Bola2)	0.50
Bola3	NM_054070.2	bolA-like 3 (E. coli) (Bola3)	0.51
Bop1	NM_001037136.1	block of proliferation 1 (Bop1)	0.56
Bpnt1	NM_010472.2	bisphosphate 3'-nucleotidase 1 (Bpnt1)	0.50
Brd2	NM_178162.2	bromodomain containing 2 (Brd2), transcript variant 1	0.65
Brip1	NM_018743.3	BRCA1 interacting protein C-terminal helicase 1 (Brip1)	0.63
Brrn1	NM_007428.3		0.22
Btaf1	NM_009642.4	BTAF1 RNA polymerase II, B-TFIID transcription factor-associated, (Mot1 homolog, S. cerevisiae) (Btaf1)	0.61
Btbd11	NM_026375.1	BTB (POZ) domain containing 11 (Btbd11), transcript variant 2	0.40
Btf3	NM_016661		0.57
Btg1	NM_145542.3	B-cell translocation gene 1, anti-proliferative (Btg1)	0.57
Bub1b	NM_009643.1		0.17
Bub3	NM_001002895.2	budding uninhibited by benzimidazoles 3 homolog (S. cerevisiae) (Bub3)	0.66
Bves	XM_136041.3		0.59
Bxdc1	XM_148986.1		0.58
Bxdc2	NM_177907.2	brix domain containing 2 (Bxdc2)	0.56
Bxdc5	NM_198860.1	brix domain containing 5 (Bxdc5), transcript variant 2	0.60
Bzw1	NM_178926.2		0.49
Bzw2	NM_182807.2	basic leucine zipper and W2 domains 2 (Bzw2)	0.42
C030003D03Rik		RIKEN cDNA C030003D03 gene (C030003D03Rik)	0.43
C030025P15Rik	NM_016666.2		0.64
C130007D14	NM_016895.3		0.66
C130032J12Rik	NM_009647.4	RIKEN cDNA C130032J12 gene (C130032J12Rik)	0.55
C1galt1c1	NM_031185.2	C1GALT1-specific chaperone 1 (C1galt1c1)	0.60
C230040D10Rik	NM_001033785.1	PREDICTED: RIKEN cDNA C230040D10 gene (C230040D10Rik)	0.66
C230053B09Rik	NM_018747.3		0.62
C230071H18Rik	NM_017476.2	RIKEN cDNA C230071H18 gene (C230071H18Rik)	0.61
C230078M08Rik	NM_023423.3	RIKEN cDNA C230078M08 gene (C230078M08Rik)	0.64
C330006P03Rik	NM_021473.2		0.48
C330023M02Rik	NM_013916.1	RIKEN cDNA C330023M02 gene (C330023M02Rik)	0.51
C530008M17Rik	NM_008525.3	PREDICTED: RIKEN cDNA C530008M17 gene, transcript variant 3 (C530008M1	0.46
C730029F17Rik	NM_020559.1		0.58
C78339	NM_019698.1		0.50
C79267	NM_007437		0.49

C79407	NM_172532	expressed sequence C79407 (C79407)	0.45
C920004C08Rik	NM_134042.2		0.21
Cacna1h	NM_007438.3	calcium channel, voltage-dependent, T type, alpha 1H subunit (Cacna1h)	0.54
Cad	NM_009657.3	carbamoyl-phosphate synthetase 2, aspartate transcarbamylase, and dihydroorotase (Cad)	0.66
Calm2	NM_199035.1	calmodulin 2 (Calm2)	0.22
Calr	NM_133981.1	calreticulin (Calr)	0.50
Calu	NM_001081395.1	calumenin (Calu), transcript variant 1	0.59
Camk1	NM_009667.2	calcium/calmodulin-dependent protein kinase I (Camk1)	0.54
Camk2b	NM_175007.1	calcium/calmodulin-dependent protein kinase II, beta (Camk2b)	0.32
Camk2n2	NM_007446.1	PREDICTED: calcium/calmodulin-dependent protein kinase II inhibitor 2 (Camk2	0.53
Camkk2	NM_001042711.2	calcium/calmodulin-dependent protein kinase kinase 2, beta (Camkk2)	0.53
Camta1	NM_021505.2	calmodulin binding transcription activator 1 (Camta1), transcript variant 1	0.59
Cand1	NM_009640.3	PREDICTED: cullin associated and neddylation disassociated 1, transcript variant 1 (Cand1)	0.64
Caprin1	NM_007426.3	cell cycle associated protein 1 (Caprin1)	0.59
Car2	NM_020581.1	carbonic anhydrase 2 (Car2)	0.43
Cask	NM_020332.3	calcium/calmodulin-dependent serine protein kinase (MAGUK family) (Cask)	0.37
Cbfb	NM_170687.3	core binding factor beta (Cbfb)	0.29
Cbr3	NM_028390.2	carbonyl reductase 3 (Cbr3)	0.58
Cbx3	NM_023210.3		0.51
Cbx5	NM_013469.1	chromobox homolog 5 (Drosophila HP1a) (Cbx5), transcript variant 2	0.58
Ccdc101	NM_013470.1	coiled-coil domain containing 101 (Ccdc101)	0.65
Ccdc124	NM_009673.1	coiled-coil domain containing 124 (Ccdc124)	0.60
Ccdc43	NM_001035854.2	coiled-coil domain containing 43 (Ccdc43)	0.64
Ccdc58	NM_029505.1	coiled-coil domain containing 58 (Ccdc58)	0.61
Ccdc86	NM_021710.1	coiled-coil domain containing 86 (Ccdc86)	0.63
Ccdc99	NM_001042558.1	coiled-coil domain containing 99 (Ccdc99)	0.57
Ccnb1	NM_009687.1	cyclin B1 (Ccnb1)	0.13
Ccnd1	NM_027263.1		0.06
Ccnd2	NM_009691.2		0.23
Ccne2	NM_009696.2	cyclin E2 (Ccne2), transcript variant 1	0.67
Ccng1	NM_025825	cyclin G1 (Ccng1)	0.64
Ccnh	NM_175310.5	cyclin H (Ccnh)	0.61
Ccnyl1	NM_009698.1	cyclin Y-like 1 (Ccnyl1)	0.41
Cct3	NM_009700.1	chaperonin containing Tcp1, subunit 3 (gamma) (Cct3)	0.48
Cct4	NM_022026.2	chaperonin containing Tcp1, subunit 4 (delta) (Cct4)	0.52
Cct6a	NM_018790.2	chaperonin containing Tcp1, subunit 6a (zeta) (Cct6a)	0.48
Cct8	NM_019870.1	chaperonin containing Tcp1, subunit 8 (theta) (Cct8)	0.45
Cd164	NM_019870.2	CD164 antigen (Cd164)	0.62
Cd276	NM_009704.3	CD276 antigen (Cd276)	0.39
Cd8b	NM_007477.4		0.64
Cdc20	NM_007481.3	cell division cycle 20 homolog (S. cerevisiae) (Cdc20)	0.19
Cdc25a	NM_176837.2	cell division cycle 25 homolog A (S. pombe) (Cdc25a)	0.62
Cdc25c	NM_183019.2	cell division cycle 25 homolog C (S. pombe) (Cdc25c)	0.63
Cdc2a	NM_024191.1	cell division cycle 2 homolog A (S. pombe) (Cdc2a)	0.16
Cdc42	NM_177305.3	cell division cycle 42 homolog (S. cerevisiae) (Cdc42)	0.60
Cdc42ep1	NM_019419.1	CDC42 effector protein (Rho GTPase binding) 1 (Cdc42ep1)	0.65
Cdc42se1	NM_001081083.1	CDC42 small effector 1 (Cdc42se1), transcript variant 1	0.57
Cdc45l	NM_030184.2	cell division cycle 45 homolog (S. cerevisiae)-like (Cdc45l)	0.48
Cdc5l	NM_026139.3	cell division cycle 5-like (S. pombe) (Cdc5l)	0.60
Cdc6	NM_023142.1	cell division cycle 6 homolog (S. cerevisiae) (Cdc6), transcript variant 1	0.58
Cdc7	NM_029711.1	cell division cycle 7 (S. cerevisiae) (Cdc7)	0.31
Cdca2	NM_019824.3	cell division cycle associated 2 (Cdca2)	0.25
Cdca3	NM_178917.2	cell division cycle associated 3 (Cdca3)	0.11
Cdca4	NM_009712.3	cell division cycle associated 4 (Cdca4)	0.44
Cdca5	NM_028710.2	cell division cycle associated 5 (Cdca5)	0.58
Cdca7	NM_019734.1	cell division cycle associated 7 (Cdca7)	0.21
Cdca8	NM_029291.1	cell division cycle associated 8 (Cdca8)	0.13
Cdh13	NM_012055.1	cadherin 13 (Cdh13)	0.35
Cdk2	NM_028386.1	cyclin-dependent kinase 2 (Cdk2), transcript variant 1	0.41
Cdk4	NM_009791.4	cyclin-dependent kinase 4 (Cdk4)	0.47
Cdk8	NM_007494.3	cyclin-dependent kinase 8 (Cdk8)	0.62
Cdkn1a	NM_179203.1	cyclin-dependent kinase inhibitor 1A (P21) (Cdkn1a)	0.49
Cdkn2aipnl	NM_007498.2	CDKN2A interacting protein N-terminal like (Cdkn2aipnl)	0.55
Cdkn3	NM_009716.2	PREDICTED: cyclin-dependent kinase inhibitor 3, transcript variant 5 (Cdkn3)	0.24
Cdon	NM_019426.2		0.62
Cdr2	NM_026217.1	cerebellar degeneration-related 2 (Cdr2)	0.40
Cdr2l	NM_026402.3	cerebellar degeneration-related protein 2-like (Cdr2l)	0.44
Cdt1	NM_026195.1	chromatin licensing and DNA replication factor 1 (Cdt1)	0.28
Cebpd	XM_001480958.1	CCAAT/enhancer binding protein (C/EBP), delta (Cebpd)	0.66

Cenpa	NM_144900.1	centromere protein A (Cenpa)	0.06
Cenpb	NM_009721.4	centromere protein B (Cenpb)	0.62
Cenpf	NM_013415.5	PREDICTED: centromere autoantigen F, transcript variant 3 (Cenpf)	0.23
Cenpi	NM_007502.3	centromere protein I (Cenpi)	0.62
Cenpk	NM_007505.2	centromere protein K (Cenpk), transcript variant 1	0.45
Cenpl	NM_016774.3	centromere protein L (Cenpl)	0.41
Cenpm	NM_020615.3	centromere protein M (Cenpm), transcript variant 2	0.57
Cenpn	NM_025313.1	centromere protein N (Cenpn)	0.55
Cenpp	NM_009725.3	centromere protein P (Cenpp)	0.58
Cenpq	NM_026468.1	centromere protein Q (Cenpq)	0.57
Centd1	NM_175015.2	PREDICTED: centaurin, delta 1, transcript variant 5 (Centd1)	0.56
Cep152	NM_027862.1	centrosomal protein 152 (Cep152)	0.63
Cep170	NM_016755.2	centrosomal protein 170 (Cep170)	0.61
Cep55	NM_013795.4	centrosomal protein 55 (Cep55)	0.60
Cep78	NM_138597.2	centrosomal protein 78 (Cep78)	0.59
Cetn3	NM_016920.1	centrin 3 (Cetn3)	0.63
Cfl1	NM_025494.2	cofilin 1, non-muscle (Cfl1)	0.64
Cggbp1	NM_023721.2	CGG triplet repeat binding protein 1 (Cggbp1)	0.63
Chaf1a	NM_007510.2	chromatin assembly factor 1, subunit A (p150) (Chaf1a)	0.60
Chaf1b	NM_133826.2	chromatin assembly factor 1, subunit B (p60) (Chaf1b)	0.31
Chchd1	NM_009727.2	coiled-coil-helix-coiled-coil-helix domain containing 1 (Chchd1)	0.47
Chchd4	NM_007512.2	coiled-coil-helix-coiled-coil-helix domain containing 4 (Chchd4), nuclear gene encoding mitochondrial protein	0.61
Chd1	NM_009124.4		0.60
Chmp4b	NM_016843.3	chromatin modifying protein 4B (Chmp4b)	0.57
Chmp7	NM_177629.2	CHMP family, member 7 (Chmp7)	0.64
Chn2	NM_133886.2	chimerin (chimaerin) 2 (Chn2)	0.27
Chrm3	NM_007517.3	cholinergic receptor, muscarinic 3, cardiac (Chrm3)	0.56
Chst11	NM_011497.3	carbohydrate sulfotransferase 11 (Chst11)	0.66
Chst2	NM_025338.3	carbohydrate sulfotransferase 2 (Chst2)	0.49
Chst7	NM_011496.1	carbohydrate (N-acetylglucosamino) sulfotransferase 7 (Chst7)	0.15
Chtf18	NM_177672	CTF18, chromosome transmission fidelity factor 18 homolog (S. cerevisiae) (Chtf18)	0.55
Cirh1a		cirrhosis, autosomal recessive 1A (human) (Cirh1a)	0.59
Ckap2	XM_127132.3		0.32
Ckap2l	NM_009465.3	cytoskeleton associated protein 2-like (Ckap2l)	0.57
Cldnd1	NM_001039518.1	claudin domain containing 1 (Cldnd1)	0.52
Clic1			0.54
Clic4	AK045406	chloride intracellular channel 4 (mitochondrial) (Clic4), nuclear gene encoding mitochondrial protein	0.43
Cln5	NM_001024616.1		0.47
Clns1a	XM_981366.1	chloride channel, nucleotide-sensitive, 1A (Clns1a)	0.60
Clpp	XM_001001884.2	caseinolytic peptidase, ATP-dependent, proteolytic subunit homolog (E. coli) (Clpp)	0.61
Clspn	NM_133898.3	claspin homolog (Xenopus laevis) (Clspn)	0.28
Clta	AK046118	clathrin, light polypeptide (Lca) (Clta), transcript variant 2	0.63
Cltc			0.56
Cmas	NM_009735.2	cytidine monophospho-N-acetylneuraminic acid synthetase (Cmas)	0.61
Cmc1	NM_033149.2	COX assembly mitochondrial protein homolog (S. cerevisiae) (Cmc1), nuclear gene encoding mitochondrial protein	0.56
Cmpk	NM_080445.4	cytidylate kinase (Cmpk)	0.63
Cnih	NM_029792.1	cornichon homolog (Drosophila) (Cnih)	0.60
Cnih4	NM_054052.2	cornichon homolog 4 (Drosophila) (Cnih4)	0.43
Cntnap4	NM_020579.1		0.42
Col16a1	AK046967	collagen, type XVI, alpha 1 (Col16a1)	0.30
Col18a1	AK047284	procollagen, type XVIII, alpha 1 (Col18a1)	0.11
Commd1	NM_013717.2	COMM domain containing 1 (Commd1)	0.59
Cops6	NM_019517.2	COP9 (constitutive photomorphogenic) homolog, subunit 6 (Arabidopsis thaliana) (Cops6)	0.61
Cops7a	NM_009736.1	COP9 (constitutive photomorphogenic) homolog, subunit 7a (Arabidopsis thaliana) (Cops7a)	0.61
Coq7	NM_198423.2	demethyl-Q 7 (Coq7)	0.64
Coro1c	NM_173071.1	coronin, actin binding protein 1C (Coro1c)	0.40
Cox10	NM_001038231.1	COX10 homolog, cytochrome c oxidase assembly protein, heme A: farnesyltransferase (yeast) (Cox10), nuclear gene encoding mitochondrial protein	0.63
Cox17	NM_019693.2	cytochrome c oxidase, subunit XVII assembly protein homolog (yeast) (Cox17)	0.48
Cox18	NM_007527.2	COX18 cytochrome c oxidase assembly homolog (S. cerevisiae) (Cox18)	0.60
Cox5a	NM_172742	cytochrome c oxidase, subunit Va (Cox5a), nuclear gene encoding mitochondrial protein	0.42

Cox5b	NM_133234.1		0.64
Cox6a1	NM_130452.1		0.57
Cox6b1	NM_026116.2	cytochrome c oxidase, subunit VIb polypeptide 1 (Cox6b1)	0.56
Cox6c	NM_175325.2	cytochrome c oxidase, subunit VIc (Cox6c)	0.62
Cox7a2	NM_030249	cytochrome c oxidase, subunit VIIa 2 (Cox7a2)	0.46
Cox7a2l	NM_198609.2	cytochrome c oxidase subunit VIIa polypeptide 2-like (Cox7a2l)	0.66
Cplx1	XM_128528.2	complexin 1 (Cplx1)	0.61
Cpne8	NM_145430.1	copine VIII (Cpne8), transcript variant 2	0.50
Creld2	NM_145460	cysteine-rich with EGF-like domains 2 (Creld2)	0.57
Crlf3	NM_145450.3	cytokine receptor-like factor 3 (Crlf3)	0.66
Crtap	NM_146237.1	cartilage associated protein (Crtap)	0.52
Cs	XM_001472442.1	citrate synthase (Cs), nuclear gene encoding mitochondrial protein	0.38
Csad	NM_173421.1		0.67
Csda	NM_153584.1	cold shock domain protein A (Csda)	0.52
Csde1	NM_145943.1	cold shock domain containing E1, RNA binding (Csde1)	0.62
Cse1l	XM_196478	chromosome segregation 1-like (S. cerevisiae) (Cse1l)	0.53
Csk	XM_001481316.1	c-src tyrosine kinase (Csk)	0.66
Csnk1d	NM_177673.2	casein kinase 1, delta (Csnk1d), transcript variant 2	0.61
Csnk1e	NM_198861.1	casein kinase 1, epsilon (Csnk1e)	0.64
Csnk1g2	NM_178418.2	casein kinase 1, gamma 2 (Csnk1g2)	0.66
Csnk2a1-rs3	NM_183170.2		0.56
Csnk2a2	NM_001001493.2	casein kinase 2, alpha prime polypeptide (Csnk2a2)	0.58
Cspg5	NM_198625.1	chondroitin sulfate proteoglycan 5 (Cspg5)	0.49
Csrp2	NM_177782.3	cysteine and glycine-rich protein 2 (Csrp2)	0.48
Cstf1	NM_001008551.1	cleavage stimulation factor, 3' pre-RNA, subunit 1 (Cstf1)	0.64
Ctgf	NM_013867.1		0.49
Ctps	NM_029815.1	cytidine 5'-triphosphate synthase (Ctps)	0.60
Ctsc	NM_138681.3	cathepsin C (Ctsc)	0.39
Ctsh	NM_025392.1	cathepsin H (Ctsh)	0.59
Cttnbp2nl	NM_207680.2	CTTNBP2 N-terminal like (Cttnbp2nl)	0.41
Cugbp1	NM_009746.2	CUG triplet repeat, RNA binding protein 1 (Cugbp1), transcript variant 2	0.60
Cugbp2	NM_025784.2	CUG triplet repeat, RNA binding protein 2 (Cugbp2), transcript variant 6	0.47
Cul3	NM_027208.1	cullin 3 (Cul3)	0.58
Cx3cl1	NM_001048141.1	chemokine (C-X3-C motif) ligand 1 (Cx3cl1)	0.42
Cxadr	NM_009749.1	coxsackie virus and adenovirus receptor (Cxadr), transcript variant 1	0.33
Cxcl1	NM_011498.4	chemokine (C-X-C motif) ligand 1 (Cxcl1)	0.46
Cxcl4	NM_031397.2	chemokine (C-X-C motif) ligand 4 (Cxcl4)	0.47
Cyc1	NM_029791	cytochrome c-1 (Cyc1)	0.37
Cycs	NM_009668.1	cytochrome c, somatic (Cycs), nuclear gene encoding mitochondrial protein	0.27
Cyp51		cytochrome P450, family 51 (Cyp51)	0.23
D030038A19Rik	NM_009689.2		0.65
D030056L22Rik	NM_007550.3	RIKEN cDNA D030056L22 gene (D030056L22Rik)	0.58
D10627	XM_193940.2	cDNA sequence D10627 (D10627)	0.64
D10Ertd322e	NM_026678.3	DNA segment, Chr 10, ERATO Doi 322, expressed (D10Ertd322e)	0.49
D10Wsu102e	NM_007557.2	DNA segment, Chr 10, Wayne State University 102, expressed (D10Wsu102e)	0.60
D10Wsu52e	NM_007560.3	DNA segment, Chr 10, Wayne State University 52, expressed (D10Wsu52e)	0.58
D12Ertd553e	NM_001008238.2	DNA segment, Chr 12, ERATO Doi 553, expressed (D12Ertd553e)	0.61
D12Wsu95e	NM_016778		0.46
D130004H04Rik	NM_175103.2		0.62
D15Mgi27	NM_175277.2	DNA Segment, Chr 15, Mouse Genome Informatics 27 (D15Mgi27)	0.60
D17H6S56E-5	NM_013481.1	DNA segment, Chr 17, human D6S56E 5 (D17H6S56E-5)	0.55
D17Wsu104e	NM_011794.3		0.56
D19Bwg1357e	NM_010238.3	DNA segment, Chr 19, Brigham & Women's Genetics 1357 expressed (D19Bwg1357e)	0.48
D1Ertd471e	NM_178309.1		0.48
D2Bwg1335e	NM_144818	DNA segment, Chr 2, Brigham & Women's Genetics 1335 expressed (D2Bwg1335e)	0.50
D2Ertd750e	NM_001080706.1	DNA segment, Chr 2, ERATO Doi 750, expressed (D2Ertd750e)	0.35
D4Bwg0951e	NM_001017525.1	DNA segment, Chr 4, Brigham & Women's Genetics 0951 expressed (D4Bwg0951e)	0.52
D6Wsu176e	NM_145455.1		0.60
D930046M13Rik	NM_007569.1		0.41
D9Wsu20e	NM_009773		0.58
Dad1	NM_009774.3		0.63
Dap3	NM_024285	death associated protein 3 (Dap3), nuclear gene encoding mitochondrial protein	0.67
Dazap1	NM_023323		0.33
Dbf4	NM_026396.2	DBF4 homolog (S. cerevisiae) (Dbf4)	0.29
Dbt	NM_027371.2	dihydrolipoamide branched chain transacylase E2 (Dbt)	0.61
Dbx2	NM_172449.1		0.56
Dclk1	NM_025824	doublecortin-like kinase 1 (Dclk1)	0.27
Dcps	NM_025840.2	decapping enzyme, scavenger (Dcps)	0.64

Dcun1d5	NM_029881.2	DCN1, defective in cullin neddylation 1, domain containing 5 (S. cerevisiae) (Dcun1d5)	0.62
Ddost		dolichyl-di-phosphooligosaccharide-protein glycotransferase (Ddost)	0.55
Ddx1	NM_177287	DEAD (Asp-Glu-Ala-Asp) box polypeptide 1 (Ddx1)	0.55
Ddx11			0.39
Ddx20	XM_975397.2	DEAD (Asp-Glu-Ala-Asp) box polypeptide 20 (Ddx20)	0.59
Ddx21	NM_177914.1	DEAD (Asp-Glu-Ala-Asp) box polypeptide 21 (Ddx21)	0.53
Ddx25		DEAD (Asp-Glu-Ala-Asp) box polypeptide 25 (Ddx25)	0.61
Ddx26	NM_178684.3		0.64
Ddx27	AK081724	DEAD (Asp-Glu-Ala-Asp) box polypeptide 27 (Ddx27)	0.65
Ddx3x	NM_021550.2	DEAD/H (Asp-Glu-Ala-Asp/His) box polypeptide 3, X-linked (Ddx3x)	0.63
Ddx54	NM_207233.1	DEAD (Asp-Glu-Ala-Asp) box polypeptide 54 (Ddx54)	0.53
Ddx6	XM_976346.1	DEAD (Asp-Glu-Ala-Asp) box polypeptide 6 (Ddx6)	0.57
Deb1	AK048765	differentially expressed in B16F10 1 (Deb1)	0.48
Dek	AK082620	DEK oncogene (DNA binding) (Dek)	0.61
Dennd2a	NM_207265.2	DENN/MADD domain containing 2A (Dennd2a)	0.35
Dgkd	NM_176995.2		0.57
Dgkk	NM_009778.1	diacylglycerol kinase kappa (Dgkk)	0.49
Dgkz		diacylglycerol kinase zeta (Dgkz)	0.47
Dhcr24	NM_172722.3		0.37
Dhcr7	NM_175286.3	7-dehydrocholesterol reductase (Dhcr7)	0.38
Dhh	NM_011413.2	desert hedgehog (Dhh)	0.42
Dhps	NM_009780.1	deoxyhypusine synthase (Dhps)	0.66
Dhrsx	XM_916109.2	dehydrogenase/reductase (SDR family) X chromosome (Dhrsx)	0.53
Dhx15		DEAH (Asp-Glu-Ala-His) box polypeptide 15 (Dhx15), transcript variant 2	0.44
Dhx30	AK049826	DEAH (Asp-Glu-Ala-His) box polypeptide 30 (Dhx30)	0.65
Diras2	AK050233		0.55
Dlgap5	XM_127312	discs, large (Drosophila) homolog-associated protein 5 (Dlgap5)	0.46
Dlst	NM_183148		0.51
Dnahc11	NM_172578.2	dynein, axonemal, heavy chain 11 (Dnahc11)	0.32
Dnajb11			0.47
Dnajc19	NM_198037.1	DnaJ (Hsp40) homolog, subfamily C, member 19 (Dnajc19), transcript variant 2	0.65
Dnajc2	NM_021415.3	DnaJ (Hsp40) homolog, subfamily C, member 2 (Dnajc2)	0.52
Dnmt1	NM_009785.1	DNA methyltransferase (cytosine-5) 1 (Dnmt1)	0.36
Dock1	NM_146123.2	dedicator of cytokinesis 1 (Dock1)	0.65
Dock10	NM_023525.1		0.43
Dos	NM_007589.4	downstream of Stk11 (Dos)	0.55
Dpm3	NM_007591.3		0.65
Dpp3	NM_007594.3	dipeptidylpeptidase 3 (Dpp3)	0.62
Dpy30	NM_133926.2	dpy-30 homolog (C. elegans) (Dpy30)	0.56
Dpysl3	NM_007595.3	dihydropyrimidinase-like 3 (Dpysl3)	0.60
Drg1	NM_178597.4	developmentally regulated GTP binding protein 1 (Drg1)	0.64
Dtymk	XM_990809.1		0.61
Dusp1	NM_145358.1	dual specificity phosphatase 1 (Dusp1)	0.35
Dusp4	NM_001081557.1	dual specificity phosphatase 4 (Dusp4)	0.36
Dusp6	XM_125901.8	dual specificity phosphatase 6 (Dusp6)	0.10
Dusp7	NM_007600.2	dual specificity phosphatase 7 (Dusp7)	0.29
Dynll1	NM_007602.3	dynein light chain LC8-type 1 (Dynll1)	0.62
E030026I10Rik	NM_016739.2		0.56
E130016E03Rik	NM_009801.3	RIKEN cDNA E130016E03 gene (E130016E03Rik)	0.33
E130306D19Rik	NM_025821.2		0.48
E230013M07Rik	NM_009806.2		0.31
E2f1	NM_009814.1	E2F transcription factor 1 (E2f1)	0.32
E2f2	NM_009804.1	E2F transcription factor 2 (E2f2)	0.54
E2f6	NM_022309.3	E2F transcription factor 6 (E2f6)	0.64
E330016A19Rik	NM_173047.2	RIKEN cDNA E330016A19 gene (E330016A19Rik)	0.46
E430028B21Rik	NM_007624	RIKEN cDNA E430028B21 gene (E430028B21Rik)	0.64
E430039I23Rik	NM_001076789.1		0.43
E530011J04Rik	NM_172274.1		0.60
Ebna1bp2	NM_029339.2	EBNA1 binding protein 2 (Ebna1bp2)	0.45
Ebp	NM_026964.3	phenylalkylamine Ca2+ antagonist (emopamil) binding protein (Ebp)	0.66
Ece1	XM_355284.5	endothelin converting enzyme 1 (Ece1)	0.53
Ecm1	NM_026222.2	extracellular matrix protein 1 (Ecm1)	0.63
Edf1	NM_025918.3	endothelial differentiation-related factor 1 (Edf1)	0.56
Eef1a1	NM_198645.1	eukaryotic translation elongation factor 1 alpha 1 (Eef1a1)	0.62
Eef1b2	NM_023731.2	eukaryotic translation elongation factor 1 beta 2 (Eef1b2)	0.48
Eef1e1	NM_025725.2	eukaryotic translation elongation factor 1 epsilon 1 (Eef1e1)	0.33
Eef1g	NM_027411.1	eukaryotic translation elongation factor 1 gamma (Eef1g)	0.50
Efnb1	NM_172301.3	ephrin B1 (Efnb1)	0.66
Efs	NM_007631.1	embryonal Fyn-associated substrate (Efs)	0.42

EG216185	NM_009829	PREDICTED: predicted gene, EG216185 (EG216185)	0.50
EG237361	NM_001037134.1	PREDICTED: predicted gene, EG237361 (EG237361)	0.36
EG243642	NM_009831.2	PREDICTED: predicted gene, EG243642 (EG243642)	0.41
EG245297	NM_023243.4	predicted gene, EG245297 (EG245297)	0.64
EG268795	NM_001097644.1	PREDICTED: predicted gene, EG268795, transcript variant 4 (EG268795)	0.45
EG269105	NM_053180.2	PREDICTED: predicted gene, EG269105 (EG269105)	0.57
EG382843	NM_009836.1	PREDICTED: predicted gene, EG382843 (EG382843)	0.51
EG432448	NM_009837.1	PREDICTED: predicted gene, EG432448, transcript variant 1 (EG432448)	0.63
EG432681	NM_009838.1	PREDICTED: predicted gene, EG432681 (EG432681)	0.47
EG432721	NM_009840.3	PREDICTED: predicted gene, EG432721 (EG432721)	0.39
EG432879	NM_153098.2	predicted gene, EG432879 (EG432879)	0.58
EG433144	NM_016898.2	PREDICTED: predicted gene, EG433144 (EG433144)	0.62
EG433182	NM_133983.3	predicted gene, EG433182 (EG433182)	0.58
EG433923	NM_007652.2	predicted gene, EG433923 (EG433923)	0.53
EG434404	NM_007656.4	PREDICTED: predicted gene, EG434404 (EG434404)	0.57
EG434858	NM_009858	predicted gene, EG434858 (EG434858), non-coding RNA.	0.64
EG545056	NM_023223.1	predicted gene, EG545056 (EG545056), non-coding RNA.	0.65
EG622236	NM_007658.3	PREDICTED: predicted gene, EG622236, transcript variant 1 (EG622236)	0.49
EG622339	NM_009860.2	predicted gene, EG622339 (EG622339)	0.51
EG623818	NM_007659.3	PREDICTED: predicted gene, EG623818 (EG623818)	0.57
EG624124	NM_009861.1	PREDICTED: predicted gene, EG624124, transcript variant 1 (EG624124)	0.48
EG625054	NM_027219.3	predicted gene, EG625054 (EG625054), non-coding RNA.	0.58
EG625917	NM_020006.1	PREDICTED: predicted gene, EG625917 (EG625917)	0.49
EG626175	NM_172395.2	PREDICTED: predicted gene, EG626175 (EG626175)	0.40
EG629595	NM_009862.1	PREDICTED: predicted gene, EG629595 (EG629595)	0.42
EG632248	NM_152810.1	PREDICTED: predicted gene, EG632248 (EG632248)	0.42
EG633120	NM_011799.2	PREDICTED: predicted gene, EG633120 (EG633120), misc RNA.	0.53
EG637748	NM_009863.2	PREDICTED: predicted gene, EG637748 (EG637748)	0.49
EG665369	NM_175384.3	PREDICTED: predicted gene, EG665369 (EG665369)	0.52
EG666577	NM_013538.4	PREDICTED: predicted gene, EG666577 (EG666577)	0.45
EG666609	NM_028023.3	PREDICTED: predicted gene, EG666609 (EG666609)	0.61
EG667190	NM_026410.1	PREDICTED: predicted gene, EG667190 (EG667190), misc RNA.	0.30
EG667728	NM_025866.3	PREDICTED: predicted gene, EG667728 (EG667728), misc RNA.	0.22
EG668668	NM_026560.3	predicted gene, EG668668 (EG668668)	0.65
EG668829	NM_019707.4	PREDICTED: predicted gene, EG668829 (EG668829)	0.53
EG668850	NM_183417.2	PREDICTED: predicted gene, EG668850 (EG668850), misc RNA.	0.51
Egr1	NM_009870.2	early growth response 1 (Egr1)	0.60
Ehd4	NM_153599.3	EH-domain containing 4 (Ehd4)	0.35
Eif1a	NM_007669.2	eukaryotic translation initiation factor 1A (Eif1a)	0.61
Eif1ad	NM_029976.2	eukaryotic translation initiation factor 1A domain containing (Eif1ad)	0.46
Eif2b3	XM_919022.2		0.56
Eif2s2	NM_033037.3	eukaryotic translation initiation factor 2, subunit 2 (beta) (Eif2s2)	0.61
Eif2s3x	AK040711	eukaryotic translation initiation factor 2, subunit 3, structural gene X-linked (Eif2s3x)	0.56
Eif3a	NM_007672.1	eukaryotic translation initiation factor 3, subunit A (Eif3a)	0.50
Eif3b	NM_001080929.1	eukaryotic translation initiation factor 3, subunit B (Eif3b)	0.66
Eif3d	NM_026014.3	eukaryotic translation initiation factor 3, subunit D (Eif3d)	0.59
Eif3eip	NM_007679.4	eukaryotic translation initiation factor 3, subunit E interacting protein (Eif3eip)	0.65
Eif3f	NM_009886.2	eukaryotic translation initiation factor 3, subunit F (Eif3f)	0.66
Eif3g	NM_007681.2	eukaryotic translation initiation factor 3, subunit G (Eif3g)	0.49
Eif3i	NM_007682.2	eukaryotic translation initiation factor 3, subunit I (Eif3i)	0.58
Eif3k	XM_916605.2	eukaryotic translation initiation factor 3, subunit K (Eif3k)	0.62
Eif3s4	NM_145924.2		0.46
Eif3s5	NM_021790.1		0.65
Eif4a1	NM_027429.1	eukaryotic translation initiation factor 4A1 (Eif4a1)	0.28
Eif4a3	NM_178269.3	eukaryotic translation initiation factor 4A, isoform 3 (Eif4a3)	0.66
Eif4ebp1	NM_028131.3	eukaryotic translation initiation factor 4E binding protein 1 (Eif4ebp1)	0.51
Eif4el3	NM_025495.1		0.55
Eif4g1	NM_031863.3	eukaryotic translation initiation factor 4, gamma 1 (Eif4g1), transcript variant 2	0.61
Eif5	XM_001001363.1	eukaryotic translation initiation factor 5 (Eif5), transcript variant 1	0.58
Eif5a	NM_001081091.1	eukaryotic translation initiation factor 5A (Eif5a)	0.39
Eif6	NM_001099637.1	eukaryotic translation initiation factor 6 (Eif6)	0.62
Elavl1	NM_028760.1	ELAV (embryonic lethal, abnormal vision, Drosophila)-like 1 (Hu antigen R) (Elavl1)	0.52
Elk3	NM_198019.2	ELK3, member of ETS oncogene family (Elk3), transcript variant 2	0.43
Elmo1	NM_207298.2	engulfment and cell motility 1, ced-12 homolog (C. elegans) (Elmo1), transcript variant 2	0.58
Elovl1	NM_007684.3	elongation of very long chain fatty acids (FEN1/Elo2, SUR4/Elo3, yeast)-like 1 (Elovl1), transcript variant 2	0.51
Elovl4	NM_145825.1	elongation of very long chain fatty acids (FEN1/Elo2, SUR4/Elo3, yeast)-like 4 (Elovl4)	0.63

Elovl5	NM_007687.2	ELOVL family member 5, elongation of long chain fatty acids (yeast) (Elovl5)	0.62
Elovl6	NM_008823.3	ELOVL family member 6, elongation of long chain fatty acids (yeast) (Elovl6)	0.21
Emg1	NM_178647.2	EMG1 nucleolar protein homolog (S. cerevisiae) (Emg1)	0.53
Eno1	NM_013733.3	enolase 1, alpha non-neuron (Eno1)	0.39
Entpd4	NM_028083.3	ectonucleoside triphosphate diphosphohydrolase 4 (Entpd4)	0.52
Epn2	NM_025366.2		0.20
Ercc1	NM_133928.2	excision repair cross-complementing rodent repair deficiency, complementation group 1 (Ercc1)	0.64
Ergic1	AK021188	endoplasmic reticulum-golgi intermediate compartment (ERGIC) 1 (Ergic1)	0.39
Ergic2	NM_007695.2	ERGIC and golgi 2 (Ergic2), transcript variant 1	0.59
Erh	NM_001025566.1	enhancer of rudimentary homolog (Drosophila) (Erh)	0.58
Erlin1	NM_029362.3	ER lipid raft associated 1 (Erlin1)	0.62
Ermp1	NM_134078.3	endoplasmic reticulum metallopeptidase 1 (Ermp1)	0.32
Erp29	NM_023543.1	endoplasmic reticulum protein 29 (Erp29)	0.64
Errfi1	NM_033269.2	ERBB receptor feedback inhibitor 1 (Errfi1)	0.62
Esco2	NM_021439.2	establishment of cohesion 1 homolog 2 (S. cerevisiae) (Esco2)	0.31
Espl1	NM_018763.1	extra spindle poles-like 1 (S. cerevisiae) (Espl1)	0.65
Esrra	NM_021715.1	estrogen related receptor, alpha (Esrra)	0.55
Esrrb	NM_145409.1	estrogen related receptor, beta (Esrrb)	0.52
Etf1	NM_011574.1	eukaryotic translation termination factor 1 (Etf1)	0.54
Ets1	XM_134100.3	E26 avian leukemia oncogene 1, 5' domain (Ets1), transcript variant 2	0.66
Ets2	NM_181589.2	E26 avian leukemia oncogene 2, 3' domain (Ets2)	0.48
Etv1	NM_029437.1	ets variant gene 1 (Etv1)	0.35
Etv4	NM_008770.2	ets variant gene 4 (E1A enhancer binding protein, E1AF) (Etv4)	0.38
Etv5	NM_171826.2	ets variant gene 5 (Etv5)	0.22
Exosc1	NM_033444.1	exosome component 1 (Exosc1)	0.55
Exosc2	NM_013885.2	exosome component 2 (Exosc2)	0.66
Exosc6	NM_030179.2	exosome component 6 (Exosc6)	0.65
Exosc7	NM_001042634.1	exosome component 7 (Exosc7)	0.64
Exosc8	NM_007714.2	exosome component 8 (Exosc8)	0.51
Ext1	XM_127882.3	exostoses (multiple) 1 (Ext1)	0.61
Extl3	NM_023671.1	exostoses (multiple)-like 3 (Extl3)	0.58
Ezh2	NM_017393.1	enhancer of zeste homolog 2 (Drosophila) (Ezh2)	0.35
F2r	NM_175554.3	coagulation factor II (thrombin) receptor (F2r)	0.36
F630043A04Rik	NM_001080385.1	RIKEN cDNA F630043A04 gene (F630043A04Rik)	0.58
Faah	NM_001003908	fatty acid amide hydrolase (Faah)	0.66
Fabp5	NM_009908.1	fatty acid binding protein 5, epidermal (Fabp5)	0.30
Fabp7	NM_026442.2		0.58
Faf1	NM_023160.1	Fas-associated factor 1 (Faf1)	0.58
Fahd1	NM_023455.2	fumarylacetoacetate hydrolase domain containing 1 (Fahd1), nuclear gene encoding mitochondrial protein	0.62
Fam101a	NM_025647.2	family with sequence similarity 101, member A (Fam101a)	0.48
Fam108a	NM_026066.2	family with sequence similarity 108, member A (Fam108a)	0.64
Fam110a	NM_009919.1	family with sequence similarity 110, member A (Fam110a), transcript variant 1	0.54
Fam120a	NM_030131.2	family with sequence similarity 120, member A (Fam120a)	0.56
Fam125a	NM_009923.1	family with sequence similarity 125, member A (Fam125a)	0.65
Fam131a	AK039251	family with sequence similarity 131, member A (Fam131a)	0.42
Fam171a1	NM_130457	family with sequence similarity 171, member A1 (Fam171a1)	0.49
Fam173a	NM_028266.4	family with sequence similarity 173, member A (Fam173a)	0.55
Fam84a	NM_009929.2	family with sequence similarity 84, member A (Fam84a)	0.38
Fancd2	XM_907370.3	Fanconi anemia, complementation group D2 (Fancd2)	0.66
Fance	NM_153393.1	PREDICTED: Fanconi anemia, complementation group E (Fance)	0.56
Farsb	NM_199473	phenylalanyl-tRNA synthetase, beta subunit (Farsb)	0.42
Fastk	NM_144514.2	Fas-activated serine/threonine kinase (Fastk)	0.61
Fblim1	AK018238	filamin binding LIM protein 1 (Fblim1)	0.55
Fbln1	NM_012002.3	fibulin 1 (Fbln1)	0.37
Fbln2	NM_012003.1	fibulin 2 (Fbln2), transcript variant 2	0.17
Fbn1	NM_009940.2		0.64
Fbxl10	NM_011779.3	F-box and leucine-rich repeat protein 10 (Fbxl10), transcript variant 3	0.51
Fbxl14	NM_178379.2	F-box and leucine-rich repeat protein 14 (Fbxl14)	0.51
Fbxl4	NM_001017429.2	F-box and leucine-rich repeat protein 4 (Fbxl4)	0.67
Fbxo9	NM_001033310.1	f-box protein 9 (Fbxo9), transcript variant 1	0.62
Fchsd2	NM_007747.2	FCH and double SH3 domains 2 (Fchsd2)	0.63
Fcmd	NM_009942		0.65
Fdps	NM_007748.2	farnesyl diphosphate synthetase (Fdps)	0.14
Fen1	NM_025628.2	flap structure specific endonuclease 1 (Fen1)	0.35
Fgf1	NM_053071.2	fibroblast growth factor 1 (Fgf1)	0.64
Fgf22	NM_009945.3	fibroblast growth factor 22 (Fgf22)	0.62
Fgfr1op2	NM_009187.1	FGFR1 oncogene partner 2 (Fgfr1op2)	0.60
Fh1	NM_013494.2	fumarate hydratase 1 (Fh1)	0.37

Fibp	NM_007756.2	fibroblast growth factor (acidic) intracellular binding protein (Fibp)	0.51
Fignl1	NM_001033851.1	fidgetin-like 1 (Fignl1)	0.27
Fkbp11	NM_029720.1	FK506 binding protein 11 (Fkbp11)	0.39
Fkbp1a	NM_018776.1	FK506 binding protein 1a (Fkbp1a)	0.44
Fkbp2	NM_019922.1	FK506 binding protein 2 (Fkbp2)	0.60
Fkbp4	NM_009964.1	FK506 binding protein 4 (Fkbp4)	0.48
Fkbp5	NM_009967.1	FK506 binding protein 5 (Fkbp5)	0.64
Fmnl2	NM_026444.3	formin-like 2 (Fmnl2)	0.39
Fndc3b	NM_144942	fibronectin type III domain containing 3B (Fndc3b)	0.35
Foxk2	NM_139117.2	forkhead box K2 (Foxk2)	0.56
Foxm1	NM_144901.2	forkhead box M1 (Foxm1)	0.45
Foxp3	NM_023565.2	forkhead box P3 (Foxp3)	0.63
Foxred1	NM_172753.3	FAD-dependent oxidoreductase domain containing 1 (Foxred1)	0.64
Fpgs	NM_007783.2	folylpolyglutamyl synthetase (Fpgs)	0.62
Frmd4a	NM_027874.2		0.40
Fscn1	NM_013767.5	fascin homolog 1, actin bundling protein (Strongylocentrotus purpuratus) (Fscn1)	0.32
Ftl1	NM_134002.1		0.63
Fubp1	AK031617		0.65
Fus	NM_009974.2		0.51
Fut8	NM_013884.2	fucosyltransferase 8 (Fut8)	0.35
Fxc1	NM_026493.3	fractured callus expressed transcript 1 (Fxc1)	0.42
Fxn	NM_007792.3	frataxin (Fxn)	0.51
Fxr1h	NM_009976.3		0.63
Fxr2	NM_024199.2	fragile X mental retardation, autosomal homolog 2 (Fxr2)	0.63
Fyn	NM_146012.1	Fyn proto-oncogene (Fyn)	0.57
Fzd1	NM_010217	frizzled homolog 1 (Drosophila) (Fzd1)	0.58
Fzr1	NM_009819.1	fizzy/cell division cycle 20 related 1 (Drosophila) (Fzr1)	0.56
G3bp1	NM_018761.2	Ras-GTPase-activating protein SH3-domain binding protein 1 (G3bp1)	0.44
G3bp2	NM_016748.1	GTPase activating protein (SH3 domain) binding protein 2 (G3bp2), transcript variant 1	0.60
Gaa	NM_001038492.1	glucosidase, alpha, acid (Gaa)	0.63
Gad1	NM_009982.3	glutamic acid decarboxylase 1 (Gad1)	0.53
Gadd45a	NM_007801.1	growth arrest and DNA-damage-inducible 45 alpha (Gadd45a)	0.41
Galnt1	NM_030249.3	UDP-N-acetyl-alpha-D-galactosamine:polypeptide N-acetylgalactosaminyltransferase 1 (Galnt1)	0.56
Galnt10	NM_198683.1	UDP-N-acetyl-alpha-D-galactosamine:polypeptide N-acetylgalactosaminyltransferase 10 (Galnt10)	0.53
Galnt2	NM_010160.2	UDP-N-acetyl-alpha-D-galactosamine:polypeptide N-acetylgalactosaminyltransferase 2 (Galnt2)	0.59
Ganab	NM_016716.4	alpha glucosidase 2 alpha neutral subunit (Ganab)	0.55
Gap43	NM_009142.3	growth associated protein 43 (Gap43)	0.31
Gapdh	NM_001025192.2	glyceraldehyde-3-phosphate dehydrogenase (Gapdh)	0.44
Gars	NM_008176.1	glycyl-tRNA synthetase (Gars)	0.47
Gart	NM_019932.2	phosphoribosylglycinamide formyltransferase (Gart)	0.65
Gas2l3	NM_009911.2	growth arrest-specific 2 like 3 (Gas2l3), transcript variant 2	0.27
Gas7	NM_025567.2	growth arrest specific 7 (Gas7)	0.61
Gatad1	NM_007808.4	GATA zinc finger domain containing 1 (Gatad1)	0.56
Gcap14	NM_180962.1	granule cell antiserum positive 14 (Gcap14), transcript variant 2	0.41
Gcap27	NM_009994.1		0.62
Gcat	NM_130882.1		0.37
Gcs1	NM_022434.1	glucosidase 1 (Gcs1)	0.41
Gdf15	NM_133969.2	growth differentiation factor 15 (Gdf15)	0.27
Gdpd1	NM_020010.2	glycerophosphodiester phosphodiesterase domain containing 1 (Gdpd1)	0.57
Gemin4	NM_007825.3	gem (nuclear organelle) associated protein 4 (Gemin4)	0.56
Gfm1	AK050914	G elongation factor, mitochondrial 1 (Gfm1)	0.52
Gga2	AK083512	golgi associated, gamma adaptin ear containing, ARF binding protein 2 (Gga2)	0.65
Gins2	NM_177640.4	GINS complex subunit 2 (Psf2 homolog) (Gins2)	0.53
Gipc1	NM_053078.3	GIPC PDZ domain containing family, member 1 (Gipc1)	0.52
Glb1	NM_001013379.2	galactosidase, beta 1 (Glb1)	0.50
Glg1	NM_026065.2		0.59
Glrx1	NM_028027.2		0.66
Glrx2	NM_026579.2	glutaredoxin 2 (thioltransferase) (Glrx2), transcript variant 1	0.59
Glrx3	NM_145422.3	glutaredoxin 3 (Glrx3)	0.61
Glrx5	NM_029758.3	glutaredoxin 5 homolog (S. cerevisiae) (Glrx5)	0.53
Glt25d1	NM_198023	glycosyltransferase 25 domain containing 1 (Glt25d1)	0.39
Gm444		PREDICTED: gene model 444, (NCBI) (Gm444)	0.62
Gm561	NM_134100.2	gene model 561, (NCBI) (Gm561)	0.41
Gmds	NM_138583.1	GDP-mannose 4, 6-dehydratase (Gmds)	0.57
Gmpr2	NM_033075.2	guanosine monophosphate reductase 2 (Gmpr2)	0.61
Gmps	NM_080837.1	guanine monphosphate synthetase (Gmps)	0.61

Gna11	NM_177474.4		0.63
Gnai1		guanine nucleotide binding protein (G protein), alpha inhibiting 1 (Gnai1)	0.50
Gnaq	AK051822	guanine nucleotide binding protein, alpha q polypeptide (Gnaq)	0.66
Gnb1	NM_026828.2	guanine nucleotide binding protein (G protein), beta 1 (Gnb1)	0.49
Gnb4	NM_026412.1	guanine nucleotide binding protein (G protein), beta 4 (Gnb4)	0.57
Gne	NM_177617.2		0.66
Gng12	NM_001081022.1		0.39
Gng2	NM_026821.4	guanine nucleotide binding protein (G protein), gamma 2 (Gng2), transcript variant 2	0.64
Gnl3		guanine nucleotide binding protein-like 3 (nucleolar) (Gnl3), transcript variant 2	0.36
Gnpda1	NM_001081232.1	glucosamine-6-phosphate deaminase 1 (Gnpda1)	0.48
Golga3	NM_207274.1	golgi autoantigen, golgin subfamily a, 3 (Golga3)	0.66
Golim4	NM_138587.3	golgi integral membrane protein 4 (Golim4)	0.60
Golm1	NM_172681.2	golgi membrane protein 1 (Golm1), transcript variant 2	0.21
Gorasp2		golgi reassembly stacking protein 2 (Gorasp2)	0.49
Got2	NM_028181	glutamate oxaloacetate transaminase 2, mitochondrial (Got2), nuclear gene encoding mitochondrial protein	0.41
Gpd1	NM_133718	glycerol-3-phosphate dehydrogenase 1 (soluble) (Gpd1)	0.58
Gpd2	NM_010015.1	glycerol phosphate dehydrogenase 2, mitochondrial (Gpd2), nuclear gene encoding mitochondrial protein	0.57
Gpi1	NM_022994.2	glucose phosphate isomerase 1 (Gpi1)	0.47
Gpnmb	NM_134062.1	glycoprotein (transmembrane) nmb (Gpnmb)	0.40
Gpr162	NM_010045.2	G protein-coupled receptor 162 (Gpr162)	0.54
Gpsm1	AK010657	G-protein signalling modulator 1 (AGS3-like, C. elegans) (Gpsm1)	0.58
Gpsn2	NM_013726.2	glycoprotein, synaptic 2 (Gpsn2)	0.62
Grcc10	NM_010022.1	gene rich cluster, C10 gene (Grcc10)	0.54
Gria3	XM_147966.3	glutamate receptor, ionotropic, AMPA3 (alpha 3) (Gria3)	0.60
Grip1	NM_019978.2		0.56
Grm5	NM_027030.2	glutamate receptor, metabotropic 5 (Grm5)	0.16
Grwd1	NM_029775.2	glutamate-rich WD repeat containing 1 (Grwd1)	0.59
Gsg2	NM_028119.2	germ cell-specific gene 2 (Gsg2)	0.56
Gspt1	NM_027442.4	G1 to S phase transition 1 (Gspt1)	0.44
Gstm5	NM_007838.2	glutathione S-transferase, mu 5 (Gstm5)	0.56
Gstp1	NM_007584.2	glutathione S-transferase, pi 1 (Gstp1)	0.53
Gstt1	NM_134040.1	glutathione S-transferase, theta 1 (Gstt1)	0.52
Gtf2e2	XM_128714.3	general transcription factor II E, polypeptide 2 (beta subunit) (Gtf2e2)	0.58
Gtf2f2	NM_017397.2	general transcription factor IIF, polypeptide 2 (Gtf2f2)	0.63
Gtf2h2	NM_019553.2	general transcription factor II H, polypeptide 2 (Gtf2h2)	0.65
Gtf3a	NM_013932.3	general transcription factor III A (Gtf3a)	0.61
Gtf3c1	NM_008715	general transcription factor III C 1 (Gtf3c1)	0.65
Gtpbp3	NM_153065.3	GTP binding protein 3 (Gtpbp3), nuclear gene encoding mitochondrial protein	0.41
Gtpbp4	NM_010028.2	GTP binding protein 4 (Gtpbp4)	0.52
Gtse1	NM_012008.1	G two S phase expressed protein 1 (Gtse1)	0.47
Gusb	NM_028041.2	glucuronidase, beta (Gusb)	0.57
H1fx	NM_007841.3	H1 histone family, member X (H1fx)	0.65
H2afx	NM_026794.2	H2A histone family, member X (H2afx)	0.26
H2afz	NM_025900.1	H2A histone family, member Z (H2afz)	0.21
Hat1	NM_172477.3	histone aminotransferase 1 (Hat1)	0.42
Hbegf		heparin-binding EGF-like growth factor (Hbegf)	0.42
Hccs	NM_177914.2	holocytochrome c synthetase (Hccs)	0.61
Hdac2	NM_138306.1		0.44
Hdgf	XM_131538.1	hepatoma-derived growth factor (Hdgf)	0.65
Heatr1	NM_007856.2	HEAT repeat containing 1 (Heatr1)	0.52
Hectd1	NM_007857.3	PREDICTED: HECT domain containing 1 (Hectd1)	0.56
Hectd2	NM_001039514.1	HECT domain containing 2 (Hectd2)	0.29
Herc4	NM_026819.2	hect domain and RLD 4 (Herc4)	0.66
Hes1	NM_001033326.2	hairy and enhancer of split 1 (Drosophila) (Hes1)	0.55
Hes6	NM_007839.2	hairy and enhancer of split 6 (Drosophila) (Hes6)	0.66
Hif1a	NM_133347.1	hypoxia inducible factor 1, alpha subunit (Hif1a)	0.64
Hint1	NM_133941.2	histidine triad nucleotide binding protein 1 (Hint1)	0.54
Hip1	NM_011805.2	huntingtin interacting protein 1 (Hip1)	0.12
HIP-1	NM_145217.2		0.52
Hip2			0.53
Hirip3	NM_178118	HIRA interacting protein 3 (Hirip3)	0.45
Hist1h2ad	NM_177639.5	histone cluster 1, H2ad (Hist1h2ad)	0.09
Hist1h2af	NM_144553.1	histone cluster 1, H2af (Hist1h2af)	0.08
Hist1h2ag	NM_030225	histone cluster 1, H2ag (Hist1h2ag)	0.26
Hist1h2ah	NM_032418	histone cluster 1, H2ah (Hist1h2ah)	0.13
Hist1h2ai	NM_010060.2	histone cluster 1, H2ai (Hist1h2ai)	0.08
Hist1h2ak	XM_148071.1	histone cluster 1, H2ak (Hist1h2ak)	0.15

Hist1h2an	NM_153527.2	histone cluster 1, H2an (Hist1h2an)	0.08
Hist1h2ao	NM_178055.3	histone cluster 1, H2ao (Hist1h2ao)	0.09
Hist2h2ab	NM_013760.3	histone cluster 2, H2ab (Hist2h2ab)	0.28
Hist2h2ac	NM_001026211.1	histone cluster 2, H2ac (Hist2h2ac)	0.17
Hmga1	NM_009584.3	high mobility group AT-hook 1 (Hmga1), transcript variant 1	0.36
Hmgb2	NM_138664.2	high mobility group box 2 (Hmgb2)	0.31
Hmgcl	NM_175223.2	3-hydroxy-3-methylglutaryl-Coenzyme A lyase (Hmgcl)	0.60
Hmgcr	NM_152915.1	3-hydroxy-3-methylglutaryl-Coenzyme A reductase (Hmgcr)	0.47
Hmgn2	NM_010066.3	high mobility group nucleosomal binding domain 2 (Hmgn2)	0.62
Hn1	NM_001033420.2	hematological and neurological expressed sequence 1 (Hn1)	0.49
Hn1l	XM_129913.4	hematological and neurological expressed 1-like (Hn1l)	0.52
Hnrnpa2b1	NM_015761.2	heterogeneous nuclear ribonucleoprotein A2/B1 (Hnrnpa2b1), transcript variant 2	0.36
Hnrnpab	XM_130951.1	heterogeneous nuclear ribonucleoprotein A/B (Hnrnpab), transcript variant 1	0.41
Hnrnpf	NM_133803.1	heterogeneous nuclear ribonucleoprotein F (Hnrnpf)	0.64
Hnrnph1	NM_024428.3	heterogeneous nuclear ribonucleoprotein H1 (Hnrnph1)	0.50
Hnrnpk	NM_009468.3	heterogeneous nuclear ribonucleoprotein K (Hnrnpk)	0.64
Hnrpa1	NM_011993.2		0.24
Hnrpab	NM_023047.2		0.64
Hnrpdl	NM_007879.1	heterogeneous nuclear ribonucleoprotein D-like (Hnrpdl)	0.63
Hnrpl	NM_010078.2	heterogeneous nuclear ribonucleoprotein L (Hnrpl)	0.51
Hnrpll	NM_019466.2	heterogeneous nuclear ribonucleoprotein L-like (Hnrpll)	0.52
Hnrpm	NM_172442.2	heterogeneous nuclear ribonucleoprotein M (Hnrpm)	0.60
Homer1	NM_023136	homer homolog 1 (Drosophila) (Homer1), transcript variant d	0.53
Hpcal1	NM_013642.2	hippocalcin-like 1 (Hpcal1)	0.51
Hprt1	NM_176933.4	hypoxanthine guanine phosphoribosyl transferase 1 (Hprt1)	0.27
Hras1	NM_026268.2		0.60
Hrmt1l2	NM_153459.2		0.23
Hs2st1	NM_172256.1	heparan sulfate 2-O-sulfotransferase 1 (Hs2st1)	0.62
Hs3st3b1	NM_019682.2	heparan sulfate (glucosamine) 3-O-sulfotransferase 3B1 (Hs3st3b1)	0.61
Hs6st2	NM_029297.1	heparan sulfate 6-O-sulfotransferase 2 (Hs6st2), transcript variant 2	0.55
Hsd17b12	NM_021469.2	hydroxysteroid (17-beta) dehydrogenase 12 (Hsd17b12)	0.40
Hsd17b7	NM_175553	hydroxysteroid (17-beta) dehydrogenase 7 (Hsd17b7)	0.60
Hsp90ab1	AK087162	heat shock protein 90kDa alpha (cytosolic), class B member 1 (Hsp90ab1)	0.51
Hsp90b1	NM_001081756.1	heat shock protein 90, beta (Grp94), member 1 (Hsp90b1)	0.40
Hspa8	NM_001039556.2	heat shock protein 8 (Hspa8)	0.63
Hspa9	NM_178791.4	heat shock protein 9 (Hspa9)	0.60
Hspd1	XM_131404.3	heat shock protein 1 (chaperonin) (Hspd1)	0.36
Hspe1	NM_001015681.1	heat shock protein 1 (chaperonin 10) (Hspe1)	0.41
Hspg2	AK054036		0.64
Htra1	NM_007891.3	HtrA serine peptidase 1 (Htra1)	0.66
Htra2	NM_177733.2	HtrA serine peptidase 2 (Htra2), nuclear gene encoding mitochondrial protein	0.65
Hyal2	NM_033270.1	hyaluronoglucosaminidase 2 (Hyal2)	0.50
Iars	NM_175528	isoleucine-tRNA synthetase (Iars)	0.47
Iars2	NM_173386.3	isoleucine-tRNA synthetase 2, mitochondrial (Iars2)	0.65
Ibtk	NM_173749		0.65
Ick	NM_178668.3	intestinal cell kinase (Ick)	0.61
Ide	AK089103	insulin degrading enzyme (Ide)	0.59
Idh3a	AK089133	isocitrate dehydrogenase 3 (NAD+) alpha (Idh3a), nuclear gene encoding mitochondrial protein	0.46
Ier3	NM_026932.3	immediate early response 3 (Ier3)	0.18
Ier5l	NM_007898.2	immediate early response 5-like (Ier5l)	0.35
Igf2r	NM_199307.1	insulin-like growth factor 2 receptor (Igf2r)	0.54
Igfbp2	NM_016772.1	insulin-like growth factor binding protein 2 (Igfbp2)	0.06
Igfbp3	NM_026728.1	insulin-like growth factor binding protein 3 (Igfbp3)	0.05
Igfbp4	NM_007899.1	insulin-like growth factor binding protein 4 (Igfbp4)	0.08
Ilf2	NM_021519.1	interleukin enhancer binding factor 2 (Ilf2)	0.55
Ilvbl	NM_010332.2	ilvB (bacterial acetolactate synthase)-like (Ilvbl)	0.63
Immt	NM_007904.3	inner membrane protein, mitochondrial (Immt), nuclear gene encoding mitochondrial protein	0.43
Imp3	NM_010106.2	IMP3, U3 small nucleolar ribonucleoprotein, homolog (yeast) (Imp3)	0.45
Impa2	NM_018796.2	inositol (myo)-1(or 4)-monophosphatase 2 (Impa2)	0.59
Impad1	NM_025380.2	inositol monophosphatase domain containing 1 (Impad1)	0.47
Impdh2	NM_026007.4		0.44
Incenp	NM_010110.2	inner centromere protein (Incenp)	0.37
Inpp5f	NM_010112.3	inositol polyphosphate-5-phosphatase F (Inpp5f)	0.47
Insig1	XM_911906.3	insulin induced gene 1 (Insig1)	0.34
Ints7	NM_177689.3	integrator complex subunit 7 (Ints7)	0.64
Ipo4	XM_905111.2	importin 4 (Ipo4)	0.62
Ipo5	XM_145024.8	importin 5 (Ipo5)	0.46
Ipo9	NM_001018086.2	importin 9 (Ipo9)	0.53

Iqgap1	XM_921134.3		0.46
Iqgap3	XM_913807.2	IQ motif containing GTPase activating protein 3 (Iqgap3)	0.51
Isca1	XM_905000.3	iron-sulfur cluster assembly 1 homolog (S. cerevisiae) (Isca1)	0.66
Isy1	XM_890293.3	ISY1 splicing factor homolog (S. cerevisiae) (Isy1)	0.54
Itgav	XM_484165.5	integrin alpha V (Itgav)	0.61
Itgb1	XM_484210.5	integrin beta 1 (fibronectin receptor beta) (Itgb1)	0.60
Itpr2	NM_001034882.2	inositol 1,4,5-triphosphate receptor 2 (Itpr2), transcript variant 1	0.39
Jagn1	XM_484670.5	jagunal homolog 1 (Drosophila) (Jagn1)	0.62
Jam2	NM_001025388.1	junction adhesion molecule 2 (Jam2)	0.62
Jmjd6	NM_001081233.1	jumonji domain containing 6 (Jmjd6)	0.49
Josd2	XM_486222.5	Josephin domain containing 2 (Josd2)	0.56
Jun	NR_002883.1	Jun oncogene (Jun)	0.60
Kars	NR_002889.2	lysyl-tRNA synthetase (Kars)	0.54
Kbtbd2	XM_620762.4	kelch repeat and BTB (POZ) domain containing 2 (Kbtbd2)	0.64
Kcnc1	XM_917756.3	potassium voltage gated channel, Shaw-related subfamily, member 1 (Kcnc1)	0.33
Kcnc4	NM_001081297.1	potassium voltage gated channel, Shaw-related subfamily, member 4 (Kcnc4)	0.38
Kcnip3	XM_888318.3	Kv channel interacting protein 3, calsenilin (Kcnip3)	0.42
Kcnk13	XM_889835.2	potassium channel, subfamily K, member 13 (Kcnk13)	0.66
Kctd12	NR_003363.1	potassium channel tetramerisation domain containing 12 (Kctd12)	0.44
Kctd20	XM_917009.3	potassium channel tetramerisation domain containing 20 (Kctd20)	0.63
Kdelc1	XM_908326.3	KDEL (Lys-Asp-Glu-Leu) containing 1 (Kdelc1)	0.65
Kdelr2	XM_894495.2	KDEL (Lys-Asp-Glu-Leu) endoplasmic reticulum protein retention receptor 2 (Kdelr2)	0.43
Khdrbs1	XM_001473902.1	KH domain containing, RNA binding, signal transduction associated 1 (Khdrbs1)	0.50
Kif11	XM_978542.1	kinesin family member 11 (Kif11)	0.40
Kif15	XR_031485.1	kinesin family member 15 (Kif15)	0.37
Kif18a	XM_917238.2	kinesin family member 18A (Kif18a)	0.37
Kif20b	XM_912043.2	kinesin family member 20B (Kif20b)	0.32
Kif22	NM_001081746.1	kinesin family member 22 (Kif22)	0.16
Kif23	XM_984280.2		0.11
Kif2c	XM_984926.2	kinesin family member 2C (Kif2c)	0.26
Kif3c	XR_002058.1	kinesin family member 3C (Kif3c)	0.31
Kif4	XR_035278.1	kinesin family member 4 (Kif4)	0.31
Kifc1	NM_001081036.1		0.50
Kirrel3	XM_001005405.1	kin of IRRE like 3 (Drosophila) (Kirrel3)	0.44
Kitl	XR_002259.2	kit ligand (Kitl)	0.56
Klf6	NM_007913.5	Kruppel-like factor 6 (Klf6)	0.65
Klf7	NM_133838.4		0.62
Klhdc2	NM_010120.4	kelch domain containing 2 (Klhdc2)	0.55
Kntc1	NM_027236.2	kinetochore associated 1 (Kntc1)	0.38
Kpna1	XM_131572.4	karyopherin (importin) alpha 1 (Kpna1)	0.64
Kpna2	NM_026030.2	karyopherin (importin) alpha 2 (Kpna2)	0.56
Kpnb1	NM_012010.3	karyopherin (importin) beta 1 (Kpnb1)	0.53
Kras	NM_010123.3	v-Ki-ras2 Kirsten rat sarcoma viral oncogene homolog (Kras)	0.65
Krtcap2	NM_133916.2	keratinocyte associated protein 2 (Krtcap2)	0.53
Lama5	NM_018749.2	laminin, alpha 5 (Lama5)	0.43
Lamc1	NM_145139.2	laminin, gamma 1 (Lamc1)	0.59
Lass2	NM_025344.1	LAG1 homolog, ceramide synthase 2 (Lass2)	0.54
Lass5	NM_016876.3	LAG1 homolog, ceramide synthase 5 (Lass5)	0.59
Lbh	NM_018799.1	limb-bud and heart (Lbh)	0.38
Lbr	NM_028659.2		0.23
Ldha	NM_016876.2	lactate dehydrogenase A (Ldha)	0.33
Leo1	NM_025344	Leo1, Paf1/RNA polymerase II complex component, homolog (S. cerevisiae) (Le	0.58
Lgals1	NM_144958.2		0.17
Lig1	NM_138669.1	ligase I, DNA, ATP-dependent (Lig1), transcript variant 2	0.27
Lig3	NM_007918.3		0.60
Limd1	NM_023314.2	LIM domains containing 1 (Limd1)	0.61
Lin54	NM_001005331.1	lin-54 homolog (C. elegans) (Lin54)	0.49
Lip1	NM_173363.4		0.52
Liph	NM_181582.3	lipase, member H (Liph), transcript variant 2	0.58
Litaf	NM_010579.2	LPS-induced TN factor (Litaf)	0.36
Lman1	NM_010485.3	lectin, mannose-binding, 1 (Lman1)	0.59
Lmnb1	NM_205536.1	lamin B1 (Lmnb1)	0.15
Lmo2	NM_198093.2	LIM domain only 2 (Lmo2)	0.32
LOC100039346	NM_019422.2	PREDICTED: hypothetical protein LOC100039346 (LOC100039346)	0.33
LOC100039532	NM_148941.1	PREDICTED: similar to ribosomal protein L35a (LOC100039532)	0.52
LOC100039571	NM_134255.2	PREDICTED: similar to ribosomal protein L23a (LOC100039571)	0.35
LOC100039751	NM_130450.2	PREDICTED: similar to 40S ribosomal protein S12 (LOC100039751), misc RNA	0.37
LOC100039786	NM_013536.1	PREDICTED: similar to Ywhaq protein (LOC100039786)	0.58
LOC100040573	NM_007929.2	PREDICTED: similar to putative transcription factor ZNF131, transcript variant 1 (LOC100040573)	0.59

LOC100040592	NM_028013.2	PREDICTED: similar to Hmgcs1 protein, transcript variant 1 (LOC100040592)	0.38
LOC100040605	NM_023119.1	PREDICTED: similar to ribosomal protein L13 (LOC100040605)	0.44
LOC100041500	NM_032003.1	PREDICTED: similar to LSM7 homolog, U6 small nuclear RNA associated (LOC100041500)	0.58
LOC100041835	NM_009849.1	PREDICTED: similar to H+ ATP synthase (LOC100041835)	0.41
LOC100041864	NM_026174.2	PREDICTED: similar to Rpl11 protein (LOC100041864)	0.55
LOC100042405	NM_001026214.1	PREDICTED: similar to high mobility group nucleosomal binding domain 2 (LOC100042405)	0.45
LOC100042427	NM_173447.2	PREDICTED: similar to Glyceraldehyde-3-phosphate dehydrogenase (GAPDH), transcript variant 4 (LOC100042427)	0.39
LOC100042777	NM_007940.3	PREDICTED: similar to human protein homologous to DROER protein (LOC100042777)	0.65
LOC100042970	NM_010148	PREDICTED: similar to Kifc1 protein (LOC100042970)	0.42
LOC100043192	NM_007948.1	PREDICTED: similar to ribosomal protein L31 (LOC100043192)	0.50
LOC100043209	NM_026170.3	PREDICTED: similar to ribosomal protein L15 (LOC100043209)	0.54
LOC100043257	NM_026168.3	PREDICTED: similar to RNA binding motif protein 3 (LOC100043257)	0.48
LOC100043391	NM_007951.1	PREDICTED: similar to QM protein (LOC100043391)	0.48
LOC100043527	NM_145502.2	PREDICTED: similar to ribosomal protein S28 (LOC100043527)	0.49
LOC100043555	NM_001081213.1	PREDICTED: similar to Aak1 protein (LOC100043555)	0.52
LOC100043671	NM_026129.2	PREDICTED: hypothetical protein LOC100043671 (LOC100043671)	0.51
LOC100043675	NM_133753.1	PREDICTED: similar to Translocase of inner mitochondrial membrane 23 homolog (yeast) (LOC100043675)	0.54
LOC100043796	NM_028039.1	PREDICTED: similar to macrophage migration inhibitory factor, transcript variant 1 (LOC100043796)	0.53
LOC100043822	NM_001014976.1	PREDICTED: hypothetical protein LOC100043822 (LOC100043822)	0.63
LOC100043906	NM_007953.2	PREDICTED: similar to nuclear pore-targeting complex component of 58 kDa, transcript variant 1 (LOC100043906)	0.36
LOC100044322	NM_011934.3	PREDICTED: similar to UDP-glucose ceramide glucosyltransferase-like 1 (LOC100044322)	0.49
LOC100044468	NM_144866.3	PREDICTED: similar to nemo-like kinase (LOC100044468)	0.58
LOC100044475	NM_001038642.1	PREDICTED: similar to SH2/SH3 adaptor protein (LOC100044475)	0.66
LOC100044696	NM_011809.2	PREDICTED: similar to cysteine-rich PAK1inhibitor (LOC100044696)	0.53
LOC100044829	NM_007960.3	PREDICTED: similar to Fibrillarin, transcript variant 1 (LOC100044829)	0.35
LOC100044948	NM_008815.2	PREDICTED: similar to macrophage migration inhibitory factor (LOC100044948)	0.45
LOC100045040	NM_023794.2	PREDICTED: similar to ING1 protein (LOC100045040), misc RNA.	0.66
LOC100045300	NM_025644.3	PREDICTED: similar to Dr1 associated protein 1 (negative cofactor 2 alpha) (LOC100045300)	0.61
LOC100045312	NM_144886.1	PREDICTED: similar to ribosomal protein (LOC100045312)	0.35
LOC100045343	NM_028274.2	PREDICTED: similar to CDNA sequence BC046404 (LOC100045343), misc RN/	0.64
LOC100045542	NM_001081188.1	PREDICTED: similar to FERMRhoGEF (Arhgef) and pleckstrin domain protein 1 (LOC100045542)	0.63
LOC100045668	NM_027148.2	PREDICTED: similar to Rpl17 protein (LOC100045668)	0.49
LOC100045677	NM_010162.2	PREDICTED: similar to DNA replication licensing factor MCM3 (DNA polymerase alpha holoenzyme-associated protein P1) (P1-MCM3) (LOC100045677), misc RNA.	0.49
LOC100045697	NM_018788.2	PREDICTED: similar to ornithine decarboxylase antizyme (LOC100045697), misc RNA.	0.65
LOC100045737	NM_007971.1	PREDICTED: hypothetical protein LOC100045737 (LOC100045737), misc RNA.	0.62
LOC100045780	NM_010169.3	PREDICTED: similar to metalloprotease-disintegrin meltrin beta (LOC100045780)	0.18
LOC100045887	NM_198605.1	PREDICTED: similar to PTB-associated splicing factor (LOC100045887)	0.51
LOC100045967	AK089303	PREDICTED: hypothetical protein LOC100045967 (LOC100045967), misc RNA.	0.34
LOC100045981	AK089567	PREDICTED: similar to synaptotagmin XI (LOC100045981)	0.65
LOC100045999	NM_010173.3	PREDICTED: similar to RAN, member RAS oncogene family (LOC100045999)	0.42
LOC100046039	NM_010634.2	PREDICTED: similar to histone deacetylase HD1 (LOC100046039)	0.64
LOC100046343	NM_021272.2	PREDICTED: similar to CCR4-NOT transcription complex, subunit 6 (LOC100046343)	0.49
LOC100046650	NM_021890.3	PREDICTED: similar to PRELI domain containing 1 (LOC100046650)	0.53
LOC100046744	NM_007983.2	PREDICTED: similar to Serine/arginine repetitive matrix protein 2 (LOC100046744)	0.59
LOC100046853	NM_010176.2	PREDICTED: similar to heterogeneous nuclear ribonucleoprotein K (LOC100046853)	0.26
LOC100046855	NM_023480.2	PREDICTED: similar to BKLF (LOC100046855)	0.44
LOC100046895	NM_028443.2	PREDICTED: similar to Quaking protein (LOC100046895)	0.66
LOC100047155	NM_153560.4	PREDICTED: similar to Small nuclear ribonucleoprotein polypeptide A (LOC100047155)	0.44
LOC100047167	NM_145421.1	PREDICTED: similar to mKIAA0990 protein (LOC100047167)	0.36
LOC100047184	NM_028666.2	PREDICTED: similar to proteasome alpha7/C8 subunit (LOC100047184)	0.65

LOC100047261	NM_001033268.2	PREDICTED: similar to spermidine/spermine N1-acetyltransferase (LOC100047261), misc RNA.	0.63
LOC100047268	NM_028617.2	PREDICTED: similar to Immediate early response 5-like (LOC100047268)	0.26
LOC100047353	NM_133778.2	PREDICTED: similar to myocardial vascular inhibition factor (LOC100047353)	0.63
LOC100047579	NM_025459.2	PREDICTED: similar to transmembrane protein 20 (LOC100047579)	0.66
LOC100047615	NM_001081161.1	PREDICTED: similar to 40S ribosomal protein S17 (LOC100047615), misc RNA.	0.57
LOC100047619	NM_145410.3	PREDICTED: similar to solute carrier family 7 (cationic amino acid transporter, y+ system), member 5 (LOC100047619), misc RNA.	0.32
LOC100047634	NM_001081428.1	PREDICTED: similar to Eukaryotic translation initiation factor 2 alpha kinase 3 (LOC100047634), misc RNA.	0.63
LOC100047651	NM_153782.1	PREDICTED: similar to FOG (LOC100047651)	0.56
LOC100047712	NM_029007.2	PREDICTED: similar to coiled-coil domain containing 101 (LOC100047712)	0.63
LOC100047759	NM_001033244.2	PREDICTED: similar to acidic ribosomal phosphoprotein P1, transcript variant 1 (LOC100047759)	0.43
LOC100047762	XM_986338.2	PREDICTED: similar to Aspartate aminotransferase, cytoplasmic (Transaminase A) (Glutamate oxaloacetate transaminase 1) (LOC100047762)	0.59
LOC100047827	NM_025850.2	PREDICTED: similar to Hmgn2 protein (LOC100047827)	0.42
LOC100047888	NM_011811.2	PREDICTED: similar to Neurocan (LOC100047888)	0.42
LOC100047934	NM_007987.1	PREDICTED: hypothetical protein LOC100047934 (LOC100047934)	0.45
LOC100047935	NM_023229.2	PREDICTED: similar to ribosomal protein L5 (LOC100047935), misc RNA.	0.52
LOC100047998	NM_133754.3	PREDICTED: similar to ribosomal protein L37a (LOC100047998)	0.43
LOC100048187	NM_010180.1	PREDICTED: similar to ribosomal protein S27 (metallopanstimulin 1) (LOC100048187)	0.59
LOC100048413	NM_001081437.1	PREDICTED: similar to Myeloid leukemia factor 2 (LOC100048413)	0.56
LOC100048445	NM_007993	PREDICTED: similar to fau (LOC100048445)	0.50
LOC100048480	NM_001005866.1	PREDICTED: similar to ubiquinol-cytochrome c reductase binding protein (LOC100048480)	0.59
LOC100048483	NM_133940.1	PREDICTED: similar to cytochrome c oxidase subunit VIII (LOC100048483)	0.43
LOC100048508	NM_172988.1	PREDICTED: similar to Rpl36 protein, transcript variant 1 (LOC100048508)	0.43
LOC100048613	NM_176848.1	PREDICTED: similar to cytochrome c oxidase, subunit VIIc, transcript variant 1 (LOC100048613)	0.47
LOC100048645	NM_145564.3	PREDICTED: similar to cysteine and glycine-rich protein 2 binding protein (LOC100048645)	0.59
LOC100048803	NM_025785.2	PREDICTED: hypothetical protein LOC100048803 (LOC100048803)	0.54
LOC212399	NM_026346.1		0.39
LOC216443	NM_025386.2		0.59
LOC219049	NM_001001160.2		0.50
LOC219106	NM_173401.2		0.57
LOC219145	NM_023605.2		0.40
LOC225058	NM_010189.1		0.47
LOC225134	NM_199012.1	PREDICTED: similar to ribosomal protein S4, X-linked (LOC225134), misc RNA.	0.42
LOC226416	NM_139309		0.47
LOC229810	NM_134469.3		0.63
LOC230253	NM_007999.3		0.25
LOC230896	XM_001480162.1		0.55
LOC231368	NM_183171.1	PREDICTED: similar to 60S acidic ribosomal protein P0 (L10E) (LOC231368)	0.40
LOC232606	NM_199448.1		0.59
LOC232887	NM_010197.3		0.60
LOC232890	NM_023304.1		0.47
LOC236831	NM_026218.2	PREDICTED: similar to 60S ribosomal protein L7a (LOC236831)	0.43
LOC237512	NM_010209.1		0.40
LOC237877	NM_001077361.1		0.60
LOC238943	NM_021438.2		0.62
LOC241621	NM_021891.2	PREDICTED: similar to ribosomal protein L27a-like (LOC241621)	0.29
LOC245668	NM_024169.3		0.45
LOC245892	NM_008019.2		0.16
LOC268569	NM_008020.2		0.20
LOC268700	NM_010219.3		0.39
LOC269251	NM_010220.2		0.33
LOC270037	NM_201518.1		0.33
LOC271490	NM_019445.1		0.49
LOC271505	NM_172409.2		0.54
LOC272681	NM_022014.2		0.48
LOC277856	NM_173182.1		0.56
LOC280205	NM_010234.2		0.56
LOC329076	NM_008240.2		0.41
LOC329750	NM_001080932.1		0.55
LOC331102	NM_008021.4		0.51
LOC331507	NM_054039.1		0.63
LOC380692	NM_172291.1		0.26

LOC380707	NM_010236.1		0.55
LOC380927	NM_172475		0.57
LOC380930	NM_007984.2		0.48
LOC381046	NM_008047.4		0.37
LOC381114	NM_010240.1		0.27
LOC381140	NM_057172.1		0.50
LOC381215	NM_139149.1		0.49
LOC381230	NM_016893.2		0.54
LOC381330	NM_019502.2		0.42
LOC381365	NM_008044.1		0.38
LOC381398	AF124385		0.37
LOC381448	NM_011814.2		0.36
LOC381561	NM_148925.1		0.49
LOC381649	NM_008054.1		0.46
LOC381799	NM_021457.2		0.64
LOC381808	NM_008056		0.58
LOC381891	NM_019757.1		0.60
LOC381940	NM_013716.2		0.65
LOC381999	NM_011816.3		0.44
LOC382061	NM_008064.2		0.43
LOC382230	NM_021356.2		0.52
LOC382885	NM_020590.4		0.51
LOC383010	NM_008069.4		0.63
LOC383099	NM_008077.4		0.53
LOC383308	NM_007836.1		0.34
LOC383330	NM_013814.2		0.62
LOC383897	NM_134189.2		0.54
LOC383942	NM_139272.2		0.64
LOC384104	NM_001081421.1		0.52
LOC384206	NM_008060.1		0.58
LOC384727	NM_172672.2		0.55
LOC385699	NM_008083.2		0.62
LOC385905	NM_008084.2	PREDICTED: similar to proteasome alpha7/C8 subunit (LOC385905), misc RNA	0.58
LOC433476	NM_178888.4	PREDICTED: similar to ribosomal protein L27a (LOC433476)	0.28
LOC433546	NM_180678.3	PREDICTED: similar to 60S ribosomal protein L9 (LOC433546)	0.59
LOC433745	NM_010256.2	PREDICTED: similar to ribosomal protein L3 (LOC433745)	0.50
LOC433749	NM_001079876.1	PREDICTED: similar to Rho family GTPase RhoA (LOC433749)	0.66
LOC433943	NM_008088.1	PREDICTED: similar to Glutathione S-transferase Mu 1 (GST class-mu 1) (Glutathione S-transferase GT8.7) (pmGT10) (GST 1-1) (LOC433943), misc RNA.	0.66
LOC433955	NM_026033.1	PREDICTED: similar to HSPC008 (LOC433955), misc RNA.	0.64
LOC545056	XM_289726.2	ubiquitin-conjugating enzyme E2, J2 homolog pseudogene (LOC545056) on chromosome 14.	0.53
LOC545369	NM_010260.1	PREDICTED: similar to ribosomal protein L4 (LOC545369), misc RNA.	0.60
LOC545472	NM_145523.3	PREDICTED: similar to BTB (POZ) domain containing 1 (LOC545472)	0.60
LOC546015	NM_028407.3	PREDICTED: similar to ribosomal protein S9 (LOC546015), misc RNA.	0.65
LOC620678		PREDICTED: similar to 60S ribosomal protein L7 (LOC620678), misc RNA.	0.53
LOC622655	NM_013847	PREDICTED: similar to hCG31107 (LOC622655), misc RNA.	0.34
LOC622994	NM_133219.1	PREDICTED: hypothetical LOC622994 (LOC622994), misc RNA.	0.45
LOC623006	NM_020619.2	PREDICTED: similar to retinoblastoma-binding protein mRbAp46 (LOC623006), misc RNA.	0.58
LOC623466	NM_144891.1	PREDICTED: similar to MGC27348 protein (LOC623466), misc RNA.	0.42
LOC623568	NM_011819.1	PREDICTED: similar to ribosomal protein S20 (LOC623568)	0.41
LOC624662	NM_025638.1	PREDICTED: similar to MGC69457 protein (LOC624662), misc RNA.	0.65
LOC630729	NM_023608.2	PREDICTED: similar to glutathione reductase 1 (LOC630729)	0.57
LOC630936	NM_177367.2	PREDICTED: similar to nuclease sensitive element binding protein 1 (LOC630936), misc RNA.	0.31
LOC632022	NM_010277	PREDICTED: similar to 40S ribosomal protein S18 (LOC632022), misc RNA.	0.55
LOC632230	NM_138591.1	PREDICTED: similar to Ribosomal protein L5 (LOC632230), misc RNA.	0.46
LOC632667	NM_028758.2	PREDICTED: similar to PNG protein (LOC632667)	0.39
LOC632684	NM_027544.1	PREDICTED: hypothetical LOC632684 (LOC632684), misc RNA.	0.58
LOC633016	NM_178856.1	PREDICTED: similar to Chromobox homolog 3 (HP1 gamma homolog, Drosophila), transcript variant 2 (LOC633016)	0.54
LOC634015	NM_018771.3	PREDICTED: similar to Proteasome subunit beta type 3 (Proteasome theta chain) (Proteasome chain 13) (Proteasome component C10-II) (LOC634015)	0.64
LOC635470	NM_010288.2	PREDICTED: similar to ribosomal protein L27a-like (LOC635470)	0.33
LOC638892	NM_009752.1	PREDICTED: similar to cpn10 protein (LOC638892)	0.52
LOC639931		PREDICTED: similar to zinc RING finger protein SAG (LOC639931)	0.57
LOC640739	NM_009149	PREDICTED: hypothetical LOC640739 (LOC640739)	0.16

LOC654426	NM_027450	ATP synthase, H+ transporting, mitochondrial F0 complex, subunit F pseudogene (LOC654426), non-coding RNA.	0.53
LOC654467	NM_053108	heterogeneous nuclear ribonucleoprotein A1 pseudogene (LOC654467) on chromosome 9.	0.34
LOC654842	NM_001038592.1	hypothetical protein LOC654842 (LOC654842)	0.41
LOC664894	NM_023140.4	PREDICTED: similar to fusion protein: ubiquitin (bases 43_513); ribosomal protein S27a (bases 217_532) (LOC664894)	0.63
LOC665032	NM_028419.2	PREDICTED: similar to ribosomal protein (LOC665032)	0.50
LOC665235	NM_146211.2	PREDICTED: similar to hCG1642689 (LOC665235), misc RNA.	0.36
LOC665237	NM_008133.2	PREDICTED: similar to hCG31107 (LOC665237), misc RNA.	0.29
LOC665281	NM_008134.2	PREDICTED: similar to 40S ribosomal protein S12 (LOC665281), misc RNA.	0.36
LOC666559	XM_144253.6	PREDICTED: similar to farnesyl pyrophosphate synthase (LOC666559), misc RN	0.20
LOC666676	NM_001033297.2	PREDICTED: similar to ADP-ribosylation factor (LOC666676), misc RNA.	0.46
LOC667005	NM_001013771.2	PREDICTED: similar to cyclin B1 (LOC667005), misc RNA.	0.43
LOC667251	NM_146041.2	PREDICTED: similar to NADPH cytochrome B5 oxidoreductase (LOC667251), misc RNA.	0.64
LOC668038	NM_025508.3	PREDICTED: similar to ribosomal protein (LOC668038), misc RNA.	0.39
LOC668239	NM_177992.2	PREDICTED: similar to Rps13 protein (LOC668239)	0.42
LOC668387	NM_001033300.2	PREDICTED: similar to ribosomal protein S6 (LOC668387), misc RNA.	0.48
LOC668492	NM_010301.1	PREDICTED: similar to zinc finger CCHC-type and RNA binding motif 1 (LOC668492), misc RNA.	0.58
LOC668573	NM_010305.1	PREDICTED: similar to ribosomal protein (LOC668573), misc RNA.	0.42
LOC668706	NM_010308.3	PREDICTED: similar to Ribosomal protein L12 (LOC668706)	0.32
LOC669658	NM_008139.5	PREDICTED: similar to melanoma antigen (LOC669658)	0.67
LOC671523	NM_008142.3	PREDICTED: similar to ring finger and WD repeat domain 3 (LOC671523)	0.66
LOC671641	NM_013531.3	PREDICTED: similar to ribosomal protein S12 (LOC671641)	0.44
LOC671878	NM_015828	PREDICTED: similar to spermine synthase (LOC671878)	0.44
LOC672474	AK005561	PREDICTED: similar to 60S ribosomal protein L29 (LOC672474)	0.55
LOC674611	NM_001038637.1	PREDICTED: similar to Su48, transcript variant 3 (LOC674611)	0.33
LOC675377	NM_178846.2	PREDICTED: similar to Ribosomal protein L8 (LOC675377), misc RNA.	0.47
LOC675440	NM_011937.1	PREDICTED: similar to SMT3 suppressor of mif two 3 homolog 2 (S. cerevisiae), transcript variant 1 (LOC675440)	0.51
LOC675813	NM_008146.3	PREDICTED: similar to ribosomal protein S20 (LOC675813), misc RNA.	0.63
LOC675899	NM_175193.5	PREDICTED: similar to H2A histone family, member Z (LOC675899)	0.22
LOC676640	NM_001035122.2	PREDICTED: similar to neuron navigator 3, transcript variant 5 (LOC676640)	0.43
LOC676724	NM_027352.3	PREDICTED: hypothetical LOC676724 (LOC676724)	0.41
LOC676974	NM_029674.1	PREDICTED: similar to Glucose phosphate isomerase 1, transcript variant 2 (LOC676974)	0.57
LOC677205	NM_010325.2	PREDICTED: similar to DEAD (Asp-Glu-Ala-Asp) box polypeptide 18 (LOC677205), misc RNA.	0.57
LOC677213	NM_008149.3	PREDICTED: similar to U2AF homology motif (UHM) kinase 1 (LOC677213)	0.66
LOC677215	NM_010271.2	PREDICTED: similar to acidic ribosomal phosphoprotein P1, transcript variant 1 (LOC677215)	0.58
LOC677317	NM_010274.2	PREDICTED: similar to Mod1 protein, transcript variant 4 (LOC677317)	0.50
LOC677448	NM_008155.2	PREDICTED: similar to actin (LOC677448), misc RNA.	0.55
LOC677551	NM_008156.2	PREDICTED: similar to Ribosomal protein L19 (LOC677551), misc RNA.	0.62
Lor	AK020224	loricrin (Lor)	0.33
Lpl	NM_053110.3	lipoprotein lipase (Lpl)	0.56
Lrig1	NM_177469.3	leucine-rich repeats and immunoglobulin-like domains 1 (Lrig1)	0.51
Lrp12	NR_003568.1	low density lipoprotein-related protein 12 (Lrp12)	0.62
Lrrc59	NM_013533.3	leucine rich repeat containing 59 (Lrrc59)	0.39
Lrrfip1	NM_001025381.1	leucine rich repeat (in FLII) interacting protein 1 (Lrrfip1)	0.32
Lsg1	NM_175271.2	large subunit GTPase 1 homolog (S. cerevisiae) (Lsg1)	0.67
Lsm1	NM_134438.1	LSM1 homolog, U6 small nuclear RNA associated (S. cerevisiae) (Lsm1)	0.64
Lsm12	NM_022420.1	LSM12 homolog (S. cerevisiae) (Lsm12)	0.59
Lsm2	NM_153410.4	LSM2 homolog, U6 small nuclear RNA associated (S. cerevisiae) (Lsm2)	0.41
Lsm3	NM_134118.3	LSM3 homolog, U6 small nuclear RNA associated (S. cerevisiae) (Lsm3)	0.57
Lsm4	NM_008160.5	LSM4 homolog, U6 small nuclear RNA associated (S. cerevisiae) (Lsm4)	0.42
Lsm5	NM_013535.1	LSM5 homolog, U6 small nuclear RNA associated (S. cerevisiae) (Lsm5)	0.44
Lsm6	NM_080289.1	LSM6 homolog, U6 small nuclear RNA associated (S. cerevisiae) (Lsm6)	0.40
Lsm8	NM_008165.2	LSM8 homolog, U6 small nuclear RNA associated (S. cerevisiae) (Lsm8)	0.43
Lss	NM_016886.2	lanosterol synthase (Lss)	0.37
Ltbp1	NM_028736	latent transforming growth factor beta binding protein 1 (Ltbp1), transcript variant 2	0.46
Ly96	NM_019497.2	lymphocyte antigen 96 (Ly96)	0.55
Lyar	NM_001081414.1	Ly1 antibody reactive clone (Lyar)	0.58
Lypla2	NM_153419.1	lysophospholipase 2 (Lypla2)	0.58
Mad2l1	NM_010353.1	MAD2 (mitotic arrest deficient, homolog)-like 1 (yeast) (Mad2l1)	0.25
Mad2l1bp	NM_146066.1	MAD2L1 binding protein (Mad2l1bp)	0.63
Maged1	NM_010356.3	melanoma antigen, family D, 1 (Maged1)	0.39

Mageh1	NM_010357.1	melanoma antigen, family H, 1 (Mageh1)	0.61
Mak16	NM_010360.2	MAK16 homolog (S. cerevisiae) (Mak16)	0.50
Man2a1	NM_013541.1	mannosidase 2, alpha 1 (Man2a1)	0.51
Manba	NM_008185.2	mannosidase, beta A, lysosomal (Manba)	0.49
Map2k2	NM_026584.2	mitogen activated protein kinase kinase 2 (Map2k2)	0.66
Map2k3	NM_026816.3	mitogen activated protein kinase kinase 3 (Map2k3)	0.48
Map2k4	NM_022011.3	mitogen activated protein kinase kinase 4 (Map2k4)	0.66
Map3k11	NM_025652.1	mitogen-activated protein kinase kinase kinase 11 (Map3k11)	0.64
Map4k4	NM_207239.1	mitogen-activated protein kinase kinase kinase kinase 4 (Map4k4)	0.54
Mapk1ip1l	NM_032544.3	mitogen-activated protein kinase 1 interacting protein 1-like (Mapk1ip1l)	0.64
Mapk4	NM_027000.4	mitogen-activated protein kinase 4 (Mapk4)	0.66
Mapk6	NM_013882.1	mitogen-activated protein kinase 6 (Mapk6), transcript variant 2	0.53
Marcksl1	NM_010368.1	MARCKS-like 1 (Marcksl1)	0.57
Mast2	NM_010376.3	microtubule associated serine/threonine kinase 2 (Mast2), transcript variant 2	0.65
Mbd3	NM_198622.1	methyl-CpG binding domain protein 3 (Mbd3)	0.61
Mbnl1	NM_010436.2	muscleblind-like 1 (Drosophila) (Mbnl1)	0.48
Mboat1	NM_016750.1	membrane bound O-acyltransferase domain containing 1 (Mboat1)	0.47
Mcfd2	NM_010380.3	multiple coagulation factor deficiency 2 (Mcfd2)	0.61
Mcl1	XM_915849.1	myeloid cell leukemia sequence 1 (Mcl1)	0.57
Mcm10	NM_026115.3	minichromosome maintenance deficient 10 (S. cerevisiae) (Mcm10)	0.24
Mcm2	NM_010415.1	minichromosome maintenance deficient 2 mitotin (S. cerevisiae) (Mcm2)	0.30
Mcm3	NM_153198	minichromosome maintenance deficient 3 (S. cerevisiae) (Mcm3)	0.55
Mcm4	NM_010406.1	minichromosome maintenance deficient 4 homolog (S. cerevisiae) (Mcm4)	0.27
Mcm5	NM_008222.2	minichromosome maintenance deficient 5, cell division cycle 46 (S. cerevisiae) (Mcm5)	0.15
Mcm6	NM_008229.1	minichromosome maintenance deficient 6 (MIS5 homolog, S. pombe) (S. cerevisiae) (Mcm6)	0.26
Mcm7	NM_008230.4	minichromosome maintenance deficient 7 (S. cerevisiae) (Mcm7)	0.47
Mdga2	NM_008231.2	MAM domain containing glycosylphosphatidylinositol anchor 2 (Mdga2)	0.38
Mdh1	NM_144835.4	malate dehydrogenase 1, NAD (soluble) (Mdh1)	0.49
Mdh2	XM_974905.2	malate dehydrogenase 2, NAD (mitochondrial) (Mdh2), nuclear gene encoding mitochondrial protein	0.28
Med10	NM_172637.1	mediator of RNA polymerase II transcription, subunit 10 homolog (NUT2, S. cerevisiae) (Med10)	0.58
Mela	NM_026101.2		0.23
Melk	NM_008235.2	maternal embryonic leucine zipper kinase (Melk)	0.66
Mest	NM_019479.2	mesoderm specific transcript (Mest)	0.30
Metap1	NM_138753.2	methionyl aminopeptidase 1 (Metap1)	0.64
Metrn	NM_010423.2		0.34
Mettl9	NM_029095.1	methyltransferase like 9 (Mettl9)	0.65
Mex3a	NM_010431.1	mex3 homolog A (C. elegans) (Mex3a)	0.57
Mfge8	NM_008248.2		0.65
Mfhas1	NM_146001.1	malignant fibrous histiocytoma amplified sequence 1 (Mfhas1)	0.48
Mfn1	AK087586	mitofusin 1 (Mfn1)	0.58
Mif	NM_016786.2	macrophage migration inhibitory factor (Mif)	0.42
Mki67	NM_172746.2	PREDICTED: antigen identified by monoclonal antibody Ki 67 (Mki67)	0.15
Mki67ip	NM_178189.3	Mki67 (FHA domain) interacting nucleolar phosphoprotein (Mki67ip)	0.54
mKIAA0282	NM_178188.3		0.32
Mkks	NM_175661.1	McKusick-Kaufman syndrome protein (Mkks)	0.65
Mknk2	NM_178186.2	MAP kinase-interacting serine/threonine kinase 2 (Mknk2)	0.60
Mllt11	NM_175659.1	myeloid/lymphoid or mixed-lineage leukemia (trithorax homolog, Drosophila); translocated to, 11 (Mllt11)	0.40
Mmp14	NM_178182.1	matrix metallopeptidase 14 (membrane-inserted) (Mmp14)	0.64
Mmp15	NM_178183.1	matrix metallopeptidase 15 (Mmp15)	0.31
Mmp17	NM_178184.1	matrix metallopeptidase 17 (Mmp17)	0.58
Mod1	NM_178185.1	malic enzyme, supernatant (Mod1)	0.51
Mphosph6	NM_178194.3	M phase phosphoprotein 6 (Mphosph6)	0.55
Mphosph9	NM_013549.1	M-phase phosphoprotein 9 (Mphosph9)	0.47
Mpi	NM_178212.1	mannose phosphate isomerase (Mpi)	0.58
Mpnd	NM_178213.3	MPN domain containing (Mpnd)	0.60
Mre11a	NM_175662.1	meiotic recombination 11 homolog A (S. cerevisiae) (Mre11a)	0.57
Mrpl10	NM_016660.2	mitochondrial ribosomal protein L10 (Mrpl10), nuclear gene encoding mitochondrial protein	0.63
Mrpl12	NM_008252.2	mitochondrial ribosomal protein L12 (Mrpl12), nuclear gene encoding mitochondrial protein	0.43
Mrpl15	NM_008254.1		0.45
Mrpl17	NM_008255.1		0.51
Mrpl18	NM_016957.3	mitochondrial ribosomal protein L18 (Mrpl18), nuclear gene encoding mitochondrial protein	0.60

Mrpl2	NM_008258.1	mitochondrial ribosomal protein L2 (Mrpl2), nuclear gene encoding mitochondrial protein	0.52
Mrpl20	NM_198937.2	mitochondrial ribosomal protein L20 (Mrpl20), nuclear gene encoding mitochondrial protein	0.49
Mrpl22	NM_182650.3	mitochondrial ribosomal protein L22 (Mrpl22), nuclear gene encoding mitochondrial protein	0.62
Mrpl23	NM_001048061.1	mitochondrial ribosomal protein L23 (Mrpl23), nuclear gene encoding mitochondrial protein	0.61
Mrpl3	NM_133834.1	mitochondrial ribosomal protein L3 (Mrpl3), nuclear gene encoding mitochondrial protein	0.64
Mrpl30	NM_021510.2	mitochondrial ribosomal protein L30 (Mrpl30), nuclear gene encoding mitochondrial protein	0.54
Mrpl34	NM_025279.2	mitochondrial ribosomal protein L34 (Mrpl34), nuclear gene encoding mitochondrial protein	0.51
Mrpl37	NM_010447	mitochondrial ribosomal protein L37 (Mrpl37), nuclear gene encoding mitochondrial protein	0.60
Mrpl38	AK013709	mitochondrial ribosomal protein L38 (Mrpl38), nuclear gene encoding mitochondrial protein	0.60
Mrpl40	NM_016690.2	mitochondrial ribosomal protein L40 (Mrpl40), nuclear gene encoding mitochondrial protein	0.61
Mrpl43	NM_177301.4	mitochondrial ribosomal protein L43 (Mrpl43), nuclear gene encoding mitochondrial protein	0.65
Mrpl47	NM_144802.3	mitochondrial ribosomal protein L47 (Mrpl47), nuclear gene encoding mitochondrial protein	0.64
Mrpl50	NM_029804.1	mitochondrial ribosomal protein L50 (Mrpl50), nuclear gene encoding mitochondrial protein	0.66
Mrpl52	NM_152134.1	mitochondrial ribosomal protein L52 (Mrpl52), nuclear gene encoding mitochondrial protein	0.54
Mrpl53	NM_175606.2	mitochondrial ribosomal protein L53 (Mrpl53), nuclear gene encoding mitochondrial protein	0.44
Mrpl54	NM_016677.1	mitochondrial ribosomal protein L54 (Mrpl54), nuclear gene encoding mitochondrial protein	0.58
Mrps10	NM_013556.2		0.50
Mrps16	NM_008284	mitochondrial ribosomal protein S16 (Mrps16), nuclear gene encoding mitochondrial protein	0.49
Mrps18b	NM_019830.1	mitochondrial ribosomal protein S18B (Mrps18b), nuclear gene encoding mitochondrial protein	0.50
Mrps18c	NM_008287.2	mitochondrial ribosomal protein S18C (Mrps18c), nuclear gene encoding mitochondrial protein	0.62
Mrps2	NM_011828.2	mitochondrial ribosomal protein S2 (Mrps2), nuclear gene encoding mitochondrial protein	0.64
Mrps23	NM_018805.2	mitochondrial ribosomal protein S23 (Mrps23), nuclear gene encoding mitochondrial protein	0.66
Mrps24	NM_015819.3	mitochondrial ribosomal protein S24 (Mrps24), nuclear gene encoding mitochondrial protein	0.66
Mrps28	NM_019657.2	mitochondrial ribosomal protein S28 (Mrps28), nuclear gene encoding mitochondrial protein	0.57
Mrps30	NM_010476.3	mitochondrial ribosomal protein S30 (Mrps30), nuclear gene encoding mitochondrial protein	0.61
Mrps31	NM_153193.2	mitochondrial ribosomal protein S31 (Mrps31), nuclear gene encoding mitochondrial protein	0.65
Mrps35	NM_008302.3	mitochondrial ribosomal protein S35 (Mrps35), nuclear gene encoding mitochondrial protein	0.56
Mrps7	NM_011631.1	mitchondrial ribosomal protein S7 (Mrps7)	0.49
Mrps9	NM_008301.4	mitochondrial ribosomal protein S9 (Mrps9)	0.62
Mrto4	NM_031165.4	MRT4, mRNA turnover 4, homolog (S. cerevisiae) (Mrto4)	0.53
Msto1	NM_010481.1	misato homolog 1 (Drosophila) (Msto1)	0.66
Mta2	NM_030704.1	metastasis-associated gene family, member 2 (Mta2)	0.66
Mtap	NM_010477.3	methylthioadenosine phosphorylase (Mtap)	0.56
Mtap1s	NM_008303.2	microtubule-associated protein 1S (Mtap1s)	0.66
Mtap7d1		microtubule-associated protein 7 domain containing 1 (Mtap7d1)	0.63
mt-Atp6	NM_016865.2		0.65
Mtch2	NM_019564.2	mitochondrial carrier homolog 2 (C. elegans) (Mtch2), nuclear gene encoding mitochondrial protein	0.56
mt-Co2	NM_019752.2		0.55
Mtdh	NM_010489.2	Metadherin (Mtdh)	0.49
mtDNA_ATP8	NM_010490.2		0.55
mtDNA_ND1	NM_172015.1		0.66
Mthfd2	NM_198653.1		0.21
Mtmr11	XM_135030.4	myotubularin related protein 11 (Mtmr11)	0.40

Mtmr12	NM_019987.1	myotubularin related protein 12 (Mtmr12)	0.53
Mtmr2	NM_031166.2	myotubularin related protein 2 (Mtmr2)	0.58
Mtx1	AK013239	metaxin 1 (Mtx1)	0.66
Mvd	NM_031156.2	mevalonate (diphospho) decarboxylase (Mvd)	0.56
Mxra8	NM_029573.2	matrix-remodelling associated 8 (Mxra8)	0.44
Mybbp1a	NM_133662.2	MYB binding protein (P160) 1a (Mybbp1a)	0.47
Mybl2	NM_030244.3	myeloblastosis oncogene-like 2 (Mybl2)	0.48
Myef2	NM_010501.2	myelin basic protein expression factor 2, repressor (Myef2)	0.53
Myh9	NM_026820.2		0.47
Mylk	NM_008338.2	myosin, light polypeptide kinase (Mylk)	0.27
Myo1b	NM_031177.2	myosin IB (Myo1b)	0.65
Myo5a	NM_009879.2	myosin Va (Myo5a)	0.50
Myo6	NM_010515.1	myosin VI (Myo6)	0.58
N4wbp5-pending	NM_008342.2		0.60
N6amt2	NM_008343.2	N-6 adenine-specific DNA methyltransferase 2 (putative) (N6amt2)	0.51
Nab1	NM_010517.3	Ngfi-A binding protein 1 (Nab1)	0.59
Naca	NM_010518.2	nascent polypeptide-associated complex alpha polypeptide (Naca)	0.43
Naglu	NM_008048.2	alpha-N-acetylglucosaminidase (Sanfilippo disease IIIB) (Naglu)	0.54
Nans	NM_018741.1	N-acetylneuraminic acid synthase (sialic acid synthase) (Nans)	0.48
Nap1l1	NM_170599.2	nucleosome assembly protein 1-like 1 (Nap1l1)	0.39
Nap1l5	NM_019440.2	nucleosome assembly protein 1-like 5 (Nap1l5)	0.60
Narg1	NM_010555.4	NMDA receptor-regulated gene 1 (Narg1)	0.47
Nars	NM_010560.2	asparaginyl-tRNA synthetase (Nars)	0.66
Nasp	NM_026374.3		0.45
Nat5	NM_173751.3	N-acetyltransferase 5 (ARD1 homolog, S. cerevisiae) (Nat5)	0.47
Nav1	NM_029673.2		0.45
Ncan	NM_133976.1	neurocan (Ncan)	0.31
Ncapd2	NM_053261.2	non-SMC condensin I complex, subunit D2 (Ncapd2)	0.22
Ncapd3	NM_177730.3	non-SMC condensin II complex, subunit D3 (Ncapd3)	0.58
Ncaph	NM_011830	non-SMC condensin I complex, subunit H (Ncaph)	0.19
Ncdn	NM_016692.1	neurochondrin (Ncdn)	0.56
Ncl	NM_133345.2	nucleolin (Ncl)	0.66
Ncstn	NM_178641.3	nicastrin (Ncstn)	0.57
Ndc80	NM_008916.2	NDC80 homolog, kinetochore complex component (S. cerevisiae) (Ndc80)	0.43
Ndfip1	NM_153526.4	Nedd4 family interacting protein 1 (Ndfip1)	0.61
Ndufa12	NM_013754.1	NADH dehydrogenase (ubiquinone) 1 alpha subcomplex, 12 (Ndufa12)	0.55
Ndufa12l	NM_178632.4	PREDICTED: Ndufa12-like (Ndufa12l)	0.51
Ndufa3	NM_024267.6	NADH dehydrogenase (ubiquinone) 1 alpha subcomplex, 3 (Ndufa3)	0.67
Ndufa4	NM_023579.4	NADH dehydrogenase (ubiquinone) 1 alpha subcomplex, 4 (Ndufa4)	0.54
Ndufa5	NM_153774.1	NADH dehydrogenase (ubiquinone) 1 alpha subcomplex, 5 (Ndufa5), nuclear gene encoding mitochondrial protein	0.54
Ndufa6	NM_177128.3	NADH dehydrogenase (ubiquinone) 1 alpha subcomplex, 6 (B14) (Ndufa6), nuclear gene encoding mitochondrial protein	0.60
Ndufa8	NM_178378.2	NADH dehydrogenase (ubiquinone) 1 alpha subcomplex, 8 (Ndufa8), nuclear gene encoding mitochondrial protein	0.59
Ndufa9	NM_016721	NADH dehydrogenase (ubiquinone) 1 alpha subcomplex, 9 (Ndufa9)	0.61
Ndufb10	NM_001033484.1		0.62
Ndufb3	NM_026921.2	NADH dehydrogenase (ubiquinone) 1 beta subcomplex 3 (Ndufb3)	0.60
Ndufb5	NM_133934.2	NADH dehydrogenase (ubiquinone) 1 beta subcomplex, 5 (Ndufb5), nuclear gene encoding mitochondrial protein	0.58
Ndufb6	NM_013565.2	NADH dehydrogenase (ubiquinone) 1 beta subcomplex, 6 (Ndufb6), nuclear gene encoding mitochondrial protein	0.44
Ndufb8	NM_008402.1	NADH dehydrogenase (ubiquinone) 1 beta subcomplex 8 (Ndufb8)	0.63
Ndufc1	NM_010578.1	NADH dehydrogenase (ubiquinone) 1, subcomplex unknown, 1 (Ndufc1)	0.49
Ndufc2	NM_010580.1	NADH dehydrogenase (ubiquinone) 1, subcomplex unknown, 2 (Ndufc2)	0.50
Ndufs2	NM_008407.1	NADH dehydrogenase (ubiquinone) Fe-S protein 2 (Ndufs2)	0.62
Ndufs3	NM_008410.1	NADH dehydrogenase (ubiquinone) Fe-S protein 3 (Ndufs3)	0.63
Ndufs4	NM_172584.3	NADH dehydrogenase (ubiquinone) Fe-S protein 4 (Ndufs4), nuclear gene encoding mitochondrial protein	0.58
Ndufs7	XM_205854	NADH dehydrogenase (ubiquinone) Fe-S protein 7 (Ndufs7), nuclear gene encoding mitochondrial protein	0.62
Ndufs8	NM_019923.3	NADH dehydrogenase (ubiquinone) Fe-S protein 8 (Ndufs8)	0.56
Ndufv1	NM_026365.2	NADH dehydrogenase (ubiquinone) flavoprotein 1 (Ndufv1), nuclear gene encoding mitochondrial protein	0.66
Ndufv2	NM_146145.2	NADH dehydrogenase (ubiquinone) flavoprotein 2 (Ndufv2)	0.52
Necap1	NM_178394.3	NECAP endocytosis associated 1 (Necap1)	0.62
Nedd1	NM_023844.2		0.61
Nek3	NM_033398.2	NIMA (never in mitosis gene a)-related expressed kinase 3 (Nek3)	0.52
Nes	NM_025368.3	nestin (Nes)	0.51
Neto2	NM_010591.1	neuropilin (NRP) and tolloid (TLL)-like 2 (Neto2)	0.56

Neu1	NM_172872.2	neuraminidase 1 (Neu1)	0.65
Neurl	NM_053092.1		0.30
Nfatc3	NM_145958.1	nuclear factor of activated T-cells, cytoplasmic, calcineurin-dependent 3 (Nfatc3)	0.61
Nfib	NM_013568.3	nuclear factor I/B (Nfib)	0.65
Nfkbiz	NM_008421.2	nuclear factor of kappa light polypeptide gene enhancer in B-cells inhibitor, zeta (Nfkbiz)	0.55
Nhlrc2	NM_145922.2	NHL repeat containing 2 (Nhlrc2)	0.58
Nip7	NM_019789.2	nuclear import 7 homolog (S. cerevisiae) (Nip7)	0.63
Nipsnap3a	NM_008430.1	nipsnap homolog 3A (C. elegans) (Nipsnap3a)	0.56
Nkrf	NM_146037.1	NF-kappaB repressing factor (Nkrf)	0.58
Nkx2-2	NM_010607.1	NK2 transcription factor related, locus 2 (Drosophila) (Nkx2-2), transcript variant 1	0.37
Nme1	NM_177715.4	non-metastatic cells 1, protein (NM23A) expressed in (Nme1)	0.37
Nme2	NM_025888.5	non-metastatic cells 2, protein (NM23B) expressed in (Nme2), transcript variant 1	0.35
Nme4	NM_027008.1	non-metastatic cells 4, protein expressed in (Nme4), nuclear gene encoding mitochondrial protein	0.58
Nmral1	NM_023645.2	NmrA-like family domain containing 1 (Nmral1)	0.58
Nmt1	NM_025841.3	N-myristoyltransferase 1 (Nmt1)	0.60
Noc2l	NM_011317.3	nucleolar complex associated 2 homolog (S. cerevisiae) (Noc2l)	0.52
Noc4l	NM_010615.1	nucleolar complex associated 4 homolog (S. cerevisiae) (Noc4l)	0.61
Nol1	NM_010620.1	nucleolar protein 1 (Nol1)	0.55
Nol10	NM_139303.1	nucleolar protein 10 (Nol10)	0.63
Nol5	NM_183046.1	nucleolar protein 5 (Nol5)	0.27
Nol5a	NM_016705.2	nucleolar protein 5A (Nol5a)	0.45
Nola1	NM_145588.1	nucleolar protein family A, member 1 (H/ACA small nucleolar RNPs) (Nola1)	0.38
Nola2	NM_024245	nucleolar protein family A, member 2 (Nola2)	0.47
Nola3	NM_134471.3	nucleolar protein family A, member 3 (Nola3)	0.40
Nomo1	NM_008445.2	nodal modulator 1 (Nomo1)	0.37
Npc2	NM_008446.1	Niemann Pick type C2 (Npc2)	0.64
Npm1	NM_016761.1	nucleophosmin 1 (Npm1)	0.43
Npm3	NM_026324.2	nucleoplasmin 3 (Npm3)	0.22
Npm3-ps1	NM_013598.1	nucleoplasmin 3, pseudogene 1 (Npm3-ps1), non-coding RNA.	0.20
Nptn	NM_011803.2	neuroplastin (Nptn)	0.54
Nptx1	AK086122	neuronal pentraxin 1 (Nptx1)	0.22
Nqo2	NM_027117.1	NAD(P)H dehydrogenase, quinone 2 (Nqo2)	0.56
Nr2c2ap	NM_030075.1	nuclear receptor 2C2-associated protein (Nr2c2ap), transcript variant 2	0.67
Nr2f6	NM_172052.1	nuclear receptor subfamily 2, group F, member 6 (Nr2f6)	0.62
Nrarp	NM_177261.4	Notch-regulated ankyrin repeat protein (Nrarp)	0.43
Nrcam	NM_001042421.1	neuron-glia-CAM-related cell adhesion molecule (Nrcam)	0.53
Nrd1	NM_008465.4	nardilysin, N-arginine dibasic convertase, NRD convertase 1 (Nrd1)	0.60
Nrm	NM_010655.3	nurim (nuclear envelope membrane protein) (Nrm)	0.24
Nrn1	NM_008379.3	neuritin 1 (Nrn1)	0.38
Nrp1	NM_021284.4	neuropilin 1 (Nrp1)	0.46
Nsbp1	NM_025327.2	nucleosome binding protein 1 (Nsbp1)	0.53
Nsdhl	NM_024291.3	NAD(P) dependent steroid dehydrogenase-like (Nsdhl)	0.44
Nsep1	NM_001081171.2		0.47
Nsfl1c	NM_008483.2	NSFL1 (p97) cofactor (p47) (Nsfl1c)	0.62
Nt5c	NM_010683.1	5',3'-nucleotidase, cytosolic (Nt5c)	0.65
Nt5dc2	NM_001017959.1	5'-nucleotidase domain containing 2 (Nt5dc2)	0.54
Ntan1	NM_029789.1		0.64
Nubp1	NM_028015.2	nucleotide binding protein 1 (Nubp1)	0.35
Nucb1	NM_029999.3	nucleobindin 1 (Nucb1)	0.65
Nudc	NM_133815	nuclear distribution gene C homolog (Aspergillus) (Nudc)	0.64
Nudt19	NM_010699.1	nudix (nucleoside diphosphate linked moiety X)-type motif 19 (Nudt19)	0.61
Nudt21	NM_001039522.1	nudix (nucleoside diphosphate linked moiety X)-type motif 21 (Nudt21)	0.44
Nudt5	NM_008495.1	nudix (nucleoside diphosphate linked moiety X)-type motif 5 (Nudt5)	0.44
Nup133	NM_010705.2	nucleoporin 133 (Nup133)	0.61
Nup160	NM_010706.1	nucleoporin 160 (Nup160)	0.52
Nup205	NM_018886.3	PREDICTED: nucleoporin 205, transcript variant 1 (Nup205)	0.36
Nup210	NM_145219.2	nucleoporin 210 (Nup210)	0.19
Nup43	NM_008133.3	nucleoporin 43 (Nup43)	0.45
Nup62	NM_175386.3	nucleoporin 62 (Nup62)	0.32
Nup85	NM_010715.2	nucleoporin 85 (Nup85)	0.51
Nup88	NM_010716	nucleoporin 88 (Nup88), transcript variant 1	0.48
Nus1	NM_013860.1	nuclear undecaprenyl pyrophosphate synthase 1 homolog (S. cerevisiae) (Nus1)	0.57
Nusap1	NM_001034030.1	nucleolar and spindle associated protein 1 (Nusap1), transcript variant 2	0.21
Nxph1	NM_172714.1	neurexophilin 1 (Nxph1)	0.16
Nxt1	NM_021460.1	NTF2-related export protein 1 (Nxt1)	0.54

Ociad2	NM_153404.2	OCIA domain containing 2 (Ociad2)	0.37
Ogdh	NM_019980.1	oxoglutarate dehydrogenase (lipoamide) (Ogdh), nuclear gene encoding mitochondrial protein	0.60
Ola1	NM_025681.2	Obg-like ATPase 1 (Ola1), transcript variant 1	0.59
Olfm2	NM_008502.1	olfactomedin 2 (Olfm2)	0.43
Olfml2b	NM_027400.2	olfactomedin-like 2B (Olfml2b)	0.41
Olig1	NM_010721.1	oligodendrocyte transcription factor 1 (Olig1)	0.41
Opn3	NM_008505.3	opsin 3 (Opn3)	0.53
Oprs1	XM_001472500.1	opioid receptor, sigma 1 (Oprs1)	0.57
Orai1	XM_001473099.1	ORAI calcium release-activated calcium modulator 1 (Orai1)	0.61
Orc5l	XM_001473215.1	origin recognition complex, subunit 5-like (S. cerevisiae) (Orc5l)	0.62
Orc6l	XR_031048.1	origin recognition complex, subunit 6-like (S. cerevisiae) (Orc6l)	0.48
Osbpl3	XM_001473500.1	oxysterol binding protein-like 3 (Osbpl3)	0.67
Osbpl6	XM_001475112.1	oxysterol binding protein-like 6 (Osbpl6)	0.32
Osbpl8	XM_001475189.1	oxysterol binding protein-like 8 (Osbpl8), transcript variant 1	0.66
Ostm1	XM_001475096.1	osteopetrosis associated transmembrane protein 1 (Ostm1)	0.52
Oxct1	XM_001476627.1	3-oxoacid CoA transferase 1 (Oxct1)	0.65
Oxr1	XM_001478166.1	oxidation resistance 1 (Oxr1), transcript variant 1	0.59
P2ry5	XM_001478119.1	purinergic receptor P2Y, G-protein coupled, 5 (P2ry5)	0.30
P4hb	XM_001477137.1	prolyl 4-hydroxylase, beta polypeptide (P4hb)	0.54
Pa2g4	XM_001478555.1	proliferation-associated 2G4 (Pa2g4)	0.40
Pabpc4	XM_001478518.1	poly A binding protein, cytoplasmic 4 (Pabpc4), transcript variant 1	0.50
Pafah1b1	XM_001478939.1	platelet-activating factor acetylhydrolase, isoform 1b, beta1 subunit (Pafah1b1)	0.55
Pafah1b3	XM_001479207.1	platelet-activating factor acetylhydrolase, isoform 1b, alpha1 subunit (Pafah1b3)	0.66
Pak4	XM_001479822.1	p21 (CDKN1A)-activated kinase 4 (Pak4)	0.66
Palmd	XM_001480293.1	palmdelphin (Palmd)	0.41
Pank1	XM_001480197.1	pantothenate kinase 1 (Pank1), transcript variant 2	0.62
Pank3	XM_001479903.1	pantothenate kinase 3 (Pank3)	0.64
Parp1	XM_001480106.1	poly (ADP-ribose) polymerase family, member 1 (Parp1)	0.52
Pbk	XM_001480194.1	PDZ binding kinase (Pbk)	0.08
Pbrm1	XM_001481024.1	polybromo 1 (Pbrm1)	0.66
Pcbp1	XM_001480621.1	poly(rC) binding protein 1 (Pcbp1)	0.45
Pcbp2	XM_001481075.1	poly(rC) binding protein 2 (Pcbp2)	0.57
Pcdh17	XM_001481083.1	protocadherin 17 (Pcdh17)	0.46
Pcdh7	XM_001481017.1	protocadherin 7 (Pcdh7)	0.45
Pcdh9	XM_001481025.1	protocadherin 9 (Pcdh9)	0.55
Pcyt2	XM_001481214.1	phosphate cytidylyltransferase 2, ethanolamine (Pcyt2)	0.64
Pdap1	XM_001474702.1	PDGFA associated protein 1 (Pdap1)	0.36
Pdcd2l	XM_001471637.1	programmed cell death 2-like (Pdcd2l)	0.62
Pdgfa	XM_001471968.1		0.38
Pdhb	XM_001472489.1	pyruvate dehydrogenase (lipoamide) beta (Pdhb)	0.54
Pdia3	XM_001472256.1	protein disulfide isomerase associated 3 (Pdia3)	0.49
Pdia5	XM_001473268.1	protein disulfide isomerase associated 5 (Pdia5)	0.41
Pdlim4	XM_001472872.1	PDZ and LIM domain 4 (Pdlim4)	0.63
Pdlim5	XM_001473652.1	PDZ and LIM domain 5 (Pdlim5), transcript variant 1	0.62
Pdss1	XR_031422.1	prenyl (solanesyl) diphosphate synthase, subunit 1 (Pdss1)	0.53
Pdxp	XR_031278.1	pyridoxal (pyridoxine, vitamin B6) phosphatase (Pdxp)	0.54
Pdzrn4	XM_001474658.1	PREDICTED: PDZ domain containing RING finger 4 (Pdzrn4)	0.49
Peli1	XM_001473900.1	pellino 1 (Peli1)	0.56
Pfdn2	XR_031575.1	prefoldin 2 (Pfdn2)	0.59
Pfdn4	XM_001474411.1	prefoldin 4 (Pfdn4), transcript variant 2	0.32
Pfn1	XM_001473941.1	profilin 1 (Pfn1)	0.44
Pgam1	XM_001474718.1	phosphoglycerate mutase 1 (Pgam1)	0.54
Pgam5	XR_031705.1	phosphoglycerate mutase family member 5 (Pgam5)	0.45
Pgk1	XM_001475152.1	phosphoglycerate kinase 1 (Pgk1)	0.55
Pgls	XR_031796.1	6-phosphogluconolactonase (Pgls)	0.52
Pgp	XR_031944.1	phosphoglycolate phosphatase (Pgp)	0.34
Pgs1	XM_001475019.1	phosphatidylglycerophosphate synthase 1 (Pgs1)	0.64
Phb2	XM_001475055.1	prohibitin 2 (Phb2)	0.42
Phc2	XM_001475106.1	polyhomeotic-like 2 (Drosophila) (Phc2)	0.41
Phca	XR_032154.1	phytoceramidase, alkaline (Phca)	0.65
Phf10	XM_001475298.1	PHD finger protein 10 (Phf10)	0.62
Phf20	XM_001475422.1	PHD finger protein 20 (Phf20)	0.46
Phf5a	XM_001475447.1	PHD finger protein 5A (Phf5a)	0.57
Phf6	XM_001475611.1	PHD finger protein 6 (Phf6)	0.61
Phlda1	XM_001475801.1	pleckstrin homology-like domain, family A, member 1 (Phlda1)	0.41
Phtf2	XM_001475830.1	putative homeodomain transcription factor 2 (Phtf2)	0.60
Pi4ka	XM_001476721.1	phosphatidylinositol 4-kinase, catalytic, alpha polypeptide (Pi4ka)	0.58
Picalm	XM_001476728.1		0.60
Pif1	XM_001475765.1	PIF1 5'-to-3' DNA helicase homolog (S. cerevisiae) (Pif1)	0.39
Piga	XM_001476780.1	phosphatidylinositol glycan anchor biosynthesis, class A (Piga)	0.63

Pih1d1	XM_001476909.1	PIH1 domain containing 1 (Pih1d1)	0.65
Pip4k2b	XM_001476916.1	phosphatidylinositol-5-phosphate 4-kinase, type II, beta (Pip4k2b)	0.56
Pitpnb	XM_001477026.1	phosphatidylinositol transfer protein, beta (Pitpnb)	0.63
Pitrm1	XM_001477492.1	pitrilysin metallepetidase 1 (Pitrm1)	0.63
Pja1	XM_001477537.1		0.58
Pja2	XM_001477568.1	praja 2, RING-H2 motif containing (Pja2), transcript variant 2	0.57
Pkd1	XM_001477596.1	polycystic kidney disease 1 homolog (Pkd1)	0.66
Pkia	XR_033770.1	protein kinase inhibitor, alpha (Pkia)	0.62
Pkm2	XM_001477665.1		0.43
Pkn2	XR_033502.1	protein kinase N2 (Pkn2)	0.65
Pla2g12a	XR_033381.1	phospholipase A2, group XIIA (Pla2g12a), transcript variant 1	0.63
Plaa	XM_001477759.1	phospholipase A2, activating protein (Plaa)	0.61
Pld1	XM_001477774.1		0.52
Plekha8	XM_001477963.1	pleckstrin homology domain containing, family A (phosphoinositide binding specific) member 8 (Plekha8)	0.61
Plekhk1	XM_001478437.1		0.28
Plk1	XR_033484.1	polo-like kinase 1 (Drosophila) (Plk1)	0.11
Plk2	XR_033736.1	polo-like kinase 2 (Drosophila) (Plk2)	0.21
Plk4	XR_032896.1		0.57
Plvap	XM_001478602.1	plasmalemma vesicle associated protein (Plvap)	0.15
Plxna2	XM_001478657.1	plexin A2 (Plxna2)	0.59
Pml	XM_001478724.1	promyelocytic leukemia (Pml), transcript variant 1	0.61
Pmp22	XM_001478788.1	peripheral myelin protein 22 (Pmp22)	0.38
Pno1	XM_001479214.1	partner of NOB1 homolog (S. cerevisiae) (Pno1)	0.46
Pola2	XM_001478835.1	polymerase (DNA directed), alpha 2 (Pola2)	0.66
Pold1	XM_001479392.1	polymerase (DNA directed), delta 1, catalytic subunit (Pold1)	0.29
Pold2	XM_001479068.1	polymerase (DNA directed), delta 2, regulatory subunit (Pold2)	0.51
Pold4	XM_001478074.1		0.55
Pole	XR_033756.1	polymerase (DNA directed), epsilon (Pole)	0.45
Pole4	XR_033905.1	polymerase (DNA-directed), epsilon 4 (p12 subunit) (Pole4)	0.60
Polg	XM_001479497.1	polymerase (DNA directed), gamma (Polg)	0.65
Polr1a	XR_034387.1	polymerase (RNA) I polypeptide A (Polr1a)	0.58
Polr1d	XM_001480136.1	polymerase (RNA) I polypeptide D (Polr1d), transcript variant 1	0.61
Polr1e	XR_034509.1	polymerase (RNA) I polypeptide E (Polr1e)	0.58
Polr2a	XM_001480186.1		0.59
Polr2g	XM_001480467.1	polymerase (RNA) II (DNA directed) polypeptide G (Polr2g)	0.66
Polr2h	XM_001480451.1	polymerase (RNA) II (DNA directed) polypeptide H (Polr2h)	0.64
Polr3k	XM_001480453.1	polymerase (RNA) III (DNA directed) polypeptide K (Polr3k)	0.53
Pomt2	XM_001480806.1	protein-O-mannosyltransferase 2 (Pomt2)	0.63
Pop5	XM_001480785.1	processing of precursor 5, ribonuclease P/MRP family (S. cerevisiae) (Pop5)	0.57
Por	XM_001480380.1	P450 (cytochrome) oxidoreductase (Por)	0.60
Pou3f1	XM_001480791.1	POU domain, class 3, transcription factor 1 (Pou3f1)	0.20
Ppa1	XM_001471617.1	pyrophosphatase (inorganic) 1 (Ppa1)	0.43
Ppa2	XM_137003.2	pyrophosphatase (inorganic) 2 (Ppa2), nuclear gene encoding mitochondrial protein	0.56
Ppan	XM_125952.4		0.42
Ppargc1a	XM_122711.2	peroxisome proliferative activated receptor, gamma, coactivator 1 alpha (Ppargc1a)	0.58
Ppat	XM_139078.2	PREDICTED: phosphoribosyl pyrophosphate amidotransferase, transcript variant 8 (Ppat)	0.59
Ppia	XM_122761.3	peptidylprolyl isomerase A (Ppia)	0.43
Ppib	XM_140116.1	peptidylprolyl isomerase B (Ppib)	0.44
Ppic	XR_031257.1	peptidylprolyl isomerase C (Ppic)	0.65
Ppid	XM_129460.4		0.58
Ppif	XM_124173.2	peptidylprolyl isomerase F (cyclophilin F) (Ppif), nuclear gene encoding mitochondrial protein	0.43
Ppih	XM_124269.2		0.56
Ppm1b	XM_124427.4	protein phosphatase 1B, magnesium dependent, beta isoform (Ppm1b)	0.62
Ppm1g	XR_002491.1	protein phosphatase 1G (formerly 2C), magnesium-dependent, gamma isoform (Ppm1g)	0.61
Ppp1ca	XM_124826.2	protein phosphatase 1, catalytic subunit, alpha isoform (Ppp1ca)	0.63
Ppp1r12a	XM_124858.2	protein phosphatase 1, regulatory (inhibitor) subunit 12A (Ppp1r12a)	0.53
Ppp1r14b	XM_145374.2		0.39
Ppp1r8	XR_001889.1	protein phosphatase 1, regulatory (inhibitor) subunit 8 (Ppp1r8)	0.64
Ppp2cb	XM_137226.3	protein phosphatase 2 (formerly 2A), catalytic subunit, beta isoform (Ppp2cb)	0.61
Ppp2r5c	XM_111221.4	protein phosphatase 2, regulatory subunit B (B56), gamma isoform (Ppp2r5c), transcript variant 3	0.41
Ppp3ca	XM_138902.2	protein phosphatase 3, catalytic subunit, alpha isoform (Ppp3ca)	0.66
Ppp3cb	XM_917314.2		0.47

Ppp3r1	XM_142236.4	protein phosphatase 3, regulatory subunit B, alpha isoform (calcineurin B, type I) (Ppp3r1)	0.63
Ppp6c	XM_147335.2	protein phosphatase 6, catalytic subunit (Ppp6c)	0.60
Pprc1	XM_195821.2	peroxisome proliferative activated receptor, gamma, coactivator-related 1 (Pprc1)	0.54
Prc1	XM_193742.2	protein regulator of cytokinesis 1 (Prc1)	0.06
Prcp	XM_193262.3	prolylcarboxypeptidase (angiotensinase C) (Prcp)	0.33
Prdx4	XM_193374.2	peroxiredoxin 4 (Prdx4)	0.48
Prep	XM_193524.2	prolyl endopeptidase (Prep)	0.46
Prkaca	XM_195069.2	protein kinase, cAMP dependent, catalytic, alpha (Prkaca)	0.49
Prkacb	XM_195066.3	protein kinase, cAMP dependent, catalytic, beta (Prkacb)	0.63
Prkar1a	XM_195399.2	protein kinase, cAMP dependent regulatory, type I, alpha (Prkar1a)	0.66
Prkar2b	XM_204210.2	protein kinase, cAMP dependent regulatory, type II beta (Prkar2b)	0.55
Prkcb	XM_207178.1	protein kinase C, beta (Prkcb)	0.09
Prkcbp1	XM_282724.2	protein kinase C binding protein 1 (Prkcbp1)	0.61
Prkcsh	XM_287286.2	protein kinase C substrate 80K-H (Prkcsh)	0.60
Prkd3	XM_284573.2	protein kinase D3 (Prkd3)	0.61
Prmt3	XM_284733.2	protein arginine N-methyltransferase 3 (Prmt3)	0.58
Prmt5	XM_284853.2	protein arginine N-methyltransferase 5 (Prmt5)	0.60
Prmt7	XR_032458.1	protein arginine N-methyltransferase 7 (Prmt7)	0.47
Prokr1	XM_354560.1	prokineticin receptor 1 (Prokr1)	0.51
Prpf19	XM_354604.1	PRP19/PSO4 pre-mRNA processing factor 19 homolog (S. cerevisiae) (Prpf19)	0.58
Prpf38a	XM_354622.1	PRP38 pre-mRNA processing factor 38 (yeast) domain containing A (Prpf38a)	0.52
Prr18	XM_354834.1	proline rich region 18 (Prr18)	0.49
Prrc1	XM_358467.1	proline-rich coiled-coil 1 (Prrc1)	0.58
Prss35	XM_354911.1	protease, serine, 35 (Prss35)	0.27
Psip1	XM_354963.1	PC4 and SFRS1 interacting protein 1 (Psip1)	0.61
Psma5	XM_355032.1	proteasome (prosome, macropain) subunit, alpha type 5 (Psma5)	0.56
Psma7	XM_355049.1	proteasome (prosome, macropain) subunit, alpha type 7 (Psma7)	0.52
Psmb1	XM_355056.1	proteasome (prosome, macropain) subunit, beta type 1 (Psmb1)	0.62
Psmb10	XM_355149.1	proteasome (prosome, macropain) subunit, beta type 10 (Psmb10)	0.50
Psmb2	XM_355167.1	proteasome (prosome, macropain) subunit, beta type 2 (Psmb2)	0.65
Psmb3	XM_355202.1	proteasome (prosome, macropain) subunit, beta type 3 (Psmb3)	0.63
Psmb4	XM_355288.1	proteasome (prosome, macropain) subunit, beta type 4 (Psmb4)	0.59
Psmb5	XM_355330.1	proteasome (prosome, macropain) subunit, beta type 5 (Psmb5)	0.55
Psmb6	XM_355356.1	proteasome (prosome, macropain) subunit, beta type 6 (Psmb6)	0.66
Psmb7	XM_355403.1	proteasome (prosome, macropain) subunit, beta type 7 (Psmb7)	0.55
Psmc1	XM_355526.1		0.53
Psmc2	XM_355612.1	proteasome (prosome, macropain) 26S subunit, ATPase 2 (Psmc2)	0.61
Psmc3ip	XM_355721.1	proteasome (prosome, macropain) 26S subunit, ATPase 3, interacting protein (Psmc3ip)	0.52
Psmc4	XM_355801.1	proteasome (prosome, macropain) 26S subunit, ATPase, 4 (Psmc4)	0.63
Psmc5	XM_355809.1	protease (prosome, macropain) 26S subunit, ATPase 5 (Psmc5)	0.63
Psmd12	XM_355907.1	proteasome (prosome, macropain) 26S subunit, non-ATPase, 12 (Psmd12)	0.50
Psmd14	XM_355964.1		0.62
Psmd8	XM_358681.1	proteasome (prosome, macropain) 26S subunit, non-ATPase, 8 (Psmd8)	0.57
Psme3	XM_356062.1	proteaseome (prosome, macropain) 28 subunit, 3 (Psme3)	0.59
Ptcd2	XM_356141.1	pentatricopeptide repeat domain 2 (Ptcd2)	0.64
Pten	XM_356263.1	phosphatase and tensin homolog (Pten)	0.65
Ptk2	XM_356342.1	PTK2 protein tyrosine kinase 2 (Ptk2)	0.39
Ptn	XM_356731.1	pleiotrophin (Ptn)	0.55
Ptpn12	XM_356798.1	protein tyrosine phosphatase, non-receptor type 12 (Ptpn12)	0.63
Ptpro	XM_356873.1	protein tyrosine phosphatase, receptor type, O (Ptpro)	0.31
Ptprz1	XM_356900.1	protein tyrosine phosphatase, receptor type Z, polypeptide 1 (Ptprz1)	0.46
Pum2	XM_356977.1	pumilio 2 (Drosophila) (Pum2)	0.64
Pus7l	XM_356987.1	pseudouridylate synthase 7 homolog (S. cerevisiae)-like (Pus7l)	0.48
Pusl1	XM_357315.1	PREDICTED: pseudouridylate synthase-like 1, transcript variant 1 (Pusl1)	0.58
Pvrl3	XM_357338.1	poliovirus receptor-related 3 (Pvrl3), transcript variant alpha	0.62
Pycr2	XM_357429.1	pyrroline-5-carboxylate reductase family, member 2 (Pycr2)	0.59
Qpct	XM_357488.1	glutaminyl-peptide cyclotransferase (glutaminyl cyclase) (Qpct)	0.55
Qrsl1	XM_357823.1	glutaminyl-tRNA synthase (glutamine-hydrolyzing)-like 1 (Qrsl1)	0.55
Rab26	XM_358153.1	PREDICTED: RAB26, member RAS oncogene family, transcript variant 1 (Rab2(	0.06
Rab34	XM_358163.1	RAB34, member of RAS oncogene family (Rab34)	0.56
Rab35	XM_358915.1	RAB35, member RAS oncogene family (Rab35)	0.51
Rab3a	XM_358960.1	RAB3A, member RAS oncogene family (Rab3a)	0.47
Rab3b	XM_358981.1		0.37
Rab3gap2	XR_034995.1	PREDICTED: RAB3 GTPase activating protein subunit 2, transcript variant 4 (Rab3gap2)	0.52
Rab5c	XM_359014.1	RAB5C, member RAS oncogene family (Rab5c)	0.59
Rab8b	XM_359028.1	RAB8B, member RAS oncogene family (Rab8b)	0.38
Rabggtb	XM_359031.1	RAB geranylgeranyl transferase, b subunit (Rabggtb)	0.54

Rabif	XM_359035.1	RAB interacting factor (Rabif)	0.63
Rad51	XM_359058.1	RAD51 homolog (S. cerevisiae) (Rad51)	0.57
Rad51c	XM_359091.1	Rad51 homolog c (S. cerevisiae) (Rad51c)	0.48
Rad54l	XM_359123.1	RAD54 like (S. cerevisiae) (Rad54l)	0.56
Raet1b	XM_359142.1	retinoic acid early transcript beta (Raet1b)	0.36
Rag1ap1	XM_359148.1	recombination activating gene 1 activating protein 1 (Rag1ap1)	0.64
Rai12	XM_359163.1	retinoic acid induced 12 (Rai12)	0.65
Raly	XM_359184.1	hnRNP-associated with lethal yellow (Raly)	0.34
Ramp2	XM_917865.2	receptor (calcitonin) activity modifying protein 2 (Ramp2)	0.65
Ran	XM_906947.2	RAN, member RAS oncogene family (Ran)	0.57
Ranbp1	XM_001475733.1	RAN binding protein 1 (Ranbp1)	0.36
Rangap1	XM_485435.3		0.44
Rangrf	XR_034042.1	RAN guanine nucleotide release factor (Rangrf)	0.50
Rap1b	XR_033167.1	RAS related protein 1b (Rap1b)	0.50
Rap2c	NR_002889.1	RAP2C, member of RAS oncogene family (Rap2c)	0.29
Rars	XR_034542.1	arginyl-tRNA synthetase (Rars)	0.66
Rasa3	XM_619824.4	RAS p21 protein activator 3 (Rasa3)	0.33
Rasal2	XR_034790.1	RAS protein activator like 2 (Rasal2)	0.61
Rassf3	XR_031536.1	Ras association (RalGDS/AF-6) domain family member 3 (Rassf3)	0.18
Rbbp4	XR_032410.1	retinoblastoma binding protein 4 (Rbbp4)	0.64
Rbbp7	XR_031862.1	retinoblastoma binding protein 7 (Rbbp7)	0.57
Rbl1	XR_004752.1	retinoblastoma-like 1 (p107) (Rbl1)	0.39
Rbm13	XR_033501.1	RNA binding motif protein 13 (Rbm13)	0.37
Rbm15	XM_001480472.1		0.61
Rbms3	XR_033663.1	RNA binding motif, single stranded interacting protein (Rbms3)	0.55
Rbmx	XM_904332.3	RNA binding motif protein, X chromosome (Rbmx)	0.31
Rbx1	XR_001819.2	ring-box 1 (Rbx1)	0.60
Rcc1	XR_030945.1	regulator of chromosome condensation 1 (Rcc1)	0.54
Rcl1	XR_031205.1	RNA terminal phosphate cyclase-like 1 (Rcl1)	0.63
Rdh11	XM_906862.3		0.53
Reps1	XR_031426.1	RalBP1 associated Eps domain containing protein (Reps1)	0.52
Rerg	XM_921371.2	RAS-like, estrogen-regulated, growth-inhibitor (Rerg)	0.35
Rev3l	XM_907756.3	REV3-like, catalytic subunit of DNA polymerase zeta RAD54 like (S. cerevisiae) (Rev3l)	0.59
Rfc1	XR_003906.1	replication factor C (activator 1) 1 (Rfc1)	0.67
Rfc2	XM_915945.3	replication factor C (activator 1) 2 (Rfc2)	0.60
Rfc3	XM_915073.1		0.65
Rfc4	XM_915139.3		0.33
Rfc5	XM_916567.2	replication factor C (activator 1) 5 (Rfc5)	0.55
Rgl1	XM_925296.2	ral guanine nucleotide dissociation stimulator,-like 1 (Rgl1)	0.59
Rgs7	NR_002690.1	regulator of G protein signaling 7 (Rgs7)	0.35
Rgs7bp	NR_002885.1	regulator of G-protein signalling 7 binding protein (Rgs7bp)	0.47
Rhbdf1	NM_001081681.1	rhomboid family 1 (Drosophila) (Rhbdf1)	0.59
Rhobtb2	XM_973311.2	Rho-related BTB domain containing 2 (Rhobtb2)	0.58
Rhoc	XM_991641.1	ras homolog gene family, member C (Rhoc)	0.57
Rhoj	XR_031650.1	ras homolog gene family, member J (Rhoj)	0.64
Rhot2	XR_031476.1	ras homolog gene family, member T2 (Rhot2), nuclear gene encoding mitochondrial protein	0.64
Riok3	XR_031065.1	RIO kinase 3 (yeast) (Riok3)	0.63
Rn18s	XR_031145.1	18S RNA (Rn18s), non-coding RNA.	0.28
Rnaseh2b	XR_030655.1	ribonuclease H2, subunit B (Rnaseh2b)	0.57
Rnf14	XR_030797.1	ring finger protein 14 (Rnf14)	0.62
Rnf145	XR_032081.1	ring finger protein 145 (Rnf145)	0.65
Rnf26	XM_001480084.1	ring finger protein 26 (Rnf26)	0.42
Rnf4	XR_034201.1	ring finger protein 4 (Rnf4)	0.63
Rnf44	XM_001000226.2	ring finger protein 44 (Rnf44)	0.53
Rnps1	XR_031651.1		0.54
Robo1	XR_034647.1	roundabout homolog 1 (Drosophila) (Robo1)	0.40
Rpa3	XR_035016.1	replication protein A3 (Rpa3)	0.46
Rpl10a	XM_001003212.2	ribosomal protein L10A (Rpl10a)	0.48
Rpl12	XM_976371.1	ribosomal protein L12 (Rpl12)	0.30
Rpl13a	XM_987550.1	ribosomal protein L13a (Rpl13a)	0.26
Rpl18	XM_001479052.1	ribosomal protein L18 (Rpl18)	0.42
Rpl18a	XM_001473434.1	Ribosomal protein L18A (Rpl18a)	0.42
Rpl19	XM_993721.1	ribosomal protein L19 (Rpl19)	0.37
Rpl23	XM_980150.1	ribosomal protein L23 (Rpl23)	0.58
Rpl24	XR_005113.2	ribosomal protein L24 (Rpl24)	0.62
Rpl26	XM_001479274.1	ribosomal protein L26 (Rpl26)	0.49
Rpl27	XM_001481199.1	ribosomal protein L27 (Rpl27)	0.45
Rpl27a	XR_032773.1	ribosomal protein L27a (Rpl27a)	0.30
Rpl28	XM_985882.1		0.43

Rpl29	XM_001003712.1	ribosomal protein L29 (Rpl29)	0.41
Rpl3	XM_992449.1	ribosomal protein L3 (Rpl3)	0.64
Rpl30	XM_001003154.1	ribosomal protein L30 (Rpl30)	0.54
Rpl31	XR_034818.1	ribosomal protein L31 (Rpl31)	0.46
Rpl32	XM_001001408.2		0.42
Rpl34	XM_001001411.2	ribosomal protein L34 (Rpl34), transcript variant 1	0.54
Rpl35	XM_001004685.1	ribosomal protein L35 (Rpl35)	0.40
Rpl36a	XR_005046.1	ribosomal protein L36a (Rpl36a)	0.37
Rpl36al	XM_001002943.1	ribosomal protein L36a-like (Rpl36al)	0.42
Rpl37	XR_005062.2	ribosomal protein L37 (Rpl37)	0.57
Rpl38	XR_005084.2	ribosomal protein L38 (Rpl38), transcript variant 1	0.59
Rpl39	NM_008508.2	ribosomal protein L39 (Rpl39)	0.46
Rpl4	NM_008509.2	ribosomal protein L4 (Rpl4)	0.38
Rpl41	NM_008377.2	ribosomal protein L41 (Rpl41)	0.63
Rpl7l1	NM_172814.1	ribosomal protein L7-like 1 (Rpl7l1)	0.65
Rpl9	NM_001081088.1	ribosomal protein L9 (Rpl9)	0.62
Rplp0	NM_001033371.2	ribosomal protein, large, P0 (Rplp0)	0.52
Rplp1	NM_029044.2	ribosomal protein, large, P1 (Rplp1)	0.51
Rplp2	XM_890234.2	ribosomal protein, large P2 (Rplp2)	0.43
Rpn2	NM_133807.1	ribophorin II (Rpn2)	0.47
Rpp21	NM_177725.2	ribonuclease P 21 subunit (human) (Rpp21)	0.60
Rprm	NM_008515.1	reprimo, TP53 dependent G2 arrest mediator candidate (Rprm)	0.40
Rps10	NM_008516.2	ribosomal protein S10 (Rps10)	0.50
Rps11	NM_178069.5	ribosomal protein S11 (Rps11)	0.52
Rps12	NM_026032.1		0.43
Rps13	NM_172947.1	ribosomal protein S13 (Rps13)	0.41
Rps15	NM_030597.2	ribosomal protein S15 (Rps15)	0.51
Rps16	NM_026309.1	ribosomal protein S16 (Rps16)	0.40
Rps19	NM_015816.1	ribosomal protein S19 (Rps19)	0.48
Rps2	NM_025520.2	ribosomal protein S2 (Rps2)	0.53
Rps21	NM_030145.2	ribosomal protein S21 (Rps21)	0.58
Rps24	NM_133939.1	ribosomal protein S24 (Rps24), transcript variant 1	0.61
Rps25	NM_146006.1	ribosomal protein S25 (Rps25)	0.45
Rps26	NM_206958.1	ribosomal protein S26 (Rps26)	0.40
Rps27a	NM_138680	ribosomal protein S27a (Rps27a), transcript variant 2	0.45
Rps27l	NM_178705.5	ribosomal protein S27-like (Rps27l)	0.55
Rps29	NM_016753.4	ribosomal protein S29 (Rps29)	0.53
Rps3	NM_016923.1		0.49
Rps3a	NM_025281.2	ribosomal protein S3a (Rps3a)	0.56
Rps4x	NM_145100.3	ribosomal protein S4, X-linked (Rps4x)	0.53
Rps5	NM_011942.1	ribosomal protein S5 (Rps5)	0.41
Rps6	NM_134147.3	ribosomal protein S6 (Rps6)	0.50
Rps6ka1	NM_019499.2	ribosomal protein S6 kinase polypeptide 1 (Rps6ka1)	0.61
Rps7	NM_025649.1	ribosomal protein S7 (Rps7)	0.48
Rps8	NM_019791.2	ribosomal protein S8 (Rps8)	0.49
Rps9	NM_023788.3	ribosomal protein S9 (Rps9)	0.62
Rrbp1	NM_026453.3	ribosome binding protein 1 (Rrbp1), transcript variant 2	0.23
Rrm1	NM_207237		0.33
Rrm2	NM_008549.1	ribonucleotide reductase M2 (Rrm2)	0.30
Rrp12	NM_027288.2	ribosomal RNA processing 12 homolog (S. cerevisiae) (Rrp12)	0.66
Rrp15	NM_026160.3	ribosomal RNA processing 15 homolog (S. cerevisiae) (Rrp15)	0.54
Rrp1b	NM_023138.3	ribosomal RNA processing 1 homolog B (S. cerevisiae) (Rrp1b)	0.63
Rrp9	NM_008928.3	RRP9, small subunit (SSU) processome component, homolog (yeast) (Rrp9)	0.63
Rsl1d1	NM_009157.2	ribosomal L1 domain containing 1 (Rsl1d1)	0.49
Rtkn2	NM_022012.3	rhotekin 2 (Rtkn2)	0.54
Rtn3	NM_138667.2		0.55
Rtn4rl1	NM_008696.2	reticulon 4 receptor-like 1 (Rtn4rl1)	0.57
Ruvbl1	NM_178684.5	RuvB-like protein 1 (Ruvbl1)	0.66
Ruvbl2	NM_172632.2	RuvB-like protein 2 (Ruvbl2)	0.53
Rwdd1	NM_027418.1	RWD domain containing 1 (Rwdd1)	0.58
Rybp	NM_011162.2	RING1 and YY1 binding protein (Rybp)	0.66
Ryk	NM_021921.2	receptor-like tyrosine kinase (Ryk), transcript variant 2	0.53
Saal1	NM_010838.2	PREDICTED: serum amyloid A-like 1, transcript variant 1 (Saal1)	0.63
Sacm1l	NM_010807.3	SAC1 (suppressor of actin mutations 1, homolog)-like (S. cerevisiae) (Sacm1l)	0.43
Sae1	NM_008641.2	SUMO1 activating enzyme subunit 1 (Sae1)	0.58
Sall3	NM_013592.2	sal-like 3 (Drosophila) (Sall3)	0.65
Sap30	NM_013595.2	sin3 associated polypeptide (Sap30)	0.42
Sart3	NM_020007.3	squamous cell carcinoma antigen recognized by T-cells 3 (Sart3)	0.62
Sc4mol	NM_153546.3	sterol-C4-methyl oxidase-like (Sc4mol)	0.31
Scd1	NM_026037.2	stearoyl-Coenzyme A desaturase 1 (Scd1)	0.52
scl0001118.1_0	NM_139295.2		0.57

scl0001284.1_18	NM_008562.3		0.61
scl0001849.1_2273	NM_027290.1		0.33
scl0001978.1_6	NM_008564.1		0.59
scl0002315.1_12	NM_008563.2		0.63
scl0002507.1_236	NM_008565.2		0.60
scl0002624.1_576	NM_008566.2		0.56
scl0002791.1_134	NM_008567.1		0.65
scl0004020.1_31	NM_008568.2		0.65
Scml4	NM_207010.1	sex comb on midleg-like 4 (Drosophila) (Scml4)	0.48
Scoc	NM_008618.2	short coiled-coil protein (Scoc), transcript variant 2	0.55
Scye1	NM_029696.3	small inducible cytokine subfamily E, member 1 (Scye1)	0.65
Sdc3	NM_008617.2	syndecan 3 (Sdc3)	0.35
Sdhb	NM_138596.1	succinate dehydrogenase complex, subunit B, iron sulfur (Ip) (Sdhb), nuclear gene encoding mitochondrial protein	0.45
Sdhd	NM_008581	succinate dehydrogenase complex, subunit D, integral membrane protein (Sdhd), nuclear gene encoding mitochondrial protein	0.60
Sdpr	NM_010790.2	serum deprivation response (Sdpr)	0.51
Sec11a	NM_008590.1	SEC11 homolog A (S. cerevisiae) (Sec11a)	0.64
Sec11c	NM_175224.4	SEC11 homolog C (S. cerevisiae) (Sec11c)	0.44
Sec13	NM_133719	SEC13 homolog (S. cerevisiae) (Sec13)	0.54
Sec61g	NM_021554.2	SEC61, gamma subunit (Sec61g)	0.57
Sec63	NM_001029890.1	SEC63-like (S. cerevisiae) (Sec63)	0.53
Seh1l	NM_008594	SEH1-like (S. cerevisiae (Seh1l), transcript variant 1	0.60
Sema3a	NM_001081279.1	sema domain, immunoglobulin domain (Ig), short basic domain, secreted, (semaphorin) 3A (Sema3a)	0.63
Sema5a	NM_024200.2		0.56
Sept2	NM_145486.4	septin 2 (Sept2)	0.60
Sept6	NM_010891.1	septin 6 (Sept6)	0.57
Sept9	NM_011889.2	septin 9 (Sept9)	0.13
Sept11	NM_019942.2	septin 11 (Sept11)	0.38
Serbp1	NM_001081259.1	Serpine1 mRNA binding protein 1 (Serbp1)	0.49
Serf2	NM_011844.3	small EDRK-rich factor 2 (Serf2)	0.61
Serpine2	NM_019946.3	serine (or cysteine) peptidase inhibitor, clade E, member 2 (Serpine2)	0.49
Set	NM_019394		0.60
Setd8	NM_026524.2	SET domain containing (lysine methyltransferase) 8 (Setd8)	0.66
Sf3a1	NM_010798.2	splicing factor 3a, subunit 1 (Sf3a1)	0.60
Sf3a3	XM_001000692.2	splicing factor 3a, subunit 3 (Sf3a3)	0.63
Sf3b3	NM_026472.3	splicing factor 3b, subunit 3 (Sf3b3)	0.51
Sf3b4	AK129109	splicing factor 3b, subunit 4 (Sf3b4)	0.51
Sf3b5	NM_021527.1		0.64
Sfrs1	NM_021462.3	splicing factor, arginine/serine-rich 1 (ASF/SF2) (Sfrs1), transcript variant 1	0.55
Sfrs10	NM_001039684.2	splicing factor, arginine/serine-rich 10 (transformer 2 homolog, Drosophila) (Sfrs10)	0.46
Sfrs11	NM_133241.2	splicing factor, arginine/serine-rich 11 (Sfrs11), transcript variant 1	0.48
Sfrs2	NM_010801.1	splicing factor, arginine/serine-rich 2 (SC-35) (Sfrs2)	0.39
Sfrs4	XM_485570	splicing factor, arginine/serine-rich 4 (SRp75) (Sfrs4)	0.64
Sfrs9	NM_019914.3	splicing factor, arginine/serine rich 9 (Sfrs9)	0.47
Sfxn1	NM_026178.2	sideroflexin 1 (Sfxn1)	0.60
Sgk1	NM_175217.6	serum/glucocorticoid regulated kinase 1 (Sgk1)	0.45
Sgk3	NM_008608.2	serum/glucocorticoid regulated kinase 3 (Sgk3), transcript variant 1	0.64
Sgol1	NM_008609.3	shugoshin-like 1 (S. pombe) (Sgol1)	0.51
Sgol2	NM_011846.4	shugoshin-like 2 (S. pombe) (Sgol2)	0.34
Sgpp1	NM_008610.2	sphingosine-1-phosphate phosphatase 1 (Sgpp1)	0.52
Sh3glb1	NM_010808.3	SH3-domain GRB2-like B1 (endophilin) (Sh3glb1)	0.65
Sh3glb2	NM_008615.1	SH3-domain GRB2-like endophilin B2 (Sh3glb2)	0.50
Sh3pxd2b	NM_029309.2	SH3 and PX domains 2B (Sh3pxd2b)	0.39
Shc1	NM_010820.2	src homology 2 domain-containing transforming protein C1 (Shc1)	0.66
Shmt1	NM_026758.3		0.38
Sipa1l2	NM_001081323.1	signal-induced proliferation-associated 1 like 2 (Sipa1l2)	0.66
Siva1	NM_025837.2	SIVA1, apoptosis-inducing factor (Siva1)	0.48
Skil	NM_026530.5	SKI-like (Skil), transcript variant 2	0.52
Skiv2l2	NM_019939.1	superkiller viralicidic activity 2-like 2 (S. cerevisiae) (Skiv2l2)	0.50
Skp2	NM_033564.2	S-phase kinase-associated protein 2 (p45) (Skp2), transcript variant 2	0.32
Slain2	NM_008624.3	SLAIN motif family, member 2 (Slain2)	0.65
Slc12a2	NM_018736.2	solute carrier family 12, member 2 (Slc12a2)	0.62
Slc19a2	NM_026154.1	solute carrier family 19 (thiamine transporter), member 2 (Slc19a2)	0.57
Slc1a4	NM_027204.2	solute carrier family 1 (glutamate/neutral amino acid transporter), member 4 (Slc1a4)	0.47
Slc25a11	AK003026	solute carrier family 25 (mitochondrial carrier oxoglutarate carrier), member 11 (Slc25a11), nuclear gene encoding mitochondrial protein	0.57

Slc25a19	NM_025301.1	solute carrier family 25 (mitochondrial thiamine pyrophosphate carrier), member 19 (Slc25a19), nuclear gene encoding mitochondrial protein	0.64
Slc25a25	NM_026310.3	solute carrier family 25 (mitochondrial carrier, phosphate carrier), member 25 (Slc25a25), nuclear gene encoding mitochondrial protein	0.27
Slc25a3	NM_025302.3	solute carrier family 25 (mitochondrial carrier, phosphate carrier), member 3 (Slc25a3)	0.53
Slc25a38	NM_025570.2	solute carrier family 25, member 38 (Slc25a38)	0.61
Slc25a39	NM_175001.2	solute carrier family 25, member 39 (Slc25a39)	0.43
Slc25a5	NM_011288.1	solute carrier family 25 (mitochondrial carrier, adenine nucleotide translocator), member 5 (Slc25a5), nuclear gene encoding mitochondrial protein	0.59
Slc29a1	NM_053159.3	solute carrier family 29 (nucleoside transporters), member 1 (Slc29a1)	0.53
Slc30a5	NM_027098.2	solute carrier family 30 (zinc transporter), member 5 (Slc30a5)	0.59
Slc30a7	NM_053162.2	solute carrier family 30 (zinc transporter), member 7 (Slc30a7)	0.65
Slc31a1	NM_025500.1	solute carrier family 31, member 1 (Slc31a1)	0.56
Slc35c2	NM_024177.3	solute carrier family 35, member C2 (Slc35c2)	0.53
Slc35e1	NM_010922.1	solute carrier family 35, member E1 (Slc35e1)	0.58
Slc35f3	NM_053164.3	solute carrier family 35, member F3 (Slc35f3)	0.51
Slc38a10	NM_029017.1	solute carrier family 38, member 10 (Slc38a10)	0.57
Slc39a6	NM_178603.4	solute carrier family 39 (metal ion transporter), member 6 (Slc39a6)	0.56
Slc39a7	NM_026851.2	solute carrier family 39 (zinc transporter), member 7 (Slc39a7), transcript variant 1	0.65
Slc41a2	NM_026744.3	solute carrier family 41, member 2 (Slc41a2)	0.52
Slc4a4	NM_025317.2	solute carrier family 4 (anion exchanger), member 4 (Slc4a4)	0.61
Slc4a7	NM_183086		0.44
Slc4a8	NM_025440.2	solute carrier family 4 (anion exchanger), member 8 (Slc4a8)	0.60
Slc6a1	NM_025878.1	solute carrier family 6 (neurotransmitter transporter, GABA), member 1 (Slc6a1)	0.64
Slc6a6	NM_026826.1	solute carrier family 6 (neurotransmitter transporter, taurine), member 6 (Slc6a6)	0.26
Slmap	NM_080452.2	sarcolemma associated protein (Slmap)	0.64
Slmo2	NM_024174.5	slowmo homolog 2 (Drosophila) (Slmo2)	0.46
Sltm	NM_026080.2	SAFB-like, transcription modulator (Sltm), transcript variant 1	0.66
Smad3	NM_025434.2		0.63
Smap2	NM_021556.3	stromal membrane-associated GTPase-activating protein 2 (Smap2)	0.62
Smc1a	NM_020560.1	structural maintenance of chromosomes 1A (Smc1a)	0.59
Smn1	NM_145573.2	survival motor neuron 1 (Smn1)	0.52
Smtn	NM_025305.1	smoothelin (Smtn)	0.33
Smyd2	NM_023514.3	SET and MYND domain containing 2 (Smyd2)	0.63
Snapc4	NM_023536.2	small nuclear RNA activating complex, polypeptide 4 (Snapc4)	0.49
Snca	AK031365	synuclein, alpha (Snca), transcript variant 2	0.49
Snora65	NM_144898.1	small nucleolar RNA, H/ACA box 65 (Snora65) on chromosome 2.	0.65
Snrpa	NM_010835.2	small nuclear ribonucleoprotein polypeptide A (Snrpa), transcript variant 2	0.59
Snrpb	NM_008630.2	small nuclear ribonucleoprotein B (Snrpb)	0.45
Snrpd1	NM_013603.1	small nuclear ribonucleoprotein D1 (Snrpd1)	0.48
Snrpd2	NM_011842.3	small nuclear ribonucleoprotein D2 (Snrpd2)	0.58
Snrpd3	NM_024433.1	small nuclear ribonucleoprotein D3 (Snrpd3)	0.57
Snrpf	NM_173013.2	PREDICTED: small nuclear ribonucleoprotein polypeptide F (Snrpf)	0.37
Snx9	NM_144941.2	sorting nexin 9 (Snx9)	0.61
Socs5		suppressor of cytokine signaling 5 (Socs5)	0.36
Sod2	NM_019758.2		0.56
Sorcs1		VPS10 domain receptor protein SORCS 1 (Sorcs1)	0.43
Sorcs2	NM_026002.4	sortilin-related VPS10 domain containing receptor 2 (Sorcs2)	0.59
Sox11			0.42
Sox5		SRY-box containing gene 5 (Sox5)	0.61
Spag5	NM_008638	sperm associated antigen 5 (Spag5)	0.22
Spag7	NM_181409.2	sperm associated antigen 7 (Spag7)	0.63
Spast	NM_172958.3	spastin (Spast)	0.61
Spata6	NM_023858.1	spermatogenesis associated 6 (Spata6)	0.63
Spc25	NM_013604.1	SPC25, NDC80 kinetochore complex component, homolog (S. cerevisiae) (Spc2	0.29
Spcs3	NM_138656.1	signal peptidase complex subunit 3 homolog (S. cerevisiae) (Spcs3)	0.48
Specc1	NM_024263.3	sperm antigen with calponin homology and coiled-coil domains 1 (Specc1)	0.45
Spg7	NM_016969.1	spastic paraplegia 7 homolog (human) (Spg7), nuclear gene encoding mitochondrial protein	0.64
Sphk1	NM_016776.2	sphingosine kinase 1 (Sphk1), transcript variant 1	0.47
Spna2	NM_008652.2		0.46
Spp1	NM_170671.1	secreted phosphoprotein 1 (Spp1)	0.26
Spred1	NM_010852.1		0.45
Spry4	NM_177369.3	sprouty homolog 4 (Drosophila) (Spry4)	0.31
Spsb4	NM_022410.1	splA/ryanodine receptor domain and SOCS box containing 4 (Spsb4)	0.23
Sqle	XM_977718.2	squalene epoxidase (Sqle)	0.50
Srebf2	NM_139300.3	sterol regulatory element binding factor 2 (Srebf2)	0.59

Srgap2	NM_010863.2	PREDICTED: SLIT-ROBO Rho GTPase activating protein 2, transcript variant 3 (Srgap2)	0.61
Srm	NM_010864.2	spermidine synthase (Srm)	0.41
Srpk1	NM_001039546.1	serine/arginine-rich protein specific kinase 1 (Srpk1)	0.58
Srpr	NM_017479.2	signal recognition particle receptor ('docking protein') (Srpr)	0.63
Srprb	NM_008665.3	signal recognition particle receptor, B subunit (Srprb)	0.64
Srrm2	NM_001024917.1	serine/arginine repetitive matrix 2 (Srrm2)	0.45
Ssbp4	AK050560		0.48
Ssna1	NM_026526.2	Sjogren's syndrome nuclear autoantigen 1 (Ssna1)	0.60
Ssr3	NM_008667.2	signal sequence receptor, gamma (Ssr3)	0.57
Ssrp1	NM_013608.2	structure specific recognition protein 1 (Ssrp1)	0.63
Sssca1	NM_001037098.1	Sjogren's syndrome/scleroderma autoantigen 1 homolog (human) (Sssca1)	0.53
Ssu72	NM_013792.1	Ssu72 RNA polymerase II CTD phosphatase homolog (yeast) (Ssu72)	0.66
St13	NM_053179.3	suppression of tumorigenicity 13 (St13)	0.61
St3gal3	NM_015781.3	ST3 beta-galactoside alpha-2,3-sialyltransferase 3 (St3gal3)	0.61
St6gal1	NM_021432.2	beta galactoside alpha 2,6 sialyltransferase 1 (St6gal1)	0.47
St6galnac5	NM_053089.2	ST6 (alpha-N-acetyl-neuraminyl-2,3-beta-galactosyl-1, 3)-N-acetylgalactosaminide alpha-2,6-sialyltransferase 5 (St6galnac5)	0.62
Stab1	NM_027350.2	stabilin 1 (Stab1)	0.37
Stag2	NM_016777.2	stromal antigen 2 (Stag2), transcript variant 2	0.65
Stambpl1	NM_026425.1	Stam binding protein like 1 (Stambpl1)	0.35
Stard4	NM_173437	StAR-related lipid transfer (START) domain containing 4 (Stard4)	0.40
Stau2	NM_008675.1	staufen (RNA binding protein) homolog 2 (Drosophila) (Stau2), transcript variant 3	0.65
Stc1	NM_007789.2	stanniocalcin 1 (Stc1)	0.35
Stim2	NM_146171.1	stromal interaction molecule 2 (Stim2)	0.62
Stip1	NM_178113.2	stress-induced phosphoprotein 1 (Stip1)	0.47
Stk17b	NM_144818.1	serine/threonine kinase 17b (apoptosis-inducing) (Stk17b)	0.38
Strap	NM_011986.2	serine/threonine kinase receptor associated protein (Strap)	0.58
Strn4	NM_010880.3	striatin, calmodulin binding protein 4 (Strn4), transcript variant 1	0.66
Stt3b	NM_021607.2	STT3, subunit of the oligosaccharyltransferase complex, homolog B (S. cerevisiae) (Stt3b)	0.66
Stx4a	NM_023294.1	syntaxin 4A (placental) (Stx4a)	0.53
Stxbp1	NM_022996.1	syntaxin binding protein 1 (Stxbp1)	0.48
Suclg2	NM_008681.2	succinate-Coenzyme A ligase, GDP-forming, beta subunit (Suclg2)	0.66
Sulf2	NM_013864	sulfatase 2 (Sulf2)	0.58
Sumo2	NM_008681	SMT3 suppressor of mif two 3 homolog 2 (yeast) (Sumo2)	0.53
Sumo3	NM_025551.3	SMT3 suppressor of mif two 3 homolog 3 (yeast) (Sumo3)	0.56
Supt3h	XM_001476048.1	suppressor of Ty 3 homolog (S. cerevisiae) (Supt3h)	0.64
Supv3l1	NM_025348.1	suppressor of var1, 3-like 1 (S. cerevisiae) (Supv3l1)	0.67
Suz12	NM_010886.1	suppressor of zeste 12 homolog (Drosophila) (Suz12)	0.50
Sv2a	NM_026614.2	synaptic vesicle glycoprotein 2 a (Sv2a)	0.39
Swap70	NM_025987.1	SWA-70 protein (Swap70)	0.62
Syde1	NM_026703.2	PREDICTED: synapse defective 1, Rho GTPase, homolog 1 (C. elegans) (Syde1)	0.63
Syncrip	NM_025358.2	synaptotagmin binding, cytoplasmic RNA interacting protein (Syncrip), transcript variant 2	0.59
Tada1l	XM_128594.4	transcriptional adaptor 1 (HFI1 homolog, yeast) like (Tada1l)	0.59
Taf10	NM_025597.2	TAF10 RNA polymerase II, TATA box binding protein (TBP)-associated factor (Taf10)	0.52
Taf12	NM_025316.2		0.56
Taf9	NM_001033305.2	TAF9 RNA polymerase II, TATA box binding protein (TBP)-associated factor (Taf9), transcript variant 1	0.49
Tagap	NM_026061.2	T-cell activation Rho GTPase-activating protein (Tagap)	0.59
Tagln2	NM_025523.1	transgelin 2 (Tagln2)	0.51
Tanc1	NM_024220.1	tetratricopeptide repeat, ankyrin repeat and coiled-coil containing 1 (Tanc1)	0.46
Tank	NM_153064.4	TRAF family member-associated Nf-kappa B activator (Tank)	0.66
Tbc1d1	NM_026688.2	TBC1 domain family, member 1 (Tbc1d1)	0.40
Tbca	NM_010887.1	tubulin cofactor a (Tbca)	0.60
Tbrg4	NM_029272.3	transforming growth factor beta regulated gene 4 (Tbrg4)	0.39
Tceal8	NM_144870.4	transcription elongation factor A (SII)-like 8 (Tceal8)	0.66
Tcerg1	NM_133666.2	transcription elongation regulator 1 (CA150) (Tcerg1)	0.52
Tcf12	NM_028388.1	transcription factor 12 (Tcf12)	0.52
Tcf4	NM_026267.1	transcription factor 4 (Tcf4), transcript variant 1	0.60
Tcn2	NM_008682	transcobalamin 2 (Tcn2)	0.64
Tcp1	NM_031881.1	t-complex protein 1 (Tcp1)	0.37
Tead2	NM_017464.2	TEA domain family member 2 (Tead2)	0.45
Tfdp1	XM_973899.1		0.48
Tfg	NM_011848.2	Trk-fused gene (Tfg)	0.61
Tfrc	NM_177898.4	transferrin receptor (Tfrc)	0.44

Thap4	NM_145138.1	THAP domain containing 4 (Thap4)	0.66
Them2	NM_016701.3	thioesterase superfamily member 2 (Them2)	0.65
Thoc4	NM_001081324.1	THO complex 4 (Thoc4)	0.35
Thoc7	NM_010893.2	THO complex 7 homolog (Drosophila) (Thoc7), transcript variant 1	0.48
Thumpd1	NM_021360	THUMP domain containing 1 (Thumpd1)	0.58
Thyn1	NM_010901.2	thymocyte nuclear protein 1 (Thyn1)	0.54
Timeless	NM_177176.2	timeless homolog (Drosophila) (Timeless), transcript variant 2	0.41
Timm10	NM_008687.2	translocase of inner mitochondrial membrane 10 homolog (yeast) (Timm10)	0.56
Timm17a	NM_030612.2	translocase of inner mitochondrial membrane 17a (Timm17a), nuclear gene enc	0.44
Timm50	NM_019867	translocase of inner mitochondrial membrane 50 homolog (yeast) (Timm50), nuc	0.62
Timm8a1	NM_025811.1	translocase of inner mitochondrial membrane 8 homolog a1 (yeast) (Timm8a1)	0.49
Timm8b	NM_008695.2	translocase of inner mitochondrial membrane 8 homolog b (yeast) (Timm8b)	0.50
Timm9	NM_025391.1	translocase of inner mitochondrial membrane 9 homolog (yeast) (Timm9), nuclear gene encoding mitochondrial protein, transcript variant 1	0.54
Timp1	NM_025623.2	tissue inhibitor of metalloproteinase 1 (Timp1), transcript variant 2	0.31
Timp4	AK036043	tissue inhibitor of metalloproteinase 4 (Timp4)	0.22
Tipin	NM_029891.2	timeless interacting protein (Tipin)	0.41
Tk1	NM_010919.2	thymidine kinase 1 (Tk1)	0.19
Tlcd1	NM_008704.2	TLC domain containing 1 (Tlcd1)	0.50
Tle2	NM_008705.4	transducin-like enhancer of split 2, homolog of Drosophila E(spl) (Tle2)	0.57
Tle6	NM_019731.1	transducin-like enhancer of split 6, homolog of Drosophila E(spl) (Tle6)	0.17
Tlk1	NM_080637.3	tousled-like kinase 1 (Tlk1)	0.61
Tmed3	NM_138314.2	transmembrane emp24 domain containing 3 (Tmed3)	0.54
Tmed9	NM_026393.1	transmembrane emp24 protein transport domain containing 9 (Tmed9)	0.59
Tmem109	NM_008707.3	transmembrane protein 109 (Tmem109)	0.50
Tmem11	NM_021303.2	transmembrane protein 11 (Tmem11)	0.49
Tmem126a	NM_153570.2	transmembrane protein 126A (Tmem126a)	0.49
Tmem132a	NM_138747.1	transmembrane protein 132A (Tmem132a)	0.39
Tmem147	NM_001008421.1	transmembrane protein 147 (Tmem147)	0.66
Tmem14c	NM_018868.2	transmembrane protein 14C (Tmem14c)	0.57
Tmem176a	NM_024193.2	transmembrane protein 176A (Tmem176a)	0.23
Tmem176b	NM_026578.2	transmembrane protein 176B (Tmem176b)	0.29
Tmem184b	NM_026631.3	transmembrane protein 184b (Tmem184b)	0.56
Tmem185b	NM_025403.2	transmembrane protein 185B (Tmem185b)	0.60
Tmem199	NM_153057.3	transmembrane protein 199 (Tmem199)	0.58
Tmem41a	NM_020043.2	transmembrane protein 41a (Tmem41a)	0.56
Tmem44	NM_028995.3	transmembrane protein 44 (Tmem44)	0.43
Tmem48	NM_023409.4	transmembrane protein 48 (Tmem48)	0.51
Tmem49	NM_008722.1	transmembrane protein 49 (Tmem49)	0.41
Tmem55a	NM_008723.1	transmembrane protein 55A (Tmem55a)	0.64
Tmem55b	NR_002702.1	transmembrane protein 55b (Tmem55b)	0.48
Tmem56	NM_009145.1	transmembrane protein 56 (Tmem56)	0.62
Tmem63b	NM_008730.2	transmembrane protein 63b (Tmem63b)	0.51
Tmem68	NM_020282.2	transmembrane protein 68 (Tmem68)	0.48
Tmem70	NM_001025587.1	transmembrane protein 70 (Tmem70), transcript variant 1	0.61
Tmem88	NM_010150.2	transmembrane protein 88 (Tmem88)	0.56
Tmem97	NM_025980.2	transmembrane protein 97 (Tmem97)	0.27
Tmpo	NM_144847.1	thymopoietin (Tmpo), transcript variant 6	0.62
Tmsb10	NM_176930.3	thymosin, beta 10 (Tmsb10)	0.63
Tmsb4x	NM_146150.2	thymosin, beta 4, X chromosome (Tmsb4x)	0.58
Tnc	NM_134122.2	tenascin C (Tnc)	0.04
Tnfaip8	NM_153529.1	tumor necrosis factor, alpha-induced protein 8 (Tnfaip8)	0.59
Tnfrsf10b	NM_008737.1	tumor necrosis factor receptor superfamily, member 10b (Tnfrsf10b)	0.62
Tnfrsf12a	NM_172544.2	tumor necrosis factor receptor superfamily, member 12a (Tnfrsf12a)	0.44
Tnfrsf21	NM_016710.2	tumor necrosis factor receptor superfamily, member 21 (Tnfrsf21)	0.36
Tomm22	NM_010941.3	translocase of outer mitochondrial membrane 22 homolog (yeast) (Tomm22), nuclear gene encoding mitochondrial protein	0.55
Tomm40l	NM_011732	translocase of outer mitochondrial membrane 40 homolog-like (yeast) (Tomm40l), nuclear gene encoding mitochondrial protein	0.54
Tomm70a	NM_198326.2	translocase of outer mitochondrial membrane 70 homolog A (yeast) (Tomm70a)	0.66
Top2a	NM_008741.1	topoisomerase (DNA) II alpha (Top2a)	0.16
Tpi1	NM_015807.1	triosephosphate isomerase 1 (Tpi1)	0.40
Tpm3	NM_027289.1	tropomyosin 3, gamma (Tpm3)	0.39
Tpm4	NM_010946.2	tropomyosin 4 (Tpm4)	0.46
Tpr	NM_001025074.1	translocated promoter region (Tpr)	0.64
Tpst1	NM_008747.2	protein-tyrosine sulfotransferase 1 (Tpst1), transcript variant 2	0.61
Tpx2	NM_001004363.1	TPX2, microtubule-associated protein homolog (Xenopus laevis) (Tpx2)	0.31
Traf3	NM_028778.3	Tnf receptor-associated factor 3 (Traf3), transcript variant 1	0.58
Traf4	NM_011955.1	Tnf receptor associated factor 4 (Traf4)	0.49
Trak2	NM_008749.1	trafficking protein, kinesin binding 2 (Trak2)	0.52
Tram1	NM_010948.1	translocating chain-associating membrane protein 1 (Tram1)	0.55

Trap1	NM_001031664.1	TNF receptor-associated protein 1 (Trap1)	0.64
Trappc1	NM_033080.2	trafficking protein particle complex 1 (Trappc1)	0.63
Trappc4	NM_026623.3	trafficking protein particle complex 4 (Trappc4)	0.58
Trf	NM_016918.2	transferrin (Trf)	0.13
Trib2	NM_172288.1		0.31
Trim27	NM_021512.2	tripartite motif-containing 27 (Trim27)	0.65
Trim28	XM_133073.7	tripartite motif protein 28 (Trim28)	0.44
Trim59	NM_018815.1	tripartite motif-containing 59 (Trim59)	0.49
Trim62	NM_145706.1	tripartite motif-containing 62 (Trim62)	0.43
Trim8	NM_053074.1	tripartite motif protein 8 (Trim8)	0.43
Trio	NM_001002929.3		0.57
Trip12	NM_172394.2	thyroid hormone receptor interactor 12 (Trip12)	0.58
Troap	NM_030250.1	PREDICTED: trophinin associated protein (Troap)	0.30
Trp53	NM_001042652.1	transformation related protein 53 (Trp53)	0.55
Trp53i11	NM_008750.4	transformation related protein 53 inducible protein 11 (Trp53i11)	0.32
Trp53inp2	NM_008751.2	transformation related protein 53 inducible nuclear protein 2 (Trp53inp2)	0.42
Tsen15	NM_019761.4	tRNA splicing endonuclease 15 homolog (S. cerevisiae) (Tsen15)	0.67
Tsfm	NM_016978.1	Ts translation elongation factor, mitochondrial (Tsfm), nuclear gene encoding mitochondrial protein	0.65
Tsg101	NM_026950.3	tumor susceptibility gene 101 (Tsg101)	0.62
Tspan12	NM_011858.3	tetraspanin 12 (Tspan12)	0.57
Tssc4	NM_010956.3	tumor-suppressing subchromosomal transferable fragment 4 (Tssc4), transcript variant 2	0.58
Ttc3	NM_008760.2	tetratricopeptide repeat domain 3 (Ttc3)	0.66
Ttc7b	NM_139144.2	PREDICTED: tetratricopeptide repeat domain 7B, transcript variant 1 (Ttc7b)	0.51
Ttc9c	NM_025942.2	tetratricopeptide repeat domain 9C (Ttc9c)	0.63
Ttyh3	NM_173777.2	tweety homolog 3 (Drosophila) (Ttyh3)	0.46
Tuba6	NM_172907.2		0.36
Tubb5	NM_177068.3	tubulin, beta 5 (Tubb5)	0.39
Tubb6	NM_016968.4	tubulin, beta 6 (Tubb6)	0.26
Tubg1	NM_153122.2	tubulin, gamma 1 (Tubg1)	0.43
Tufm	NM_010098.2	Tu translation elongation factor, mitochondrial (Tufm), nuclear gene encoding mitochondrial protein	0.56
Txn1	NM_011014.2	thioredoxin 1 (Txn1)	0.28
Txndc12	NM_175423.3	thioredoxin domain containing 12 (endoplasmic reticulum) (Txndc12)	0.47
Txnl4	NM_011959.2		0.58
Txnl4a	NM_019716.1	thioredoxin-like 4A (Txnl4a), transcript variant 2	0.63
Tyms	NM_025661.3	thymidylate synthase (Tyms)	0.34
Tyms-ps	NM_027881.1	thymidylate synthase, pseudogene (Tyms-ps), non-coding RNA.	0.37
U2af1	NM_024289		0.63
U2af2	NM_145525.2	U2 small nuclear ribonucleoprotein auxiliary factor (U2AF) 2 (U2af2)	0.55
Uaca	NM_175489.3	uveal autoantigen with coiled-coil domains and ankyrin repeats (Uaca)	0.55
Uba1	NM_011019.1	ubiquitin-like modifier activating enzyme 1 (Uba1)	0.53
Uba2	NM_172416.2	ubiquitin-like modifier activating enzyme 2 (Uba2)	0.53
Ubap2	NM_024188.5	ubiquitin-associated protein 2 (Ubap2)	0.62
Ube2f	NM_130885.2	ubiquitin-conjugating enzyme E2F (putative) (Ube2f)	0.50
Ube2g1	NM_175116.2	ubiquitin-conjugating enzyme E2G 1 (UBC7 homolog, C. elegans) (Ube2g1)	0.63
Ube2k	NM_011032.2	ubiquitin-conjugating enzyme E2K (UBC1 homolog, yeast) (Ube2k)	0.59
Ube2m	NM_011119.3	ubiquitin-conjugating enzyme E2M (UBC12 homolog, yeast) (Ube2m)	0.60
Ube2q1	NM_130881.2	ubiquitin-conjugating enzyme E2Q (putative) 1 (Ube2q1)	0.63
Ube2t	NM_027032.2	ubiquitin-conjugating enzyme E2T (putative) (Ube2t)	0.58
Ube3c	NM_001081170.1	ubiquitin protein ligase E3C (Ube3c)	0.64
Ubfd1	NM_008812.1	ubiquitin family domain containing 1 (Ubfd1)	0.66
Ubr7	NM_013625.1	ubiquitin protein ligase E3 component n-recognin 7 (putative) (Ubr7)	0.58
Uchl5	NM_008776.1	ubiquitin carboxyl-terminal esterase L5 (Uchl5)	0.54
Uck2	NM_027470.3	uridine-cytidine kinase 2 (Uck2)	0.35
Ugcg	XM_974900.1		0.53
Uhrf1	NM_023245.3	ubiquitin-like, containing PHD and RING finger domains, 1 (Uhrf1)	0.12
Uhrf2	NM_023792.1	ubiquitin-like, containing PHD and RING finger domains 2 (Uhrf2)	0.61
Ung	NM_145962.2	uracil DNA glycosylase (Ung), transcript variant 2	0.37
Upp1	NM_027995.2	uridine phosphorylase 1 (Upp1)	0.23
Uqcr	NM_007415.2	ubiquinol-cytochrome c reductase (6.4kD) subunit (Uqcr)	0.66
Uqcrc1	NM_054056.2	ubiquinol-cytochrome c reductase core protein 1 (Uqcrc1)	0.54
Uqcrc2	NM_023209.1	ubiquinol cytochrome c reductase core protein 2 (Uqcrc2)	0.62
Uqcrh	NM_001081251.1	ubiquinol-cytochrome c reductase hinge protein (Uqcrh)	0.53
Usp1	NM_011865.3	ubiquitin specific peptdiase 1 (Usp1)	0.47
Usp39	NM_011042.1	ubiquitin specific peptidase 39 (Usp39)	0.63
Uspl1	NM_021567.2	ubiquitin specific peptidase like 1 (Uspl1)	0.62
Utp11l	NM_011043.3	UTP11-like, U3 small nucleolar ribonucleoprotein, (yeast) (Utp11l)	0.55
Uxt	NM_001013753.1	ubiquitously expressed transcript (Uxt)	0.49

Vamp3	NM_018764.1	vesicle-associated membrane protein 3 (Vamp3)	0.60
Vapa	NM_001081377.1	vesicle-associated membrane protein, associated protein A (Vapa)	0.60
Vapb	NM_177546.2	vesicle-associated membrane protein, associated protein B and C (Vapb)	0.58
Vars	NM_024229.2	valyl-tRNA synthetase (Vars), nuclear gene encoding mitochondrial protein	0.46
Vav3	NM_001033313.3	vav 3 oncogene (Vav3), transcript variant 1	0.28
Vbp1	NM_026549.3	von Hippel-Lindau binding protein 1 (Vbp1)	0.57
Vdac1	NM_008808	voltage-dependent anion channel 1 (Vdac1)	0.54
Vdac2	NM_026840.2	voltage-dependent anion channel 2 (Vdac2)	0.26
Vdac3	NM_024221.3	voltage-dependent anion channel 3 (Vdac3)	0.57
Vegfa	NM_007952.2	vascular endothelial growth factor A (Vegfa), transcript variant 2	0.40
Vegfb	NM_028295.1	vascular endothelial growth factor B (Vegfb)	0.54
Vegfc	NM_145978.1	vascular endothelial growth factor C (Vegfc)	0.56
Vkorc1	NM_019417.2	vitamin K epoxide reductase complex, subunit 1 (Vkorc1)	0.64
Vkorc1l1	NM_019808.1	vitamin K epoxide reductase complex, subunit 1-like 1 (Vkorc1l1), transcript variant 1	0.48
Vldlr	NM_026131	very low density lipoprotein receptor (Vldlr)	0.45
Vps29	NM_178939.2	vacuolar protein sorting 29 (S. pombe) (Vps29)	0.51
Vps36	NM_019501.3	vacuolar protein sorting 36 (yeast) (Vps36)	0.29
Vps54	NM_020271.3	vacuolar protein sorting 54 (yeast) (Vps54)	0.57
Vrk1	NM_018884.1		0.44
Wasf2	XM_001481302.1		0.52
Wbp11	NM_008556.1	WW domain binding protein 11 (Wbp11)	0.57
Wbscr17	NM_023324.2	Williams-Beuren syndrome chromosome region 17 homolog (human) (Wbscr17)	0.53
Wdr43	NM_033602	PREDICTED: WD repeat domain 43, transcript variant 9 (Wdr43)	0.65
Wdr51b	NM_011070.3	WD repeat domain 51B (Wdr51b)	0.61
Wdr74	NM_001013369.1	WD repeat domain 74 (Wdr74)	0.60
Wdr75	NM_008825.3	WD repeat domain 75 (Wdr75)	0.55
Wdr76	NM_173019.5	WD repeat domain 76 (Wdr76)	0.54
Wdr82	NM_011072.3	WD repeat domain containing 82 (Wdr82)	0.62
Wdr89	NM_023418.2	PREDICTED: WD repeat domain 89, transcript variant 2 (Wdr89)	0.56
Wdsof1	NM_028273.1	WD repeats and SOF domain containing 1 (Wdsof1)	0.66
Whsc1	NM_008828.2		0.47
Wsb2	NM_025396.3	WD repeat and SOCS box-containing 2 (Wsb2)	0.34
Wscd1	NM_025954.3	WSC domain containing 1 (Wscd1)	0.41
Wwp1	NM_133757.2	WW domain containing E3 ubiquitin protein ligase 1 (Wwp1)	0.65
Wwtr1	NM_007531.2	WW domain containing transcription regulator 1 (Wwtr1)	0.60
Xbp1	NM_018774.3	X-box binding protein 1 (Xbp1)	0.46
Xpnpep1	NM_025408.2	X-prolyl aminopeptidase (aminopeptidase P) 1, soluble (Xpnpep1)	0.54
Xpo6	NM_024250.3	exportin 6 (Xpo6)	0.64
Xrcc6	NM_028716.1	X-ray repair complementing defective repair in Chinese hamster cells 6 (Xrcc6)	0.50
Yeats4	NM_172674.2	YEATS domain containing 4 (Yeats4)	0.58
Ywhab	NM_026737.3	tyrosine 3-monooxygenase/tryptophan 5-monooxygenase activation protein, beta polypeptide (Ywhab)	0.58
Ywhag	NM_027642.1	tyrosine 3-monooxygenase/tryptophan 5-monooxygenase activation protein, gamma polypeptide (Ywhag)	0.47
Ywhah	NM_011079.2	tyrosine 3-monooxygenase/tryptophan 5-monooxygenase activation protein, eta polypeptide (Ywhah)	0.35
Ywhaq	NM_009344.1	tyrosine 3-monooxygenase/tryptophan 5-monooxygenase activation protein, theta polypeptide (Ywhaq)	0.62
Zbtb7c	NM_172992.2	zinc finger and BTB domain containing 7C (Zbtb7c)	0.54
Zbtb8os	NM_008835.1	zinc finger and BTB domain containing 8 opposite strand (Zbtb8os)	0.63
Zc3hc1	NM_001001983.1	zinc finger, C3HC type 1 (Zc3hc1)	0.62
Zcrb1	NM_146194	zinc finger CCHC-type and RNA binding motif 1 (Zcrb1)	0.63
Zdhhc21	NM_172453.1	zinc finger, DHHC domain containing 21 (Zdhhc21)	0.55
Zdhhc6	NM_011081.2	zinc finger, DHHC domain containing 6 (Zdhhc6), transcript variant 2	0.62
Zeb1	NM_029406.1	zinc finger E-box binding homeobox 1 (Zeb1)	0.31
Zfp106	NM_178149.4	zinc finger protein 106 (Zfp106)	0.63
Zfp131	NM_054051.1	zinc finger protein 131 (Zfp131)	0.63
Zfp148	NM_019640.4	zinc finger protein 148 (Zfp148)	0.58
Zfp180	NM_011256.1		0.65
Zfp207	NM_145131.1	zinc finger protein 207 (Zfp207)	0.62
Zfp330	NM_008853.1	zinc finger protein 330 (Zfp330)	0.54
Zfp365	NM_144859.2	zinc finger protein 365 (Zfp365)	0.41
Zfp518b	NM_013630.2	zinc finger protein 518B (Zfp518b)	0.63
Zfp710	NM_008862.3	zinc finger protein 710 (Zfp710)	0.48
Zfp770	XM_131934.4	zinc finger protein 770 (Zfp770)	0.51
Znrd1	NM_023058.3	zinc ribbon domain containing, 1 (Znrd1)	0.58
Znrf2	NM_178654.3	zinc and ring finger 2 (Znrf2)	0.26
Zswim6	NM_175464.2		0.60

Zwint	NM_023196.2		0.62
Zyx	NM_013737.2	zyxin (Zyx)	0.54
