# Supplementary material for: miR-30 Family miRNAs Mediate the Effect of Chronic Social Defeat Stress on Hippocampal Neurogenesis in Mouse Depression Model
Source: Front Mol Neurosci. 2019 Aug 8;12:188. doi: 10.3389/fnmol.2019.00188 (PMC6694739; doi:10.3389/fnmol.2019.00188)
Supplement: TABLE S9 — The list comprises of genes which demonstrated increased expression (fold change ≥1.5 and p ≤ 0.05) in the differentiated cells from the late phase (day 7) when compared with the proliferating neurospheres. [file Table_9.pdf]

**Table S9. List of genes, which demonstrated increased expression (fold change ≥ 1.5 and p ≤ 0.05) in the differentiated cells from the late phase (day 7) when compared with the proliferating neurospheres**

| Gene Symbol | Accession No. | Gene Definition | Fold Change |
|---|---|---|---|
| *0610010D24Rik* | NM_026681 | | 1.59 |
| *1110012J17Rik* | NM_172963.3 | RIKEN cDNA 1110012J17 gene (1110012J17Rik) | 1.90 |
| *1110017I16Rik* | NM_026754.2 | RIKEN cDNA 1110017I16 gene (1110017I16Rik), transcript variant 1 | 9.28 |
| *1110036D12Rik* | | | 2.25 |
| *1190002N15Rik* | XM_147036 | | 1.53 |
| *1190005I06Rik* | NM_197988.1 | RIKEN cDNA 1190005I06 gene (1190005I06Rik) | 2.64 |
| *1190007F08Rik* | NM_001033144.1 | RIKEN cDNA 1190007F08 gene (1190007F08Rik) | 2.68 |
| *1300010F03Rik* | XM_001002205.2 | PREDICTED: RIKEN cDNA 1300010F03 gene (1300010F03Rik) | 1.55 |
| *1500015O10Rik* | NM_024283.2 | RIKEN cDNA 1500015O10 gene (1500015O10Rik) | 2.92 |
| *1500031L02Rik* | NM_025892.1 | RIKEN cDNA 1500031L02 gene (1500031L02Rik) | 1.51 |
| *1700003E16Rik* | XM_001478332.1 | PREDICTED: RIKEN cDNA 1700003E16 gene, transcript variant 1 (1700003E16Rik) | 2.08 |
| *1700007K13Rik* | NM_027040.1 | RIKEN cDNA 1700007K13 gene (1700007K13Rik) | 4.02 |
| *1700009P17Rik* | NM_001081275.1 | RIKEN cDNA 1700009P17 gene (1700009P17Rik) | 2.09 |
| *1700016J18Rik* | XM_994504.1 | PREDICTED: RIKEN cDNA 1700016J18 gene (1700016J18Rik) | 3.01 |
| *1700019L03Rik* | NM_025619.1 | RIKEN cDNA 1700019L03 gene (1700019L03Rik) | 1.59 |
| *1700025K23Rik* | NM_183254.1 | RIKEN cDNA 1700025K23 gene (1700025K23Rik) | 1.95 |
| *1700027A23Rik* | NM_029604.2 | RIKEN cDNA 1700027A23 gene (1700027A23Rik) | 1.58 |
| *1700027N10Rik* | NM_029338.3 | RIKEN cDNA 1700027N10 gene (1700027N10Rik) | 3.65 |
| *1700029J07Rik* | NM_001033148.2 | RIKEN cDNA 1700029J07 gene (1700029J07Rik) | 2.87 |
| *1700073K01Rik* | NM_026626 | | 1.76 |
| *1700084C01Rik* | NM_001033185.2 | RIKEN cDNA 1700084C01 gene (1700084C01Rik) | 5.74 |
| *1700088E04Rik* | NM_138581.2 | RIKEN cDNA 1700088E04 gene (1700088E04Rik) | 3.77 |
| *1810007P19Rik* | NM_172701.2 | RIKEN cDNA 1810007P19 gene (1810007P19Rik) | 1.78 |
| *1810013B01Rik* | NM_029631 | | 2.32 |
| *2010001J22Rik* | NM_001013022.1 | RIKEN cDNA 2010001J22 gene (2010001J22Rik) | 1.51 |
| *2010004M13Rik* | | | 2.35 |
| *2010005O13Rik* | NM_145512 | | 1.54 |
| *2310001H12Rik* | XM_901486.2 | PREDICTED: RIKEN cDNA 2310001H12 gene, transcript variant 21 (2310001H12Rik) | 1.62 |
| *2310007A19Rik* | NM_025506.2 | RIKEN cDNA 2310007A19 gene (2310007A19Rik) | 2.49 |
| *2310009E04Rik* | XM_131496.4 | | 1.74 |
| *2310014G06Rik* | NM_001082975.1 | RIKEN cDNA 2310014G06 gene (2310014G06Rik) | 1.66 |
| *2310022B05Rik* | NM_175149.3 | RIKEN cDNA 2310022B05 gene (2310022B05Rik) | 3.15 |
| *2310040A07Rik* | XM_912979.2 | PREDICTED: RIKEN cDNA 2310040A07 gene (2310040A07Rik) | 1.83 |
| *2310043N10Rik* | NR_003513.2 | RIKEN cDNA 2310043N10 gene (2310043N10Rik), non-coding RNA. | 3.26 |
| *2310061J03Rik* | XM_148411.1 | | 1.98 |
| *2310079P10Rik* | | | 1.96 |
| *2410001C21Rik* | NM_025542.1 | RIKEN cDNA 2410001C21 gene (2410001C21Rik) | 1.69 |
| *2410025L10Rik* | NM_028596.2 | RIKEN cDNA 2410025L10 gene (2410025L10Rik) | 1.83 |
| *2410187C16Rik* | NM_029734.1 | RIKEN cDNA 2410187C16 gene (2410187C16Rik) | 1.58 |
| *2610035D17Rik* | XM_902349.2 | PREDICTED: RIKEN cDNA 2610035D17 gene (2610035D17Rik) | 1.97 |
| *2610204M08Rik* | NM_198411.2 | RIKEN cDNA 2610204M08 gene (2610204M08Rik) | 1.51 |
| *2610524A10Rik* | NM_028258.1 | | 1.83 |
| *2700046G09Rik* | XR_035347.1 | PREDICTED: RIKEN cDNA 2700046G09 gene (2700046G09Rik), misc RNA. | 1.53 |
| *2700050C19Rik* | | | 1.83 |
| *2700060E02Rik* | NM_026528.3 | RIKEN cDNA 2700060E02 gene (2700060E02Rik) | 3.78 |
| *2810003C17Rik* | NM_145144.1 | RIKEN cDNA 2810003C17 gene (2810003C17Rik) | 3.75 |
| *2810029C07Rik* | | | 1.74 |
| *2810441K11Rik* | NM_026798.1 | RIKEN cDNA 2810441K11 gene (2810441K11Rik) | 1.83 |
| *2810459M11Rik* | XM_484890 | | 2.19 |
| *2900011O08Rik* | NM_144518.3 | RIKEN cDNA 2900011O08 gene (2900011O08Rik) | 2.56 |
| *2900026A02Rik* | NM_172884 | | 1.54 |
| *3010015F07Rik* | AK076147 | | 1.56 |
| *3100002J23Rik* | XM_001002726.1 | PREDICTED: RIKEN cDNA 3100002J23 gene, transcript variant 2 (3100002J23Rik) | 5.16 |
| *3110018K01Rik* | | | 2.48 |
| *3110023G01Rik* | AK014074 | | 1.63 |
| *4432405B04Rik* | NM_026486.1 | RIKEN cDNA 4432405B04 gene (4432405B04Rik) | 1.97 |
| *4631416L12Rik* | NM_001081295.1 | RIKEN cDNA 4631416L12 gene (4631416L12Rik) | 1.82 |
| *4632408A20Rik* | XM_126946.2 | | 1.57 |
| *4832420L08Rik* | AK029315 | | 1.97 |
| *4833409N03Rik* | | | 2.40 |
| *4833414E09Rik* | NM_027845.1 | | 1.69 |
| *4833420G17Rik* | NM_026127.3 | RIKEN cDNA 4833420G17 gene (4833420G17Rik) | 1.57 |
| *4833441J24Rik* | NM_133923.2 | | 3.13 |

| | | | |
|---|---|---|---|
| *4921530F17Rik* | AK029558 | | 1.61 |
| *4922501L14Rik* | XM_001481326.1 | PREDICTED: RIKEN cDNA 4922501L14 gene (4922501L14Rik) | 1.71 |
| *4930402H24Rik* | AK015050 | | 2.74 |
| *4930438O05Rik* | NM_027507.1 | | 1.77 |
| *4930455F23Rik* | NM_029115.1 | RIKEN cDNA 4930455F23 gene (4930455F23Rik) | 1.91 |
| *4930528G09Rik* | XM_355576.1 | | 1.78 |
| *4930533K18Rik* | | | 2.09 |
| *4930570C03Rik* | NM_026353.2 | RIKEN cDNA 4930570C03 gene (4930570C03Rik) | 1.85 |
| *4931426K16Rik* | XM_357260.1 | | 1.82 |
| *4932425I24Rik* | NM_001081025.1 | RIKEN cDNA 4932425I24 gene (4932425I24Rik) | 3.18 |
| *4932441K18Rik* | NM_178935.2 | RIKEN cDNA 4932441K18 gene (4932441K18Rik) | 1.66 |
| *4932443I19Rik* | XM_620624.3 | PREDICTED: RIKEN cDNA 4932443I19 gene, transcript variant 1 (4932443I19Rik) | 2.07 |
| *4933407L21Rik* | XM_129929.1 | | 4.03 |
| *4933421G18Rik* | NM_027707 | | 2.14 |
| *4933428D01Rik* | XM_127662.2 | | 1.55 |
| *4933434I06Rik* | NM_027728.2 | RIKEN cDNA 4933434I06 gene (4933434I06Rik) | 5.62 |
| *4933439C20Rik* | NM_001004146.1 | RIKEN cDNA 4933439C20 gene (4933439C20Rik) | 2.14 |
| *5031425E22Rik* | XM_149592.1 | | 1.70 |
| *5031428M13Rik* | AK030317 | | 2.06 |
| *5031436O03Rik* | | | 1.54 |
| *5031439G07Rik* | XM_128281 | | 1.77 |
| *5033414K04Rik* | NM_001003948.1 | RIKEN cDNA 5033414K04 gene (5033414K04Rik) | 2.06 |
| *5330403J18Rik* | AK030367 | | 2.57 |
| *5330431K02Rik* | NM_026606.1 | | 1.71 |
| *5430405G05Rik* | AK077378 | | 2.01 |
| *5430434G16Rik* | | | 1.67 |
| *5430435G22Rik* | NM_145509.1 | RIKEN cDNA 5430435G22 gene (5430435G22Rik) | 1.52 |
| *5730402C02Rik* | NM_027442 | | 2.45 |
| *5730410E15Rik* | NM_178765.3 | RIKEN cDNA 5730410E15 gene (5730410E15Rik), transcript variant c | 2.22 |
| *5830407P18Rik* | XM_147876.1 | | 1.59 |
| *6030410I10Rik* | AK031346 | | 2.00 |
| *6230400G14Rik* | | | 1.79 |
| *6330403K07Rik* | NM_134022.2 | RIKEN cDNA 6330403K07 gene (6330403K07Rik) | 2.21 |
| *6330405H19* | XM_358637.1 | | 1.70 |
| *6330414G02Rik* | | | 9.37 |
| *6330505N24Rik* | NM_001033301.2 | RIKEN cDNA 6330505N24 gene (6330505N24Rik) | 2.08 |
| *6430537H07Rik* | NM_178689.3 | RIKEN cDNA 6430537H07 gene (6430537H07Rik) | 2.28 |
| *6430548M08Rik* | NM_172286 | | 2.62 |
| *7330410H16Rik* | | | 1.82 |
| *7530408C15Rik* | | | 1.74 |
| *8030404L10Rik* | | | 1.51 |
| *8030467N07Rik* | AK078769 | | 1.64 |
| *9130020G22Rik* | AK082376 | | 1.61 |
| *9330155G14Rik* | AK079073 | | 1.53 |
| *9330177P20Rik* | NM_177462 | | 1.65 |
| *9330185J12Rik* | | | 2.05 |
| *9630009A08Rik* | AK035833 | | 2.25 |
| *9630025I21Rik* | NM_001076791.2 | RIKEN cDNA 9630025I21 gene (9630025I21Rik) | 1.71 |
| *9830134K01Rik* | AK036567 | | 1.58 |
| *9830169C18Rik* | NM_172581.1 | RIKEN cDNA 9830169C18 gene (9830169C18Rik) | 1.65 |
| *A130022A09Rik* | AK037499 | | 1.52 |
| *A230057G18Rik* | XM_489103 | | 3.67 |
| *A230057M07Rik* | AK038731 | | 1.83 |
| *a2ld1* | NM_145466.1 | AIG2-like domain 1 (a2ld1) | 1.50 |
| *A330021E22Rik* | NM_172447.2 | RIKEN cDNA A330021E22 gene (A330021E22Rik) | 2.48 |
| *A430010E21Rik* | AK039823 | | 1.51 |
| *A730036E13Rik* | XM_989657.2 | PREDICTED: RIKEN cDNA A730036E13 gene (A730036E13Rik) | 1.54 |
| *A830073O21Rik* | NM_177118 | | 2.44 |
| *A830080H07Rik* | | | 1.91 |
| *A830087P12Rik* | AK044077 | | 1.51 |
| *A830094I09Rik* | NM_177225 | | 1.91 |
| *A930008A22Rik* | NM_172768 | | 2.94 |
| *Abat* | NM_172961.2 | 4-aminobutyrate aminotransferase (Abat), nuclear gene encoding mitochondrial protein | 1.59 |
| *Abcc12* | NM_172912.4 | ATP-binding cassette, sub-family C (CFTR/MRP), member 12 (Abcc12) | 2.79 |
| *Abcc5* | NM_176839.1 | ATP-binding cassette, sub-family C (CFTR/MRP), member 5 (Abcc5), transcript variant 2 | 1.85 |
| *Abhd1* | NR_003522.1 | abhydrolase domain containing 1 (Abhd1), transcribed RNA. | 1.80 |
| *Abhd14b* | NM_029631.2 | abhydrolase domain containing 14b (Abhd14b) | 5.69 |
| *Abhd4* | NM_134076.1 | abhydrolase domain containing 4 (Abhd4) | 1.55 |

| | | | |
|---|---|---|---|
| *Abhd7* | NM_001001804.2 | abhydrolase domain containing 7 (Abhd7) | 1.50 |
| *Ablim2* | NM_177678.6 | actin-binding LIM protein 2 (Ablim2) | 2.29 |
| *Acadsb* | NM_025826.3 | acyl-Coenzyme A dehydrogenase, short/branched chain (Acadsb), nuclear gene encoding mitochondrial protein | 1.53 |
| *Acot1* | NM_012006.2 | acyl-CoA thioesterase 1 (Acot1) | 1.87 |
| *Acot11* | NM_025590.3 | acyl-CoA thioesterase 11 (Acot11) | 2.15 |
| *Acsl6* | NM_001033597.1 | acyl-CoA synthetase long-chain family member 6 (Acsl6), transcript variant 2 | 2.28 |
| *Acss1* | NM_080575.1 | acyl-CoA synthetase short-chain family member 1 (Acss1), nuclear gene encoding mitochondrial protein | 2.04 |
| *Acta2* | NM_007392.2 | actin, alpha 2, smooth muscle, aorta (Acta2) | 2.38 |
| *Actn1* | NM_134156.1 | actinin, alpha 1 (Actn1) | 2.59 |
| *Adamts16* | NM_172053.2 | a disintegrin-like and metallopeptidase (reprolysin type) with thrombospondin type 1 motif, 16 (Adamts16) | 2.14 |
| *Adamtsl4* | NM_144899.2 | ADAMTS-like 4 (Adamtsl4) | 2.33 |
| *Adck4* | NM_133770.1 | | 1.85 |
| *Adcy2* | NM_153534.2 | adenylate cyclase 2 (Adcy2) | 2.43 |
| *Adcy8* | NM_009623.2 | adenylate cyclase 8 (Adcy8) | 2.36 |
| *Adora1* | NM_001008533.2 | adenosine A1 receptor (Adora1), transcript variant 1 | 1.63 |
| *Adra2a* | NM_007417.2 | adrenergic receptor, alpha 2a (Adra2a) | 8.83 |
| *Agpat3* | NM_053014.3 | 1-acylglycerol-3-phosphate O-acyltransferase 3 (Agpat3) | 1.52 |
| *Agpat4* | NM_026644.1 | 1-acylglycerol-3-phosphate O-acyltransferase 4 (lysophosphatidic acid acyltransferase, delta) (Agpat4) | 1.79 |
| *Ahnak* | NM_009643.1 | AHNAK nucleoprotein (desmoyokin) (Ahnak), transcript variant 1 | 22.31 |
| *AI316807* | NM_001012667.1 | expressed sequence AI316807 (AI316807) | 1.53 |
| *AI646023* | NM_198860.1 | expressed sequence AI646023 (AI646023) | 1.58 |
| *AI662250* | NM_178926.2 | expressed sequence AI662250 (AI662250) | 1.95 |
| *AI875142* | | | 2.74 |
| *Aifm2* | NM_153779.1 | apoptosis-inducing factor, mitochondrion-associated 2 (Aifm2), nuclear gene encoding mitochondrial protein, transcript variant 1 | 1.53 |
| *Ak3* | NM_021299.1 | adenylate kinase 3 (Ak3), nuclear gene encoding mitochondrial protein | 1.51 |
| *Ak3l1* | NM_009647.4 | adenylate kinase 3-like 1 (Ak3l1), nuclear gene encoding mitochondrial protein | 1.73 |
| *Akap14* | NM_001033785.1 | A kinase (PRKA) anchor protein 14 (Akap14) | 2.75 |
| *Akap8l* | NM_017476.2 | A kinase (PRKA) anchor protein 8-like (Akap8l) | 2.03 |
| *Aktip* | NM_010241.1 | thymoma viral proto-oncogene 1 interacting protein (Aktip) | 1.71 |
| *Aldh1a1* | NM_013467.3 | aldehyde dehydrogenase family 1, subfamily A1 (Aldh1a1) | 2.29 |
| *Aldh3a2* | NM_007437 | | 1.72 |
| *Aldh4a1* | NM_175438.3 | aldehyde dehydrogenase 4 family, member A1 (Aldh4a1), nuclear gene encoding mitochondrial protein | 1.90 |
| *Aldh6a1* | NM_134042.2 | aldehyde dehydrogenase family 6, subfamily A1 (Aldh6a1), nuclear gene encoding mitochondrial protein | 1.76 |
| *Aldoc* | NM_009657.3 | aldolase C, fructose-bisphosphate (Aldoc) | 1.55 |
| *Amy1* | NM_007446.1 | amylase 1, salivary (Amy1) | 1.67 |
| *Amy2-2* | NM_001042711.2 | amylase 2-2, pancreatic (Amy2-2) | 4.70 |
| *Anapc2* | NM_175300.2 | anaphase promoting complex subunit 2 (Anapc2) | 1.95 |
| *Angpt1* | NM_009640.3 | angiopoietin 1 (Angpt1) | 1.76 |
| *Angptl4* | NM_020581.1 | angiopoietin-like 4 (Angptl4) | 2.20 |
| *Ank* | NM_020332.3 | progressive ankylosis (Ank) | 3.90 |
| *Anks3* | NM_028301.2 | ankyrin repeat and sterile alpha motif domain containing 3 (Anks3) | 1.56 |
| *Anxa11* | NM_013469.1 | annexin A11 (Anxa11) | 1.75 |
| *Anxa4* | NM_013471 | | 1.57 |
| *Anxa5* | NM_009673.1 | annexin A5 (Anxa5) | 2.74 |
| *Ap3m2* | NM_029505.1 | adaptor-related protein complex 3, mu 2 subunit (Ap3m2) | 3.35 |
| *Apbb1* | NM_009685.2 | amyloid beta (A4) precursor protein-binding, family B, member 1 (Apbb1) | 1.64 |
| *Aph1b* | NM_177583.4 | anterior pharynx defective 1b homolog (C. elegans) (Aph1b) | 1.78 |
| *Apoe* | NM_009696.2 | apolipoprotein E (Apoe) | 7.47 |
| *Appl2* | NM_145220.2 | adaptor protein, phosphotyrosine interaction, PH domain and leucine zipper containing 2 (Appl2) | 1.54 |
| *Aqp4* | NM_009700.1 | aquaporin 4 (Aqp4) | 4.26 |
| *Aqp9* | NM_022026.2 | aquaporin 9 (Aqp9) | 1.62 |
| *Arf2* | NM_007477.4 | ADP-ribosylation factor 2 (Arf2) | 1.63 |
| *Arhgap12* | NM_029277.2 | Rho GTPase activating protein 12 (Arhgap12), transcript variant 2 | 2.32 |
| *Arhgef18* | NM_133962.3 | rho/rac guanine nucleotide exchange factor (GEF) 18 (Arhgef18) | 2.08 |
| *Arhgef4* | NM_183019.2 | Rho guanine nucleotide exchange factor (GEF) 4 (Arhgef4) | 3.57 |
| *Armc3* | NM_001081083.1 | armadillo repeat containing 3 (Armc3) | 2.37 |
| *Armc9* | NM_030184.2 | armadillo repeat containing 9 (Armc9), transcript variant 1 | 1.69 |
| *Arrdc3* | NM_178917.2 | | 2.53 |
| *Arsb* | NM_009712.3 | arylsulfatase B (Arsb) | 4.94 |
| *Ascc1* | NM_026937.1 | activating signal cointegrator 1 complex subunit 1 (Ascc1) | 1.97 |
| *Asphd2* | NM_028386.1 | aspartate beta-hydroxylase domain containing 2 (Asphd2) | 1.77 |
| *Ass1* | NM_007494.3 | argininosuccinate synthetase 1 (Ass1) | 2.32 |
| *Atf3* | NM_007498.2 | activating transcription factor 3 (Atf3) | 1.92 |

| | | | |
|---|---|---|---|
| *Atp1a1* | NM_144900.1 | ATPase, Na+/K+ transporting, alpha 1 polypeptide (Atp1a1) | 2.31 |
| *Atp1b1* | NM_009721.4 | ATPase, Na+/K+ transporting, beta 1 polypeptide (Atp1b1) | 2.17 |
| *AU021034* | NM_177629.2 | expressed sequence AU021034 (AU021034) | 4.04 |
| *AU040829* | NM_175003.3 | expressed sequence AU040829 (AU040829), transcript variant 2 | 1.53 |
| *AW555464* | XM_127132.3 | | 2.82 |
| *Axl* | NM_009465.3 | AXL receptor tyrosine kinase (Axl) | 2.27 |
| *B230112P13Rik* | | | 2.27 |
| *B230208N19Rik* | XM_981366.1 | PREDICTED: RIKEN cDNA B230208N19 gene, transcript variant 1 (B230208N19Rik) | 1.65 |
| *B230311B06Rik* | XM_001001884.2 | PREDICTED: RIKEN cDNA B230311B06 gene (B230311B06Rik) | 1.79 |
| *B230312I18Rik* | XM_001002154.1 | PREDICTED: RIKEN cDNA B230312I18 gene, transcript variant 3 (B230312I18Rik) | 1.57 |
| *B230326M20Rik* | AK045953 | | 2.32 |
| *B230342M21Rik* | NM_133898.3 | RIKEN cDNA B230342M21 gene (B230342M21Rik) | 1.58 |
| *B230342N21Rik* | AK046118 | | 1.88 |
| *B230373P09Rik* | NM_177336.2 | | 1.50 |
| *B3galt5* | NM_033149.2 | UDP-Gal:betaGlcNAc beta 1,3-galactosyltransferase, polypeptide 5 (B3galt5) | 1.52 |
| *B930008G03Rik* | AK046967 | | 4.55 |
| *B930041G04* | NM_177750.2 | hypothetical protein B930041G04 (B930041G04) | 1.62 |
| *B930045J24Rik* | AK047284 | | 1.87 |
| *B9d1* | NM_013717.2 | B9 protein domain 1 (B9d1) | 2.25 |
| *Bace2* | NM_019517.2 | beta-site APP-cleaving enzyme 2 (Bace2) | 2.91 |
| *BB128963* | NM_172742 | | 1.57 |
| *Bbc3* | NM_133234.1 | BCL2 binding component 3 (Bbc3) | 1.94 |
| *Bbox1* | NM_130452.1 | butyrobetaine (gamma), 2-oxoglutarate dioxygenase 1 (gamma-butyrobetaine hydroxylase) (Bbox1) | 3.68 |
| *Bbs2* | NM_026116.2 | Bardet-Biedl syndrome 2 (human) (Bbs2) | 1.96 |
| *Bbs4* | NM_175325.2 | Bardet-Biedl syndrome 4 (human) (Bbs4) | 2.30 |
| *Bbs9* | NM_181316.3 | Bardet-Biedl syndrome 9 (Bbs9) | 1.79 |
| *BC022687* | NM_145450.3 | cDNA sequence BC022687 (BC022687) | 1.63 |
| *BC024537* | NM_146237.1 | | 1.68 |
| *BC030476* | NM_173421.1 | cDNA sequence BC030476 (BC030476) | 3.19 |
| *BC038167* | XM_196478 | | 3.23 |
| *BC050811* | NM_178418.2 | cDNA sequence BC050811 (BC050811) | 2.23 |
| *BC051142* | NM_001001177.1 | cDNA sequence BC051142 (BC051142) | 1.58 |
| *BC099439* | NM_001025564.1 | cDNA sequence BC099439 (BC099439) | 3.32 |
| *Bcar3* | NM_013867.1 | breast cancer anti-estrogen resistance 3 (Bcar3) | 1.72 |
| *Bcas3* | NM_138681.3 | breast carcinoma amplified sequence 3 (Bcas3) | 2.39 |
| *Bdh2* | NM_027208.1 | 3-hydroxybutyrate dehydrogenase, type 2 (Bdh2) | 5.05 |
| *Bicc1* | NM_031397.2 | bicaudal C homolog 1 (Drosophila) (Bicc1) | 2.27 |
| *Birc1cl* | | | 2.79 |
| *Bloc1s1* | NM_015740.2 | biogenesis of lysosome-related organelles complex-1, subunit 1 (Bloc1s1) | 1.54 |
| *Bmpr1b* | NM_007560.3 | bone morphogenetic protein receptor, type 1B (Bmpr1b) | 1.50 |
| *Bzrap1* | NM_172449.1 | benzodiazapine receptor associated protein 1 (Bzrap1) | 3.28 |
| *C030009J22Rik* | | | 7.44 |
| *C030014I23Rik* | | | 1.61 |
| *C030027H14Rik* | | | 1.95 |
| *C030048H21Rik* | XM_975397.2 | PREDICTED: RIKEN cDNA C030048H21 gene (C030048H21Rik) | 1.80 |
| *C130023A14Rik* | | | 1.78 |
| *C130072A16Rik* | AK081724 | | 1.55 |
| *C130092E12* | | | 1.72 |
| *C1ql2* | NM_207233.1 | complement component 1, q subcomponent-like 2 (C1ql2) | 1.72 |
| *C230037E05Rik* | | | 1.52 |
| *C230070D10Rik* | AK082620 | | 2.72 |
| *C3* | NM_009778.1 | | 51.54 |
| *C330001K17Rik* | XM_134745.2 | | 1.60 |
| *C430004E15Rik* | NM_175286.3 | RIKEN cDNA C430004E15 gene (C430004E15Rik) | 1.60 |
| *C4a* | NM_011413.2 | complement component 4A (Rodgers blood group) (C4a) | 7.50 |
| *C4b* | NM_009780.1 | complement component 4B (Childo blood group) (C4b) | 6.26 |
| *C530044C16Rik* | | | 7.10 |
| *C530044N13Rik* | NM_146067.3 | RIKEN cDNA C530044N13 gene (C530044N13Rik) | 1.51 |
| *C630002C17Rik* | AK049826 | | 3.41 |
| *C730013O11Rik* | AK050083 | | 1.70 |
| *Cachd1* | NM_198037.1 | cache domain containing 1 (Cachd1) | 2.54 |
| *Cacna2d3* | NM_009785.1 | calcium channel, voltage-dependent, alpha2/delta subunit 3 (Cacna2d3) | 1.53 |
| *Cacnb4* | NM_146123.2 | calcium channel, voltage-dependent, beta 4 subunit (Cacnb4), transcript variant 2 | 1.64 |
| *Camk2g* | NM_178597.4 | calcium/calmodulin-dependent protein kinase II gamma (Camk2g) | 1.62 |
| *Capn1* | NM_007600.2 | calpain 1 (Capn1) | 1.53 |
| *Capn6* | NM_007603.2 | calpain 6 (Capn6) | 1.55 |
| *Capns1* | NM_009795 | | 1.62 |

| | | | |
|---|---|---|---|
| *Capsl* | NM_029341.1 | calcyphosine-like (Capsl) | 1.72 |
| *Carhsp1* | NM_025821.2 | calcium regulated heat stable protein 1 (Carhsp1) | 1.79 |
| *Casq2* | NM_009814.1 | calsequestrin 2 (Casq2) | 2.03 |
| *Cbx7* | NM_144811.3 | chromobox homolog 7 (Cbx7) | 1.59 |
| *Cc2d2a* | NM_172274.1 | coiled-coil and C2 domain containing 2A (Cc2d2a) | 1.52 |
| *Ccdc103* | NM_028492.2 | coiled-coil domain containing 103 (Ccdc103) | 1.55 |
| *Ccdc114* | NM_001033243.2 | coiled-coil domain containing 114 (Ccdc114) | 1.70 |
| *Ccdc19* | XM_355284.5 | PREDICTED: coiled-coil domain containing 19, transcript variant 1 (Ccdc19) | 2.52 |
| *Ccdc39* | NM_026222.2 | coiled-coil domain containing 39 (Ccdc39) | 2.24 |
| *Ccdc65* | NM_153518.1 | coiled-coil domain containing 65 (Ccdc65) | 1.65 |
| *Ccdc80* | NM_026439.2 | coiled-coil domain containing 80 (Ccdc80) | 1.54 |
| *Ccdc96* | NM_025725.2 | coiled-coil domain containing 96 (Ccdc96) | 1.89 |
| *Ccl27* | NM_001048179.1 | chemokine (C-C motif) ligand 27 (Ccl27), transcript variant 2 | 1.62 |
| *Ccrk* | NM_053180.2 | cell cycle related kinase (Ccrk) | 2.18 |
| *Cd109* | NM_153098.2 | CD109 antigen (Cd109) | 2.37 |
| *Cd14* | NM_009841.3 | CD14 antigen (Cd14) | 1.96 |
| *Cd200* | NM_010818.3 | CD200 antigen (Cd200) | 1.53 |
| *Cd59a* | NM_007652.2 | CD59a antigen (Cd59a) | 6.72 |
| *Cd59b* | NM_181858.1 | CD59b antigen (Cd59b) | 1.68 |
| *Cdc42ep4* | NM_020006.1 | CDC42 effector protein (Rho GTPase binding) 4 (Cdc42ep4) | 2.29 |
| *Cdkn2b* | NM_007670.3 | cyclin-dependent kinase inhibitor 2B (p15, inhibits CDK4) (Cdkn2b) | 1.88 |
| *Cdo1* | NM_033037.3 | cysteine dioxygenase 1, cytosolic (Cdo1) | 2.10 |
| *Cds1* | NM_173370.3 | CDP-diacylglycerol synthase 1 (Cds1) | 1.86 |
| *Celsr1* | NM_009886.2 | cadherin, EGF LAG seven-pass G-type receptor 1 (flamingo homolog, Drosophila) (Celsr1) | 1.69 |
| *Cetn4* | NM_145825.1 | centrin 4 (Cetn4) | 2.74 |
| *Chchd10* | NM_175329.3 | coiled-coil-helix-coiled-coil-helix domain containing 10 (Chchd10) | 2.89 |
| *Chchd6* | NM_025351.2 | coiled-coil-helix-coiled-coil-helix domain containing 6 (Chchd6) | 1.51 |
| *Chd5* | NM_001081376.1 | chromodomain helicase DNA binding protein 5 (Chd5), transcript variant 1 | 1.64 |
| *Chi3l1* | NM_007695.2 | chitinase 3-like 1 (Chi3l1) | 8.13 |
| *Chka* | NM_001025566.1 | choline kinase alpha (Chka), transcript variant 2 | 1.93 |
| *Ckap5* | NM_029437.1 | cytoskeleton associated protein 5 (Ckap5) | 1.57 |
| *Ckmt1* | NM_009897.2 | creatine kinase, mitochondrial 1, ubiquitous (Ckmt1), nuclear gene encoding mitochondrial protein | 1.69 |
| *Cldn2* | NM_016675.3 | claudin 2 (Cldn2) | 1.51 |
| *Clip4* | NM_030179.2 | CAP-GLY domain containing linker protein family, member 4 (Clip4) | 1.82 |
| *Cln3* | NM_009907.2 | ceroid lipofuscinosis, neuronal 3, juvenile (Batten, Spielmeyer-Vogt disease) (Cln3) | 1.58 |
| *Cml1* | NM_023160.1 | camello-like 1 (Cml1) | 3.11 |
| *Cml4* | NM_023455.2 | camello-like 4 (Cml4) | 2.21 |
| *Cmtm5* | NM_026066.2 | CKLF-like MARVEL transmembrane domain containing 5 (Cmtm5) | 6.81 |
| *Cnp* | NM_009923.1 | 2',3'-cyclic nucleotide 3' phosphodiesterase (Cnp) | 1.67 |
| *Col22a1* | XM_907370.3 | PREDICTED: collagen, type XXII, alpha 1, transcript variant 3 (Col22a1) | 1.93 |
| *Col8a2* | NM_199473 | | 1.95 |
| *Cox6b2* | NM_183405.1 | cytochrome c oxidase subunit VIb polypeptide 2 (Cox6b2) | 1.58 |
| *Cp* | NM_007752 | | 2.68 |
| *Cpe* | NM_013494.2 | | 1.74 |
| *Cpt1c* | NM_153679.1 | carnitine palmitoyltransferase 1c (Cpt1c) | 1.60 |
| *Crip2* | NM_024223.1 | cysteine rich protein 2 (Crip2) | 1.55 |
| *Crtc3* | NM_173863.2 | CREB regulated transcription coactivator 3 (Crtc3) | 1.51 |
| *Cryab* | NM_009964.1 | crystallin, alpha B (Cryab) | 3.40 |
| *Crygs* | NM_009967.1 | crystallin, gamma S (Crygs) | 2.00 |
| *Csf1* | NM_007778.3 | colony stimulating factor 1 (macrophage) (Csf1) | 2.24 |
| *Cspp1* | NM_026493.3 | centrosome and spindle pole associated protein 1 (Cspp1) | 1.61 |
| *Csrp1* | NM_007791.4 | cysteine and glycine-rich protein 1 (Csrp1) | 1.57 |
| *Ctdsp2* | NM_146012.1 | CTD (carboxy-terminal domain, RNA polymerase II, polypeptide A) small phosphatase 2 (Ctdsp2) | 1.65 |
| *Ctnna2* | NM_009819.1 | catenin (cadherin associated protein), alpha 2 (Ctnna2), transcript variant 2 | 2.32 |
| *Ctnnal1* | NM_018761.2 | catenin (cadherin associated protein), alpha-like 1 (Ctnnal1) | 3.35 |
| *Ctns* | NM_031251.4 | cystinosis, nephropathic (Ctns) | 1.59 |
| *Ctsa* | NM_001038492.1 | cathepsin A (Ctsa), transcript variant 2 | 8.16 |
| *Ctsb* | NM_007798.2 | cathepsin B (Ctsb) | 2.19 |
| *Cxcl10* | NM_021274.1 | chemokine (C-X-C motif) ligand 10 (Cxcl10) | 1.88 |
| *Cxcr4* | NM_009911.2 | | 3.50 |
| *Cyhr1* | NM_180962.1 | cysteine and histidine rich 1 (Cyhr1), transcript variant 2 | 2.29 |
| *Cyld* | NM_173369.1 | cylindromatosis (turban tumor syndrome) (Cyld) | 1.72 |
| *Cyp1b1* | NM_009994.1 | cytochrome P450, family 1, subfamily b, polypeptide 1 (Cyp1b1) | 3.17 |
| *Cyp2j9* | NM_028979.2 | cytochrome P450, family 2, subfamily j, polypeptide 9 (Cyp2j9) | 1.62 |
| *Cyp4f13* | NM_130882.1 | cytochrome P450, family 4, subfamily f, polypeptide 13 (Cyp4f13) | 2.07 |
| *Cyp4f14* | NM_022434.1 | cytochrome P450, family 4, subfamily f, polypeptide 14 (Cyp4f14) | 3.05 |
| *Cyp4v3* | NM_133969.2 | cytochrome P450, family 4, subfamily v, polypeptide 3 (Cyp4v3) | 2.31 |

| | | | |
|---|---|---|---|
| *Cyp7b1* | NM_007825.3 | cytochrome P450, family 7, subfamily b, polypeptide 1 (Cyp7b1) | 1.76 |
| *Cyr61* | NM_010516.1 | cysteine rich protein 61 (Cyr61) | 1.59 |
| *D030034I04Rik* | AK050914 | | 1.51 |
| *D0H4S114* | NM_053078.3 | DNA segment, human D4S114 (D0H4S114) | 2.60 |
| *D10Ertd610e* | NM_028027.2 | DNA segment, Chr 10, ERATO Doi 610, expressed (D10Ertd610e) | 2.06 |
| *D12Ertd647e* | NM_194068.1 | DNA segment, Chr 12, ERATO Doi 647, expressed (D12Ertd647e), transcript variant 4 | 1.59 |
| *D15Mit260* | | | 1.56 |
| *D16H22S680E* | NM_138583.1 | DNA segment, Chr 16, human D22S680E, expressed (D16H22S680E) | 1.67 |
| *D230005E09Rik* | AK051822 | | 1.83 |
| *D330014H01Rik* | NM_177617.2 | | 2.05 |
| *D430042O09Rik* | NM_001081022.1 | RIKEN cDNA D430042O09 gene (D430042O09Rik) | 2.43 |
| *D4Bwg1540e* | NM_026257.1 | | 1.64 |
| *D4Ertd22e* | NM_001025608.1 | DNA segment, Chr 4, ERATO Doi 22, expressed (D4Ertd22e), transcript variant 1 | 1.51 |
| *D4Ertd681e* | | | 2.03 |
| *D630003M21Rik* | NM_177657.2 | RIKEN cDNA D630003M21 gene (D630003M21Rik) | 1.57 |
| *D730039F16Rik* | NM_030021.1 | RIKEN cDNA D730039F16 gene (D730039F16Rik) | 1.61 |
| *D9Ertd392e* | NM_028181 | | 2.14 |
| *Dapk1* | NM_134062.1 | death associated protein kinase 1 (Dapk1), transcript variant 2 | 1.58 |
| *Darc* | NM_010045.2 | Duffy blood group, chemokine receptor (Darc) | 1.94 |
| *Dbi* | NM_007830.3 | diazepam binding inhibitor (Dbi), transcript variant 2 | 1.76 |
| *Dbp* | NM_016974.1 | D site albumin promoter binding protein (Dbp) | 1.61 |
| *Dcun1d4* | NM_178896.4 | DCN1, defective in cullin neddylation 1, domain containing 4 (S. cerevisiae) (Dcun1d4) | 1.54 |
| *Ddit4l* | NM_030143.3 | DNA-damage-inducible transcript 4-like (Ddit4l) | 1.55 |
| *Ddo* | NM_027442.3 | D-aspartate oxidase (Ddo) | 6.50 |
| *Ddr1* | NM_007584.2 | discoidin domain receptor family, member 1 (Ddr1), transcript variant 1 | 2.58 |
| *Ddx3y* | NM_012008.1 | DEAD (Asp-Glu-Ala-Asp) box polypeptide 3, Y-linked (Ddx3y) | 1.62 |
| *Dedd2* | NM_207677.3 | death effector domain-containing DNA binding protein 2 (Dedd2) | 1.55 |
| *Dhrs1* | NM_026819.2 | dehydrogenase/reductase (SDR family) member 1 (Dhrs1) | 4.26 |
| *Dhx32* | NM_133941.2 | DEAH (Asp-Glu-Ala-His) box polypeptide 32 (Dhx32) | 1.93 |
| *Dido1* | NM_011805.2 | death inducer-obliterator 1 (Dido1), transcript variant 1 | 1.52 |
| *Dixdc1* | NM_178118 | | 1.66 |
| *Dm15* | NM_032418 | | 1.87 |
| *Dnahc9* | XM_917040.2 | PREDICTED: dynein, axonemal, heavy chain 9, transcript variant 5 (Dnahc9) | 1.64 |
| *Dnaic1* | NM_175138.3 | dynein, axonemal, intermediate chain 1 (Dnaic1) | 1.61 |
| *Dnajb13* | NM_153527.2 | DnaJ (Hsp40) related, subfamily B, member 13 (Dnajb13) | 4.31 |
| *Dnajb2* | NM_178055.3 | DnaJ (Hsp40) homolog, subfamily B, member 2 (Dnajb2), transcript variant 2 | 2.03 |
| *Dnajb4* | NM_025926.1 | DnaJ (Hsp40) homolog, subfamily B, member 4 (Dnajb4) | 1.52 |
| *Dnajb9* | NM_013760.3 | DnaJ (Hsp40) homolog, subfamily B, member 9 (Dnajb9) | 1.66 |
| *Dnajc3* | NM_008929.2 | DnaJ (Hsp40) homolog, subfamily C, member 3 (Dnajc3) | 1.93 |
| *Dnali1* | NM_175223.2 | dynein, axonemal, light intermediate polypeptide 1 (Dnali1) | 3.48 |
| *Dner* | NM_152915.1 | delta/notch-like EGF-related receptor (Dner) | 1.79 |
| *Dock6* | NM_177030.3 | dedicator of cytokinesis 6 (Dock6) | 2.57 |
| *Dpysl5* | NM_023047.2 | dihydropyrimidinase-like 5 (Dpysl5) | 2.89 |
| *Drp2* | NM_010078.2 | dystrophin related protein 2 (Drp2) | 5.02 |
| *Dscr1* | NM_019466.2 | | 1.85 |
| *Dtna* | NM_010087.3 | dystrobrevin alpha (Dtna), transcript variant 2 | 1.53 |
| *Dtx4* | NM_172442.2 | deltex 4 homolog (Drosophila) (Dtx4) | 1.76 |
| *Dync2li1* | NM_172256.1 | dynein cytoplasmic 2 light intermediate chain 1 (Dync2li1) | 2.33 |
| *Dynlrb2* | NM_029297.1 | dynein light chain roadblock-type 2 (Dynlrb2) | 6.62 |
| *Dysf* | NM_021469.2 | dysferlin (Dysf), transcript variant 1 | 1.96 |
| *E130203B14Rik* | NM_178791.4 | RIKEN cDNA E130203B14 gene (E130203B14Rik) | 2.90 |
| *E330035H20Rik* | AK054516 | | 1.65 |
| *E430002G05Rik* | NM_173749 | | 4.40 |
| *E430013K19Rik* | | | 1.61 |
| *Ech1* | NM_016772.1 | enoyl coenzyme A hydratase 1, peroxisomal (Ech1) | 1.66 |
| *Ednra* | NM_010332.2 | endothelin receptor type A (Ednra) | 2.00 |
| *Ednrb* | NM_007904.2 | endothelin receptor type B (Ednrb) | 1.60 |
| *EG232599* | NM_177689.3 | predicted gene, EG232599 (EG232599) | 1.93 |
| *EG433229* | XM_899874.3 | PREDICTED: predicted gene, EG433229, transcript variant 7 (EG433229) | 1.66 |
| *EG546143* | XM_620762.4 | PREDICTED: predicted gene, EG546143 (EG546143) | 2.43 |
| *EG630499* | NM_001081015.1 | predicted gene, EG630499 (EG630499) | 1.62 |
| *EG632802* | XM_978542.1 | PREDICTED: predicted gene, EG632802 (EG632802) | 2.20 |
| *EG665378* | NM_001081746.1 | predicted gene, EG665378 (EG665378) | 3.03 |
| *Ei24* | NM_007915 | | 1.53 |
| *Endod1* | NM_028013.2 | endonuclease domain containing 1 (Endod1) | 2.59 |
| *Enpp5* | NM_032003.1 | ectonucleotide pyrophosphatase/phosphodiesterase 5 (Enpp5) | 1.71 |
| *Entpd2* | NM_009849.1 | ectonucleoside triphosphate diphosphohydrolase 2 (Entpd2) | 5.54 |
| *Etl4* | NM_001081006.1 | enhancer trap locus 4 (Etl4) | 1.62 |

| | | | |
|---|---|---|---|
| *F830002E14Rik* | AK089567 | | 6.28 |
| *Fads3* | NM_021890.3 | fatty acid desaturase 3 (Fads3) | 1.88 |
| *Fah* | NM_010176.2 | fumarylacetoacetate hydrolase (Fah) | 1.84 |
| *Fam102a* | NM_153560.4 | family with sequence similarity 102, member A (Fam102a) | 2.69 |
| *Fam129b* | NM_146119.1 | family with sequence similarity 129, member B (Fam129b) | 1.79 |
| *Fam134b* | NM_025459.2 | family with sequence similarity 134, member B (Fam134b), transcript variant 2 | 3.72 |
| *Fam178a* | NM_001081225.1 | family with sequence similarity 178, member A (Fam178a) | 1.64 |
| *Fam184a* | NM_001081428.1 | family with sequence similarity 184, member A (Fam184a) | 3.08 |
| *Fam20a* | NM_153782.1 | family with sequence similarity 20, member A (Fam20a) | 1.69 |
| *Fank1* | NM_025850.2 | fibronectin type 3 and ankyrin repeat domains 1 (Fank1) | 1.80 |
| *Fas* | NM_007987.1 | Fas (TNF receptor superfamily member 6) (Fas) | 4.91 |
| *Fbxo2* | NM_176848.1 | F-box protein 2 (Fbxo2) | 8.52 |
| *Fbxo32* | NM_026346.1 | F-box protein 32 (Fbxo32) | 1.53 |
| *Fbxo44* | NM_173401.2 | F-box protein 44 (Fbxo44) | 1.74 |
| *Fcgrt* | NM_010189 | | 1.97 |
| *Fer1l3* | XM_001480162.1 | PREDICTED: fer-1-like 3, myoferlin (C. elegans), transcript variant 1 (Fer1l3) | 2.16 |
| *Fez1* | NM_183171.1 | fasciculation and elongation protein zeta 1 (zygin I) (Fez1) | 1.76 |
| *Fez2* | NM_199448.1 | | 1.81 |
| *Fhl1* | NM_001077361.1 | four and a half LIM domains 1 (Fhl1), transcript variant 1 | 2.54 |
| *Fhod1* | NM_177699 | | 1.52 |
| *Fin15* | NM_008016.1 | | 1.71 |
| *Flnb* | NM_134080.1 | filamin, beta (Flnb) | 1.84 |
| *Fmn2* | AK013585 | | 1.57 |
| *Fn3k* | NM_022014.2 | fructosamine 3 kinase (Fn3k), transcript variant 1 | 2.12 |
| *Fnbp1* | NM_001038700.1 | formin binding protein 1 (Fnbp1), transcript variant 1 | 1.79 |
| *Folr1* | NM_008034.2 | folate receptor 1 (adult) (Folr1) | 1.64 |
| *Fos* | NM_010234.2 | FBJ osteosarcoma oncogene (Fos) | 2.68 |
| *Frmd6* | NM_028127.3 | FERM domain containing 6 (Frmd6) | 1.55 |
| *Fundc1* | NM_028058.3 | FUN14 domain containing 1 (Fundc1) | 1.93 |
| *Fyco1* | NM_148925.1 | FYVE and coiled-coil domain containing 1 (Fyco1) | 1.81 |
| *Gab1* | NM_021356.2 | growth factor receptor bound protein 2-associated protein 1 (Gab1) | 3.05 |
| *Gabarapl1* | NM_020590.4 | gamma-aminobutyric acid (GABA(A)) receptor-associated protein-like 1 (Gabarapl1) | 2.03 |
| *Gabrb1* | NM_008069.4 | gamma-aminobutyric acid (GABA-A) receptor, subunit beta 1 (Gabrb1) | 1.71 |
| *Galntl1* | NM_001081421.1 | UDP-N-acetyl-alpha-D-galactosamine:polypeptide N-acetylgalactosaminyltransferase-like 1 (Galntl1) | 3.77 |
| *Ganc* | NM_172672.2 | glucosidase, alpha; neutral C (Ganc) | 1.56 |
| *Garnl3* | NM_178888.4 | GTPase activating RANGAP domain-like 3 (Garnl3) | 3.83 |
| *Gats* | XM_289726.2 | | 1.62 |
| *Gbp2* | NM_010260.1 | guanylate binding protein 2 (Gbp2) | 6.50 |
| *Gca* | NM_145523.3 | grancalcin (Gca) | 1.79 |
| *Gcnt2* | NM_133219.1 | glucosaminyl (N-acetyl) transferase 2, I-branching enzyme (Gcnt2), transcript variant 3 | 1.76 |
| *Gdap1l1* | NM_144891.1 | ganglioside-induced differentiation-associated protein 1-like 1 (Gdap1l1) | 1.54 |
| *Gdpd2* | NM_023608.2 | glycerophosphodiester phosphodiesterase domain containing 2 (Gdpd2) | 2.26 |
| *Gdpd5* | NM_201352.2 | glycerophosphodiester phosphodiesterase domain containing 5 (Gdpd5) | 1.50 |
| *Gfap* | NM_010277 | | 7.80 |
| *Ggnbp1* | NM_027544.1 | gametogenetin binding protein 1 (Ggnbp1) | 2.04 |
| *Ggta1* | NM_010283.1 | glycoprotein galactosyltransferase alpha 1, 3 (Ggta1) | 1.78 |
| *Gja1* | NM_010288.2 | gap junction membrane channel protein alpha 1 (Gja1) | 2.36 |
| *Glb1l* | | | 2.05 |
| *Glipr2* | NM_027450 | | 2.59 |
| *Glycam1* | NM_008134.2 | glycosylation dependent cell adhesion molecule 1 (Glycam1) | 2.21 |
| *Gm347* | NM_001005420.1 | gene model 347, (NCBI) (Gm347) | 1.56 |
| *Gm973* | NM_001013771.2 | gene model 973, (NCBI) (Gm973) | 2.89 |
| *Gmpr* | NM_025508.3 | guanosine monophosphate reductase (Gmpr) | 1.78 |
| *Gnao1* | NM_010308.3 | guanine nucleotide binding protein, alpha O (Gnao1), transcript variant A | 1.55 |
| *Gnptg* | NM_172529.3 | N-acetylglucosamine-1-phosphotransferase, gamma subunit (Gnptg) | 1.54 |
| *Golgb1* | XM_001001861.2 | PREDICTED: golgi autoantigen, golgin subfamily b, macrogolgin 1, transcript variant 9 (Golgb1) | 1.57 |
| *Gp38* | NM_010329.1 | | 1.84 |
| *Gpam* | NM_008149.3 | glycerol-3-phosphate acyltransferase, mitochondrial (Gpam), nuclear gene encoding mitochondrial protein | 1.63 |
| *Gpc4* | NM_008150.1 | glypican 4 (Gpc4) | 1.57 |
| *Gpld1* | NM_008156.2 | glycosylphosphatidylinositol specific phospholipase D1 (Gpld1) | 1.87 |
| *Gpr123* | NM_177469.3 | G protein-coupled receptor 123 (Gpr123) | 2.05 |
| *Gpr137b-ps* | NR_003568.1 | G protein-coupled receptor 137B, pseudogene (Gpr137b-ps), non-coding RNA. | 1.56 |
| *Gpr17* | NM_001025381.1 | G protein-coupled receptor 17 (Gpr17) | 1.50 |
| *Gpr23* | NM_175271 | | 2.13 |
| *Gpr37l1* | NM_134438.1 | G protein-coupled receptor 37-like 1 (Gpr37l1) | 3.20 |
| *Gprc5b* | NM_022420.1 | G protein-coupled receptor, family C, group 5, member B (Gprc5b) | 2.09 |

| | | | |
|---|---|---|---|
| *Gpx1* | NM_008160.5 | glutathione peroxidase 1 (Gpx1) | 2.78 |
| *Gpx4* | NM_001037741.2 | glutathione peroxidase 4 (Gpx4), transcript variant 1 | 1.87 |
| *Grhpr* | NM_080289.1 | glyoxylate reductase/hydroxypyruvate reductase (Grhpr) | 2.10 |
| *Gria1* | NM_008165.2 | glutamate receptor, ionotropic, AMPA1 (alpha 1) (Gria1) | 3.88 |
| *Grk4* | NM_019497.2 | G protein-coupled receptor kinase 4 (Grk4), transcript variant 1 | 1.60 |
| *Gsta3* | NM_010356.3 | glutathione S-transferase, alpha 3 (Gsta3), transcript variant 2 | 1.62 |
| *Gsta4* | NM_010357.1 | glutathione S-transferase, alpha 4 (Gsta4) | 2.12 |
| *H13* | NM_010376.3 | histocompatibility 13 (H13) | 1.54 |
| *H2-D1* | NM_010380.3 | histocompatibility 2, D region locus 1 (H2-D1) | 1.98 |
| *H2-K1* | NM_001001892.2 | histocompatibility 2, K1, K region (H2-K1), transcript variant 1 | 1.66 |
| *Hbp1* | NM_153198 | | 1.62 |
| *Hc* | NM_010406.1 | hemolytic complement (Hc) | 1.97 |
| *Hdc* | NM_008230.4 | histidine decarboxylase (Hdc) | 4.30 |
| *Heg1* | NM_172934.4 | HEG homolog 1 (zebrafish) (Heg1), transcript variant 2 | 1.70 |
| *Herpud1* | NM_022331.1 | homocysteine-inducible, endoplasmic reticulum stress-inducible, ubiquitin-like domain member 1 (Herpud1) | 1.61 |
| *Hexdc* | NM_001001333.1 | hexosaminidase (glycosyl hydrolase family 20, catalytic domain) containing (Hexdc) | 1.64 |
| *Hexim1* | NM_138753.2 | hexamethylene bis-acetamide inducible 1 (Hexim1) | 1.58 |
| *Hey1* | NM_010423.2 | hairy/enhancer-of-split related with YRPW motif 1 (Hey1) | 2.46 |
| *Hist1h2be* | NM_178194.3 | histone cluster 1, H2be (Hist1h2be) | 2.28 |
| *Hist1h4a* | NM_178192.1 | histone cluster 1, H4a (Hist1h4a) | 1.74 |
| *Hist1h4j* | NM_178210.1 | histone cluster 1, H4j (Hist1h4j) | 1.66 |
| *Hist2h2aa1* | NM_013549.1 | histone cluster 2, H2aa1 (Hist2h2aa1) | 2.78 |
| *Hist2h2aa2* | NM_178212.1 | histone cluster 2, H2aa2 (Hist2h2aa2) | 2.56 |
| *Hopx* | NM_175606.2 | HOP homeobox (Hopx) | 3.50 |
| *Hrsp12* | NM_008287.2 | heat-responsive protein 12 (Hrsp12) | 3.31 |
| *Hsd3b2* | NM_153193.2 | hydroxy-delta-5-steroid dehydrogenase, 3 beta- and steroid delta-isomerase 2 (Hsd3b2) | 3.94 |
| *Hspa2* | NM_008301.4 | heat shock protein 2 (Hspa2), transcript variant 1 | 2.29 |
| *Hspb8* | NM_030704.1 | heat shock protein 8 (Hspb8) | 9.66 |
| *Htatip2* | NM_016865.2 | HIV-1 tat interactive protein 2, homolog (human) (Htatip2) | 1.57 |
| *Id4* | NM_031166.2 | inhibitor of DNA binding 4 (Id4) | 4.70 |
| *Idh2* | NM_173011.1 | isocitrate dehydrogenase 2 (NADP+), mitochondrial (Idh2), nuclear gene encoding mitochondrial protein | 1.57 |
| *Ifit2* | NM_008332.2 | interferon-induced protein with tetratricopeptide repeats 2 (Ifit2) | 1.97 |
| *Ifit3* | NM_010501.1 | interferon-induced protein with tetratricopeptide repeats 3 (Ifit3) | 8.11 |
| *Ifngr2* | NM_008338.2 | interferon gamma receptor 2 (Ifngr2) | 2.15 |
| *Ift122* | NM_031177.2 | intraflagellar transport 122 homolog (Chlamydomonas) (Ift122) | 1.69 |
| *Ift172* | NM_026298.4 | intraflagellar transport 172 homolog (Chlamydomonas) (Ift172) | 1.72 |
| *Ift80* | NM_026641.1 | intraflagellar transport 80 homolog (Chlamydomonas) (Ift80) | 1.65 |
| *Ift81* | NM_009879.2 | intraflagellar transport 81 homolog (Chlamydomonas) (Ift81) | 2.18 |
| *Igfbp5* | NM_010518.2 | insulin-like growth factor binding protein 5 (Igfbp5) | 18.71 |
| *Igfbp7* | NM_008048.2 | insulin-like growth factor binding protein 7 (Igfbp7) | 2.78 |
| *IGFBPL* | AK043464 | | 1.60 |
| *Igfbpl1* | NM_018741.2 | insulin-like growth factor binding protein-like 1 (Igfbpl1) | 5.75 |
| *Igsf11* | NM_170599.2 | immunoglobulin superfamily, member 11 (Igsf11) | 7.84 |
| *Igtp* | NM_018738.3 | interferon gamma induced GTPase (Igtp) | 1.92 |
| *Ihpk1* | NM_013785.2 | inositol hexaphosphate kinase 1 (Ihpk1) | 1.54 |
| *Iigp2* | NM_019440.2 | interferon inducible GTPase 2 (Iigp2) | 2.07 |
| *Il6st* | NM_010560.2 | interleukin 6 signal transducer (Il6st) | 3.06 |
| *Inpp5k* | NM_008916.2 | inositol polyphosphate 5-phosphatase K (Inpp5k) | 2.88 |
| *Insl6* | NM_013754.1 | insulin-like 6 (Insl6) | 1.93 |
| *Iqcb1* | NM_177128.3 | IQ calmodulin-binding motif containing 1 (Iqcb1) | 1.65 |
| *Iqcg* | NM_178378.2 | IQ motif containing G (Iqcg) | 1.75 |
| *Itfg3* | NM_207217.3 | integrin alpha FG-GAP repeat containing 3 (Itfg3) | 1.54 |
| *Itga3* | NM_013565.2 | integrin alpha 3 (Itga3) | 3.26 |
| *Itgb4* | NM_133663.2 | integrin beta 4 (Itgb4), transcript variant 2 | 1.69 |
| *Itgb5* | NM_010580.1 | integrin beta 5 (Itgb5) | 1.87 |
| *Itih3* | NM_008407 | | 3.82 |
| *Itm2b* | NM_008410.1 | integral membrane protein 2B (Itm2b) | 2.63 |
| *Itpk1* | NM_172584.3 | inositol 1,3,4-triphosphate 5/6 kinase (Itpk1) | 1.56 |
| *Itpkb* | XM_205854 | | 2.50 |
| *Jakmip1* | NM_178394.3 | janus kinase and microtubule interacting protein 1 (Jakmip1) | 2.28 |
| *Kank4* | NM_172872.2 | KN motif and ankyrin repeat domains 4 (Kank4) | 2.01 |
| *Kcna6* | NM_013568.3 | | 2.92 |
| *Kcnk2* | NM_010607.1 | potassium channel, subfamily K, member 2 (Kcnk2) | 1.81 |
| *Khk* | NM_008439.3 | ketohexokinase (Khk) | 1.60 |
| *Kif1c* | NM_153103.2 | kinesin family member 1C (Kif1c) | 1.97 |
| *Kif21a* | NM_016705.2 | kinesin family member 21A (Kif21a) | 2.52 |
| *Kif27* | NM_175214.3 | kinesin family member 27 (Kif27) | 1.59 |

| | | | |
|---|---|---|---|
| *Kif9* | NM_010628.2 | kinesin family member 9 (Kif9) | 1.51 |
| *Klhdc8b* | NM_030075.1 | kelch domain containing 8B (Klhdc8b) | 2.32 |
| *Klhdc9* | NM_001033039.2 | kelch domain containing 9 (Klhdc9) | 1.62 |
| *Klhl26* | NM_172052.1 | kelch-like 26 (Drosophila) (Klhl26), transcript variant 1 | 2.17 |
| *Kndc1* | NM_177261.4 | kinase non-catalytic C-lobe domain (KIND) containing 1 (Kndc1) | 7.75 |
| *Ky* | NM_024291.3 | kyphoscoliosis peptidase (Ky) | 2.09 |
| *Lamb2* | NM_008483.2 | laminin, beta 2 (Lamb2) | 2.27 |
| *Lamp2* | NM_001017959.1 | lysosomal-associated membrane protein 2 (Lamp2), transcript variant 1 | 1.53 |
| *Lbp* | NM_008489.2 | lipopolysaccharide binding protein (Lbp) | 3.16 |
| *Lcn2* | NM_008491.1 | lipocalin 2 (Lcn2) | 10.93 |
| *Lgals3* | NM_010705.2 | lectin, galactose binding, soluble 3 (Lgals3) | 10.89 |
| *Lgals4* | NM_010706.1 | lectin, galactose binding, soluble 4 (Lgals4) | 1.79 |
| *Lgi2* | NM_144945.2 | leucine-rich repeat LGI family, member 2 (Lgi2) | 1.89 |
| *Limk2* | NM_001034030.1 | LIM motif-containing protein kinase 2 (Limk2), transcript variant 3 | 1.94 |
| *Lix1* | NM_025681.2 | limb expression 1 homolog (chicken) (Lix1) | 1.87 |
| *LOC100041569* | XM_001476596.1 | PREDICTED: hypothetical protein LOC100041569 (LOC100041569) | 1.52 |
| *LOC100043798* | XM_001481083.1 | PREDICTED: hypothetical protein LOC100043798 (LOC100043798) | 1.51 |
| *LOC100043821* | XM_001481017.1 | PREDICTED: hypothetical protein LOC100043821 (LOC100043821) | 2.42 |
| *LOC100044124* | XM_001474702.1 | PREDICTED: similar to Nedd4 binding protein 2 (LOC100044124) | 1.77 |
| *LOC100044177* | XM_001471637.1 | PREDICTED: hypothetical protein LOC100044177 (LOC100044177) | 3.16 |
| *LOC100045005* | XR_031422.1 | PREDICTED: similar to Deltex3 (LOC100045005), misc RNA. | 2.07 |
| *LOC100045501* | XM_001474411.1 | PREDICTED: hypothetical protein LOC100045501 (LOC100045501) | 1.78 |
| *LOC100045680* | XM_001475152.1 | PREDICTED: similar to complement C4 (LOC100045680) | 1.90 |
| *LOC100045864* | XR_032055.1 | PREDICTED: similar to HLA-G protein (LOC100045864), misc RNA. | 1.65 |
| *LOC100045869* | XM_001475055.1 | PREDICTED: similar to Limb expression 1 homolog (chicken) (LOC100045869) | 4.71 |
| *LOC100046120* | XM_001475611.1 | PREDICTED: similar to clusterin (LOC100046120) | 11.41 |
| *LOC100046207* | XM_001475801.1 | PREDICTED: similar to Lymphocyte antigen 6H precursor (Ly-6H) (LOC100046207) | 2.83 |
| *LOC100046690* | XM_001476775.1 | PREDICTED: hypothetical protein LOC100046690 (LOC100046690) | 1.54 |
| *LOC100046741* | XM_001476728.1 | PREDICTED: similar to red-1 (LOC100046741) | 4.78 |
| *LOC100047126* | XM_001477492.1 | PREDICTED: similar to Sctr protein, transcript variant 1 (LOC100047126) | 2.18 |
| *LOC100047193* | XR_033770.1 | PREDICTED: similar to kinesin family member 9 (LOC100047193), misc RNA. | 1.93 |
| *LOC100047214* | XM_001477665.1 | PREDICTED: similar to PTEN induced putative kinase 1 (LOC100047214) | 3.64 |
| *LOC100047226* | XR_033502.1 | PREDICTED: hypothetical protein LOC100047226 (LOC100047226), misc RNA. | 1.59 |
| *LOC100047264* | XM_001477759.1 | PREDICTED: hypothetical protein LOC100047264 (LOC100047264) | 1.93 |
| *LOC100047674* | XM_001478657.1 | PREDICTED: similar to solute carrier family 35 (UDP-glucuronic acid/UDP-N-acetylgalactosamine dual transporter), member D1 (LOC100047674) | 1.94 |
| *LOC100047738* | XM_001478788.1 | PREDICTED: similar to DENN/MADD domain containing 1A (LOC100047738) | 2.47 |
| *LOC100047856* | XM_001479297.1 | PREDICTED: similar to calponin 3, acidic (LOC100047856) | 1.61 |
| *LOC100047936* | XR_033905.1 | PREDICTED: similar to sortilin-related receptor, LDLR class A repeats-containing (LOC100047936), misc RNA. | 2.54 |
| *LOC100048083* | XR_034387.1 | PREDICTED: similar to Tect2 (LOC100048083), misc RNA. | 1.99 |
| *LOC100048331* | XR_034509.1 | PREDICTED: similar to DnaJ (Hsp40) homolog, subfamily A, member 4 (LOC100048331), misc RNA. | 2.03 |
| *LOC100048534* | XM_001480785.1 | PREDICTED: similar to D19Ertd652e protein (LOC100048534) | 1.80 |
| *LOC234987* | XM_134668.2 | | 1.64 |
| *LOC331595* | XM_284853.2 | | 1.75 |
| *LOC380653* | XM_354560.1 | | 1.91 |
| *LOC381000* | XM_354911.1 | | 2.91 |
| *LOC381105* | XM_355021.1 | | 1.78 |
| *LOC381132* | XM_355049.1 | | 2.60 |
| *LOC381150* | XM_358516.1 | | 1.53 |
| *LOC381260* | XM_355202.1 | | 1.79 |
| *LOC381738* | XM_355721.1 | | 3.25 |
| *LOC381739* | XM_358649.1 | | 1.84 |
| *LOC382050* | XM_356123.1 | | 1.55 |
| *LOC382163* | XM_356263.1 | | 2.75 |
| *LOC382237* | XM_356351.1 | | 1.79 |
| *LOC382308* | XM_358725.1 | | 1.51 |
| *LOC383154* | XM_356900.1 | | 1.85 |
| *LOC385256* | XM_358153.1 | | 2.23 |
| *LOC385274* | XM_358163.1 | | 2.07 |
| *LOC385825* | XM_358960.1 | | 1.72 |
| *LOC385877* | XM_358981.1 | | 1.54 |
| *LOC386005* | XM_359031.1 | | 3.37 |
| *LOC386021* | XM_359035.1 | | 1.61 |
| *LOC386078* | XM_359058.1 | | 1.73 |
| *LOC386085* | XM_359063.1 | | 1.66 |

| | | | |
|---|---|---|---|
| *LOC386169* | XM_359104.1 | | 2.19 |
| *LOC386218* | XM_359123.1 | | 1.61 |
| *LOC386233* | XM_359128.1 | | 1.70 |
| *LOC386256* | XM_359142.1 | | 1.65 |
| *LOC386405* | XM_359211.1 | | 1.55 |
| *LOC633360* | XM_907756.3 | PREDICTED: hypothetical LOC633360 (LOC633360) | 1.67 |
| *LOC633966* | XM_908539.2 | PREDICTED: similar to guanine nucleotide binding protein-like 2 (nucleolar) (LOC633966) | 1.54 |
| *LOC638935* | XM_915139.3 | PREDICTED: similar to peptidylarginine deiminase, type II (LOC638935) | 1.70 |
| *LOC667370* | XM_001480084.1 | PREDICTED: similar to interferon-induced protein with tetratricopeptide repeats 3 (LOC667370) | 2.71 |
| *LOC675572* | XM_001481199.1 | PREDICTED: hypothetical LOC675572 (LOC675572) | 7.85 |
| *LOC677369* | XR_005046.1 | PREDICTED: similar to Alpha-2-macroglobulin (LOC677369), misc RNA. | 3.29 |
| *LOC677375* | XM_001002943.1 | PREDICTED: hypothetical LOC677375 (LOC677375) | 2.34 |
| *Lrp2* | NM_001081088.1 | low density lipoprotein receptor-related protein 2 (Lrp2) | 3.57 |
| *Lrrc23* | NM_013588.1 | leucine rich repeat containing 23 (Lrrc23) | 1.69 |
| *Lrrc36* | NM_001033371.2 | leucine rich repeat containing 36 (Lrrc36) | 1.69 |
| *Lrrc48* | NM_029044.1 | leucine rich repeat containing 48 (Lrrc48) | 2.28 |
| *Lrrc51* | XM_890234.2 | PREDICTED: leucine rich repeat containing 51, transcript variant 2 (Lrrc51) | 2.23 |
| *Lrrc8* | NM_177725.2 | | 2.20 |
| *Lrrn4cl* | NM_001013019.2 | LRRN4 C-terminal like (Lrrn4cl) | 1.57 |
| *Luzp2* | NM_178705.5 | leucine zipper protein 2 (Luzp2) | 1.60 |
| *Lxn* | NM_016753.4 | latexin (Lxn) | 2.44 |
| *Macf1* | XM_110503 | | 1.62 |
| *Macrod1* | NM_134147.3 | MACRO domain containing 1 (Macrod1) | 2.11 |
| *Man1c1* | NM_207237 | | 1.55 |
| *Map1lc3b* | NM_026160.3 | microtubule-associated protein 1 light chain 3 beta (Map1lc3b) | 1.92 |
| *Map3k7ip2* | NM_138667.2 | mitogen-activated protein kinase kinase kinase 7 interacting protein 2 (Map3k7ip2) | 1.88 |
| *Mapk1ip1* | NM_001045483.1 | mitogen-activated protein kinase 1 interacting protein 1 (Mapk1ip1), transcript variant 1 | 1.53 |
| *Mapt* | NM_010838.2 | | 1.97 |
| *March 2* | NM_145486.4 | membrane-associated ring finger (C3HC4) 2 (March2) | 1.60 |
| *Matn4* | NM_013592.2 | matrilin 4 (Matn4) | 2.50 |
| *Mdh1b* | NM_029696.3 | malate dehydrogenase 1B, NAD (soluble) (Mdh1b) | 2.09 |
| *Med25* | NM_029365.1 | mediator of RNA polymerase II transcription, subunit 25 homolog (yeast) (Med25) | 1.50 |
| *Mfsd7b* | NM_001081259.1 | major facilitator superfamily domain containing 7B (Mfsd7b) | 4.93 |
| *Mgmt* | NM_008598.1 | | 1.64 |
| *Mgst1* | NM_019946.4 | microsomal glutathione S-transferase 1 (Mgst1) | 2.54 |
| *Mia1* | NM_019394 | | 1.75 |
| *Mib2* | NM_145124.2 | mindbomb homolog 2 (Drosophila) (Mib2) | 1.72 |
| *Mid1ip1* | NM_026524.2 | Mid1 interacting protein 1 (gastrulation specific G12-like (zebrafish)) (Mid1ip1) | 2.04 |
| *Mif4gd* | NM_027162.3 | MIF4G domain containing (Mif4gd) | 1.79 |
| *Mkrn1* | NM_018810.2 | makorin, ring finger protein, 1 (Mkrn1) | 1.63 |
| *Mks1* | NM_001039684.2 | Meckel syndrome, type 1 (Mks1) | 1.69 |
| *Mlf1* | NM_001039543.1 | myeloid leukemia factor 1 (Mlf1), transcript variant 1 | 2.32 |
| *Mmd2* | NM_175217.6 | monocyte to macrophage differentiation-associated 2 (Mmd2) | 4.55 |
| *Mmp11* | NM_008606.2 | matrix metallopeptidase 11 (Mmp11) | 1.81 |
| *Mobkl2b* | NM_178061.4 | MOB1, Mps One Binder kinase activator-like 2B (yeast) (Mobkl2b) | 1.65 |
| *Morn2* | NM_194269.1 | MORN repeat containing 2 (Morn2) | 2.02 |
| *Morn5* | NM_029309.2 | MORN repeat containing 5 (Morn5) | 1.94 |
| *Mpdz* | NM_010820.2 | multiple PDZ domain protein (Mpdz) | 1.67 |
| *Mpp6* | NM_019939.1 | membrane protein, palmitoylated 6 (MAGUK p55 subfamily member 6) (Mpp6) | 2.93 |
| *Msi2h* | AK031365 | | 1.65 |
| *Mt2* | NM_008630.2 | metallothionein 2 (Mt2) | 3.06 |
| *Mt3* | NM_013603.1 | metallothionein 3 (Mt3) | 3.73 |
| *Mtap6* | NM_001043355.2 | microtubule-associated protein 6 (Mtap6), transcript variant 3 | 1.52 |
| *Mvp* | NM_080638.1 | major vault protein (Mvp) | 2.00 |
| *Myadm* | NM_016969.1 | myeloid-associated differentiation marker (Myadm) | 3.42 |
| *Myo1e* | NM_181072 | | 1.75 |
| *Myo7a* | NM_008663.2 | myosin VIIa (Myo7a) | 1.50 |
| *Myst4* | NM_017479.2 | MYST histone acetyltransferase monocytic leukemia 4 (Myst4) | 1.56 |
| *Nacc2* | NM_001037098.1 | nucleus accumbens associated 2, BEN and BTB (POZ) domain containing (Nacc2), transcript variant 2 | 1.65 |
| *Nbl1* | NM_008675.1 | neuroblastoma, suppression of tumorigenicity 1 (Nbl1) | 4.82 |
| *Ndrg1* | NM_008681.2 | N-myc downstream regulated gene 1 (Ndrg1) | 2.53 |
| *Ndrg2* | NM_013864 | | 7.25 |
| *Ndrl* | NM_008681 | | 2.39 |
| *Ndst1* | NM_008306 | | 1.56 |

| | | | |
|---|---|---|---|
| *Nedd4l* | NM_031881.1 | neural precursor cell expressed, developmentally down-regulated gene 4-like (Nedd4l) | 1.90 |
| *Nedd9* | NM_017464.2 | neural precursor cell expressed, developmentally down-regulated gene 9 (Nedd9) | 1.56 |
| *Nek5* | NM_177898.4 | NIMA (never in mitosis gene a)-related expressed kinase 5 (Nek5) | 1.62 |
| *Nek9* | NM_145138.1 | NIMA (never in mitosis gene a)-related expressed kinase 9 (Nek9) | 1.57 |
| *Nfkbia* | NM_010907 | | 1.58 |
| *Ngef* | NM_019867 | | 3.57 |
| *Nicn1* | NM_025449.2 | nicolin 1 (Nicn1) | 1.58 |
| *Nid2* | NM_008695.2 | nidogen 2 (Nid2) | 2.85 |
| *Nme5* | NM_080637.3 | non-metastatic cells 5, protein expressed in (nucleoside-diphosphate kinase) (Nme5) | 5.98 |
| *Nme7* | NM_138314.2 | non-metastatic cells 7, protein expressed in (nucleoside-diphosphate kinase) (Nme7), transcript variant 1 | 1.66 |
| *Nope* | NM_020043.2 | neighbor of Punc E11 (Nope) | 2.24 |
| *Npal3* | NM_028995.3 | NIPA-like domain containing 3 (Npal3) | 3.09 |
| *Npr2* | NM_173788.3 | natriuretic peptide receptor 2 (Npr2) | 1.61 |
| *Nrbp2* | NM_144847.1 | nuclear receptor binding protein 2 (Nrbp2) | 12.59 |
| *Nrxn3* | NM_172544.2 | neurexin III (Nrxn3) | 1.76 |
| *Ntrk2* | NM_008745.2 | neurotrophic tyrosine kinase, receptor, type 2 (Ntrk2), transcript variant 2 | 2.00 |
| *Ntsr2* | NM_008747.2 | neurotensin receptor 2 (Ntsr2) | 8.93 |
| *Nuak1* | NM_001004363.1 | NUAK family, SNF1-like kinase, 1 (Nuak1) | 4.94 |
| *Nuak2* | NM_028778.3 | NUAK family, SNF1-like kinase, 2 (Nuak2) | 3.56 |
| *Nudt10* | NM_001031664.1 | nudix (nucleoside diphosphate linked moiety X)-type motif 10 (Nudt10) | 1.63 |
| *Nxn* | NM_008750.4 | nucleoredoxin (Nxn) | 4.17 |
| *Oasl2* | NM_011854.1 | 2'-5' oligoadenylate synthetase-like 2 (Oasl2) | 1.68 |
| *Oat* | NM_016978.1 | ornithine aminotransferase (Oat) | 2.37 |
| *Ogn* | NM_008760.2 | osteoglycin (Ogn) | 1.90 |
| *Olfm1* | NM_001038612.1 | olfactomedin 1 (Olfm1), transcript variant 2 | 2.25 |
| *Olfml1* | NM_172907.2 | olfactomedin-like 1 (Olfml1) | 3.11 |
| *Oplah* | NM_153122.2 | 5-oxoprolinase (ATP-hydrolysing) (Oplah) | 2.19 |
| *Orc3l* | NM_015824.3 | origin recognition complex, subunit 3-like (S. cerevisiae) (Orc3l) | 1.78 |
| *Ormdl3* | NM_025661.3 | ORM1-like 3 (S. cerevisiae) (Ormdl3) | 1.73 |
| *Osbpl5* | NM_024289 | | 3.29 |
| *Osmr* | NM_011019.1 | oncostatin M receptor (Osmr) | 2.60 |
| *OTTMUSG0000002540* | NR_003564.1 | predicted gene, OTTMUSG00000025408 (OTTMUSG00000025408), non-coding RNA. | 3.57 |
| *Pacrg* | NM_027032.2 | Park2 co-regulated (Pacrg) | 5.00 |
| *Pacs2* | NM_001081170.1 | phosphofurin acidic cluster sorting protein 2 (Pacs2) | 1.88 |
| *Padi2* | NM_008812.1 | peptidyl arginine deiminase, type II (Padi2) | 1.81 |
| *Palld* | XM_974582.1 | PREDICTED: palladin, cytoskeletal associated protein, transcript variant 4 (Palld) | 1.99 |
| *Palm* | NM_023128.2 | paralemmin (Palm) | 2.05 |
| *Paqr7* | NM_027995.2 | progestin and adipoQ receptor family member VII (Paqr7) | 1.74 |
| *Parp14* | NM_001039530.1 | poly (ADP-ribose) polymerase family, member 14 (Parp14) | 1.55 |
| *Parp4* | XM_994286.2 | PREDICTED: poly (ADP-ribose) polymerase family, member 4 (Parp4) | 1.54 |
| *Pawr* | NM_054056.2 | PRKC, apoptosis, WT1, regulator (Pawr) | 2.91 |
| *Pbx1* | NM_183355.2 | pre B-cell leukemia transcription factor 1 (Pbx1), transcript variant a | 1.58 |
| *Pcbp4* | NM_021567.2 | poly(rC) binding protein 4 (Pcbp4) | 1.90 |
| *Pcdh10* | NM_011043.3 | protocadherin 10 (Pcdh10), transcript variant 4 | 1.66 |
| *Pcyt1b* | NM_177546.2 | phosphate cytidylyltransferase 1, choline, beta isoform (Pcyt1b), transcript variant 2 | 1.59 |
| *Pdgfrl* | NM_026840.2 | platelet-derived growth factor receptor-like (Pdgfrl) | 1.64 |
| *Pdk4* | NM_013743.2 | pyruvate dehydrogenase kinase, isoenzyme 4 (Pdk4) | 1.54 |
| *Pdlim2* | NM_145978.1 | PDZ and LIM domain 2 (Pdlim2) | 2.38 |
| *Pdlim7* | NM_026131 | | 1.87 |
| *Pdzrn3* | NM_018884.1 | PDZ domain containing RING finger 3 (Pdzrn3) | 2.24 |
| *Pea15* | NM_008556.1 | | 1.69 |
| *Peli2* | NM_033602 | | 2.18 |
| *Pfkfb2* | NM_008825.3 | 6-phosphofructo-2-kinase/fructose-2,6-biphosphatase 2 (Pfkfb2) | 1.65 |
| *Pfkfb4* | NM_173019.5 | 6-phosphofructo-2-kinase/fructose-2,6-biphosphatase 4 (Pfkfb4) | 1.98 |
| *Pfkl* | NM_008826.3 | phosphofructokinase, liver, B-type (Pfkl) | 1.56 |
| *Pgpep1* | NM_023217.2 | pyroglutamyl-peptidase I (Pgpep1) | 1.95 |
| *Phkg1* | NM_011079.2 | phosphorylase kinase gamma 1 (Phkg1) | 3.53 |
| *Phxr4* | NM_008835.1 | | 2.16 |
| *Pik3ip1* | NM_178149.4 | phosphoinositide-3-kinase interacting protein 1 (Pik3ip1) | 2.63 |
| *Pitpnm2* | NM_011256.1 | phosphatidylinositol transfer protein, membrane-associated 2 (Pitpnm2) | 2.93 |
| *Pitpnm3* | NM_001024927.2 | PITPNM family member 3 (Pitpnm3), transcript variant 1 | 1.67 |
| *Pkd2* | NM_008861.2 | polycystic kidney disease 2 (Pkd2) | 1.66 |
| *Pkmyt1* | NM_023058.3 | protein kinase, membrane associated tyrosine/threonine 1 (Pkmyt1) | 1.52 |
| *Pkp2* | NM_026163.1 | plakophilin 2 (Pkp2) | 1.74 |

| | | | |
|---|---|---|---|
| *Pkp4* | NM_175464.2 | plakophilin 4 (Pkp4), transcript variant 2 | 1.86 |
| *Pla2g15* | NM_133792.2 | phospholipase A2, group XV (Pla2g15) | 1.68 |
| *Pla2g7* | NM_013737.2 | phospholipase A2, group VII (platelet-activating factor acetylhydrolase, plasma) (Pla2g7) | 4.20 |
| *Plcd1* | NM_019676.1 | | 2.01 |
| *Plcd4* | NM_001081456.1 | phospholipase C, delta 4 (Plcd4), transcript variant 1 | 2.04 |
| *Plce1* | NM_019588.2 | phospholipase C, epsilon 1 (Plce1) | 1.60 |
| *Pld2* | NM_008876.2 | phospholipase D2 (Pld2) | 1.61 |
| *Plekha2* | NM_031257.2 | pleckstrin homology domain-containing, family A (phosphoinositide binding specific) member 2 (Plekha2) | 1.87 |
| *Plekhb1* | NM_013746.1 | pleckstrin homology domain containing, family B (evectins) member 1 (Plekhb1) | 3.58 |
| *Plekhm3* | NM_001039493.1 | pleckstrin homology domain containing, family M, member 3 (Plekhm3) | 1.56 |
| *Plp1* | NM_011123.2 | proteolipid protein (myelin) 1 (Plp1) | 1.50 |
| *Plscr2* | NM_008880.2 | phospholipid scramblase 2 (Plscr2) | 2.25 |
| *Plscr4* | NM_178711.2 | | 2.33 |
| *Pltp* | NM_011125.2 | phospholipid transfer protein (Pltp) | 2.08 |
| *Plxdc1* | NM_028199.2 | plexin domain containing 1 (Plxdc1) | 2.40 |
| *Plxnb1* | NM_172775.1 | plexin B1 (Plxnb1) | 1.69 |
| *Pnma2* | NM_175498.3 | paraneoplastic antigen MA2 (Pnma2) | 2.60 |
| *Podxl* | NM_013723.2 | podocalyxin-like (Podxl) | 1.81 |
| *Porcn* | NM_145908.1 | porcupine homolog (Drosophila) (Porcn), transcript variant Mporc-b | 1.51 |
| *Ppap2a* | NM_008247.2 | phosphatidic acid phosphatase 2a (Ppap2a), transcript variant 1 | 1.77 |
| *Ppap2b* | NM_080555.2 | phosphatidic acid phosphatase type 2B (Ppap2b) | 2.00 |
| *Ppapdc2* | NM_028922.2 | phosphatidic acid phosphatase type 2 domain containing 2 (Ppapdc2) | 1.71 |
| *Ppil6* | XM_994766.1 | PREDICTED: peptidylprolyl isomerase (cyclophilin)-like 6, transcript variant 2 (Ppil6) | 2.19 |
| *Ppm2c* | NM_001098231.1 | protein phosphatase 2C, magnesium dependent, catalytic subunit (Ppm2c), nuclear gene encoding mitochondrial protein, transcript variant 1 | 5.03 |
| *Ppp1r13b* | NM_011625.1 | protein phosphatase 1, regulatory (inhibitor) subunit 13B (Ppp1r13b) | 1.50 |
| *Ppp1r1b* | NM_144828.1 | protein phosphatase 1, regulatory (inhibitor) subunit 1B (Ppp1r1b) | 2.07 |
| *Ppp2r2b* | NM_028392.2 | protein phosphatase 2 (formerly 2A), regulatory subunit B (PR 52), beta isoform (Ppp2r2b), transcript variant 2 | 4.33 |
| *Pqlc3* | NM_172574.1 | PQ loop repeat containing (Pqlc3) | 1.81 |
| *Prdm16* | NM_027504.3 | PR domain containing 16 (Prdm16) | 1.64 |
| *Prdx6* | NM_007453.3 | peroxiredoxin 6 (Prdx6) | 1.90 |
| *Prelp* | NM_054077.3 | proline arginine-rich end leucine-rich repeat (Prelp) | 9.89 |
| *Prf1* | NM_011073.2 | perforin 1 (pore forming protein) (Prf1) | 2.62 |
| *Prkcdbp* | NM_028444.1 | protein kinase C, delta binding protein (Prkcdbp) | 1.57 |
| *Prnp* | NM_011170.1 | prion protein (Prnp) | 1.92 |
| *Prodh* | NM_011172.1 | | 2.57 |
| *Prom* | AK029921 | | 2.10 |
| *Prosapip1* | NM_197945.2 | ProSAPiP1 protein (Prosapip1) | 2.07 |
| *Prps2* | NM_026662.4 | phosphoribosyl pyrophosphate synthetase 2 (Prps2) | 1.74 |
| *Psap* | NM_011179.2 | prosaposin (Psap) | 1.62 |
| *Psd2* | NM_028707.3 | pleckstrin and Sec7 domain containing 2 (Psd2) | 7.68 |
| *Psg23* | NM_020261.2 | pregnancy-specific glycoprotein 23 (Psg23) | 1.57 |
| *Pstpip1* | NM_011193.1 | proline-serine-threonine phosphatase-interacting protein 1 (Pstpip1) | 1.65 |
| *Punc* | NM_008988.2 | putative neuronal cell adhesion molecule (Punc) | 2.24 |
| *Pvalb* | NM_013645.3 | parvalbumin (Pvalb) | 6.87 |
| *Pygb* | NM_153781.1 | brain glycogen phosphorylase (Pygb) | 5.82 |
| *Rab11b* | NM_008997.3 | RAB11B, member RAS oncogene family (Rab11b) | 1.95 |
| *Rab11fip4* | NM_175543 | | 2.29 |
| *Rab32* | NM_026405.3 | RAB32, member RAS oncogene family (Rab32) | 1.57 |
| *Rab3d* | NM_031874.4 | RAB3D, member RAS oncogene family (Rab3d) | 1.92 |
| *Rab6b* | NM_173781.3 | | 1.61 |
| *Rab7l1* | NM_144875.1 | RAB7, member RAS oncogene family-like 1 (Rab7l1) | 1.60 |
| *Rabl2a* | NM_026817.3 | RAB, member of RAS oncogene family-like 2A (Rabl2a) | 1.80 |
| *Rabl5* | NM_026073.2 | | 1.51 |
| *Rage* | NM_011973.2 | renal tumor antigen (Rage) | 2.20 |
| *Ralgps1* | NM_175211 | | 2.08 |
| *Rapgef3* | NM_144850.1 | Rap guanine nucleotide exchange factor (GEF) 3 (Rapgef3) | 1.96 |
| *Rarres2* | NM_027852.2 | retinoic acid receptor responder (tazarotene induced) 2 (Rarres2) | 1.79 |
| *Rassf4* | NM_178045.3 | Ras association (RalGDS/AF-6) domain family member 4 (Rassf4) | 6.70 |
| *Rcsd1* | NM_178593.3 | RCSD domain containing 1 (Rcsd1), transcript variant 1 | 1.99 |
| *Rdh5* | NM_134006.4 | retinol dehydrogenase 5 (Rdh5) | 2.62 |
| *Reep1* | NM_178608.2 | receptor accessory protein 1 (Reep1) | 1.81 |
| *Reep5* | NM_007874.2 | receptor accessory protein 5 (Reep5) | 2.14 |
| *Reln* | NM_011261.2 | reelin (Reln) | 3.28 |
| *Retsat* | NM_026159.4 | retinol saturase (all trans retinol 13,14 reductase) (Retsat) | 1.51 |
| *Rftn2* | NM_028713.1 | raftlin family member 2 (Rftn2) | 1.97 |

| | | | |
|---|---|---|---|
| *Rfx2* | NM_009056.1 | regulatory factor X, 2 (influences HLA class II expression) (Rfx2) | 4.18 |
| *Rgl2* | NM_009059.2 | ral guanine nucleotide dissociation stimulator-like 2 (Rgl2) | 1.86 |
| *Rgma* | NM_177740.4 | RGM domain family, member A (Rgma) | 2.33 |
| *Rgs4* | NM_009062.3 | regulator of G-protein signaling 4 (Rgs4) | 1.68 |
| *Rgs9* | NM_011268.2 | regulator of G-protein signaling 9 (Rgs9) | 1.89 |
| *Rhbdl2* | NM_183163.2 | rhomboid, veinlet-like 2 (Drosophila) (Rhbdl2) | 2.51 |
| *Rhob* | NM_007483.2 | ras homolog gene family, member B (Rhob) | 2.36 |
| *Rhou* | NM_133955.3 | ras homolog gene family, member U (Rhou) | 1.69 |
| *Rhpn1* | NM_008164.1 | rhophilin, Rho GTPase binding protein 1 (Rhpn1) | 1.52 |
| *Rhpn2* | NM_027897.3 | rhophilin, Rho GTPase binding protein 2 (Rhpn2) | 2.51 |
| *Rin2* | NM_028724.2 | Ras and Rab interactor 2 (Rin2) | 1.83 |
| *Rln1* | NM_011272.1 | relaxin 1 (Rln1) | 1.65 |
| *Rnase1* | NM_011271.2 | ribonuclease, RNase A family, 1 (pancreatic) (Rnase1) | 2.65 |
| *Rnf167* | NM_027445.1 | ring finger protein 167 (Rnf167) | 1.68 |
| *Rnf19a* | NM_013923.2 | ring finger protein 19A (Rnf19a) | 1.61 |
| *Rorc* | NM_011281.1 | RAR-related orphan receptor gamma (Rorc) | 1.75 |
| *Rpgr* | NM_011285.1 | retinitis pigmentosa GTPase regulator (Rpgr) | 1.75 |
| *Rps6ka5* | NM_153587.2 | ribosomal protein S6 kinase, polypeptide 5 (Rps6ka5) | 1.65 |
| *Rshl3* | XM_137041.6 | PREDICTED: radial spokehead-like 3 (Rshl3) | 4.55 |
| *Rsph1* | NM_025290.3 | radial spoke head 1 homolog (Chlamydomonas) (Rsph1) | 2.98 |
| *Rtn1* | NM_001007596.1 | reticulon 1 (Rtn1), transcript variant 2 | 1.89 |
| *S100a1* | NM_011309.3 | S100 calcium binding protein A1 (S100a1) | 2.35 |
| *S100a13* | NM_009113.3 | S100 calcium binding protein A13 (S100a13) | 1.56 |
| *S100a6* | NM_011313.2 | S100 calcium binding protein A6 (calcyclin) (S100a6) | 4.52 |
| *S3-12* | NM_020568.2 | plasma membrane associated protein, S3-12 (S3-12) | 1.54 |
| *Sall2* | NM_015772.2 | sal-like 2 (Drosophila) (Sall2) | 1.67 |
| *Samd9l* | XM_620286.3 | PREDICTED: sterile alpha motif domain containing 9-like, transcript variant 1 (Samd9l) | 3.18 |
| *Scd4* | NM_183216.3 | stearoyl-coenzyme A desaturase 4 (Scd4) | 2.11 |
| *Scg3* | NM_009130.1 | secretogranin III (Scg3) | 4.33 |
| *Scg5* | NM_009162.3 | secretogranin V (Scg5) | 1.68 |
| *scl0002540.1_6* | AK019418.1 | | 2.61 |
| *scl0003799.1_2* | AK076154.1 | | 1.97 |
| *Sdc2* | NM_008304.2 | syndecan 2 (Sdc2) | 2.18 |
| *Sema4g* | NM_011976.1 | sema domain, immunoglobulin domain (Ig), transmembrane domain (TM) and short cytoplasmic domain, (semaphorin) 4G (Sema4g) | 1.66 |
| *Sema6d* | NM_172537.2 | sema domain, transmembrane domain (TM), and cytoplasmic domain, (semaphorin) 6D (Sema6d), transcript variant 1 | 2.04 |
| *Serpina3n* | NM_009252.2 | serine (or cysteine) peptidase inhibitor, clade A, member 3N (Serpina3n) | 6.59 |
| *Serpinb6a* | NM_009254.2 | serine (or cysteine) peptidase inhibitor, clade B, member 6a (Serpinb6a) | 3.46 |
| *Serping1* | NM_009776.1 | serine (or cysteine) peptidase inhibitor, clade G, member 1 (Serping1) | 2.15 |
| *Sesn1* | NM_001013370.1 | sestrin 1 (Sesn1) | 2.89 |
| *Sft2d2* | NM_145512.3 | SFT2 domain containing 2 (Sft2d2) | 1.62 |
| *Sfxn5* | NM_178639.2 | sideroflexin 5 (Sfxn5) | 2.55 |
| *Sh3bp5l* | NM_024480.3 | SH3 binding domain protein 5 like (Sh3bp5l) | 1.58 |
| *Shisa4* | NM_175259.4 | shisa homolog 4 (Xenopus laevis) (Shisa4) | 2.07 |
| *Shroom2* | NM_172441.2 | shroom family member 2 (Shroom2) | 1.71 |
| *Shroom3* | NM_001077596.1 | shroom family member 3 (Shroom3), transcript variant 3 | 3.15 |
| *Sidt2* | NM_172257.3 | SID1 transmembrane family, member 2 (Sidt2) | 1.52 |
| *Sirpa* | NM_007547.2 | signal-regulatory protein alpha (Sirpa) | 1.80 |
| *Sirt2* | NM_022432.3 | sirtuin 2 (silent mating type information regulation 2, homolog) 2 (S. cerevisiae) (Sirt2) | 2.08 |
| *Sirt3* | NM_022433.1 | sirtuin 3 (silent mating type information regulation 2, homolog) 3 (S. cerevisiae) (Sirt3) | 1.70 |
| *Slc15a2* | XM_147213.1 | | 1.70 |
| *Slc16a9* | NM_025807.1 | solute carrier family 16 (monocarboxylic acid transporters), member 9 (Slc16a9) | 1.78 |
| *Slc22a4* | NM_019687.3 | solute carrier family 22 (organic cation transporter), member 4 (Slc22a4) | 3.22 |
| *Slc22a5* | NM_011396.2 | solute carrier family 22 (organic cation transporter), member 5 (Slc22a5) | 1.63 |
| *Slc24a4* | NM_172152 | | 1.73 |
| *Slc25a18* | NM_001081048.1 | solute carrier family 25 (mitochondrial carrier), member 18 (Slc25a18) | 2.19 |
| *Slc26a3* | AK018566 | | 1.80 |
| *Slc29a3* | NM_023596.3 | solute carrier family 29 (nucleoside transporters), member 3 (Slc29a3) | 1.69 |
| *Slc2a1* | NM_011400.2 | solute carrier family 2 (facilitated glucose transporter), member 1 (Slc2a1) | 3.79 |
| *Slc38a2* | NM_175121.3 | solute carrier family 38, member 2 (Slc38a2) | 2.15 |
| *Slc38a3* | NM_023805.2 | solute carrier family 38, member 3 (Slc38a3) | 1.90 |
| *Slc39a12* | NM_001012305.1 | solute carrier family 39 (zinc transporter), member 12 (Slc39a12) | 5.12 |
| *Slc39a13* | NM_026721.2 | solute carrier family 39 (metal ion transporter), member 13 (Slc39a13) | 1.73 |
| *Slc39a4* | NM_028064.2 | solute carrier family 39 (zinc transporter), member 4 (Slc39a4) | 1.93 |
| *Slc44a2* | NM_152808.2 | solute carrier family 44, member 2 (Slc44a2) | 2.40 |
| *Slc46a3* | NM_027872.3 | solute carrier family 46, member 3 (Slc46a3) | 2.38 |

| | | | |
|---|---|---|---|
| *Slc6a8* | NM_133987.1 | solute carrier family 6 (neurotransmitter transporter, creatine), member 8 (Slc6a8) | 1.61 |
| *Slc7a3* | NM_007515.2 | solute carrier family 7 (cationic amino acid transporter, y+ system), member 3 (Slc7a3) | 1.55 |
| *Slc8a1* | NM_011406.1 | | 1.71 |
| *Slco1a5* | NM_130861.2 | solute carrier organic anion transporter family, member 1a5 (Slco1a5) | 1.66 |
| *Smad1* | NM_008539.3 | MAD homolog 1 (Drosophila) (Smad1) | 1.55 |
| *Smarca2* | NM_011416.2 | SWI/SNF related, matrix associated, actin dependent regulator of chromatin, subfamily a, member 2 (Smarca2), transcript variant 1 | 1.64 |
| *Smoc1* | NM_022316.1 | SPARC related modular calcium binding 1 (Smoc1) | 1.58 |
| *Snapc3* | NM_029949.1 | small nuclear RNA activating complex, polypeptide 3 (Snapc3) | 1.51 |
| *Snph* | NM_198214.2 | syntaphilin (Snph) | 2.82 |
| *Snta1* | NM_009228.1 | syntrophin, acidic 1 (Snta1) | 3.16 |
| *Sntg2* | NM_172951.1 | syntrophin, gamma 2 (Sntg2) | 1.80 |
| *Soat1* | NM_009230.3 | sterol O-acyltransferase 1 (Soat1) | 1.89 |
| *Sobp* | NM_175407.3 | sine oculis-binding protein homolog (Drosophila) (Sobp) | 2.04 |
| *Socs3* | NM_007707.2 | suppressor of cytokine signaling 3 (Socs3) | 1.85 |
| *Sorbs1* | NM_009166.3 | sorbin and SH3 domain containing 1 (Sorbs1), transcript variant 1 | 2.44 |
| *Sorl1* | NM_011436.3 | sortilin-related receptor, LDLR class A repeats-containing (Sorl1) | 1.81 |
| *Sox21* | NM_145464.1 | SRY-box containing gene 21 (Sox21) | 1.58 |
| *Sox6* | AK084290 | | 1.59 |
| *Sox9* | NM_011448 | | 2.64 |
| *Spag1* | NM_012031.1 | sperm associated antigen 1 (Spag1) | 1.54 |
| *Spag6* | NM_015773.1 | sperm associated antigen 6 (Spag6) | 3.99 |
| *Spata13* | XM_147847.4 | | 1.69 |
| *Spg20* | NM_144895 | | 1.72 |
| *Spnb2* | NM_175836.2 | spectrin beta 2 (Spnb2), transcript variant 1 | 1.59 |
| *Spop* | NM_025287.2 | speckle-type POZ protein (Spop) | 2.27 |
| *Srgap3* | NM_080448.4 | SLIT-ROBO Rho GTPase activating protein 3 (Srgap3) | 3.59 |
| *Srl* | NM_175347.4 | sarcalumenin (Srl) | 2.88 |
| *Srr* | AK080830 | | 2.22 |
| *Sstr2* | NM_009217.1 | | 1.77 |
| *St5* | NM_001001326.1 | suppression of tumorigenicity 5 (St5), transcript variant 1 | 2.29 |
| *St8sia2* | NM_009181.1 | ST8 alpha-N-acetyl-neuraminide alpha-2,8-sialyltransferase 2 (St8sia2) | 2.18 |
| *Stard10* | NM_019990.4 | START domain containing 10 (Stard10) | 1.74 |
| *Stard13* | NM_146258.1 | StAR-related lipid transfer (START) domain containing 13 (Stard13) | 1.87 |
| *Stard8* | NM_199018.1 | START domain containing 8 (Stard8) | 4.58 |
| *Stk36* | NM_175031.3 | serine/threonine kinase 36 (fused homolog, Drosophila) (Stk36) | 2.01 |
| *Stxbp2* | NM_011503.2 | | 1.69 |
| *Sulf1* | NM_172294.1 | sulfatase 1 (Sulf1) | 3.02 |
| *Svop* | NM_026805.1 | SV2 related protein (Svop) | 1.91 |
| *Syap1* | NM_025932.1 | synapse associated protein 1 (Syap1) | 2.82 |
| *Synm* | NM_183312.3 | synemin, intermediate filament protein (Synm), transcript variant 3 | 2.10 |
| *Tagln3* | NM_019754.3 | transgelin 3 (Tagln3) | 2.29 |
| *Tap2* | NM_011530.2 | transporter 2, ATP-binding cassette, sub-family B (MDR/TAP) (Tap2) | 2.27 |
| *Tbc1d2b* | NM_194334.2 | TBC1 domain family, member 2B (Tbc1d2b) | 1.87 |
| *Tbcel* | NM_173038.3 | tubulin folding cofactor E-like (Tbcel) | 3.17 |
| *Tceal6* | NM_025355.2 | transcription elongation factor A (SII)-like 6 (Tceal6) | 1.88 |
| *Tcfcp2l1* | NM_023755.2 | transcription factor CP2-like 1 (Tcfcp2l1) | 2.37 |
| *Tecta* | NM_009347.1 | tectorin alpha (Tecta) | 1.54 |
| *Tekt1* | NM_011569.2 | tektin 1 (Tekt1) | 2.47 |
| *Tens1* | XM_109868 | | 1.60 |
| *Tex264* | NM_011573.2 | testis expressed gene 264 (Tex264), transcript variant 1 | 1.67 |
| *Tgm2* | NM_009373.3 | transglutaminase 2, C polypeptide (Tgm2) | 2.68 |
| *Thra* | XM_126580.1 | | 1.57 |
| *Timp2* | NM_011594.3 | tissue inhibitor of metalloproteinase 2 (Timp2) | 3.06 |
| *Timp3* | NM_011595.2 | tissue inhibitor of metalloproteinase 3 (Timp3) | 2.02 |
| *Tjp2* | NM_011597.2 | tight junction protein 2 (Tjp2) | 1.50 |
| *Tlr2* | NM_011905.2 | toll-like receptor 2 (Tlr2) | 2.11 |
| *Tlr3* | NM_126166.4 | toll-like receptor 3 (Tlr3) | 1.53 |
| *Tm2d2* | NM_027194.2 | TM2 domain containing 2 (Tm2d2) | 1.64 |
| *Tmem107* | NM_025838.1 | transmembrane protein 107 (Tmem107), transcript variant 1 | 1.63 |
| *Tmem108* | NM_178638.2 | transmembrane protein 108 (Tmem108) | 1.88 |
| *Tmem166* | NM_145570.1 | transmembrane protein 166 (Tmem166) | 1.65 |
| *Tmem25* | NM_027865.1 | transmembrane protein 25 (Tmem25) | 1.83 |
| *Tmem43* | NM_028766.2 | transmembrane protein 43 (Tmem43) | 1.72 |
| *Tmem47* | NM_138751.1 | transmembrane protein 47 (Tmem47) | 4.11 |
| *Tmem50a* | NM_027935.2 | transmembrane protein 50A (Tmem50a) | 1.74 |
| *Tmem66* | NM_026432.2 | transmembrane protein 66 (Tmem66) | 1.54 |
| *Tmie* | NM_146260.2 | transmembrane inner ear (Tmie) | 2.85 |
| *Tmod2* | NM_001038710.1 | tropomodulin 2 (Tmod2), transcript variant 1 | 1.82 |

| | | | |
|---|---|---|---|
| *Tmtc4* | NM_028651.2 | transmembrane and tetratricopeptide repeat containing 4 (Tmtc4) | 1.53 |
| *Tmub1* | NM_022418.1 | transmembrane and ubiquitin-like domain containing 1 (Tmub1) | 1.66 |
| *Tnfrsf6* | AK086933 | | 1.51 |
| *Tns3* | NM_001083587.1 | tensin 3 (Tns3) | 1.92 |
| *Tpbg* | NM_011627.3 | trophoblast glycoprotein (Tpbg) | 1.84 |
| *Tpp1* | NM_009906.4 | tripeptidyl peptidase I (Tpp1) | 2.04 |
| *Tppp3* | NM_026481.2 | tubulin polymerization-promoting protein family member 3 (Tppp3) | 2.60 |
| *Tprgl* | NM_026388.2 | transformation related protein 63 regulated like (Tprgl) | 1.62 |
| *Tprkb* | NM_176842.2 | Tp53rk binding protein (Tprkb) | 2.06 |
| *Traf1* | AK089281 | | 6.20 |
| *Trip6* | NM_011639.2 | thyroid hormone receptor interactor 6 (Trip6) | 1.72 |
| *Trp53inp1* | NM_021897.1 | transformation related protein 53 inducible nuclear protein 1 (Trp53inp1) | 1.51 |
| *Trpc7* | NM_012035.2 | transient receptor potential cation channel, subfamily C, member 7 (Trpc7) | 1.51 |
| *Trps1* | AK036590 | | 1.97 |
| *Tsc22d3* | NM_001077364.1 | TSC22 domain family, member 3 (Tsc22d3), transcript variant 1 | 1.89 |
| *Tsc22d4* | NM_023910.5 | TSC22 domain family, member 4 (Tsc22d4) | 2.23 |
| *Tspan14* | NM_145928.1 | tetraspanin 14 (Tspan14) | 1.82 |
| *Tspan15* | NM_197996.2 | tetraspanin 15 (Tspan15) | 2.48 |
| *Tspan17* | NM_028841.1 | tetraspanin 17 (Tspan17) | 2.69 |
| *Tspan6* | NM_019656.3 | tetraspanin 6 (Tspan6) | 1.59 |
| *Tst* | NM_009437.4 | thiosulfate sulfurtransferase, mitochondrial (Tst), nuclear gene encoding mitochondrial protein | 1.64 |
| *Ttc17* | NM_183106.2 | tetratricopeptide repeat domain 17 (Ttc17) | 1.54 |
| *Ttc30b* | NM_028235.1 | tetratricopeptide repeat domain 30B (Ttc30b) | 3.37 |
| *Ttc8* | NM_198311.1 | tetratricopeptide repeat domain 8 (Ttc8), transcript variant 2 | 1.52 |
| *Ttll3* | NM_133923.3 | tubulin tyrosine ligase-like family, member 3 (Ttll3) | 3.86 |
| *Ttyh1* | NM_021324.4 | tweety homolog 1 (Drosophila) (Ttyh1), transcript variant 2 | 4.28 |
| *Ttyh2* | NM_053273.2 | tweety homolog 2 (Drosophila) (Ttyh2) | 3.05 |
| *Tuba1a* | NM_011653.1 | tubulin, alpha 1A (Tuba1a) | 1.83 |
| *Tuba3a* | NM_009446.2 | tubulin, alpha 3A (Tuba3a) | 1.52 |
| *Tubb2c* | NM_146116.1 | tubulin, beta 2c (Tubb2c) | 1.72 |
| *Txnip* | NM_001009935.2 | thioredoxin interacting protein (Txnip), transcript variant 1 | 2.96 |
| *Tyki* | NM_020557.3 | | 2.30 |
| *Uap1l1* | NM_001033293.2 | UDP-N-acteylglucosamine pyrophosphorylase 1-like 1 (Uap1l1) | 1.54 |
| *Ube2e2* | NM_144839.1 | ubiquitin-conjugating enzyme E2E 2 (UBC4/5 homolog, yeast) (Ube2e2) | 1.51 |
| *Ugp2* | NM_139297.4 | UDP-glucose pyrophosphorylase 2 (Ugp2) | 1.51 |
| *Ugt1a10* | NM_201641.2 | UDP glycosyltransferase 1 family, polypeptide A10 (Ugt1a10) | 3.77 |
| *Ugt1a6b* | NM_201410.1 | UDP glucuronosyltransferase 1 family, polypeptide A6B (Ugt1a6b) | 1.54 |
| *Unc45a* | NM_133952.1 | unc-45 homolog A (C. elegans) (Unc45a) | 1.64 |
| *Usp20* | NM_028846.3 | ubiquitin specific peptidase 20 (Usp20) | 1.56 |
| *Usp53* | NM_133857.3 | ubiquitin specific peptidase 53 (Usp53) | 3.11 |
| *Vamp4* | NM_016796.2 | vesicle-associated membrane protein 4 (Vamp4) | 1.50 |
| *Vasn* | NM_139307.2 | vasorin (Vasn) | 2.17 |
| *Vim* | NM_011701.3 | vimentin (Vim) | 1.63 |
| *Vps37b* | NM_177876.4 | vacuolar protein sorting 37B (yeast) (Vps37b) | 1.57 |
| *Vps41* | NM_172120.2 | vacuolar protein sorting 41 (yeast) (Vps41) | 1.57 |
| *Vwa5a* | NM_172767.2 | von Willebrand factor A domain containing 5A (Vwa5a) | 2.27 |
| *Wbscr27* | NM_024479.2 | Williams Beuren syndrome chromosome region 27 (human) (Wbscr27) | 1.54 |
| *Wdr16* | NM_027963.2 | WD repeat domain 16 (Wdr16) | 1.83 |
| *Wdr45* | NM_172372.1 | WD repeat domain 45 (Wdr45) | 1.57 |
| *Wdr60* | NM_146039.3 | WD repeat domain 60 (Wdr60) | 1.53 |
| *Wdr69* | NM_027725.2 | WD repeat domain 69 (Wdr69) | 2.94 |
| *Wdr78* | NM_146254.2 | WD repeat domain 78 (Wdr78) | 3.86 |
| *Wdr92* | NM_178909.4 | WD repeat domain 92 (Wdr92) | 1.64 |
| *Wisp2* | NM_016873.1 | WNT1 inducible signaling pathway protein 2 (Wisp2) | 1.74 |
| *Wwc1* | NM_170779.1 | WW, C2 and coiled-coil domain containing 1 (Wwc1) | 2.66 |
| *Wwp2* | NM_025830.3 | WW domain containing E3 ubiquitin protein ligase 2 (Wwp2) | 1.64 |
| *X99384* | NM_013753.1 | cDNA sequence X99384 (X99384) | 1.85 |
| *Ybx3* | AK029441 | | 1.50 |
| *Ypel3* | NM_025347.1 | yippee-like 3 (Drosophila) (Ypel3) | 3.48 |
| *Zcchc18* | NM_025893.2 | zinc finger, CCHC domain containing 18 (Zcchc18), transcript variant 3 | 3.87 |
| *Zfp185* | NM_009549.2 | zinc finger protein 185 (Zfp185) | 1.52 |
| *Zfp288* | AK034574 | | 3.26 |
| *Zfp36* | NM_011756.4 | zinc finger protein 36 (Zfp36) | 1.86 |
| *Zfp36l1* | NM_007564.4 | zinc finger protein 36, C3H type-like 1 (Zfp36l1) | 1.97 |
| *Zfp608* | NM_175751.3 | zinc finger protein 608 (Zfp608) | 1.63 |
| *Zfp703* | XM_912161.3 | PREDICTED: zinc finger protein 703 (Zfp703) | 1.79 |
| *Zfp91-cntf* | NM_001039718.1 | zinc finger protein 91, ciliary neurotrophic factor transcription unit (Zfp91-cntf) | 12.63 |
| *Zhx1* | NM_009572.3 | zinc fingers and homeoboxes 1 (Zhx1), transcript variant 1 | 1.62 |
| *Zmym3* | NM_019831.2 | zinc finger, MYM-type 3 (Zmym3) | 1.64 |
| *Zxda* | NR_003292.1 | zinc finger, X-linked, duplicated A (Zxda), non-coding RNA. | 2.80 |
