# Supplementary material for: miR-30 Family miRNAs Mediate the Effect of Chronic Social Defeat Stress on Hippocampal Neurogenesis in Mouse Depression Model
Source: Front Mol Neurosci. 2019 Aug 8;12:188. doi: 10.3389/fnmol.2019.00188 (PMC6694739; doi:10.3389/fnmol.2019.00188)
Supplement: TABLE S10 — The list comprises of genes which demonstrated decreased expression (fold change ≥1.5 and p ≤ 0.05) in the differentiated cells from the late phase (day 7) when compared with the proliferating neurospheres. [file Table_10.pdf]

**Table S10. List of genes, which demonstrated decreased expression (fold change ≥ 1.5 and p ≤ 0.05) in the differentiated cells from the late phase (day 7) when compared with the proliferating neurospheres**

| Gene Symbol | Accession No. | Gene Definition | Fold Change |
|---|---|---|---|
| *0610007J10Rik* | AK018717 | | 0.51 |
| *0610010F05Rik* | NM_027860 | | 0.55 |
| *0610037L13Rik* | NM_028754.2 | RIKEN cDNA 0610037L13 gene (0610037L13Rik) | 0.64 |
| *0910001A06Rik* | NM_144846.4 | RIKEN cDNA 0910001A06 gene (0910001A06Rik) | 0.51 |
| *1100001F19Rik* | XM_124187.1 | | 0.61 |
| *1110001A07Rik* | NM_025377.1 | RIKEN cDNA 1110001A07 gene (1110001A07Rik) | 0.65 |
| *1110001C20Rik* | NM_177730 | | 0.42 |
| *1110003F05Rik* | | | 0.35 |
| *1110007M04Rik* | NM_026742.3 | RIKEN cDNA 1110007M04 gene (1110007M04Rik) | 0.35 |
| *1110008F13Rik* | NM_026124.1 | RIKEN cDNA 1110008F13 gene (1110008F13Rik) | 0.45 |
| *1110012D08Rik* | NM_178066.2 | RIKEN cDNA 1110012D08 gene (1110012D08Rik) | 0.47 |
| *1110018J23Rik* | XM_140742.4 | | 0.45 |
| *1110019N10Rik* | NM_026753.1 | RIKEN cDNA 1110019N10 gene (1110019N10Rik) | 0.52 |
| *1110020P15Rik* | NM_197979.2 | RIKEN cDNA 1110020P15 gene (1110020P15Rik) | 0.65 |
| *1110038B12Rik* | XM_922038.2 | PREDICTED: RIKEN cDNA 1110038B12 gene, transcript variant 6 (1110038B12Rik) | 0.48 |
| *1110038D17Rik* | NM_175133.1 | RIKEN cDNA 1110038D17 gene (1110038D17Rik) | 0.66 |
| *1110067D22Rik* | NM_173752.3 | RIKEN cDNA 1110067D22 gene (1110067D22Rik) | 0.48 |
| *1110070O15Rik* | AK075684 | | 0.63 |
| *1190002F15Rik* | | | 0.53 |
| *1190009E20Rik* | | | 0.54 |
| *1200003C05Rik* | NM_024205.1 | RIKEN cDNA 1200003C05 gene (1200003C05Rik) | 0.48 |
| *1500001M20Rik* | NM_026894.1 | RIKEN cDNA 1500001M20 gene (1500001M20Rik) | 0.54 |
| *1500012F01Rik* | NM_001081005.1 | RIKEN cDNA 1500012F01 gene (1500012F01Rik) | 0.48 |
| *1500032D16Rik* | NM_001083891.1 | RIKEN cDNA 1500032D16 gene (1500032D16Rik), transcript variant 2 | 0.66 |
| *1500034J01Rik* | XM_912953.3 | PREDICTED: RIKEN cDNA 1500034J01 gene (1500034J01Rik) | 0.66 |
| *1600012H06Rik* | NM_001083882.1 | RIKEN cDNA 1600012H06 gene (1600012H06Rik), transcript variant 4 | 0.61 |
| *1700011E24Rik* | XM_128789.5 | PREDICTED: RIKEN cDNA 1700011E24 gene, transcript variant 2 (1700011E24Rik) | 0.22 |
| *1700013A01Rik* | AK005934 | | 0.65 |
| *1700021F05Rik* | NM_026411.1 | RIKEN cDNA 1700021F05 gene (1700021F05Rik) | 0.67 |
| *1700025G04Rik* | NM_197990.2 | RIKEN cDNA 1700025G04 gene (1700025G04Rik) | 0.31 |
| *1700027M01Rik* | NM_023544 | | 0.66 |
| *1700029F09Rik* | XM_919077.2 | PREDICTED: RIKEN cDNA 1700029F09 gene, transcript variant 20 (1700029F09Rik) | 0.37 |
| *1700037H04Rik* | NM_026091.2 | RIKEN cDNA 1700037H04 gene (1700037H04Rik) | 0.66 |
| *1700041B20Rik* | XM_485065 | | 0.52 |
| *1700052N19Rik* | NM_024261.2 | RIKEN cDNA 1700052N19 gene (1700052N19Rik) | 0.65 |
| *1700086L19Rik* | XM_358420.3 | PREDICTED: RIKEN cDNA 1700086L19 gene (1700086L19Rik) | 0.53 |
| *1700113I22Rik* | NM_026865.2 | RIKEN cDNA 1700113I22 gene (1700113I22Rik) | 0.48 |
| *1810006K21Rik* | XM_983803.1 | PREDICTED: RIKEN cDNA 1810006K21 gene (1810006K21Rik) | 0.60 |
| *1810022K09Rik* | XM_485210 | | 0.60 |
| *1810055G02Rik* | NM_028077.2 | RIKEN cDNA 1810055G02 gene (1810055G02Rik) | 0.41 |
| *1810074P20Rik* | NM_026194.3 | RIKEN cDNA 1810074P20 gene (1810074P20Rik) | 0.66 |
| *2010003J03Rik* | NM_027236.1 | RIKEN cDNA 2010003J03 gene (2010003J03Rik) | 0.63 |
| *2010107E04Rik* | NM_027360.2 | RIKEN cDNA 2010107E04 gene (2010107E04Rik) | 0.60 |
| *2010309L07Rik* | | | 0.65 |
| *2010317E24Rik* | XM_130053.2 | | 0.48 |
| *2010321I05Rik* | | | 0.58 |
| *2010321M09Rik* | NM_175153.3 | RIKEN cDNA 2010321M09 gene (2010321M09Rik), transcript variant 1 | 0.59 |
| *2210412D01Rik* | NM_133722.1 | RIKEN cDNA 2210412D01 gene (2210412D01Rik) | 0.59 |
| *2300002D11Rik* | NM_001081156.1 | RIKEN cDNA 2300002D11 gene (2300002D11Rik) | 0.62 |
| *2310003L22Rik* | NM_027093.3 | RIKEN cDNA 2310003L22 gene (2310003L22Rik) | 0.62 |
| *2310005E10Rik* | NM_172398.3 | RIKEN cDNA 2310005E10 gene (2310005E10Rik) | 0.44 |
| *2310008M10Rik* | NM_025509.2 | RIKEN cDNA 2310008M10 gene (2310008M10Rik) | 0.62 |
| *2310009B15Rik* | NM_001081226.1 | RIKEN cDNA 2310009B15 gene (2310009B15Rik) | 0.59 |
| *2310011G17Rik* | | | 0.62 |
| *2310014H01Rik* | XM_001003622.2 | PREDICTED: RIKEN cDNA 2310014H01 gene, transcript variant 3 (2310014H01Rik) | 0.36 |
| *2310016C08Rik* | NM_023516.3 | RIKEN cDNA 2310016C08 gene (2310016C08Rik) | 0.41 |
| *2310036D04Rik* | XM_488563 | | 0.60 |
| *2310037I24Rik* | NM_133714.2 | RIKEN cDNA 2310037I24 gene (2310037I24Rik) | 0.62 |
| *2310039H08Rik* | NM_025966.3 | RIKEN cDNA 2310039H08 gene (2310039H08Rik) | 0.64 |
| *2310044H10Rik* | NM_197991 | | 0.62 |
| *2310045K21Rik* | AK009823 | | 0.53 |
| *2400010C15Rik* | AK010307 | | 0.60 |

| | | | |
|---|---|---|---|
| *2410003K15Rik* | XM_132537.3 | | 0.42 |
| *2410006H16Rik* | XM_001479267.1 | PREDICTED: RIKEN cDNA 2410006H16 gene (2410006H16Rik) | 0.65 |
| *2410015N17Rik* | NM_023203.1 | RIKEN cDNA 2410015N17 gene (2410015N17Rik) | 0.33 |
| *2410016O06Rik* | NM_023633 | | 0.63 |
| *2510009E07Rik* | NM_001001881.1 | RIKEN cDNA 2510009E07 gene (2510009E07Rik) | 0.45 |
| *2600005C20Rik* | NM_028244 | | 0.44 |
| *2610002D18Rik* | XM_131720.2 | | 0.65 |
| *2610017I09Rik* | XR_035175.1 | PREDICTED: RIKEN cDNA 2610017I09 gene (2610017I09Rik), misc RNA. | 0.46 |
| *2610019E17Rik* | | | 0.44 |
| *2610024B07Rik* | | | 0.45 |
| *2610024G14Rik* | NM_019836.2 | RIKEN cDNA 2610024G14 gene (2610024G14Rik) | 0.52 |
| *2610024H22Rik* | | | 0.61 |
| *2610029G23Rik* | NM_026312.2 | RIKEN cDNA 2610029G23 gene (2610029G23Rik) | 0.57 |
| *2610036L11Rik* | XM_488549.3 | PREDICTED: RIKEN cDNA 2610036L11 gene, transcript variant 1 (2610036L11Rik) | 0.27 |
| *2610039C10Rik* | NM_025642.1 | RIKEN cDNA 2610039C10 gene (2610039C10Rik) | 0.34 |
| *2610101N10Rik* | NM_026476.1 | RIKEN cDNA 2610101N10 gene (2610101N10Rik) | 0.64 |
| *2610103J23Rik* | NM_026002 | | 0.62 |
| *2610207P08Rik* | XM_127746.2 | | 0.47 |
| *2610307O08Rik* | XM_921606.2 | PREDICTED: RIKEN cDNA 2610307O08 gene, transcript variant 12 (2610307O08Rik) | 0.30 |
| *2610510J17Rik* | NM_028131.1 | RIKEN cDNA 2610510J17 gene (2610510J17Rik) | 0.38 |
| *2700029M09Rik* | XM_001474993.1 | PREDICTED: RIKEN cDNA 2700029M09 gene (2700029M09Rik) | 0.50 |
| *2700055A20Rik* | NM_027314 | | 0.66 |
| *2700092H06Rik* | XM_132529.2 | | 0.65 |
| *2700094K13Rik* | NM_001037279.1 | RIKEN cDNA 2700094K13 gene (2700094K13Rik), transcript variant 2 | 0.16 |
| *2810025M15Rik* | XR_035184.1 | PREDICTED: RIKEN cDNA 2810025M15 gene (2810025M15Rik), misc RNA. | 0.37 |
| *2810026P18Rik* | | | 0.63 |
| *2810405J04Rik* | NM_133747.2 | RIKEN cDNA 2810405J04 gene (2810405J04Rik) | 0.49 |
| *2810417H13Rik* | NM_026515.2 | RIKEN cDNA 2810417H13 gene (2810417H13Rik) | 0.15 |
| *2810417K24Rik* | AK013109 | | 0.37 |
| *2810423A18Rik* | | | 0.62 |
| *2810452K22Rik* | NM_026048.2 | RIKEN cDNA 2810452K22 gene (2810452K22Rik) | 0.62 |
| *2900010M23Rik* | NM_026063.1 | RIKEN cDNA 2900010M23 gene (2900010M23Rik) | 0.65 |
| *2900056M07Rik* | AK013705 | | 0.62 |
| *2900060B14Rik* | | | 0.56 |
| *2900060P06Rik* | | | 0.39 |
| *2900072M03Rik* | | | 0.53 |
| *3000004C01Rik* | NM_197959.1 | RIKEN cDNA 3000004C01 gene (3000004C01Rik) | 0.40 |
| *3010015K02Rik* | | | 0.67 |
| *3110009E18Rik* | NM_028439.1 | RIKEN cDNA 3110009E18 gene (3110009E18Rik) | 0.48 |
| *3110013H01Rik* | XM_203393.1 | | 0.48 |
| *3110035E14Rik* | NM_178399.4 | RIKEN cDNA 3110035E14 gene (3110035E14Rik) | 0.60 |
| *3110043A19Rik* | XM_001476479.1 | PREDICTED: RIKEN cDNA 3110043A19 gene (3110043A19Rik) | 0.55 |
| *3300001G02Rik* | NM_030093.1 | RIKEN cDNA 3300001G02 gene (3300001G02Rik) | 0.63 |
| *3632451O06Rik* | NM_026142.1 | RIKEN cDNA 3632451O06 gene (3632451O06Rik) | 0.33 |
| *3830612M24* | | | 0.14 |
| *3930401E15Rik* | AK076210 | | 0.60 |
| *4631422C13Rik* | XM_984472.1 | PREDICTED: RIKEN cDNA 4631422C13 gene (4631422C13Rik) | 0.64 |
| *4631427C17Rik* | NM_021414.3 | RIKEN cDNA 4631427C17 gene (4631427C17Rik) | 0.65 |
| *4632417K18Rik* | NM_026640.2 | RIKEN cDNA 4632417K18 gene (4632417K18Rik) | 0.27 |
| *4833424O15Rik* | NM_029425.1 | RIKEN cDNA 4833424O15 gene (4833424O15Rik) | 0.44 |
| *4833426J09Rik* | | | 0.66 |
| *4833427B12Rik* | XM_195728 | | 0.58 |
| *4930427A07Rik* | NM_134041.2 | RIKEN cDNA 4930427A07 gene (4930427A07Rik) | 0.64 |
| *4930430F08Rik* | NM_175128 | | 0.67 |
| *4930487N19Rik* | AK015640 | | 0.65 |
| *4930547N16Rik* | NM_029249.2 | RIKEN cDNA 4930547N16 gene (4930547N16Rik) | 0.54 |
| *4930572J05Rik* | NM_198607.1 | RIKEN cDNA 4930572J05 gene (4930572J05Rik) | 0.46 |
| *4932417H02Rik* | NM_028898.2 | RIKEN cDNA 4932417H02 gene (4932417H02Rik) | 0.57 |
| *4933401P20Rik* | AK016608 | | 0.58 |
| *4933407C03Rik* | XM_001004724.2 | PREDICTED: RIKEN cDNA 4933407C03 gene, transcript variant 6 (4933407C03Rik) | 0.49 |
| *4933407N01Rik* | NM_025745.2 | | 0.66 |
| *4933407P14Rik* | NM_199019.1 | | 0.60 |
| *4933427G23Rik* | XR_005167.1 | PREDICTED: RIKEN cDNA 4933427G23 gene (4933427G23Rik), misc RNA. | 0.60 |
| *4933428A15Rik* | NM_027756 | | 0.60 |
| *5430417J04Rik* | AK030671 | | 0.61 |
| *5530401N12Rik* | | | 0.39 |
| *5730410I19Rik* | NM_025666.1 | RIKEN cDNA 5730410I19 gene (5730410I19Rik) | 0.62 |
| *5730427N09Rik* | NM_021552.4 | RIKEN cDNA 5730427N09 gene (5730427N09Rik) | 0.44 |

| | | | |
|---|---|---|---|
| *5730494J16Rik* | AK077637 | | 0.34 |
| *5830411I20* | | | 0.36 |
| *5930416I19Rik* | NM_197981.1 | RIKEN cDNA 5930416I19 gene (5930416I19Rik) | 0.51 |
| *6030405A18* | NM_177854 | | 0.56 |
| *6030405A18Rik* | NM_177854.2 | RIKEN cDNA 6030405A18 gene (6030405A18Rik) | 0.47 |
| *6230416J20Rik* | NM_173400 | | 0.64 |
| *6330509M05Rik* | | | 0.66 |
| *6330549D23Rik* | NR_003619.1 | RIKEN cDNA 6330549D23 gene (6330549D23Rik), non-coding RNA. | 0.59 |
| *6330569M22Rik* | NM_153563.1 | RIKEN cDNA 6330569M22 gene (6330569M22Rik) | 0.62 |
| *6330578E17Rik* | NM_198006.3 | RIKEN cDNA 6330578E17 gene (6330578E17Rik) | 0.50 |
| *6332401O19Rik* | NM_177013.3 | RIKEN cDNA 6332401O19 gene (6332401O19Rik) | 0.60 |
| *6430402L23Rik* | AK078178 | | 0.46 |
| *6430527G18Rik* | NM_145836.1 | RIKEN cDNA 6430527G18 gene (6430527G18Rik) | 0.44 |
| *6430537F04* | | | 0.31 |
| *6430706D22Rik* | NM_198652.1 | RIKEN cDNA 6430706D22 gene (6430706D22Rik) | 0.40 |
| *6530401D06Rik* | AK018306 | | 0.66 |
| *6530418L21Rik* | NM_175398.3 | RIKEN cDNA 6530418L21 gene (6530418L21Rik) | 0.40 |
| *6720458F09Rik* | NM_177374.2 | RIKEN cDNA 6720458F09 gene (6720458F09Rik) | 0.52 |
| *6720460F02Rik* | NM_144526.2 | RIKEN cDNA 6720460F02 gene (6720460F02Rik) | 0.66 |
| *6720463M24Rik* | NM_175265.4 | RIKEN cDNA 6720463M24 gene (6720463M24Rik) | 0.48 |
| *8030402P03Rik* | AK078743 | | 0.62 |
| *9030024J15Rik* | | | 0.48 |
| *9330134C04Rik* | NM_001013608.1 | RIKEN cDNA 9330134C04 gene (9330134C04Rik) | 0.64 |
| *9430008C03Rik* | XM_001004952.1 | PREDICTED: RIKEN cDNA 9430008C03 gene (9430008C03Rik) | 0.65 |
| *9430065L19Rik* | AK034952 | | 0.51 |
| *9430080K19Rik* | | | 0.46 |
| *9530018I07Rik* | | | 0.49 |
| *9530058B02Rik* | NM_026633.1 | RIKEN cDNA 9530058B02 gene (9530058B02Rik) | 0.64 |
| *9530064J02* | XM_489200 | | 0.13 |
| *9830001H06Rik* | XM_283804.7 | PREDICTED: RIKEN cDNA 9830001H06 gene (9830001H06Rik) | 0.66 |
| *9930014A18Rik* | NM_177166.2 | | 0.60 |
| *A230046K03Rik* | NM_001033375.2 | RIKEN cDNA A230046K03 gene (A230046K03Rik) | 0.61 |
| *A230079E18Rik* | AK038962 | | 0.47 |
| *A830059I20Rik* | NM_021427.1 | | 0.32 |
| *A930030J18Rik* | AK044660 | | 0.53 |
| *AA960436* | NM_133954.1 | expressed sequence AA960436 (AA960436) | 0.60 |
| *Aadacl1* | NM_178772.2 | arylacetamide deacetylase-like 1 (Aadacl1) | 0.63 |
| *Aard* | NM_175503.3 | alanine and arginine rich domain containing protein (Aard) | 0.32 |
| *Aars* | NM_146217.3 | alanyl-tRNA synthetase (Aars) | 0.48 |
| *Aatk* | NM_007377.3 | apoptosis-associated tyrosine kinase (Aatk) | 0.20 |
| *Abcb7* | XM_907304.2 | PREDICTED: ATP-binding cassette, sub-family B (MDR/TAP), member 7, transcript variant 5 (Abcb7) | 0.47 |
| *Abcf1* | NM_013854.1 | ATP-binding cassette, sub-family F (GCN20), member 1 (Abcf1) | 0.66 |
| *Abcf2* | NM_013853.1 | ATP-binding cassette, sub-family F (GCN20), member 2 (Abcf2), nuclear gene encoding mitochondrial protein | 0.63 |
| *Abi2* | NM_198127.1 | abl-interactor 2 (Abi2) | 0.46 |
| *Acadl* | NM_007381.3 | acyl-Coenzyme A dehydrogenase, long-chain (Acadl), nuclear gene encoding mitochondrial protein | 0.47 |
| *Acads* | NM_007383.2 | acyl-Coenzyme A dehydrogenase, short chain (Acads), nuclear gene encoding mitochondrial protein | 0.59 |
| *Acap3* | NM_207223.1 | ArfGAP with coiled-coil, ankyrin repeat and PH domains 3 (Acap3) | 0.39 |
| *Acat2* | NM_009338.3 | acetyl-Coenzyme A acetyltransferase 2 (Acat2) | 0.39 |
| *Acbd3* | NM_133225.2 | acyl-Coenzyme A binding domain containing 3 (Acbd3) | 0.65 |
| *Acly* | NM_134037.2 | ATP citrate lyase (Acly) | 0.46 |
| *Acn9* | XM_355744.1 | | 0.64 |
| *Aco2* | NM_080633.2 | aconitase 2, mitochondrial (Aco2), nuclear gene encoding mitochondrial protein | 0.51 |
| *Acot7* | NM_133348.1 | acyl-CoA thioesterase 7 (Acot7) | 0.51 |
| *Acpl2* | NM_153420.2 | acid phosphatase-like 2 (Acpl2) | 0.54 |
| *Acsbg1* | NM_053178.2 | acyl-CoA synthetase bubblegum family member 1 (Acsbg1) | 0.36 |
| *Acsl5* | NM_027976.2 | acyl-CoA synthetase long-chain family member 5 (Acsl5) | 0.43 |
| *Acss2* | NM_019811.3 | acyl-CoA synthetase short-chain family member 2 (Acss2) | 0.51 |
| *Actb* | NM_007393.1 | actin, beta, cytoplasmic (Actb) | 0.44 |
| *Actl6a* | NM_019673.1 | actin-like 6A (Actl6a) | 0.53 |
| *Actr3* | NM_023735.1 | ARP3 actin-related protein 3 homolog (yeast) (Actr3) | 0.56 |
| *Acy1* | NM_025371.1 | aminoacylase 1 (Acy1) | 0.67 |
| *Adam12* | NM_007400.2 | a disintegrin and metallopeptidase domain 12 (meltrin alpha) (Adam12) | 0.19 |
| *Adam15* | NM_009614.2 | a disintegrin and metallopeptidase domain 15 (metargidin) (Adam15), transcript variant 2 | 0.60 |
| *Adam17* | NM_009615.5 | a disintegrin and metallopeptidase domain 17 (Adam17) | 0.53 |

| | | | |
|---|---|---|---|
| *Adamts19* | NM_175506.3 | a disintegrin-like and metallopeptidase (reprolysin type) with thrombospondin type 1 motif, 19 (Adamts19) | 0.38 |
| *Adamts4* | NM_172845.1 | | 0.35 |
| *Adm* | NM_009627.1 | adrenomedullin (Adm) | 0.51 |
| *Adnp* | NM_009628.2 | activity-dependent neuroprotective protein (Adnp) | 0.62 |
| *Adpgk* | NM_028121.1 | | 0.57 |
| *Adrb2* | NM_007420.2 | adrenergic receptor, beta 2 (Adrb2) | 0.54 |
| *Adsl* | NM_009634 | | 0.62 |
| *Aes* | NM_010347.2 | amino-terminal enhancer of split (Aes) | 0.41 |
| *Afg3l1* | NM_054070.2 | AFG3(ATPase family gene 3)-like 1 (yeast) (Afg3l1) | 0.63 |
| *Agap1* | NM_001037136.1 | ArfGAP with GTPase domain, ankyrin repeat and PH domain 1 (Agap1), transcript variant 2 | 0.60 |
| *Agfg2* | NM_178162.2 | ArfGAP with FG repeats 2 (Agfg2), transcript variant 2 | 0.66 |
| *Ahctf1* | NM_026375.1 | AT hook containing transcription factor 1 (Ahctf1) | 0.56 |
| *Ahcy* | NM_016661 | | 0.52 |
| *Ahcyl1* | NM_145542.3 | S-adenosylhomocysteine hydrolase-like 1 (Ahcyl1) | 0.60 |
| *AI413582* | NM_001002895.2 | expressed sequence AI413582 (AI413582) | 0.60 |
| *AI448196* | XM_136041.3 | | 0.48 |
| *AI464131* | NM_001085515.1 | expressed sequence AI464131 (AI464131) | 0.62 |
| *AI481316* | XM_148986.1 | | 0.39 |
| *AI593442* | NM_177907.2 | expressed sequence AI593442 (AI593442), transcript variant 1 | 0.50 |
| *AI851790* | NM_182807.2 | expressed sequence AI851790 (AI851790) | 0.50 |
| *Ak2* | NM_001033966.3 | adenylate kinase 2 (Ak2), transcript variant 1 | 0.57 |
| *Akap12* | NM_031185.2 | A kinase (PRKA) anchor protein (gravin) 12 (Akap12) | 0.09 |
| *Akap7* | NM_018747.3 | A kinase (PRKA) anchor protein 7 (Akap7) | 0.42 |
| *Akirin1* | NM_023423.3 | akirin 1 (Akirin1) | 0.65 |
| *Alad* | NM_008525.3 | aminolevulinate, delta-, dehydratase (Alad) | 0.58 |
| *Aldh18a1* | NM_019698.1 | aldehyde dehydrogenase 18 family, member A1 (Aldh18a1), nuclear gene encoding mitochondrial protein, transcript variant 1 | 0.42 |
| *Aldh1a3* | AK043530 | | 0.57 |
| *Alg6* | NM_001081264.1 | asparagine-linked glycosylation 6 homolog (yeast, alpha-1,3,-glucosyltransferase) (Alg6) | 0.66 |
| *Alg8* | NM_199035.1 | asparagine-linked glycosylation 8 homolog (yeast, alpha-1,3-glucosyltransferase) (Alg8) | 0.53 |
| *Alg9* | NM_133981.1 | asparagine-linked glycosylation 9 homolog (yeast, alpha 1,2 mannosyltransferase) (Alg9) | 0.62 |
| *Alkbh3* | NM_026944.1 | alkB, alkylation repair homolog 3 (E. coli) (Alkbh3) | 0.63 |
| *Als2* | NM_028717.2 | amyotrophic lateral sclerosis 2 (juvenile) homolog (human) (Als2) | 0.66 |
| *Amotl1* | NM_001081395.1 | angiomotin-like 1 (Amotl1) | 0.41 |
| *Amph* | NM_175007.1 | amphiphysin (Amph) | 0.54 |
| *Anapc5* | NM_021505.2 | anaphase-promoting complex subunit 5 (Anapc5), transcript variant 1 | 0.61 |
| *Angpt2* | NM_007426.3 | angiopoietin 2 (Angpt2) | 0.04 |
| *Ank3* | NM_170687.3 | ankyrin 3, epithelial (Ank3), transcript variant 6 | 0.22 |
| *Ankrd49* | NM_019683.3 | ankyrin repeat domain 49 (Ankrd49) | 0.59 |
| *Anln* | NM_028390.2 | anillin, actin binding protein (Anln) | 0.22 |
| *Anp32e* | NM_023210.3 | acidic (leucine-rich) nuclear phosphoprotein 32 family, member E (Anp32e) | 0.34 |
| *Ap2b1* | NM_001035854.2 | adaptor-related protein complex 2, beta 1 subunit (Ap2b1), transcript variant 1 | 0.52 |
| *Apex1* | NM_009687.1 | apurinic/apyrimidinic endonuclease 1 (Apex1) | 0.65 |
| *Apitd1* | NM_027263.1 | apoptosis-inducing, TAF9-like domain 1 (Apitd1) | 0.66 |
| *Appbp2* | NM_025825 | | 0.54 |
| *Appl1* | NM_145221.2 | adaptor protein, phosphotyrosine interaction, PH domain and leucine zipper containing 1 (Appl1) | 0.65 |
| *Aprin* | NM_175310.5 | androgen-induced proliferation inhibitor (Aprin) | 0.46 |
| *Arc* | NM_018790.2 | activity regulated cytoskeletal-associated protein (Arc) | 0.56 |
| *Ard1* | NM_019870.1 | | 0.59 |
| *Ard1a* | NM_019870.2 | ARD1 homolog A, N-acetyltransferase (S. cerevisiae) (Ard1a) | 0.43 |
| *Areg* | NM_009704.3 | amphiregulin (Areg) | 0.24 |
| *Arf6* | NM_007481.3 | ADP-ribosylation factor 6 (Arf6) | 0.56 |
| *Arhgap18* | NM_176837.2 | Rho GTPase activating protein 18 (Arhgap18) | 0.23 |
| *Arhgap20* | NM_175535.3 | Rho GTPase activating protein 20 (Arhgap20) | 0.64 |
| *Arih1* | NM_019927.1 | ariadne ubiquitin-conjugating enzyme E2 binding protein homolog 1 (Drosophila) (Arih1) | 0.64 |
| *Arl4c* | NM_177305.3 | ADP-ribosylation factor-like 4C (Arl4c) | 0.63 |
| *Armcx2* | NM_026139.3 | armadillo repeat containing, X-linked 2 (Armcx2) | 0.63 |
| *Arpc1b* | NM_023142.1 | actin related protein 2/3 complex, subunit 1B (Arpc1b) | 0.33 |
| *Arpc3* | NM_019824.3 | actin related protein 2/3 complex, subunit 3 (Arpc3) | 0.45 |
| *Arpp21* | NM_028755.2 | cyclic AMP-regulated phosphoprotein, 21 (Arpp21), transcript variant 1 | 0.46 |
| *Asah1* | NM_019734.1 | N-acylsphingosine amidohydrolase 1 (Asah1) | 0.37 |
| *Asb1* | NM_023046.4 | ankyrin repeat and SOCS box-containing 1 (Asb1), transcript variant 2 | 0.65 |

| | | | |
|---|---|---|---|
| *Ascc3* | XM_125617.7 | PREDICTED: activating signal cointegrator 1 complex subunit 3, transcript variant 1 (Ascc3) | 0.66 |
| *Asns* | NM_012055.1 | asparagine synthetase (Asns) | 0.51 |
| *Aspm* | NM_009791.4 | asp (abnormal spindle)-like, microcephaly associated (Drosophila) (Aspm) | 0.35 |
| *Atad1* | NM_026487.3 | ATPase family, AAA domain containing 1 (Atad1) | 0.64 |
| *Atad3a* | NM_179203.1 | ATPase family, AAA domain containing 3A (Atad3a) | 0.59 |
| *Atf4* | NM_009716.2 | activating transcription factor 4 (Atf4) | 0.55 |
| *Atf7ip* | NM_019426.2 | activating transcription factor 7 interacting protein (Atf7ip) | 0.52 |
| *Atic* | NM_026195.1 | 5-aminoimidazole-4-carboxamide ribonucleotide formyltransferase/IMP cyclohydrolase (Atic) | 0.58 |
| *Atp13a3* | XM_001480958.1 | PREDICTED: ATPase type 13A3, transcript variant 1 (Atp13a3) | 0.59 |
| *Atp1b3* | NM_007502.3 | ATPase, Na+/K+ transporting, beta 3 polypeptide (Atp1b3) | 0.57 |
| *Atp5a1* | NM_007505.2 | ATP synthase, H+ transporting, mitochondrial F1 complex, alpha subunit, isoform 1 (Atp5a1), nuclear gene encoding mitochondrial protein | 0.57 |
| *Atp5c1* | NM_020615.3 | ATP synthase, H+ transporting, mitochondrial F1 complex, gamma polypeptide 1 (Atp5c1) | 0.63 |
| *Atp5d* | NM_025313.1 | ATP synthase, H+ transporting, mitochondrial F1 complex, delta subunit (Atp5d) | 0.57 |
| *Atp5f1* | NM_009725.3 | ATP synthase, H+ transporting, mitochondrial F0 complex, subunit b, isoform 1 (Atp5f1), nuclear gene encoding mitochondrial protein | 0.56 |
| *Atp5g2* | NM_026468.1 | ATP synthase, H+ transporting, mitochondrial F0 complex, subunit c (subunit 9), isoform 2 (Atp5g2) | 0.63 |
| *Atp5g3* | NM_175015.2 | ATP synthase, H+ transporting, mitochondrial F0 complex, subunit c (subunit 9), isoform 3 (Atp5g3), nuclear gene encoding mitochondrial protein | 0.66 |
| *Atp5j* | NM_016755.2 | ATP synthase, H+ transporting, mitochondrial F0 complex, subunit F (Atp5j), nuclear gene encoding mitochondrial protein | 0.64 |
| *Atp5l* | NM_013795.4 | ATP synthase, H+ transporting, mitochondrial F0 complex, subunit g (Atp5l), nuclear gene encoding mitochondrial protein | 0.57 |
| *Atp5o* | NM_138597.2 | ATP synthase, H+ transporting, mitochondrial F1 complex, O subunit (Atp5o), nuclear gene encoding mitochondrial protein | 0.61 |
| *Atp6v0a1* | NM_016920.1 | | 0.66 |
| *AU022870* | NM_177682.3 | expressed sequence AU022870 (AU022870) | 0.63 |
| *AU040320* | NM_133886.2 | expressed sequence AU040320 (AU040320), transcript variant 3 | 0.50 |
| *Aurka* | NM_011497.3 | aurora kinase A (Aurka) | 0.18 |
| *Aurkaip1* | NM_025338.3 | aurora kinase A interacting protein 1 (Aurkaip1) | 0.63 |
| *Aurkb* | NM_011496.1 | aurora kinase B (Aurkb) | 0.25 |
| *AW120700* | | | 0.38 |
| *B230114H05Rik* | AK045406 | | 0.53 |
| *B230208H17Rik* | NM_001024616.1 | RIKEN cDNA B230208H17 gene (B230208H17Rik) | 0.62 |
| *B230343A10Rik* | | | 0.46 |
| *B3galt6* | NM_080445.4 | UDP-Gal:betaGal beta 1,3-galactosyltransferase, polypeptide 6 (B3galt6) | 0.49 |
| *B3gnt5* | NM_054052.2 | UDP-GlcNAc:betaGal beta-1,3-N-acetylglucosaminyltransferase 5 (B3gnt5) | 0.58 |
| *B4galt3* | NM_020579.1 | UDP-Gal:betaGlcNAc beta 1,4-galactosyltransferase, polypeptide 3 (B4galt3) | 0.54 |
| *Bahcc1* | NM_198423.2 | BAH domain and coiled-coil containing 1 (Bahcc1) | 0.42 |
| *Bai2* | NM_173071.1 | brain-specific angiogenesis inhibitor 2 (Bai2) | 0.49 |
| *Banf1* | NM_001038231.1 | barrier to autointegration factor 1 (Banf1), transcript variant 2 | 0.28 |
| *BC002199* | NM_145964.2 | cDNA sequence BC002199 (BC002199) | 0.60 |
| *BC003236* | NM_030249 | | 0.47 |
| *BC003885* | NM_198609.2 | cDNA sequence BC003885 (BC003885) | 0.60 |
| *BC003965* | XM_128528.2 | | 0.55 |
| *BC017647* | NM_145430.1 | cDNA sequence BC017647 (BC017647) | 0.47 |
| *BC024659* | XM_001472442.1 | PREDICTED: cDNA sequence BC024659 (BC024659) | 0.58 |
| *BC025076* | NM_175002.1 | | 0.55 |
| *BC026590* | NM_001081420.1 | cDNA sequence BC026590 (BC026590) | 0.67 |
| *BC031781* | NM_145943.1 | cDNA sequence BC031781 (BC031781) | 0.66 |
| *BC046331* | NM_177673.2 | cDNA sequence BC046331 (BC046331) | 0.61 |
| *BC046404* | NM_198861.1 | cDNA sequence BC046404 (BC046404) | 0.57 |
| *BC060632* | NM_198625.1 | cDNA sequence BC060632 (BC060632) | 0.59 |
| *BC067047* | NM_177782.3 | cDNA sequence BC067047 (BC067047) | 0.42 |
| *BC085271* | NM_001008551.1 | cDNA sequence BC085271 (BC085271) | 0.44 |
| *Bcan* | NM_007529.1 | brevican (Bcan) | 0.42 |
| *Bcas1* | NM_029815.1 | breast carcinoma amplified sequence 1 (Bcas1) | 0.32 |
| *Bccip* | NM_025392.1 | BRCA2 and CDKN1A interacting protein (Bccip) | 0.63 |
| *Bcl7c* | NM_009746.2 | B-cell CLL/lymphoma 7C (Bcl7c) | 0.57 |
| *Bdnf* | NM_001048141.1 | brain derived neurotrophic factor (Bdnf), transcript variant 3 | 0.55 |
| *Bex2* | XM_977338.1 | PREDICTED: brain expressed X-linked 2 (Bex2) | 0.22 |
| *Bhlhb9* | NM_198161.1 | basic helix-loop-helix domain containing, class B9 (Bhlhb9) | 0.61 |
| *Bicd2* | NM_029791 | | 0.57 |
| *Bin1* | NM_009668.1 | bridging integrator 1 (Bin1) | 0.60 |
| *Birc5* | NM_009689.2 | baculoviral IAP repeat-containing 5 (Birc5), transcript variant 1 | 0.30 |

| | | | |
|---|---|---|---|
| *Blm* | NM_007550.3 | Bloom syndrome homolog (human) (Blm), transcript variant 1 | 0.39 |
| *Bloc1s2* | XM_193940.2 | | 0.63 |
| *Bmp7* | NM_007557.2 | bone morphogenetic protein 7 (Bmp7) | 0.64 |
| *Bmper* | NM_028472.1 | BMP-binding endothelial regulator (Bmper) | 0.51 |
| *Bnip2* | NM_001008238.2 | BCL2/adenovirus E1B interacting protein 2 (Bnip2), transcript variant alpha | 0.47 |
| *Bok* | NM_016778 | | 0.35 |
| *Bola2* | NM_175103.2 | bolA-like 2 (E. coli) (Bola2) | 0.59 |
| *Bola3* | NM_175277.2 | bolA-like 3 (E. coli) (Bola3) | 0.65 |
| *Bop1* | NM_013481.1 | block of proliferation 1 (Bop1) | 0.66 |
| *Bpnt1* | NM_011794.3 | bisphosphate 3'-nucleotidase 1 (Bpnt1) | 0.56 |
| *Brd2* | NM_001025387.2 | bromodomain containing 2 (Brd2), transcript variant 2 | 0.61 |
| *Brd3* | NM_023336 | | 0.65 |
| *Brip1* | NM_178309.1 | BRCA1 interacting protein C-terminal helicase 1 (Brip1) | 0.62 |
| *Brp17* | NM_019999.1 | | 0.53 |
| *Brrn1* | NM_144818 | | 0.20 |
| *Btaf1* | NM_001080706.1 | BTAF1 RNA polymerase II, B-TFIID transcription factor-associated, (Mot1 homolog, S. cerevisiae) (Btaf1) | 0.66 |
| *Btbd11* | NM_001017525.1 | BTB (POZ) domain containing 11 (Btbd11), transcript variant 2 | 0.38 |
| *Btbd12* | NM_177472.4 | BTB (POZ) domain containing 12 (Btbd12) | 0.67 |
| *Btf3* | NM_145455.1 | | 0.56 |
| *Btg1* | NM_007569.1 | B-cell translocation gene 1, anti-proliferative (Btg1) | 0.54 |
| *Bub1b* | NM_009773 | | 0.17 |
| *Bves* | NM_024285 | | 0.43 |
| *Bxdc1* | NM_023323 | | 0.56 |
| *Bxdc2* | NM_026396.2 | brix domain containing 2 (Bxdc2) | 0.53 |
| *Bxdc5* | NM_027371.2 | brix domain containing 5 (Bxdc5), transcript variant 2 | 0.57 |
| *Bzw1* | NM_025824 | | 0.42 |
| *Bzw2* | NM_025840.2 | basic leucine zipper and W2 domains 2 (Bzw2) | 0.38 |
| *C030003D03Rik* | NM_029881.2 | RIKEN cDNA C030003D03 gene (C030003D03Rik) | 0.38 |
| *C130007D14* | NM_177914.1 | | 0.59 |
| *C130020C13Rik* | | | 0.63 |
| *C130032J12Rik* | NM_178684.3 | RIKEN cDNA C130032J12 gene (C130032J12Rik) | 0.56 |
| *C1galt1c1* | NM_021550.2 | C1GALT1-specific chaperone 1 (C1galt1c1) | 0.61 |
| *C230043G09Rik* | AK048752 | | 0.60 |
| *C230053B09Rik* | AK048765 | | 0.60 |
| *C230071H18Rik* | NM_207265.2 | RIKEN cDNA C230071H18 gene (C230071H18Rik) | 0.60 |
| *C230078M08Rik* | NM_176995.2 | RIKEN cDNA C230078M08 gene (C230078M08Rik) | 0.62 |
| *C330006P03Rik* | | | 0.41 |
| *C330023M02Rik* | NM_172722 | | 0.58 |
| *C530008M17Rik* | XM_916109.2 | PREDICTED: RIKEN cDNA C530008M17 gene, transcript variant 3 (C530008M17Rik) | 0.25 |
| *C730026E21Rik* | NM_175439 | | 0.66 |
| *C730029F17Rik* | AK050233 | | 0.59 |
| *C78339* | XM_127312 | | 0.53 |
| *C79267* | NM_183148 | | 0.56 |
| *C79407* | NM_172578.2 | expressed sequence C79407 (C79407) | 0.46 |
| *C80913* | NM_011274.4 | expressed sequence C80913 (C80913) | 0.62 |
| *C920004C08Rik* | | | 0.21 |
| *Cacna1h* | NM_021415.3 | calcium channel, voltage-dependent, T type, alpha 1H subunit (Cacna1h) | 0.39 |
| *Cacna2d1* | NM_009784.1 | calcium channel, voltage-dependent, alpha2/delta subunit 1 (Cacna2d1) | 0.66 |
| *Cacng5* | NM_080644.3 | calcium channel, voltage-dependent, gamma subunit 5 (Cacng5) | 0.57 |
| *Calm2* | NM_007589.4 | calmodulin 2 (Calm2) | 0.29 |
| *Calr* | NM_007591.3 | calreticulin (Calr) | 0.53 |
| *Calu* | NM_007594.3 | calumenin (Calu), transcript variant 1 | 0.34 |
| *Camk1* | NM_133926.2 | calcium/calmodulin-dependent protein kinase I (Camk1) | 0.64 |
| *Camk2b* | NM_007595.3 | calcium/calmodulin-dependent protein kinase II, beta (Camk2b) | 0.16 |
| *Camk2n2* | XM_990809.1 | PREDICTED: calcium/calmodulin-dependent protein kinase II inhibitor 2 (Camk2n2) | 0.55 |
| *Camkk2* | NM_145358.1 | calcium/calmodulin-dependent protein kinase kinase 2, beta (Camkk2) | 0.52 |
| *Camta1* | NM_001081557.1 | calmodulin binding transcription activator 1 (Camta1), transcript variant 1 | 0.61 |
| *Cand1* | XM_125901.8 | PREDICTED: cullin associated and neddylation disassociated 1, transcript variant 1 (Cand1) | 0.65 |
| *Caprin1* | NM_016739.2 | cell cycle associated protein 1 (Caprin1) | 0.64 |
| *Car2* | NM_009801.3 | carbonic anhydrase 2 (Car2) | 0.39 |
| *Cask* | NM_009806.2 | calcium/calmodulin-dependent serine protein kinase (MAGUK family) (Cask) | 0.35 |
| *Cbfb* | NM_022309.2 | core binding factor beta (Cbfb) | 0.21 |
| *Cbr3* | NM_173047.2 | carbonyl reductase 3 (Cbr3) | 0.61 |
| *Cbx3* | NM_007624 | | 0.45 |
| *Cbx5* | NM_001076789.1 | chromobox homolog 5 (Drosophila HP1a) (Cbx5), transcript variant 2 | 0.59 |
| *Ccdc124* | NM_026964.3 | coiled-coil domain containing 124 (Ccdc124) | 0.64 |
| *Ccdc28b* | NM_025455.2 | coiled coil domain containing 28B (Ccdc28b) | 0.62 |

| | | | |
|---|---|---|---|
| *Ccdc41* | NM_029852.2 | coiled-coil domain containing 41 (Ccdc41) | 0.65 |
| *Ccdc58* | NM_198645.1 | coiled-coil domain containing 58 (Ccdc58) | 0.64 |
| *Ccdc59* | NM_025602.3 | coiled-coil domain containing 59 (Ccdc59) | 0.64 |
| *Ccdc85b* | NM_198616.2 | coiled-coil domain containing 85B (Ccdc85b) | 0.59 |
| *Ccdc86* | NM_023731.2 | coiled-coil domain containing 86 (Ccdc86) | 0.61 |
| *Ccdc99* | NM_027411.1 | coiled-coil domain containing 99 (Ccdc99) | 0.57 |
| *Ccnb1* | NM_172301.3 | cyclin B1 (Ccnb1) | 0.12 |
| *Ccnd1* | NM_007631.1 | | 0.04 |
| *Ccnd2* | AK077367 | | 0.43 |
| *Ccne2* | NM_001037134.1 | cyclin E2 (Ccne2), transcript variant 1 | 0.65 |
| *Ccnyl1* | NM_001097644.1 | cyclin Y-like 1 (Ccnyl1) | 0.38 |
| *Cct3* | NM_009836.1 | chaperonin containing Tcp1, subunit 3 (gamma) (Cct3) | 0.51 |
| *Cct4* | NM_009837.1 | chaperonin containing Tcp1, subunit 4 (delta) (Cct4) | 0.56 |
| *Cct6a* | NM_009838.1 | chaperonin containing Tcp1, subunit 6a (zeta) (Cct6a) | 0.48 |
| *Cct8* | NM_009840.3 | chaperonin containing Tcp1, subunit 8 (theta) (Cct8) | 0.52 |
| *Cd1d1* | NM_007639.2 | CD1d1 antigen (Cd1d1) | 0.49 |
| *Cd276* | NM_133983.3 | CD276 antigen (Cd276) | 0.27 |
| *Cdc20* | NM_023223.1 | cell division cycle 20 homolog (S. cerevisiae) (Cdc20) | 0.19 |
| *Cdc25a* | NM_007658.3 | cell division cycle 25 homolog A (S. pombe) (Cdc25a) | 0.58 |
| *Cdc25c* | NM_009860.2 | cell division cycle 25 homolog C (S. pombe) (Cdc25c) | 0.61 |
| *Cdc2a* | NM_007659.3 | cell division cycle 2 homolog A (S. pombe) (Cdc2a) | 0.15 |
| *Cdc2l1* | NM_007661.3 | cell division cycle 2-like 1 (Cdc2l1) | 0.65 |
| *Cdc42se1* | NM_172395.2 | CDC42 small effector 1 (Cdc42se1), transcript variant 1 | 0.48 |
| *Cdc45l* | NM_009862.1 | cell division cycle 45 homolog (S. cerevisiae)-like (Cdc45l) | 0.47 |
| *Cdc5l* | NM_152810.1 | cell division cycle 5-like (S. pombe) (Cdc5l) | 0.58 |
| *Cdc6* | NM_011799.2 | cell division cycle 6 homolog (S. cerevisiae) (Cdc6), transcript variant 1 | 0.55 |
| *Cdc7* | NM_009863.2 | cell division cycle 7 (S. cerevisiae) (Cdc7) | 0.30 |
| *Cdca2* | NM_175384.3 | cell division cycle associated 2 (Cdca2) | 0.25 |
| *Cdca3* | NM_013538.4 | cell division cycle associated 3 (Cdca3) | 0.12 |
| *Cdca4* | NM_028023.3 | cell division cycle associated 4 (Cdca4) | 0.44 |
| *Cdca5* | NM_026410.1 | cell division cycle associated 5 (Cdca5) | 0.57 |
| *Cdca7* | NM_025866.3 | cell division cycle associated 7 (Cdca7) | 0.17 |
| *Cdca8* | NM_026560.3 | cell division cycle associated 8 (Cdca8) | 0.14 |
| *Cdh13* | NM_019707.4 | cadherin 13 (Cdh13) | 0.20 |
| *Cdh4* | NM_009867.1 | cadherin 4 (Cdh4) | 0.63 |
| *Cdk2* | NM_183417.2 | cyclin-dependent kinase 2 (Cdk2), transcript variant 1 | 0.31 |
| *Cdk4* | NM_009870.2 | cyclin-dependent kinase 4 (Cdk4) | 0.43 |
| *Cdk5r1* | NM_009871.2 | cyclin-dependent kinase 5, regulatory subunit 1 (p35) (Cdk5r1) | 0.54 |
| *Cdk8* | NM_153599.3 | cyclin-dependent kinase 8 (Cdk8) | 0.59 |
| *Cdkn2aipnl* | NM_029976.2 | CDKN2A interacting protein N-terminal like (Cdkn2aipnl) | 0.50 |
| *Cdkn3* | XM_919022.2 | PREDICTED:  cyclin-dependent kinase inhibitor 3, transcript variant 5 (Cdkn3) | 0.25 |
| *Cdon* | AK040711 | | 0.53 |
| *Cdr2* | NM_007672.1 | cerebellar degeneration-related 2 (Cdr2) | 0.44 |
| *Cdr2l* | NM_001080929.1 | cerebellar degeneration-related protein 2-like (Cdr2l) | 0.42 |
| *Cdt1* | NM_026014.3 | chromatin licensing and DNA replication factor 1 (Cdt1) | 0.29 |
| *Cenpa* | NM_007681.2 | centromere protein A (Cenpa) | 0.06 |
| *Cenpf* | XM_916605.2 | PREDICTED:  centromere autoantigen F, transcript variant 3 (Cenpf) | 0.23 |
| *Cenph* | NM_021886.1 | centromere protein H (Cenph) | 0.65 |
| *Cenpi* | NM_145924.2 | centromere protein I (Cenpi) | 0.57 |
| *Cenpk* | NM_021790.1 | centromere protein K (Cenpk), transcript variant 1 | 0.42 |
| *Cenpl* | NM_027429.1 | centromere protein L (Cenpl) | 0.41 |
| *Cenpm* | NM_025639.4 | centromere protein M (Cenpm), transcript variant 1 | 0.63 |
| *Cenpn* | NM_028131.3 | centromere protein N (Cenpn) | 0.55 |
| *Cenpp* | NM_025495.1 | centromere protein P (Cenpp) | 0.59 |
| *Cenpq* | NM_031863.3 | centromere protein Q (Cenpq) | 0.55 |
| *Cenpt* | NM_177150.2 | centromere protein T (Cenpt) | 0.63 |
| *Centd1* | XM_001001363.1 | PREDICTED:  centaurin, delta 1, transcript variant 5 (Centd1) | 0.60 |
| *Cep170* | NM_001099637.1 | centrosomal protein 170 (Cep170) | 0.48 |
| *Cep55* | NM_028760.1 | centrosomal protein 55 (Cep55) | 0.60 |
| *Cep78* | NM_198019.2 | centrosomal protein 78 (Cep78) | 0.52 |
| *Cetn3* | NM_007684.3 | centrin 3 (Cetn3) | 0.62 |
| *Cfl1* | NM_007687.2 | cofilin 1, non-muscle (Cfl1) | 0.41 |
| *Chaf1a* | NM_013733.3 | chromatin assembly factor 1, subunit A (p150) (Chaf1a) | 0.60 |
| *Chaf1b* | NM_028083.3 | chromatin assembly factor 1, subunit B (p60) (Chaf1b) | 0.31 |
| *Chchd1* | NM_025366.2 | coiled-coil-helix-coiled-coil-helix domain containing 1 (Chchd1) | 0.50 |
| *Chchd8* | NM_183270.2 | coiled-coil-helix-coiled-coil-helix domain containing 8 (Chchd8) | 0.64 |
| *Chd1* | AK021188 | | 0.58 |
| *Chd4* | NM_145979.2 | chromodomain helicase DNA binding protein 4 (Chd4) | 0.63 |
| *Chfr* | NM_172717.1 | checkpoint with forkhead and ring finger domains (Chfr) | 0.47 |
| *Chmp4b* | NM_029362.3 | chromatin modifying protein 4B (Chmp4b) | 0.59 |

| | | | |
|---|---|---|---|
| *Chn2* | NM_023543.1 | chimerin (chimaerin) 2 (Chn2) | 0.23 |
| *Chrm3* | NM_033269.2 | cholinergic receptor, muscarinic 3, cardiac (Chrm3) | 0.55 |
| *Chst2* | NM_018763.1 | carbohydrate sulfotransferase 2 (Chst2) | 0.35 |
| *Chst7* | NM_021715.1 | carbohydrate (N-acetylglucosamino) sulfotransferase 7 (Chst7) | 0.12 |
| *Chtf18* | NM_145409.1 | CTF18, chromosome transmission fidelity factor 18 homolog (S. cerevisiae) (Chtf18) | 0.53 |
| *Cirh1a* | NM_011574.1 | cirrhosis, autosomal recessive 1A (human) (Cirh1a) | 0.65 |
| *Ckap2* | XM_134100.3 | | 0.39 |
| *Ckap2l* | NM_181589.2 | cytoskeleton associated protein 2-like (Ckap2l) | 0.56 |
| *Cldnd1* | NM_171826.2 | claudin domain containing 1 (Cldnd1) | 0.52 |
| *Clic4* | NM_013885.2 | chloride intracellular channel 4 (mitochondrial) (Clic4), nuclear gene encoding mitochondrial protein | 0.47 |
| *Clic5* | NM_172621.2 | chloride intracellular channel 5 (Clic5) | 0.66 |
| *Cln5* | XM_127882.3 | | 0.56 |
| *Clns1a* | NM_023671.1 | chloride channel, nucleotide-sensitive, 1A (Clns1a) | 0.55 |
| *Clspn* | NM_175554.3 | claspin homolog (Xenopus laevis) (Clspn) | 0.27 |
| *Clta* | NM_001080385.1 | clathrin, light polypeptide (Lca) (Clta), transcript variant 2 | 0.64 |
| *Cltc* | NM_001003908 | | 0.58 |
| *Cmas* | NM_009908.1 | cytidine monophospho-N-acetylneuraminic acid synthetase (Cmas) | 0.53 |
| *Cmc1* | NM_026442.2 | COX assembly mitochondrial protein homolog (S. cerevisiae) (Cmc1), nuclear gene encoding mitochondrial protein | 0.61 |
| *Cmpk* | NM_025647.2 | cytidylate kinase (Cmpk) | 0.54 |
| *Cnih* | NM_009919.1 | cornichon homolog (Drosophila) (Cnih) | 0.61 |
| *Cnih4* | NM_030131.2 | cornichon homolog 4 (Drosophila) (Cnih4) | 0.49 |
| *Cntnap4* | NM_130457 | | 0.43 |
| *Col16a1* | NM_028266.4 | collagen, type XVI, alpha 1 (Col16a1) | 0.23 |
| *Col18a1* | NM_009929.2 | procollagen, type XVIII, alpha 1 (Col18a1) | 0.08 |
| *Col4a1* | NM_009931.1 | procollagen, type IV, alpha 1 (Col4a1) | 0.62 |
| *Commd1* | NM_144514.2 | COMM domain containing 1 (Commd1) | 0.62 |
| *Commd10* | NM_178377.4 | COMM domain containing 10 (Commd10) | 0.64 |
| *Cops2* | NM_009939.2 | COP9 (constitutive photomorphogenic) homolog, subunit 2 (Arabidopsis thaliana) (Cops2) | 0.62 |
| *Cops7a* | NM_012003.1 | COP9 (constitutive photomorphogenic) homolog, subunit 7a (Arabidopsis thaliana) (Cops7a) | 0.62 |
| *Coq9* | NM_026452.2 | coenzyme Q9 homolog (yeast) (Coq9) | 0.64 |
| *Coro1c* | NM_011779.3 | coronin, actin binding protein 1C (Coro1c) | 0.48 |
| *Cox10* | NM_178379.2 | COX10 homolog, cytochrome c oxidase assembly protein, heme A: farnesyltransferase (yeast) (Cox10), nuclear gene encoding mitochondrial protein | 0.60 |
| *Cox15* | NM_144874.3 | COX15 homolog, cytochrome c oxidase assembly protein (yeast) (Cox15), nuclear gene encoding mitochondrial protein | 0.54 |
| *Cox17* | NM_001017429.2 | cytochrome c oxidase, subunit XVII assembly protein homolog (yeast) (Cox17) | 0.66 |
| *Cox18* | NM_001033310.1 | COX18 cytochrome c oxidase assembly homolog (S. cerevisiae) (Cox18) | 0.53 |
| *Cox5a* | NM_007747.2 | cytochrome c oxidase, subunit Va (Cox5a), nuclear gene encoding mitochondrial protein | 0.55 |
| *Cox6a1* | NM_007748.2 | | 0.64 |
| *Cox6c* | NM_053071.2 | cytochrome c oxidase, subunit VIc (Cox6c) | 0.65 |
| *Cox7a2* | NM_009945.3 | cytochrome c oxidase, subunit VIIa 2 (Cox7a2) | 0.51 |
| *Cox7a2l* | NM_009187.1 | cytochrome c oxidase subunit VIIa polypeptide 2-like (Cox7a2l) | 0.64 |
| *Cpeb4* | NM_026252 | | 0.60 |
| *Cplx1* | NM_007756.2 | complexin 1 (Cplx1) | 0.58 |
| *Cpne8* | NM_001033851.1 | copine VIII (Cpne8), transcript variant 2 | 0.56 |
| *Crispld2* | NM_030209.2 | cysteine-rich secretory protein LCCL domain containing 2 (Crispld2) | 0.66 |
| *Crlf3* | NM_018776.1 | cytokine receptor-like factor 3 (Crlf3) | 0.65 |
| *Crtap* | NM_019922.1 | cartilage associated protein (Crtap) | 0.53 |
| *Cs* | NM_026444.3 | citrate synthase (Cs), nuclear gene encoding mitochondrial protein | 0.42 |
| *Csad* | NM_144942.1 | cysteine sulfinic acid decarboxylase (Csad) | 0.57 |
| *Csda* | NM_139117.2 | cold shock domain protein A (Csda) | 0.55 |
| *Cse1l* | NM_023565.2 | chromosome segregation 1-like (S. cerevisiae) (Cse1l) | 0.50 |
| *Csnk1d* | NM_027874.2 | casein kinase 1, delta (Csnk1d), transcript variant 2 | 0.48 |
| *Csnk1e* | NM_013767.5 | casein kinase 1, epsilon (Csnk1e) | 0.60 |
| *Csnk1g3* | NM_152809.2 | casein kinase 1, gamma 3 (Csnk1g3) | 0.65 |
| *Csnk2a1-rs3* | AK031617 | | 0.59 |
| *Csnk2a2* | NM_009974.2 | casein kinase 2, alpha prime polypeptide (Csnk2a2) | 0.59 |
| *Cspg5* | NM_013884.2 | chondroitin sulfate proteoglycan 5 (Cspg5) | 0.36 |
| *Csrp2* | NM_007792.3 | cysteine and glycine-rich protein 2 (Csrp2) | 0.47 |
| *Cstf1* | NM_024199.2 | cleavage stimulation factor, 3' pre-RNA, subunit 1 (Cstf1) | 0.65 |
| *Cstf2* | NM_133196.5 | cleavage stimulation factor, 3' pre-RNA subunit 2 (Cstf2) | 0.59 |
| *Cstf3* | NM_145529.2 | cleavage stimulation factor, 3' pre-RNA, subunit 3 (Cstf3), transcript variant 1 | 0.66 |

| | | | |
|---|---|---|---|
| *Ctdspl2* | NM_212450.3 | CTD (carboxy-terminal domain, RNA polymerase II, polypeptide A) small phosphatase like 2 (Ctdspl2) | 0.65 |
| *Ctgf* | NM_010217 | | 0.63 |
| *Ctps* | NM_016748.1 | cytidine 5'-triphosphate synthase (Ctps) | 0.66 |
| *Ctsc* | NM_009982.3 | cathepsin C (Ctsc) | 0.34 |
| *Cttnbp2nl* | NM_030249.3 | CTTNBP2 N-terminal like (Cttnbp2nl) | 0.40 |
| *Cugbp2* | NM_010160.2 | CUG triplet repeat, RNA binding protein 2 (Cugbp2), transcript variant 6 | 0.45 |
| *Cul3* | NM_016716.4 | cullin 3 (Cul3) | 0.53 |
| *Cx3cl1* | NM_009142.3 | chemokine (C-X3-C motif) ligand 1 (Cx3cl1) | 0.46 |
| *Cxadr* | NM_009988 | | 0.44 |
| *Cxcl4* | NM_019932.2 | chemokine (C-X-C motif) ligand 4 (Cxcl4) | 0.46 |
| *Cyb5r4* | NM_024195.1 | cytochrome b5 reductase 4 (Cyb5r4) | 0.62 |
| *Cyc1* | NM_025567.2 | cytochrome c-1 (Cyc1) | 0.40 |
| *Cycs* | NM_007808.4 | cytochrome c, somatic (Cycs), nuclear gene encoding mitochondrial protein | 0.37 |
| *Cyp51* | NM_020010.2 | cytochrome P450, family 51 (Cyp51) | 0.30 |
| *D030038A19Rik* | AK083512 | | 0.55 |
| *D030056L22Rik* | NM_177640.4 | RIKEN cDNA D030056L22 gene (D030056L22Rik) | 0.53 |
| *D030063E12* | | | 0.62 |
| *D10Ertd322e* | NM_026065.2 | DNA segment, Chr 10, ERATO Doi 322, expressed (D10Ertd322e) | 0.57 |
| *D10Wsu102e* | NM_026579.2 | DNA segment, Chr 10, Wayne State University 102, expressed (D10Wsu102e) | 0.59 |
| *D10Wsu52e* | NM_145422.3 | DNA segment, Chr 10, Wayne State University 52, expressed (D10Wsu52e) | 0.61 |
| *D12Ertd553e* | NM_029758.3 | DNA segment, Chr 12, ERATO Doi 553, expressed (D12Ertd553e) | 0.59 |
| *D12Wsu95e* | NM_198023 | | 0.45 |
| *D130004H04Rik* | | | 0.60 |
| *D17H6S56E-5* | NM_033075.2 | DNA segment, Chr 17, human D6S56E 5 (D17H6S56E-5) | 0.54 |
| *D17Wsu104e* | NM_080837.1 | | 0.55 |
| *D19Bwg1357e* | NM_177474.4 | DNA segment, Chr 19, Brigham & Women's Genetics 1357 expressed (D19Bwg1357e) | 0.51 |
| *D1Ertd471e* | | | 0.33 |
| *D2Bwg1335e* | NM_026828.2 | DNA segment, Chr 2, Brigham & Women's Genetics 1335 expressed (D2Bwg1335e) | 0.51 |
| *D2Ertd750e* | NM_026412.1 | DNA segment, Chr 2, ERATO Doi 750, expressed (D2Ertd750e) | 0.37 |
| *D4Bwg0951e* | NM_026821.4 | DNA segment, Chr 4, Brigham & Women's Genetics 0951 expressed (D4Bwg0951e) | 0.47 |
| *D6Wsu176e* | NM_138587.4 | DNA segment, Chr 6, Wayne State University 176, expressed (D6Wsu176e) | 0.53 |
| *D930028F11Rik* | NM_172921.2 | RIKEN cDNA D930028F11 gene (D930028F11Rik) | 0.56 |
| *D930046M13Rik* | | | 0.28 |
| *D9Wsu20e* | NM_133718 | | 0.43 |
| *Dab2* | NM_023118.1 | | 0.60 |
| *Dazap1* | NM_133188.1 | DAZ associated protein 1 (Dazap1) | 0.53 |
| *Dbf4* | NM_013726.2 | DBF4 homolog (S. cerevisiae) (Dbf4) | 0.25 |
| *Dbt* | NM_010022.1 | dihydrolipoamide branched chain transacylase E2 (Dbt) | 0.64 |
| *Dbx2* | XM_147966.3 | | 0.48 |
| *Dcbld1* | NM_025705.2 | discoidin, CUB and LCCL domain containing 1 (Dcbld1) | 0.60 |
| *Dclk1* | NM_019978.2 | doublecortin-like kinase 1 (Dclk1) | 0.23 |
| *Dclk2* | NM_027539.3 | doublecortin-like kinase 2 (Dclk2) | 0.61 |
| *Dcun1d5* | NM_029775.2 | DCN1, defective in cullin neddylation 1, domain containing 5 (S. cerevisiae) (Dcun1d5) | 0.60 |
| *Ddah1* | NM_026993 | | 0.48 |
| *Ddost* | NM_007838.2 | dolichyl-di-phosphooligosaccharide-protein glycotransferase (Ddost) | 0.58 |
| *Ddx1* | NM_134040.1 | DEAD (Asp-Glu-Ala-Asp) box polypeptide 1 (Ddx1) | 0.63 |
| *Ddx11* | XM_128714.3 | | 0.36 |
| *Ddx20* | NM_017397.2 | DEAD (Asp-Glu-Ala-Asp) box polypeptide 20 (Ddx20) | 0.60 |
| *Ddx21* | NM_019553.2 | DEAD (Asp-Glu-Ala-Asp) box polypeptide 21 (Ddx21) | 0.50 |
| *Ddx25* | NM_013932.3 | DEAD (Asp-Glu-Ala-Asp) box polypeptide 25 (Ddx25) | 0.56 |
| *Ddx26* | NM_008715 | | 0.56 |
| *Ddx27* | NM_153065.2 | DEAD (Asp-Glu-Ala-Asp) box polypeptide 27 (Ddx27) | 0.67 |
| *Ddx3x* | NM_010028.2 | DEAD/H (Asp-Glu-Ala-Asp/His) box polypeptide 3, X-linked (Ddx3x) | 0.59 |
| *Ddx54* | NM_028041.2 | DEAD (Asp-Glu-Ala-Asp) box polypeptide 54 (Ddx54) | 0.54 |
| *Ddx6* | NM_007841.3 | DEAD (Asp-Glu-Ala-Asp) box polypeptide 6 (Ddx6) | 0.51 |
| *Deb1* | NM_026794.2 | differentially expressed in B16F10 1 (Deb1) | 0.53 |
| *Dek* | NM_025900.1 | DEK oncogene (DNA binding) (Dek) | 0.60 |
| *Dennd2a* | NM_172477.3 | DENN/MADD domain containing 2A (Dennd2a) | 0.33 |
| *Dgkb* | NM_178681 | | 0.55 |
| *Dgkd* | | | 0.52 |
| *Dgkk* | NM_177914.2 | diacylglycerol kinase kappa (Dgkk) | 0.43 |
| *Dgkz* | NM_138306.1 | diacylglycerol kinase zeta (Dgkz) | 0.43 |
| *Dhcr24* | XM_131538.1 | | 0.45 |
| *Dhcr7* | NM_007856.2 | 7-dehydrocholesterol reductase (Dhcr7) | 0.40 |
| *Dhh* | NM_007857.3 | desert hedgehog (Dhh) | 0.41 |

| | | | |
|---|---|---|---|
| *Dhrsx* | NM_001033326.2 | dehydrogenase/reductase (SDR family) X chromosome (Dhrsx) | 0.58 |
| *Dhx15* | NM_007839.2 | DEAH (Asp-Glu-Ala-His) box polypeptide 15 (Dhx15), transcript variant 2 | 0.57 |
| *Diras2* | NM_001024474.2 | DIRAS family, GTP-binding RAS-like 2 (Diras2) | 0.40 |
| *Dlg5* | XM_981375.2 | PREDICTED: discs, large homolog 5 (Drosophila) (Dlg5) | 0.66 |
| *Dlgap5* | NM_144553.1 | discs, large (Drosophila) homolog-associated protein 5 (Dlgap5) | 0.47 |
| *Dlst* | NM_030225 | | 0.53 |
| *Dnahc11* | NM_010060.2 | dynein, axonemal, heavy chain 11 (Dnahc11) | 0.30 |
| *Dnajb11* | XM_148071.1 | | 0.51 |
| *Dnajc2* | NM_009584.3 | DnaJ (Hsp40) homolog, subfamily C, member 2 (Dnajc2) | 0.48 |
| *Dnclc1* | NM_019682 | | 0.58 |
| *Dnmt1* | NM_010066.3 | DNA methyltransferase (cytosine-5) 1 (Dnmt1) | 0.36 |
| *Dock1* | NM_001033420.2 | dedicator of cytokinesis 1 (Dock1) | 0.62 |
| *Dock10* | XM_129913.4 | | 0.36 |
| *Dos* | NM_015761.2 | downstream of Stk11 (Dos) | 0.60 |
| *Dph2* | NM_026344.3 | DPH2 homolog (S. cerevisiae) (Dph2) | 0.63 |
| *Dpp3* | NM_133803.1 | dipeptidylpeptidase 3 (Dpp3) | 0.66 |
| *Dpy30* | NM_024428.3 | dpy-30 homolog (C. elegans) (Dpy30) | 0.61 |
| *Dpysl3* | NM_009468.3 | dihydropyrimidinase-like 3 (Dpysl3) | 0.45 |
| *Drctnnb1a* | NM_053090 | | 0.48 |
| *Drg1* | NM_007879.1 | developmentally regulated GTP binding protein 1 (Drg1) | 0.65 |
| *Dtymk* | NM_023136 | | 0.60 |
| *Dus4l* | NM_028002.2 | dihydrouridine synthase 4-like (S. cerevisiae) (Dus4l) | 0.66 |
| *Dusp11* | NM_028099.2 | dual specificity phosphatase 11 (RNA/RNP complex 1-interacting) (Dusp11) | 0.63 |
| *Dusp4* | NM_176933.4 | dual specificity phosphatase 4 (Dusp4) | 0.34 |
| *Dusp6* | NM_026268.2 | dual specificity phosphatase 6 (Dusp6) | 0.10 |
| *Dusp7* | NM_153459.2 | dual specificity phosphatase 7 (Dusp7) | 0.23 |
| *Dynll1* | NM_019682.2 | dynein light chain LC8-type 1 (Dynll1) | 0.54 |
| *E030026I10Rik* | NM_175553 | | 0.61 |
| *E130016E03Rik* | NM_001039556.2 | RIKEN cDNA E130016E03 gene (E130016E03Rik) | 0.31 |
| *E130306D19Rik* | XM_131404.3 | | 0.47 |
| *E230013M07Rik* | AK054036 | | 0.25 |
| *E2f1* | NM_007891.3 | E2F transcription factor 1 (E2f1) | 0.33 |
| *E2f2* | NM_177733.2 | E2F transcription factor 2 (E2f2) | 0.54 |
| *E330016A19Rik* | NM_173386.3 | RIKEN cDNA E330016A19 gene (E330016A19Rik) | 0.41 |
| *E430028B21Rik* | NM_178668.3 | RIKEN cDNA E430028B21 gene (E430028B21Rik) | 0.60 |
| *E430039I23Rik* | AK089103 | | 0.58 |
| *E530011J04Rik* | AK089133 | | 0.55 |
| *Ebna1bp2* | NM_026932.3 | EBNA1 binding protein 2 (Ebna1bp2) | 0.48 |
| *Ece1* | NM_199307.1 | endothelin converting enzyme 1 (Ece1) | 0.44 |
| *Ecm1* | NM_007899.1 | extracellular matrix protein 1 (Ecm1) | 0.58 |
| *Eed* | NM_021876.2 | embryonic ectoderm development (Eed) | 0.65 |
| *Eef1a1* | NM_010106.2 | eukaryotic translation elongation factor 1 alpha 1 (Eef1a1) | 0.60 |
| *Eef1b2* | NM_018796.2 | eukaryotic translation elongation factor 1 beta 2 (Eef1b2) | 0.48 |
| *Eef1d* | NM_023240.2 | eukaryotic translation elongation factor 1 delta (guanine nucleotide exchange protein) (Eef1d), transcript variant 2 | 0.66 |
| *Eef1e1* | NM_025380.2 | eukaryotic translation elongation factor 1 epsilon 1 (Eef1e1) | 0.34 |
| *Eef1g* | NM_026007.4 | eukaryotic translation elongation factor 1 gamma (Eef1g) | 0.54 |
| *Eef2* | NM_007907.1 | eukaryotic translation elongation factor 2 (Eef2) | 0.58 |
| *Efnb1* | NM_010110.2 | ephrin B1 (Efnb1) | 0.64 |
| *Efs* | NM_010112.3 | embryonal Fyn-associated substrate (Efs) | 0.52 |
| *EG216185* | XM_911906.3 | PREDICTED: predicted gene, EG216185 (EG216185) | 0.58 |
| *EG237361* | XM_905111.2 | PREDICTED: predicted gene, EG237361 (EG237361) | 0.44 |
| *EG243642* | XM_145024.8 | PREDICTED: predicted gene, EG243642 (EG243642) | 0.41 |
| *EG245297* | NM_001018086.2 | predicted gene, EG245297 (EG245297) | 0.65 |
| *EG268795* | XM_921134.3 | PREDICTED: predicted gene, EG268795, transcript variant 4 (EG268795) | 0.44 |
| *EG382843* | XM_905000.3 | PREDICTED: predicted gene, EG382843 (EG382843) | 0.66 |
| *EG432681* | XM_484165.5 | PREDICTED: predicted gene, EG432681 (EG432681) | 0.51 |
| *EG432721* | XM_484210.5 | PREDICTED: predicted gene, EG432721 (EG432721) | 0.44 |
| *EG432879* | NM_001034882.2 | predicted gene, EG432879 (EG432879) | 0.55 |
| *EG433144* | XM_484670.5 | PREDICTED: predicted gene, EG433144 (EG433144) | 0.66 |
| *EG433182* | NM_001025388.1 | predicted gene, EG433182 (EG433182) | 0.67 |
| *EG433923* | NM_001081233.1 | predicted gene, EG433923 (EG433923) | 0.45 |
| *EG434404* | XM_486222.5 | PREDICTED: predicted gene, EG434404 (EG434404) | 0.58 |
| *EG434858* | NR_002883.1 | predicted gene, EG434858 (EG434858), non-coding RNA. | 0.67 |
| *EG545056* | NR_002889.2 | predicted gene, EG545056 (EG545056), non-coding RNA. | 0.62 |
| *EG622236* | XM_917756.3 | PREDICTED: predicted gene, EG622236, transcript variant 1 (EG622236) | 0.50 |
| *EG622339* | NM_001081297.1 | predicted gene, EG622339 (EG622339) | 0.64 |
| *EG623818* | XM_888318.3 | PREDICTED: predicted gene, EG623818 (EG623818) | 0.50 |
| *EG624124* | XM_889835.2 | PREDICTED: predicted gene, EG624124, transcript variant 1 (EG624124) | 0.55 |
| *EG625917* | XM_917009.3 | PREDICTED: predicted gene, EG625917 (EG625917) | 0.56 |
| *EG626175* | XM_908326.3 | PREDICTED: predicted gene, EG626175 (EG626175) | 0.41 |

| | | | |
|---|---|---|---|
| *EG629595* | XM_894495.2 | PREDICTED: predicted gene, EG629595 (EG629595) | 0.51 |
| *EG632248* | XM_001473902.1 | PREDICTED: predicted gene, EG632248 (EG632248) | 0.54 |
| *EG633120* | XR_031485.1 | PREDICTED: predicted gene, EG633120 (EG633120), misc RNA. | 0.56 |
| *EG637748* | XM_917238.2 | PREDICTED: predicted gene, EG637748 (EG637748) | 0.59 |
| *EG665369* | XM_912043.2 | PREDICTED: predicted gene, EG665369 (EG665369) | 0.51 |
| *EG666577* | XM_984280.2 | PREDICTED: predicted gene, EG666577 (EG666577) | 0.46 |
| *EG666609* | XM_984926.2 | PREDICTED: predicted gene, EG666609 (EG666609) | 0.66 |
| *EG667190* | XR_002058.1 | PREDICTED: predicted gene, EG667190 (EG667190), misc RNA. | 0.38 |
| *EG667728* | XR_035278.1 | PREDICTED: predicted gene, EG667728 (EG667728), misc RNA. | 0.31 |
| *EG668668* | NM_001081036.1 | predicted gene, EG668668 (EG668668) | 0.57 |
| *EG668829* | XM_001005405.1 | PREDICTED: predicted gene, EG668829 (EG668829) | 0.48 |
| *EG668850* | XR_002259.2 | PREDICTED: predicted gene, EG668850 (EG668850), misc RNA. | 0.60 |
| *Egfr* | NM_007912.4 | epidermal growth factor receptor (Egfr), transcript variant 2 | 0.64 |
| *Egr3* | NM_018781 | | 0.54 |
| *Ehd4* | NM_133838.4 | EH-domain containing 4 (Ehd4) | 0.36 |
| *Eif1a* | NM_010120.4 | eukaryotic translation initiation factor 1A (Eif1a) | 0.63 |
| *Eif1ad* | NM_027236.2 | eukaryotic translation initiation factor 1A domain containing (Eif1ad) | 0.49 |
| *Eif2b1* | NM_145371.3 | eukaryotic translation initiation factor 2B, subunit 1 (alpha) (Eif2b1) | 0.66 |
| *Eif2b3* | XM_131572.4 | | 0.62 |
| *Eif2s2* | NM_026030.2 | eukaryotic translation initiation factor 2, subunit 2 (beta) (Eif2s2) | 0.46 |
| *Eif2s3x* | NM_012010.3 | eukaryotic translation initiation factor 2, subunit 3, structural gene X-linked (Eif2s3x) | 0.52 |
| *Eif3a* | NM_010123.3 | eukaryotic translation initiation factor 3, subunit A (Eif3a) | 0.57 |
| *Eif3b* | NM_133916.2 | eukaryotic translation initiation factor 3, subunit B (Eif3b) | 0.66 |
| *Eif3d* | NM_018749.2 | eukaryotic translation initiation factor 3, subunit D (Eif3d) | 0.58 |
| *Eif3eip* | NM_145139.2 | eukaryotic translation initiation factor 3, subunit E interacting protein (Eif3eip) | 0.60 |
| *Eif3g* | NM_016876.3 | eukaryotic translation initiation factor 3, subunit G (Eif3g) | 0.51 |
| *Eif3i* | NM_018799.1 | eukaryotic translation initiation factor 3, subunit I (Eif3i) | 0.57 |
| *Eif3s1* | NM_144545.2 | eukaryotic translation initiation factor 3, subunit 1 alpha (Eif3s1) | 0.66 |
| *Eif3s4* | NM_016876.2 | | 0.48 |
| *Eif4a1* | NM_144958.2 | eukaryotic translation initiation factor 4A1 (Eif4a1) | 0.32 |
| *Eif4a3* | NM_138669.1 | eukaryotic translation initiation factor 4A, isoform 3 (Eif4a3) | 0.64 |
| *Eif4ebp1* | NM_007918.3 | eukaryotic translation initiation factor 4E binding protein 1 (Eif4ebp1) | 0.53 |
| *Eif4el3* | NM_023314.2 | | 0.53 |
| *Eif4g1* | NM_001005331.1 | eukaryotic translation initiation factor 4, gamma 1 (Eif4g1), transcript variant 2 | 0.61 |
| *Eif4g2* | NM_013507.2 | | 0.66 |
| *Eif4h* | NM_033561.1 | eukaryotic translation initiation factor 4H (Eif4h) | 0.62 |
| *Eif5* | NM_173363.4 | eukaryotic translation initiation factor 5 (Eif5), transcript variant 1 | 0.41 |
| *Eif5a* | | | 0.35 |
| *Elavl1* | NM_010485.3 | ELAV (embryonic lethal, abnormal vision, Drosophila)-like 1 (Hu antigen R) (Elavl1) | 0.65 |
| *Elk3* | NM_205536.1 | ELK3, member of ETS oncogene family (Elk3), transcript variant 2 | 0.35 |
| *Elmo1* | NM_198093.2 | engulfment and cell motility 1, ced-12 homolog (C. elegans) (Elmo1), transcript variant 2 | 0.35 |
| *Elmo2* | NM_080287.2 | engulfment and cell motility 2, ced-12 homolog (C. elegans) (Elmo2), transcript variant 3 | 0.63 |
| *Elovl1* | NM_019422.2 | elongation of very long chain fatty acids (FEN1/Elo2, SUR4/Elo3, yeast)-like 1 (Elovl1), transcript variant 2 | 0.60 |
| *Elovl4* | NM_148941.1 | elongation of very long chain fatty acids (FEN1/Elo2, SUR4/Elo3, yeast)-like 4 (Elovl4) | 0.51 |
| *Elovl5* | NM_134255.2 | ELOVL family member 5, elongation of long chain fatty acids (yeast) (Elovl5) | 0.52 |
| *Elovl6* | NM_130450.2 | ELOVL family member 6, elongation of long chain fatty acids (yeast) (Elovl6) | 0.21 |
| *Emg1* | NM_013536.1 | EMG1 nucleolar protein homolog (S. cerevisiae) (Emg1) | 0.61 |
| *Eno1* | NM_023119.1 | enolase 1, alpha non-neuron (Eno1) | 0.46 |
| *Entpd4* | NM_026174.2 | ectonucleoside triphosphate diphosphohydrolase 4 (Entpd4) | 0.52 |
| *Epb4.1l1* | XM_487678 | | 0.65 |
| *Epn2* | NM_010148.2 | epsin 2 (Epn2) | 0.44 |
| *Eprs* | NM_029735.1 | glutamyl-prolyl-tRNA synthetase (Eprs) | 0.53 |
| *Ercc1* | NM_007948.1 | excision repair cross-complementing rodent repair deficiency, complementation group 1 (Ercc1) | 0.62 |
| *Ergic1* | NM_026170.3 | endoplasmic reticulum-golgi intermediate compartment (ERGIC) 1 (Ergic1) | 0.36 |
| *Ergic2* | NM_026168.3 | ERGIC and golgi 2 (Ergic2), transcript variant 1 | 0.52 |
| *Erh* | NM_007951.1 | enhancer of rudimentary homolog (Drosophila) (Erh) | 0.50 |
| *Erlin1* | NM_145502.2 | ER lipid raft associated 1 (Erlin1) | 0.58 |
| *Ermp1* | NM_001081213.1 | endoplasmic reticulum metallopeptidase 1 (Ermp1) | 0.26 |
| *Erp29* | NM_026129.2 | endoplasmic reticulum protein 29 (Erp29) | 0.64 |
| *Errfi1* | NM_133753.1 | ERBB receptor feedback inhibitor 1 (Errfi1) | 0.64 |
| *Esco2* | NM_028039.1 | establishment of cohesion 1 homolog 2 (S. cerevisiae) (Esco2) | 0.32 |
| *Espl1* | NM_001014976.1 | extra spindle poles-like 1 (S. cerevisiae) (Espl1) | 0.64 |
| *Esrra* | NM_007953.2 | estrogen related receptor, alpha (Esrra) | 0.66 |

| | | | |
|---|---|---|---|
| *Esrrb* | NM_011934.3 | estrogen related receptor, beta (Esrrb) | 0.52 |
| *Etaa1* | NM_026576.2 | Ewing's tumor-associated antigen 1 (Etaa1) | 0.61 |
| *Etf1* | NM_144866.3 | eukaryotic translation termination factor 1 (Etf1) | 0.56 |
| *Ets1* | NM_001038642.1 | E26 avian leukemia oncogene 1, 5' domain (Ets1), transcript variant 2 | 0.54 |
| *Ets2* | NM_011809.2 | E26 avian leukemia oncogene 2, 3' domain (Ets2) | 0.38 |
| *Etv1* | NM_007960.3 | ets variant gene 1 (Etv1) | 0.29 |
| *Etv4* | NM_008815.2 | ets variant gene 4 (E1A enhancer binding protein, E1AF) (Etv4) | 0.33 |
| *Etv5* | NM_023794.2 | ets variant gene 5 (Etv5) | 0.20 |
| *Exosc1* | NM_025644.3 | exosome component 1 (Exosc1) | 0.52 |
| *Exosc2* | NM_144886.1 | exosome component 2 (Exosc2) | 0.63 |
| *Exosc8* | NM_027148.2 | exosome component 8 (Exosc8) | 0.52 |
| *Ext1* | NM_010162.2 | exostoses (multiple) 1 (Ext1) | 0.57 |
| *Ezh2* | NM_007971.1 | enhancer of zeste homolog 2 (Drosophila) (Ezh2) | 0.27 |
| *F2r* | NM_010169.3 | coagulation factor II (thrombin) receptor (F2r) | 0.24 |
| *F630043A04Rik* | NM_198605.1 | RIKEN cDNA F630043A04 gene (F630043A04Rik) | 0.56 |
| *Faah* | NM_010173.3 | fatty acid amide hydrolase (Faah) | 0.60 |
| *Fabp5* | NM_010634.2 | fatty acid binding protein 5, epidermal (Fabp5) | 0.29 |
| *Fabp7* | NM_021272.2 | | 0.45 |
| *Fam101a* | NM_028443.2 | family with sequence similarity 101, member A (Fam101a) | 0.51 |
| *Fam108a* | NM_145421.1 | family with sequence similarity 108, member A (Fam108a) | 0.63 |
| *Fam108b* | NM_146096.3 | family with sequence similarity 108, member B (Fam108b) | 0.66 |
| *Fam120a* | NM_001033268.2 | family with sequence similarity 120, member A (Fam120a) | 0.48 |
| *Fam131a* | NM_133778.2 | family with sequence similarity 131, member A (Fam131a) | 0.48 |
| *Fam171a1* | NM_001081161.1 | family with sequence similarity 171, member A1 (Fam171a1) | 0.50 |
| *Fam171b* | NM_175514.2 | family with sequence similarity 171, member B (Fam171b) | 0.37 |
| *Fam173a* | NM_145410.3 | family with sequence similarity 173, member A (Fam173a) | 0.58 |
| *Fam84a* | NM_029007.2 | family with sequence similarity 84, member A (Fam84a) | 0.33 |
| *Fancd2* | XM_132796.4 | | 0.66 |
| *Fance* | XM_986338.2 | PREDICTED: Fanconi anemia, complementation group E (Fance) | 0.59 |
| *Farsb* | NM_011811.2 | phenylalanyl-tRNA synthetase, beta subunit (Farsb) | 0.46 |
| *Fblim1* | NM_133754.3 | filamin binding LIM protein 1 (Fblim1) | 0.54 |
| *Fbln1* | NM_010180 | | 0.55 |
| *Fbln2* | NM_001081437.1 | fibulin 2 (Fbln2), transcript variant 2 | 0.13 |
| *Fbn1* | NM_007993 | | 0.46 |
| *Fbxl10* | NM_001005866.1 | F-box and leucine-rich repeat protein 10 (Fbxl10), transcript variant 3 | 0.50 |
| *Fbxl14* | NM_133940.1 | F-box and leucine-rich repeat protein 14 (Fbxl14) | 0.66 |
| *Fchsd2* | NM_199012.1 | FCH and double SH3 domains 2 (Fchsd2) | 0.56 |
| *Fdps* | NM_134469.3 | farnesyl diphosphate synthetase (Fdps) | 0.20 |
| *Fen1* | NM_007999.3 | flap structure specific endonuclease 1 (Fen1) | 0.34 |
| *Fgf1* | NM_010197.3 | fibroblast growth factor 1 (Fgf1) | 0.49 |
| *Fgf22* | NM_023304.1 | fibroblast growth factor 22 (Fgf22) | 0.60 |
| *Fh1* | NM_010209.1 | fumarate hydratase 1 (Fh1) | 0.45 |
| *Fibp* | NM_021438.2 | fibroblast growth factor (acidic) intracellular binding protein (Fibp) | 0.54 |
| *Fignl1* | NM_021891.2 | fidgetin-like 1 (Fignl1) | 0.25 |
| *Fkbp11* | NM_024169.3 | FK506 binding protein 11 (Fkbp11) | 0.41 |
| *Fkbp1a* | NM_008019.2 | FK506 binding protein 1a (Fkbp1a) | 0.39 |
| *Fkbp4* | NM_010219.3 | FK506 binding protein 4 (Fkbp4) | 0.66 |
| *Fkbp5* | NM_010220.2 | FK506 binding protein 5 (Fkbp5) | 0.61 |
| *Fmnl2* | NM_172409.2 | formin-like 2 (Fmnl2) | 0.35 |
| *Fnbp1l* | NM_153118.1 | formin binding protein 1-like (Fnbp1l) | 0.55 |
| *Fndc3b* | NM_173182.1 | fibronectin type III domain containing 3B (Fndc3b) | 0.38 |
| *Foxk2* | NM_001080932.1 | forkhead box K2 (Foxk2) | 0.62 |
| *Foxm1* | NM_008021.4 | forkhead box M1 (Foxm1) | 0.43 |
| *Foxred1* | NM_172291.1 | FAD-dependent oxidoreductase domain containing 1 (Foxred1) | 0.66 |
| *Fpgs* | NM_010236.1 | folylpolyglutamyl synthetase (Fpgs) | 0.66 |
| *Frmd4a* | NM_172475 | | 0.45 |
| *Fscn1* | NM_007984.2 | fascin homolog 1, actin bundling protein (Strongylocentrotus purpuratus) (Fscn1) | 0.22 |
| *Fubp3* | NM_001033389.2 | far upstream element (FUSE) binding protein 3 (Fubp3) | 0.61 |
| *Fus* | NM_139149.1 | | 0.48 |
| *Fut8* | NM_016893.2 | fucosyltransferase 8 (Fut8) | 0.39 |
| *Fxc1* | NM_019502.2 | fractured callus expressed transcript 1 (Fxc1) | 0.40 |
| *Fxn* | NM_008044.1 | frataxin (Fxn) | 0.46 |
| *Fxyd6* | NM_022004.6 | FXYD domain-containing ion transport regulator 6 (Fxyd6) | 0.48 |
| *Fyn* | NM_008054.1 | Fyn proto-oncogene (Fyn) | 0.56 |
| *Fzd1* | NM_021457.2 | frizzled homolog 1 (Drosophila) (Fzd1) | 0.53 |
| *Fzd5* | NM_022721.3 | frizzled homolog 5 (Drosophila) (Fzd5), transcript variant 1 | 0.51 |
| *Fzr1* | NM_019757.1 | fizzy/cell division cycle 20 related 1 (Drosophila) (Fzr1) | 0.64 |
| *G3bp1* | NM_013716.2 | Ras-GTPase-activating protein SH3-domain binding protein 1 (G3bp1) | 0.54 |
| *G3bp2* | NM_001080794.1 | GTPase activating protein (SH3 domain) binding protein 2 (G3bp2), transcript variant 2 | 0.62 |

| | | | |
|---|---|---|---|
| *Gad1* | NM_008077.4 | glutamic acid decarboxylase 1 (Gad1) | 0.44 |
| *Gadd45a* | NM_007836.1 | growth arrest and DNA-damage-inducible 45 alpha (Gadd45a) | 0.52 |
| *Galnt1* | NM_013814.2 | UDP-N-acetyl-alpha-D-galactosamine:polypeptide N-acetylgalactosaminyltransferase 1 (Galnt1) | 0.56 |
| *Galnt10* | NM_134189.2 | UDP-N-acetyl-alpha-D-galactosamine:polypeptide N-acetylgalactosaminyltransferase 10 (Galnt10) | 0.49 |
| *Galnt2* | NM_139272.2 | UDP-N-acetyl-alpha-D-galactosamine:polypeptide N-acetylgalactosaminyltransferase 2 (Galnt2) | 0.60 |
| *Gamt* | NM_010255.1 | guanidinoacetate methyltransferase (Gamt) | 0.61 |
| *Ganab* | NM_008060.1 | alpha glucosidase 2 alpha neutral subunit (Ganab) | 0.54 |
| *Gap43* | NM_008083.2 | growth associated protein 43 (Gap43) | 0.43 |
| *Gapdh* | NM_008084.2 | glyceraldehyde-3-phosphate dehydrogenase (Gapdh) | 0.60 |
| *Gars* | NM_180678.3 | glycyl-tRNA synthetase (Gars) | 0.46 |
| *Gart* | NM_010256.2 | phosphoribosylglycinamide formyltransferase (Gart) | 0.60 |
| *Gas2l3* | NM_001079876.1 | growth arrest-specific 2 like 3 (Gas2l3), transcript variant 2 | 0.27 |
| *Gas7* | NM_008088.1 | growth arrest specific 7 (Gas7) | 0.59 |
| *Gatad1* | NM_026033.1 | GATA zinc finger domain containing 1 (Gatad1) | 0.59 |
| *Gcap14* | NM_028407.3 | granule cell antiserum positive 14 (Gcap14), transcript variant 2 | 0.35 |
| *Gcat* | NM_013847 | | 0.65 |
| *Gch1* | NM_008102.3 | GTP cyclohydrolase 1 (Gch1) | 0.63 |
| *Gcs1* | NM_020619.2 | glucosidase 1 (Gcs1) | 0.46 |
| *Gdf15* | NM_011819.1 | growth differentiation factor 15 (Gdf15) | 0.30 |
| *Gdpd1* | NM_025638.1 | glycerophosphodiester phosphodiesterase domain containing 1 (Gdpd1) | 0.56 |
| *Gemin4* | NM_177367.2 | gem (nuclear organelle) associated protein 4 (Gemin4) | 0.53 |
| *Gfm1* | NM_138591.1 | G elongation factor, mitochondrial 1 (Gfm1) | 0.46 |
| *Gins2* | NM_178856.1 | GINS complex subunit 2 (Psf2 homolog) (Gins2) | 0.53 |
| *Gipc1* | NM_018771.3 | GIPC PDZ domain containing family, member 1 (Gipc1) | 0.54 |
| *Glb1* | NM_009752.1 | galactosidase, beta 1 (Glb1) | 0.44 |
| *Gldc* | NM_138595.1 | glycine decarboxylase (Gldc) | 0.46 |
| *Glg1* | NM_009149 | | 0.61 |
| *Glrx5* | NM_028419.2 | glutaredoxin 5 homolog (S. cerevisiae) (Glrx5) | 0.58 |
| *Glt25d1* | NM_146211.2 | glycosyltransferase 25 domain containing 1 (Glt25d1) | 0.39 |
| *Glt25d2* | NM_177756.3 | glycosyltransferase 25 domain containing 2 (Glt25d2) | 0.59 |
| *Gm444* | XM_144253.6 | PREDICTED: gene model 444, (NCBI) (Gm444) | 0.53 |
| *Gm561* | NM_001033297.2 | gene model 561, (NCBI) (Gm561) | 0.45 |
| *Gm949* | NM_001033446.2 | gene model 949, (NCBI) (Gm949) | 0.65 |
| *Gmds* | NM_146041.2 | GDP-mannose 4, 6-dehydratase (Gmds) | 0.62 |
| *Gna11* | NM_010301.3 | guanine nucleotide binding protein, alpha 11 (Gna11) | 0.66 |
| *Gnai1* | NM_010305.1 | guanine nucleotide binding protein (G protein), alpha inhibiting 1 (Gnai1) | 0.38 |
| *Gnb1* | NM_008142.3 | guanine nucleotide binding protein (G protein), beta 1 (Gnb1) | 0.55 |
| *Gnb4* | NM_013531.3 | guanine nucleotide binding protein (G protein), beta 4 (Gnb4) | 0.51 |
| *Gng10* | NM_025277.3 | guanine nucleotide binding protein (G protein), gamma 10 (Gng10) | 0.66 |
| *Gng12* | AK005561 | | 0.46 |
| *Gng2* | NM_001038637.1 | guanine nucleotide binding protein (G protein), gamma 2 (Gng2), transcript variant 2 | 0.60 |
| *Gnl3* | NM_178846.1 | guanine nucleotide binding protein-like 3 (nucleolar) (Gnl3), transcript variant 2 | 0.43 |
| *Gnpda1* | NM_011937.1 | glucosamine-6-phosphate deaminase 1 (Gnpda1) | 0.51 |
| *Golim4* | NM_175193.5 | golgi integral membrane protein 4 (Golim4) | 0.54 |
| *Golm1* | NM_027307.4 | golgi membrane protein 1 (Golm1), transcript variant 1 | 0.15 |
| *Gorasp2* | NM_027352.3 | golgi reassembly stacking protein 2 (Gorasp2) | 0.47 |
| *Got2* | NM_010325.2 | glutamate oxaloacetate transaminase 2, mitochondrial (Got2), nuclear gene encoding mitochondrial protein | 0.48 |
| *Gpc1* | NM_016696.3 | glypican 1 (Gpc1) | 0.60 |
| *Gpd1* | NM_010271.2 | glycerol-3-phosphate dehydrogenase 1 (soluble) (Gpd1) | 0.58 |
| *Gpd2* | NM_010274.2 | glycerol phosphate dehydrogenase 2, mitochondrial (Gpd2), nuclear gene encoding mitochondrial protein | 0.58 |
| *Gphn* | NM_172952.2 | gephyrin (Gphn) | 0.64 |
| *Gpi1* | NM_008155.2 | glucose phosphate isomerase 1 (Gpi1) | 0.60 |
| *Gpnmb* | NM_053110.3 | glycoprotein (transmembrane) nmb (Gpnmb) | 0.44 |
| *Gpr125* | XM_132089.7 | PREDICTED: G protein-coupled receptor 125, transcript variant 1 (Gpr125) | 0.58 |
| *Gps1* | NM_145370.1 | G protein pathway suppressor 1 (Gps1) | 0.61 |
| *Gpsm1* | NM_153410.4 | G-protein signalling modulator 1 (AGS3-like, C. elegans) (Gpsm1) | 0.62 |
| *Gpx3* | NM_008161.2 | glutathione peroxidase 3 (Gpx3), transcript variant 2 | 0.59 |
| *Grcc10* | NM_013535.1 | gene rich cluster, C10 gene (Grcc10) | 0.62 |
| *Gria2* | NM_001039195.1 | glutamate receptor, ionotropic, AMPA2 (alpha 2) (Gria2), transcript variant 1 | 0.55 |
| *Grip1* | NM_028736 | | 0.58 |
| *Grm5* | NM_001081414.1 | glutamate receptor, metabotropic 5 (Grm5) | 0.13 |
| *Grwd1* | NM_153419.1 | glutamate-rich WD repeat containing 1 (Grwd1) | 0.59 |
| *Gsg2* | NM_010353.1 | germ cell-specific gene 2 (Gsg2) | 0.54 |
| *Gspt1* | NM_146066.1 | G1 to S phase transition 1 (Gspt1) | 0.47 |

| | | | |
|---|---|---|---|
| *Gstp1* | NM_013541.1 | glutathione S-transferase, pi 1 (Gstp1) | 0.61 |
| *Gtf2e2* | NM_026584.2 | general transcription factor II E, polypeptide 2 (beta subunit) (Gtf2e2) | 0.54 |
| *Gtf2f2* | NM_026816.3 | general transcription factor IIF, polypeptide 2 (Gtf2f2) | 0.56 |
| *Gtf2h2* | NM_022011.3 | general transcription factor II H, polypeptide 2 (Gtf2h2) | 0.64 |
| *Gtf3a* | NM_025652.1 | general transcription factor III A (Gtf3a) | 0.63 |
| *Gtpbp3* | NM_032544.3 | GTP binding protein 3 (Gtpbp3), nuclear gene encoding mitochondrial protein | 0.42 |
| *Gtpbp4* | NM_027000.4 | GTP binding protein 4 (Gtpbp4) | 0.50 |
| *Gtse1* | NM_013882.1 | G two S phase expressed protein 1 (Gtse1) | 0.57 |
| *Gucy1a3* | NM_021896.4 | guanylate cyclase 1, soluble, alpha 3 (Gucy1a3) | 0.39 |
| *Gusb* | NM_010368.1 | glucuronidase, beta (Gusb) | 0.54 |
| *H1fx* | NM_198622.1 | H1 histone family, member X (H1fx) | 0.53 |
| *H2afx* | NM_010436.2 | H2A histone family, member X (H2afx) | 0.26 |
| *H2afz* | NM_016750.1 | H2A histone family, member Z (H2afz) | 0.24 |
| *Hat1* | NM_026115.3 | histone aminotransferase 1 (Hat1) | 0.44 |
| *Hbegf* | NM_010415.1 | heparin-binding EGF-like growth factor (Hbegf) | 0.36 |
| *Hccs* | NM_008222.2 | holocytochrome c synthetase (Hccs) | 0.63 |
| *Hdac2* | NM_008229.1 | | 0.45 |
| *Heatr1* | NM_144835.4 | HEAT repeat containing 1 (Heatr1) | 0.53 |
| *Heatr3* | NM_172757.3 | HEAT repeat containing 3 (Heatr3) | 0.66 |
| *Hectd1* | XM_974905.2 | PREDICTED: HECT domain containing 1 (Hectd1) | 0.63 |
| *Hectd2* | NM_172637.1 | HECT domain containing 2 (Hectd2) | 0.22 |
| *Hes1* | NM_008235.2 | hairy and enhancer of split 1 (Drosophila) (Hes1) | 0.52 |
| *Hes6* | NM_019479.2 | hairy and enhancer of split 6 (Drosophila) (Hes6) | 0.58 |
| *Hip1* | NM_146001.1 | huntingtin interacting protein 1 (Hip1) | 0.10 |
| *HIP-1* | AK087586 | | 0.52 |
| *Hip2* | NM_016786.2 | | 0.62 |
| *Hirip3* | NM_172746.2 | HIRA interacting protein 3 (Hirip3) | 0.51 |
| *Hist1h2ad* | NM_178188.3 | histone cluster 1, H2ad (Hist1h2ad) | 0.18 |
| *Hist1h2af* | NM_175661.1 | histone cluster 1, H2af (Hist1h2af) | 0.29 |
| *Hist1h2ag* | NM_178186.2 | histone cluster 1, H2ag (Hist1h2ag) | 0.25 |
| *Hist1h2ah* | NM_175659.1 | histone cluster 1, H2ah (Hist1h2ah) | 0.12 |
| *Hist1h2ai* | NM_178182.1 | histone cluster 1, H2ai (Hist1h2ai) | 0.23 |
| *Hist1h2ak* | NM_178183.1 | histone cluster 1, H2ak (Hist1h2ak) | 0.14 |
| *Hist1h2an* | NM_178184.1 | histone cluster 1, H2an (Hist1h2an) | 0.08 |
| *Hist1h2ao* | NM_178185.1 | histone cluster 1, H2ao (Hist1h2ao) | 0.08 |
| *Hist2h2ab* | NM_178213.3 | histone cluster 2, H2ab (Hist2h2ab) | 0.32 |
| *Hist2h2ac* | NM_175662.1 | histone cluster 2, H2ac (Hist2h2ac) | 0.21 |
| *Hmga1* | NM_016660.2 | high mobility group AT-hook 1 (Hmga1), transcript variant 1 | 0.36 |
| *Hmgb2* | NM_008252.2 | high mobility group box 2 (Hmgb2) | 0.31 |
| *Hmgcl* | NM_008254.1 | 3-hydroxy-3-methylglutaryl-Coenzyme A lyase (Hmgcl) | 0.57 |
| *Hmgcr* | NM_008255.1 | 3-hydroxy-3-methylglutaryl-Coenzyme A reductase (Hmgcr) | 0.48 |
| *Hmgn2* | NM_016957.3 | high mobility group nucleosomal binding domain 2 (Hmgn2) | 0.56 |
| *Hn1* | NM_008258.1 | hematological and neurological expressed sequence 1 (Hn1) | 0.29 |
| *Hn1l* | NM_198937.2 | hematological and neurological expressed 1-like (Hn1l) | 0.54 |
| *Hnrnpa2b1* | NM_182650.3 | heterogeneous nuclear ribonucleoprotein A2/B1 (Hnrnpa2b1), transcript variant 2 | 0.37 |
| *Hnrnpab* | NM_001048061.1 | heterogeneous nuclear ribonucleoprotein A/B (Hnrnpab), transcript variant 1 | 0.38 |
| *Hnrnpf* | NM_133834.1 | heterogeneous nuclear ribonucleoprotein F (Hnrnpf) | 0.64 |
| *Hnrnph1* | NM_021510.2 | heterogeneous nuclear ribonucleoprotein H1 (Hnrnph1) | 0.63 |
| *Hnrnpk* | NM_025279.2 | heterogeneous nuclear ribonucleoprotein K (Hnrnpk) | 0.56 |
| *Hnrpa1* | NM_010447 | | 0.34 |
| *Hnrpab* | AK013709 | | 0.62 |
| *Hnrpdl* | NM_016690.4 | heterogeneous nuclear ribonucleoprotein D-like (Hnrpdl) | 0.65 |
| *Hnrpl* | NM_177301.4 | heterogeneous nuclear ribonucleoprotein L (Hnrpl) | 0.44 |
| *Hnrpll* | NM_144802.3 | heterogeneous nuclear ribonucleoprotein L-like (Hnrpll) | 0.50 |
| *Hnrpm* | NM_029804.1 | heterogeneous nuclear ribonucleoprotein M (Hnrpm) | 0.60 |
| *Homer1* | NM_152134.1 | homer homolog 1 (Drosophila) (Homer1), transcript variant d | 0.47 |
| *Homer2* | XM_133550.4 | | 0.47 |
| *Hpcal1* | NM_016677.1 | hippocalcin-like 1 (Hpcal1) | 0.54 |
| *Hprt1* | NM_013556.2 | hypoxanthine guanine phosphoribosyl transferase 1 (Hprt1) | 0.65 |
| *Hrmt1l2* | NM_019830.1 | | 0.22 |
| *Hs2st1* | NM_011828.2 | heparan sulfate 2-O-sulfotransferase 1 (Hs2st1) | 0.65 |
| *Hs6st2* | NM_015819.3 | heparan sulfate 6-O-sulfotransferase 2 (Hs6st2), transcript variant 2 | 0.50 |
| *Hsd17b12* | NM_019657.2 | hydroxysteroid (17-beta) dehydrogenase 12 (Hsd17b12) | 0.39 |
| *Hsp90ab1* | NM_008302.3 | heat shock protein 90kDa alpha (cytosolic), class B member 1 (Hsp90ab1) | 0.56 |
| *Hsp90b1* | NM_011631.1 | heat shock protein 90, beta (Grp94), member 1 (Hsp90b1) | 0.53 |
| *Hspa8* | NM_031165.4 | heat shock protein 8 (Hspa8) | 0.53 |
| *Hspa9* | NM_010481.1 | heat shock protein 9 (Hspa9) | 0.57 |
| *Hspd1* | NM_010477.3 | heat shock protein 1 (chaperonin) (Hspd1) | 0.33 |
| *Hspe1* | NM_008303.2 | heat shock protein 1 (chaperonin 10) (Hspe1) | 0.43 |

| | | | |
|---|---|---|---|
| *Htra1* | NM_019564.1 | HtrA serine peptidase 1 (Htra1) | 0.53 |
| *Htra2* | NM_019752.2 | HtrA serine peptidase 2 (Htra2), nuclear gene encoding mitochondrial protein | 0.64 |
| *Hyal2* | NM_010489.2 | hyaluronoglucosaminidase 2 (Hyal2) | 0.51 |
| *Iars* | NM_172015.1 | isoleucine-tRNA synthetase (Iars) | 0.49 |
| *Iars2* | NM_198653.1 | isoleucine-tRNA synthetase 2, mitochondrial (Iars2) | 0.66 |
| *Ibtk* | XM_135030.4 | | 0.65 |
| *Ick* | NM_019987.1 | intestinal cell kinase (Ick) | 0.61 |
| *Ide* | NM_031156.2 | insulin degrading enzyme (Ide) | 0.42 |
| *Idh1* | NM_010497.2 | isocitrate dehydrogenase 1 (NADP+), soluble (Idh1) | 0.61 |
| *Idh3a* | NM_029573.2 | isocitrate dehydrogenase 3 (NAD+) alpha (Idh3a), nuclear gene encoding mitochondrial protein | 0.48 |
| *Ier3* | NM_133662.2 | immediate early response 3 (Ier3) | 0.33 |
| *Ier5l* | NM_030244.3 | immediate early response 5-like (Ier5l) | 0.38 |
| *Igf2r* | NM_010515.1 | insulin-like growth factor 2 receptor (Igf2r) | 0.54 |
| *Igfbp2* | NM_008342.2 | insulin-like growth factor binding protein 2 (Igfbp2) | 0.06 |
| *Igfbp3* | NM_008343.2 | insulin-like growth factor binding protein 3 (Igfbp3) | 0.07 |
| *Igfbp4* | NM_010517.3 | insulin-like growth factor binding protein 4 (Igfbp4) | 0.04 |
| *Ilf2* | NM_026374.3 | interleukin enhancer binding factor 2 (Ilf2) | 0.59 |
| *Immt* | NM_029673.2 | inner membrane protein, mitochondrial (Immt), nuclear gene encoding mitochondrial protein | 0.43 |
| *Imp3* | NM_133976.1 | IMP3, U3 small nucleolar ribonucleoprotein, homolog (yeast) (Imp3) | 0.46 |
| *Impa2* | NM_053261.2 | inositol (myo)-1(or 4)-monophosphatase 2 (Impa2) | 0.56 |
| *Impad1* | NM_177730.3 | inositol monophosphatase domain containing 1 (Impad1) | 0.42 |
| *Impdh2* | NM_011830 | | 0.49 |
| *Incenp* | NM_016692.1 | inner centromere protein (Incenp) | 0.37 |
| *Inpp5f* | NM_178641.3 | inositol polyphosphate-5-phosphatase F (Inpp5f) | 0.52 |
| *Insig1* | NM_153526.2 | insulin induced gene 1 (Insig1) | 0.26 |
| *Insm1* | NM_016889.2 | insulinoma-associated 1 (Insm1) | 0.66 |
| *Ints7* | NM_178632.4 | integrator complex subunit 7 (Ints7) | 0.52 |
| *Ipo5* | NM_023579.4 | importin 5 (Ipo5) | 0.48 |
| *Ipo9* | NM_153774.1 | importin 9 (Ipo9) | 0.62 |
| *Iqgap1* | NM_016721 | | 0.53 |
| *Iqgap3* | NM_001033484.1 | IQ motif containing GTPase activating protein 3 (Iqgap3) | 0.49 |
| *Isca1* | NM_026921.2 | iron-sulfur cluster assembly 1 homolog (S. cerevisiae) (Isca1) | 0.57 |
| *Isg20l2* | NM_177663.2 | interferon stimulated exonuclease gene 20-like 2 (Isg20l2) | 0.63 |
| *Itgav* | NM_008402.1 | integrin alpha V (Itgav) | 0.57 |
| *Itgb1* | NM_010578.1 | integrin beta 1 (fibronectin receptor beta) (Itgb1) | 0.62 |
| *Itpr2* | NM_019923.3 | inositol 1,4,5-triphosphate receptor 2 (Itpr2), transcript variant 1 | 0.33 |
| *Jagn1* | NM_026365.2 | jagunal homolog 1 (Drosophila) (Jagn1) | 0.65 |
| *Jam2* | NM_023844.2 | junction adhesion molecule 2 (Jam2) | 0.60 |
| *Jmjd6* | NM_033398.2 | jumonji domain containing 6 (Jmjd6) | 0.52 |
| *Josd2* | NM_025368.3 | Josephin domain containing 2 (Josd2) | 0.60 |
| *Kars* | NM_053092.1 | lysyl-tRNA synthetase (Kars) | 0.57 |
| *Kbtbd2* | NM_145958.1 | kelch repeat and BTB (POZ) domain containing 2 (Kbtbd2) | 0.63 |
| *Kcnc1* | NM_008421.2 | potassium voltage gated channel, Shaw-related subfamily, member 1 (Kcnc1) | 0.37 |
| *Kcnc4* | NM_145922.2 | potassium voltage gated channel, Shaw-related subfamily, member 4 (Kcnc4) | 0.35 |
| *Kcnd2* | NM_019697.3 | potassium voltage-gated channel, Shal-related family, member 2 (Kcnd2) | 0.54 |
| *Kcnip3* | NM_019789.2 | Kv channel interacting protein 3, calsenilin (Kcnip3) | 0.36 |
| *Kcnk13* | NM_146037.1 | potassium channel, subfamily K, member 13 (Kcnk13) | 0.61 |
| *Kctd12* | NM_177715.4 | potassium channel tetramerisation domain containing 12 (Kctd12) | 0.33 |
| *Kdelc1* | NM_023645.2 | KDEL (Lys-Asp-Glu-Leu) containing 1 (Kdelc1) | 0.63 |
| *Kdelr2* | NM_025841.3 | KDEL (Lys-Asp-Glu-Leu) endoplasmic reticulum protein retention receptor 2 (Kdelr2) | 0.45 |
| *Khdrbs1* | NM_011317.3 | KH domain containing, RNA binding, signal transduction associated 1 (Khdrbs1) | 0.51 |
| *Kif11* | NM_010615.1 | kinesin family member 11 (Kif11) | 0.39 |
| *Kif15* | NM_010620.1 | kinesin family member 15 (Kif15) | 0.36 |
| *Kif18a* | NM_139303.1 | kinesin family member 18A (Kif18a) | 0.34 |
| *Kif20b* | NM_183046.1 | kinesin family member 20B (Kif20b) | 0.32 |
| *Kif22* | NM_145588.1 | kinesin family member 22 (Kif22) | 0.17 |
| *Kif23* | NM_024245 | | 0.10 |
| *Kif2a* | NM_008442.1 | kinesin family member 2A (Kif2a) | 0.64 |
| *Kif2c* | NM_134471.3 | kinesin family member 2C (Kif2c) | 0.26 |
| *Kif3c* | NM_008445.2 | kinesin family member 3C (Kif3c) | 0.33 |
| *Kif4* | NM_008446.1 | kinesin family member 4 (Kif4) | 0.32 |
| *Kifc1* | NM_016761.1 | | 0.51 |
| *Kirrel3* | NM_026324.2 | kin of IRRE like 3 (Drosophila) (Kirrel3) | 0.42 |
| *Kitl* | NM_013598.1 | kit ligand (Kitl) | 0.58 |
| *Klf6* | NM_011803.2 | Kruppel-like factor 6 (Klf6) | 0.63 |

| | | | |
|---|---|---|---|
| *Klf7* | NM_033563 | | 0.65 |
| *Klhdc2* | NM_027117.1 | kelch domain containing 2 (Klhdc2) | 0.60 |
| *Kntc1* | NM_001042421.1 | kinetochore associated 1 (Kntc1) | 0.36 |
| *Kpna1* | NM_008465.4 | karyopherin (importin) alpha 1 (Kpna1) | 0.57 |
| *Kpna2* | NM_010655.3 | karyopherin (importin) alpha 2 (Kpna2) | 0.55 |
| *Kpnb1* | NM_008379.3 | karyopherin (importin) beta 1 (Kpnb1) | 0.57 |
| *Kras* | NM_021284.4 | v-Ki-ras2 Kirsten rat sarcoma viral oncogene homolog (Kras) | 0.60 |
| *Krtcap2* | NM_025327.2 | keratinocyte associated protein 2 (Krtcap2) | 0.57 |
| *Kti12* | NM_029571.2 | KTI12 homolog, chromatin associated (S. cerevisiae) (Kti12) | 0.61 |
| *L2hgdh* | NM_145443.1 | L-2-hydroxyglutarate dehydrogenase (L2hgdh), nuclear gene encoding mitochondrial protein | 0.64 |
| *Lama5* | NM_001081171.2 | laminin, alpha 5 (Lama5) | 0.49 |
| *Lamc1* | NM_010683.1 | laminin, gamma 1 (Lamc1) | 0.48 |
| *Lass2* | NM_029789.1 | LAG1 homolog, ceramide synthase 2 (Lass2) | 0.63 |
| *Lass5* | NM_028015.2 | LAG1 homolog, ceramide synthase 5 (Lass5) | 0.63 |
| *Lbh* | NM_029999.3 | limb-bud and heart (Lbh) | 0.32 |
| *Lbr* | NM_133815 | | 0.24 |
| *Ldha* | NM_010699.1 | lactate dehydrogenase A (Ldha) | 0.32 |
| *Leo1* | NM_001039522.1 | Leo1, Paf1/RNA polymerase II complex component, homolog (S. cerevisiae) (Leo1) | 0.64 |
| *Lgals1* | NM_008495.1 | | 0.16 |
| *Lig1* | NM_010715.2 | ligase I, DNA, ATP-dependent (Lig1), transcript variant 2 | 0.29 |
| *Lig3* | NM_010716 | | 0.64 |
| *Lima1* | NM_023063.1 | LIM domain and actin binding 1 (Lima1) | 0.64 |
| *Limd1* | NM_013860.1 | LIM domains containing 1 (Limd1) | 0.62 |
| *Lin54* | NM_172714.1 | lin-54 homolog (C. elegans) (Lin54) | 0.49 |
| *Lip1* | NM_021460.1 | | 0.38 |
| *Liph* | NM_153404.2 | lipase, member H (Liph), transcript variant 2 | 0.55 |
| *Litaf* | NM_019980.1 | LPS-induced TN factor (Litaf) | 0.42 |
| *Lman1* | NM_027400.2 | lectin, mannose-binding, 1 (Lman1) | 0.67 |
| *Lmnb1* | NM_010721.1 | lamin B1 (Lmnb1) | 0.13 |
| *Lmo2* | NM_008505.3 | LIM domain only 2 (Lmo2) | 0.29 |
| *LOC100039346* | XM_001472500.1 | PREDICTED: hypothetical protein LOC100039346 (LOC100039346) | 0.51 |
| *LOC100039532* | XM_001473099.1 | PREDICTED: similar to ribosomal protein L35a (LOC100039532) | 0.58 |
| *LOC100039571* | XM_001473215.1 | PREDICTED: similar to ribosomal protein L23a (LOC100039571) | 0.34 |
| *LOC100039751* | XR_031048.1 | PREDICTED: similar to 40S ribosomal protein S12 (LOC100039751), misc RNA. | 0.61 |
| *LOC100039786* | XM_001473500.1 | PREDICTED: similar to Ywhaq protein (LOC100039786) | 0.66 |
| *LOC100040573* | XM_001475112.1 | PREDICTED: similar to putative transcription factor ZNF131, transcript variant 1 (LOC100040573) | 0.50 |
| *LOC100040592* | XM_001475189.1 | PREDICTED: similar to Hmgcs1 protein, transcript variant 1 (LOC100040592) | 0.58 |
| *LOC100040605* | XM_001475096.1 | PREDICTED: similar to ribosomal protein L13 (LOC100040605) | 0.48 |
| *LOC100041500* | XM_001478166.1 | PREDICTED: similar to LSM7 homolog, U6 small nuclear RNA associated (LOC100041500) | 0.61 |
| *LOC100041835* | XM_001478119.1 | PREDICTED: similar to H+ ATP synthase (LOC100041835) | 0.50 |
| *LOC100042405* | XM_001478555.1 | PREDICTED: similar to high mobility group nucleosomal binding domain 2 (LOC100042405) | 0.34 |
| *LOC100042427* | XM_001478518.1 | PREDICTED: similar to Glyceraldehyde-3-phosphate dehydrogenase (GAPDH), transcript variant 4 (LOC100042427) | 0.61 |
| *LOC100042777* | XM_001478939.1 | PREDICTED: similar to human protein homologous to DROER protein (LOC100042777) | 0.62 |
| *LOC100042970* | XM_001479207.1 | PREDICTED: similar to Kifc1 protein (LOC100042970) | 0.43 |
| *LOC100043192* | XM_001479822.1 | PREDICTED: similar to ribosomal protein L31 (LOC100043192) | 0.60 |
| *LOC100043209* | XM_001480293.1 | PREDICTED: similar to ribosomal protein L15 (LOC100043209) | 0.56 |
| *LOC100043257* | XM_001480197.1 | PREDICTED: similar to RNA binding motif protein 3 (LOC100043257) | 0.44 |
| *LOC100043391* | XM_001479903.1 | PREDICTED: similar to QM protein (LOC100043391) | 0.53 |
| *LOC100043527* | XM_001480106.1 | PREDICTED: similar to ribosomal protein S28 (LOC100043527) | 0.58 |
| *LOC100043555* | XM_001480194.1 | PREDICTED: similar to Aak1 protein (LOC100043555) | 0.52 |
| *LOC100043671* | XM_001481024.1 | PREDICTED: hypothetical protein LOC100043671 (LOC100043671) | 0.53 |
| *LOC100043675* | XM_001480621.1 | PREDICTED: similar to Translocase of inner mitochondrial membrane 23 homolog (yeast) (LOC100043675) | 0.61 |
| *LOC100043822* | XM_001481025.1 | PREDICTED: hypothetical protein LOC100043822 (LOC100043822) | 0.59 |
| *LOC100043906* | XM_001481214.1 | PREDICTED: similar to nuclear pore-targeting complex component of 58 kDa, transcript variant 1 (LOC100043906) | 0.37 |
| *LOC100044322* | XM_001471968.1 | PREDICTED: similar to UDP-glucose ceramide glucosyltransferase-like 1 (LOC100044322) | 0.54 |
| *LOC100044468* | XM_001472489.1 | PREDICTED: similar to nemo-like kinase (LOC100044468) | 0.54 |
| *LOC100044696* | XM_001473268.1 | PREDICTED: similar to cysteine-rich PAK1inhibitor (LOC100044696) | 0.63 |
| *LOC100044756* | XM_001472989.1 | PREDICTED: similar to PX domain-containing protein kinase-like protein (Modulator of Na,K-ATPase) (MONaKA) (LOC100044756) | 0.66 |

| | | | |
|---|---|---|---|
| *LOC100044829* | XM_001472872.1 | PREDICTED: similar to Fibrillarin, transcript variant 1 (LOC100044829) | 0.40 |
| *LOC100045040* | XR_031278.1 | PREDICTED: similar to ING1 protein (LOC100045040), misc RNA. | 0.61 |
| *LOC100045312* | XM_001473900.1 | PREDICTED: similar to ribosomal protein (LOC100045312) | 0.44 |
| *LOC100045343* | XR_031575.1 | PREDICTED: similar to CDNA sequence BC046404 (LOC100045343), misc RNA. | 0.53 |
| *LOC100045542* | XM_001473941.1 | PREDICTED: similar to FERMRhoGEF (Arhgef) and pleckstrin domain protein 1 (LOC100045542) | 0.64 |
| *LOC100045668* | XM_001474718.1 | PREDICTED: similar to Rpl17 protein (LOC100045668) | 0.64 |
| *LOC100045677* | XR_031705.1 | PREDICTED: similar to DNA replication licensing factor MCM3 (DNA polymerase alpha holoenzyme-associated protein P1) (P1-MCM3) (LOC100045677), misc RNA. | 0.49 |
| *LOC100045737* | XR_031944.1 | PREDICTED: hypothetical protein LOC100045737 (LOC100045737), misc RNA. | 0.64 |
| *LOC100045780* | XM_001475019.1 | PREDICTED: similar to metalloprotease-disintegrin meltrin beta (LOC100045780) | 0.18 |
| *LOC100045887* | XM_001475106.1 | PREDICTED: similar to PTB-associated splicing factor (LOC100045887) | 0.51 |
| *LOC100045967* | XR_032154.1 | PREDICTED: hypothetical protein LOC100045967 (LOC100045967), misc RNA. | 0.44 |
| *LOC100045981* | XM_001475298.1 | PREDICTED: similar to synaptotagmin XI (LOC100045981) | 0.50 |
| *LOC100045999* | XM_001475422.1 | PREDICTED: similar to RAN, member RAS oncogene family (LOC100045999) | 0.53 |
| *LOC100046320* | XR_032844.1 | PREDICTED: similar to Matrin 3 (LOC100046320), misc RNA. | 0.66 |
| *LOC100046343* | XM_001475830.1 | PREDICTED: similar to CCR4-NOT transcription complex, subunit 6 (LOC100046343) | 0.50 |
| *LOC100046483* | XM_001476285.1 | PREDICTED: similar to casein kinase II, alpha prime subunit (LOC100046483) | 0.64 |
| *LOC100046518* | XM_001476353.1 | PREDICTED: similar to Gprin1 protein (LOC100046518) | 0.56 |
| *LOC100046650* | XM_001476721.1 | PREDICTED: similar to PRELI domain containing 1 (LOC100046650) | 0.46 |
| *LOC100046744* | XM_001475765.1 | PREDICTED: similar to Serine/arginine repetitive matrix protein 2 (LOC100046744) | 0.55 |
| *LOC100046853* | XM_001476909.1 | PREDICTED: similar to heterogeneous nuclear ribonucleoprotein K (LOC100046853) | 0.29 |
| *LOC100046855* | XM_001476916.1 | PREDICTED: similar to BKLF (LOC100046855) | 0.38 |
| *LOC100046895* | XM_001477026.1 | PREDICTED: similar to Quaking protein (LOC100046895) | 0.48 |
| *LOC100047155* | XM_001477537.1 | PREDICTED: similar to Small nuclear ribonucleoprotein polypeptide A (LOC100047155) | 0.55 |
| *LOC100047167* | XM_001477568.1 | PREDICTED: similar to mKIAA0990 protein (LOC100047167) | 0.39 |
| *LOC100047268* | XM_001477774.1 | PREDICTED: similar to Immediate early response 5-like (LOC100047268) | 0.24 |
| *LOC100047353* | XM_001477963.1 | PREDICTED: similar to myocardial vascular inhibition factor (LOC100047353) | 0.60 |
| *LOC100047490* | XM_001478266.1 | PREDICTED: similar to putative oral cancer suppressor (LOC100047490) | 0.62 |
| *LOC100047606* | XR_033719.1 | PREDICTED: similar to neurotrophic tyrosine kinase, receptor, type 3 (LOC100047606), misc RNA. | 0.67 |
| *LOC100047615* | XR_033484.1 | PREDICTED: similar to 40S ribosomal protein S17 (LOC100047615), misc RNA. | 0.57 |
| *LOC100047619* | XR_033736.1 | PREDICTED: similar to solute carrier family 7 (cationic amino acid transporter, y+ system), member 5 (LOC100047619), misc RNA. | 0.19 |
| *LOC100047634* | XR_032896.1 | PREDICTED: similar to Eukaryotic translation initiation factor 2 alpha kinase 3 (LOC100047634), misc RNA. | 0.61 |
| *LOC100047712* | XM_001478724.1 | PREDICTED: similar to coiled-coil domain containing 101 (LOC100047712) | 0.65 |
| *LOC100047759* | XM_001479214.1 | PREDICTED: similar to acidic ribosomal phosphoprotein P1, transcript variant 1 (LOC100047759) | 0.49 |
| *LOC100047762* | XM_001478835.1 | PREDICTED: similar to Aspartate aminotransferase, cytoplasmic (Transaminase A) (Glutamate oxaloacetate transaminase 1) (LOC100047762) | 0.64 |
| *LOC100047827* | XM_001479392.1 | PREDICTED: similar to Hmgn2 protein (LOC100047827) | 0.39 |
| *LOC100047888* | XM_001479068.1 | PREDICTED: similar to Neurocan (LOC100047888) | 0.31 |
| *LOC100047915* | XM_001479122.1 | PREDICTED: similar to Memo1 protein (LOC100047915) | 0.61 |
| *LOC100047934* | XM_001478074.1 | PREDICTED: hypothetical protein LOC100047934 (LOC100047934) | 0.38 |
| *LOC100047935* | XR_033756.1 | PREDICTED: similar to ribosomal protein L5 (LOC100047935), misc RNA. | 0.63 |
| *LOC100047937* | XM_001479188.1 | PREDICTED: similar to Aldehyde dehydrogenase 1 family, member L1 (LOC100047937) | 0.64 |
| *LOC100047998* | XM_001479497.1 | PREDICTED: similar to ribosomal protein L37a (LOC100047998) | 0.51 |
| *LOC100048413* | XM_001480186.1 | PREDICTED: similar to Myeloid leukemia factor 2 (LOC100048413) | 0.52 |
| *LOC100048445* | XM_001480467.1 | PREDICTED: similar to fau (LOC100048445) | 0.57 |
| *LOC100048483* | XM_001480453.1 | PREDICTED: similar to cytochrome c oxidase subunit VIII (LOC100048483) | 0.59 |
| *LOC100048508* | XM_001480806.1 | PREDICTED: similar to Rpl36 protein, transcript variant 1 (LOC100048508) | 0.61 |
| *LOC100048613* | XM_001480380.1 | PREDICTED: similar to cytochrome c oxidase, subunit VIIc, transcript variant 1 (LOC100048613) | 0.56 |

| | | | |
|---|---|---|---|
| *LOC100048645* | XM_001480791.1 | PREDICTED: similar to cysteine and glycine-rich protein 2 binding protein (LOC100048645) | 0.60 |
| *LOC209281* | XR_002517.1 | PREDICTED: similar to capping protein (actin filament) muscle Z-line, alpha 1 (LOC209281) | 0.64 |
| *LOC212399* | XM_137003.2 | | 0.38 |
| *LOC219106* | XM_139078.2 | | 0.64 |
| *LOC219145* | XM_122761.3 | | 0.43 |
| *LOC225058* | XM_140116.1 | | 0.58 |
| *LOC225134* | XR_031257.1 | PREDICTED: similar to ribosomal protein S4, X-linked (LOC225134), misc RNA. | 0.51 |
| *LOC226416* | XM_129460.4 | | 0.61 |
| *LOC229810* | XM_124173.2 | | 0.66 |
| *LOC230253* | XM_124269.2 | | 0.29 |
| *LOC230896* | XM_124427.4 | | 0.64 |
| *LOC231368* | XR_002491.1 | PREDICTED: similar to 60S acidic ribosomal protein P0 (L10E) (LOC231368) | 0.41 |
| *LOC232606* | XM_124826.2 | | 0.48 |
| *LOC232887* | XM_124858.2 | | 0.66 |
| *LOC232890* | XM_145374.2 | | 0.49 |
| *LOC236831* | XR_001889.1 | PREDICTED: similar to 60S ribosomal protein L7a (LOC236831) | 0.48 |
| *LOC237512* | XM_137226.3 | | 0.51 |
| *LOC237877* | XM_111221.4 | | 0.57 |
| *LOC238943* | XM_138902.2 | | 0.65 |
| *LOC241621* | XM_917314.2 | PREDICTED: similar to ribosomal protein L27a-like (LOC241621) | 0.37 |
| *LOC245668* | XM_142236.4 | | 0.43 |
| *LOC245892* | XM_147335.2 | | 0.17 |
| *LOC268569* | XM_195821.2 | | 0.23 |
| *LOC268700* | XM_193742.2 | | 0.52 |
| *LOC269251* | XM_193262.3 | | 0.39 |
| *LOC269515* | XM_196313.1 | | 0.67 |
| *LOC270037* | XM_193374.2 | | 0.38 |
| *LOC271490* | XM_195069.2 | | 0.47 |
| *LOC271505* | XM_195066.3 | | 0.55 |
| *LOC272681* | XM_195399.2 | | 0.54 |
| *LOC277856* | XM_204210.2 | | 0.58 |
| *LOC329076* | XM_282724.2 | | 0.45 |
| *LOC331102* | XM_284573.2 | | 0.65 |
| *LOC331507* | XM_284733.2 | | 0.64 |
| *LOC380692* | XM_354604.1 | | 0.28 |
| *LOC380707* | XM_354622.1 | | 0.61 |
| *LOC380927* | XM_354834.1 | | 0.51 |
| *LOC380930* | XM_358467.1 | | 0.44 |
| *LOC381046* | XM_354963.1 | | 0.37 |
| *LOC381114* | XM_355032.1 | | 0.29 |
| *LOC381140* | XM_355056.1 | | 0.63 |
| *LOC381215* | XM_355149.1 | | 0.55 |
| *LOC381230* | XM_355167.1 | | 0.61 |
| *LOC381285* | XM_355225.1 | | 0.65 |
| *LOC381330* | XM_355288.1 | | 0.48 |
| *LOC381365* | XM_355330.1 | | 0.56 |
| *LOC381398* | XM_355356.1 | | 0.43 |
| *LOC381448* | XM_355403.1 | | 0.46 |
| *LOC381561* | XM_355526.1 | | 0.60 |
| *LOC381649* | XM_355612.1 | | 0.54 |
| *LOC381799* | XM_355801.1 | | 0.65 |
| *LOC381808* | XM_355809.1 | | 0.56 |
| *LOC381891* | XM_355907.1 | | 0.56 |
| *LOC381940* | XM_355964.1 | | 0.62 |
| *LOC381999* | XM_356062.1 | | 0.50 |
| *LOC382061* | XM_356141.1 | | 0.52 |
| *LOC382128* | XM_356225.1 | | 0.66 |
| *LOC382230* | XM_356342.1 | | 0.50 |
| *LOC382885* | XM_356731.1 | | 0.56 |
| *LOC383099* | XM_356873.1 | | 0.65 |
| *LOC383308* | XM_356977.1 | | 0.42 |
| *LOC383330* | XM_356987.1 | | 0.54 |
| *LOC383897* | XM_357315.1 | | 0.57 |
| *LOC383942* | XM_357338.1 | | 0.65 |
| *LOC384104* | XM_357429.1 | | 0.65 |
| *LOC384206* | XM_357488.1 | | 0.57 |
| *LOC384727* | XM_357823.1 | | 0.59 |

| | | | |
|---|---|---|---|
| *LOC385905* | XR_034995.1 | PREDICTED:  similar to proteasome alpha7/C8 subunit (LOC385905), misc RNA. | 0.65 |
| *LOC433476* | XM_917865.2 | PREDICTED:  similar to ribosomal protein L27a (LOC433476) | 0.37 |
| *LOC433745* | XM_001475733.1 | PREDICTED:  similar to ribosomal protein L3 (LOC433745) | 0.56 |
| *LOC545056* | NR_002889.1 | ubiquitin-conjugating enzyme E2, J2 homolog pseudogene (LOC545056) on chromosome 14. | 0.57 |
| *LOC545369* | XR_034542.1 | PREDICTED:  similar to ribosomal protein L4 (LOC545369), misc RNA. | 0.59 |
| *LOC545396* | XR_003545.1 | PREDICTED:  similar to TGF beta-inducible nuclear protein 1 (L-name-related protein 42) (LNR42) (LOC545396) | 0.62 |
| *LOC545472* | XM_619824.4 | PREDICTED:  similar to BTB (POZ) domain containing 1 (LOC545472) | 0.67 |
| *LOC546015* | XR_034790.1 | PREDICTED:  similar to ribosomal protein S9 (LOC546015), misc RNA. | 0.61 |
| *LOC620678* | XR_031536.1 | PREDICTED:  similar to 60S ribosomal protein L7 (LOC620678), misc RNA. | 0.55 |
| *LOC622655* | XR_032410.1 | PREDICTED:  similar to hCG31107 (LOC622655), misc RNA. | 0.33 |
| *LOC622994* | XR_031862.1 | PREDICTED:  hypothetical LOC622994 (LOC622994), misc RNA. | 0.47 |
| *LOC623466* | XR_033501.1 | PREDICTED:  similar to MGC27348 protein (LOC623466), misc RNA. | 0.43 |
| *LOC623568* | XM_001480472.1 | PREDICTED:  similar to ribosomal protein S20 (LOC623568) | 0.41 |
| *LOC630729* | XM_904332.3 | PREDICTED:  similar to glutathione reductase 1 (LOC630729) | 0.54 |
| *LOC630936* | XR_001819.2 | PREDICTED:  similar to nuclease sensitive element binding protein 1 (LOC630936), misc RNA. | 0.27 |
| *LOC632022* | XR_030945.1 | PREDICTED:  similar to 40S ribosomal protein S18 (LOC632022), misc RNA. | 0.65 |
| *LOC632230* | XR_031205.1 | PREDICTED:  similar to Ribosomal protein L5 (LOC632230), misc RNA. | 0.56 |
| *LOC632667* | XM_906862.3 | PREDICTED:  similar to PNG protein (LOC632667) | 0.57 |
| *LOC632684* | XR_031426.1 | PREDICTED:  hypothetical LOC632684 (LOC632684), misc RNA. | 0.59 |
| *LOC633016* | XM_921371.2 | PREDICTED:  similar to Chromobox homolog 3 (HP1 gamma homolog, Drosophila), transcript variant 2 (LOC633016) | 0.46 |
| *LOC635470* | XM_915945.3 | PREDICTED:  similar to ribosomal protein L27a-like (LOC635470) | 0.43 |
| *LOC638892* | XM_915073.1 | PREDICTED:  similar to cpn10 protein (LOC638892) | 0.62 |
| *LOC640739* | XM_925296.2 | PREDICTED:  hypothetical LOC640739 (LOC640739) | 0.16 |
| *LOC640972* | XM_918222.2 | PREDICTED:  similar to cyclin-dependent kinase 2-interacting protein (LOC640972) | 0.62 |
| *LOC654426* | NR_002690.1 | ATP synthase, H+ transporting, mitochondrial F0 complex, subunit F pseudogene (LOC654426), non-coding RNA. | 0.65 |
| *LOC654467* | NR_002885.1 | heterogeneous nuclear ribonucleoprotein A1 pseudogene (LOC654467) on chromosome 9. | 0.42 |
| *LOC654842* | NM_001081681.1 | hypothetical protein LOC654842 (LOC654842) | 0.39 |
| *LOC665032* | XM_991641.1 | PREDICTED:  similar to ribosomal protein (LOC665032) | 0.51 |
| *LOC665235* | XR_031650.1 | PREDICTED:  similar to hCG1642689 (LOC665235), misc RNA. | 0.63 |
| *LOC665237* | XR_031476.1 | PREDICTED:  similar to hCG31107 (LOC665237), misc RNA. | 0.33 |
| *LOC665281* | XR_031065.1 | PREDICTED:  similar to 40S ribosomal protein S12 (LOC665281), misc RNA. | 0.44 |
| *LOC666559* | XR_031145.1 | PREDICTED:  similar to farnesyl pyrophosphate synthase (LOC666559), misc RNA. | 0.26 |
| *LOC666676* | XR_030655.1 | PREDICTED:  similar to ADP-ribosylation factor (LOC666676), misc RNA. | 0.49 |
| *LOC667005* | XR_030797.1 | PREDICTED:  similar to cyclin B1 (LOC667005), misc RNA. | 0.43 |
| *LOC667251* | XR_032081.1 | PREDICTED:  similar to NADPH cytochrome B5 oxidoreductase (LOC667251), misc RNA. | 0.61 |
| *LOC667609* | XR_032582.1 | PREDICTED:  similar to mitochondrial ribosomal protein S5 (LOC667609), misc RNA. | 0.66 |
| *LOC668038* | XR_034201.1 | PREDICTED:  similar to ribosomal protein (LOC668038), misc RNA. | 0.46 |
| *LOC668239* | XM_001000226.2 | PREDICTED:  similar to Rps13 protein (LOC668239) | 0.47 |
| *LOC668387* | XR_031651.1 | PREDICTED:  similar to ribosomal protein S6 (LOC668387), misc RNA. | 0.54 |
| *LOC668492* | XR_034647.1 | PREDICTED:  similar to zinc finger CCHC-type and RNA binding motif 1 (LOC668492), misc RNA. | 0.63 |
| *LOC668573* | XR_035016.1 | PREDICTED:  similar to ribosomal protein (LOC668573), misc RNA. | 0.40 |
| *LOC668706* | XM_001003212.2 | PREDICTED:  similar to Ribosomal protein L12 (LOC668706) | 0.36 |
| *LOC669658* | XM_976371.1 | PREDICTED:  similar to melanoma antigen (LOC669658) | 0.54 |
| *LOC669660* | XM_976375.1 | PREDICTED:  similar to PDZ and LIM domain protein 5 (Enigma homolog) (Enigma-like PDZ and LIM domains protein) (LOC669660) | 0.62 |
| *LOC671641* | XM_001479052.1 | PREDICTED:  similar to ribosomal protein S12 (LOC671641) | 0.54 |
| *LOC671878* | XM_001473434.1 | PREDICTED:  similar to spermine synthase (LOC671878) | 0.37 |
| *LOC672474* | XM_993721.1 | PREDICTED:  similar to 60S ribosomal protein L29 (LOC672474) | 0.58 |
| *LOC674611* | XM_980150.1 | PREDICTED:  similar to Su48, transcript variant 3 (LOC674611) | 0.38 |
| *LOC675377* | XR_005113.2 | PREDICTED:  similar to Ribosomal protein L8 (LOC675377), misc RNA. | 0.51 |
| *LOC675440* | XM_001479274.1 | PREDICTED:  similar to SMT3 suppressor of mif two 3 homolog 2 (S. cerevisiae), transcript variant 1 (LOC675440) | 0.62 |
| *LOC675813* | XR_032773.1 | PREDICTED:  similar to ribosomal protein S20 (LOC675813), misc RNA. | 0.58 |
| *LOC675899* | XM_985882.1 | PREDICTED:  similar to H2A histone family, member Z (LOC675899) | 0.26 |
| *LOC676420* | XR_031436.1 | PREDICTED:  similar to ceramide kinases (LOC676420), misc RNA. | 0.57 |
| *LOC676640* | XM_001003712.1 | PREDICTED:  similar to neuron navigator 3, transcript variant 5 (LOC676640) | 0.32 |
| *LOC676724* | XM_992449.1 | PREDICTED:  hypothetical LOC676724 (LOC676724) | 0.47 |
| *LOC677205* | XR_034818.1 | PREDICTED:  similar to DEAD (Asp-Glu-Ala-Asp) box polypeptide 18 (LOC677205), misc RNA. | 0.53 |

| | | | |
|---|---|---|---|
| *LOC677215* | XM_001001411.2 | PREDICTED: similar to acidic ribosomal phosphoprotein P1, transcript variant 1 (LOC677215) | 0.64 |
| *LOC677317* | XM_001004685.1 | PREDICTED: similar to Mod1 protein, transcript variant 4 (LOC677317) | 0.53 |
| *LOC677448* | XR_005062.2 | PREDICTED: similar to actin (LOC677448), misc RNA. | 0.57 |
| *LOC677551* | XR_005084.2 | PREDICTED: similar to Ribosomal protein L19 (LOC677551), misc RNA. | 0.56 |
| *Lor* | NM_008508.2 | loricrin (Lor) | 0.31 |
| *Lphn3* | NM_198702.2 | latrophilin 3 (Lphn3) | 0.58 |
| *Lpl* | NM_008509.2 | lipoprotein lipase (Lpl) | 0.31 |
| *Lrig1* | NM_008377.2 | leucine-rich repeats and immunoglobulin-like domains 1 (Lrig1) | 0.33 |
| *Lrp12* | NM_172814.1 | low density lipoprotein-related protein 12 (Lrp12) | 0.54 |
| *Lrrc59* | NM_133807.1 | leucine rich repeat containing 59 (Lrrc59) | 0.44 |
| *Lrrfip1* | NM_008515.1 | leucine rich repeat (in FLII) interacting protein 1 (Lrrfip1) | 0.32 |
| *Lsg1* | NM_178069.5 | large subunit GTPase 1 homolog (S. cerevisiae) (Lsg1) | 0.64 |
| *Lsm12* | NM_172947.1 | LSM12 homolog (S. cerevisiae) (Lsm12) | 0.62 |
| *Lsm2* | NM_030597.2 | LSM2 homolog, U6 small nuclear RNA associated (S. cerevisiae) (Lsm2) | 0.44 |
| *Lsm3* | NM_026309.1 | LSM3 homolog, U6 small nuclear RNA associated (S. cerevisiae) (Lsm3) | 0.54 |
| *Lsm4* | NM_015816.1 | LSM4 homolog, U6 small nuclear RNA associated (S. cerevisiae) (Lsm4) | 0.47 |
| *Lsm5* | NM_025520.2 | LSM5 homolog, U6 small nuclear RNA associated (S. cerevisiae) (Lsm5) | 0.41 |
| *Lsm6* | NM_030145.2 | LSM6 homolog, U6 small nuclear RNA associated (S. cerevisiae) (Lsm6) | 0.48 |
| *Lsm8* | NM_133939.1 | LSM8 homolog, U6 small nuclear RNA associated (S. cerevisiae) (Lsm8) | 0.42 |
| *Lss* | NM_146006.1 | lanosterol synthase (Lss) | 0.40 |
| *Ltbp1* | NM_206958.1 | latent transforming growth factor beta binding protein 1 (Ltbp1), transcript variant 2 | 0.42 |
| *Ly96* | NM_016923.1 | lymphocyte antigen 96 (Ly96) | 0.58 |
| *Lyar* | NM_025281.2 | Ly1 antibody reactive clone (Lyar) | 0.60 |
| *Lypla2* | NM_011942.1 | lysophospholipase 2 (Lypla2) | 0.56 |
| *Mad2l1* | NM_019499.2 | MAD2 (mitotic arrest deficient, homolog)-like 1 (yeast) (Mad2l1) | 0.27 |
| *Maged1* | NM_019791.2 | melanoma antigen, family D, 1 (Maged1) | 0.36 |
| *Mageh1* | NM_023788.3 | melanoma antigen, family H, 1 (Mageh1) | 0.57 |
| *Magi1* | NM_010367.2 | membrane associated guanylate kinase, WW and PDZ domain containing 1 (Magi1), transcript variant 1 | 0.55 |
| *Mak16* | NM_026453.3 | MAK16 homolog (S. cerevisiae) (Mak16) | 0.49 |
| *Mamdc2* | NM_174857.3 | MAM domain containing 2 (Mamdc2) | 0.47 |
| *Man2a1* | NM_008549.1 | mannosidase 2, alpha 1 (Man2a1) | 0.48 |
| *Manba* | NM_027288.2 | mannosidase, beta A, lysosomal (Manba) | 0.56 |
| *Map2k2* | NM_023138.3 | mitogen activated protein kinase kinase 2 (Map2k2) | 0.65 |
| *Map2k3* | NM_008928.3 | mitogen activated protein kinase kinase 3 (Map2k3) | 0.45 |
| *Map2k4* | NM_009157.2 | mitogen activated protein kinase kinase 4 (Map2k4) | 0.55 |
| *Map4k4* | NM_008696.2 | mitogen-activated protein kinase kinase kinase kinase 4 (Map4k4) | 0.50 |
| *Mapk1* | NM_011949.3 | mitogen-activated protein kinase 1 (Mapk1), transcript variant 1 | 0.64 |
| *Mapk6* | NM_027418.1 | mitogen-activated protein kinase 6 (Mapk6), transcript variant 2 | 0.53 |
| *Marcks* | NM_008538.2 | myristoylated alanine rich protein kinase C substrate (Marcks) | 0.60 |
| *Marcksl1* | NM_010807.3 | MARCKS-like 1 (Marcksl1) | 0.64 |
| *Mars2* | NM_175439.3 | methionine-tRNA synthetase 2 (mitochondrial) (Mars2), nuclear gene encoding mitochondrial protein | 0.62 |
| *Mast2* | NM_008641.2 | microtubule associated serine/threonine kinase 2 (Mast2), transcript variant 2 | 0.66 |
| *Mbd3* | NM_013595.2 | methyl-CpG binding domain protein 3 (Mbd3) | 0.62 |
| *Mbnl1* | NM_020007.3 | muscleblind-like 1 (Drosophila) (Mbnl1) | 0.54 |
| *Mboat1* | NM_153546.3 | membrane bound O-acyltransferase domain containing 1 (Mboat1) | 0.63 |
| *Mcat* | NM_001030014.2 | malonyl CoA:ACP acyltransferase (mitochondrial) (Mcat), nuclear gene encoding mitochondrial protein | 0.66 |
| *Mcc* | NM_001033406.1 | mutated in colorectal cancers (Mcc) | 0.66 |
| *Mcfd2* | NM_139295.2 | multiple coagulation factor deficiency 2 (Mcfd2) | 0.58 |
| *Mcm10* | NM_027290.1 | minichromosome maintenance deficient 10 (S. cerevisiae) (Mcm10) | 0.23 |
| *Mcm2* | NM_008564.1 | minichromosome maintenance deficient 2 mitotin (S. cerevisiae) (Mcm2) | 0.29 |
| *Mcm3* | NM_008563.2 | minichromosome maintenance deficient 3 (S. cerevisiae) (Mcm3) | 0.53 |
| *Mcm4* | NM_008565.2 | minichromosome maintenance deficient 4 homolog (S. cerevisiae) (Mcm4) | 0.28 |
| *Mcm5* | NM_008566.2 | minichromosome maintenance deficient 5, cell division cycle 46 (S. cerevisiae) (Mcm5) | 0.14 |
| *Mcm6* | NM_008567.1 | minichromosome maintenance deficient 6 (MIS5 homolog, S. pombe) (S. cerevisiae) (Mcm6) | 0.25 |
| *Mcm7* | NM_008568.2 | minichromosome maintenance deficient 7 (S. cerevisiae) (Mcm7) | 0.48 |
| *Mdga2* | NM_207010.1 | MAM domain containing glycosylphosphatidylinositol anchor 2 (Mdga2) | 0.51 |
| *Mdh2* | NM_008617.2 | malate dehydrogenase 2, NAD (mitochondrial) (Mdh2), nuclear gene encoding mitochondrial protein | 0.40 |
| *Med10* | NM_138596.1 | mediator of RNA polymerase II transcription, subunit 10 homolog (NUT2, S. cerevisiae) (Med10) | 0.55 |
| *Mef2c* | NM_025282.2 | myocyte enhancer factor 2C (Mef2c) | 0.58 |
| *Mela* | NM_008581 | | 0.20 |
| *Melk* | NM_010790.2 | maternal embryonic leucine zipper kinase (Melk) | 0.66 |

| | | | |
|---|---|---|---|
| *Mertk* | NM_008587.1 | c-mer proto-oncogene tyrosine kinase (Mertk) | 0.63 |
| *Mest* | NM_008590.1 | mesoderm specific transcript (Mest) | 0.14 |
| *Metap1* | NM_175224.4 | methionyl aminopeptidase 1 (Metap1) | 0.64 |
| *Metap2* | NM_019648.3 | methionine aminopeptidase 2 (Metap2) | 0.66 |
| *Metrn* | NM_133719 | | 0.39 |
| *Mex3a* | NM_001029890.1 | mex3 homolog A (C. elegans) (Mex3a) | 0.49 |
| *Mfap3* | NM_145426.2 | microfibrillar-associated protein 3 (Mfap3), transcript variant 1 | 0.63 |
| *Mfge8* | NM_001045489.1 | milk fat globule-EGF factor 8 protein (Mfge8), transcript variant 2 | 0.42 |
| *Mfhas1* | NM_001081279.1 | malignant fibrous histiocytoma amplified sequence 1 (Mfhas1) | 0.42 |
| *Mki67* | XM_001000692.2 | PREDICTED: antigen identified by monoclonal antibody Ki 67 (Mki67) | 0.15 |
| *Mki67ip* | NM_026472.3 | Mki67 (FHA domain) interacting nucleolar phosphoprotein (Mki67ip) | 0.58 |
| *mKIAA0282* | AK129109 | | 0.17 |
| *Mknk2* | NM_021462.3 | MAP kinase-interacting serine/threonine kinase 2 (Mknk2) | 0.62 |
| *Mllt11* | NM_019914.3 | myeloid/lymphoid or mixed-lineage leukemia (trithorax homolog, Drosophila); translocated to, 11 (Mllt11) | 0.39 |
| *Mllt4* | NM_010806.1 | myeloid/lymphoid or mixed-lineage leukemia (trithorax homolog, Drosophila); translocated to, 4 (Mllt4) | 0.61 |
| *Mmp15* | NM_008609.3 | matrix metallopeptidase 15 (Mmp15) | 0.32 |
| *Mmp17* | NM_011846.4 | matrix metallopeptidase 17 (Mmp17) | 0.55 |
| *Mod1* | NM_008615.1 | malic enzyme, supernatant (Mod1) | 0.49 |
| *Morc2a* | NM_198162.1 | microrchidia 2A (Morc2a) | 0.65 |
| *Mphosph6* | NM_026758.3 | M phase phosphoprotein 6 (Mphosph6) | 0.53 |
| *Mphosph9* | NM_001081323.1 | M-phase phosphoprotein 9 (Mphosph9) | 0.46 |
| *Mpi* | NM_025837.2 | mannose phosphate isomerase (Mpi) | 0.64 |
| *Mpnd* | NM_026530.5 | MPN domain containing (Mpnd) | 0.66 |
| *Mre11a* | NM_018736.2 | meiotic recombination 11 homolog A (S. cerevisiae) (Mre11a) | 0.59 |
| *Mrpl12* | NM_027204.2 | mitochondrial ribosomal protein L12 (Mrpl12), nuclear gene encoding mitochondrial protein | 0.46 |
| *Mrpl15* | AK017804 | | 0.56 |
| *Mrpl17* | NM_025301.1 | | 0.55 |
| *Mrpl18* | NM_026310.3 | mitochondrial ribosomal protein L18 (Mrpl18), nuclear gene encoding mitochondrial protein | 0.64 |
| *Mrpl20* | NM_025570.2 | mitochondrial ribosomal protein L20 (Mrpl20), nuclear gene encoding mitochondrial protein | 0.58 |
| *Mrpl22* | NM_175001.2 | mitochondrial ribosomal protein L22 (Mrpl22), nuclear gene encoding mitochondrial protein | 0.61 |
| *Mrpl3* | NM_053159.3 | mitochondrial ribosomal protein L3 (Mrpl3), nuclear gene encoding mitochondrial protein | 0.62 |
| *Mrpl30* | NM_027098.2 | mitochondrial ribosomal protein L30 (Mrpl30), nuclear gene encoding mitochondrial protein | 0.59 |
| *Mrpl34* | NM_053162.2 | mitochondrial ribosomal protein L34 (Mrpl34), nuclear gene encoding mitochondrial protein | 0.59 |
| *Mrpl37* | NM_025500.1 | mitochondrial ribosomal protein L37 (Mrpl37), nuclear gene encoding mitochondrial protein | 0.63 |
| *Mrpl38* | NM_024177.3 | mitochondrial ribosomal protein L38 (Mrpl38), nuclear gene encoding mitochondrial protein | 0.61 |
| *Mrpl43* | NM_053164.3 | mitochondrial ribosomal protein L43 (Mrpl43), nuclear gene encoding mitochondrial protein | 0.62 |
| *Mrpl52* | NM_026851.2 | mitochondrial ribosomal protein L52 (Mrpl52), nuclear gene encoding mitochondrial protein | 0.65 |
| *Mrpl53* | NM_026744.3 | mitochondrial ribosomal protein L53 (Mrpl53), nuclear gene encoding mitochondrial protein | 0.46 |
| *Mrpl54* | NM_025317.2 | mitochondrial ribosomal protein L54 (Mrpl54), nuclear gene encoding mitochondrial protein | 0.60 |
| *Mrps10* | NM_183086 | | 0.53 |
| *Mrps16* | NM_025440.2 | mitochondrial ribosomal protein S16 (Mrps16), nuclear gene encoding mitochondrial protein | 0.64 |
| *Mrps18b* | NM_025878.1 | mitochondrial ribosomal protein S18B (Mrps18b), nuclear gene encoding mitochondrial protein | 0.58 |
| *Mrps2* | NM_080452.2 | mitochondrial ribosomal protein S2 (Mrps2), nuclear gene encoding mitochondrial protein | 0.64 |
| *Mrps28* | NM_025434.2 | mitochondrial ribosomal protein S28 (Mrps28), nuclear gene encoding mitochondrial protein | 0.58 |
| *Mrps30* | NM_021556.3 | mitochondrial ribosomal protein S30 (Mrps30), nuclear gene encoding mitochondrial protein | 0.63 |
| *Mrps35* | NM_145573.2 | mitochondrial ribosomal protein S35 (Mrps35), nuclear gene encoding mitochondrial protein | 0.62 |
| *Mrps7* | NM_025305.1 | mitchondrial ribosomal protein S7 (Mrps7) | 0.47 |
| *Mrto4* | NM_023536.2 | MRT4, mRNA turnover 4, homolog (S. cerevisiae) (Mrto4) | 0.52 |
| *Msl2l1* | XM_150227.8 | PREDICTED: male-specific lethal 2-like 1 (Drosophila) (Msl2l1) | 0.58 |

| | | | |
|---|---|---|---|
| *Msto1* | NM_144898.1 | misato homolog 1 (Drosophila) (Msto1) | 0.64 |
| *Mta2* | NM_011842.3 | metastasis-associated gene family, member 2 (Mta2) | 0.61 |
| *Mtap* | NM_024433.1 | methylthioadenosine phosphorylase (Mtap) | 0.49 |
| *Mtch2* | NM_019758.2 | mitochondrial carrier homolog 2 (C. elegans) (Mtch2), nuclear gene encoding mitochondrial protein | 0.66 |
| *mt-Co2* | | | 0.63 |
| *Mtdh* | NM_026002.4 | Metadherin (Mtdh) | 0.48 |
| *mtDNA_ATP8* | | | 0.64 |
| *Mthfd2* | NM_008638 | | 0.22 |
| *Mtmr11* | NM_181409.2 | myotubularin related protein 11 (Mtmr11) | 0.51 |
| *Mtmr12* | NM_172958.3 | myotubularin related protein 12 (Mtmr12) | 0.56 |
| *Mtmr2* | NM_023858.1 | myotubularin related protein 2 (Mtmr2) | 0.52 |
| *Mvd* | NM_138656.1 | mevalonate (diphospho) decarboxylase (Mvd) | 0.56 |
| *Mxra8* | NM_024263.3 | matrix-remodelling associated 8 (Mxra8) | 0.60 |
| *Mybbp1a* | NM_016776.2 | MYB binding protein (P160) 1a (Mybbp1a) | 0.56 |
| *Mybl2* | NM_008652.2 | myeloblastosis oncogene-like 2 (Mybl2) | 0.46 |
| *Myef2* | NM_010852.1 | myelin basic protein expression factor 2, repressor (Myef2) | 0.49 |
| *Myh9* | NM_022410.1 | | 0.56 |
| *Mylk* | NM_139300.3 | myosin, light polypeptide kinase (Mylk) | 0.22 |
| *Myo1b* | NM_010863.2 | myosin IB (Myo1b) | 0.55 |
| *Myo5a* | NM_010864.2 | myosin Va (Myo5a) | 0.44 |
| *Myo6* | NM_001039546.1 | myosin VI (Myo6) | 0.49 |
| *N4wbp5-pending* | AK050560 | | 0.64 |
| *N6amt1* | NM_026366.1 | N-6 adenine-specific DNA methyltransferase 1 (putative) (N6amt1) | 0.60 |
| *N6amt2* | NM_026526.2 | N-6 adenine-specific DNA methyltransferase 2 (putative) (N6amt2) | 0.51 |
| *Nab1* | NM_008667.2 | Ngfi-A binding protein 1 (Nab1) | 0.52 |
| *Naca* | NM_013608.2 | nascent polypeptide-associated complex alpha polypeptide (Naca) | 0.50 |
| *Nadk* | NM_138671.1 | NAD kinase (Nadk) | 0.66 |
| *Nans* | NM_053179.3 | N-acetylneuraminic acid synthase (sialic acid synthase) (Nans) | 0.43 |
| *Nap1l1* | NM_015781.3 | nucleosome assembly protein 1-like 1 (Nap1l1) | 0.35 |
| *Nap1l4* | NM_008672 | | 0.65 |
| *Nap1l5* | NM_021432.2 | nucleosome assembly protein 1-like 5 (Nap1l5) | 0.59 |
| *Narg1* | NM_053089.2 | NMDA receptor-regulated gene 1 (Narg1) | 0.46 |
| *Nasp* | NM_016777.3 | nuclear autoantigenic sperm protein (histone-binding) (Nasp), transcript variant 2 | 0.53 |
| *Nat5* | NM_026425.1 | N-acetyltransferase 5 (ARD1 homolog, S. cerevisiae) (Nat5) | 0.56 |
| *Nav1* | NM_173437 | | 0.41 |
| *Ncam1* | NM_010875.3 | neural cell adhesion molecule 1 (Ncam1), transcript variant 2 | 0.40 |
| *Ncan* | NM_007789.2 | neurocan (Ncan) | 0.13 |
| *Ncapd2* | NM_146171.1 | non-SMC condensin I complex, subunit D2 (Ncapd2) | 0.24 |
| *Ncapd3* | NM_178113.2 | non-SMC condensin II complex, subunit D3 (Ncapd3) | 0.45 |
| *Ncapg2* | NM_133762.3 | non-SMC condensin II complex, subunit G2 (Ncapg2) | 0.62 |
| *Ncaph* | NM_144818.1 | non-SMC condensin I complex, subunit H (Ncaph) | 0.16 |
| *Ncdn* | NM_011986.2 | neurochondrin (Ncdn) | 0.60 |
| *Ncstn* | NM_021607.2 | nicastrin (Ncstn) | 0.58 |
| *Ndc80* | NM_023294.1 | NDC80 homolog, kinetochore complex component (S. cerevisiae) (Ndc80) | 0.42 |
| *Ndfip1* | NM_022996.1 | Nedd4 family interacting protein 1 (Ndfip1) | 0.65 |
| *Ndufa12* | NM_025551.3 | NADH dehydrogenase (ubiquinone) 1 alpha subcomplex, 12 (Ndufa12) | 0.64 |
| *Ndufa12l* | XM_001476048.1 | PREDICTED: Ndufa12-like (Ndufa12l) | 0.66 |
| *Ndufa4* | NM_010886.1 | NADH dehydrogenase (ubiquinone) 1 alpha subcomplex, 4 (Ndufa4) | 0.63 |
| *Ndufa5* | NM_026614.2 | NADH dehydrogenase (ubiquinone) 1 alpha subcomplex, 5 (Ndufa5), nuclear gene encoding mitochondrial protein | 0.64 |
| *Ndufb6* | NM_001033305.1 | NADH dehydrogenase (ubiquinone) 1 beta subcomplex, 6 (Ndufb6) | 0.52 |
| *Ndufb8* | NM_026061.2 | NADH dehydrogenase (ubiquinone) 1 beta subcomplex 8 (Ndufb8) | 0.62 |
| *Ndufc1* | NM_025523.1 | NADH dehydrogenase (ubiquinone) 1, subcomplex unknown, 1 (Ndufc1) | 0.56 |
| *Ndufc2* | NM_024220.1 | NADH dehydrogenase (ubiquinone) 1, subcomplex unknown, 2 (Ndufc2) | 0.51 |
| *Ndufs2* | NM_153064.4 | NADH dehydrogenase (ubiquinone) Fe-S protein 2 (Ndufs2) | 0.58 |
| *Ndufs4* | NM_010887.1 | NADH dehydrogenase (ubiquinone) Fe-S protein 4 (Ndufs4), nuclear gene encoding mitochondrial protein | 0.59 |
| *Ndufv2* | NM_028388.1 | NADH dehydrogenase (ubiquinone) flavoprotein 2 (Ndufv2) | 0.59 |
| *Necap1* | NM_026267.1 | NECAP endocytosis associated 1 (Necap1) | 0.62 |
| *Nedd1* | NM_008682.1 | neural precursor cell expressed, developmentally down-regulated gene 1 (Nedd1) | 0.62 |
| *Nek3* | NM_011848.2 | NIMA (never in mitosis gene a)-related expressed kinase 3 (Nek3) | 0.60 |
| *Neo1* | NM_008684.1 | | 0.53 |
| *Neto2* | NM_001081324.1 | neuropilin (NRP) and tolloid (TLL)-like 2 (Neto2) | 0.40 |
| *Neurl* | NM_021360 | | 0.21 |
| *Nfib* | NM_008687.2 | nuclear factor I/B (Nfib) | 0.56 |
| *Nhsl1* | NM_173390.3 | NHS-like 1 (Nhsl1) | 0.63 |
| *Nip7* | NM_025391.1 | nuclear import 7 homolog (S. cerevisiae) (Nip7) | 0.64 |
| *Nipsnap3a* | NM_025623.2 | nipsnap homolog 3A (C. elegans) (Nipsnap3a) | 0.54 |

| | | | |
|---|---|---|---|
| *Nkrf* | NM_029891.2 | NF-kappaB repressing factor (Nkrf) | 0.57 |
| *Nkx2-2* | NM_010919.2 | NK2 transcription factor related, locus 2 (Drosophila) (Nkx2-2), transcript variant 1 | 0.30 |
| *Nme1* | NM_008704.2 | non-metastatic cells 1, protein (NM23A) expressed in (Nme1) | 0.43 |
| *Nme2* | NM_008705.4 | non-metastatic cells 2, protein (NM23B) expressed in (Nme2), transcript variant 1 | 0.39 |
| *Nme4* | NM_019731.1 | non-metastatic cells 4, protein expressed in (Nme4), nuclear gene encoding mitochondrial protein | 0.57 |
| *Nmral1* | NM_026393.1 | NmrA-like family domain containing 1 (Nmral1) | 0.58 |
| *Nmt1* | NM_008707.3 | N-myristoyltransferase 1 (Nmt1) | 0.63 |
| *Nob1* | NM_026277.1 | NIN1/RPN12 binding protein 1 homolog (S. cerevisiae) (Nob1) | 0.62 |
| *Noc2l* | NM_021303.2 | nucleolar complex associated 2 homolog (S. cerevisiae) (Noc2l) | 0.57 |
| *Noc4l* | NM_153570.2 | nucleolar complex associated 4 homolog (S. cerevisiae) (Noc4l) | 0.57 |
| *Nol1* | NM_138747.1 | nucleolar protein 1 (Nol1) | 0.59 |
| *Nol10* | NM_001008421.1 | nucleolar protein 10 (Nol10) | 0.63 |
| *Nol5* | NM_018868.2 | nucleolar protein 5 (Nol5) | 0.24 |
| *Nol5a* | NM_024193.2 | nucleolar protein 5A (Nol5a) | 0.47 |
| *Nola1* | NM_026578.2 | nucleolar protein family A, member 1 (H/ACA small nucleolar RNPs) (Nola1) | 0.40 |
| *Nola2* | NM_026631.3 | nucleolar protein family A, member 2 (Nola2) | 0.49 |
| *Nola3* | NM_025403.2 | nucleolar protein family A, member 3 (Nola3) | 0.48 |
| *Nomo1* | NM_153057.3 | nodal modulator 1 (Nomo1) | 0.39 |
| *Npc2* | NM_023409.4 | Niemann Pick type C2 (Npc2) | 0.66 |
| *Npm1* | NM_008722.1 | nucleophosmin 1 (Npm1) | 0.41 |
| *Npm3* | NM_008723.1 | nucleoplasmin 3 (Npm3) | 0.25 |
| *Npm3-ps1* | NR_002702.1 | nucleoplasmin 3, pseudogene 1 (Npm3-ps1), non-coding RNA. | 0.25 |
| *Nptn* | NM_009145.1 | neuroplastin (Nptn) | 0.45 |
| *Nptx1* | NM_008730.2 | neuronal pentraxin 1 (Nptx1) | 0.22 |
| *Nqo2* | NM_020282.2 | NAD(P)H dehydrogenase, quinone 2 (Nqo2) | 0.64 |
| *Nr2c2ap* | NM_001025587.1 | nuclear receptor 2C2-associated protein (Nr2c2ap), transcript variant 2 | 0.66 |
| *Nrarp* | NM_025980.2 | Notch-regulated ankyrin repeat protein (Nrarp) | 0.43 |
| *Nrm* | NM_134122.2 | nurim (nuclear envelope membrane protein) (Nrm) | 0.20 |
| *Nrn1* | NM_153529.1 | neuritin 1 (Nrn1) | 0.42 |
| *Nrp1* | NM_008737.1 | neuropilin 1 (Nrp1) | 0.35 |
| *Nsbp1* | NM_016710.2 | nucleosome binding protein 1 (Nsbp1) | 0.51 |
| *Nsdhl* | NM_010941.3 | NAD(P) dependent steroid dehydrogenase-like (Nsdhl) | 0.53 |
| *Nsep1* | NM_011732 | | 0.41 |
| *Nt5dc2* | NM_027289.1 | 5'-nucleotidase domain containing 2 (Nt5dc2) | 0.50 |
| *Nubp1* | NM_011955.1 | nucleotide binding protein 1 (Nubp1) | 0.48 |
| *Nudcd2* | NM_026023.4 | NudC domain containing 2 (Nudcd2) | 0.58 |
| *Nudt21* | NM_026623.3 | nudix (nucleoside diphosphate linked moiety X)-type motif 21 (Nudt21) | 0.50 |
| *Nudt5* | NM_016918.3 | nudix (nucleoside diphosphate linked moiety X)-type motif 5 (Nudt5) | 0.50 |
| *Nup133* | NM_172288.1 | nucleoporin 133 (Nup133) | 0.64 |
| *Nup160* | NM_021512.2 | nucleoporin 160 (Nup160) | 0.51 |
| *Nup205* | XM_133073.7 | PREDICTED: nucleoporin 205, transcript variant 1 (Nup205) | 0.39 |
| *Nup210* | NM_018815.1 | nucleoporin 210 (Nup210) | 0.17 |
| *Nup43* | NM_145706.1 | nucleoporin 43 (Nup43) | 0.48 |
| *Nup62* | NM_053074.1 | nucleoporin 62 (Nup62) | 0.36 |
| *Nup85* | NM_001002929.3 | nucleoporin 85 (Nup85) | 0.54 |
| *Nup88* | NM_172394.2 | nucleoporin 88 (Nup88), transcript variant 1 | 0.50 |
| *Nup93* | NM_172410 | | 0.66 |
| *Nus1* | NM_030250.1 | nuclear undecaprenyl pyrophosphate synthase 1 homolog (S. cerevisiae) (Nus1) | 0.57 |
| *Nusap1* | NM_001042652.1 | nucleolar and spindle associated protein 1 (Nusap1), transcript variant 2 | 0.20 |
| *Nutf2* | NM_026532.3 | nuclear transport factor 2 (Nutf2) | 0.59 |
| *Nxph1* | NM_008751.2 | neurexophilin 1 (Nxph1) | 0.15 |
| *Nxt1* | NM_019761.4 | NTF2-related export protein 1 (Nxt1) | 0.63 |
| *Ociad2* | NM_026950.3 | OCIA domain containing 2 (Ociad2) | 0.40 |
| *Ogdh* | NM_010956.3 | oxoglutarate dehydrogenase (lipoamide) (Ogdh), nuclear gene encoding mitochondrial protein | 0.59 |
| *Ola1* | NM_025942.2 | Obg-like ATPase 1 (Ola1), transcript variant 1 | 0.52 |
| *Olfm2* | NM_173777.2 | olfactomedin 2 (Olfm2) | 0.39 |
| *Olfml2b* | NM_177068.3 | olfactomedin-like 2B (Olfml2b) | 0.28 |
| *Olig1* | NM_016968.4 | oligodendrocyte transcription factor 1 (Olig1) | 0.24 |
| *Omg* | NM_019409.1 | oligodendrocyte myelin glycoprotein (Omg) | 0.55 |
| *Opn3* | NM_010098.2 | opsin 3 (Opn3) | 0.50 |
| *Oprs1* | NM_011014.2 | opioid receptor, sigma 1 (Oprs1) | 0.56 |
| *Orai1* | NM_175423.3 | ORAI calcium release-activated calcium modulator 1 (Orai1) | 0.63 |
| *Orc5l* | NM_011959.2 | origin recognition complex, subunit 5-like (S. cerevisiae) (Orc5l) | 0.53 |
| *Orc6l* | NM_019716.1 | origin recognition complex, subunit 6-like (S. cerevisiae) (Orc6l) | 0.47 |
| *Osbpl6* | NM_145525.2 | oxysterol binding protein-like 6 (Osbpl6) | 0.27 |
| *Osbpl8* | NM_175489.3 | oxysterol binding protein-like 8 (Osbpl8), transcript variant 1 | 0.58 |

| | | | |
|---|---|---|---|
| *Ostm1* | NM_172416.2 | osteopetrosis associated transmembrane protein 1 (Ostm1) | 0.51 |
| *Oxct1* | NM_024188.5 | 3-oxoacid CoA transferase 1 (Oxct1) | 0.63 |
| *Oxr1* | NM_130885.2 | oxidation resistance 1 (Oxr1), transcript variant 1 | 0.59 |
| *P2ry5* | NM_175116.2 | purinergic receptor P2Y, G-protein coupled, 5 (P2ry5) | 0.32 |
| *P4hb* | NM_011032.2 | prolyl 4-hydroxylase, beta polypeptide (P4hb) | 0.54 |
| *Pa2g4* | NM_011119.3 | proliferation-associated 2G4 (Pa2g4) | 0.42 |
| *Pabpc1* | NM_008774.2 | poly A binding protein, cytoplasmic 1 (Pabpc1) | 0.60 |
| *Pabpc4* | NM_130881.2 | poly A binding protein, cytoplasmic 4 (Pabpc4), transcript variant 1 | 0.46 |
| *Pabpn1* | NM_019402.1 | poly(A) binding protein, nuclear 1 (Pabpn1) | 0.52 |
| *Pafah1b1* | NM_013625.1 | platelet-activating factor acetylhydrolase, isoform 1b, beta1 subunit (Pafah1b1) | 0.58 |
| *Pafah1b3* | NM_008776.1 | platelet-activating factor acetylhydrolase, isoform 1b, alpha1 subunit (Pafah1b3) | 0.62 |
| *Palmd* | NM_023245.3 | palmdelphin (Palmd) | 0.38 |
| *Pank3* | NM_145962.2 | pantothenate kinase 3 (Pank3) | 0.54 |
| *Papola* | NM_011112.2 | poly (A) polymerase alpha (Papola) | 0.61 |
| *Parp1* | NM_007415.2 | poly (ADP-ribose) polymerase family, member 1 (Parp1) | 0.50 |
| *Pbk* | NM_023209.1 | PDZ binding kinase (Pbk) | 0.08 |
| *Pcbp1* | NM_011865.3 | poly(rC) binding protein 1 (Pcbp1) | 0.55 |
| *Pcdh17* | NM_001013753.1 | protocadherin 17 (Pcdh17) | 0.33 |
| *Pcdh7* | NM_018764.1 | protocadherin 7 (Pcdh7) | 0.44 |
| *Pcdh9* | NM_001081377.1 | protocadherin 9 (Pcdh9) | 0.45 |
| *Pcp4l1* | XM_484933.5 | PREDICTED: Purkinje cell protein 4-like 1 (Pcp4l1) | 0.64 |
| *Pctk1* | NM_011049.4 | PCTAIRE-motif protein kinase 1 (Pctk1) | 0.64 |
| *Pdap1* | NM_001033313.3 | PDGFA associated protein 1 (Pdap1) | 0.28 |
| *Pdcd2l* | NM_026549.3 | programmed cell death 2-like (Pdcd2l) | 0.65 |
| *Pde1b* | NM_008800 | | 0.66 |
| *Pdgfa* | NM_008808 | | 0.53 |
| *Pdha1* | NM_008810.2 | pyruvate dehydrogenase E1 alpha 1 (Pdha1), nuclear gene encoding mitochondrial protein | 0.65 |
| *Pdhb* | NM_024221.3 | pyruvate dehydrogenase (lipoamide) beta (Pdhb) | 0.56 |
| *Pdia3* | NM_007952.2 | protein disulfide isomerase associated 3 (Pdia3) | 0.57 |
| *Pdia5* | NM_028295.1 | protein disulfide isomerase associated 5 (Pdia5) | 0.41 |
| *Pdk3* | NM_145630.2 | pyruvate dehydrogenase kinase, isoenzyme 3 (Pdk3) | 0.64 |
| *Pdlim4* | NM_019417.2 | PDZ and LIM domain 4 (Pdlim4) | 0.53 |
| *Pdlim5* | NM_019808.1 | PDZ and LIM domain 5 (Pdlim5), transcript variant 1 | 0.57 |
| *Pdss1* | NM_019501.3 | prenyl (solanesyl) diphosphate synthase, subunit 1 (Pdss1) | 0.48 |
| *Pdxp* | NM_020271.3 | pyridoxal (pyridoxine, vitamin B6) phosphatase (Pdxp) | 0.53 |
| *Pdzrn4* | XM_001481302.1 | PREDICTED: PDZ domain containing RING finger 4 (Pdzrn4) | 0.33 |
| *Peli1* | NM_023324.2 | pellino 1 (Peli1) | 0.49 |
| *Pfdn4* | NM_001013369.1 | prefoldin 4 (Pfdn4), transcript variant 2 | 0.32 |
| *Pfn1* | NM_011072.2 | profilin 1 (Pfn1) | 0.52 |
| *Pgam1* | NM_023418.2 | phosphoglycerate mutase 1 (Pgam1) | 0.57 |
| *Pgam5* | NM_028273.1 | phosphoglycerate mutase family member 5 (Pgam5) | 0.44 |
| *Pgls* | NM_025396.3 | 6-phosphogluconolactonase (Pgls) | 0.57 |
| *Pgp* | NM_025954.3 | phosphoglycolate phosphatase (Pgp) | 0.29 |
| *Phb2* | NM_007531.2 | prohibitin 2 (Phb2) | 0.49 |
| *Phc2* | NM_018774.3 | polyhomeotic-like 2 (Drosophila) (Phc2) | 0.47 |
| *Phf20* | NM_172674.2 | PHD finger protein 20 (Phf20) | 0.50 |
| *Phf5a* | NM_026737.3 | PHD finger protein 5A (Phf5a) | 0.60 |
| *Phf6* | NM_027642.1 | PHD finger protein 6 (Phf6) | 0.54 |
| *Phlda1* | NM_009344.1 | pleckstrin homology-like domain, family A, member 1 (Phlda1) | 0.34 |
| *Phtf2* | NM_172992.2 | putative homeodomain transcription factor 2 (Phtf2) | 0.58 |
| *Pi4ka* | NM_001001983.1 | phosphatidylinositol 4-kinase, catalytic, alpha polypeptide (Pi4ka) | 0.65 |
| *Pif1* | NM_172453.1 | PIF1 5'-to-3' DNA helicase homolog (S. cerevisiae) (Pif1) | 0.37 |
| *Piga* | NM_011081.2 | phosphatidylinositol glycan anchor biosynthesis, class A (Piga) | 0.61 |
| *Pip4k2a* | NM_008845.4 | phosphatidylinositol-5-phosphate 4-kinase, type II, alpha (Pip4k2a) | 0.66 |
| *Pip4k2b* | NM_054051.1 | phosphatidylinositol-5-phosphate 4-kinase, type II, beta (Pip4k2b) | 0.53 |
| *Pitrm1* | NM_145131.1 | pitrilysin metallepetidase 1 (Pitrm1) | 0.61 |
| *Pja1* | NM_008853.2 | praja1, RING-H2 motif containing (Pja1) | 0.57 |
| *Pja2* | NM_144859.2 | praja 2, RING-H2 motif containing (Pja2), transcript variant 2 | 0.61 |
| *Pkia* | NM_008862.3 | protein kinase inhibitor, alpha (Pkia) | 0.48 |
| *Pkm2* | XM_131934.4 | | 0.50 |
| *Pkn2* | NM_178654.3 | protein kinase N2 (Pkn2) | 0.63 |
| *Plaa* | NM_172695.2 | phospholipase A2, activating protein (Plaa) | 0.53 |
| *Pld1* | XM_130807.1 | | 0.51 |
| *Plekha1* | NM_133942.2 | pleckstrin homology domain containing, family A (phosphoinositide binding specific) member 1 (Plekha1) | 0.63 |
| *Plekha8* | NM_001001335.1 | pleckstrin homology domain containing, family A (phosphoinositide binding specific) member 8 (Plekha8) | 0.63 |
| *Plekhk1* | NM_133244.2 | | 0.23 |

| | | | |
|---|---|---|---|
| *Plk1* | NM_011121.3 | polo-like kinase 1 (Drosophila) (Plk1) | 0.11 |
| *Plk2* | NM_152804.1 | polo-like kinase 2 (Drosophila) (Plk2) | 0.29 |
| *Plk4* | NM_011495.1 | | 0.57 |
| *Plvap* | NM_032398.1 | plasmalemma vesicle associated protein (Plvap) | 0.13 |
| *Plxnd1* | NM_026376.3 | plexin D1 (Plxnd1) | 0.54 |
| *Pmp22* | NM_008885.2 | peripheral myelin protein 22 (Pmp22) | 0.51 |
| *Pno1* | NM_025443.2 | partner of NOB1 homolog (S. cerevisiae) (Pno1) | 0.41 |
| *Pola2* | NM_008893.2 | polymerase (DNA directed), alpha 2 (Pola2) | 0.64 |
| *Pold1* | NM_011131.2 | polymerase (DNA directed), delta 1, catalytic subunit (Pold1) | 0.33 |
| *Pold2* | NM_008894.1 | polymerase (DNA directed), delta 2, regulatory subunit (Pold2) | 0.56 |
| *Pold4* | NM_027196 | | 0.61 |
| *Pole* | NM_011132.1 | polymerase (DNA directed), epsilon (Pole) | 0.45 |
| *Pole4* | NM_025882.3 | polymerase (DNA-directed), epsilon 4 (p12 subunit) (Pole4) | 0.60 |
| *Polg* | NM_017462.2 | polymerase (DNA directed), gamma (Polg) | 0.58 |
| *Polr1a* | NM_009088.2 | polymerase (RNA) I polypeptide A (Polr1a) | 0.58 |
| *Polr1d* | NM_009087.1 | polymerase (RNA) I polypeptide D (Polr1d), transcript variant 1 | 0.64 |
| *Polr1e* | NM_022811.2 | polymerase (RNA) I polypeptide E (Polr1e) | 0.60 |
| *Polr2g* | NM_026329.2 | polymerase (RNA) II (DNA directed) polypeptide G (Polr2g) | 0.67 |
| *Polr2h* | NM_145632.1 | polymerase (RNA) II (DNA directed) polypeptide H (Polr2h) | 0.66 |
| *Polr3k* | NM_025901.2 | polymerase (RNA) III (DNA directed) polypeptide K (Polr3k) | 0.60 |
| *Pop5* | NM_026398.2 | processing of precursor 5, ribonuclease P/MRP family (S. cerevisiae) (Pop5) | 0.60 |
| *Pou3f1* | NM_011141.2 | POU domain, class 3, transcription factor 1 (Pou3f1) | 0.19 |
| *Ppa1* | NM_026438.2 | pyrophosphatase (inorganic) 1 (Ppa1) | 0.48 |
| *Ppa2* | NM_146141.1 | pyrophosphatase (inorganic) 2 (Ppa2), nuclear gene encoding mitochondrial protein | 0.61 |
| *Ppan* | NM_145610 | | 0.48 |
| *Ppat* | XM_001002886.2 | PREDICTED: phosphoribosyl pyrophosphate amidotransferase, transcript variant 8 (Ppat) | 0.61 |
| *Ppia* | NM_008907.1 | peptidylprolyl isomerase A (Ppia) | 0.54 |
| *Ppic* | NM_008908.1 | peptidylprolyl isomerase C (Ppic) | 0.35 |
| *Ppid* | NM_026352.2 | | 0.61 |
| *Ppif* | NM_134084.1 | peptidylprolyl isomerase F (cyclophilin F) (Ppif), nuclear gene encoding mitochondrial protein | 0.47 |
| *Ppih* | NM_028677 | | 0.51 |
| *Ppm1b* | NM_011151.1 | protein phosphatase 1B, magnesium dependent, beta isoform (Ppm1b) | 0.64 |
| *Ppm1g* | NM_008014.3 | protein phosphatase 1G (formerly 2C), magnesium-dependent, gamma isoform (Ppm1g) | 0.62 |
| *Ppp1ca* | NM_031868.2 | protein phosphatase 1, catalytic subunit, alpha isoform (Ppp1ca) | 0.65 |
| *Ppp1r10* | NM_175934.2 | protein phosphatase 1, regulatory subunit 10 (Ppp1r10) | 0.62 |
| *Ppp1r12a* | NM_027892.2 | protein phosphatase 1, regulatory (inhibitor) subunit 12A (Ppp1r12a) | 0.44 |
| *Ppp1r14b* | NM_008889.1 | | 0.54 |
| *Ppp1r8* | NM_146154.1 | protein phosphatase 1, regulatory (inhibitor) subunit 8 (Ppp1r8) | 0.65 |
| *Ppp1r9b* | NM_172261.2 | protein phosphatase 1, regulatory subunit 9B (Ppp1r9b) | 0.60 |
| *Ppp2cb* | NM_017374.3 | protein phosphatase 2 (formerly 2A), catalytic subunit, beta isoform (Ppp2cb) | 0.59 |
| *Ppp2r2d* | NM_026391.2 | protein phosphatase 2, regulatory subunit B, delta isoform (Ppp2r2d) | 0.64 |
| *Ppp2r5c* | NM_001081458.1 | protein phosphatase 2, regulatory subunit B (B56), gamma isoform (Ppp2r5c), transcript variant 3 | 0.51 |
| *Ppp3cb* | NM_008914 | | 0.46 |
| *Ppp3r1* | NM_024459.2 | protein phosphatase 3, regulatory subunit B, alpha isoform (calcineurin B, type I) (Ppp3r1) | 0.66 |
| *Ppp4r1* | NM_146081.1 | protein phosphatase 4, regulatory subunit 1 (Ppp4r1) | 0.64 |
| *Ppp6c* | NM_024209.2 | protein phosphatase 6, catalytic subunit (Ppp6c) | 0.57 |
| *Pprc1* | NM_001081214.1 | peroxisome proliferative activated receptor, gamma, coactivator-related 1 (Pprc1) | 0.54 |
| *Prc1* | NM_145150.1 | protein regulator of cytokinesis 1 (Prc1) | 0.06 |
| *Prcp* | NM_028243.2 | prolylcarboxypeptidase (angiotensinase C) (Prcp) | 0.36 |
| *Prdx4* | NM_016764.3 | peroxiredoxin 4 (Prdx4) | 0.63 |
| *Prep* | NM_011156.2 | prolyl endopeptidase (Prep) | 0.48 |
| *Prkaca* | NM_008854.3 | protein kinase, cAMP dependent, catalytic, alpha (Prkaca) | 0.54 |
| *Prkacb* | NM_011100.3 | protein kinase, cAMP dependent, catalytic, beta (Prkacb) | 0.46 |
| *Prkar2b* | NM_011158.3 | protein kinase, cAMP dependent regulatory, type II beta (Prkar2b) | 0.40 |
| *Prkcb* | NM_008855.1 | | 0.15 |
| *Prkcbp1* | NM_027230.3 | protein kinase C binding protein 1 (Prkcbp1) | 0.67 |
| *Prkcsh* | NM_008925.1 | protein kinase C substrate 80K-H (Prkcsh) | 0.56 |
| *Prkd3* | NM_029239.2 | protein kinase D3 (Prkd3) | 0.50 |
| *Prkg1* | NM_011160 | | 0.55 |
| *Prmt3* | NM_133740.1 | protein arginine N-methyltransferase 3 (Prmt3) | 0.54 |

| | | | |
|---|---|---|---|
| *Prmt5* | NM_013768.2 | protein arginine N-methyltransferase 5 (Prmt5) | 0.59 |
| *Prmt7* | NM_145404.1 | protein arginine N-methyltransferase 7 (Prmt7) | 0.47 |
| *Prokr1* | NM_021381.3 | prokineticin receptor 1 (Prokr1) | 0.46 |
| *Prpf19* | NM_134129.2 | PRP19/PSO4 pre-mRNA processing factor 19 homolog (S. cerevisiae) (Prpf19) | 0.57 |
| *Prpf38a* | NM_172697.1 | PRP38 pre-mRNA processing factor 38 (yeast) domain containing A (Prpf38a) | 0.60 |
| *Prpf40a* | NM_018785.2 | PRP40 pre-mRNA processing factor 40 homolog A (yeast) (Prpf40a) | 0.59 |
| *Prr18* | NM_178774.3 | proline rich region 18 (Prr18) | 0.33 |
| *Prrc1* | NM_028447.2 | proline-rich coiled-coil 1 (Prrc1) | 0.63 |
| *Prss35* | NM_178738.2 | protease, serine, 35 (Prss35) | 0.18 |
| *Psip1* | NM_133948.4 | PC4 and SFRS1 interacting protein 1 (Psip1) | 0.64 |
| *Psma5* | NM_011967.2 | proteasome (prosome, macropain) subunit, alpha type 5 (Psma5) | 0.59 |
| *Psma7* | NM_011969.1 | proteasome (prosome, macropain) subunit, alpha type 7 (Psma7) | 0.64 |
| *Psmb10* | NM_013640.2 | proteasome (prosome, macropain) subunit, beta type 10 (Psmb10) | 0.56 |
| *Psmb2* | NM_011970.2 | proteasome (prosome, macropain) subunit, beta type 2 (Psmb2) | 0.62 |
| *Psmb4* | NM_008945.2 | proteasome (prosome, macropain) subunit, beta type 4 (Psmb4) | 0.61 |
| *Psmb7* | NM_011187.1 | proteasome (prosome, macropain) subunit, beta type 7 (Psmb7) | 0.66 |
| *Psmc1* | NM_008947 | | 0.60 |
| *Psmc3ip* | NM_008949.2 | proteasome (prosome, macropain) 26S subunit, ATPase 3, interacting protein (Psmc3ip) | 0.58 |
| *Psmc5* | NM_008950.1 | protease (prosome, macropain) 26S subunit, ATPase 5 (Psmc5) | 0.65 |
| *Psmd1* | NM_027357.2 | proteasome (prosome, macropain) 26S subunit, non-ATPase, 1 (Psmd1) | 0.56 |
| *Psmd12* | NM_025894.1 | proteasome (prosome, macropain) 26S subunit, non-ATPase, 12 (Psmd12) | 0.56 |
| *Psmd14* | NM_021526.1 | proteasome (prosome, macropain) 26S subunit, non-ATPase, 14 (Psmd14) | 0.64 |
| *Psmd8* | NM_026545.2 | proteasome (prosome, macropain) 26S subunit, non-ATPase, 8 (Psmd8) | 0.67 |
| *Psme3* | NM_011192.3 | proteaseome (prosome, macropain) 28 subunit, 3 (Psme3) | 0.59 |
| *Ptcd2* | NM_026873.2 | pentatricopeptide repeat domain 2 (Ptcd2) | 0.66 |
| *Pten* | NM_008960.2 | phosphatase and tensin homolog (Pten) | 0.66 |
| *Ptk2* | NM_007982.1 | PTK2 protein tyrosine kinase 2 (Ptk2) | 0.36 |
| *Ptn* | NM_008973.2 | pleiotrophin (Ptn) | 0.28 |
| *Ptp4a2* | NM_008974.3 | protein tyrosine phosphatase 4a2 (Ptp4a2) | 0.62 |
| *Ptpla* | NM_001012396.2 | protein tyrosine phosphatase-like (proline instead of catalytic arginine), member a (Ptpla), transcript variant 2 | 0.61 |
| *Ptpn12* | NM_011203.2 | protein tyrosine phosphatase, non-receptor type 12 (Ptpn12) | 0.61 |
| *Ptpre* | NM_011212.3 | protein tyrosine phosphatase, receptor type, E (Ptpre) | 0.53 |
| *Ptprm* | NM_008984 | | 0.65 |
| *Ptpro* | NM_011216.2 | protein tyrosine phosphatase, receptor type, O (Ptpro) | 0.28 |
| *Ptprz1* | XM_988289.1 | PREDICTED: protein tyrosine phosphatase, receptor type Z, polypeptide 1, transcript variant 3 (Ptprz1) | 0.19 |
| *Pum2* | NM_030723.1 | pumilio 2 (Drosophila) (Pum2) | 0.64 |
| *Purb* | NM_011221.2 | purine rich element binding protein B (Purb) | 0.64 |
| *Pus7l* | NM_172437.2 | pseudouridylate synthase 7 homolog (S. cerevisiae)-like (Pus7l) | 0.41 |
| *Pusl1* | XM_001476573.1 | PREDICTED: pseudouridylate synthase-like 1, transcript variant 1 (Pusl1) | 0.53 |
| *Pvrl3* | NM_021495.2 | poliovirus receptor-related 3 (Pvrl3), transcript variant alpha | 0.55 |
| *Pycr2* | NM_133705.1 | pyrroline-5-carboxylate reductase family, member 2 (Pycr2) | 0.58 |
| *Qpct* | NM_027455.1 | glutaminyl-peptide cyclotransferase (glutaminyl cyclase) (Qpct) | 0.43 |
| *Qrsl1* | NM_001081054.2 | glutaminyl-tRNA synthase (glutamine-hydrolyzing)-like 1 (Qrsl1) | 0.56 |
| *R3hdm1* | NM_181750.2 | R3H domain 1 (binds single-stranded nucleic acids) (R3hdm1) | 0.67 |
| *Rab26* | XM_283428.6 | PREDICTED: RAB26, member RAS oncogene family, transcript variant 1 (Rab26) | 0.06 |
| *Rab31* | NM_133685.1 | RAB31, member RAS oncogene family (Rab31) | 0.64 |
| *Rab34* | NM_033475.2 | RAB34, member of RAS oncogene family (Rab34) | 0.53 |
| *Rab35* | NM_198163.1 | RAB35, member RAS oncogene family (Rab35) | 0.61 |
| *Rab3a* | NM_009001.3 | RAB3A, member RAS oncogene family (Rab3a) | 0.50 |
| *Rab3b* | NM_023537.4 | | 0.36 |
| *Rab3gap2* | XM_980542.1 | PREDICTED: RAB3 GTPase activating protein subunit 2, transcript variant 4 (Rab3gap2) | 0.55 |
| *Rab5c* | NM_024456.2 | RAB5C, member RAS oncogene family (Rab5c) | 0.65 |
| *Rab8b* | NM_173413.2 | RAB8B, member RAS oncogene family (Rab8b) | 0.43 |
| *Rabggtb* | NM_011231.1 | RAB geranylgeranyl transferase, b subunit (Rabggtb) | 0.53 |
| *Rabif* | NM_145510.1 | RAB interacting factor (Rabif) | 0.63 |
| *Rabl3* | NM_026297.1 | | 0.63 |
| *Rad51* | NM_011234.2 | RAD51 homolog (S. cerevisiae) (Rad51) | 0.56 |
| *Rad51c* | NM_053269.2 | Rad51 homolog c (S. cerevisiae) (Rad51c) | 0.47 |
| *Rad54l* | NM_009015.2 | RAD54 like (S. cerevisiae) (Rad54l) | 0.49 |
| *Raet1b* | NM_009017.1 | retinoic acid early transcript beta (Raet1b) | 0.34 |
| *Raly* | NM_023130.2 | hnRNP-associated with lethal yellow (Raly) | 0.36 |
| *Ramp2* | NM_019444.2 | receptor (calcitonin) activity modifying protein 2 (Ramp2) | 0.55 |
| *Ran* | NM_009391.3 | RAN, member RAS oncogene family (Ran) | 0.59 |
| *Ranbp1* | NM_011239.2 | RAN binding protein 1 (Ranbp1) | 0.39 |

| | | | |
|---|---|---|---|
| *Rangap1* | NM_011241 | | 0.52 |
| *Rangrf* | NM_021329.2 | RAN guanine nucleotide release factor (Rangrf) | 0.54 |
| *Rap1b* | NM_024457.2 | RAS related protein 1b (Rap1b) | 0.52 |
| *Rap1gap* | NM_001081155.1 | Rap1 GTPase-activating protein (Rap1gap) | 0.66 |
| *Rap2c* | NM_172413.2 | RAP2C, member of RAS oncogene family (Rap2c) | 0.27 |
| *Rasa1* | NM_145452.2 | RAS p21 protein activator 1 (Rasa1) | 0.51 |
| *Rasa3* | NM_009025.2 | RAS p21 protein activator 3 (Rasa3) | 0.29 |
| *Rasal2* | NM_177644.4 | RAS protein activator like 2 (Rasal2) | 0.50 |
| *Rasl11b* | NM_026878.1 | RAS-like, family 11, member B (Rasl11b) | 0.66 |
| *Rassf3* | NM_138956.3 | Ras association (RalGDS/AF-6) domain family member 3 (Rassf3) | 0.20 |
| *Rbbp4* | NM_009030.3 | retinoblastoma binding protein 4 (Rbbp4) | 0.60 |
| *Rbbp7* | NM_009031.2 | retinoblastoma binding protein 7 (Rbbp7) | 0.58 |
| *Rbl1* | NM_011249.1 | retinoblastoma-like 1 (p107) (Rbl1) | 0.41 |
| *Rbm13* | NM_026453.1 | RNA binding motif protein 13 (Rbm13) | 0.41 |
| *Rbms3* | NM_178660.2 | RNA binding motif, single stranded interacting protein (Rbms3) | 0.60 |
| *Rbmx* | NM_011252.2 | RNA binding motif protein, X chromosome (Rbmx) | 0.32 |
| *Rbx1* | NM_019712.3 | ring-box 1 (Rbx1) | 0.62 |
| *Rcc1* | NM_133878.2 | regulator of chromosome condensation 1 (Rcc1) | 0.56 |
| *Rcl1* | NM_021525.2 | RNA terminal phosphate cyclase-like 1 (Rcl1) | 0.62 |
| *Rdh11* | NM_021557 | | 0.48 |
| *Reep3* | NM_178606.4 | receptor accessory protein 3 (Reep3) | 0.52 |
| *Reps1* | NM_009048.1 | RalBP1 associated Eps domain containing protein (Reps1) | 0.48 |
| *Rerg* | NM_181988.1 | RAS-like, estrogen-regulated, growth-inhibitor (Rerg) | 0.33 |
| *Rev3l* | NM_011264.3 | REV3-like, catalytic subunit of DNA polymerase zeta RAD54 like (S. cerevisiae) (Rev3l) | 0.44 |
| *Rfc1* | NM_011258.1 | replication factor C (activator 1) 1 (Rfc1) | 0.64 |
| *Rfc2* | NM_020022 | | 0.63 |
| *Rfc3* | XM_132528.3 | | 0.64 |
| *Rfc4* | NM_145480 | | 0.36 |
| *Rfc5* | NM_028128.1 | replication factor C (activator 1) 5 (Rfc5) | 0.53 |
| *Rg9mtd1* | NM_029092.2 | RNA (guanine-9-) methyltransferase domain containing 1 (Rg9mtd1) | 0.66 |
| *Rgs10* | NM_026418.2 | regulator of G-protein signalling 10 (Rgs10) | 0.66 |
| *Rgs7* | NM_011880.2 | regulator of G protein signaling 7 (Rgs7) | 0.26 |
| *Rgs7bp* | NM_029879.2 | regulator of G-protein signalling 7 binding protein (Rgs7bp) | 0.41 |
| *Rhbdf1* | NM_010117.1 | rhomboid family 1 (Drosophila) (Rhbdf1) | 0.55 |
| *Rhobtb1* | NM_001081347.1 | Rho-related BTB domain containing 1 (Rhobtb1) | 0.62 |
| *Rhobtb2* | NM_153514.4 | Rho-related BTB domain containing 2 (Rhobtb2) | 0.66 |
| *Rhoc* | NM_007484.1 | ras homolog gene family, member C (Rhoc) | 0.58 |
| *Rhot1* | NM_021536.6 | ras homolog gene family, member T1 (Rhot1) | 0.65 |
| *Rhot2* | NM_145999.2 | ras homolog gene family, member T2 (Rhot2), nuclear gene encoding mitochondrial protein | 0.63 |
| *Riok3* | NM_024182.4 | RIO kinase 3 (yeast) (Riok3) | 0.66 |
| *Rnaseh2b* | NM_026001.2 | ribonuclease H2, subunit B (Rnaseh2b) | 0.45 |
| *Rnf14* | NM_020012.1 | ring finger protein 14 (Rnf14) | 0.65 |
| *Rnf145* | NM_028862.2 | ring finger protein 145 (Rnf145) | 0.66 |
| *Rnf26* | NM_153762.3 | ring finger protein 26 (Rnf26) | 0.46 |
| *Rnf4* | NM_011278.1 | ring finger protein 4 (Rnf4) | 0.59 |
| *Rnf44* | NM_134064.1 | ring finger protein 44 (Rnf44) | 0.52 |
| *Rnps1* | NM_001080127.1 | ribonucleic acid binding protein S1 (Rnps1), transcript variant 2 | 0.48 |
| *Rnu65* | | | 0.66 |
| *Robo1* | NM_019413.2 | roundabout homolog 1 (Drosophila) (Robo1) | 0.44 |
| *Rock2* | NM_009072.2 | Rho-associated coiled-coil containing protein kinase 2 (Rock2) | 0.62 |
| *Rorb* | NM_001043354.1 | RAR-related orphan receptor beta (Rorb), transcript variant 1 | 0.66 |
| *Rpa3* | NM_026632.3 | replication protein A3 (Rpa3) | 0.43 |
| *Rpl10a* | NM_011287.1 | ribosomal protein L10A (Rpl10a) | 0.45 |
| *Rpl12* | NM_009076.1 | ribosomal protein L12 (Rpl12) | 0.37 |
| *Rpl13a* | NM_009438.3 | ribosomal protein L13a (Rpl13a) | 0.27 |
| *Rpl18* | NM_009077.2 | ribosomal protein L18 (Rpl18) | 0.43 |
| *Rpl18a* | NM_029751.3 | Ribosomal protein L18A (Rpl18a) | 0.47 |
| *Rpl19* | NM_009078.1 | ribosomal protein L19 (Rpl19) | 0.44 |
| *Rpl23* | NM_022891.2 | ribosomal protein L23 (Rpl23) | 0.61 |
| *Rpl24* | NM_024218.2 | ribosomal protein L24 (Rpl24) | 0.65 |
| *Rpl26* | NM_009080.2 | ribosomal protein L26 (Rpl26) | 0.58 |
| *Rpl27* | NM_011289.1 | ribosomal protein L27 (Rpl27) | 0.45 |
| *Rpl27a* | NM_011975.3 | ribosomal protein L27a (Rpl27a) | 0.36 |
| *Rpl28* | NM_009081 | | 0.46 |
| *Rpl29* | NM_009082.2 | ribosomal protein L29 (Rpl29) | 0.46 |
| *Rpl30* | NM_009083.2 | ribosomal protein L30 (Rpl30) | 0.60 |
| *Rpl31* | NM_053257.1 | ribosomal protein L31 (Rpl31) | 0.36 |
| *Rpl32* | NM_172086 | | 0.52 |
| *Rpl34* | NM_026724.1 | ribosomal protein L34 (Rpl34), transcript variant 1 | 0.54 |

| | | | |
|---|---|---|---|
| *Rpl35* | NM_025592.3 | ribosomal protein L35 (Rpl35) | 0.49 |
| *Rpl36a* | NM_019865.2 | ribosomal protein L36a (Rpl36a) | 0.35 |
| *Rpl36al* | NM_025589.1 | ribosomal protein L36a-like (Rpl36al) | 0.45 |
| *Rpl37* | NM_026069.2 | ribosomal protein L37 (Rpl37) | 0.56 |
| *Rpl39* | NM_026055.1 | ribosomal protein L39 (Rpl39) | 0.56 |
| *Rpl4* | NM_024212.2 | ribosomal protein L4 (Rpl4) | 0.33 |
| *Rpl9* | NM_011292.1 | ribosomal protein L9 (Rpl9) | 0.58 |
| *Rplp0* | NM_007475.4 | ribosomal protein, large, P0 (Rplp0) | 0.51 |
| *Rplp1* | NM_018853.3 | ribosomal protein, large, P1 (Rplp1) | 0.57 |
| *Rplp2* | NM_026020.4 | ribosomal protein, large P2 (Rplp2) | 0.47 |
| *Rpn2* | NM_019642.3 | ribophorin II (Rpn2) | 0.54 |
| *Rprm* | NM_023396.4 | reprimo, TP53 dependent G2 arrest mediator candidate (Rprm) | 0.46 |
| *Rps10* | NM_025963.1 | ribosomal protein S10 (Rps10) | 0.54 |
| *Rps11* | NM_013725.3 | ribosomal protein S11 (Rps11) | 0.55 |
| *Rps12* | NM_011295.2 | | 0.44 |
| *Rps13* | NM_026533.1 | ribosomal protein S13 (Rps13) | 0.46 |
| *Rps15* | NM_009091.1 | ribosomal protein S15 (Rps15) | 0.50 |
| *Rps16* | NM_013647.1 | ribosomal protein S16 (Rps16) | 0.42 |
| *Rps19* | NM_023133.1 | ribosomal protein S19 (Rps19) | 0.62 |
| *Rps2* | NM_008503.4 | ribosomal protein S2 (Rps2) | 0.52 |
| *Rps24* | NM_011297.2 | ribosomal protein S24 (Rps24), transcript variant 1 | 0.61 |
| *Rps25* | NM_024266.3 | ribosomal protein S25 (Rps25) | 0.50 |
| *Rps26* | NM_013765.1 | ribosomal protein S26 (Rps26) | 0.38 |
| *Rps27a* | NM_001033865.1 | ribosomal protein S27a (Rps27a), transcript variant 2 | 0.48 |
| *Rps27l* | NM_026467.2 | ribosomal protein S27-like (Rps27l) | 0.64 |
| *Rps29* | NM_009093.1 | ribosomal protein S29 (Rps29) | 0.61 |
| *Rps3* | NM_012052.1 | | 0.51 |
| *Rps3a* | NM_016959.2 | ribosomal protein S3a (Rps3a) | 0.56 |
| *Rps4x* | NM_009094.1 | ribosomal protein S4, X-linked (Rps4x) | 0.45 |
| *Rps5* | NM_009095.1 | ribosomal protein S5 (Rps5) | 0.43 |
| *Rps6* | NM_009096.2 | ribosomal protein S6 (Rps6) | 0.53 |
| *Rps6ka1* | NM_009097.1 | ribosomal protein S6 kinase polypeptide 1 (Rps6ka1) | 0.55 |
| *Rps7* | NM_011300.2 | ribosomal protein S7 (Rps7) | 0.49 |
| *Rps8* | NM_009098.2 | ribosomal protein S8 (Rps8) | 0.40 |
| *Rps9* | NM_029767.2 | ribosomal protein S9 (Rps9) | 0.61 |
| *Rrbp1* | NM_133626.2 | ribosome binding protein 1 (Rrbp1), transcript variant 2 | 0.22 |
| *Rrm1* | NM_009103.2 | ribonucleotide reductase M1 (Rrm1) | 0.33 |
| *Rrm2* | NM_009104.1 | ribonucleotide reductase M2 (Rrm2) | 0.30 |
| *Rrn3* | NM_001039521.1 | RRN3 RNA polymerase I transcription factor homolog (yeast) (Rrn3) | 0.62 |
| *Rrp15* | NM_026041.2 | ribosomal RNA processing 15 homolog (S. cerevisiae) (Rrp15) | 0.55 |
| *Rrp1b* | NM_028244.1 | ribosomal RNA processing 1 homolog B (S. cerevisiae) (Rrp1b) | 0.66 |
| *Rsl1d1* | NM_025546.2 | ribosomal L1 domain containing 1 (Rsl1d1) | 0.52 |
| *Rtkn2* | NM_001081346.1 | rhotekin 2 (Rtkn2) | 0.50 |
| *Rtn3* | NM_001003934.1 | reticulon 3 (Rtn3), transcript variant 1 | 0.63 |
| *Rtn4ip1* | NM_130892.2 | reticulon 4 interacting protein 1 (Rtn4ip1) | 0.66 |
| *Rtn4rl1* | NM_177708.5 | reticulon 4 receptor-like 1 (Rtn4rl1) | 0.53 |
| *Ruvbl2* | NM_011304.3 | RuvB-like protein 2 (Ruvbl2) | 0.65 |
| *Rwdd1* | NM_025614.1 | RWD domain containing 1 (Rwdd1) | 0.61 |
| *Rybp* | NM_019743.3 | RING1 and YY1 binding protein (Rybp) | 0.63 |
| *Ryk* | NM_001042607.1 | receptor-like tyrosine kinase (Ryk), transcript variant 2 | 0.46 |
| *Sacm1l* | NM_030692.1 | SAC1 (suppressor of actin mutations 1, homolog)-like (S. cerevisiae) (Sacm1l) | 0.55 |
| *Sae1* | NM_019748.2 | SUMO1 activating enzyme subunit 1 (Sae1) | 0.55 |
| *Sall3* | NM_178280.3 | sal-like 3 (Drosophila) (Sall3) | 0.54 |
| *Sap30* | NM_021788.1 | sin3 associated polypeptide (Sap30) | 0.42 |
| *Saps3* | NM_029456.1 | SAPS domain family, member 3 (Saps3) | 0.66 |
| *Satb1* | NM_009122.1 | special AT-rich sequence binding protein 1 (Satb1) | 0.43 |
| *Sav1* | NM_022028.2 | salvador homolog 1 (Drosophila) (Sav1) | 0.65 |
| *Sbk* | NM_145587.1 | | 0.57 |
| *Sc4mol* | NM_025436.1 | sterol-C4-methyl oxidase-like (Sc4mol) | 0.58 |
| *Scamp4* | NM_019575.4 | secretory carrier membrane protein 4 (Scamp4) | 0.59 |
| *Scara3* | NM_172604.3 | scavenger receptor class A, member 3 (Scara3) | 0.52 |
| *Scara5* | NM_028903.1 | scavenger receptor class A, member 5 (putative) (Scara5) | 0.54 |
| *Scd1* | NM_009127.3 | stearoyl-Coenzyme A desaturase 1 (Scd1) | 0.57 |
| *scl0001118.1_0* | AK002910.1 | | 0.58 |
| *scl0001849.1_2273* | AK031598.1 | | 0.30 |
| *scl0001978.1_6* | AK050665.1 | | 0.53 |
| *scl0002315.1_12* | XM_358429.1 | | 0.61 |
| *scl0002507.1_236* | AK083573.1 | | 0.56 |
| *scl0002624.1_576* | AK087461.1 | | 0.55 |
| *scl0002791.1_134* | | | 0.63 |

| | | | |
|---|---|---|---|
| *scl0004020.1_31* | AK081191.1 | | 0.66 |
| *Scml4* | NM_172938.2 | sex comb on midleg-like 4 (Drosophila) (Scml4) | 0.46 |
| *Scoc* | NM_001039137.2 | short coiled-coil protein (Scoc), transcript variant 1 | 0.55 |
| *Scye1* | NM_007926.2 | small inducible cytokine subfamily E, member 1 (Scye1) | 0.67 |
| *Sdc3* | NM_011520.3 | syndecan 3 (Sdc3) | 0.27 |
| *Sdhb* | NM_023374.3 | succinate dehydrogenase complex, subunit B, iron sulfur (Ip) (Sdhb), nuclear gene encoding mitochondrial protein | 0.59 |
| *Sdhd* | NM_025848.2 | succinate dehydrogenase complex, subunit D, integral membrane protein (Sdhd), nuclear gene encoding mitochondrial protein | 0.60 |
| *Sdpr* | NM_138741.1 | serum deprivation response (Sdpr) | 0.36 |
| *Sec11c* | NM_025468.2 | SEC11 homolog C (S. cerevisiae) (Sec11c) | 0.65 |
| *Sec13* | NM_024206.4 | SEC13 homolog (S. cerevisiae) (Sec13) | 0.56 |
| *Sec61g* | NM_011343.2 | SEC61, gamma subunit (Sec61g) | 0.66 |
| *Sec63* | NM_153055 | | 0.64 |
| *Seh1l* | NM_001039088.1 | SEH1-like (S. cerevisiae (Seh1l), transcript variant 1 | 0.66 |
| *Sema3a* | NM_009152.2 | sema domain, immunoglobulin domain (Ig), short basic domain, secreted, (semaphorin) 3A (Sema3a) | 0.57 |
| *Sema4b* | NM_013659.3 | sema domain, immunoglobulin domain (Ig), transmembrane domain (TM) and short cytoplasmic domain, (semaphorin) 4B (Sema4b) | 0.50 |
| *Sema5a* | NM_009154.1 | | 0.48 |
| *Septin 11* | NM_001009818.1 | septin 11 (Sept11) | 0.31 |
| *Septin 6* | NM_019942.2 | septin 6 (Sept6) | 0.53 |
| *Septin 9* | NM_017380.1 | septin 9 (Sept9) | 0.12 |
| *Serbp1* | NM_025814.1 | Serpine1 mRNA binding protein 1 (Serbp1) | 0.47 |
| *Serpine2* | NM_009255.2 | serine (or cysteine) peptidase inhibitor, clade E, member 2 (Serpine2) | 0.40 |
| *Set* | AK011630 | | 0.58 |
| *Setd8* | NM_030241.2 | SET domain containing (lysine methyltransferase) 8 (Setd8) | 0.64 |
| *Sf3b3* | NM_133953.2 | splicing factor 3b, subunit 3 (Sf3b3) | 0.57 |
| *Sf3b4* | NM_153053.3 | splicing factor 3b, subunit 4 (Sf3b4) | 0.53 |
| *Sfrs1* | NM_173374.3 | splicing factor, arginine/serine-rich 1 (ASF/SF2) (Sfrs1), transcript variant 1 | 0.53 |
| *Sfrs10* | NM_009186.4 | splicing factor, arginine/serine-rich 10 (transformer 2 homolog, Drosophila) (Sfrs10) | 0.47 |
| *Sfrs11* | NM_001093753.1 | splicing factor, arginine/serine-rich 11 (Sfrs11), transcript variant 1 | 0.50 |
| *Sfrs14* | NM_172755.2 | splicing factor, arginine/serine-rich 14 (Sfrs14) | 0.66 |
| *Sfrs2* | NM_011358.1 | splicing factor, arginine/serine-rich 2 (SC-35) (Sfrs2) | 0.51 |
| *Sfrs2ip* | NM_028148.2 | splicing factor, arginine/serine-rich 2, interacting protein (Sfrs2ip) | 0.67 |
| *Sfrs4* | NM_020587.1 | splicing factor, arginine/serine-rich 4 (SRp75) (Sfrs4) | 0.63 |
| *Sfrs9* | NM_025573.3 | splicing factor, arginine/serine rich 9 (Sfrs9) | 0.50 |
| *Sfxn1* | NM_027324.4 | sideroflexin 1 (Sfxn1) | 0.55 |
| *Sgk1* | NM_011361.1 | serum/glucocorticoid regulated kinase 1 (Sgk1) | 0.32 |
| *Sgol1* | NM_028232.1 | shugoshin-like 1 (S. pombe) (Sgol1) | 0.49 |
| *Sgol2* | NM_199007.1 | shugoshin-like 2 (S. pombe) (Sgol2) | 0.35 |
| *Sgpp1* | NM_030750.2 | sphingosine-1-phosphate phosphatase 1 (Sgpp1) | 0.57 |
| *Sh3gl2* | NM_019535.2 | SH3-domain GRB2-like 2 (Sh3gl2) | 0.48 |
| *Sh3glb2* | NM_139302.1 | SH3-domain GRB2-like endophilin B2 (Sh3glb2) | 0.52 |
| *Sh3kbp1* | NM_021389.3 | SH3-domain kinase binding protein 1 (Sh3kbp1) | 0.63 |
| *Sh3pxd2b* | NM_177364.3 | SH3 and PX domains 2B (Sh3pxd2b) | 0.47 |
| *Shcbp1* | NM_011369.2 | Shc SH2-domain binding protein 1 (Shcbp1) | 0.66 |
| *Shmt1* | NM_009171 | | 0.35 |
| *Skil* | NM_001039090.1 | SKI-like (Skil), transcript variant 2 | 0.44 |
| *Skiv2l2* | NM_028151.2 | superkiller viralicidic activity 2-like 2 (S. cerevisiae) (Skiv2l2) | 0.51 |
| *Skp2* | NM_145468.1 | S-phase kinase-associated protein 2 (p45) (Skp2), transcript variant 2 | 0.31 |
| *Slain2* | NM_153567.1 | SLAIN motif family, member 2 (Slain2) | 0.66 |
| *Slc12a2* | NM_009194.2 | solute carrier family 12, member 2 (Slc12a2) | 0.62 |
| *Slc1a4* | NM_018861.2 | solute carrier family 1 (glutamate/neutral amino acid transporter), member 4 (Slc1a4) | 0.47 |
| *Slc24a3* | NM_053195.2 | solute carrier family 24 (sodium/potassium/calcium exchanger), member 3 (Slc24a3) | 0.44 |
| *Slc25a11* | NM_024211.2 | solute carrier family 25 (mitochondrial carrier oxoglutarate carrier), member 11 (Slc25a11), nuclear gene encoding mitochondrial protein | 0.63 |
| *Slc25a25* | NM_146118.2 | solute carrier family 25 (mitochondrial carrier, phosphate carrier), member 25 (Slc25a25), nuclear gene encoding mitochondrial protein | 0.30 |
| *Slc25a3* | NM_133668.2 | solute carrier family 25 (mitochondrial carrier, phosphate carrier), member 3 (Slc25a3) | 0.59 |
| *Slc25a39* | NM_026542.1 | solute carrier family 25, member 39 (Slc25a39) | 0.58 |
| *Slc25a5* | NM_007451.3 | solute carrier family 25 (mitochondrial carrier, adenine nucleotide translocator), member 5 (Slc25a5), nuclear gene encoding mitochondrial protein | 0.54 |
| *Slc29a1* | NM_022880.1 | solute carrier family 29 (nucleoside transporters), member 1 (Slc29a1) | 0.47 |
| *Slc30a5* | NM_022885.2 | solute carrier family 30 (zinc transporter), member 5 (Slc30a5) | 0.58 |

| | | | |
|---|---|---|---|
| *Slc31a1* | NM_175090.3 | solute carrier family 31, member 1 (Slc31a1) | 0.52 |
| *Slc35c2* | NM_144893.1 | solute carrier family 35, member C2 (Slc35c2) | 0.61 |
| *Slc35e1* | NM_177766.2 | solute carrier family 35, member E1 (Slc35e1) | 0.57 |
| *Slc35f3* | NM_175434.3 | solute carrier family 35, member F3 (Slc35f3) | 0.45 |
| *Slc38a10* | NM_024249.4 | solute carrier family 38, member 10 (Slc38a10) | 0.61 |
| *Slc39a6* | NM_139143.2 | solute carrier family 39 (metal ion transporter), member 6 (Slc39a6) | 0.55 |
| *Slc41a2* | NM_177388.3 | solute carrier family 41, member 2 (Slc41a2) | 0.54 |
| *Slc4a4* | NM_018760.1 | solute carrier family 4 (anion exchanger), member 4 (Slc4a4) | 0.50 |
| *Slc4a7* | XM_147798.4 | | 0.38 |
| *Slc6a1* | NM_178703.2 | solute carrier family 6 (neurotransmitter transporter, GABA), member 1 (Slc6a1 | 0.64 |
| *Slc6a6* | NM_009320.3 | solute carrier family 6 (neurotransmitter transporter, taurine), member 6 (Slc6a6) | 0.36 |
| *Slitrk5* | NM_198865.1 | SLIT and NTRK-like family, member 5 (Slitrk5) | 0.64 |
| *Slmo2* | NM_025531.2 | slowmo homolog 2 (Drosophila) (Slmo2) | 0.47 |
| *Smap2* | NM_133716.2 | stromal membrane-associated GTPase-activating protein 2 (Smap2) | 0.66 |
| *Smc1a* | NM_019710.1 | structural maintenance of chromosomes 1A (Smc1a) | 0.56 |
| *Smc5l1* | NM_153808 | | 0.66 |
| *Smn1* | NM_011420.2 | survival motor neuron 1 (Smn1) | 0.55 |
| *Smtn* | NM_013870.2 | smoothelin (Smtn) | 0.29 |
| *Smyd2* | NM_026796.1 | SET and MYND domain containing 2 (Smyd2) | 0.67 |
| *Snapc4* | NM_172339.2 | small nuclear RNA activating complex, polypeptide 4 (Snapc4) | 0.45 |
| *Snca* | NM_009221.2 | synuclein, alpha (Snca), transcript variant 2 | 0.49 |
| *Snd1* | NM_019776.2 | staphylococcal nuclease and tudor domain containing 1 (Snd1) | 0.65 |
| *Snora65* | NR_002898.1 | small nucleolar RNA, H/ACA box 65 (Snora65) on chromosome 2. | 0.60 |
| *Snrpb* | NM_009225.2 | small nuclear ribonucleoprotein B (Snrpb) | 0.53 |
| *Snrpd1* | NM_009226.4 | small nuclear ribonucleoprotein D1 (Snrpd1) | 0.53 |
| *Snrpd2* | NM_026943.1 | small nuclear ribonucleoprotein D2 (Snrpd2) | 0.63 |
| *Snrpd3* | NM_026095.4 | small nuclear ribonucleoprotein D3 (Snrpd3) | 0.64 |
| *Snrpf* | XM_892627.2 | PREDICTED: small nuclear ribonucleoprotein polypeptide F (Snrpf) | 0.41 |
| *Snx9* | NM_025664.5 | sorting nexin 9 (Snx9) | 0.61 |
| *Socs5* | NM_019654.2 | suppressor of cytokine signaling 5 (Socs5) | 0.38 |
| *Sorcs1* | NM_021377.1 | VPS10 domain receptor protein SORCS 1 (Sorcs1) | 0.36 |
| *Sorcs2* | NM_030889.2 | sortilin-related VPS10 domain containing receptor 2 (Sorcs2) | 0.54 |
| *Sox11* | NM_009234.5 | SRY-box containing gene 11 (Sox11) | 0.24 |
| *Sox12* | NM_011438.2 | SRY-box containing gene 12 (Sox12) | 0.57 |
| *Sox5* | NM_011444.1 | SRY-box containing gene 5 (Sox5) | 0.56 |
| *Sox8* | NM_011447.1 | SRY-box containing gene 8 (Sox8) | 0.43 |
| *Spag5* | NM_017407.1 | sperm associated antigen 5 (Spag5) | 0.18 |
| *Spag7* | NM_172561.1 | sperm associated antigen 7 (Spag7) | 0.63 |
| *Spast* | NM_016962.1 | spastin (Spast) | 0.57 |
| *Spata6* | NM_026470.3 | spermatogenesis associated 6 (Spata6) | 0.63 |
| *Spc25* | NM_025565.1 | SPC25, NDC80 kinetochore complex component, homolog (S. cerevisiae) (Spc25) | 0.30 |
| *Spcs3* | NM_029701.1 | signal peptidase complex subunit 3 homolog (S. cerevisiae) (Spcs3) | 0.53 |
| *Specc1* | NM_001029936.2 | sperm antigen with calponin homology and coiled-coil domains 1 (Specc1) | 0.29 |
| *Spg7* | NM_153176.4 | spastic paraplegia 7 homolog (human) (Spg7), nuclear gene encoding mitochondrial protein | 0.49 |
| *Sphk1* | NM_011451.2 | sphingosine kinase 1 (Sphk1), transcript variant 1 | 0.46 |
| *Spna2* | XM_207079.2 | | 0.57 |
| *Spp1* | NM_009263.1 | secreted phosphoprotein 1 (Spp1) | 0.26 |
| *Spred1* | NM_033524 | | 0.45 |
| *Spry4* | NM_011898.2 | sprouty homolog 4 (Drosophila) (Spry4) | 0.30 |
| *Spsb4* | NM_145134.2 | splA/ryanodine receptor domain and SOCS box containing 4 (Spsb4) | 0.24 |
| *Sqle* | NM_009270.3 | squalene epoxidase (Sqle) | 0.62 |
| *Srm* | NM_009272.2 | spermidine synthase (Srm) | 0.45 |
| *Srpk1* | NM_016795.3 | serine/arginine-rich protein specific kinase 1 (Srpk1) | 0.66 |
| *Srprb* | NM_009275.4 | signal recognition particle receptor, B subunit (Srprb) | 0.65 |
| *Srrm2* | NM_175229.3 | serine/arginine repetitive matrix 2 (Srrm2) | 0.42 |
| *Ss18* | NM_009280.1 | synovial sarcoma translocation, Chromosome 18 (Ss18) | 0.60 |
| *Ssbp4* | XM_356132.1 | | 0.59 |
| *Ssna1* | NM_023464.2 | Sjogren's syndrome nuclear autoantigen 1 (Ssna1) | 0.65 |
| *Sspn* | NM_010656.2 | sarcospan (Sspn) | 0.65 |
| *Ssr3* | NM_026155.1 | signal sequence receptor, gamma (Ssr3) | 0.43 |
| *Sssca1* | NM_020491.4 | Sjogren's syndrome/scleroderma autoantigen 1 homolog (human) (Sssca1) | 0.62 |
| *St13* | NM_133726.2 | suppression of tumorigenicity 13 (St13) | 0.67 |
| *St6gal1* | NM_145933.3 | beta galactoside alpha 2,6 sialyltransferase 1 (St6gal1) | 0.66 |
| *St6galnac5* | NM_012028.2 | ST6 (alpha-N-acetyl-neuraminyl-2,3-beta-galactosyl-1, 3)-N-acetylgalactosaminide alpha-2,6-sialyltransferase 5 (St6galnac5) | 0.57 |
| *Stab1* | NM_138672.2 | stabilin 1 (Stab1) | 0.35 |
| *Stag2* | NM_001077712.1 | stromal antigen 2 (Stag2), transcript variant 2 | 0.65 |

| | | | |
|---|---|---|---|
| *Stambpl1* | NM_029682.4 | Stam binding protein like 1 (Stambpl1) | 0.32 |
| *Stard4* | NM_133774.4 | StAR-related lipid transfer (START) domain containing 4 (Stard4) | 0.39 |
| *Stc1* | NM_009285.3 | stanniocalcin 1 (Stc1) | 0.32 |
| *Stim2* | NM_001081103.1 | stromal interaction molecule 2 (Stim2) | 0.64 |
| *Stip1* | NM_016737.1 | stress-induced phosphoprotein 1 (Stip1) | 0.45 |
| *Stk17b* | NM_133810.2 | serine/threonine kinase 17b (apoptosis-inducing) (Stk17b) | 0.40 |
| *Stk39* | NM_016866.2 | serine/threonine kinase 39, STE20/SPS1 homolog (yeast) (Stk39) | 0.64 |
| *Stk4* | NM_021420.2 | serine/threonine kinase 4 (Stk4) | 0.67 |
| *Strap* | NM_011499.2 | serine/threonine kinase receptor associated protein (Strap) | 0.56 |
| *Stt3b* | NM_024222.2 | STT3, subunit of the oligosaccharyltransferase complex, homolog B (S. cerevisiae) (Stt3b) | 0.59 |
| *Stx4a* | NM_009294.3 | syntaxin 4A (placental) (Stx4a) | 0.60 |
| *Stxbp1* | NM_009295.1 | syntaxin binding protein 1 (Stxbp1) | 0.53 |
| *Styx* | NM_019637.3 | serine/threonine/tyrosine interaction protein (Styx) | 0.63 |
| *Suclg2* | NM_011507.1 | succinate-Coenzyme A ligase, GDP-forming, beta subunit (Suclg2) | 0.65 |
| *Sumo2* | NM_133354.1 | SMT3 suppressor of mif two 3 homolog 2 (yeast) (Sumo2) | 0.64 |
| *Sumo3* | NM_019929.3 | SMT3 suppressor of mif two 3 homolog 3 (yeast) (Sumo3) | 0.66 |
| *Susd4* | NM_144796.4 | sushi domain containing 4 (Susd4) | 0.64 |
| *Suz12* | NM_199196.1 | suppressor of zeste 12 homolog (Drosophila) (Suz12) | 0.51 |
| *Sv2a* | NM_022030.3 | synaptic vesicle glycoprotein 2 a (Sv2a) | 0.37 |
| *Swap70* | NM_009302.2 | SWA-70 protein (Swap70) | 0.58 |
| *Syncrip* | NM_019666.2 | synaptotagmin binding, cytoplasmic RNA interacting protein (Syncrip), transcript variant 1 | 0.65 |
| *Syt16* | NM_172804.2 | synaptotagmin XVI (Syt16) | 0.62 |
| *Taf10* | NM_020024.3 | TAF10 RNA polymerase II, TATA box binding protein (TBP)-associated factor (Taf10) | 0.57 |
| *Taf12* | BC019668 | | 0.63 |
| *Taf9* | NM_027139.3 | TAF9 RNA polymerase II, TATA box binding protein (TBP)-associated factor (Taf9), transcript variant 1 | 0.56 |
| *Tagap* | NM_145968.1 | T-cell activation Rho GTPase-activating protein (Tagap) | 0.65 |
| *Tanc1* | NM_198294.2 | tetratricopeptide repeat, ankyrin repeat and coiled-coil containing 1 (Tanc1) | 0.50 |
| *Tbc1d1* | NM_019636.2 | TBC1 domain family, member 1 (Tbc1d1) | 0.40 |
| *Tbc1d20* | NM_024196.3 | TBC1 domain family, member 20 (Tbc1d20) | 0.65 |
| *Tbca* | NM_009321.2 | tubulin cofactor a (Tbca) | 0.59 |
| *Tbrg4* | NM_134011.1 | transforming growth factor beta regulated gene 4 (Tbrg4) | 0.38 |
| *Tcerg1* | NM_001039474.1 | transcription elongation regulator 1 (CA150) (Tcerg1) | 0.51 |
| *Tcf12* | NM_011544.2 | transcription factor 12 (Tcf12) | 0.47 |
| *Tcf19* | NM_025674.1 | transcription factor 19 (Tcf19) | 0.61 |
| *Tcf4* | NM_013685.2 | transcription factor 4 (Tcf4), transcript variant 1 | 0.53 |
| *Tcof1* | NM_011552.2 | Treacher Collins Franceschetti syndrome 1, homolog (Tcof1) | 0.67 |
| *Tcp1* | NM_013686.3 | t-complex protein 1 (Tcp1) | 0.59 |
| *Tdg* | NM_011561.1 | thymine DNA glycosylase (Tdg), transcript variant 1 | 0.63 |
| *Tdrkh* | NM_028307.1 | tudor and KH domain containing protein (Tdrkh) | 0.66 |
| *Tead2* | NM_011565.2 | TEA domain family member 2 (Tead2) | 0.32 |
| *Tfdp1* | NM_009361.1 | | 0.44 |
| *Tfg* | NM_019678.2 | Trk-fused gene (Tfg) | 0.63 |
| *Tfrc* | NM_011638.3 | transferrin receptor (Tfrc) | 0.34 |
| *Tgfbr1* | NM_009370.2 | transforming growth factor, beta receptor I (Tgfbr1) | 0.59 |
| *Thoc4* | NM_011568.1 | THO complex 4 (Thoc4) | 0.38 |
| *Thoc7* | NM_025435.2 | THO complex 7 homolog (Drosophila) (Thoc7), transcript variant 1 | 0.53 |
| *Thumpd1* | NM_145585.1 | THUMP domain containing 1 (Thumpd1) | 0.58 |
| *Thy1* | NM_009382.3 | thymus cell antigen 1, theta (Thy1) | 0.36 |
| *Thyn1* | NM_144543.1 | thymocyte nuclear protein 1 (Thyn1) | 0.65 |
| *Timeless* | NM_011589.1 | timeless homolog (Drosophila) (Timeless), transcript variant 2 | 0.39 |
| *Timm10* | NM_013899.1 | translocase of inner mitochondrial membrane 10 homolog (yeast) (Timm10) | 0.60 |
| *Timm17a* | NM_011590.2 | translocase of inner mitochondrial membrane 17a (Timm17a), nuclear gene encoding mitochondrial protein | 0.47 |
| *Timm8a1* | NM_013898.1 | translocase of inner mitochondrial membrane 8 homolog a1 (yeast) (Timm8a1) | 0.60 |
| *Timm8b* | NM_013897.1 | translocase of inner mitochondrial membrane 8 homolog b (yeast) (Timm8b) | 0.50 |
| *Timm9* | NM_013896.3 | translocase of inner mitochondrial membrane 9 homolog (yeast) (Timm9), nuclear gene encoding mitochondrial protein, transcript variant 1 | 0.56 |
| *Timp1* | NM_011593.2 | tissue inhibitor of metalloproteinase 1 (Timp1), transcript variant 2 | 0.35 |
| *Timp4* | NM_080639.3 | tissue inhibitor of metalloproteinase 4 (Timp4) | 0.17 |
| *Tipin* | NM_025372.1 | timeless interacting protein (Tipin) | 0.42 |
| *Tk1* | NM_009387.1 | thymidine kinase 1 (Tk1) | 0.18 |
| *Tlcd1* | NM_026708.1 | TLC domain containing 1 (Tlcd1) | 0.59 |
| *Tle1* | NM_011599.3 | transducin-like enhancer of split 1, homolog of Drosophila E(spl) (Tle1) | 0.57 |
| *Tle2* | NM_019725.1 | transducin-like enhancer of split 2, homolog of Drosophila E(spl) (Tle2) | 0.53 |

| | | | |
|---|---|---|---|
| *Tle6* | NM_053254.2 | transducin-like enhancer of split 6, homolog of Drosophila E(spl) (Tle6) | 0.18 |
| *Tmed3* | NM_025360.1 | transmembrane emp24 domain containing 3 (Tmed3) | 0.55 |
| *Tmed7* | XM_990639.1 | PREDICTED: transmembrane emp24 protein transport domain containing 7 (Tmed7) | 0.59 |
| *Tmed9* | NM_026211.1 | transmembrane emp24 protein transport domain containing 9 (Tmed9) | 0.55 |
| *Tmem109* | NM_134142.1 | transmembrane protein 109 (Tmem109) | 0.60 |
| *Tmem11* | NM_173453.2 | transmembrane protein 11 (Tmem11) | 0.55 |
| *Tmem126a* | NM_025460.1 | transmembrane protein 126A (Tmem126a) | 0.61 |
| *Tmem132a* | NM_133804.2 | transmembrane protein 132A (Tmem132a) | 0.46 |
| *Tmem14c* | NM_025387.1 | transmembrane protein 14C (Tmem14c) | 0.54 |
| *Tmem167* | NM_025335.2 | transmembrane protein 167 (Tmem167) | 0.58 |
| *Tmem176a* | NM_025326.2 | transmembrane protein 176A (Tmem176a) | 0.29 |
| *Tmem176b* | NM_023056.3 | transmembrane protein 176B (Tmem176b) | 0.43 |
| *Tmem184b* | NM_172608.1 | transmembrane protein 184b (Tmem184b) | 0.52 |
| *Tmem199* | NM_199199.2 | transmembrane protein 199 (Tmem199) | 0.58 |
| *Tmem2* | NM_001033759.1 | transmembrane protein 2 (Tmem2), transcript variant 2 | 0.61 |
| *Tmem41a* | NM_025693.3 | transmembrane protein 41a (Tmem41a) | 0.60 |
| *Tmem44* | NM_172614.3 | transmembrane protein 44 (Tmem44) | 0.36 |
| *Tmem48* | NM_028355.3 | transmembrane protein 48 (Tmem48) | 0.47 |
| *Tmem49* | NM_029478.3 | transmembrane protein 49 (Tmem49) | 0.48 |
| *Tmem55b* | NM_001033271.3 | transmembrane protein 55b (Tmem55b) | 0.46 |
| *Tmem56* | NM_178936.3 | transmembrane protein 56 (Tmem56) | 0.61 |
| *Tmem63b* | NM_198167.2 | transmembrane protein 63b (Tmem63b) | 0.51 |
| *Tmem68* | NM_028097.3 | transmembrane protein 68 (Tmem68) | 0.46 |
| *Tmem70* | NM_027415.2 | transmembrane protein 70 (Tmem70), transcript variant 1 | 0.62 |
| *Tmem88* | NM_025915.3 | transmembrane protein 88 (Tmem88) | 0.64 |
| *Tmem97* | NM_133706.2 | transmembrane protein 97 (Tmem97) | 0.33 |
| *Tmpo* | NM_001080129.1 | thymopoietin (Tmpo), transcript variant 3 | 0.61 |
| *Tmsb10* | NM_025284.3 | thymosin, beta 10 (Tmsb10) | 0.53 |
| *Tmsb4x* | NM_021278.2 | thymosin, beta 4, X chromosome (Tmsb4x) | 0.61 |
| *Tnc* | NM_011607.2 | tenascin C (Tnc) | 0.03 |
| *Tnfrsf10b* | NM_020275.3 | tumor necrosis factor receptor superfamily, member 10b (Tnfrsf10b) | 0.52 |
| *Tnfrsf12a* | NM_013749.1 | tumor necrosis factor receptor superfamily, member 12a (Tnfrsf12a) | 0.38 |
| *Tnfrsf21* | NM_178589.2 | tumor necrosis factor receptor superfamily, member 21 (Tnfrsf21) | 0.16 |
| *Tnik* | XM_130797 | | 0.61 |
| *Tnpo3* | NM_177296.4 | transportin 3 (Tnpo3) | 0.62 |
| *Tnrc6a* | NM_144925.3 | trinucleotide repeat containing 6a (Tnrc6a) | 0.62 |
| *Tomm22* | NM_172609.3 | translocase of outer mitochondrial membrane 22 homolog (yeast) (Tomm22), nuclear gene encoding mitochondrial protein | 0.55 |
| *Tomm40l* | NM_001037170.2 | translocase of outer mitochondrial membrane 40 homolog-like (yeast) (Tomm40l), nuclear gene encoding mitochondrial protein | 0.61 |
| *Top1* | NM_009408.2 | topoisomerase (DNA) I (Top1) | 0.66 |
| *Top2a* | NM_011623.1 | topoisomerase (DNA) II alpha (Top2a) | 0.15 |
| *Tpi1* | NM_009415.1 | triosephosphate isomerase 1 (Tpi1) | 0.63 |
| *Tpm3* | NM_022314.2 | tropomyosin 3, gamma (Tpm3) | 0.50 |
| *Tpm4* | NM_001001491.1 | tropomyosin 4 (Tpm4) | 0.46 |
| *Tpp2* | NM_009418.2 | tripeptidyl peptidase II (Tpp2) | 0.59 |
| *Tpst1* | NM_013837.1 | protein-tyrosine sulfotransferase 1 (Tpst1), transcript variant 2 | 0.63 |
| *Tpx2* | NM_028109.3 | TPX2, microtubule-associated protein homolog (Xenopus laevis) (Tpx2) | 0.31 |
| *Traf3* | NM_011632.2 | Tnf receptor-associated factor 3 (Traf3), transcript variant 1 | 0.65 |
| *Traf4* | NM_009423.3 | Tnf receptor associated factor 4 (Traf4) | 0.43 |
| *Trak2* | NM_172406.2 | trafficking protein, kinesin binding 2 (Trak2) | 0.48 |
| *Tram1* | NM_028173.1 | translocating chain-associating membrane protein 1 (Tram1) | 0.57 |
| *Trappc1* | NM_001024206.1 | trafficking protein particle complex 1 (Trappc1) | 0.49 |
| *Trf* | NM_133977.2 | transferrin (Trf) | 0.10 |
| *Trib2* | NM_144551.3 | | 0.20 |
| *Trim27* | NM_009054.2 | tripartite motif-containing 27 (Trim27) | 0.64 |
| *Trim28* | NM_011588.2 | tripartite motif protein 28 (Trim28) | 0.44 |
| *Trim59* | NM_025863.2 | tripartite motif-containing 59 (Trim59) | 0.54 |
| *Trim62* | NM_178110.2 | tripartite motif-containing 62 (Trim62) | 0.44 |
| *Trim8* | NM_053100.1 | tripartite motif protein 8 (Trim8) | 0.57 |
| *Trim9* | NM_053167.1 | tripartite motif protein 9 (Trim9) | 0.67 |
| *Trio* | | | 0.56 |
| *Trip12* | NM_133975.4 | thyroid hormone receptor interactor 12 (Trip12) | 0.62 |
| *Trnt1* | NM_027296.2 | tRNA nucleotidyl transferase, CCA-adding, 1 (Trnt1) | 0.65 |
| *Troap* | XM_912265.2 | PREDICTED: trophinin associated protein (Troap) | 0.31 |
| *Trp53* | NM_011640.2 | transformation related protein 53 (Trp53) | 0.62 |
| *Trp53i11* | NM_001025246.1 | transformation related protein 53 inducible protein 11 (Trp53i11) | 0.30 |
| *Trp53inp2* | NM_178111.3 | transformation related protein 53 inducible nuclear protein 2 (Trp53inp2) | 0.49 |
| *Tsen15* | NM_025677.2 | tRNA splicing endonuclease 15 homolog (S. cerevisiae) (Tsen15) | 0.67 |

| | | | |
|---|---|---|---|
| *Tsfm* | NM_025537.3 | Ts translation elongation factor, mitochondrial (Tsfm), nuclear gene encoding mitochondrial protein | 0.62 |
| *Tspan12* | NM_173007.3 | tetraspanin 12 (Tspan12) | 0.45 |
| *Tspan7* | NM_019634.2 | tetraspanin 7 (Tspan7) | 0.66 |
| *Tssc4* | NM_138631.1 | tumor-suppressing subchromosomal transferable fragment 4 (Tssc4), transcript variant 2 | 0.52 |
| *Ttc13* | XM_358379.1 | | 0.55 |
| *Ttc3* | NM_009441.1 | tetratricopeptide repeat domain 3 (Ttc3) | 0.53 |
| *Ttc7b* | XM_127105.8 | PREDICTED: tetratricopeptide repeat domain 7B, transcript variant 1 (Ttc7b) | 0.51 |
| *Ttyh3* | NM_175274.3 | tweety homolog 3 (Drosophila) (Ttyh3) | 0.21 |
| *Tuba6* | XM_147357.1 | | 0.33 |
| *Tubb5* | NM_011655.4 | tubulin, beta 5 (Tubb5) | 0.39 |
| *Tubb6* | NM_026473.2 | tubulin, beta 6 (Tubb6) | 0.19 |
| *Tubg1* | NM_134024.2 | tubulin, gamma 1 (Tubg1) | 0.45 |
| *Tufm* | NM_172745.2 | Tu translation elongation factor, mitochondrial (Tufm), nuclear gene encoding mitochondrial protein | 0.62 |
| *Txn1* | NM_011660.3 | thioredoxin 1 (Txn1) | 0.28 |
| *Txndc12* | NM_025334.3 | thioredoxin domain containing 12 (endoplasmic reticulum) (Txndc12) | 0.61 |
| *Txnl4a* | NM_025299.3 | thioredoxin-like 4A (Txnl4a), transcript variant 1 | 0.65 |
| *Tyms* | NM_021288.3 | thymidylate synthase (Tyms) | 0.36 |
| *Tyms-ps* | NR_000040.1 | thymidylate synthase, pseudogene (Tyms-ps), non-coding RNA. | 0.39 |
| *U2af1* | NM_024187 | | 0.64 |
| *U2af2* | NM_133671.1 | U2 small nuclear ribonucleoprotein auxiliary factor (U2AF) 2 (U2af2) | 0.64 |
| *Uba1* | NM_009457.3 | ubiquitin-like modifier activating enzyme 1 (Uba1) | 0.56 |
| *Uba2* | NM_016682.2 | ubiquitin-like modifier activating enzyme 2 (Uba2) | 0.49 |
| *Ubap2* | NM_026872.1 | ubiquitin-associated protein 2 (Ubap2) | 0.62 |
| *Ube2a* | NM_019668.3 | ubiquitin-conjugating enzyme E2A, RAD6 homolog (S. cerevisiae) (Ube2a) | 0.54 |
| *Ube2f* | NM_026454.2 | ubiquitin-conjugating enzyme E2F (putative) (Ube2f) | 0.51 |
| *Ube2g1* | NM_025985.4 | ubiquitin-conjugating enzyme E2G 1 (UBC7 homolog, C. elegans) (Ube2g1) | 0.54 |
| *Ube2k* | NM_016786.3 | ubiquitin-conjugating enzyme E2K (UBC1 homolog, yeast) (Ube2k) | 0.58 |
| *Ube2m* | NM_145578.1 | ubiquitin-conjugating enzyme E2M (UBC12 homolog, yeast) (Ube2m) | 0.64 |
| *Ube2q1* | NM_027315.3 | ubiquitin-conjugating enzyme E2Q (putative) 1 (Ube2q1) | 0.65 |
| *Ube2t* | NM_026024.2 | ubiquitin-conjugating enzyme E2T (putative) (Ube2t) | 0.58 |
| *Ube3c* | NM_133907.3 | ubiquitin protein ligase E3C (Ube3c) | 0.58 |
| *Ubr7* | NM_025666.2 | ubiquitin protein ligase E3 component n-recognin 7 (putative) (Ubr7) | 0.54 |
| *Uchl3* | NM_016723.1 | ubiquitin carboxyl-terminal esterase L3 (ubiquitin thiolesterase) (Uchl3) | 0.56 |
| *Uchl5* | NM_019562.1 | ubiquitin carboxyl-terminal esterase L5 (Uchl5) | 0.58 |
| *Uck2* | NM_030724.3 | uridine-cytidine kinase 2 (Uck2) | 0.48 |
| *Ugcg* | NM_011673 | | 0.54 |
| *Uhrf1* | NM_010931.2 | ubiquitin-like, containing PHD and RING finger domains, 1 (Uhrf1) | 0.11 |
| *Uhrf2* | NM_144873.2 | ubiquitin-like, containing PHD and RING finger domains 2 (Uhrf2) | 0.59 |
| *Ung* | NM_011677.2 | uracil DNA glycosylase (Ung), transcript variant 2 | 0.36 |
| *Upf3a* | NM_025924.2 | UPF3 regulator of nonsense transcripts homolog A (yeast) (Upf3a) | 0.59 |
| *Upp1* | NM_009477.1 | uridine phosphorylase 1 (Upp1) | 0.22 |
| *Uqcrc1* | NM_025407.2 | ubiquinol-cytochrome c reductase core protein 1 (Uqcrc1) | 0.57 |
| *Uqcrc2* | NM_025899.2 | ubiquinol cytochrome c reductase core protein 2 (Uqcrc2) | 0.62 |
| *Uqcrh* | NM_025641.3 | ubiquinol-cytochrome c reductase hinge protein (Uqcrh) | 0.54 |
| *Usp1* | NM_146144.2 | ubiquitin specific peptdiase 1 (Usp1) | 0.45 |
| *Usp39* | NM_138592.3 | ubiquitin specific peptidase 39 (Usp39) | 0.62 |
| *Uspl1* | NM_001013378.1 | ubiquitin specific peptidase like 1 (Uspl1) | 0.67 |
| *Utp11l* | NM_026031.3 | UTP11-like, U3 small nucleolar ribonucleoprotein, (yeast) (Utp11l) | 0.55 |
| *Uxt* | NM_013840.2 | ubiquitously expressed transcript (Uxt) | 0.64 |
| *Vamp3* | NM_009498.3 | vesicle-associated membrane protein 3 (Vamp3) | 0.61 |
| *Vapa* | NM_013933.2 | vesicle-associated membrane protein, associated protein A (Vapa) | 0.66 |
| *Vapb* | NM_019806.5 | vesicle-associated membrane protein, associated protein B and C (Vapb) | 0.63 |
| *Vars* | NM_011690.2 | valyl-tRNA synthetase (Vars), nuclear gene encoding mitochondrial protein | 0.53 |
| *Vav3* | NM_020505.2 | vav 3 oncogene (Vav3), transcript variant 1 | 0.21 |
| *Vbp1* | NM_011692.2 | von Hippel-Lindau binding protein 1 (Vbp1) | 0.54 |
| *Vcp* | NM_009503.3 | valosin containing protein (Vcp) | 0.67 |
| *Vdac1* | NM_011694.3 | voltage-dependent anion channel 1 (Vdac1) | 0.55 |
| *Vdac2* | NM_011695.2 | voltage-dependent anion channel 2 (Vdac2) | 0.32 |
| *Vdac3* | NM_011696.1 | voltage-dependent anion channel 3 (Vdac3) | 0.62 |
| *Vegfa* | NM_009505.3 | vascular endothelial growth factor A (Vegfa), transcript variant 2 | 0.41 |
| *Vegfc* | NM_009506.2 | vascular endothelial growth factor C (Vegfc) | 0.52 |
| *Vkorc1l1* | NM_027121.3 | vitamin K epoxide reductase complex, subunit 1-like 1 (Vkorc1l1), transcript variant 1 | 0.49 |
| *Vldlr* | NM_013703.1 | very low density lipoprotein receptor (Vldlr) | 0.47 |
| *Vps29* | NM_019780.1 | vacuolar protein sorting 29 (S. pombe) (Vps29) | 0.53 |
| *Vps36* | NM_027338.1 | vacuolar protein sorting 36 (yeast) (Vps36) | 0.43 |
| *Vps54* | NM_139061.3 | vacuolar protein sorting 54 (yeast) (Vps54) | 0.55 |

| | | | |
|---|---|---|---|
| *Vrk1* | NM_001029843.1 | vaccinia related kinase 1 (Vrk1), transcript variant 2 | 0.53 |
| *Wasf1* | NM_031877.2 | WASP family 1 (Wasf1) | 0.62 |
| *Wasf2* | NM_153423 | | 0.66 |
| *Wbp11* | NM_021714.3 | WW domain binding protein 11 (Wbp11) | 0.58 |
| *Wbscr17* | NM_145218.3 | Williams-Beuren syndrome chromosome region 17 homolog (human) (Wbscr17) | 0.52 |
| *Wdr43* | XM_917905.3 | PREDICTED: WD repeat domain 43, transcript variant 9 (Wdr43) | 0.66 |
| *Wdr51b* | NM_027740.3 | WD repeat domain 51B (Wdr51b) | 0.65 |
| *Wdr74* | NM_134139.1 | WD repeat domain 74 (Wdr74) | 0.65 |
| *Wdr75* | NM_028599.1 | WD repeat domain 75 (Wdr75) | 0.52 |
| *Wdr76* | NM_030234.2 | WD repeat domain 76 (Wdr76) | 0.56 |
| *Wdr82* | NM_029896.1 | WD repeat domain containing 82 (Wdr82) | 0.64 |
| *Wdr89* | XM_001479426.1 | PREDICTED: WD repeat domain 89, transcript variant 2 (Wdr89) | 0.65 |
| *Whsc1* | XM_132006 | | 0.43 |
| *Wipf1* | NM_153138.3 | WAS/WASL interacting protein family, member 1 (Wipf1) | 0.64 |
| *Wsb2* | NM_021539.4 | WD repeat and SOCS box-containing 2 (Wsb2) | 0.31 |
| *Wscd1* | NM_177618.4 | WSC domain containing 1 (Wscd1) | 0.35 |
| *Wwp1* | NM_177327.3 | WW domain containing E3 ubiquitin protein ligase 1 (Wwp1) | 0.62 |
| *Xpnpep1* | NM_133216.2 | X-prolyl aminopeptidase (aminopeptidase P) 1, soluble (Xpnpep1) | 0.59 |
| *Xrcc6* | NM_010247.1 | X-ray repair complementing defective repair in Chinese hamster cells 6 (Xrcc6) | 0.50 |
| *Yeats4* | NM_026570.1 | YEATS domain containing 4 (Yeats4) | 0.52 |
| *Yrdc* | NM_153566.1 | yrdC domain containing (E.coli) (Yrdc) | 0.66 |
| *Ythdf1* | NM_173761.3 | YTH domain family 1 (Ythdf1) | 0.66 |
| *Ythdf2* | NM_145393.3 | YTH domain family 2 (Ythdf2) | 0.66 |
| *Ywhag* | NM_018871.2 | tyrosine 3-monooxygenase/tryptophan 5-monooxygenase activation protein, gamma polypeptide (Ywhag) | 0.62 |
| *Ywhah* | NM_011738.1 | tyrosine 3-monooxygenase/tryptophan 5-monooxygenase activation protein, eta polypeptide (Ywhah) | 0.31 |
| *Zbed4* | NM_181412.3 | zinc finger, BED domain containing 4 (Zbed4) | 0.64 |
| *Zbtb7c* | NM_145356.3 | zinc finger and BTB domain containing 7C (Zbtb7c) | 0.53 |
| *Zc3h13* | NM_026083.2 | zinc finger CCCH type containing 13 (Zc3h13) | 0.61 |
| *Zc3h18* | NM_001029993.1 | zinc finger CCCH-type containing 18 (Zc3h18), transcript variant 1 | 0.65 |
| *Zc3hc1* | NM_172735.1 | zinc finger, C3HC type 1 (Zc3hc1) | 0.66 |
| *Zcchc8* | NM_027494.1 | zinc finger, CCHC domain containing 8 (Zcchc8) | 0.61 |
| *Zdhhc21* | NM_026647.2 | zinc finger, DHHC domain containing 21 (Zdhhc21) | 0.49 |
| *Zdhhc6* | NM_025883.3 | zinc finger, DHHC domain containing 6 (Zdhhc6), transcript variant 2 | 0.59 |
| *Zeb1* | NM_011546.2 | zinc finger E-box binding homeobox 1 (Zeb1) | 0.25 |
| *Zfp106* | NM_011743.2 | zinc finger protein 106 (Zfp106) | 0.62 |
| *Zfp131* | NM_028245.1 | zinc finger protein 131 (Zfp131) | 0.53 |
| *Zfp148* | NM_011749.4 | zinc finger protein 148 (Zfp148) | 0.51 |
| *Zfp207* | NM_011751.2 | zinc finger protein 207 (Zfp207) | 0.65 |
| *Zfp276* | NM_020497 | | 0.66 |
| *Zfp326* | NM_018759.1 | zinc finger protein 326 (Zfp326) | 0.66 |
| *Zfp330* | NM_145600.1 | zinc finger protein 330 (Zfp330) | 0.49 |
| *Zfp365* | NM_178679.2 | zinc finger protein 365 (Zfp365) | 0.38 |
| *Zfp518b* | NM_001081144.1 | zinc finger protein 518B (Zfp518b) | 0.59 |
| *Zfp710* | NM_175433.4 | zinc finger protein 710 (Zfp710) | 0.53 |
| *Zfp770* | NM_175466.2 | zinc finger protein 770 (Zfp770) | 0.49 |
| *Zfpm2* | NM_011766.2 | zinc finger protein, multitype 2 (Zfpm2) | 0.66 |
| *Zmiz1* | NM_183208.2 | zinc finger, MIZ-type containing 1 (Zmiz1) | 0.56 |
| *Znrd1* | NM_023162.4 | zinc ribbon domain containing, 1 (Znrd1) | 0.67 |
| *Znrf2* | NM_199143.1 | zinc and ring finger 2 (Znrf2) | 0.27 |
| *Zswim6* | XM_358311 | | 0.56 |
| *Zwint* | NM_025635.1 | | 0.59 |
| *Zyx* | NM_011777.2 | zyxin (Zyx) | 0.49 |
