# Supplementary material for: miR-30 Family miRNAs Mediate the Effect of Chronic Social Defeat Stress on Hippocampal Neurogenesis in Mouse Depression Model
Source: Front Mol Neurosci. 2019 Aug 8;12:188. doi: 10.3389/fnmol.2019.00188 (PMC6694739; doi:10.3389/fnmol.2019.00188)
Supplement: TABLE S11 — The list comprises of genes which demonstrated increased expression (fold change ≥1.5 and p ≤ 0.05) in the differentiated cells from the late phase (day 7) when compared with the differentiated cells from an early phase (day 2). [file Table_11.pdf]

Table S11. List of genes, which demonstrated increased expression (fold change ≥ 1.5 and p ≤ 0.05) in the differentiated cells from the late phase (day 7) when compared with the differentiated cells from an early phase (day 2)

Gene Symbol	Accession No.	Gene Definition	Fold Change
1700007K13Rik	NM_027040.1	RIKEN cDNA 1700007K13 gene (1700007K13Rik)	1.80
1700027N10Rik	NM_029338.3	RIKEN cDNA 1700027N10 gene (1700027N10Rik)	1.58
2010004M13Rik			1.63
4932425I24Rik	NM_001081025.1	RIKEN cDNA 4932425I24 gene (4932425I24Rik)	1.58
4933426M11Rik	NM_178682.3	RIKEN cDNA 4933426M11 gene (4933426M11Rik)	1.53
5031428M13Rik	AK030317		1.57
9330185J12Rik			1.54
Abhd7	NM_001001804.1	abhydrolase domain containing 7 (Abhd7)	2.13
Acsl6	NM_001033597.1	acyl-CoA synthetase long-chain family member 6 (Acsl6), transcript variant 2	1.62
Adamtsl4	NM_144899.2	ADAMTS-like 4 (Adamtsl4)	1.56
Ankrd50	NM_001033198.2	ankrin repeat domain 50 (Ankrd50)	1.53
Arhgap12	NM_029277.2	Rho GTPase activating protein 12 (Arhgap12), transcript variant 2	1.71
Arsb	NM_009712.3	arylsulfatase B (Arsb)	2.30
AW555464	XM_127132.3		1.61
B230326M20Rik	AK045953		1.67
BC099439	NM_001025564.1	cDNA sequence BC099439 (BC099439)	2.74
Bdh2	NM_027208.1	3-hydroxybutyrate dehydrogenase, type 2 (Bdh2)	1.79
Bicc1	NM_031397.2	bicaudal C homolog 1 (Drosophila) (Bicc1)	1.53
C3	NM_009778.1		12.22
C530044C16Rik			1.93
Ccng1	NM_009831.2	cyclin G1 (Ccng1)	1.56
Cd109	NM_153098.2	CD109 antigen (Cd109)	1.56
Cd59a	NM_007652.2	CD59a antigen (Cd59a)	1.72
Cdkn1a	NM_007669.2	cyclin-dependent kinase inhibitor 1A (P21) (Cdkn1a)	1.51
Chchd10	NM_175329.3	coiled-coil-helix-coiled-coil-helix domain containing 10 (Chchd10)	2.96
Cln3	NM_009907.2	ceroid lipofuscinosis, neuronal 3, juvenile (Batten, Spielmeyer-Vogt disease) (Cln3)	1.70
Col5a1	NM_015734.1	procollagen, type V, alpha 1 (Col5a1)	2.20
Cox6b2	NM_183405.1	cytochrome c oxidase subunit VIb polypeptide 2 (Cox6b2)	1.74
Cp	NM_007752		1.84
Csf1	NM_007778.3	colony stimulating factor 1 (macrophage) (Csf1)	1.50
Ctsa	NM_001038492.1	cathepsin A (Ctsa), transcript variant 2	2.59
Cyb5	NM_025797.3	cytochrome b-5 (Cyb5)	1.56
Cyld	NM_173369.1	cylindromatosis (turban tumor syndrome) (Cyld)	1.50
Dcxr	NM_026428.1	dicarbonyl L-xylulose reductase (Dcxr)	1.63
Dock6	NM_177030.3	dedicator of cytokinesis 6 (Dock6)	1.96
E430002G05Rik	NM_173749		2.16
EG433229	XM_899874.3	PREDICTED: predicted gene, EG433229, transcript variant 7 (EG433229)	1.57
EG665378	NM_001081746.1	predicted gene, EG665378 (EG665378)	1.60
Fzd10	NM_175284.3	frizzled homolog 10 (Drosophila) (Fzd10)	1.50
Gcat	NM_013847		1.79
Gdpd5	NM_201352.2	glycerophosphodiester phosphodiesterase domain containing 5 (Gdpd5)	1.52
Gpx4	NM_001037741.2	glutathione peroxidase 4 (Gpx4), transcript variant 1	1.66
Gria3	NM_016886.2	glutamate receptor, ionotropic, AMPA3 (alpha 3) (Gria3)	1.65
Gtse1	NM_013882.1	G two S phase expressed protein 1 (Gtse1)	1.57
H2-K1	NM_001001892.2	histocompatibility 2, K1, K region (H2-K1), transcript variant 1	1.71
Hdc	NM_008230.4	histidine decarboxylase (Hdc)	1.66
Herpud1	NM_022331.1	homocysteine-inducible, endoplasmic reticulum stress-inducible, ubiquitin-like domain member 1 (Herpud1)	1.89
Hist1h1c	NM_015786		1.67
Hist1h4a	NM_178192.1	histone cluster 1, H4a (Hist1h4a)	1.76
Hist1h4j	NM_178210.1	histone cluster 1, H4j (Hist1h4j)	1.57
Ifit3	NM_010501.2	interferon-induced protein with tetratricopeptide repeats 3 (Ifit3)	1.51
Ifitm1	NM_026820.2	interferon induced transmembrane protein 1 (Ifitm1)	1.74
Igfbpl1	NM_018741.2	insulin-like growth factor binding protein-like 1 (Igfbpl1)	1.66
Il11ra1	NM_010549.2	interleukin 11 receptor, alpha chain 1 (Il11ra1)	1.72
Ivns1abp	NM_054102.2	influenza virus NS1A binding protein (Ivns1abp), transcript variant 2	1.52
Kndc1	NM_177261.4	kinase non-catalytic C-lobe domain (KIND) containing 1 (Kndc1)	1.91
Lbp	NM_008489.2	lipopolysaccharide binding protein (Lbp)	2.41
Lcn2	NM_008491.1	lipocalin 2 (Lcn2)	11.25
Lgals3	NM_010705.2	lectin, galactose binding, soluble 3 (Lgals3)	2.46
LOC100039346	XM_001472500.1	PREDICTED: hypothetical protein LOC100039346 (LOC100039346)	1.54
LOC100039751	XR_031048.1	PREDICTED: similar to 40S ribosomal protein S12 (LOC100039751), misc RNA.	1.64
LOC100042427	XM_001478518.1	PREDICTED: similar to Glyceraldehyde-3-phosphate dehydrogenase (GAPDH), transcript variant 4 (LOC100042427)	1.53

LOC100044948	XM_001473652.1	PREDICTED: similar to macrophage migration inhibitory factor (LOC100044948)	1.91
LOC100047856	XM_001479297.1	PREDICTED: similar to calponin 3, acidic (LOC100047856)	1.63
LOC226017	XM_129164.2		1.76
LOC383712	XM_911155.2	PREDICTED: similar to putative translation initiation factor A121/Sui1 (LOC383712)	1.50
LOC665235	XR_031650.1	PREDICTED: similar to hCG1642689 (LOC665235), misc RNA.	1.74
Lrp2	NM_001081088.1	low density lipoprotein receptor-related protein 2 (Lrp2)	1.81
Mdh1	NM_008618.2	malate dehydrogenase 1, NAD (soluble) (Mdh1)	1.55
Mgst1	NM_019946.4	microsomal glutathione S-transferase 1 (Mgst1)	1.71
Mif	NM_010798.2	macrophage migration inhibitory factor (Mif)	1.87
Nbl1	NM_008675.1	neuroblastoma, suppression of tumorigenicity 1 (Nbl1)	1.86
Ndufb10	XM_128594.4		1.51
Nuak2	NM_028778.3	NUAK family, SNF1-like kinase, 2 (Nuak2)	1.64
Osbpl5	NM_024289		1.71
OTTMUSG00000025408	NR_003564.1	predicted gene, OTTMUSG00000025408 (OTTMUSG00000025408), non-coding RNA.	2.74
Phlda3	NM_013750.1	pleckstrin homology-like domain, family A, member 3 (Phlda3)	1.56
Pltp	NM_011125.2	phospholipid transfer protein (Pltp)	1.55
Por	NM_008898.1	P450 (cytochrome) oxidoreductase (Por)	1.58
Ppib	NM_011149.2	peptidylprolyl isomerase B (Ppib)	1.53
Pqlc3	NM_172574.1	PQ loop repeat containing (Pqlc3)	1.71
Pramel4	NM_178248.2	preferentially expressed antigen in melanoma like 4 (Pramel4)	1.51
Prelp	NM_054077.3	proline arginine-rich end leucine-rich repeat (Prelp)	2.88
Prodh	NM_011172.1		1.58
Pros1	NM_011173.2	protein S (alpha) (Pros1)	1.58
Rarres2	NM_027852.1	retinoic acid receptor responder (tazarotene induced) 2 (Rarres2)	1.65
Serpinb6a	NM_009254.2	serine (or cysteine) peptidase inhibitor, clade B, member 6a (Serpinb6a)	1.52
Serping1	NM_009776		1.55
Shroom3	NM_001077596.1	shroom family member 3 (Shroom3), transcript variant 3	1.65
Siva1	NM_013929.1	SIVA1, apoptosis-inducing factor (Siva1)	1.54
Slc19a2	NM_054087.2	solute carrier family 19 (thiamine transporter), member 2 (Slc19a2)	1.61
Slc2a1	NM_011400.2	solute carrier family 2 (facilitated glucose transporter), member 1 (Slc2a1)	2.33
Slc46a3	NM_027872.3	solute carrier family 46, member 3 (Slc46a3)	1.52
Snta1	NM_009228.1	syntrophin, acidic 1 (Snta1)	1.55
Spag6	NM_015773.1	sperm associated antigen 6 (Spag6)	1.82
Tap2	NM_011530.2	transporter 2, ATP-binding cassette, sub-family B (MDR/TAP) (Tap2)	1.65
Tpi1	NM_009415.1	triosephosphate isomerase 1 (Tpi1)	1.60
Usp53	NM_133857.3	ubiquitin specific peptidase 53 (Usp53)	1.77
Vegfb	NM_011697.2	vascular endothelial growth factor B (Vegfb)	1.50
Xbp1	NM_013842.2	X-box binding protein 1 (Xbp1)	1.57
