# Supplementary material for: miR-30 Family miRNAs Mediate the Effect of Chronic Social Defeat Stress on Hippocampal Neurogenesis in Mouse Depression Model
Source: Front Mol Neurosci. 2019 Aug 8;12:188. doi: 10.3389/fnmol.2019.00188 (PMC6694739; doi:10.3389/fnmol.2019.00188)
Supplement: TABLE S12 — The list comprises of genes which demonstrated decreased expression (fold change ≥1.5 and p ≤ 0.05) in the differentiated cells from the late phase (day 7) when compared with the differentiated cells from an early phase (day 2). [file Table_12.pdf]

Table S12. List of genes, which demonstrated decreased expression (fold change ≥ 1.5 and p ≤ 0.05) in the differentiated cells from the late phase (day 7) when compared with the differentiated cells from an early phase (day 2)

Gene Symbol	Accession No.	Gene Definition	Fold Change
1110003F05Rik			0.65
1190002H23Rik	NM_025427.2	RIKEN cDNA 1190002H23 gene (1190002H23Rik)	0.57
1200009O22Rik	NM_025817.3	RIKEN cDNA 1200009O22 gene (1200009O22Rik)	0.57
1700019G17Rik	NM_029331.2	RIKEN cDNA 1700019G17 gene (1700019G17Rik)	0.66
2210408F11Rik			0.66
2810402K13Rik			0.65
2900062L11Rik	NR_003642.1	RIKEN cDNA 2900062L11 gene (2900062L11Rik), non-coding RNA.	0.60
6330403K07Rik	NM_134022.2	RIKEN cDNA 6330403K07 gene (6330403K07Rik)	0.56
6430537F04			0.61
A230057G18Rik	XM_489103		0.51
Aard	NM_175503.3	alanine and arginine rich domain containing protein (Aard)	0.61
Abhd1	NR_003522.1	abhydrolase domain containing 1 (Abhd1), transcribed RNA.	0.66
Adamts12	NM_175501		0.51
Adamts9			0.51
Agt	NM_007428.3	angiotensinogen (serpin peptidase inhibitor, clade A, member 8) (Agt)	0.48
AI481316	XM_148986.1		0.65
B3gat1	NM_029792.1	beta-1,3-glucuronyltransferase 1 (glucuronosyltransferase P) (B3gat1)	0.50
Bcan	NM_007529.1	brevican (Bcan)	0.48
Brp17	NM_019999.1		0.62
C530008M17Rik	XM_916109.2	PREDICTED: RIKEN cDNA C530008M17 gene, transcript variant 3 (C530008M17Rik)	0.54
C630002C17Rik	AK049826		0.62
Camk2b	NM_007595.3	calcium/calmodulin-dependent protein kinase II, beta (Camk2b)	0.49
Cav1	NM_007616.3	caveolin 1, caveolae protein (Cav1)	0.49
Cd1d1	NM_007639.2	CD1d1 antigen (Cd1d1)	0.66
Cdh13	NM_019707.4	cadherin 13 (Cdh13)	0.57
Cdk5r1	NM_009871.2	cyclin-dependent kinase 5, regulatory subunit 1 (p35) (Cdk5r1)	0.64
Cfl1	NM_007687.2	cofilin 1, non-muscle (Cfl1)	0.65
Chrna4	NM_015730.4	cholinergic receptor, nicotinic, alpha polypeptide 4 (Chrna4)	0.59
Clk1	NM_001042634.1	CDC-like kinase 1 (Clk1), transcript variant 1	0.57
Col4a1	NM_009931.1	procollagen, type IV, alpha 1 (Col4a1)	0.55
Cpxm1	NM_019696.1	carboxypeptidase X 1 (M14 family) (Cpxm1)	0.55
D0H4S114	NM_053078.3	DNA segment, human D4S114 (D0H4S114)	0.51
Ddah1	NM_026993		0.53
Dgkb	NM_178681		0.65
Dpysl4	NM_011993.2	dihydropyrimidinase-like 4 (Dpysl4)	0.65
E030030K01Rik	AK087162		0.54
EG232599	NM_177689.3	predicted gene, EG232599 (EG232599)	0.59
Egr3	NM_018781		0.60
Elmo1	NM_198093.2	engulfment and cell motility 1, ced-12 homolog (C. elegans) (Elmo1), transcript variant 2	0.61
Ephb1	NM_173447.2	Eph receptor B1 (Ephb1)	0.52
F2r	NM_010169.3	coagulation factor II (thrombin) receptor (F2r)	0.66
Fam171b	NM_175514.2	family with sequence similarity 171, member B (Fam171b)	0.50
Fxyd6	NM_022004.6	FXYD domain-containing ion transport regulator 6 (Fxyd6)	0.38
Gamt	NM_010255.1	guanidinoacetate methyltransferase (Gamt)	0.52
Gldc	NM_138595.1	glycine decarboxylase (Gldc)	0.57
Gria2	NM_013540		0.46
Hr	NM_021877.2	hairless (Hr)	0.56
Idb2	AK013239		0.61
Igfbp4	NM_010517.2		0.55
Kcna6	NM_013568.3		0.63
Kcnd2	NM_019697.3	potassium voltage-gated channel, Shal-related family, member 2 (Kcnd2)	0.62
Kctd5	NM_027008.1	potassium channel tetramerisation domain containing 5 (Kctd5)	0.57
Ky	NM_024291.3	kyphoscoliosis peptidase (Ky)	0.62
Lgals8	NM_018886.3	lectin, galactose binding, soluble 8 (Lgals8)	0.64
LOC100041388	XM_001476627.1	PREDICTED: similar to ORF1 (LOC100041388)	0.66
LOC100047606	XR_033719.1	PREDICTED: similar to neurotrophic tyrosine kinase, receptor, type 3 (LOC100047606), misc RNA.	0.64
LOC100047619	XR_033736.1	PREDICTED: similar to solute carrier family 7 (cationic amino acid transporter, y+ system), member 5 (LOC100047619), misc RNA.	0.59
LOC386199	XM_359116.1		0.62
Lpl	NM_008509.2	lipoprotein lipase (Lpl)	0.55
Mest	NM_008590.1	mesoderm specific transcript (Mest)	0.46
Mfge8	NM_001045489.1	milk fat globule-EGF factor 8 protein (Mfge8), transcript variant 2	0.57
mKIAA0282	AK129109		0.53
Mmd2	NM_175217.6	monocyte to macrophage differentiation-associated 2 (Mmd2)	0.61
Myh8	NM_177369.3	myosin, heavy polypeptide 8, skeletal muscle, perinatal (Myh8)	0.46

Nav1	NM_173437.1	neuron navigator 1 (Nav1)	0.65
Ncam1	NM_010875.3	neural cell adhesion molecule 1 (Ncam1), transcript variant 2	0.57
Ncan	NM_007789.2	neurocan (Ncan)	0.43
Neo1	NM_008684.1		0.64
Neto2	NM_001081324.1	neuropilin (NRP) and tolloid (TLL)-like 2 (Neto2)	0.66
Nfia	NM_177176.2		0.66
Olig1	NM_016968.4	oligodendrocyte transcription factor 1 (Olig1)	0.58
Omg	NM_019409.1	oligodendrocyte myelin glycoprotein (Omg)	0.52
P2ry1	NM_008772.4	purinergic receptor P2Y, G-protein coupled 1 (P2ry1)	0.65
Pde1b	NM_008800		0.66
Pdzrn3	NM_018884.1	PDZ domain containing RING finger 3 (Pdzrn3)	0.62
Phf19	NM_028716.1	PHD finger protein 19 (Phf19)	0.66
Plekha4	NM_148927.1	pleckstrin homology domain containing, family A (phosphoinositide binding specific) member 4 (Plekha4)	0.41
Ppic	NM_008908.3	peptidylprolyl isomerase C (Ppic)	0.65
Prkcb	NM_008855.2	protein kinase C, beta (Prkcb)	0.60
Ptn	NM_008973.2	pleiotrophin (Ptn)	0.52
Ptprz1	XM_988289.1	PREDICTED: protein tyrosine phosphatase, receptor type Z, polypeptide 1, transcript variant 3 (Ptprz1)	0.22
Reep3	NM_178606.4	receptor accessory protein 3 (Reep3)	0.60
Scara3	NM_172604.3	scavenger receptor class A, member 3 (Scara3)	0.61
Scara5	NM_028903.1	scavenger receptor class A, member 5 (putative) (Scara5)	0.62
Scd4	NM_183216.3	stearoyl-coenzyme A desaturase 4 (Scd4)	0.46
Scgb1c1	NM_001099742.1	secretoglobin, family 1C, member 1 (Scgb1c1)	0.66
scl0002255.1_1	AK017012.1		0.58
Slc24a3	NM_053195.2	solute carrier family 24 (sodium/potassium/calcium exchanger), member 3 (Slc24a3)	0.42
Slc44a1	NM_133891.2	solute carrier family 44, member 1 (Slc44a1)	0.56
Sox8	NM_011447.1	SRY-box containing gene 8 (Sox8)	0.64
Specc1	NM_001029936.2	sperm antigen with calponin homology and coiled-coil domains 1 (Specc1)	0.64
Svep1	NM_022814.2	sushi, von Willebrand factor type A, EGF and pentraxin domain containing 1 (Svep1)	0.58
Tnfrsf21	NM_178589.2	tumor necrosis factor receptor superfamily, member 21 (Tnfrsf21)	0.43
Trib2	NM_144551.3		0.65
Tspan7	NM_019634.2	tetraspanin 7 (Tspan7)	0.54
Ttyh3	NM_175274.3	tweety homolog 3 (Drosophila) (Ttyh3)	0.46
Wsb1	NM_001042565.2	WD repeat and SOCS box-containing 1 (Wsb1), transcript variant 2	0.56
